# Exploring Metrics to Express Energy Expenditure of Physical Activity in Youth

**DOI:** 10.1371/journal.pone.0130869

**Published:** 2015-06-23

**Authors:** Robert G. McMurray, Nancy F. Butte, Scott E. Crouter, Stewart G. Trost, Karin A. Pfeiffer, David R. Bassett, Maurice R. Puyau, David Berrigan, Kathleen B. Watson, Janet E. Fulton, For the CDC/NCI/NCCOR Research Group on Energy Expenditure in Children

**Affiliations:** 1 Exercise and Sport Science and Nutrition, University of North Carolina, Chapel Hill, NC, United States of America; 2 USDA/ARS Children’s Nutrition Research Center, Baylor College of Medicine, Houston, TX, United States of America; 3 Department of Kinesiology, Recreation and Sport Studies, University of Tennessee, Knoxville, TN, United States of America; 4 Exercise and Nutrition Sciences, Institute of Health and Biomedical Innovation, Queensland University of Technology, Brisbane, Australia; 5 Department of Kinesiology, Michigan State University, East Lansing, MI, United States of America; 6 Centers for Disease Control and Prevention/National Cancer Institute/National Collaborative on Childhood Obesity Research, Washington, DC, United States of America; 7 Division of Nutrition, Physical Activity, and Obesity, CDC, Atlanta, GA, United States of America; Karolinska Institutet, SWEDEN

## Abstract

**Background:**

Several approaches have been used to express energy expenditure in youth, but no consensus exists as to which best normalizes data for the wide range of ages and body sizes across a range of physical activities. This study examined several common metrics for expressing energy expenditure to determine whether one metric can be used for all healthy children. Such a metric could improve our ability to further advance the Compendium of Physical Activities for Youth.

**Methods:**

A secondary analysis of oxygen uptake (VO_2_) data obtained from five sites was completed, that included 947 children ages 5 to 18 years, who engaged in 14 different activities. Resting metabolic rate (RMR) was computed based on Schofield Equations [Hum Nutr Clin Nut. 39(Suppl 1), 1985]. Absolute oxygen uptake (ml.min^-1^), oxygen uptake per kilogram body mass (VO_2_ in ml.kg^-1^.min^-1^), net oxygen uptake (VO_2_ – resting metabolic rate), allometric scaled oxygen uptake (VO_2_ in ml.kg^-0.75^.min^-1^) and YOUTH-MET (VO_2_.[resting VO_2_] ^-1^) were calculated. These metrics were regressed with age, sex, height, and body mass.

**Results:**

Net and allometric-scaled VO_2_, and YOUTH-MET were least associated with age, sex and physical characteristics. For moderate-to-vigorous intensity activities, allometric scaling was least related to age and sex. For sedentary and low-intensity activities, YOUTH-MET was least related to age and sex.

**Conclusions:**

No energy expenditure metric completely eliminated the influence of age, physical characteristics, and sex. The Adult MET consistently overestimated EE. YOUTH-MET was better for expressing energy expenditure for sedentary and light activities, whereas allometric scaling was better for moderate and vigorous intensity activities. From a practical perspective, The YOUTH-MET may be the more feasible metric for improving of the Compendium of Physical Activities for Youth.

## Introduction

Physical activity (PA) plays an important role in health and in normal growth and development [1]. Substantial evidence indicates a global decline in PA and a concomitant increase in obesity, although the causal relationships between PA and obesity are likely complex and bidirectional [1,2]. Developing, testing and evaluating individual and environmental interventions and policies designed to increase youth PA would be enhanced if there were a comparable metric for PA applicable to youth differing in age, sex, and body composition. A compendium of youth energy expenditure (EE) values was developed in 2008 [3]. This compendium reports a single metabolic equivalent (MET) value for each activity, averaged for the age, sex and other characteristics and in some cases uses adult-derived values [4,5].

The youth compendium is comparable to that used in adults [4,5] and has the advantage of simplicity. However, the physical and physiological characteristics of young children are considerably different from adults, and even adolescents. The resting metabolic rate (RMR) of young children is greater per kilogram body mass than adolescents [6] and their economy of movement is less than adolescents and adults [7]. Young children are often less skilled than adults at performing certain activities; they may not have developed refined motor skill [7,8], thus greater muscle and antagonistic tension occurs causing increased EE for the activity. Current research does not take the age dependence into consideration when attempting to describe and compare the EE of activities between children and adolescents and adults. An understanding of the effects of age, sex, and body size on the energy expended across a range of physical activities is needed to meaningfully compare children to adolescents.

Absolute EE (kcal^.^min^-1^), similar to oxygen uptake (VO_2_) per min (ml^.^min^-1^), increases with age as a function of body mass or muscle mass. Thus, it is not appropriate to compare absolute EE of children and adolescents of varying sizes. Several approaches have been used to normalize EE values for differences in body mass in order to compare children and adolescents; thus an effort to examine various metrics of youth energy expenditure during different physical activities across a range of ages is important for improving our capacity to compare EE using a common scale.

Approaches for normalizing EE for body mass can be classified into three categories: 1) ratio scaling of EE for body mass [9–15]; 2) using allometric scaling by taking power functions of body mass [12,16–18]; and 3) computing net EE by subtracting the resting metabolic rate from the activity EE [9,15]. Ratio scaling of EE for body mass (ml^.^kg^-1.^min^-1^) has been criticized by Nevill et al. [16,17] because body mass is not the only variable known to influence children’s physiology. In addition, small children compared to adolescents or adults, have disproportionately higher EE per kilogram body mass for activities despite the higher RMR [6,7]. Since skeletal muscle consumes the majority of energy during activity, some researchers have suggested scaling EE per kilogram fat-free mass–kcal^.^kg_FFM_^-1.^min^-1^ [12,19]. However, the proportional composition of fat-free mass, bone, organs and muscle changes as youth ages [8]. Also, the measurement of fat-free mass is impractical to obtain on a population basis.

Researchers have used allometric scaling to adjust EE for size-related changes in physiological functioning and the disproportionate increase in muscle mass with increasing body size [6,16,17,20–22]. Although an allometric scaling power function may work well for a specific activity (e.g. rest, or maximal treadmill running), Glazier et al. [23] have noted that allometric scaling for near-maximal physical activity results in exponents that are closer to one than for lower-intensity activities. In addition, Zakeri et al. [24] noted that allometric scaling can introduce bias into EE estimates, depending on the types of activities performed. Thus, it is questionable whether the use of a single power function will work to normalize EE, for all activities. From a non-researcher’s perspective, the challenge with allometric scaling is that the results are difficult to interpret, and inter-conversion between EE units requires use of exponents and logarithms.

Some studies have used net EE to express the EE of an activity or changes in efficiency with age [3,25,26]. Expressing net EE may be problematic because there is no one consistent metric. Using the net EE calculation in units of ml^.^kg^-1.^min^-1^or ml/min has the same limitations mentioned previously for ratio scaling (ml^.^kg^-1.^min^-1^) or absolute EE (ml/min). Also, as noted previously, as children age, a disproportionate change between RMR and EE of activity EE occurs [27,28].

Another common approach to estimate EE in youth has been to describe the energy cost in terms of metabolic equivalents (MET) [3,4,5,15,27]. The ADULT-MET (activity EE /3.5 ml^.^kg^-1.^min^-1^ or 1 kcal^.^kg^-1.^h^-1^) is unacceptable for youth because the resting value used for the ADULT-MET (3.5 ml^.^kg^-1.^min^-1^) is typically less than the resting values for children (4–7 ml^.^kg^-1.^min^-1^). In addition, the ADULT-MET does not account for the physical changes that occur as children age, such as muscle mass to total mass ratio, leg length to stature ratio, and pubertal changes [7,8] and, hence, inadequately normalizes EE data across a wide range of ages and body sizes. To overcome these problems Harrell et al. [27] suggested a child MET, or YOUTH-MET (MET_y_). However, Harrell et al. have shown that MET_y_ values are somewhat sex-dependent, tend to increase with age for low-intensity activities and decrease for vigorous-intensity activities, and are influenced by the weight and size of an implement being used for the activity (e.g., ball, broom, shovel, vacuum cleaner, basketball, tennis racquet).

Normalizing youth EE to account for differences in growth and development could allow for comparisons of physical activities across ages and sex. Potentially, this might enable researchers to represent the energy cost of a physical activity using a single metric, regardless of the age of the youth, which could be useful for improving the Compendium of Physical Activities for Youth. However, no single study has compared the utility of these normalization approaches across a wide range of activities, in a heterogeneous sample of youth. Therefore, the goal of this study was to compare various approaches to normalizing the EE of children and adolescents for activities ranging from sedentary to vigorous.

## Materials and Methods

### Participants

The initial sample of 583 boys and 477 girls (total = 1060 youth) were obtained from five sites: Baylor College of Medicine [28], Michigan State University [15], Oregon State University [15], University of Massachusetts-Boston [9], and University of North Carolina [27]. The studies were approved by each research center’s Institutional Review Board, and written informed consent from parents and assent from students were obtained before data collection. The participants in these studies ranged in age from 5 to 19 years. The final sample of 933 youth included 512 boys and 421 girls. A total of 127 youth were excluded because they did not provide any data for at least one of the 14 selected activities or they were 19 years of age. The University of North Carolina contributed 43% of the sample, Baylor College of Medicine contributed 18%, and University of Massachusetts-Boston contributed 17%, while Michigan State University and Oregon State University contributed approximately 11% each. The final sample of 933 youth included 512 boys and 421 girls, between the ages of 5 and 18 years (mean = 12±3 y). The sample was 20% African American, 62% Caucasian, 11% Hispanic, and 7% other race/ethnicities. Investigation of the distribution properties indicated the final data were sufficient for parametric procedures.

Each site measured oxygen uptake, although the methodology differed. Baylor College of Medicine used a room respiration calorimeter with one-minute averaging and activities were completed for 10 to 20 minutes depending upon the intensity of the activity. Michigan State University and Oregon State University used calibrated OXYCON breath-by-breath, oxygen uptake systems with one-minute averaging and each activity performed for five minutes with rest between activities. Universities of Massachusetts–Boston (UM-B), and North Carolina (UNC) used calibrated COSMED portable metabolic units to measure breath-by-breath VO_2_ which was averaged per minute. The UM-B measured VO_2_ during the activities for eight minutes, while UNC measured the VO_2_ of each activity for 7 minutes. Further details on the site-specific instrumentation and protocols are available in the original publications (9,15,27,28). For this study all data were obtained under conditions designed to be at steady state as defined by each site. The oxygen uptake data were compared between the five sites for each activity and the data were found to be consistent across sites. Since walking and running protocols differed between sites these data were subject to further analyses as described below in the ‘Data Pooling and Cleaning’ section.

### Data Management

All study sites submitted their participant characteristics (age, sex, height, body mass) and activity-related oxygen uptake (ml O_2_^.^min^-1^) data to the first author. When available, measured resting VO_2_ also was submitted. Individual speeds for walking and running were reported and these speeds were grouped in 0.5 mph (0.8 kph) increments. Body mass index (BMI: kg^.^m^-2^) was computed from measured height (m) and body mass (kg) and BMI percentiles were obtained from CDC growth charts (attp://apps.nccd.cdc.gov/dnpabmi/calculator.aspx). In addition, we estimated RMR using the Schofield prediction equation [6] because RMR was not available on all participants and experimental conditions for measuring RMR differed across the study sites.

### Candidate Metrics

Absolute VO_2_ (ml^.^min^-1^) served as the basis for calculating all other metrics. Two metrics were scaled for body mass (kg): VO_2KG_ (ml^.^kg^-1.^min^-1^) and VO_2ALLOM_ allometrically scaled to body mass (VO_2_^.^kg^-0.75.^min^-1^). Rather than trying to determine an allometric coefficient for each activity that could have varied by activity [24], we chose to raise body mass to the 0.75 power because it is the most common exponent in the literature and has a strong theoretical basis, especially for inter-species and intra-species analyses within a developmental stage [7, 17,22]. We did not scale the VO_2_ for body surface area (ml^.^m^-2.^min^-1^) and height (ml^.^cm^-1.^min^-1^) because neither has been reported for more than a decade [29]. VO_2NET_ was estimated as VO_2_ of the activity in ml^.^kg^-1.^min^-1^, minus the child’s RMR (converted to VO_2_)_._ Two additional metrics were also computed: YOUTH-MET (MET_y_) was estimated as VO_2_ of the activity in ml^.^kg^-1.^min^-1^ divided by the youth’s estimated RMR (converted to VO_2_) and the ADULT-MET (VO_2_ in ml^.^kg^-1.^min^-1^ of the activity divided by 3.5 ml^.^kg^-1.^min^-1^) also was calculated [4,5]. The final list of candidate metrics included the following five metrics: VO_2KG_, VO_2ALLOM_, VO_2NET,_ YOUTH-MET, and ADULT-MET.

### Data Pooling and Cleaning

The statistical analyses for this study comprised three phases–data pooling and cleaning, preliminary analyses, and final analyses. For data pooling and cleaning, we used descriptive statistics (means, standard deviations and 95% confidence limits), t-tests and chi-square analyses were used to assess statistical significance of bivariate association, and graphical procedures (boxplots, histograms) to 1) obtain a general picture of the sample, 2) investigate the distributional properties of the metrics, and 3) ensure each sedentary and aerobic activity was uniquely specific and had > 100 participants that included an age range of at least 8 to 18 years. We also used general linear models to determine whether the use of pooled data was acceptable, meaning the associations between oxygen uptake and age were similar across sites.

In the combined dataset, more than 40 activities were represented. Approximately 3% to 9% of the pooled data, depending on the activity, were outliers, meaning the value differed from the age- and sex-specific mean for a specific activity by more than 2 standard deviations, and they were excluded from the analyses. We retained the most commonly reported sedentary activities (computer games and television viewing). We excluded other sedentary activities, some of which combined several different behaviors (playing board games, internet use, reading, listening to music, hand writing, coloring, math games, and video game playing while sitting) because the inclusion of multiple sedentary activities could result in a bias toward a metric that was most appropriate for low-intensity activities. We also excluded activities (bench press, leg press, and shoveling sand) deemed more anaerobic in nature. We examined the walking and running data and included only those youth who had specific speed data. Two sites reported the VO_2_ for treadmill-walking at a speed of 2.5 mph (4 kph) and running at 4.5 mph (7.2 kph). All five sites reported VO_2_ for walking speed ranging from 2.8 to 3.2 mph (4.5 to 5.1 kph), some on treadmills and other self-paced. This 2.8 to 3.2 mph range will be referred to as “Walk 3 mph”. All four sites also reported self-paced or treadmill running at approximately 5 mph or 8 kph (range 4.8–5.1 mph; 7.7–8.2 kph) in sufficient samples of children that we included this as a separate activity in our analysis (Table 1). Sensitivity testing revealed no significant differences between the sites for each activity. A similar occurrence was noted for running speeds. This resulted in data for 14 activities that were used for further analyses (Table 1). Basketball was the only activity that showed significant (p>0.01) site differences in the association between oxygen uptake and age.

**Table 1:** Activity, sample size, study-site contributing data, and age ranges for each of the activities examined in this study

Activity	Boys (n) / Girls (n)	Study Site*	Age Range (y)
**Sedentary**			
Computer games	210 / 174	B,M,MS,O	5–18
Television viewing	235 / 216	B,M,NC	5–18
**Light-intensity**			
Housework	127 / 116	M,MS,O	5–16
Sweeping	271 / 256	M,MS,NC,O	5–18
Wii Play	108 / 81	B,M	5–18
**Non-weight bearing**			
Cycling ~10 mph	144 / 152	NC	5–18
**Moderate-to-vigorous intensity**			
Aerobics	195 / 182	All	5–17
Dance	110 / 87	B,M	5–18
Walk– 2.5 mph	273 / 249	B,NC	5–18
Walk– 3 mph	185 / 190	B,M,MS,NC,O	5–16
Run– 4.5 mph	171 / 160	B,NC	5–18
Run—5 mph	80 / 54	B,M,MS,O	6–18
**Skilled**			
Basketball	161 / 123	MS,O,NC	7–16
Rope Skipping	145 / 133	NC	8–18

*B = Baylor, M = Massachusetts—Boston, MS = Michigan State, NC = North Carolina, O = Oregon State

### Statistical Analyses

We conducted preliminary analyses to identify a reduced set of metrics (from the five candidate metrics) that were least influenced by the physical characteristics of the children and therefore warranted a more comprehensive investigation. For the preliminary analyses, we calculated the correlation between the metric and age in years for each of the 14 activities, for each candidate metric. The correlations within each metric were averaged across activities (using Fisher’s Z transformation) [30], and then rank-ordered by the averaged correlations. The three correlations with the weakest rankings (i.e., weakest correlation to age) were selected for the final analysis. In addition to comparing the correlations, graphical representations of the associations were also created.

The final analyses were conducted in three steps to: 1) identify the metric that was least associated with age, 2) determine whether the preferred metric was associated with the sex of the child, and 3) visually confirm the degree of variability by age within the reduced set of metrics. In doing so, we conducted a series of linear regression models with each candidate EE metric as the dependent variable and age as the independent variable. The standardized regression coefficient (equivalent to the correlation in the simple linear regression model) measured the magnitude of association between each candidate EE metric and age–for the 14 activities. To test for differences in each of the standardized regression coefficients, we used the chi-square test for heterogeneity of a set of correlations [30,31]. For activities where the global chi-square statistic indicated at least one regression coefficient was significantly different from the others, we examined all pairwise comparisons. Height and mass were not included in the final analyses because of their known correlation with age [12].

To examine whether the association between age and each EE metric varied by sex, we included age and sex as main effect terms and the age-by-sex interaction term in the regression models. A significant interaction term indicated the association between age and the EE metric was different for boys and for girls. A significant sex main effect term indicated that, after controlling for age, the EE metric for boys and girls was different. Finally, we used box-plots to visually confirm the variability and age-dependence of the optimal EE metric(s) for the 14 activities. Due to multiple testing, the global level of significance was set to p <0.01. Using Bonferroni’s adjustment, the level of significance for post hoc pairwise comparisons was set to p< 0.003. Data analyses were conducted with a statistical software package (SAS 9.3 version; SAS, Cary, NC). All data are available as S1 Data entitled “Optimal Metrics Data”.

## Results

### Descriptive Analysis

The number of youth in each age group and their characteristics are presented by sex in Table 2. As expected, the heights of the boys increased as age increased, whereas the heights of the girls increased with age until about 14–15 years of age. Similar trends were noted for body mass and BMI. The final sample was 67.2% normal weight (BMI < 85^th^ percentile), 15.0% overweight (BMI 85^th^- 95^th^ percentile) and 17.8% obese (BMI ≥95^th^ percentile). The proportions in the three BMI categories were similar for both sexes. Overall, age was correlated with height (♂ r = 0.89; ♀ r = 0.80) and body mass (♂ r = 0.72; ♀ r = 0.67), but correlations were lower with BMI (♂ r = 0.39; ♀ r = 0.27).

**Table 2:** Mean ± SD of physical characteristics of the boys (B) and girls (G) presented by age.

	Boys				Girls			
Age (y)	n*	Height (cm)	Body mass (kg)	BMI (kg^.^m^-1^)	n*	Height (cm)	Body mass (kg)	BMI (kg^.^m^-1^)
5	11	114±5	20.8±4.0	15.8±1.8	6	110±6	17.8±1.9	14.8±1.7
6	9	122±5	25.7±4.5	17.4±3.5	13	123±5	27.1±7.5	17.8±4.6
7	25	125±6	27.6±9.6	17.3±4.1	17	124±5	26.3±5.1	17.2±3.2
8	42	133±5	32.5±8.4	18.2±4.0	44	133±7	31.8±9.7	17.7±3.6
9	47	136±6	34.8±10.3	18.7±4.5	43	138±7	35.4±9.0	18.5±3.4
10	52	142±7	39.6±9.4	19.5±3.8	32	145±7	44.7±14.8	20.8±5.2
11	57	149±7	45.9±14.5	20.5±5.3	55	151±8	47.8±13.5	20.8±4.8
12	67	159±9	56.3±17.2	22.1±5.1	57	154±15	51.4±13.5	23.4±18.4
13	70	162±10	55.4±16.3	21.0±5.3	37	160±9	59.5±13.4	23.3±5.2
14	41	170±10	64.2±18.4	22.0±4.8	32	162±7	60.5±18.5	22.8±6.0
15	35	173±8	70.1±15.6	23.3±4.6	23	163±5	67.9±16.9	25.6±6.8
16	23	175±4	71.9±16.8	23.6±5.6	21	166±5	67.6±19.0	24.9±8.3
17	17	177±7	74.9±13.3	24.0±4.4	21	164±5	62.6±12.0	23.4±4.4
18	16	175±7	72.3±14.7	23.6±4.4	20	163±5	61.0±10.0	22.9±3.6

*n = number of participants of that age and sex

SD = Standard deviation; BMI = Body mass index

The means (±SD) for the metrics of VO_2_ for the 14 physical activities are displayed in Table 3 and represent a wide range of values. As expected, values for sedentary activities were lowest, followed by light activities, with the highest values for running. Average EE for playing computer games was higher than EE for watching television, and average EE for playing Wii was lower than EE for housework and sweeping. The non-weight-bearing activity (cycling) was close to the high end of the moderate-intensity level. The skilled activities were performed at a vigorous-intensity level. In general, the mean ADULT-MET values were higher than the MET_y_ values for all activities by an average of 0.9±1.2 METs.

**Table 3:** Descriptive statistics for 14 activities, showing each metric (mean ± SD).

Activit0079	Metrics for Oxygen Uptake					METs	
	Absolute		VO_2KG_	VO_2NET_	VO_2ALLOM_	Youth	Adult
	ml^.^min^-1^		ml^.^kg^-1.^min^-1^	ml^.^kg^-1.^min^-1^	ml^.^kg^-0.75.^min^-1^		
**Sedentary**	** **		** **	** **	** **	** **	** **
Computer	282±76		6.1±1.7	1.9±1.5	16.0±4.0	1.4±0.2	1.8±0.6
Television	244±77		5.0±1.3	0.9±1.2	13.4±3.2	1.2±0.3	1.5±0.4
**Light Intensity**	** **		** **	** **	** **	** **	** **
Housework	582±167		13.3±3.3	9.1±3.2	34.3±7.8	3.0±0.6	3.9±1.1
Sweeping	658±189		14.4±3.9	10.5±4.1	38.0±10.0	3.3±0.6	4.3±1.3
Wii Play	568±230		10.8±3.3	7.2±4.1	29.8±10.4	2.6±0.7	3.3±1.3
**Non-weight bearing **				** **	** **	** **	** **
Cycling,10 mph	1160±385		23.8±5.2	20.1±6.1	63.4±14.8	5.7±1.3	7.0±1.8
**Moderate-to-Vigorous Intensity**				** **	** **	** **	** **
Aerobics	752±292		16.6±4.4	12.6±5.1	43.4±12.1	3.8±1.0	4.9±1.6
Dance	772±328		14.3±4.1	10.8±5.0	39.4±13.0	3.5±1.0	4.3±1.5
Walk, 2.5 mph	736±235		14.7±3.1	10.6±3.1	38.8±7.3	3.5±0.7	4.2±1.0
Walk, 3 mph	965±283		21.8±3.8	17.6±4.2	56.0±9.5	5.0±0.8	6.3±1.3
Run, 4.5 mph	1658±560		34.1±5.7	30.2±6.1	89.8±15.5	8.1±1.6	9.9±1.8
Run, 5 mph	1810±550		36.6±5.7	32.6±6.3	96.6±14.7	8.8±1.4	10.5±1.9
**Skilled Activities**		** **	** **	** **	** **	** **	** **
Basketball	1292±560		30.8±6.9	26.3±7.8	77.7±19.9	6.7±1.7	8.9±2.3
Rope skip	1644±542		34.0±7.2	29.6±7.9	88.3±20.2	8.1±1.7	9.7±2.4

VO_2NET_ = Activity VO_2_(ml^.^kg^-1.^min^-1^)–RMR(ml^.^kg^-1.^min^-1^); MET_y_ = VO_2_(ml^.^kg^-1.^min^-1^) / RMR(ml^.^kg^-1.^min^-1^): RMR specific to age and sex

### Preliminary Analysis

**Fig 1:**
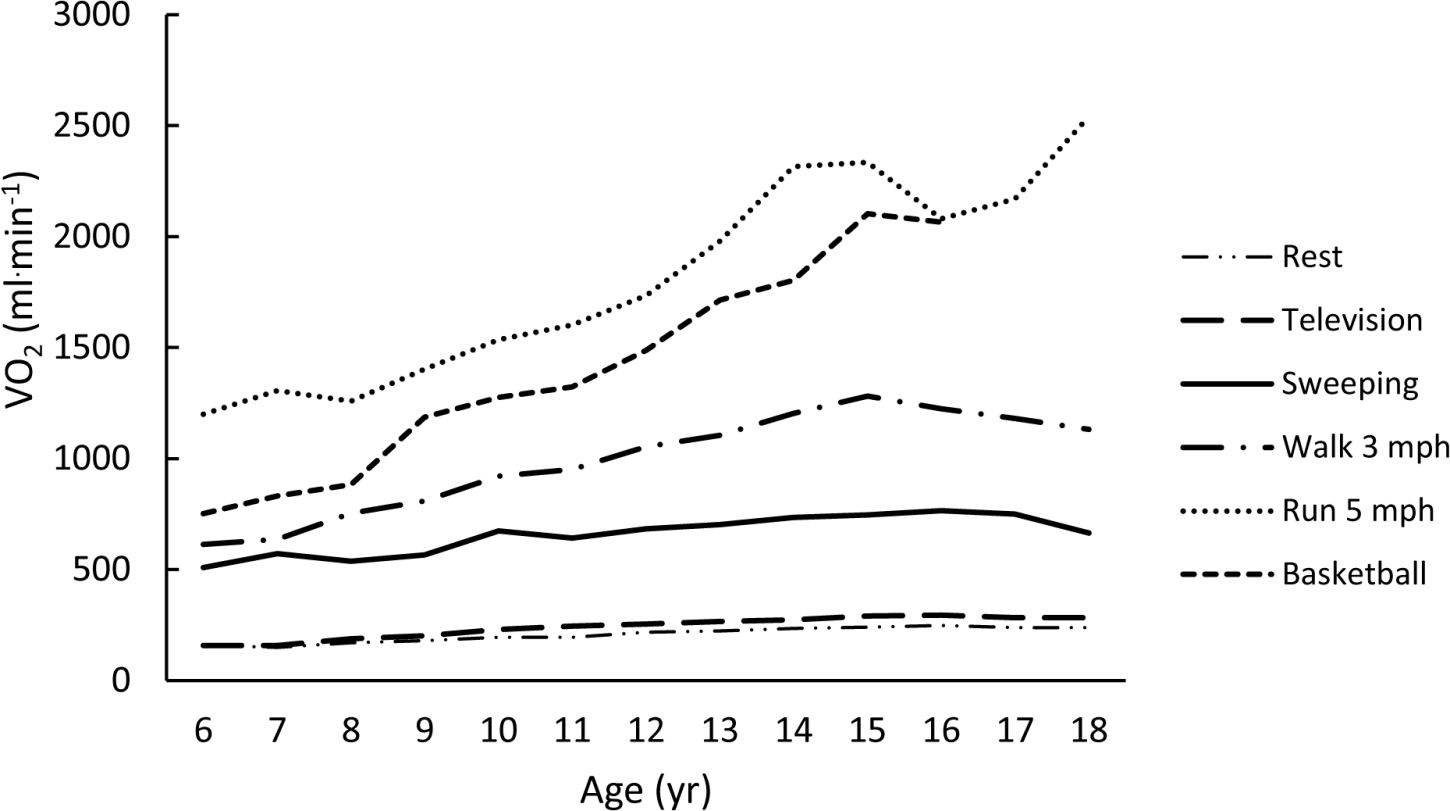
The relationships between age and absolute VO_2_ (ml^.^min^-1^) for six activities representing various intensities of physical activities.

**Fig 2:**
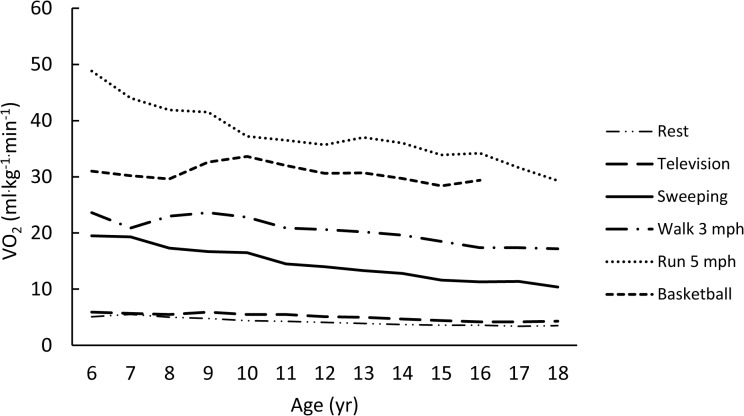
The relationships between age and VO_2KG_ (ml^.^kg^-1.^min^-1^) for six activities representing various intensities of physical activities.

**Fig 3:**
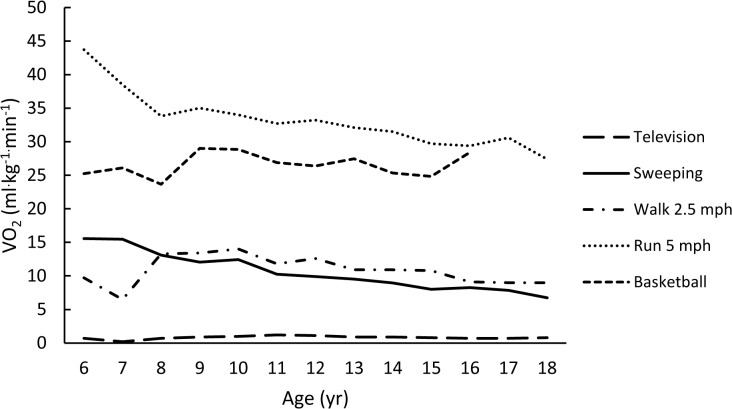
The relationships between age and VO_2NET_ (Activity VO_2_ –RMR; ml^.^kg^-1.^min^-1^) for six activities representing various intensities of physical activities.

**Fig 4:**
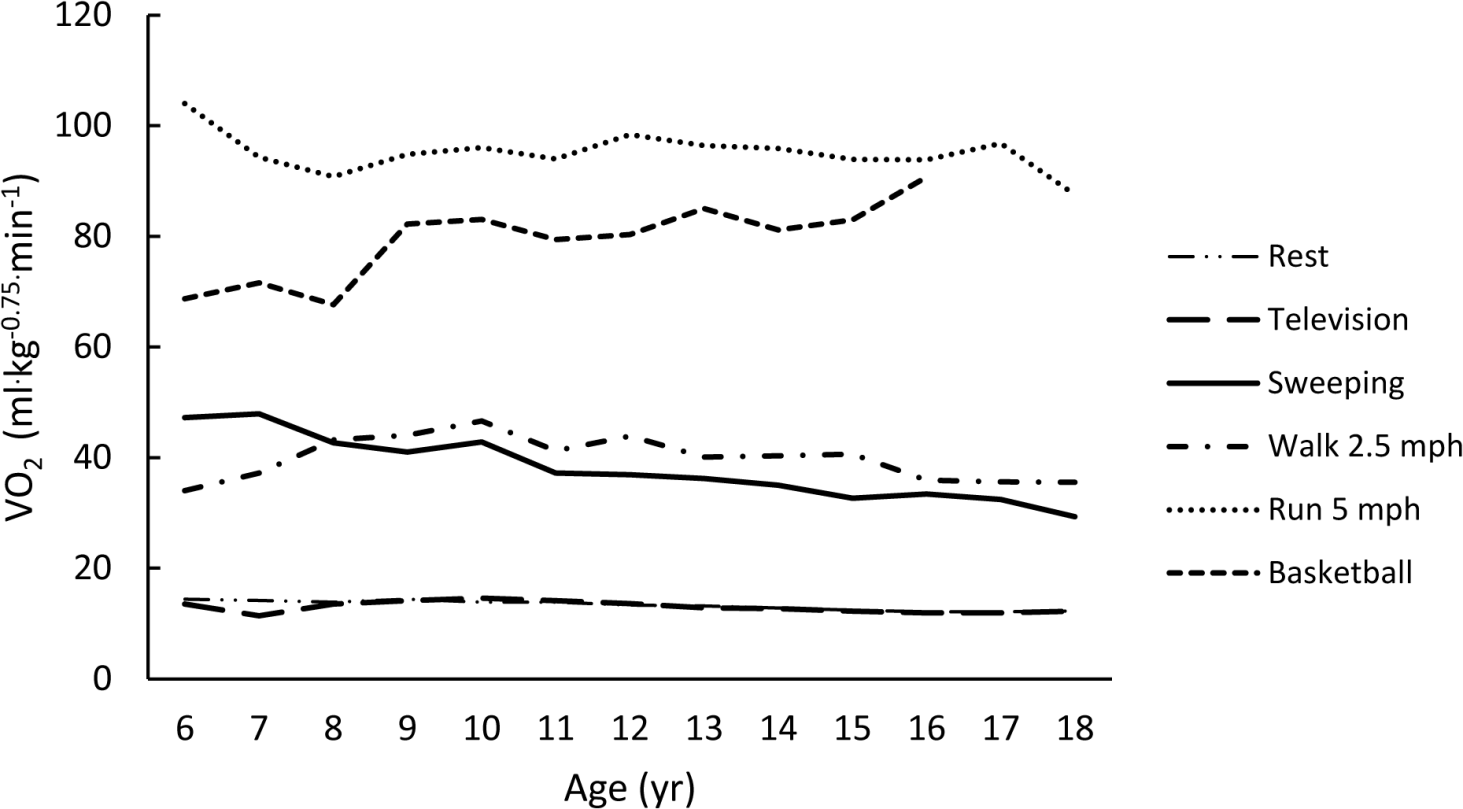
The relationships between age and VO_2ALLOM_ (ml^.^kg^-0.75.^min^-1^) for six activities representing various intensities of physical activities.

**Fig 5:**
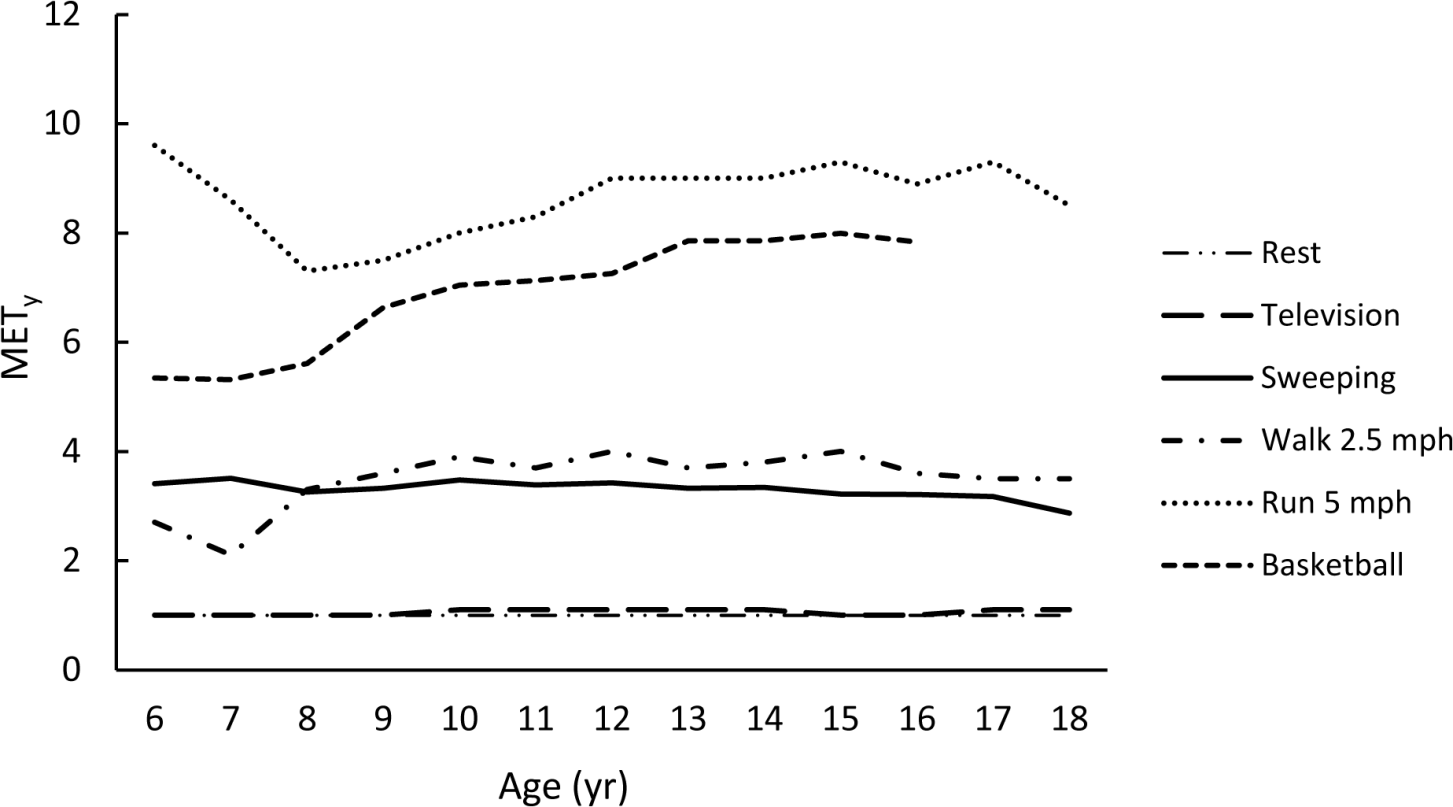
The relationships between age and YOUTH-MET (MET_y_) for six activities representing various intensities of physical activities.

The rank-ordered (weakest to strongest) averaged correlations for each metric with age (using Fisher’s Z transformation) were -0.11, -0.23, 0.28, -0.37, and -0.38 for VO_2NET,_ VO_2ALLOM_, YOUTH-MET, VO_2KG_, and ADULT-MET, respectively. Figs 1–5 are examples of the age-related trends for absolute VO_2_, VO_2KG_, VO_2NET_, VO_2ALLOM_, and MET_y_ for six representative activities of various intensities. The reader should keep in mind that the optimal metric should have slopes that remain horizontal with increasing age. The figures point out that absolute VO_2_ increases with age for all activities (Fig 1), while VO_2KG_ decreases with age for all activities but basketball (Fig 2). Fig 3 shows that the use of VO_2NET_ results in declining values as children age, except for basketball. Similarly, allometric scaling (Fig 4) results in a small decrease in values in older compared to younger children, except for basketball. The MET_y_ data (Fig 5) remain fairly consistent across ages, except for basketball and running. Therefore, the three candidate metrics (VO_2NET_, VO_2ALLOM_, and MET_y_) with the least age-dependency (weakest correlations) were further examined in the final analysis.

### Final Analysis

The standardized regression (correlation) coefficients between age and EE based on the three remaining metrics (VO_2NET_, VO_2ALLOM_, and MET_y_) are presented in Table 4. For sedentary and light-intensity standing activities, MET_y_ was not statistically influenced by age, except for television viewing, in which case the correlation was low (r = 0.149). For non-weight bearing cycling, VO_2NET_ and VO_2ALLOM_ were most independent of age; MET_y_ was positively correlated with age (r = 0.535). For moderate-to-vigorous activities, VO_2ALLOM_ generated the lowest correlations and thus, was least influenced by age. For activities requiring more motor skills (e.g., basketball, skating), the results were inconsistent.

**Table 4:** Standardized regression coefficients for the EE metrics regressed on age for the 14 activities using the three metrics: VO_2NET_, VO_2ALLOM_, and YOUTH-MET (MET_y_).

Activity	VO_2NET_	VO_2ALLOM_	MET_y_
	ml^.^kg^-1.^min^-1^	ml^.^kg^-0.75.^min^-1^	
**Sedentary**			
Computer games	-0.350**	-0.456**	-0.102
Television	-0.010	-0.251**	0.149*
**Light Intensity**			
Housework	-0.400**	-0.306**	0.120
Sweeping	-0.540**	-0.466**	-0.132*
Wii Play	-0.234*	-0.188	0.089
**Non-weight Bearing**			
Cycling ~ 10 mph	-0.101	0.094	**
**Moderate-to-Vigorous Intensity**			
Aerobics	-0.221	-0.087	0.386**
Dance	-0.038	0.048	0.308**
Walk, 2 mph	-0.289**	-0.164*	0.277**
Walk, 3 mph	-0.426**	-0.238**	0.260
Run, 4.5 mph	-0.060	0.253**	0.591**
Run, 5 mph	-0.399**	-0.102	0.291*
**Skilled Activities**			
Basketball	0.095	0.313**	0.604**
Rope Skipping	-0.200*	0.013	0.387**

Standardized regression coefficients are significant at p<0.01 (*) and p <0.001(**)

VO_2NET_ = VO_2_ (ml^.^kg^-1.^min^-1^)–RMR (ml^.^kg^-1.^min^-1^); MET_y_ = VO_2_ (ml^.^kg^-1.^min^-1^) / RMR(ml^.^kg^-1.^min^-1^): RMR specific to age and sex

Results from the activity-specific chi-square test for multiple correlations showed the correlation for at least one metric was significantly different than the other two metrics for all activities (p < 0.001). *Post-hoc* pair-wise comparisons for the remaining activities revealed the standardized regression coefficients between age and MET_y_ were significantly different than those of VO_2ALLOM_ or VO_2NET_ (p < 0.003).

Follow-up analyses revealed that for most activities, the age-by-sex interaction (PROC GLM) was not significant (p > 0.001), indicating that in the majority of cases, that association between the EE metrics and age did not differ by sex. The main effect for sex was not consistently significant; there was only minor evidence of effect modification by sex for the activity of rope skipping (p < 0.05) normalized as VO_2ALLOM_ (R^2^ = 0.051), VO_2NET_ (R^2^ = 0.069), or MET_y_ (R^2^ = 0.019).

## Discussion

Of the EE metrics examined, our findings suggest that no metric completely eliminated the influence of age, height, body mass, or sex for all 14 activities. For most sedentary and light-intensity activities, it appears that MET_y_ is independent of age and sex. For moderate-to-vigorous activities, VO_2ALLOM_ was least influenced by age, but most influenced by sex (p <0.0001). For the more skilled activities the findings were inconsistent, probably related to differences in performance capabilities, experience, or exposure that occurs with age.

The preferred metric for standardizing EE of children is dependent on the type and intensity of the physical activity. Some of the activities were controlled (e.g., walking and running speeds); whereas others were performed at self-selected intensities. These volitional activities would be expected to have greater variation in EE among children because of differences in: 1) the fitness level of the child [32], 2) the child’s perceptions of intensity, 3) the child’s motivation, 4) previous experience with the activity, or 5) the size of the child with respect to any object used to complete the activity. For example, the amount of EE required for a young child (5–7 years of age) who has a body mass of about 30 kg trying to maneuver a vacuum cleaner that has a mass of ~5 kg (~16% of the child’s mass) would be more than a late adolescent of approximately 60 kg accomplishing the same task, since the vacuum cleaner would represent less than 10% of the adolescent’s body mass. Another example would be a small child of about 120 cm in height trying to dribble a full-sized basketball (~24 cm in diameter) compared to an adolescent of 160–170 cm in height accomplishing the same task. A sixth factor affecting the EE of an activity is skill level. Children performing an activity with less skill expend more energy than do those who are more skilled, due to increased muscle activity [7,8]. This may have occurred for aerobics, basketball, dance, or rope skipping. EE variation may also be due to differences between the sexes, which could relate to some of the factors previously mentioned, and small differences in instrumentation and methodologies between the study sites.

In this study, the allometric scaling method for moderate-to-vigorous activities was less dependent on age and other physical characteristics than VO_2NET_ or MET_y_ (Table 4). However, allometric scaling still resulted in declining values with increasing age (Fig 4). There are practical challenges in using an allometric scaling method to estimate youth EE. From a non-researcher’s perspective, the challenge with allometric scaling is that the results are difficult to understand and they increase the complexity of calculating absolute EE for a specific activity for a specific child [24]. Also, as previously mentioned, different scaling factors may be need for each activity [23,24] and Zakeri et al. have noted that the coefficient varies with sex and BMI [24]. Use of multiple factors and coefficients could create confusion when trying to use this metric to predict children’s EE across a wide range of activities. Calculating EE from some power of body mass is not as straightforward as simply using mass. Allometric relationships between EE and size, as well as the underlying distribution of body size could vary in different race/ethnic groups, creating potential difficulties for the generalizability of empirically determined scaling relationships [23,24]. For example, computing absolute EE from two activities with the same allometric VO_2_, but with allometric coefficients for body mass of 0.60 and 0.85 is not a simple task, nor is the results easy to interpret.

Our findings showed that the MET_y_ may be the most favorable metric for sedentary and light-intensity activities. The EE of these activities was only slightly above rest, and therefore the child’s own resting metabolic rate does an acceptable job of standardizing rates of EE. The use of the MET_y_ has been proposed [3,15,27]; however, the MET_y_ may be problematic in that it does not completely adjust for the children’s physical characteristics. Additionally, the mean MET_y_ may underestimate the EE of younger children and overestimate EE in older children for moderate-to-vigorous activities. This is because the greater RMR per unit body mass of the younger children provides a higher denominator in the equation used to calculate the MET_y_ value. For example, the mean MET_y_ for basketball averaged 6.9 METs for all boys, but was 5.1 METs for a boy age 6 years (RMR = 6.6 ml/kg/min), 7.0 for a boy age 11 years (RMR = 4.3 ml/kg/min), and 8.8 for a boy age 16 years (RMR = 3.5 ml/kg/min). Overall, the use of the MET_y_ has limitations for moderate-to-vigorous activities because of the influence of age. The MET_y_ does have an advantage, however, over the other metrics with respect to eliminating any differences related to the sex of the child. The differences between the sexes for all activities were < 0.7 MET_y_.

Early studies by Robinson used oxygen uptake expressed as the metric of ml^.^kg^-1.^min^-1^ to compare the energy cost of treadmill exercise in boys and men [13]. Our results suggest a significant relationship (R^2^ ~33%) between the EE expressed per kilogram body mass and the child’s physical characteristics, including age (Fig 2). This makes sense when one considers that body mass is comprised of different organs and tissues that do not develop at the same rate as the child grows [2,8]. In addition to bone, organ, and muscle mass, fat mass also is a component of total body mass that is not accounted for using the general body mass adjustment. Consequently, adjusting for body mass may not be optimal.

The energy cost of an activity is significantly influenced by RMR and, as children age, their RMR per kilogram body mass declines. Thus, we thought that subtracting RMR from the total energy cost (VO_2NET_) might stabilize the energy cost with respect to the differing physical characteristics of the children. Our results show that this was not the case (Fig 3), as ~17.6% of the variance in VO_2NET_ was accounted for by the physical characteristics and the VO_2NET_ for most activities declined as age increased. Also, the question remains as to what is the proper metric to describe VO_2NET_, because the standard metrics of ml^.^kg^.-1^min^-1^, or ml^.^min^-1^, have many of the same limitations mentioned previously for these two metrics. Thus, the use of VO_2NET_ is not recommended to standardize the EEs of children of differing physical characteristics.

The ADULT-MET (activity VO_2_ ÷ 3.5 ml^.^kg^.-1^min^-1^) has been used to estimate energy expenditure in youth [3], but it is unacceptable because the RMR used for the ADULT-MET (3.5 ml^.^kg^.-1^min^-1^) is typically less than the resting values for children, that can range from 4 to 7 ml^.^kg^.-1^min^-1^. Our results show that applying the ADULT-MET value overestimates the MET_y_ values of most activities by at least one MET_y_, which would lead to an over-estimation of the EE of the activity. In addition, the ADULT-MET does not take into consideration the physical changes that occur as children age [7,8] and, hence is inadequate for normalizing EE data across a wide range of ages and body sizes. Thus, the recommendation is to avoid the use of adult METs when expressing EE for children and adolescents.

The current study has both strengths and weaknesses. It is the first to explore in detail the various common metrics used to express EE in youth, and to compare their ability to normalize EE across a wide range of ages. Strengths include the number of activities, the range of intensities of those activities, and the large number of children in the sample. The large sample also is a weakness, however, as the data were gathered at different sites on unequal distributions of children (age & sex). We did not evaluate the impact of BMI status on the age-dependency of the EE metrics [33]. However, *post hoc* analyses showed that there was a relationship between BMI and EE for all metrics for all activities. Failure to include BMI in our final analyses is a limitation, but it is also a problem with any of the units we examined. Of note, the adult MET system also fails to compensate for weight differences between healthy weight and obese adults. The reader needs to recall that the future development of a youth compendium of activities, similar to the adult compendiums [4,5], will require a compilation of data from studies to be representative of age groups, sex, and the types of physical activities in which all youth engage.

## Conclusions

A common metric to express energy expenditure for sedentary behaviors and physical activities in children and adolescents is needed to evaluate interventions and policies to increase youth physical activity. Our findings show it is difficult to clearly identify a single metric to accurately express the energy expenditure of common activities across a wide age range; thus, there is no optimal metric. In our study, none of the EE metrics we examined completely adjusted for differences in the age, sex, and physical characteristics of children and adolescents. Of the metrics we examined, the MET_y_ (using the Schofield equations to determine RMR and hence the value of one MET_y_) displayed the least age dependence for sedentary and low-intensity activities and it was not influenced by the sex of the child. VO_2ALLOM_ was the best for reducing age-dependence for moderate-to-vigorous intensity activities. Although VO_2ALLOM_ appears to work well for ambulatory activities, it did not work as well for activities that require additional equipment or more skills such as playing basketball. VO_2ALLOM_ coefficients are complex to calculate and a different mass exponent may be required for each activity, making it challenging to directly compare activities across studies. In addition, some target audiences may have difficulty interpreting and utilizing these measures.

The optimal metric for expressing EE may depend on at least two additional issues not addressed in this study–translation and generalizability. The ability of specific target audiences to calculate, interpret, and utilize VO_2ALLOM_ or MET_y_ values is important to consider. These issues are important for the further development and improvement of a Compendium of Physical Activities for Youth. Based on our findings and these considerations, we would recommend the use of the MET_y_ recognizing and accounting for its age-dependency, especially for the more vigorous activities. Age is a key factor related to energy expenditure for youth both at rest and during physical activities and should be accounted for in developing common metrics for various physical activities in children and adolescents.

## Supporting Information

S1 Data Optimal Metrics Data. (PDF)
