# Supplementary material for: Exploring Metrics to Express Energy Expenditure of Physical Activity in Youth
Source: PLoS One. 2015 Jun 23;10(6):e0130869. doi: 10.1371/journal.pone.0130869 (PMC4477976; doi:10.1371/journal.pone.0130869)
Supplement: S1 Data — (PDF) [file pone.0130869.s001.pdf]

ID	activity	Sex	ageyr	Ht	Wt	BMI	BSA
2277	Aerobics	0	5	115.5	20	1.499222	0.798513
2278	Aerobics	0	5	113.5	17.3	1.342933	0.733503
2319	Aerobics	0	5	121.5	28.1	1.903504	0.978184
2282	Aerobics	1	5	107.1	15.8	1.377457	0.682695
2283	Aerobics	1	5	106.8	17.4	1.525481	0.718246
2279	Aerobics	0	6	127.6	25.6	1.572312	0.948617
2231	Aerobics	0	7	121.3	21.6	1.468021	0.848578
2222	Aerobics	0	7	120.6	20.3	1.39573	0.818838
2224	Aerobics	0	7	127.8	25.1	1.536781	0.93919
2322	Aerobics	0	7	135.9	53.3	2.885947	1.442854
2275	Aerobics	1	7	118	33.8	2.427463	1.067888
2261	Aerobics	1	7	120.8	26.2	1.795426	0.939884
2306	Aerobics	0	8	130.3	35	2.06148	1.131733
2324	Aerobics	0	8	133.9	52.2	2.91145	1.418402
2312	Aerobics	0	8	141	52.9	2.660832	1.45816
2221	Aerobics	0	8	140.7	35.2	1.778093	1.170293
2272	Aerobics	0	8	133.5	30.2	1.694511	1.055526
2242	Aerobics	1	8	148.9	57.7	2.602474	1.561263
2276	Aerobics	1	8	136	49	2.649221	1.379439
2204	Aerobics	0	9	134.6	39.4	2.174734	1.221776
2287	Aerobics	0	9	139.6	34.1	1.74978	1.146907
2233	Aerobics	0	9	138.1	30	1.573019	1.06598
2285	Aerobics	0	9	135	33.3	1.82716	1.117417
2321	Aerobics	0	9	133.8	38.3	2.139373	1.200471
2241	Aerobics	1	9	144	60.7	2.927276	1.583267
2320	Aerobics	1	9	132.3	43	2.456681	1.271879
2225	Aerobics	1	9	139.5	30	1.541604	1.07025
2255	Aerobics	1	9	132.3	32.3	1.845367	1.090475
2245	Aerobics	1	9	140	35.7	1.821429	1.176875
2307	Aerobics	0	10	137.3	42.9	2.275706	1.289105
2286	Aerobics	0	10	148.9	63.3	2.855054	1.641008
2247	Aerobics	0	10	140.1	46.69	2.378743	1.359994
2243	Aerobics	0	10	148.3	43.6	1.982459	1.340715
2284	Aerobics	0	10	151.1	50.7	2.220644	1.464853
2294	Aerobics	0	10	139.2	54.2	2.797183	1.469818
2316	Aerobics	0	10	144.6	49	2.343471	1.413378
2216	Aerobics	0	10	151.7	37.8	1.642558	1.252847
2223	Aerobics	1	10	135.8	31.3	1.697246	1.083342
2289	Aerobics	1	10	147.3	73.1	3.369084	1.765512
2230	Aerobics	1	10	147.9	53.2	2.432066	1.490565
2271	Aerobics	1	10	144.3	40.8	1.95942	1.279754
2240	Aerobics	0	11	150.9	78	3.425438	1.845785
2213	Aerobics	0	11	147.6	46.9	2.152782	1.391755
2260	Aerobics	0	11	163.9	71.4	2.65791	1.818688
2280	Aerobics	0	11	146.2	40.4	1.890108	1.279609
2229	Aerobics	0	11	139.4	29.6	1.523232	1.06225

2244	Aerobics	1	11	148.8	66.9	3.021484	1.690107
2270	Aerobics	1	11	153.1	40.4	1.723578	1.303216
2301	Aerobics	1	11	140.4	48	2.435045	1.381555
2207	Aerobics	1	11	149.3	39.8	1.785515	1.279955
2281	Aerobics	1	11	145.6	38	1.792507	1.236133
2296	Aerobics	0	12	161.6	75	2.871961	1.857011
2203	Aerobics	0	12	147.2	46	2.122962	1.375847
2220	Aerobics	0	12	164.6	55.5	2.048488	1.59094
2215	Aerobics	0	12	156.5	59.9	2.445672	1.624748
2256	Aerobics	0	12	163.4	86.9	3.254735	2.018916
2211	Aerobics	1	12	155.9	43.7	1.797998	1.369227
2217	Aerobics	1	12	159.1	54.6	2.157011	1.55591
2227	Aerobics	0	13	145	35.7	1.697979	1.19336
2308	Aerobics	0	13	159.3	84.3	3.321972	1.966295
2274	Aerobics	0	13	140.6	35.6	1.800858	1.177095
2263	Aerobics	0	13	144.3	34.3	1.647257	1.165721
2246	Aerobics	0	13	152	44.4	1.921745	1.36718
2254	Aerobics	0	13	154.7	41.8	1.746609	1.332793
2323	Aerobics	0	13	161.3	96.5	3.709015	2.125031
2202	Aerobics	0	13	148.9	53	2.390487	1.491528
2212	Aerobics	0	13	172.4	72.3	2.432561	1.868049
2315	Aerobics	0	13	157	101.7	4.125928	2.16258
2291	Aerobics	1	13	157.8	78.1	3.13644	1.880086
2232	Aerobics	1	13	153.7	42.1	1.782109	1.334494
2288	Aerobics	1	13	159.8	80	3.132827	1.914079
2299	Aerobics	1	13	161.7	94.6	3.618021	2.104492
2218	Aerobics	0	14	172.8	70.2	2.350984	1.84036
2293	Aerobics	0	14	178.2	89	2.802687	2.116518
2228	Aerobics	0	14	184.6	69.3	2.03362	1.876121
2273	Aerobics	1	14	147.9	46	2.102914	1.378437
2259	Aerobics	1	14	162.7	96.2	3.634126	2.128756
2214	Aerobics	1	14	163.2	59.8	2.24523	1.650488
2252	Aerobics	1	14	171.9	72.3	2.446733	1.865899
2258	Aerobics	1	14	169.4	115.1	4.010962	2.382143
2262	Aerobics	0	15	175.1	61.7	2.012393	1.725974
2300	Aerobics	0	15	182.3	103.5	3.114347	2.31628
2309	Aerobics	0	15	164.4	102	3.773954	2.205916
2201	Aerobics	0	15	162.6	64.3	2.432035	1.713659
2257	Aerobics	0	15	170.8	61.2	2.097856	1.701584
2219	Aerobics	0	15	174.9	61	1.994115	1.714638
2206	Aerobics	1	15	162.5	51.7	1.95787	1.523624
2298	Aerobics	1	15	153.5	107.4	4.558139	2.207129
2205	Aerobics	1	15	156.9	52.2	2.120433	1.510388
2292	Aerobics	1	15	170.2	91.3	3.151749	2.107045
2253	Aerobics	0	16	175.4	57.2	1.859246	1.658216
2290	Aerobics	0	16	171	122.5	4.189323	2.472518
2251	Aerobics	1	16	154.7	130.8	5.465465	2.461534

2311	Aerobics	1	16	166.7	80.9	2.911235	1.95817
2264	Aerobics	0	17	194.2	77.3	2.049656	2.030043
2208	Aerobics	0	17	177	69.8	2.227968	1.852262
2226	Aerobics	0	17	177.2	65.6	2.089183	1.792265
2318	Aerobics	0	17	173.4	112.8	3.751552	2.378325
2209	Aerobics	1	17	164.9	52.5	1.930714	1.545213
2210	Aerobics	1	17	158.2	50.4	2.013806	1.487007
2234	Aerobics	1	17	162.2	46.8	1.77887	1.443121
2305	Aerobics	1	17	166.5	90.9	3.278955	2.083841
2249	Aerobics	1	17	160.2	74.4	2.898998	1.84264
2313	Aerobics	1	17	163.2	81.9	3.074989	1.954639
2314	Aerobics	1	18	161.5	86.6	3.320266	2.005843
2303	Aerobics	1	18	153.9	49.1	2.073024	1.450325
3079	Aerobics	0	5	114.5	21.033	1.604317	0.817612
4054	Aerobics	1	5	120.667	19.967	1.37131	0.811765
4090	Aerobics	1	5	110.567	15.5	1.267887	0.68428
3042	Aerobics	0	6	120.9	23.3	1.594055	0.88271
3078	Aerobics	0	6	114.35	19.5	1.491291	0.784595
4060	Aerobics	0	6	121.633	22.6	1.527586	0.870431
4066	Aerobics	0	6	119.533	21.4	1.497746	0.839446
3103	Aerobics	0	6	129.5	27.225	1.623411	0.986302
4077	Aerobics	1	6	110.867	18.7	1.521378	0.757768
4087	Aerobics	1	6	119.033	31.617	2.231444	1.03379
4073	Aerobics	1	6	121.4	23.7	1.608092	0.892286
3133	Aerobics	1	6	124.55	23.8	1.534227	0.903437
4075	Aerobics	1	6	130.567	28.617	1.678639	1.01641
4047	Aerobics	1	6	127.633	24.3	1.491696	0.922493
3122	Aerobics	1	6	131.4	24.6	1.424769	0.939369
3135	Aerobics	0	7	120.45	23.475	1.61805	0.88496
3108	Aerobics	0	7	123	27.1	1.791262	0.963983
3005	Aerobics	0	7	127	25.067	1.554157	0.936193
3049	Aerobics	0	7	122.35	23.2	1.549816	0.884842
3106	Aerobics	0	7	130.95	41.075	2.395337	1.235899
4029	Aerobics	0	7	128.3	27.05	1.643289	0.979267
4014	Aerobics	0	7	118.7	20.2	1.433672	0.811541
4063	Aerobics	0	7	141.033	55.683	2.799504	1.499066
3028	Aerobics	0	7	127.3	24.55	1.514937	0.926625
4053	Aerobics	0	7	128.567	28.567	1.728246	1.00926
3043	Aerobics	0	7	129	27.475	1.651043	0.989643
3064	Aerobics	0	7	129.2	32.1	1.923003	1.076671
4078	Aerobics	1	7	126.8	25.967	1.61504	0.953527
3080	Aerobics	1	7	116.45	21.35	1.574414	0.829752
4006	Aerobics	1	7	129.333	25.267	1.51055	0.947011
4071	Aerobics	1	7	122	21.617	1.452365	0.850876
3119	Aerobics	0	8	131.8	28.483	1.639664	1.017633
3003	Aerobics	0	8	130.5	32.275	1.895157	1.084118
3054	Aerobics	0	8	135.9	66.433	3.597037	1.624301

4032	Aerobics	0	8	129.767	26.8	1.591499	0.97879
4084	Aerobics	0	8	136.667	27.417	1.467887	1.011404
4021	Aerobics	0	8	132.667	26.583	1.510352	0.983095
3101	Aerobics	0	8	137	36.075	1.922052	1.173389
4097	Aerobics	0	8	138.033	33.75	1.771364	1.135469
3104	Aerobics	0	8	132.167	29.467	1.686902	1.037534
4061	Aerobics	0	8	135.933	31.55	1.707456	1.088409
3033	Aerobics	1	8	125.3	24.7	1.573239	0.923848
4088	Aerobics	1	8	128.033	25.117	1.53223	0.940211
3001	Aerobics	1	8	125.5	24.05	1.52696	0.911269
4030	Aerobics	1	8	131.733	31.583	1.81997	1.075557
4049	Aerobics	1	8	123.367	28.55	1.875893	0.992559
4002	Aerobics	1	8	138.267	33.2	1.736605	1.126235
3066	Aerobics	1	8	132.05	28.975	1.661677	1.02782
3008	Aerobics	1	8	134.6	31.8	1.755242	1.088777
4003	Aerobics	1	8	128.367	21.517	1.305795	0.866047
4035	Aerobics	1	8	157.033	63.383	2.570342	1.677151
3039	Aerobics	1	8	126.55	22.65	1.414308	0.885264
4033	Aerobics	1	8	132.467	32.033	1.825502	1.086161
4034	Aerobics	1	8	131.233	31.333	1.819348	1.069356
3111	Aerobics	0	9	136.5	34.875	1.871755	1.150565
4062	Aerobics	0	9	138.367	39.617	2.069267	1.238872
4005	Aerobics	0	9	134.233	31.9	1.770403	1.089438
4042	Aerobics	0	9	132.967	31.65	1.790136	1.08077
3068	Aerobics	0	9	141.1	38.525	1.935034	1.229887
3100	Aerobics	0	9	128.45	26.925	1.631877	0.977283
3127	Aerobics	0	9	147.5	35.7	1.640908	1.201474
3132	Aerobics	0	9	135.267	32.95	1.800826	1.111955
3091	Aerobics	0	9	140.45	36.875	1.869341	1.199075
3048	Aerobics	1	9	134.367	32.483	1.799165	1.100536
4027	Aerobics	1	9	139.867	41.717	2.132468	1.279223
3061	Aerobics	1	9	138.167	34.2	1.791503	1.144026
4064	Aerobics	1	9	133.233	39.933	2.249616	1.225668
4023	Aerobics	1	9	134.333	30.917	1.713294	1.071569
4104	Aerobics	1	9	133.133	32.633	1.841135	1.099238
4092	Aerobics	1	9	153	61.65	2.633602	1.635377
3056	Aerobics	1	9	134.15	30.375	1.687854	1.060851
4020	Aerobics	1	9	137.2	30.8	1.636223	1.078376
3006	Aerobics	1	9	134.75	29.375	1.617783	1.043768
3072	Aerobics	1	9	132.15	28.2	1.614785	1.013247
3118	Aerobics	1	9	146.95	42.425	1.964639	1.31638
4017	Aerobics	1	9	140.5	46.083	2.334469	1.351984
4102	Aerobics	1	9	136.8	33.217	1.774959	1.121792
3027	Aerobics	0	10	137.75	32.9	1.733855	1.119088
4013	Aerobics	0	10	149.8	45.617	2.03284	1.379214
4069	Aerobics	0	10	133.95	26.45	1.474145	0.984194
3029	Aerobics	0	10	151.85	36.575	1.586188	1.231328

4028	Aerobics	0	10	148.667	50.267	2.274332	1.448759
4055	Aerobics	0	10	147.633	41.6	1.908651	1.304947
4041	Aerobics	0	10	135.467	38.3	2.087045	1.206378
3126	Aerobics	0	10	144	31.3	1.509452	1.108815
3022	Aerobics	0	10	144.8	32.875	1.567937	1.140983
4025	Aerobics	0	10	147.633	34.767	1.595146	1.184907
3070	Aerobics	0	10	140	35.7	1.821429	1.176875
4052	Aerobics	0	10	140.867	42.75	2.154357	1.299828
4040	Aerobics	1	10	150.667	59.333	2.613726	1.592302
3041	Aerobics	1	10	142.45	36.583	1.802828	1.20067
4100	Aerobics	1	10	138.3	34.45	1.801129	1.148954
4070	Aerobics	1	10	138.5	38.483	2.006178	1.220137
3123	Aerobics	1	10	145.45	33.125	1.565772	1.147677
4048	Aerobics	1	10	132.8	24.033	1.362736	0.931568
3114	Aerobics	1	10	140.55	27.5	1.392102	1.024362
4105	Aerobics	1	10	151.533	52.033	2.266024	1.487127
4068	Aerobics	1	10	150.867	56.45	2.480136	1.551029
4081	Aerobics	1	10	142.733	35.6	1.747436	1.184141
3086	Aerobics	0	11	144	38.4	1.851852	1.23768
3102	Aerobics	0	11	149.6	38.05	1.700167	1.250368
4050	Aerobics	0	11	142.533	52.433	2.580916	1.457457
4026	Aerobics	0	11	158.633	61.25	2.433991	1.653187
3128	Aerobics	0	11	145.067	35.917	1.706722	1.197475
3002	Aerobics	0	11	141.55	31.225	1.558413	1.099878
3034	Aerobics	0	11	142.8	42.525	2.085393	1.303165
3052	Aerobics	0	11	154.4	41.9	1.757598	1.333481
4019	Aerobics	0	11	144.633	34.1	1.63012	1.163123
3038	Aerobics	0	11	143.3	34.6	1.684937	1.167969
4037	Aerobics	0	11	138.033	33.6	1.763492	1.132752
4010	Aerobics	0	11	144.567	48.3	2.311047	1.402357
3017	Aerobics	1	11	133.9	29.533	1.6472	1.044161
4096	Aerobics	1	11	155.8	77.033	3.173525	1.856816
3060	Aerobics	1	11	157.75	63.875	2.566801	1.687183
3000	Aerobics	1	11	151	52.275	2.292663	1.488762
4089	Aerobics	1	11	157.5	54.6	2.201058	1.549689
4101	Aerobics	1	11	137.633	28.2	1.488689	1.029708
3120	Aerobics	1	11	157.667	43.3	1.741832	1.368573
4016	Aerobics	1	11	148.7	42.067	1.902478	1.316558
4083	Aerobics	1	11	164.633	61.1	2.254278	1.675485
3009	Aerobics	1	11	164.5	58.367	2.156928	1.63423
4044	Aerobics	1	11	144.333	35.9	1.723309	1.194765
3113	Aerobics	0	12	157.05	62.475	2.532972	1.664258
3134	Aerobics	0	12	164.25	76.775	2.845831	1.892683
4008	Aerobics	0	12	135.9	27.967	1.514283	1.019987
4038	Aerobics	0	12	154.6	37.633	1.574526	1.259285
4039	Aerobics	0	12	155.967	40.733	1.674484	1.318644
4085	Aerobics	0	12	150.533	56.633	2.499229	1.552367

4046	Aerobics	0	12	153.633	36.45	1.544289	1.234763
4108	Aerobics	0	12	135.633	28.783	1.564607	1.035079
3095	Aerobics	0	12	160.9	78.967	3.050237	1.905923
4031	Aerobics	0	12	154.733	52.733	2.202503	1.510313
3058	Aerobics	0	12	175.4	65.95	2.143659	1.790141
3088	Aerobics	0	12	156.5	49.283	2.012188	1.462912
3004	Aerobics	0	12	159.95	48.775	1.906465	1.467412
4076	Aerobics	1	12	156.433	49.467	2.021431	1.465598
3124	Aerobics	1	12	167.65	50.217	1.786667	1.518628
3065	Aerobics	1	12	152.95	33.95	1.451245	1.186373
4109	Aerobics	1	12	157.067	51.433	2.084836	1.499044
3085	Aerobics	1	12	158	49.3	1.974844	1.468727
4098	Aerobics	1	12	158.133	46.733	1.868868	1.427567
4099	Aerobics	1	12	158.9	48.85	1.934714	1.464796
4103	Aerobics	1	12	151.467	40.75	1.776199	1.303722
4106	Aerobics	1	12	157.067	46.983	1.904456	1.427835
4107	Aerobics	1	12	152.767	50.117	2.147464	1.462115
4110	Aerobics	1	12	152.467	41.917	1.803178	1.327128
3094	Aerobics	1	12	168.7	51.575	1.812212	1.544396
4079	Aerobics	1	12	152.133	59.8	2.583773	1.605179
4057	Aerobics	0	13	158.267	47.45	1.894329	1.439788
3131	Aerobics	0	13	159.1	45.875	1.812323	1.416831
4056	Aerobics	0	13	159.533	51.317	2.016323	1.506499
4074	Aerobics	0	13	171.533	62.25	2.115651	1.72014
3087	Aerobics	0	13	167.5	48.275	1.72065	1.486229
3014	Aerobics	0	13	169.8	53.75	1.864245	1.583161
3020	Aerobics	0	13	147.5	33.7	1.54898	1.164793
3024	Aerobics	0	13	173.15	56.45	1.882864	1.638083
4091	Aerobics	0	13	168.5	66.033	2.32574	1.76308
3035	Aerobics	0	13	165.6	47.9	1.746686	1.473331
3115	Aerobics	0	13	152.25	38.525	1.661989	1.26753
3044	Aerobics	0	13	160.233	43.9	1.70986	1.387591
4086	Aerobics	0	13	167.767	83.95	2.982687	2.002591
3110	Aerobics	0	13	169.75	64.033	2.222206	1.739245
4012	Aerobics	0	13	156.467	62.25	2.542693	1.658585
3010	Aerobics	1	13	173.75	67.35	2.23094	1.803709
4022	Aerobics	1	13	158.967	49.317	1.951564	1.472557
3069	Aerobics	1	13	163.25	49.675	1.863938	1.493958
3015	Aerobics	1	13	164.5	45	1.662956	1.420909
4093	Aerobics	1	13	155.333	53.483	2.216605	1.524164
4067	Aerobics	1	13	159.167	59.85	2.362425	1.634933
4094	Aerobics	1	13	167.167	79.983	2.862178	1.948361
4043	Aerobics	1	13	162.8	54.133	2.042459	1.562917
4080	Aerobics	1	13	157.433	52.817	2.130994	1.522007
3021	Aerobics	0	14	159.75	48.225	1.88969	1.457767
4045	Aerobics	0	14	164.7	56.733	2.091455	1.61024
4007	Aerobics	0	14	163.933	51.367	1.911399	1.523632

3117	Aerobics	0	14	162.05	53.35	2.031591	1.547883
3055	Aerobics	0	14	184.6	71.3	2.092311	1.905049
3096	Aerobics	0	14	170.567	106.5	3.660662	2.290931
3081	Aerobics	0	14	179.8	68.05	2.104984	1.838543
3047	Aerobics	0	14	174.3	77.85	2.5625	1.952313
3097	Aerobics	0	14	182.3	97.35	2.929292	2.241213
3053	Aerobics	1	14	170.6	50.125	1.722251	1.527658
3057	Aerobics	1	14	158.4	44.217	1.762296	1.386632
3098	Aerobics	1	14	159.75	44.825	1.756461	1.401561
3025	Aerobics	1	14	156.35	51.275	2.097538	1.493853
4015	Aerobics	1	14	154.667	45.9	1.918745	1.401459
3112	Aerobics	1	14	160.3	69.25	2.694963	1.773347
4051	Aerobics	1	14	158.967	58.9	2.33078	1.620117
4082	Aerobics	1	14	168.133	47.517	1.680905	1.475837
4095	Aerobics	1	14	167.4	86.733	3.095091	2.036257
4024	Aerobics	1	14	175.533	54.767	1.777466	1.62039
3011	Aerobics	1	14	170.25	60.675	2.09332	1.691554
4036	Aerobics	1	14	157	72.7	2.94941	1.805378
4065	Aerobics	0	15	170	77.817	2.69263	1.932636
3023	Aerobics	0	15	174.15	71.4	2.354243	1.86295
4009	Aerobics	0	15	190.4	117.15	3.231532	2.518894
3075	Aerobics	1	15	166.5	68.125	2.457412	1.784437
3093	Aerobics	1	15	166.2	53.925	1.952217	1.572516
3016	Aerobics	1	15	159.4	61.633	2.425698	1.661913
3026	Aerobics	0	16	180.4	71.4	2.193942	1.889171
3032	Aerobics	0	16	170.3	59.7	2.058472	1.677076
3084	Aerobics	1	16	165.65	54.125	1.972491	1.573581
4011	Aerobics	1	16	163.633	62.1	2.319262	1.6861
4058	Aerobics	1	16	165.733	68.533	2.495064	1.786903
1143	Aerobics	0	8	132.6	27.2	1.54697	0.995102
1101	Aerobics	0	8	126	32.3	2.034518	1.069587
1101	Aerobics	0	8	126	32.3	2.034518	1.069587
1146	Aerobics	1	8	124.5	28.6	1.845132	0.997101
1181	Aerobics	1	8	146.2	61.9	2.895982	1.609669
1306	Aerobics	1	8	135.9	26.5	1.434852	0.990855
1152	Aerobics	1	8	123.5	25.4	1.665328	0.932474
1152	Aerobics	1	8	123.5	25.4	1.665328	0.932474
1148	Aerobics	1	8	129.9	40.5	2.400141	1.222656
1184	Aerobics	1	8	134.6	30.5	1.683487	1.064609
1104	Aerobics	0	9	131	25.5	1.485927	0.956542
1151	Aerobics	0	9	128.3	24.4	1.482301	0.926445
1316	Aerobics	0	9	142.7	38.4	1.885746	1.233239
1316	Aerobics	0	9	142.7	38.4	1.885746	1.233239
1314	Aerobics	0	9	134.2	30.9	1.715748	1.070831
1305	Aerobics	0	9	139.1	31.2	1.612502	1.091821
1315	Aerobics	0	9	134.3	27.1	1.50251	0.998161
1312	Aerobics	0	9	143.6	44.3	2.1483	1.335095

1123	Aerobics	0	9	128	23.5	1.434326	0.907065
1140	Aerobics	0	9	124	28.1	1.827523	0.986114
1311	Aerobics	1	9	133.4	27.9	1.567807	1.011202
1311	Aerobics	1	9	133.4	27.9	1.567807	1.011202
1310	Aerobics	1	9	139.9	36.1	1.844471	1.183613
1310	Aerobics	1	9	139.9	36.1	1.844471	1.183613
1308	Aerobics	1	9	135.8	35.1	1.903301	1.1522
1307	Aerobics	1	9	133.4	29.5	1.657717	1.041987
1317	Aerobics	1	9	139.2	29.9	1.543095	1.067419
1304	Aerobics	1	9	136.5	31.9	1.712085	1.096694
1128	Aerobics	1	9	158.7	63.7	2.529214	1.68871
1166	Aerobics	0	10	121	24.8	1.693873	0.91313
1137	Aerobics	0	10	145.7	35	1.648728	1.182975
1169	Aerobics	0	10	146.1	57.4	2.689128	1.54522
1309	Aerobics	0	10	137.8	33.4	1.758928	1.128365
1102	Aerobics	0	10	143	41.8	2.04411	1.291886
1127	Aerobics	1	10	132.8	43.3	2.455228	1.278554
1163	Aerobics	1	10	146.3	36.6	1.709986	1.213733
1286	Aerobics	1	10	163.6	99.4	3.713811	2.171293
1286	Aerobics	1	10	163.6	99.4	3.713811	2.171293
1103	Aerobics	1	10	144	46.8	2.256944	1.376618
1103	Aerobics	1	10	144	46.8	2.256944	1.376618
1255	Aerobics	1	10	154.5	54	2.26223	1.528808
1610	Aerobics	0	11	146.1	45.2	2.117571	1.35888
1327	Aerobics	0	11	146.8	46.3	2.148468	1.379177
1329	Aerobics	0	11	143.1	33.4	1.63105	1.145372
1318	Aerobics	0	11	147.5	33.8	1.553577	1.166651
1190	Aerobics	0	11	156.8	62.4	2.538005	1.662133
1247	Aerobics	0	11	158.3	92.8	3.703277	2.065389
1512	Aerobics	0	11	142.8	36.9	1.809547	1.207428
1330	Aerobics	0	11	156.5	47	1.918974	1.426067
1503	Aerobics	0	11	139.2	31.5	1.625669	1.097767
1135	Aerobics	0	11	151.8	65.1	2.825124	1.678728
1135	Aerobics	0	11	151.8	65.1	2.825124	1.678728
1325	Aerobics	0	11	145.5	39.6	1.870549	1.263513
1113	Aerobics	0	11	144	34.5	1.663773	1.168407
1202	Aerobics	0	11	141.1	27.9	1.401361	1.033948
1541	Aerobics	0	11	148.7	43.5	1.967285	1.340491
1537	Aerobics	0	11	146.4	82.1	3.830549	1.874712
1232	Aerobics	0	11	157.8	54.5	2.188681	1.54933
1285	Aerobics	0	11	153.1	44.3	1.889963	1.369432
1285	Aerobics	0	11	153.1	44.3	1.889963	1.369432
1540	Aerobics	0	11	163.5	63.6	2.379149	1.707332
1535	Aerobics	0	11	148.9	51	2.30028	1.460989
1122	Aerobics	1	11	147	52.5	2.429543	1.476409
1175	Aerobics	1	11	156.6	59.3	2.418083	1.616384
1175	Aerobics	1	11	156.6	59.3	2.418083	1.616384

1124	Aerobics	1	11	149	50	2.252151	1.445897
1124	Aerobics	1	11	149	50	2.252151	1.445897
1577	Aerobics	1	11	143.9	35.9	1.733696	1.193343
1328	Aerobics	1	11	145.5	32	1.511555	1.126701
1328	Aerobics	1	11	145.5	32	1.511555	1.126701
1197	Aerobics	1	11	150.7	54.3	2.390966	1.518307
1188	Aerobics	1	11	149.7	55.1	2.458714	1.526264
1211	Aerobics	1	11	157.2	36.8	1.489165	1.252469
1211	Aerobics	1	11	157.2	36.8	1.489165	1.252469
1217	Aerobics	1	11	139.6	31.1	1.595841	1.091489
1249	Aerobics	1	11	153.1	57.9	2.470178	1.581514
1109	Aerobics	1	11	171.5	89.6	3.04635	2.092155
1109	Aerobics	1	11	171.5	89.6	3.04635	2.092155
1225	Aerobics	1	11	163	62.9	2.367421	1.695141
1192	Aerobics	1	11	156.9	48.1	1.953886	1.445383
1204	Aerobics	1	11	150.1	55.1	2.445627	1.527879
1324	Aerobics	1	11	158.4	48.2	1.921041	1.452466
1324	Aerobics	1	11	158.4	48.2	1.921041	1.452466
1508	Aerobics	1	11	166.6	42.6	1.534828	1.386592
1320	Aerobics	1	11	157.6	70.2	2.826342	1.774401
1320	Aerobics	1	11	157.6	70.2	2.826342	1.774401
1319	Aerobics	1	11	140.1	29.2	1.48767	1.056602
1319	Aerobics	1	11	140.1	29.2	1.48767	1.056602
1106	Aerobics	1	11	154	46.1	1.943835	1.402335
1250	Aerobics	1	11	147.9	42.8	1.956624	1.326008
1509	Aerobics	1	11	143.3	33.3	1.62163	1.14416
1191	Aerobics	0	12	144.7	35.5	1.695475	1.188784
1191	Aerobics	0	12	144.7	35.5	1.695475	1.188784
1576	Aerobics	0	12	164.9	78.9	2.901588	1.923688
1561	Aerobics	0	12	164.8	58.4	2.150297	1.635908
1548	Aerobics	0	12	161.2	80.1	3.082495	1.922
1303	Aerobics	0	12	168.5	55	1.937148	1.597981
1303	Aerobics	0	12	168.5	55	1.937148	1.597981
1110	Aerobics	0	12	159	43	1.700882	1.368023
1110	Aerobics	0	12	159	43	1.700882	1.368023
1206	Aerobics	0	12	162.8	56.3	2.12422	1.59626
1206	Aerobics	0	12	162.8	56.3	2.12422	1.59626
1536	Aerobics	0	12	153.7	49.5	2.095354	1.455917
1220	Aerobics	0	12	145.5	38.6	1.823313	1.246252
1323	Aerobics	0	12	144.7	41.1	1.96293	1.286217
1598	Aerobics	0	12	161.7	59	2.256483	1.63259
1114	Aerobics	0	12	162	62.8	2.392928	1.689564
1114	Aerobics	0	12	162	62.8	2.392928	1.689564
1547	Aerobics	0	12	156.1	55	2.257132	1.550288
1511	Aerobics	0	12	163.4	72.1	2.700419	1.826043
1552	Aerobics	0	12	154.6	55.4	2.317879	1.550395
1578	Aerobics	0	12	164.7	88.6	3.266228	2.04648

1115	Aerobics	0	12	161	68.9	2.658076	1.771578
1564	Aerobics	0	12	160.9	80.2	3.097864	1.92187
1322	Aerobics	0	12	155.4	52.4	2.169848	1.507745
1614	Aerobics	0	12	170.7	78.1	2.680304	1.93957
1235	Aerobics	1	12	153.8	88.6	3.745597	1.99168
1506	Aerobics	1	12	161.2	51.2	1.970334	1.510863
1299	Aerobics	1	12	139.3	33.4	1.721251	1.133218
1233	Aerobics	1	12	154.8	42.1	1.756872	1.338272
1203	Aerobics	1	12	155.6	42.5	1.755374	1.347847
1203	Aerobics	1	12	155.6	42.5	1.755374	1.347847
1550	Aerobics	1	12	159.2	41.4	1.633481	1.341076
1546	Aerobics	1	12	145.4	32.7	1.546746	1.139579
1208	Aerobics	1	12	154.9	48.7	2.029673	1.447671
1321	Aerobics	1	12	57.3	50.9	15.50274	0.999506
1321	Aerobics	1	12	57.3	50.9	15.50274	0.999506
1527	Aerobics	1	12	158.2	86.3	3.448243	1.985786
1524	Aerobics	1	12	154.8	82.7	3.451148	1.924155
1118	Aerobics	1	12	151	59.4	2.605149	1.594664
1216	Aerobics	1	12	143.9	32.2	1.555014	1.125539
1502	Aerobics	1	12	152.6	47.2	2.026903	1.415101
1186	Aerobics	1	12	158.6	61.6	2.448919	1.658124
1186	Aerobics	1	12	158.6	61.6	2.448919	1.658124
1574	Aerobics	1	12	163.4	85.2	3.191064	1.997578
1214	Aerobics	1	12	155.5	46.8	1.935464	1.41919
1604	Aerobics	1	12	154.5	47.9	2.006682	1.433362
1117	Aerobics	1	12	158.7	51.2	2.0329	1.501531
1195	Aerobics	1	12	150.9	41.3	1.813726	1.311205
1229	Aerobics	1	12	151.3	46.6	2.035673	1.40064
1108	Aerobics	1	12	162	63.7	2.427221	1.702544
1525	Aerobics	1	12	154.8	48.9	2.040643	1.450494
1283	Aerobics	0	13	165.5	49.9	1.821816	1.505741
1283	Aerobics	0	13	165.5	49.9	1.821816	1.505741
1112	Aerobics	0	13	164.4	57.1	2.112674	1.614666
1572	Aerobics	0	13	157.8	63.7	2.558147	1.684908
1228	Aerobics	0	13	148.8	49.1	2.217561	1.431079
1228	Aerobics	0	13	148.8	49.1	2.217561	1.431079
1599	Aerobics	0	13	166.2	50.2	1.817363	1.513132
1241	Aerobics	0	13	182.2	65.9	1.985129	1.816596
1241	Aerobics	0	13	182.2	65.9	1.985129	1.816596
1282	Aerobics	0	13	137.8	29.7	1.564077	1.05932
1198	Aerobics	0	13	152.5	44.2	1.900564	1.365641
1198	Aerobics	0	13	152.5	44.2	1.900564	1.365641
1254	Aerobics	0	13	156.7	64	2.606405	1.684493
1326	Aerobics	0	13	154.4	67	2.810478	1.716418
1213	Aerobics	0	13	151.2	40.3	1.762794	1.295053
1245	Aerobics	0	13	175.4	100.5	3.266682	2.245321
1245	Aerobics	0	13	175.4	100.5	3.266682	2.245321

1267	Aerobics	0	13	172.9	58.4	1.953542	1.66732
1267	Aerobics	0	13	172.9	58.4	1.953542	1.66732
1566	Aerobics	0	13	169.8	62.1	2.153854	1.711009
1275	Aerobics	0	13	148.1	38.8	1.768975	1.258526
1521	Aerobics	0	13	160.7	45.8	1.77351	1.421211
1288	Aerobics	0	13	164.5	80.5	2.974843	1.942699
1288	Aerobics	0	13	164.5	80.5	2.974843	1.942699
1168	Aerobics	0	13	172	83.9	2.835992	2.021822
1168	Aerobics	0	13	172	83.9	2.835992	2.021822
1273	Aerobics	0	13	158.7	48.7	1.933638	1.461646
1273	Aerobics	0	13	158.7	48.7	1.933638	1.461646
1272	Aerobics	0	13	163.6	76.8	2.869423	1.890041
1556	Aerobics	0	13	156.8	48.3	1.964513	1.448246
1270	Aerobics	0	13	159.9	56.8	2.221526	1.592481
1270	Aerobics	0	13	159.9	56.8	2.221526	1.592481
1107	Aerobics	1	13	164	57.2	2.12671	1.614626
1187	Aerobics	1	13	143.4	77.4	3.76394	1.801363
1222	Aerobics	1	13	160.5	47.8	1.855572	1.45354
1544	Aerobics	1	13	162.2	67	2.546673	1.75028
1580	Aerobics	1	13	128	44.5	2.716064	1.278693
1569	Aerobics	1	13	151.6	44.7	1.944953	1.370708
1545	Aerobics	1	13	155.1	61.3	2.54822	1.639212
1605	Aerobics	0	14	174.6	56.2	1.84352	1.639588
1274	Aerobics	0	14	175.1	111.2	3.626874	2.369267
1274	Aerobics	0	14	175.1	111.2	3.626874	2.369267
1268	Aerobics	0	14	169.4	57.5	2.003738	1.640102
1302	Aerobics	0	14	171.2	53.4	1.821939	1.582752
1269	Aerobics	0	15	172.3	76.3	2.570123	1.922496
201019	Aerobics	0	5	110	19.3	1.595041	0.768354
201031	Aerobics	0	5	109	17	1.430856	0.715075
201054	Aerobics	1	5	108.3	19.4	1.654035	0.76575
201039	Aerobics	0	6	125.2	28.1	1.792659	0.989885
201002	Aerobics	1	6	121.8	20.3	1.368363	0.822058
201023	Aerobics	1	6	118.5	21.2	1.50973	0.832349
201025	Aerobics	1	6	123.85	28	1.825433	0.983753
201030	Aerobics	1	6	122.7667	27.8	1.844522	0.97656
201029	Aerobics	0	7	125.9667	26.7	1.682674	0.965382
201037	Aerobics	0	7	112.5	16.7	1.319506	0.71719
201038	Aerobics	0	7	128.8	35.1	2.115804	1.128281
201043	Aerobics	0	7	125.65	26.6	1.684832	0.962475
201049	Aerobics	0	7	121.6	20.6	1.393157	0.828029
201005	Aerobics	1	7	119	22.3	1.574748	0.856733
201012	Aerobics	1	7	122.7	22.8	1.514418	0.877598
201017	Aerobics	1	7	129	25.5	1.53236	0.950726
201018	Aerobics	1	7	130.15	27.7	1.635277	0.997497
201020	Aerobics	1	7	122	21.2	1.424348	0.842009
201021	Aerobics	1	7	130.2	24.1	1.421658	0.92568

201026	Aerobics	1	7	118.05	26.2	1.88005	0.931343
201046	Aerobics	1	7	127.6	23.2	1.424907	0.899703
201001	Aerobics	0	8	136.8	31.2	1.66718	1.084629
201035	Aerobics	0	8	135.5	34	1.851827	1.131645
201006	Aerobics	1	8	134.5	28.6	1.580962	1.02811
201024	Aerobics	1	8	133.45	30.9	1.735087	1.068455
201041	Aerobics	1	8	126.95	23.4	1.451946	0.902037
201044	Aerobics	1	8	128.8	26.3	1.585346	0.966058
201051	Aerobics	1	8	145	36	1.712247	1.198743
201052	Aerobics	1	8	129.85	27.7	1.642842	0.996585
3079	Basketball	0	5	114.5	21.033	1.604317	0.817612
4054	Basketball	1	5	120.667	19.967	1.37131	0.811765
4090	Basketball	1	5	110.567	15.5	1.267887	0.68428
3042	Basketball	0	6	120.9	23.3	1.594055	0.88271
3078	Basketball	0	6	114.35	19.5	1.491291	0.784595
4060	Basketball	0	6	121.633	22.6	1.527586	0.870431
4066	Basketball	0	6	119.533	21.4	1.497746	0.839446
3103	Basketball	0	6	129.5	27.225	1.623411	0.986302
4077	Basketball	1	6	110.867	18.7	1.521378	0.757768
4087	Basketball	1	6	119.033	31.617	2.231444	1.03379
4073	Basketball	1	6	121.4	23.7	1.608092	0.892286
3133	Basketball	1	6	124.55	23.8	1.534227	0.903437
4075	Basketball	1	6	130.567	28.617	1.678639	1.01641
4047	Basketball	1	6	127.633	24.3	1.491696	0.922493
3122	Basketball	1	6	131.4	24.6	1.424769	0.939369
3135	Basketball	0	7	120.45	23.475	1.61805	0.88496
3108	Basketball	0	7	123	27.1	1.791262	0.963983
3005	Basketball	0	7	127	25.067	1.554157	0.936193
3049	Basketball	0	7	122.35	23.2	1.549816	0.884842
3106	Basketball	0	7	130.95	41.075	2.395337	1.235899
4029	Basketball	0	7	128.3	27.05	1.643289	0.979267
4014	Basketball	0	7	118.7	20.2	1.433672	0.811541
4063	Basketball	0	7	141.033	55.683	2.799504	1.499066
3028	Basketball	0	7	127.3	24.55	1.514937	0.926625
4053	Basketball	0	7	128.567	28.567	1.728246	1.00926
3043	Basketball	0	7	129	27.475	1.651043	0.989643
3064	Basketball	0	7	129.2	32.1	1.923003	1.076671
4078	Basketball	1	7	126.8	25.967	1.61504	0.953527
3080	Basketball	1	7	116.45	21.35	1.574414	0.829752
4006	Basketball	1	7	129.333	25.267	1.51055	0.947011
4071	Basketball	1	7	122	21.617	1.452365	0.850876
3119	Basketball	0	8	131.8	28.483	1.639664	1.017633
3003	Basketball	0	8	130.5	32.275	1.895157	1.084118
3054	Basketball	0	8	135.9	66.433	3.597037	1.624301
4032	Basketball	0	8	129.767	26.8	1.591499	0.97879
4084	Basketball	0	8	136.667	27.417	1.467887	1.011404
4021	Basketball	0	8	132.667	26.583	1.510352	0.983095

3101	Basketball	0	8	137	36.075	1.922052	1.173389
4097	Basketball	0	8	138.033	33.75	1.771364	1.135469
3104	Basketball	0	8	132.167	29.467	1.686902	1.037534
4061	Basketball	0	8	135.933	31.55	1.707456	1.088409
3033	Basketball	1	8	125.3	24.7	1.573239	0.923848
4088	Basketball	1	8	128.033	25.117	1.53223	0.940211
3001	Basketball	1	8	125.5	24.05	1.52696	0.911269
4030	Basketball	1	8	131.733	31.583	1.81997	1.075557
4049	Basketball	1	8	123.367	28.55	1.875893	0.992559
4002	Basketball	1	8	138.267	33.2	1.736605	1.126235
3066	Basketball	1	8	132.05	28.975	1.661677	1.02782
3008	Basketball	1	8	134.6	31.8	1.755242	1.088777
4003	Basketball	1	8	128.367	21.517	1.305795	0.866047
4035	Basketball	1	8	157.033	63.383	2.570342	1.677151
3039	Basketball	1	8	126.55	22.65	1.414308	0.885264
4033	Basketball	1	8	132.467	32.033	1.825502	1.086161
4034	Basketball	1	8	131.233	31.333	1.819348	1.069356
3111	Basketball	0	9	136.5	34.875	1.871755	1.150565
4062	Basketball	0	9	138.367	39.617	2.069267	1.238872
4005	Basketball	0	9	134.233	31.9	1.770403	1.089438
4042	Basketball	0	9	132.967	31.65	1.790136	1.08077
3068	Basketball	0	9	141.1	38.525	1.935034	1.229887
3100	Basketball	0	9	128.45	26.925	1.631877	0.977283
3127	Basketball	0	9	147.5	35.7	1.640908	1.201474
3132	Basketball	0	9	135.267	32.95	1.800826	1.111955
3091	Basketball	0	9	140.45	36.875	1.869341	1.199075
3048	Basketball	1	9	134.367	32.483	1.799165	1.100536
4027	Basketball	1	9	139.867	41.717	2.132468	1.279223
3061	Basketball	1	9	138.167	34.2	1.791503	1.144026
4064	Basketball	1	9	133.233	39.933	2.249616	1.225668
4023	Basketball	1	9	134.333	30.917	1.713294	1.071569
4104	Basketball	1	9	133.133	32.633	1.841135	1.099238
4092	Basketball	1	9	153	61.65	2.633602	1.635377
3056	Basketball	1	9	134.15	30.375	1.687854	1.060851
4020	Basketball	1	9	137.2	30.8	1.636223	1.078376
3006	Basketball	1	9	134.75	29.375	1.617783	1.043768
3072	Basketball	1	9	132.15	28.2	1.614785	1.013247
3118	Basketball	1	9	146.95	42.425	1.964639	1.31638
4017	Basketball	1	9	140.5	46.083	2.334469	1.351984
4102	Basketball	1	9	136.8	33.217	1.774959	1.121792
3027	Basketball	0	10	137.75	32.9	1.733855	1.119088
4013	Basketball	0	10	149.8	45.617	2.03284	1.379214
3125	Basketball	0	10	153.5	41.817	1.774746	1.328976
4069	Basketball	0	10	133.95	26.45	1.474145	0.984194
3029	Basketball	0	10	151.85	36.575	1.586188	1.231328
4028	Basketball	0	10	148.667	50.267	2.274332	1.448759
4055	Basketball	0	10	147.633	41.6	1.908651	1.304947

4041	Basketball	0	10	135.467	38.3	2.087045	1.206378
3126	Basketball	0	10	144	31.3	1.509452	1.108815
3022	Basketball	0	10	144.8	32.875	1.567937	1.140983
4025	Basketball	0	10	147.633	34.767	1.595146	1.184907
3070	Basketball	0	10	140	35.7	1.821429	1.176875
4052	Basketball	0	10	140.867	42.75	2.154357	1.299828
4040	Basketball	1	10	150.667	59.333	2.613726	1.592302
3041	Basketball	1	10	142.45	36.583	1.802828	1.20067
4100	Basketball	1	10	138.3	34.45	1.801129	1.148954
4070	Basketball	1	10	138.5	38.483	2.006178	1.220137
3123	Basketball	1	10	145.45	33.125	1.565772	1.147677
4048	Basketball	1	10	132.8	24.033	1.362736	0.931568
3114	Basketball	1	10	140.55	27.5	1.392102	1.024362
4105	Basketball	1	10	151.533	52.033	2.266024	1.487127
4068	Basketball	1	10	150.867	56.45	2.480136	1.551029
4081	Basketball	1	10	142.733	35.6	1.747436	1.184141
3086	Basketball	0	11	144	38.4	1.851852	1.23768
3102	Basketball	0	11	149.6	38.05	1.700167	1.250368
4050	Basketball	0	11	142.533	52.433	2.580916	1.457457
4026	Basketball	0	11	158.633	61.25	2.433991	1.653187
3128	Basketball	0	11	145.067	35.917	1.706722	1.197475
3002	Basketball	0	11	141.55	31.225	1.558413	1.099878
3034	Basketball	0	11	142.8	42.525	2.085393	1.303165
3052	Basketball	0	11	154.4	41.9	1.757598	1.333481
4019	Basketball	0	11	144.633	34.1	1.63012	1.163123
3038	Basketball	0	11	143.3	34.6	1.684937	1.167969
4037	Basketball	0	11	138.033	33.6	1.763492	1.132752
4010	Basketball	0	11	144.567	48.3	2.311047	1.402357
3017	Basketball	1	11	133.9	29.533	1.6472	1.044161
4096	Basketball	1	11	155.8	77.033	3.173525	1.856816
3060	Basketball	1	11	157.75	63.875	2.566801	1.687183
3000	Basketball	1	11	151	52.275	2.292663	1.488762
4089	Basketball	1	11	157.5	54.6	2.201058	1.549689
4101	Basketball	1	11	137.633	28.2	1.488689	1.029708
3120	Basketball	1	11	157.667	43.3	1.741832	1.368573
4016	Basketball	1	11	148.7	42.067	1.902478	1.316558
4083	Basketball	1	11	164.633	61.1	2.254278	1.675485
3009	Basketball	1	11	164.5	58.367	2.156928	1.63423
3113	Basketball	0	12	157.05	62.475	2.532972	1.664258
3134	Basketball	0	12	164.25	76.775	2.845831	1.892683
4008	Basketball	0	12	135.9	27.967	1.514283	1.019987
4038	Basketball	0	12	154.6	37.633	1.574526	1.259285
4039	Basketball	0	12	155.967	40.733	1.674484	1.318644
4085	Basketball	0	12	150.533	56.633	2.499229	1.552367
4046	Basketball	0	12	153.633	36.45	1.544289	1.234763
4108	Basketball	0	12	135.633	28.783	1.564607	1.035079
3095	Basketball	0	12	160.9	78.967	3.050237	1.905923

4031	Basketball	0	12	154.733	52.733	2.202503	1.510313
3058	Basketball	0	12	175.4	65.95	2.143659	1.790141
3088	Basketball	0	12	156.5	49.283	2.012188	1.462912
3004	Basketball	0	12	159.95	48.775	1.906465	1.467412
4076	Basketball	1	12	156.433	49.467	2.021431	1.465598
3124	Basketball	1	12	167.65	50.217	1.786667	1.518628
3065	Basketball	1	12	152.95	33.95	1.451245	1.186373
4109	Basketball	1	12	157.067	51.433	2.084836	1.499044
3085	Basketball	1	12	158	49.3	1.974844	1.468727
4098	Basketball	1	12	158.133	46.733	1.868868	1.427567
4099	Basketball	1	12	158.9	48.85	1.934714	1.464796
4103	Basketball	1	12	151.467	40.75	1.776199	1.303722
4106	Basketball	1	12	157.067	46.983	1.904456	1.427835
4107	Basketball	1	12	152.767	50.117	2.147464	1.462115
4110	Basketball	1	12	152.467	41.917	1.803178	1.327128
3094	Basketball	1	12	168.7	51.575	1.812212	1.544396
4079	Basketball	1	12	152.133	59.8	2.583773	1.605179
4057	Basketball	0	13	158.267	47.45	1.894329	1.439788
3131	Basketball	0	13	159.1	45.875	1.812323	1.416831
4056	Basketball	0	13	159.533	51.317	2.016323	1.506499
4074	Basketball	0	13	171.533	62.25	2.115651	1.72014
3087	Basketball	0	13	167.5	48.275	1.72065	1.486229
3014	Basketball	0	13	169.8	53.75	1.864245	1.583161
3020	Basketball	0	13	147.5	33.7	1.54898	1.164793
3024	Basketball	0	13	173.15	56.45	1.882864	1.638083
4091	Basketball	0	13	168.5	66.033	2.32574	1.76308
3035	Basketball	0	13	165.6	47.9	1.746686	1.473331
3115	Basketball	0	13	152.25	38.525	1.661989	1.26753
3044	Basketball	0	13	160.233	43.9	1.70986	1.387591
4086	Basketball	0	13	167.767	83.95	2.982687	2.002591
3110	Basketball	0	13	169.75	64.033	2.222206	1.739245
4012	Basketball	0	13	156.467	62.25	2.542693	1.658585
3010	Basketball	1	13	173.75	67.35	2.23094	1.803709
4022	Basketball	1	13	158.967	49.317	1.951564	1.472557
3069	Basketball	1	13	163.25	49.675	1.863938	1.493958
3015	Basketball	1	13	164.5	45	1.662956	1.420909
4093	Basketball	1	13	155.333	53.483	2.216605	1.524164
4067	Basketball	1	13	159.167	59.85	2.362425	1.634933
4094	Basketball	1	13	167.167	79.983	2.862178	1.948361
4043	Basketball	1	13	162.8	54.133	2.042459	1.562917
4080	Basketball	1	13	157.433	52.817	2.130994	1.522007
3021	Basketball	0	14	159.75	48.225	1.88969	1.457767
4045	Basketball	0	14	164.7	56.733	2.091455	1.61024
4007	Basketball	0	14	163.933	51.367	1.911399	1.523632
3117	Basketball	0	14	162.05	53.35	2.031591	1.547883
3055	Basketball	0	14	184.6	71.3	2.092311	1.905049
3096	Basketball	0	14	170.567	106.5	3.660662	2.290931

3081	Basketball	0	14	179.8	68.05	2.104984	1.838543
3047	Basketball	0	14	174.3	77.85	2.5625	1.952313
3097	Basketball	0	14	182.3	97.35	2.929292	2.241213
3053	Basketball	1	14	170.6	50.125	1.722251	1.527658
3098	Basketball	1	14	159.75	44.825	1.756461	1.401561
3025	Basketball	1	14	156.35	51.275	2.097538	1.493853
4015	Basketball	1	14	154.667	45.9	1.918745	1.401459
3112	Basketball	1	14	160.3	69.25	2.694963	1.773347
4051	Basketball	1	14	158.967	58.9	2.33078	1.620117
4082	Basketball	1	14	168.133	47.517	1.680905	1.475837
4095	Basketball	1	14	167.4	86.733	3.095091	2.036257
4024	Basketball	1	14	175.533	54.767	1.777466	1.62039
3011	Basketball	1	14	170.25	60.675	2.09332	1.691554
4036	Basketball	1	14	157	72.7	2.94941	1.805378
4065	Basketball	0	15	170	77.817	2.69263	1.932636
3023	Basketball	0	15	174.15	71.4	2.354243	1.86295
4009	Basketball	0	15	190.4	117.15	3.231532	2.518894
3075	Basketball	1	15	166.5	68.125	2.457412	1.784437
3093	Basketball	1	15	166.2	53.925	1.952217	1.572516
3016	Basketball	1	15	159.4	61.633	2.425698	1.661913
3026	Basketball	0	16	180.4	71.4	2.193942	1.889171
3032	Basketball	0	16	170.3	59.7	2.058472	1.677076
3084	Basketball	1	16	165.65	54.125	1.972491	1.573581
4011	Basketball	1	16	163.633	62.1	2.319262	1.6861
4058	Basketball	1	16	165.733	68.533	2.495064	1.786903
201019	Basketball	0	5	110	19.3	1.595041	0.768354
201031	Basketball	0	5	109	17	1.430856	0.715075
201032	Basketball	0	5	115.1	21.4	1.615337	0.826965
201054	Basketball	1	5	108.3	19.4	1.654035	0.76575
201039	Basketball	0	6	125.2	28.1	1.792659	0.989885
201042	Basketball	0	6	123.3	29.9	1.966731	1.017312
201002	Basketball	1	6	121.8	20.3	1.368363	0.822058
201023	Basketball	1	6	118.5	21.2	1.50973	0.832349
201025	Basketball	1	6	123.85	28	1.825433	0.983753
201030	Basketball	1	6	122.7667	27.8	1.844522	0.97656
201029	Basketball	0	7	125.9667	26.7	1.682674	0.965382
201037	Basketball	0	7	112.5	16.7	1.319506	0.71719
201038	Basketball	0	7	128.8	35.1	2.115804	1.128281
201043	Basketball	0	7	125.65	26.6	1.684832	0.962475
201049	Basketball	0	7	121.6	20.6	1.393157	0.828029
201005	Basketball	1	7	119	22.3	1.574748	0.856733
201012	Basketball	1	7	122.7	22.8	1.514418	0.877598
201017	Basketball	1	7	129	25.5	1.53236	0.950726
201018	Basketball	1	7	130.15	27.7	1.635277	0.997497
201020	Basketball	1	7	122	21.2	1.424348	0.842009
201021	Basketball	1	7	130.2	24.1	1.421658	0.92568
201026	Basketball	1	7	118.05	26.2	1.88005	0.931343

201046	Basketball	1	7	127.6	23.2	1.424907	0.899703
201001	Basketball	0	8	136.8	31.2	1.66718	1.084629
201035	Basketball	0	8	135.5	34	1.851827	1.131645
201040	Basketball	0	8	134	37	2.060593	1.179081
210398	Basketball	0	8	136	31.5	1.703071	1.087694
210642	Basketball	0	8	135	31.8	1.744856	1.090059
210705	Basketball	0	8	143.8	38.2	1.847335	1.23353
210705	Basketball	0	8	143.8	38.2	1.847335	1.23353
210710	Basketball	0	8	130.7	23.6	1.381532	0.916693
210721	Basketball	0	8	138.6	30.9	1.608541	1.084613
201006	Basketball	1	8	134.5	28.6	1.580962	1.02811
201013	Basketball	1	8	134.6	39.4	2.174734	1.221776
201024	Basketball	1	8	133.45	30.9	1.735087	1.068455
201041	Basketball	1	8	126.95	23.4	1.451946	0.902037
201044	Basketball	1	8	128.8	26.3	1.585346	0.966058
201051	Basketball	1	8	145	36	1.712247	1.198743
201052	Basketball	1	8	129.85	27.7	1.642842	0.996585
210780	Basketball	1	8	129.3	23	1.375722	0.900234
210654	Basketball	0	9	139.7	31	1.588433	1.08991
210672	Basketball	0	9	137.1	32.3	1.718413	1.10599
210817	Basketball	0	9	134.2	27.8	1.543618	1.011646
210102	Basketball	0	10	137	30.9	1.646332	1.079633
210248	Basketball	0	10	138.3	31.8	1.662581	1.100544
210280	Basketball	0	10	152	41.8	1.809211	1.323524
210350	Basketball	0	10	152.4	59.5	2.561811	1.601957
210431	Basketball	0	10	141.8	34.5	1.7158	1.161298
210693	Basketball	0	10	149.7	46.4	2.070496	1.391527
210826	Basketball	0	10	138.7	32	1.663401	1.105526
210365	Basketball	0	11	146	40	1.876525	1.272089
210456	Basketball	0	11	141.7	34.2	1.703281	1.155534
210849	Basketball	0	11	146.8	38	1.763321	1.240161
210449	Basketball	1	11	142.2	33.2	1.64187	1.138827
210040	Basketball	0	12	159.5	54.5	2.142275	1.555925
210122	Basketball	0	12	175	59.8	1.952653	1.696799
210130	Basketball	0	12	156.8	57.5	2.338707	1.590614
210202	Basketball	0	12	138.9	30.5	1.580867	1.077963
210217	Basketball	0	12	159.3	47.7	1.879693	1.447591
210342	Basketball	0	12	148.9	49.3	2.223604	1.434593
210764	Basketball	0	12	146.5	37.8	1.761232	1.235644
210837	Basketball	0	12	156	51	2.095661	1.488217
210326	Basketball	0	13	152.8	50.4	2.158658	1.466675
210428	Basketball	0	13	160.6	46.5	1.802859	1.432498
210772	Basketball	0	13	180.5	83.4	2.559833	2.054239
210033	Basketball	1	13	163.6	66.6	2.488328	1.750606
210057	Basketball	1	13	170.2	53.4	1.843411	1.579081
210307	Basketball	0	14	171.5	63.4	2.155564	1.737026
210338	Basketball	0	14	177	72.7	2.320534	1.893259

210585	Basketball	0	14	171.3	55.5	1.891378	1.616302
210618	Basketball	0	14	170	69.8	2.415225	1.82287
210625	Basketball	0	14	171.5	56	1.903969	1.624868
210317	Basketball	0	15	176.1	79.1	2.550691	1.977145
210502	Basketball	0	15	193.3	78	2.087521	2.036156
210570	Basketball	0	15	177.6	69.2	2.193917	1.846157
210594	Basketball	0	15	180.2	70.2	2.16186	1.871206
210605	Basketball	0	15	169.8	57.2	1.983904	1.637024
210299	Basketball	0	16	184.6	70.4	2.0659	1.892078
210488	Basketball	0	16	173.3	57.7	1.921227	1.658061
210499	Basketball	0	16	174.1	54.1	1.784842	1.604525
210527	Basketball	0	16	173.2	86.2	2.873502	2.057105
201019	Cycling 10mph	0	5	110	19.3	1.595041	0.768354
201031	Cycling 10mph	0	5	109	17	1.430856	0.715075
201032	Cycling 10mph	0	5	115.1	21.4	1.615337	0.826965
201054	Cycling 10mph	1	5	108.3	19.4	1.654035	0.76575
201039	Cycling 10mph	0	6	125.2	28.1	1.792659	0.989885
201042	Cycling 10mph	0	6	123.3	29.9	1.966731	1.017312
201002	Cycling 10mph	1	6	121.8	20.3	1.368363	0.822058
201023	Cycling 10mph	1	6	118.5	21.2	1.50973	0.832349
201025	Cycling 10mph	1	6	123.85	28	1.825433	0.983753
201030	Cycling 10mph	1	6	122.7667	27.8	1.844522	0.97656
201029	Cycling 10mph	0	7	125.9667	26.7	1.682674	0.965382
201037	Cycling 10mph	0	7	112.5	16.7	1.319506	0.71719
201038	Cycling 10mph	0	7	128.8	35.1	2.115804	1.128281
201043	Cycling 10mph	0	7	125.65	26.6	1.684832	0.962475
201049	Cycling 10mph	0	7	121.6	20.6	1.393157	0.828029
201005	Cycling 10mph	1	7	119	22.3	1.574748	0.856733
201012	Cycling 10mph	1	7	122.7	22.8	1.514418	0.877598
201017	Cycling 10mph	1	7	129	25.5	1.53236	0.950726
201018	Cycling 10mph	1	7	130.15	27.7	1.635277	0.997497
201020	Cycling 10mph	1	7	122	21.2	1.424348	0.842009
201021	Cycling 10mph	1	7	130.2	24.1	1.421658	0.92568
201026	Cycling 10mph	1	7	118.05	26.2	1.88005	0.931343
201046	Cycling 10mph	1	7	127.6	23.2	1.424907	0.899703
100601	Cycling 10mph	0	8	142.2	33.6	1.661652	1.146186
100989	Cycling 10mph	0	8	130.4	29.3	1.723108	1.028863
101033	Cycling 10mph	0	8	128.3	28.1	1.707077	0.999529
101419	Cycling 10mph	0	8	133.4	29.8	1.674575	1.047673
111777	Cycling 10mph	0	8	125.1	25.7	1.642171	0.943181
112090	Cycling 10mph	0	8	126.7	23.8	1.482599	0.909587
112125	Cycling 10mph	0	8	125	23.6	1.5104	0.900632
112198	Cycling 10mph	0	8	127.2	26.5	1.637841	0.965207
113546	Cycling 10mph	0	8	134.6	31	1.711085	1.07396
201001	Cycling 10mph	0	8	136.8	31.2	1.66718	1.084629
201035	Cycling 10mph	0	8	135.5	34	1.851827	1.131645
201040	Cycling 10mph	0	8	134	37	2.060593	1.179081

100841	Cycling 10mph	1	8	134.7	27.3	1.504622	1.003298
100912	Cycling 10mph	1	8	127.1	23.4	1.448521	0.90246
101300	Cycling 10mph	1	8	135.7	35.1	1.906108	1.151864
111703	Cycling 10mph	1	8	129.9	34.3	2.032712	1.11814
111734	Cycling 10mph	1	8	132.3	30.9	1.765382	1.064796
111901	Cycling 10mph	1	8	124.5	23.7	1.529008	0.90125
111932	Cycling 10mph	1	8	130	27.2	1.609467	0.987322
112139	Cycling 10mph	1	8	140.1	34.1	1.737313	1.148534
112484	Cycling 10mph	1	8	139	37.7	1.951245	1.208447
201006	Cycling 10mph	1	8	134.5	28.6	1.580962	1.02811
201013	Cycling 10mph	1	8	134.6	39.4	2.174734	1.221776
201024	Cycling 10mph	1	8	133.45	30.9	1.735087	1.068455
201041	Cycling 10mph	1	8	126.95	23.4	1.451946	0.902037
201044	Cycling 10mph	1	8	128.8	26.3	1.585346	0.966058
201051	Cycling 10mph	1	8	145	36	1.712247	1.198743
201052	Cycling 10mph	1	8	129.85	27.7	1.642842	0.996585
100339	Cycling 10mph	0	9	133	28.5	1.611171	1.021623
100505	Cycling 10mph	0	9	143.3	26.9	1.309966	1.020087
100816	Cycling 10mph	0	9	129.3	26.1	1.561146	0.963579
100923	Cycling 10mph	0	9	132.4	28.3	1.614397	1.015939
101230	Cycling 10mph	0	9	131.4	27.1	1.569562	0.989561
112335	Cycling 10mph	0	9	148.8	48.9	2.208528	1.427941
112772	Cycling 10mph	0	9	136.5	34.4	1.846261	1.14211
112895	Cycling 10mph	0	9	143.4	34.4	1.672862	1.164656
113182	Cycling 10mph	0	9	124.7	26.2	1.684878	0.951797
113347	Cycling 10mph	0	9	139.2	28.8	1.486326	1.046117
100234	Cycling 10mph	1	9	133.3	29.6	1.665833	1.043575
100556	Cycling 10mph	1	9	134.6	27.9	1.539976	1.014798
100589	Cycling 10mph	1	9	127.2	27.6	1.705827	0.986552
100953	Cycling 10mph	1	9	155.5	38.6	1.596344	1.279525
101071	Cycling 10mph	1	9	144.2	37	1.77939	1.213872
101104	Cycling 10mph	1	9	133.4	27.6	1.550949	1.00534
101373	Cycling 10mph	1	9	134.2	32	1.776826	1.091167
112564	Cycling 10mph	1	9	131.5	28.7	1.659703	1.020872
112780	Cycling 10mph	1	9	142.2	35.4	1.750669	1.17881
113147	Cycling 10mph	1	9	130	35	2.071006	1.1307
113199	Cycling 10mph	1	9	137.9	29.2	1.535516	1.049993
113212	Cycling 10mph	1	9	129.6	26.3	1.565834	0.968432
113274	Cycling 10mph	1	9	137	31.5	1.678299	1.090857
100356	Cycling 10mph	0	10	139.2	33.9	1.749529	1.141985
100804	Cycling 10mph	0	10	129.5	26.4	1.574216	0.970114
100861	Cycling 10mph	0	10	142.2	42.2	2.086956	1.295635
101082	Cycling 10mph	0	10	146.5	30.5	1.4211	1.100968
101150	Cycling 10mph	0	10	127.1	33.1	2.048976	1.087495
101265	Cycling 10mph	0	10	133	28.7	1.622477	1.025472
101428	Cycling 10mph	0	10	137.2	29.4	1.561849	1.051731
101459	Cycling 10mph	0	10	140.9	35.5	1.78816	1.176309

111564	Cycling 10mph	0	10	140.6	31	1.568162	1.092688
111621	Cycling 10mph	0	10	137.9	32	1.682757	1.102994
111683	Cycling 10mph	0	10	142.6	35.2	1.731026	1.176532
113530	Cycling 10mph	0	10	146.5	45.2	2.106023	1.360354
100186	Cycling 10mph	1	10	142	32.6	1.616743	1.127083
100258	Cycling 10mph	1	10	151.6	59.3	2.580217	1.595726
100445	Cycling 10mph	1	10	146.2	42.4	1.983678	1.313297
100851	Cycling 10mph	1	10	149.1	43.2	1.943249	1.336934
100946	Cycling 10mph	1	10	140.5	37	1.874343	1.201429
101331	Cycling 10mph	1	10	150.9	41.3	1.813726	1.311205
111839	Cycling 10mph	1	10	155.5	46	1.902379	1.406091
112576	Cycling 10mph	1	10	148.5	38.9	1.763992	1.261617
100438	Cycling 10mph	0	11	144.4	42.4	2.033441	1.306863
100594	Cycling 10mph	0	11	150.6	57.1	2.517597	1.559513
100791	Cycling 10mph	0	11	142.2	36.2	1.790232	1.193062
100890	Cycling 10mph	0	11	146.9	37.4	1.733118	1.229923
100990	Cycling 10mph	0	11	148.6	40.2	1.820491	1.284462
101112	Cycling 10mph	0	11	141.4	32.2	1.610486	1.117747
101220	Cycling 10mph	0	11	142.8	30.9	1.515312	1.097525
101396	Cycling 10mph	0	11	160.4	52.8	2.052226	1.533048
101546	Cycling 10mph	0	11	146.4	41.6	1.940936	1.300616
112001	Cycling 10mph	0	11	158.8	44.4	1.760686	1.391105
112203	Cycling 10mph	0	11	149.9	39	1.735647	1.268068
112228	Cycling 10mph	0	11	138.5	34.1	1.777685	1.143316
112524	Cycling 10mph	0	11	151.9	43.3	1.876603	1.348506
100265	Cycling 10mph	1	11	146.9	34.4	1.594097	1.175842
100319	Cycling 10mph	1	11	147	39.3	1.818687	1.263482
100417	Cycling 10mph	1	11	155.3	49	2.03167	1.453945
100649	Cycling 10mph	1	11	149	41.5	1.869285	1.30803
101012	Cycling 10mph	1	11	147.7	58	2.658686	1.56061
101040	Cycling 10mph	1	11	146.9	35.4	1.640437	1.194103
101060	Cycling 10mph	1	11	164.8	38.7	1.42494	1.311152
111270	Cycling 10mph	1	11	140.9	38.1	1.919124	1.221884
111636	Cycling 10mph	1	11	157	45	1.825632	1.394866
111785	Cycling 10mph	1	11	153.5	83.1	3.526828	1.922716
111948	Cycling 10mph	1	11	139	45	2.329072	1.329135
112852	Cycling 10mph	1	11	149.7	43.2	1.927703	1.339064
100518	Cycling 10mph	0	12	140.3	35.6	1.808567	1.176099
100529	Cycling 10mph	0	12	155.8	46.6	1.919778	1.417007
100692	Cycling 10mph	0	12	161.1	47	1.810951	1.442538
101173	Cycling 10mph	0	12	172	53.1	1.794889	1.580883
111891	Cycling 10mph	0	12	161	47.1	1.81706	1.443832
112075	Cycling 10mph	0	12	152.7	47.1	2.01996	1.413855
112425	Cycling 10mph	0	12	153.8	34.3	1.450045	1.195559
112702	Cycling 10mph	0	12	149	47.8	2.153056	1.411327
112748	Cycling 10mph	0	12	170.3	46	1.586093	1.457689
112762	Cycling 10mph	0	12	164.5	54.5	2.014024	1.57508

112845	Cycling 10mph	0	12	165.2	60.8	2.227837	1.673335
113051	Cycling 10mph	0	12	161	42.6	1.643455	1.367926
100126	Cycling 10mph	1	12	153.8	39	1.648739	1.281045
100179	Cycling 10mph	1	12	169.1	61.3	2.143746	1.696339
100459	Cycling 10mph	1	12	162.5	54.4	2.060118	1.565913
100672	Cycling 10mph	1	12	162.2	53.9	2.048741	1.557015
100882	Cycling 10mph	1	12	157.4	45.6	1.840583	1.406256
101209	Cycling 10mph	1	12	156.7	54.2	2.2073	1.54046
101256	Cycling 10mph	1	12	154.1	45.9	1.932891	1.39942
112085	Cycling 10mph	1	12	142.2	39.2	1.938594	1.245257
112399	Cycling 10mph	1	12	155.8	54.7	2.253473	1.544556
112411	Cycling 10mph	1	12	166	60.7	2.202787	1.675059
112594	Cycling 10mph	1	12	152.6	46.6	2.001137	1.405398
112656	Cycling 10mph	1	12	152	31.2	1.350416	1.130888
113247	Cycling 10mph	1	12	138.2	33.5	1.753996	1.13148
100071	Cycling 10mph	0	13	160	43.1	1.683594	1.373142
100322	Cycling 10mph	0	13	166.7	55.3	1.990004	1.595854
100404	Cycling 10mph	0	13	172.2	47.2	1.591753	1.484533
100756	Cycling 10mph	0	13	170.1	58.6	2.025298	1.659613
100901	Cycling 10mph	0	13	173.1	41.8	1.395025	1.393509
100932	Cycling 10mph	0	13	151.8	42.4	1.840019	1.333011
101313	Cycling 10mph	0	13	151.3	35.9	1.568255	1.217302
101461	Cycling 10mph	0	13	153.9	40.2	1.697262	1.30243
101550	Cycling 10mph	0	13	170.9	52.1	1.783831	1.560826
111654	Cycling 10mph	0	13	171	68.5	2.342601	1.808733
111710	Cycling 10mph	0	13	155.9	42.7	1.756854	1.352286
111820	Cycling 10mph	0	13	167.5	59.1	2.106483	1.657065
112263	Cycling 10mph	0	13	165.5	51.3	1.872929	1.528315
100652	Cycling 10mph	1	13	167.4	70.1	2.501538	1.815951
100669	Cycling 10mph	1	13	164.2	69.4	2.574027	1.79241
100703	Cycling 10mph	1	13	173.4	63.1	2.098607	1.740184
101219	Cycling 10mph	1	13	152.4	44.5	1.915976	1.370262
112102	Cycling 10mph	1	13	167.8	61.8	2.194849	1.698562
112162	Cycling 10mph	1	13	153.6	56.4	2.390544	1.561362
112173	Cycling 10mph	1	13	159.4	52	2.04657	1.516747
112189	Cycling 10mph	1	13	166.6	55.4	1.995996	1.597025
112237	Cycling 10mph	1	13	152.5	41.3	1.775867	1.316699
112534	Cycling 10mph	1	13	163.3	79.8	2.992475	1.927992
112680	Cycling 10mph	1	13	148.3	39.1	1.777847	1.264426
112871	Cycling 10mph	1	13	163	48.4	1.821672	1.472317
100102	Cycling 10mph	0	14	179.4	96.7	3.004565	2.219003
100567	Cycling 10mph	0	14	144.5	35.5	1.700171	1.188132
101124	Cycling 10mph	0	14	166	48.2	1.749165	1.479701
101193	Cycling 10mph	0	14	178.9	60.3	1.884068	1.719371
101366	Cycling 10mph	0	14	156.5	43	1.755657	1.359456
101444	Cycling 10mph	0	14	165.3	53.7	1.965299	1.565611
111870	Cycling 10mph	0	14	173	68.6	2.292091	1.818515

111883	Cycling 10mph	0	14	180	61.7	1.904321	1.744961
111991	Cycling 10mph	0	14	158.8	46.9	1.859824	1.432696
112015	Cycling 10mph	0	14	171.9	57	1.928959	1.641924
112023	Cycling 10mph	0	14	163.4	50.5	1.891417	1.507799
112359	Cycling 10mph	0	14	173.7	83.7	2.774124	2.027116
100278	Cycling 10mph	1	14	168.6	53.4	1.878565	1.57318
100284	Cycling 10mph	1	14	168.1	54.5	1.928684	1.588654
100683	Cycling 10mph	1	14	152.5	46.2	1.986563	1.398534
111679	Cycling 10mph	1	14	161	47.7	1.840207	1.453695
111985	Cycling 10mph	1	14	154.3	40.5	1.701074	1.308995
112212	Cycling 10mph	1	14	159.1	49.2	1.94368	1.471164
112304	Cycling 10mph	1	14	175.8	108.9	3.523629	2.346494
112457	Cycling 10mph	1	14	166.9	59.1	2.121655	1.654709
112867	Cycling 10mph	1	14	151.4	50.9	2.220578	1.469112
112884	Cycling 10mph	1	14	162.6	50.6	1.913857	1.50647
112911	Cycling 10mph	1	14	165.5	69.4	2.533748	1.798022
113163	Cycling 10mph	1	14	156.5	56.9	2.323184	1.580466
100084	Cycling 10mph	0	15	179.5	63.5	1.97081	1.770203
100208	Cycling 10mph	0	15	175.1	57.9	1.888453	1.667967
101474	Cycling 10mph	0	15	172.2	69.1	2.3303	1.82228
111850	Cycling 10mph	0	15	157.5	50.3	2.027715	1.48281
112327	Cycling 10mph	0	15	165.8	72.6	2.640995	1.843467
112379	Cycling 10mph	0	15	184.7	70.8	2.075389	1.89826
112558	Cycling 10mph	0	15	161	45	1.736044	1.408847
112666	Cycling 10mph	0	15	167.4	54.8	1.955553	1.590718
112674	Cycling 10mph	0	15	177.4	87.8	2.789891	2.097379
112722	Cycling 10mph	0	15	179.5	64.1	1.989432	1.779179
112739	Cycling 10mph	0	15	174.1	57.2	1.887116	1.653334
112820	Cycling 10mph	0	15	163	55.3	2.081373	1.581718
112838	Cycling 10mph	0	15	179	67.3	2.100434	1.82439
112944	Cycling 10mph	0	15	170.8	62.5	2.142418	1.720928
100140	Cycling 10mph	1	15	166.1	80	2.899684	1.943644
100719	Cycling 10mph	1	15	172.6	71.7	2.406787	1.860551
101401	Cycling 10mph	1	15	159	67.3	2.662078	1.740687
112048	Cycling 10mph	1	15	162.6	65	2.458512	1.723667
112052	Cycling 10mph	1	15	160.4	92.8	3.606943	2.076207
112401	Cycling 10mph	1	15	158.7	52.2	2.072606	1.517233
112517	Cycling 10mph	1	15	161.1	44.1	1.699212	1.393966
112630	Cycling 10mph	1	15	163.6	88.9	3.321507	2.044765
112643	Cycling 10mph	1	15	168.8	55.2	1.937288	1.602233
112714	Cycling 10mph	1	15	174.4	60.5	1.989127	1.705129
113040	Cycling 10mph	1	15	158.8	73.7	2.922581	1.826928
113267	Cycling 10mph	1	15	161.2	53.5	2.058845	1.546993
100221	Cycling 10mph	0	16	177	69.5	2.218392	1.847976
100482	Cycling 10mph	0	16	179.2	65.5	2.039695	1.79878
100961	Cycling 10mph	0	16	174.5	70.8	2.325104	1.855991
101133	Cycling 10mph	0	16	170	53.1	1.83737	1.57357

101507	Cycling 10mph	0	16	173.9	76.1	2.516434	1.926831
112251	Cycling 10mph	0	16	173.4	57.2	1.902383	1.650695
112387	Cycling 10mph	0	16	165.7	57.2	2.083296	1.62124
112463	Cycling 10mph	0	16	167.3	62.1	2.218706	1.700978
112618	Cycling 10mph	0	16	173.6	65.6	2.176729	1.777742
112621	Cycling 10mph	0	16	179.7	80.1	2.480484	2.006581
112753	Cycling 10mph	0	16	175.3	75.1	2.443859	1.919264
112790	Cycling 10mph	0	16	176.6	76.6	2.456107	1.945475
101181	Cycling 10mph	1	16	161.8	50.9	1.944289	1.508316
111928	Cycling 10mph	1	16	159	56	2.215102	1.576847
112318	Cycling 10mph	1	16	164.1	48.4	1.797332	1.476247
112442	Cycling 10mph	1	16	167	59	2.115529	1.653596
112582	Cycling 10mph	1	16	166.2	76.4	2.765867	1.896558
113000	Cycling 10mph	1	16	160.2	99.5	3.87702	2.154457
113067	Cycling 10mph	1	16	159.8	72	2.819544	1.808638
113122	Cycling 10mph	1	16	170.8	78.1	2.677166	1.94002
113135	Cycling 10mph	1	16	165.6	61.1	2.228027	1.679379
113206	Cycling 10mph	1	16	170.9	67.7	2.317953	1.796925
113289	Cycling 10mph	1	16	172.7	63.7	2.135771	1.746261
100724	Cycling 10mph	0	17	178.3	72.6	2.283673	1.897354
111802	Cycling 10mph	0	17	181.1	71.5	2.180063	1.893498
112346	Cycling 10mph	0	17	173.3	85.6	2.850209	2.049861
112476	Cycling 10mph	0	17	172.6	69.5	2.332938	1.829628
112691	Cycling 10mph	0	17	171.8	64.1	2.171759	1.748525
113017	Cycling 10mph	0	17	170.5	72.7	2.500838	1.865387
113023	Cycling 10mph	0	17	172.8	64.3	2.153394	1.755491
113081	Cycling 10mph	0	17	173	82.2	2.7465	2.004287
113095	Cycling 10mph	0	17	183.4	94.3	2.80358	2.208433
113171	Cycling 10mph	0	17	169.2	61.6	2.151691	1.701197
100385	Cycling 10mph	1	17	164.7	59.9	2.208206	1.657974
112933	Cycling 10mph	1	17	162.5	63.7	2.412308	1.704625
112972	Cycling 10mph	1	17	168.1	48.4	1.712813	1.490408
112987	Cycling 10mph	1	17	163.6	51.4	1.920421	1.52293
112993	Cycling 10mph	1	17	171.5	69.2	2.352761	1.820756
113079	Cycling 10mph	1	17	162.9	72.8	2.7434	1.833328
113103	Cycling 10mph	1	17	155.7	49.1	2.02537	1.457025
113227	Cycling 10mph	1	17	170.9	62.3	2.133064	1.718363
113238	Cycling 10mph	1	17	168.4	67	2.362602	1.7765
113406	Cycling 10mph	1	17	159.3	55.6	2.191004	1.571954
113455	Cycling 10mph	1	17	171.5	71.6	2.43436	1.854449
113463	Cycling 10mph	1	17	159.5	62.2	2.444945	1.670533
113494	Cycling 10mph	1	17	161	56.4	2.175842	1.590757
101059	Cycling 10mph	0	18	181.8	87	2.632276	2.107447
101534	Cycling 10mph	0	18	167.7	53.1	1.888115	1.565096
111642	Cycling 10mph	0	18	184	86.4	2.551985	2.109654
111721	Cycling 10mph	0	18	176.6	61.4	1.968734	1.727286
112033	Cycling 10mph	0	18	178.1	71.6	2.257279	1.882416

112299	Cycling 10mph	0	18	164.1	61	2.265232	1.671859
112964	Cycling 10mph	0	18	178.7	71.6	2.242146	1.884928
113157	Cycling 10mph	0	18	170.4	62.5	2.152488	1.719329
113297	Cycling 10mph	0	18	169.5	54.9	1.910878	1.600166
113305	Cycling 10mph	0	18	183.5	72	2.138259	1.910553
113338	Cycling 10mph	0	18	182.5	67.8	2.035654	1.845781
113353	Cycling 10mph	0	18	163.5	57.7	2.158442	1.620239
113375	Cycling 10mph	0	18	168.7	88.4	3.106148	2.06353
113380	Cycling 10mph	0	18	179.8	69.4	2.146743	1.85807
100117	Cycling 10mph	1	18	171.8	70.1	2.375044	1.834724
100215	Cycling 10mph	1	18	165.6	71.4	2.60362	1.826143
100549	Cycling 10mph	1	18	164.9	52.6	1.934392	1.546795
111745	Cycling 10mph	1	18	164.6	62	2.2884	1.688579
112498	Cycling 10mph	1	18	159.5	62.1	2.441014	1.669088
112503	Cycling 10mph	1	18	158.2	58.6	2.341449	1.612579
112927	Cycling 10mph	1	18	171.5	64.7	2.199764	1.756091
113251	Cycling 10mph	1	18	163	46.8	1.761451	1.445938
113312	Cycling 10mph	1	18	158.6	55.8	2.218339	1.572245
113397	Cycling 10mph	1	18	168.2	50.9	1.799143	1.531689
113412	Cycling 10mph	1	18	161.5	55.4	2.12405	1.577464
113435	Cycling 10mph	1	18	168.2	65.2	2.3046	1.749847
113446	Cycling 10mph	1	18	165.4	57.9	2.116448	1.630708
113477	Cycling 10mph	1	18	154.6	51.6	2.158891	1.492265
113482	Cycling 10mph	1	18	161.3	66.5	2.555953	1.739402
113509	Cycling 10mph	1	18	159.2	63.8	2.517298	1.692244
2562	Computer Games	0	5	104.7	15.5	1.413964	0.669649
2277	Computer Games	0	5	115.5	20	1.499222	0.798513
2278	Computer Games	0	5	113.5	17.3	1.342933	0.733503
2278	Computer Games	0	5	113.5	17.3	1.342933	0.733503
2319	Computer Games	0	5	121.5	28.1	1.903504	0.978184
2319	Computer Games	0	5	121.5	28.1	1.903504	0.978184
2561	Computer Games	0	5	121.7	27.9	1.883749	0.975069
2563	Computer Games	0	5	120.2	21	1.453484	0.832806
2510	Computer Games	1	5	105.2	18.7	1.689702	0.742171
2282	Computer Games	1	5	107.1	15.8	1.377457	0.682695
2283	Computer Games	1	5	106.8	17.4	1.525481	0.718246
2310	Computer Games	0	6	114.7	33.8	2.569152	1.055948
2279	Computer Games	0	6	127.6	25.6	1.572312	0.948617
2519	Computer Games	1	6	125.8	48.5	3.064647	1.330104
2528	Computer Games	1	6	122	30.8	2.069336	1.029333
2231	Computer Games	0	7	121.3	21.6	1.468021	0.848578
2558	Computer Games	0	7	122.9	20.7	1.370461	0.833696
2222	Computer Games	0	7	120.6	20.3	1.39573	0.818838
2222	Computer Games	0	7	120.6	20.3	1.39573	0.818838
2224	Computer Games	0	7	127.8	25.1	1.536781	0.93919
2224	Computer Games	0	7	127.8	25.1	1.536781	0.93919
2322	Computer Games	0	7	135.9	53.3	2.885947	1.442854

2560	Computer Games	0	7	117.9	22	1.582687	0.84739
2507	Computer Games	0	7	117.25	21.7	1.578462	0.839314
2508	Computer Games	0	7	122.8	23	1.525215	0.882015
2275	Computer Games	1	7	118	33.8	2.427463	1.067888
2527	Computer Games	1	7	119.9	29.4	2.045074	0.997014
2554	Computer Games	1	7	131.6	41.4	2.390499	1.243587
2509	Computer Games	1	7	120.4	28.7	1.979835	0.985802
2261	Computer Games	1	7	120.8	26.2	1.795426	0.939884
2261	Computer Games	1	7	120.8	26.2	1.795426	0.939884
2306	Computer Games	0	8	130.3	35	2.06148	1.131733
2306	Computer Games	0	8	130.3	35	2.06148	1.131733
2324	Computer Games	0	8	133.9	52.2	2.91145	1.418402
2324	Computer Games	0	8	133.9	52.2	2.91145	1.418402
2515	Computer Games	0	8	138.9	36.4	1.886674	1.185517
2546	Computer Games	0	8	137.2	37.5	1.992155	1.198785
2312	Computer Games	0	8	141	52.9	2.660832	1.45816
2312	Computer Games	0	8	141	52.9	2.660832	1.45816
2221	Computer Games	0	8	140.7	35.2	1.778093	1.170293
2221	Computer Games	0	8	140.7	35.2	1.778093	1.170293
2272	Computer Games	0	8	133.5	30.2	1.694511	1.055526
2242	Computer Games	1	8	148.9	57.7	2.602474	1.561263
2242	Computer Games	1	8	148.9	57.7	2.602474	1.561263
2276	Computer Games	1	8	136	49	2.649221	1.379439
2513	Computer Games	1	8	142.1	28.9	1.431231	1.05667
2204	Computer Games	0	9	134.6	39.4	2.174734	1.221776
2204	Computer Games	0	9	134.6	39.4	2.174734	1.221776
2514	Computer Games	0	9	137.2	31.4	1.668097	1.089623
2287	Computer Games	0	9	139.6	34.1	1.74978	1.146907
2287	Computer Games	0	9	139.6	34.1	1.74978	1.146907
2233	Computer Games	0	9	138.1	30	1.573019	1.06598
2233	Computer Games	0	9	138.1	30	1.573019	1.06598
2285	Computer Games	0	9	135	33.3	1.82716	1.117417
2285	Computer Games	0	9	135	33.3	1.82716	1.117417
2522	Computer Games	0	9	136.9	66.3	3.537583	1.627274
2551	Computer Games	0	9	133.4	35.1	1.972402	1.144085
2553	Computer Games	0	9	149	78.4	3.531372	1.841597
2321	Computer Games	0	9	133.8	38.3	2.139373	1.200471
2543	Computer Games	0	9	141.9	49.9	2.478197	1.416657
2241	Computer Games	1	9	144	60.7	2.927276	1.583267
2241	Computer Games	1	9	144	60.7	2.927276	1.583267
2320	Computer Games	1	9	132.3	43	2.456681	1.271879
2225	Computer Games	1	9	139.5	30	1.541604	1.07025
2225	Computer Games	1	9	139.5	30	1.541604	1.07025
2255	Computer Games	1	9	132.3	32.3	1.845367	1.090475
2506	Computer Games	1	9	135.6	45.6	2.479964	1.32556
2245	Computer Games	1	9	140	35.7	1.821429	1.176875
2532	Computer Games	0	10	144	61.6	2.970679	1.595849

2307	Computer Games	0	10	137.3	42.9	2.275706	1.289105
2307	Computer Games	0	10	137.3	42.9	2.275706	1.289105
2286	Computer Games	0	10	148.9	63.3	2.855054	1.641008
2286	Computer Games	0	10	148.9	63.3	2.855054	1.641008
2247	Computer Games	0	10	140.1	46.69	2.378743	1.359994
2247	Computer Games	0	10	140.1	46.69	2.378743	1.359994
2243	Computer Games	0	10	148.3	43.6	1.982459	1.340715
2243	Computer Games	0	10	148.3	43.6	1.982459	1.340715
2284	Computer Games	0	10	151.1	50.7	2.220644	1.464853
2294	Computer Games	0	10	139.2	54.2	2.797183	1.469818
2316	Computer Games	0	10	144.6	49	2.343471	1.413378
2216	Computer Games	0	10	151.7	37.8	1.642558	1.252847
2216	Computer Games	0	10	151.7	37.8	1.642558	1.252847
2223	Computer Games	1	10	135.8	31.3	1.697246	1.083342
2223	Computer Games	1	10	135.8	31.3	1.697246	1.083342
2289	Computer Games	1	10	147.3	73.1	3.369084	1.765512
2526	Computer Games	1	10	149.7	61.1	2.72645	1.613508
2230	Computer Games	1	10	147.9	53.2	2.432066	1.490565
2271	Computer Games	1	10	144.3	40.8	1.95942	1.279754
2271	Computer Games	1	10	144.3	40.8	1.95942	1.279754
2240	Computer Games	0	11	150.9	78	3.425438	1.845785
2240	Computer Games	0	11	150.9	78	3.425438	1.845785
2523	Computer Games	0	11	161.3	84.9	3.263164	1.983596
2552	Computer Games	0	11	154.8	57.1	2.382836	1.57661
2213	Computer Games	0	11	147.6	46.9	2.152782	1.391755
2213	Computer Games	0	11	147.6	46.9	2.152782	1.391755
2260	Computer Games	0	11	163.9	71.4	2.65791	1.818688
2260	Computer Games	0	11	163.9	71.4	2.65791	1.818688
2280	Computer Games	0	11	146.2	40.4	1.890108	1.279609
2280	Computer Games	0	11	146.2	40.4	1.890108	1.279609
2229	Computer Games	0	11	139.4	29.6	1.523232	1.06225
2229	Computer Games	0	11	139.4	29.6	1.523232	1.06225
2545	Computer Games	1	11	156.9	56.6	2.299167	1.577575
2244	Computer Games	1	11	148.8	66.9	3.021484	1.690107
2270	Computer Games	1	11	153.1	40.4	1.723578	1.303216
2301	Computer Games	1	11	140.4	48	2.435045	1.381555
2301	Computer Games	1	11	140.4	48	2.435045	1.381555
2530	Computer Games	1	11	142.3	49.7	2.454407	1.415178
2534	Computer Games	1	11	145.7	51.5	2.425985	1.456081
2207	Computer Games	1	11	149.3	39.8	1.785515	1.279955
2207	Computer Games	1	11	149.3	39.8	1.785515	1.279955
2281	Computer Games	1	11	145.6	38	1.792507	1.236133
2281	Computer Games	1	11	145.6	38	1.792507	1.236133
2296	Computer Games	0	12	161.6	75	2.871961	1.857011
2296	Computer Games	0	12	161.6	75	2.871961	1.857011
2203	Computer Games	0	12	147.2	46	2.122962	1.375847
2203	Computer Games	0	12	147.2	46	2.122962	1.375847

2518	Computer Games	0	12	159.2	52.8	2.083281	1.528492
2547	Computer Games	0	12	172.45	94.7	3.18437	2.160104
2220	Computer Games	0	12	164.6	55.5	2.048488	1.59094
2220	Computer Games	0	12	164.6	55.5	2.048488	1.59094
2536	Computer Games	0	12	178.5	118.7	3.725412	2.472688
2215	Computer Games	0	12	156.5	59.9	2.445672	1.624748
2215	Computer Games	0	12	156.5	59.9	2.445672	1.624748
2256	Computer Games	0	12	163.4	86.9	3.254735	2.018916
2256	Computer Games	0	12	163.4	86.9	3.254735	2.018916
2520	Computer Games	1	12	154.2	44.8	1.884123	1.381638
2531	Computer Games	1	12	159.4	86.3	3.39652	1.991743
2211	Computer Games	1	12	155.9	43.7	1.797998	1.369227
2211	Computer Games	1	12	155.9	43.7	1.797998	1.369227
2217	Computer Games	1	12	159.1	54.6	2.157011	1.55591
2217	Computer Games	1	12	159.1	54.6	2.157011	1.55591
2529	Computer Games	1	12	156.6	66.9	2.727989	1.724686
2544	Computer Games	1	12	154.6	49.4	2.066845	1.457704
2227	Computer Games	0	13	145	35.7	1.697979	1.19336
2227	Computer Games	0	13	145	35.7	1.697979	1.19336
2308	Computer Games	0	13	159.3	84.3	3.321972	1.966295
2308	Computer Games	0	13	159.3	84.3	3.321972	1.966295
2274	Computer Games	0	13	140.6	35.6	1.800858	1.177095
2263	Computer Games	0	13	144.3	34.3	1.647257	1.165721
2246	Computer Games	0	13	152	44.4	1.921745	1.36718
2246	Computer Games	0	13	152	44.4	1.921745	1.36718
2254	Computer Games	0	13	154.7	41.8	1.746609	1.332793
2254	Computer Games	0	13	154.7	41.8	1.746609	1.332793
2323	Computer Games	0	13	161.3	96.5	3.709015	2.125031
2323	Computer Games	0	13	161.3	96.5	3.709015	2.125031
2202	Computer Games	0	13	148.9	53	2.390487	1.491528
2202	Computer Games	0	13	148.9	53	2.390487	1.491528
2512	Computer Games	0	13	175.2	52.2	1.7006	1.577903
2548	Computer Games	0	13	153.9	60.7	2.562781	1.625551
2212	Computer Games	0	13	172.4	72.3	2.432561	1.868049
2212	Computer Games	0	13	172.4	72.3	2.432561	1.868049
2315	Computer Games	0	13	157	101.7	4.125928	2.16258
2555	Computer Games	1	13	156.4	72.9	2.980259	1.805305
2291	Computer Games	1	13	157.8	78.1	3.13644	1.880086
2291	Computer Games	1	13	157.8	78.1	3.13644	1.880086
2232	Computer Games	1	13	153.7	42.1	1.782109	1.334494
2232	Computer Games	1	13	153.7	42.1	1.782109	1.334494
2557	Computer Games	1	13	166	53.7	1.948759	1.568236
2288	Computer Games	1	13	159.8	80	3.132827	1.914079
2299	Computer Games	1	13	161.7	94.6	3.618021	2.104492
2299	Computer Games	1	13	161.7	94.6	3.618021	2.104492
2218	Computer Games	0	14	172.8	70.2	2.350984	1.84036
2218	Computer Games	0	14	172.8	70.2	2.350984	1.84036

2550	Computer Games	0	14	170.6	54.2	1.862264	1.593241
2293	Computer Games	0	14	178.2	89	2.802687	2.116518
2559	Computer Games	0	14	154.3	47.6	1.999287	1.427794
2228	Computer Games	0	14	184.6	69.3	2.03362	1.876121
2228	Computer Games	0	14	184.6	69.3	2.03362	1.876121
2535	Computer Games	0	14	185.7	103.6	3.004249	2.334521
2273	Computer Games	1	14	147.9	46	2.102914	1.378437
2259	Computer Games	1	14	162.7	96.2	3.634126	2.128756
2214	Computer Games	1	14	163.2	59.8	2.24523	1.650488
2214	Computer Games	1	14	163.2	59.8	2.24523	1.650488
2502	Computer Games	1	14	161.1	51.6	1.988193	1.516827
2252	Computer Games	1	14	171.9	72.3	2.446733	1.865899
2252	Computer Games	1	14	171.9	72.3	2.446733	1.865899
2258	Computer Games	1	14	169.4	115.1	4.010962	2.382143
2504	Computer Games	1	14	161.1	47.1	1.814804	1.444188
2525	Computer Games	1	14	159.8	72.3	2.831293	1.812687
2262	Computer Games	0	15	175.1	61.7	2.012393	1.725974
2300	Computer Games	0	15	182.3	103.5	3.114347	2.31628
2300	Computer Games	0	15	182.3	103.5	3.114347	2.31628
2517	Computer Games	0	15	166.3	61	2.205694	1.680708
2309	Computer Games	0	15	164.4	102	3.773954	2.205916
2516	Computer Games	0	15	175	72.6	2.370612	1.883355
2201	Computer Games	0	15	162.6	64.3	2.432035	1.713659
2201	Computer Games	0	15	162.6	64.3	2.432035	1.713659
2257	Computer Games	0	15	170.8	61.2	2.097856	1.701584
2257	Computer Games	0	15	170.8	61.2	2.097856	1.701584
2540	Computer Games	0	15	169.4	92.8	3.23386	2.121626
2219	Computer Games	0	15	174.9	61	1.994115	1.714638
2219	Computer Games	0	15	174.9	61	1.994115	1.714638
2206	Computer Games	1	15	162.5	51.7	1.95787	1.523624
2206	Computer Games	1	15	162.5	51.7	1.95787	1.523624
2298	Computer Games	1	15	153.5	107.4	4.558139	2.207129
2298	Computer Games	1	15	153.5	107.4	4.558139	2.207129
2511	Computer Games	1	15	161.8	62.4	2.383568	1.682944
2205	Computer Games	1	15	156.9	52.2	2.120433	1.510388
2205	Computer Games	1	15	156.9	52.2	2.120433	1.510388
2292	Computer Games	1	15	170.2	91.3	3.151749	2.107045
2541	Computer Games	1	15	165.2	93.1	3.411376	2.104266
2542	Computer Games	0	16	174.5	105.2	3.454816	2.296509
2505	Computer Games	0	16	178.2	70.6	2.223255	1.868647
2253	Computer Games	0	16	175.4	57.2	1.859246	1.658216
2253	Computer Games	0	16	175.4	57.2	1.859246	1.658216
2290	Computer Games	0	16	171	122.5	4.189323	2.472518
2290	Computer Games	0	16	171	122.5	4.189323	2.472518
2251	Computer Games	1	16	154.7	130.8	5.465465	2.461534
2251	Computer Games	1	16	154.7	130.8	5.465465	2.461534
2311	Computer Games	1	16	166.7	80.9	2.911235	1.95817

2311	Computer Games	1	16	166.7	80.9	2.911235	1.95817
2549	Computer Games	1	16	164.7	47.9	1.765828	1.470151
2264	Computer Games	0	17	194.2	77.3	2.049656	2.030043
2264	Computer Games	0	17	194.2	77.3	2.049656	2.030043
2208	Computer Games	0	17	177	69.8	2.227968	1.852262
2208	Computer Games	0	17	177	69.8	2.227968	1.852262
2501	Computer Games	0	17	188.2	76.6	2.162666	1.99516
2556	Computer Games	0	17	172.7	74.2	2.487822	1.895598
2226	Computer Games	0	17	177.2	65.6	2.089183	1.792265
2226	Computer Games	0	17	177.2	65.6	2.089183	1.792265
2318	Computer Games	0	17	173.4	112.8	3.751552	2.378325
2318	Computer Games	0	17	173.4	112.8	3.751552	2.378325
2209	Computer Games	1	17	164.9	52.5	1.930714	1.545213
2209	Computer Games	1	17	164.9	52.5	1.930714	1.545213
2210	Computer Games	1	17	158.2	50.4	2.013806	1.487007
2234	Computer Games	1	17	162.2	46.8	1.77887	1.443121
2234	Computer Games	1	17	162.2	46.8	1.77887	1.443121
2305	Computer Games	1	17	166.5	90.9	3.278955	2.083841
2305	Computer Games	1	17	166.5	90.9	3.278955	2.083841
2524	Computer Games	1	17	157.2	74.2	3.002609	1.826238
2249	Computer Games	1	17	160.2	74.4	2.898998	1.84264
2249	Computer Games	1	17	160.2	74.4	2.898998	1.84264
2313	Computer Games	1	17	163.2	81.9	3.074989	1.954639
2537	Computer Games	0	18	178.5	105.6	3.314267	2.321968
2317	Computer Games	0	18	168.4	86.6	3.053752	2.039386
2317	Computer Games	0	18	168.4	86.6	3.053752	2.039386
2503	Computer Games	1	18	163.3	52	1.949984	1.531351
2314	Computer Games	1	18	161.5	86.6	3.320266	2.005843
2303	Computer Games	1	18	153.9	49.1	2.073024	1.450325
3079	Computer Games	0	5	114.5	21.033	1.604317	0.817612
4054	Computer Games	1	5	120.667	19.967	1.37131	0.811765
4090	Computer Games	1	5	110.567	15.5	1.267887	0.68428
3042	Computer Games	0	6	120.9	23.3	1.594055	0.88271
3078	Computer Games	0	6	114.35	19.5	1.491291	0.784595
4060	Computer Games	0	6	121.633	22.6	1.527586	0.870431
4066	Computer Games	0	6	119.533	21.4	1.497746	0.839446
3103	Computer Games	0	6	129.5	27.225	1.623411	0.986302
4077	Computer Games	1	6	110.867	18.7	1.521378	0.757768
4087	Computer Games	1	6	119.033	31.617	2.231444	1.03379
4073	Computer Games	1	6	121.4	23.7	1.608092	0.892286
3133	Computer Games	1	6	124.55	23.8	1.534227	0.903437
4075	Computer Games	1	6	130.567	28.617	1.678639	1.01641
4047	Computer Games	1	6	127.633	24.3	1.491696	0.922493
3122	Computer Games	1	6	131.4	24.6	1.424769	0.939369
3135	Computer Games	0	7	120.45	23.475	1.61805	0.88496
3108	Computer Games	0	7	123	27.1	1.791262	0.963983
3005	Computer Games	0	7	127	25.067	1.554157	0.936193

3049	Computer Games	0	7	122.35	23.2	1.549816	0.884842
3106	Computer Games	0	7	130.95	41.075	2.395337	1.235899
4029	Computer Games	0	7	128.3	27.05	1.643289	0.979267
4014	Computer Games	0	7	118.7	20.2	1.433672	0.811541
4063	Computer Games	0	7	141.033	55.683	2.799504	1.499066
3028	Computer Games	0	7	127.3	24.55	1.514937	0.926625
4053	Computer Games	0	7	128.567	28.567	1.728246	1.00926
3043	Computer Games	0	7	129	27.475	1.651043	0.989643
3064	Computer Games	0	7	129.2	32.1	1.923003	1.076671
4078	Computer Games	1	7	126.8	25.967	1.61504	0.953527
3080	Computer Games	1	7	116.45	21.35	1.574414	0.829752
4006	Computer Games	1	7	129.333	25.267	1.51055	0.947011
4071	Computer Games	1	7	122	21.617	1.452365	0.850876
3119	Computer Games	0	8	131.8	28.483	1.639664	1.017633
3003	Computer Games	0	8	130.5	32.275	1.895157	1.084118
3054	Computer Games	0	8	135.9	66.433	3.597037	1.624301
4032	Computer Games	0	8	129.767	26.8	1.591499	0.97879
4084	Computer Games	0	8	136.667	27.417	1.467887	1.011404
4021	Computer Games	0	8	132.667	26.583	1.510352	0.983095
3101	Computer Games	0	8	137	36.075	1.922052	1.173389
4097	Computer Games	0	8	138.033	33.75	1.771364	1.135469
3104	Computer Games	0	8	132.167	29.467	1.686902	1.037534
4061	Computer Games	0	8	135.933	31.55	1.707456	1.088409
3033	Computer Games	1	8	125.3	24.7	1.573239	0.923848
4088	Computer Games	1	8	128.033	25.117	1.53223	0.940211
3001	Computer Games	1	8	125.5	24.05	1.52696	0.911269
4030	Computer Games	1	8	131.733	31.583	1.81997	1.075557
4049	Computer Games	1	8	123.367	28.55	1.875893	0.992559
4002	Computer Games	1	8	138.267	33.2	1.736605	1.126235
3066	Computer Games	1	8	132.05	28.975	1.661677	1.02782
3008	Computer Games	1	8	134.6	31.8	1.755242	1.088777
4003	Computer Games	1	8	128.367	21.517	1.305795	0.866047
4035	Computer Games	1	8	157.033	63.383	2.570342	1.677151
3039	Computer Games	1	8	126.55	22.65	1.414308	0.885264
4033	Computer Games	1	8	132.467	32.033	1.825502	1.086161
4034	Computer Games	1	8	131.233	31.333	1.819348	1.069356
3111	Computer Games	0	9	136.5	34.875	1.871755	1.150565
4062	Computer Games	0	9	138.367	39.617	2.069267	1.238872
4005	Computer Games	0	9	134.233	31.9	1.770403	1.089438
4042	Computer Games	0	9	132.967	31.65	1.790136	1.08077
3068	Computer Games	0	9	141.1	38.525	1.935034	1.229887
3100	Computer Games	0	9	128.45	26.925	1.631877	0.977283
3127	Computer Games	0	9	147.5	35.7	1.640908	1.201474
3132	Computer Games	0	9	135.267	32.95	1.800826	1.111955
3091	Computer Games	0	9	140.45	36.875	1.869341	1.199075
3048	Computer Games	1	9	134.367	32.483	1.799165	1.100536
4027	Computer Games	1	9	139.867	41.717	2.132468	1.279223

3061	Computer Games	1	9	138.167	34.2	1.791503	1.144026
4064	Computer Games	1	9	133.233	39.933	2.249616	1.225668
4023	Computer Games	1	9	134.333	30.917	1.713294	1.071569
4104	Computer Games	1	9	133.133	32.633	1.841135	1.099238
4092	Computer Games	1	9	153	61.65	2.633602	1.635377
3056	Computer Games	1	9	134.15	30.375	1.687854	1.060851
4020	Computer Games	1	9	137.2	30.8	1.636223	1.078376
3006	Computer Games	1	9	134.75	29.375	1.617783	1.043768
3072	Computer Games	1	9	132.15	28.2	1.614785	1.013247
3118	Computer Games	1	9	146.95	42.425	1.964639	1.31638
4017	Computer Games	1	9	140.5	46.083	2.334469	1.351984
4102	Computer Games	1	9	136.8	33.217	1.774959	1.121792
3027	Computer Games	0	10	137.75	32.9	1.733855	1.119088
4013	Computer Games	0	10	149.8	45.617	2.03284	1.379214
3125	Computer Games	0	10	153.5	41.817	1.774746	1.328976
4069	Computer Games	0	10	133.95	26.45	1.474145	0.984194
3029	Computer Games	0	10	151.85	36.575	1.586188	1.231328
4028	Computer Games	0	10	148.667	50.267	2.274332	1.448759
4055	Computer Games	0	10	147.633	41.6	1.908651	1.304947
4041	Computer Games	0	10	135.467	38.3	2.087045	1.206378
3126	Computer Games	0	10	144	31.3	1.509452	1.108815
3022	Computer Games	0	10	144.8	32.875	1.567937	1.140983
4025	Computer Games	0	10	147.633	34.767	1.595146	1.184907
3070	Computer Games	0	10	140	35.7	1.821429	1.176875
4052	Computer Games	0	10	140.867	42.75	2.154357	1.299828
4040	Computer Games	1	10	150.667	59.333	2.613726	1.592302
3041	Computer Games	1	10	142.45	36.583	1.802828	1.20067
4100	Computer Games	1	10	138.3	34.45	1.801129	1.148954
4070	Computer Games	1	10	138.5	38.483	2.006178	1.220137
3123	Computer Games	1	10	145.45	33.125	1.565772	1.147677
4048	Computer Games	1	10	132.8	24.033	1.362736	0.931568
3114	Computer Games	1	10	140.55	27.5	1.392102	1.024362
4105	Computer Games	1	10	151.533	52.033	2.266024	1.487127
4068	Computer Games	1	10	150.867	56.45	2.480136	1.551029
4081	Computer Games	1	10	142.733	35.6	1.747436	1.184141
3086	Computer Games	0	11	144	38.4	1.851852	1.23768
3102	Computer Games	0	11	149.6	38.05	1.700167	1.250368
4050	Computer Games	0	11	142.533	52.433	2.580916	1.457457
4026	Computer Games	0	11	158.633	61.25	2.433991	1.653187
3128	Computer Games	0	11	145.067	35.917	1.706722	1.197475
3002	Computer Games	0	11	141.55	31.225	1.558413	1.099878
3034	Computer Games	0	11	142.8	42.525	2.085393	1.303165
3052	Computer Games	0	11	154.4	41.9	1.757598	1.333481
4019	Computer Games	0	11	144.633	34.1	1.63012	1.163123
3038	Computer Games	0	11	143.3	34.6	1.684937	1.167969
4037	Computer Games	0	11	138.033	33.6	1.763492	1.132752
4010	Computer Games	0	11	144.567	48.3	2.311047	1.402357

3017	Computer Games	1	11	133.9	29.533	1.6472	1.044161
4096	Computer Games	1	11	155.8	77.033	3.173525	1.856816
3060	Computer Games	1	11	157.75	63.875	2.566801	1.687183
3000	Computer Games	1	11	151	52.275	2.292663	1.488762
4089	Computer Games	1	11	157.5	54.6	2.201058	1.549689
4101	Computer Games	1	11	137.633	28.2	1.488689	1.029708
3120	Computer Games	1	11	157.667	43.3	1.741832	1.368573
4016	Computer Games	1	11	148.7	42.067	1.902478	1.316558
4083	Computer Games	1	11	164.633	61.1	2.254278	1.675485
3009	Computer Games	1	11	164.5	58.367	2.156928	1.63423
3113	Computer Games	0	12	157.05	62.475	2.532972	1.664258
3134	Computer Games	0	12	164.25	76.775	2.845831	1.892683
4008	Computer Games	0	12	135.9	27.967	1.514283	1.019987
4038	Computer Games	0	12	154.6	37.633	1.574526	1.259285
4039	Computer Games	0	12	155.967	40.733	1.674484	1.318644
4085	Computer Games	0	12	150.533	56.633	2.499229	1.552367
4046	Computer Games	0	12	153.633	36.45	1.544289	1.234763
4108	Computer Games	0	12	135.633	28.783	1.564607	1.035079
3095	Computer Games	0	12	160.9	78.967	3.050237	1.905923
4031	Computer Games	0	12	154.733	52.733	2.202503	1.510313
3058	Computer Games	0	12	175.4	65.95	2.143659	1.790141
3088	Computer Games	0	12	156.5	49.283	2.012188	1.462912
3004	Computer Games	0	12	159.95	48.775	1.906465	1.467412
4076	Computer Games	1	12	156.433	49.467	2.021431	1.465598
3124	Computer Games	1	12	167.65	50.217	1.786667	1.518628
3065	Computer Games	1	12	152.95	33.95	1.451245	1.186373
4109	Computer Games	1	12	157.067	51.433	2.084836	1.499044
3085	Computer Games	1	12	158	49.3	1.974844	1.468727
4098	Computer Games	1	12	158.133	46.733	1.868868	1.427567
4099	Computer Games	1	12	158.9	48.85	1.934714	1.464796
4103	Computer Games	1	12	151.467	40.75	1.776199	1.303722
4106	Computer Games	1	12	157.067	46.983	1.904456	1.427835
4107	Computer Games	1	12	152.767	50.117	2.147464	1.462115
4110	Computer Games	1	12	152.467	41.917	1.803178	1.327128
3094	Computer Games	1	12	168.7	51.575	1.812212	1.544396
4079	Computer Games	1	12	152.133	59.8	2.583773	1.605179
4057	Computer Games	0	13	158.267	47.45	1.894329	1.439788
3131	Computer Games	0	13	159.1	45.875	1.812323	1.416831
4056	Computer Games	0	13	159.533	51.317	2.016323	1.506499
4074	Computer Games	0	13	171.533	62.25	2.115651	1.72014
3087	Computer Games	0	13	167.5	48.275	1.72065	1.486229
3014	Computer Games	0	13	169.8	53.75	1.864245	1.583161
3020	Computer Games	0	13	147.5	33.7	1.54898	1.164793
3024	Computer Games	0	13	173.15	56.45	1.882864	1.638083
4091	Computer Games	0	13	168.5	66.033	2.32574	1.76308
3035	Computer Games	0	13	165.6	47.9	1.746686	1.473331
3115	Computer Games	0	13	152.25	38.525	1.661989	1.26753

3044	Computer Games	0	13	160.233	43.9	1.70986	1.387591
4086	Computer Games	0	13	167.767	83.95	2.982687	2.002591
3110	Computer Games	0	13	169.75	64.033	2.222206	1.739245
4012	Computer Games	0	13	156.467	62.25	2.542693	1.658585
3010	Computer Games	1	13	173.75	67.35	2.23094	1.803709
4022	Computer Games	1	13	158.967	49.317	1.951564	1.472557
3069	Computer Games	1	13	163.25	49.675	1.863938	1.493958
3015	Computer Games	1	13	164.5	45	1.662956	1.420909
4093	Computer Games	1	13	155.333	53.483	2.216605	1.524164
4067	Computer Games	1	13	159.167	59.85	2.362425	1.634933
4094	Computer Games	1	13	167.167	79.983	2.862178	1.948361
4043	Computer Games	1	13	162.8	54.133	2.042459	1.562917
4080	Computer Games	1	13	157.433	52.817	2.130994	1.522007
3021	Computer Games	0	14	159.75	48.225	1.88969	1.457767
4045	Computer Games	0	14	164.7	56.733	2.091455	1.61024
4007	Computer Games	0	14	163.933	51.367	1.911399	1.523632
3117	Computer Games	0	14	162.05	53.35	2.031591	1.547883
3055	Computer Games	0	14	184.6	71.3	2.092311	1.905049
3096	Computer Games	0	14	170.567	106.5	3.660662	2.290931
3081	Computer Games	0	14	179.8	68.05	2.104984	1.838543
3047	Computer Games	0	14	174.3	77.85	2.5625	1.952313
3097	Computer Games	0	14	182.3	97.35	2.929292	2.241213
3053	Computer Games	1	14	170.6	50.125	1.722251	1.527658
3098	Computer Games	1	14	159.75	44.825	1.756461	1.401561
3025	Computer Games	1	14	156.35	51.275	2.097538	1.493853
4015	Computer Games	1	14	154.667	45.9	1.918745	1.401459
3112	Computer Games	1	14	160.3	69.25	2.694963	1.773347
4051	Computer Games	1	14	158.967	58.9	2.33078	1.620117
4082	Computer Games	1	14	168.133	47.517	1.680905	1.475837
4095	Computer Games	1	14	167.4	86.733	3.095091	2.036257
4024	Computer Games	1	14	175.533	54.767	1.777466	1.62039
3011	Computer Games	1	14	170.25	60.675	2.09332	1.691554
4036	Computer Games	1	14	157	72.7	2.94941	1.805378
4065	Computer Games	0	15	170	77.817	2.69263	1.932636
3023	Computer Games	0	15	174.15	71.4	2.354243	1.86295
4009	Computer Games	0	15	190.4	117.15	3.231532	2.518894
3075	Computer Games	1	15	166.5	68.125	2.457412	1.784437
3093	Computer Games	1	15	166.2	53.925	1.952217	1.572516
3016	Computer Games	1	15	159.4	61.633	2.425698	1.661913
3026	Computer Games	0	16	180.4	71.4	2.193942	1.889171
3032	Computer Games	0	16	170.3	59.7	2.058472	1.677076
3084	Computer Games	1	16	165.65	54.125	1.972491	1.573581
4011	Computer Games	1	16	163.633	62.1	2.319262	1.6861
4058	Computer Games	1	16	165.733	68.533	2.495064	1.786903
1610	Computer Games	0	11	146.1	45.2	2.117571	1.35888
1512	Computer Games	0	11	142.8	36.9	1.809547	1.207428
1503	Computer Games	0	11	139.2	31.5	1.625669	1.097767

1541	Computer Games	0	11	148.7	43.5	1.967285	1.340491
1537	Computer Games	0	11	146.4	82.1	3.830549	1.874712
1540	Computer Games	0	11	163.5	63.6	2.379149	1.707332
1535	Computer Games	0	11	148.9	51	2.30028	1.460989
1577	Computer Games	1	11	143.9	35.9	1.733696	1.193343
1508	Computer Games	1	11	166.6	42.6	1.534828	1.386592
1509	Computer Games	1	11	143.3	33.3	1.62163	1.14416
1576	Computer Games	0	12	164.9	78.9	2.901588	1.923688
1561	Computer Games	0	12	164.8	58.4	2.150297	1.635908
1548	Computer Games	0	12	161.2	80.1	3.082495	1.922
1536	Computer Games	0	12	153.7	49.5	2.095354	1.455917
1598	Computer Games	0	12	161.7	59	2.256483	1.63259
1547	Computer Games	0	12	156.1	55	2.257132	1.550288
1511	Computer Games	0	12	163.4	72.1	2.700419	1.826043
1552	Computer Games	0	12	154.6	55.4	2.317879	1.550395
1578	Computer Games	0	12	164.7	88.6	3.266228	2.04648
1564	Computer Games	0	12	160.9	80.2	3.097864	1.92187
1614	Computer Games	0	12	170.7	78.1	2.680304	1.93957
1506	Computer Games	1	12	161.2	51.2	1.970334	1.510863
1550	Computer Games	1	12	159.2	41.4	1.633481	1.341076
1546	Computer Games	1	12	145.4	32.7	1.546746	1.139579
1527	Computer Games	1	12	158.2	86.3	3.448243	1.985786
1524	Computer Games	1	12	154.8	82.7	3.451148	1.924155
1502	Computer Games	1	12	152.6	47.2	2.026903	1.415101
1574	Computer Games	1	12	163.4	85.2	3.191064	1.997578
1604	Computer Games	1	12	154.5	47.9	2.006682	1.433362
1525	Computer Games	1	12	154.8	48.9	2.040643	1.450494
1572	Computer Games	0	13	157.8	63.7	2.558147	1.684908
1599	Computer Games	0	13	166.2	50.2	1.817363	1.513132
1566	Computer Games	0	13	169.8	62.1	2.153854	1.711009
1521	Computer Games	0	13	160.7	45.8	1.77351	1.421211
1556	Computer Games	0	13	156.8	48.3	1.964513	1.448246
1544	Computer Games	1	13	162.2	67	2.546673	1.75028
1580	Computer Games	1	13	128	44.5	2.716064	1.278693
1569	Computer Games	1	13	151.6	44.7	1.944953	1.370708
1545	Computer Games	1	13	155.1	61.3	2.54822	1.639212
1605	Computer Games	0	14	174.6	56.2	1.84352	1.639588
2562	Dance	0	5	104.7	15.5	1.413964	0.669649
2561	Dance	0	5	121.7	27.9	1.883749	0.975069
2563	Dance	0	5	120.2	21	1.453484	0.832806
2564	Dance	0	5	113.7	20.6	1.593479	0.806272
2510	Dance	1	5	105.2	18.7	1.689702	0.742171
2519	Dance	1	6	125.8	48.5	3.064647	1.330104
2528	Dance	1	6	122	30.8	2.069336	1.029333
2560	Dance	0	7	117.9	22	1.582687	0.84739
2507	Dance	0	7	117.25	21.7	1.578462	0.839314
2508	Dance	0	7	122.8	23	1.525215	0.882015

2527	Dance	1	7	119.9	29.4	2.045074	0.997014
2554	Dance	1	7	131.6	41.4	2.390499	1.243587
2509	Dance	1	7	120.4	28.7	1.979835	0.985802
2546	Dance	0	8	137.2	37.5	1.992155	1.198785
2513	Dance	1	8	142.1	28.9	1.431231	1.05667
2514	Dance	0	9	137.2	31.4	1.668097	1.089623
2522	Dance	0	9	136.9	66.3	3.537583	1.627274
2551	Dance	0	9	133.4	35.1	1.972402	1.144085
2553	Dance	0	9	149	78.4	3.531372	1.841597
2543	Dance	0	9	141.9	49.9	2.478197	1.416657
2506	Dance	1	9	135.6	45.6	2.479964	1.32556
2282	Dance	1	5	107.1	15.8	1.377457	0.682695
2283	Dance	1	5	106.8	17.4	1.525481	0.718246
2279	Dance	0	6	127.6	25.6	1.572312	0.948617
2231	Dance	0	7	121.3	21.6	1.468021	0.848578
2222	Dance	0	7	120.6	20.3	1.39573	0.818838
2224	Dance	0	7	127.8	25.1	1.536781	0.93919
2275	Dance	1	7	118	33.8	2.427463	1.067888
2261	Dance	1	7	120.8	26.2	1.795426	0.939884
2306	Dance	0	8	130.3	35	2.06148	1.131733
2324	Dance	0	8	133.9	52.2	2.91145	1.418402
2312	Dance	0	8	141	52.9	2.660832	1.45816
2221	Dance	0	8	140.7	35.2	1.778093	1.170293
2272	Dance	0	8	133.5	30.2	1.694511	1.055526
2242	Dance	1	8	148.9	57.7	2.602474	1.561263
2276	Dance	1	8	136	49	2.649221	1.379439
2204	Dance	0	9	134.6	39.4	2.174734	1.221776
2287	Dance	0	9	139.6	34.1	1.74978	1.146907
2233	Dance	0	9	138.1	30	1.573019	1.06598
2285	Dance	0	9	135	33.3	1.82716	1.117417
2241	Dance	1	9	144	60.7	2.927276	1.583267
2225	Dance	1	9	139.5	30	1.541604	1.07025
2255	Dance	1	9	132.3	32.3	1.845367	1.090475
2245	Dance	1	9	140	35.7	1.821429	1.176875
2532	Dance	0	10	144	61.6	2.970679	1.595849
2526	Dance	1	10	149.7	61.1	2.72645	1.613508
2523	Dance	0	11	161.3	84.9	3.263164	1.983596
2552	Dance	0	11	154.8	57.1	2.382836	1.57661
2545	Dance	1	11	156.9	56.6	2.299167	1.577575
2530	Dance	1	11	142.3	49.7	2.454407	1.415178
2534	Dance	1	11	145.7	51.5	2.425985	1.456081
2518	Dance	0	12	159.2	52.8	2.083281	1.528492
2547	Dance	0	12	172.45	94.7	3.18437	2.160104
2536	Dance	0	12	178.5	118.7	3.725412	2.472688
2520	Dance	1	12	154.2	44.8	1.884123	1.381638
2531	Dance	1	12	159.4	86.3	3.39652	1.991743
2529	Dance	1	12	156.6	66.9	2.727989	1.724686

2544	Dance	1	12	154.6	49.4	2.066845	1.457704
2307	Dance	0	10	137.3	42.9	2.275706	1.289105
2286	Dance	0	10	148.9	63.3	2.855054	1.641008
2247	Dance	0	10	140.1	46.69	2.378743	1.359994
2243	Dance	0	10	148.3	43.6	1.982459	1.340715
2284	Dance	0	10	151.1	50.7	2.220644	1.464853
2316	Dance	0	10	144.6	49	2.343471	1.413378
2216	Dance	0	10	151.7	37.8	1.642558	1.252847
2223	Dance	1	10	135.8	31.3	1.697246	1.083342
2289	Dance	1	10	147.3	73.1	3.369084	1.765512
2230	Dance	1	10	147.9	53.2	2.432066	1.490565
2271	Dance	1	10	144.3	40.8	1.95942	1.279754
2240	Dance	0	11	150.9	78	3.425438	1.845785
2213	Dance	0	11	147.6	46.9	2.152782	1.391755
2260	Dance	0	11	163.9	71.4	2.65791	1.818688
2280	Dance	0	11	146.2	40.4	1.890108	1.279609
2229	Dance	0	11	139.4	29.6	1.523232	1.06225
2244	Dance	1	11	148.8	66.9	3.021484	1.690107
2270	Dance	1	11	153.1	40.4	1.723578	1.303216
2301	Dance	1	11	140.4	48	2.435045	1.381555
2207	Dance	1	11	149.3	39.8	1.785515	1.279955
2281	Dance	1	11	145.6	38	1.792507	1.236133
2296	Dance	0	12	161.6	75	2.871961	1.857011
2203	Dance	0	12	147.2	46	2.122962	1.375847
2220	Dance	0	12	164.6	55.5	2.048488	1.59094
2215	Dance	0	12	156.5	59.9	2.445672	1.624748
2256	Dance	0	12	163.4	86.9	3.254735	2.018916
2211	Dance	1	12	155.9	43.7	1.797998	1.369227
2217	Dance	1	12	159.1	54.6	2.157011	1.55591
2512	Dance	0	13	175.2	52.2	1.7006	1.577903
2548	Dance	0	13	153.9	60.7	2.562781	1.625551
2555	Dance	1	13	156.4	72.9	2.980259	1.805305
2557	Dance	1	13	166	53.7	1.948759	1.568236
2550	Dance	0	14	170.6	54.2	1.862264	1.593241
2559	Dance	0	14	154.3	47.6	1.999287	1.427794
2535	Dance	0	14	185.7	103.6	3.004249	2.334521
2502	Dance	1	14	161.1	51.6	1.988193	1.516827
2504	Dance	1	14	161.1	47.1	1.814804	1.444188
2525	Dance	1	14	159.8	72.3	2.831293	1.812687
2517	Dance	0	15	166.3	61	2.205694	1.680708
2516	Dance	0	15	175	72.6	2.370612	1.883355
2540	Dance	0	15	169.4	92.8	3.23386	2.121626
2511	Dance	1	15	161.8	62.4	2.383568	1.682944
2541	Dance	1	15	165.2	93.1	3.411376	2.104266
2227	Dance	0	13	145	35.7	1.697979	1.19336
2308	Dance	0	13	159.3	84.3	3.321972	1.966295
2274	Dance	0	13	140.6	35.6	1.800858	1.177095

2263	Dance	0	13	144.3	34.3	1.647257	1.165721
2246	Dance	0	13	152	44.4	1.921745	1.36718
2254	Dance	0	13	154.7	41.8	1.746609	1.332793
2323	Dance	0	13	161.3	96.5	3.709015	2.125031
2202	Dance	0	13	148.9	53	2.390487	1.491528
2212	Dance	0	13	172.4	72.3	2.432561	1.868049
2315	Dance	0	13	157	101.7	4.125928	2.16258
2291	Dance	1	13	157.8	78.1	3.13644	1.880086
2232	Dance	1	13	153.7	42.1	1.782109	1.334494
2288	Dance	1	13	159.8	80	3.132827	1.914079
2299	Dance	1	13	161.7	94.6	3.618021	2.104492
2218	Dance	0	14	172.8	70.2	2.350984	1.84036
2293	Dance	0	14	178.2	89	2.802687	2.116518
2228	Dance	0	14	184.6	69.3	2.03362	1.876121
2273	Dance	1	14	147.9	46	2.102914	1.378437
2259	Dance	1	14	162.7	96.2	3.634126	2.128756
2214	Dance	1	14	163.2	59.8	2.24523	1.650488
2252	Dance	1	14	171.9	72.3	2.446733	1.865899
2258	Dance	1	14	169.4	115.1	4.010962	2.382143
2262	Dance	0	15	175.1	61.7	2.012393	1.725974
2300	Dance	0	15	182.3	103.5	3.114347	2.31628
2309	Dance	0	15	164.4	102	3.773954	2.205916
2201	Dance	0	15	162.6	64.3	2.432035	1.713659
2257	Dance	0	15	170.8	61.2	2.097856	1.701584
2219	Dance	0	15	174.9	61	1.994115	1.714638
2206	Dance	1	15	162.5	51.7	1.95787	1.523624
2298	Dance	1	15	153.5	107.4	4.558139	2.207129
2205	Dance	1	15	156.9	52.2	2.120433	1.510388
2292	Dance	1	15	170.2	91.3	3.151749	2.107045
2542	Dance	0	16	174.5	105.2	3.454816	2.296509
2505	Dance	0	16	178.2	70.6	2.223255	1.868647
2549	Dance	1	16	164.7	47.9	1.765828	1.470151
2501	Dance	0	17	188.2	76.6	2.162666	1.99516
2556	Dance	0	17	172.7	74.2	2.487822	1.895598
2524	Dance	1	17	157.2	74.2	3.002609	1.826238
2537	Dance	0	18	178.5	105.6	3.314267	2.321968
2503	Dance	1	18	163.3	52	1.949984	1.531351
2253	Dance	0	16	175.4	57.2	1.859246	1.658216
2290	Dance	0	16	171	122.5	4.189323	2.472518
2251	Dance	1	16	154.7	130.8	5.465465	2.461534
2311	Dance	1	16	166.7	80.9	2.911235	1.95817
2264	Dance	0	17	194.2	77.3	2.049656	2.030043
2208	Dance	0	17	177	69.8	2.227968	1.852262
2226	Dance	0	17	177.2	65.6	2.089183	1.792265
2318	Dance	0	17	173.4	112.8	3.751552	2.378325
2209	Dance	1	17	164.9	52.5	1.930714	1.545213
2210	Dance	1	17	158.2	50.4	2.013806	1.487007

2234	Dance	1	17	162.2	46.8	1.77887	1.443121
2305	Dance	1	17	166.5	90.9	3.278955	2.083841
2249	Dance	1	17	160.2	74.4	2.898998	1.84264
2313	Dance	1	17	163.2	81.9	3.074989	1.954639
2317	Dance	0	18	168.4	86.6	3.053752	2.039386
2314	Dance	1	18	161.5	86.6	3.320266	2.005843
2303	Dance	1	18	153.9	49.1	2.073024	1.450325
1143	Dance	0	8	132.6	27.2	1.54697	0.995102
1181	Dance	1	8	146.2	61.9	2.895982	1.609669
1148	Dance	1	8	129.9	40.5	2.400141	1.222656
1151	Dance	0	9	128.3	24.4	1.482301	0.926445
1305	Dance	0	9	139.1	31.2	1.612502	1.091821
1312	Dance	0	9	143.6	44.3	2.1483	1.335095
1308	Dance	1	9	135.8	35.1	1.903301	1.1522
1307	Dance	1	9	133.4	29.5	1.657717	1.041987
1128	Dance	1	9	158.7	63.7	2.529214	1.68871
1166	Dance	0	10	121	24.8	1.693873	0.91313
1137	Dance	0	10	145.7	35	1.648728	1.182975
1309	Dance	0	10	137.8	33.4	1.758928	1.128365
1127	Dance	1	10	132.8	43.3	2.455228	1.278554
1163	Dance	1	10	146.3	36.6	1.709986	1.213733
1255	Dance	1	10	154.5	54	2.26223	1.528808
1327	Dance	0	11	146.8	46.3	2.148468	1.379177
1318	Dance	0	11	147.5	33.8	1.553577	1.166651
1330	Dance	0	11	156.5	47	1.918974	1.426067
1325	Dance	0	11	145.5	39.6	1.870549	1.263513
1202	Dance	0	11	141.1	27.9	1.401361	1.033948
1232	Dance	0	11	157.8	54.5	2.188681	1.54933
1188	Dance	1	11	149.7	55.1	2.458714	1.526264
1217	Dance	1	11	139.6	31.1	1.595841	1.091489
1250	Dance	1	11	147.9	42.8	1.956624	1.326008
1323	Dance	0	12	144.7	41.1	1.96293	1.286217
1115	Dance	0	12	161	68.9	2.658076	1.771578
1322	Dance	0	12	155.4	52.4	2.169848	1.507745
1233	Dance	1	12	154.8	42.1	1.756872	1.338272
1216	Dance	1	12	143.9	32.2	1.555014	1.125539
1214	Dance	1	12	155.5	46.8	1.935464	1.41919
1117	Dance	1	12	158.7	51.2	2.0329	1.501531
1195	Dance	1	12	150.9	41.3	1.813726	1.311205
1213	Dance	0	13	151.2	40.3	1.762794	1.295053
1275	Dance	0	13	148.1	38.8	1.768975	1.258526
1272	Dance	0	13	163.6	76.8	2.869423	1.890041
1302	Dance	0	14	171.2	53.4	1.821939	1.582752
1269	Dance	0	15	172.3	76.3	2.570123	1.922496
1610	Housework	0	11	146.1	45.2	2.117571	1.35888
1512	Housework	0	11	142.8	36.9	1.809547	1.207428
1503	Housework	0	11	139.2	31.5	1.625669	1.097767

1541	Housework	0	11	148.7	43.5	1.967285	1.340491
1537	Housework	0	11	146.4	82.1	3.830549	1.874712
1540	Housework	0	11	163.5	63.6	2.379149	1.707332
1535	Housework	0	11	148.9	51	2.30028	1.460989
1577	Housework	1	11	143.9	35.9	1.733696	1.193343
1508	Housework	1	11	166.6	42.6	1.534828	1.386592
1509	Housework	1	11	143.3	33.3	1.62163	1.14416
1576	Housework	0	12	164.9	78.9	2.901588	1.923688
1561	Housework	0	12	164.8	58.4	2.150297	1.635908
1548	Housework	0	12	161.2	80.1	3.082495	1.922
1536	Housework	0	12	153.7	49.5	2.095354	1.455917
1598	Housework	0	12	161.7	59	2.256483	1.63259
1547	Housework	0	12	156.1	55	2.257132	1.550288
1511	Housework	0	12	163.4	72.1	2.700419	1.826043
1552	Housework	0	12	154.6	55.4	2.317879	1.550395
1578	Housework	0	12	164.7	88.6	3.266228	2.04648
1564	Housework	0	12	160.9	80.2	3.097864	1.92187
1614	Housework	0	12	170.7	78.1	2.680304	1.93957
1506	Housework	1	12	161.2	51.2	1.970334	1.510863
1550	Housework	1	12	159.2	41.4	1.633481	1.341076
1546	Housework	1	12	145.4	32.7	1.546746	1.139579
1527	Housework	1	12	158.2	86.3	3.448243	1.985786
1524	Housework	1	12	154.8	82.7	3.451148	1.924155
1502	Housework	1	12	152.6	47.2	2.026903	1.415101
1574	Housework	1	12	163.4	85.2	3.191064	1.997578
1604	Housework	1	12	154.5	47.9	2.006682	1.433362
1525	Housework	1	12	154.8	48.9	2.040643	1.450494
1572	Housework	0	13	157.8	63.7	2.558147	1.684908
1599	Housework	0	13	166.2	50.2	1.817363	1.513132
1566	Housework	0	13	169.8	62.1	2.153854	1.711009
1521	Housework	0	13	160.7	45.8	1.77351	1.421211
1556	Housework	0	13	156.8	48.3	1.964513	1.448246
1544	Housework	1	13	162.2	67	2.546673	1.75028
1580	Housework	1	13	128	44.5	2.716064	1.278693
1569	Housework	1	13	151.6	44.7	1.944953	1.370708
1545	Housework	1	13	155.1	61.3	2.54822	1.639212
1605	Housework	0	14	174.6	56.2	1.84352	1.639588
2222	Run 4.5mph	0	7	120.6	20.3	13.9573	0.818838
2224	Run 4.5mph	0	7	127.8	25.1	15.36781	0.93919
2287	Run 4.5mph	0	9	139.6	34.1	17.4978	1.146907
2231	Run 4.5mph	0	7	121.3	21.6	14.68021	0.848578
2272	Run 4.5mph	0	8	133.5	30.2	16.94511	1.055526
2306	Run 4.5mph	0	8	130.3	35	20.6148	1.131733
2221	Run 4.5mph	0	8	140.7	35.2	17.78093	1.170293
2225	Run 4.5mph	1	9	139.5	30	15.41604	1.07025
2245	Run 4.5mph	1	9	140	35.7	18.21429	1.176875
2280	Run 4.5mph	0	11	146.2	40.4	18.90108	1.279609

2256	Run 4.5mph	0	12	163.4	86.9	32.54735	2.018916
2284	Run 4.5mph	0	10	151.1	50.7	22.20644	1.464853
2215	Run 4.5mph	0	12	156.5	59.9	24.45672	1.624748
2247	Run 4.5mph	0	10	140.1	46.69	23.78743	1.359994
2307	Run 4.5mph	0	10	137.3	42.9	22.75706	1.289105
2316	Run 4.5mph	0	10	144.6	49	23.43471	1.413378
2223	Run 4.5mph	1	10	135.8	31.3	16.97246	1.083342
2270	Run 4.5mph	1	11	153.1	40.4	17.23578	1.303216
2211	Run 4.5mph	1	12	155.9	43.7	17.97998	1.369227
2230	Run 4.5mph	1	10	147.9	53.2	24.32066	1.490565
2244	Run 4.5mph	1	11	148.8	66.9	30.21484	1.690107
2300	Run 4.5mph	0	15	182.3	103.5	31.14347	2.31628
2274	Run 4.5mph	0	13	140.6	35.6	18.00858	1.177095
2308	Run 4.5mph	0	13	159.3	84.3	33.21972	1.966295
2309	Run 4.5mph	0	15	164.4	102	37.73954	2.205916
2323	Run 4.5mph	0	13	161.3	96.5	37.09015	2.125031
2288	Run 4.5mph	1	13	159.8	80	31.32827	1.914079
2252	Run 4.5mph	1	14	171.9	72.3	24.46733	1.865899
2273	Run 4.5mph	1	14	147.9	46	21.02914	1.378437
2292	Run 4.5mph	1	15	170.2	91.3	31.51749	2.107045
2556	Run 4.5mph	0	17	172.7	74.2	24.87822	1.895598
2303	Run 4.5mph	1	18	153.9	49.1	20.73024	1.450325
2313	Run 4.5mph	1	17	163.2	81.9	30.74989	1.954639
2314	Run 4.5mph	1	18	161.5	86.6	33.20266	2.005843
3042	Run 4.5mph	0	6	120.9	23.3	15.94055	0.88271
3078	Run 4.5mph	0	6	114.35	19.5	14.91291	0.784595
3079	Run 4.5mph	0	5	114.5	21.033	16.04317	0.817612
3100	Run 4.5mph	0	9	128.45	26.925	16.31877	0.977283
3108	Run 4.5mph	0	7	123	27.1	17.91262	0.963983
3132	Run 4.5mph	0	9	135.267	32.95	18.00826	1.111955
3135	Run 4.5mph	0	7	120.45	23.475	16.1805	0.88496
4042	Run 4.5mph	0	9	132.967	31.65	17.90136	1.08077
4062	Run 4.5mph	0	9	138.367	39.617	20.69267	1.238872
4084	Run 4.5mph	0	8	136.667	27.417	14.67887	1.011404
4097	Run 4.5mph	0	8	138.033	33.75	17.71364	1.135469
3001	Run 4.5mph	1	8	125.5	24.05	15.2696	0.911269
3033	Run 4.5mph	1	8	125.3	24.7	15.73239	0.923848
4003	Run 4.5mph	1	8	128.367	21.517	13.05795	0.866047
4017	Run 4.5mph	1	9	140.5	46.083	23.34469	1.351984
4027	Run 4.5mph	1	9	139.867	41.717	21.32468	1.279223
4047	Run 4.5mph	1	6	127.633	24.3	14.91696	0.922493
4078	Run 4.5mph	1	7	126.8	25.967	16.1504	0.953527
3038	Run 4.5mph	0	11	143.3	34.6	16.84937	1.167969
3113	Run 4.5mph	0	12	157.05	62.475	25.32972	1.664258
4031	Run 4.5mph	0	12	154.733	52.733	22.02503	1.510313
4039	Run 4.5mph	0	12	155.967	40.733	16.74484	1.318644
4041	Run 4.5mph	0	10	135.467	38.3	20.87045	1.206378

4052	Run 4.5mph	0	10	140.867	42.75	21.54357	1.299828
4069	Run 4.5mph	0	10	133.95	26.45	14.74145	0.984194
4108	Run 4.5mph	0	12	135.633	28.783	15.64607	1.035079
3000	Run 4.5mph	1	11	151	52.275	22.92663	1.488762
3060	Run 4.5mph	1	11	157.75	63.875	25.66801	1.687183
3065	Run 4.5mph	1	12	152.95	33.95	14.51245	1.186373
4040	Run 4.5mph	1	10	150.667	59.333	26.13726	1.592302
4048	Run 4.5mph	1	10	132.8	24.033	13.62736	0.931568
4106	Run 4.5mph	1	12	157.067	46.983	19.04456	1.427835
4110	Run 4.5mph	1	12	152.467	41.917	18.03178	1.327128
3055	Run 4.5mph	0	14	184.6	71.3	20.92311	1.905049
3096	Run 4.5mph	0	14	170.567	106.5	36.60662	2.290931
4012	Run 4.5mph	0	13	156.467	62.25	25.42693	1.658585
3016	Run 4.5mph	1	15	159.4	61.633	24.25698	1.661913
4022	Run 4.5mph	1	13	158.967	49.317	19.51564	1.472557
4051	Run 4.5mph	1	14	158.967	58.9	23.3078	1.620117
4011	Run 4.5mph	1	16	163.633	62.1	23.19262	1.6861
4058	Run 4.5mph	1	16	165.733	68.533	24.95064	1.786903
1316	Run 4.5mph	0	9	142.7	38.4	18.85746	1.233239
1547	Run 4.5mph	0	12	156.1	55	22.57132	1.550288
1541	Run 4.5mph	0	11	148.7	43.5	19.67285	1.340491
1552	Run 4.5mph	0	12	154.6	55.4	23.17879	1.550395
1573	Run 4.5mph	0	12	158.1	48.4	19.3634	1.454611
1319	Run 4.5mph	1	11	140.1	29.2	14.8767	1.056602
1328	Run 4.5mph	1	11	145.5	32	15.11555	1.126701
1124	Run 4.5mph	1	11	149	50	22.52151	1.445897
1324	Run 4.5mph	1	11	158.4	48.2	19.21041	1.452466
1558	Run 4.5mph	1	12	153.9	60.3	25.45893	1.619781
1591	Run 4.5mph	1	12	153.7	63.6	26.92213	1.666008
1509	Run 4.5mph	1	11	143.3	33.3	16.2163	1.14416
1288	Run 4.5mph	0	13	164.5	80.5	29.74843	1.942699
1168	Run 4.5mph	0	13	172	83.9	28.35992	2.021822
1198	Run 4.5mph	0	13	152.5	44.2	19.00564	1.365641
1566	Run 4.5mph	0	13	169.8	62.1	21.53854	1.711009
1605	Run 4.5mph	0	14	174.6	56.2	18.4352	1.639588
1521	Run 4.5mph	0	13	160.7	45.8	17.7351	1.421211
1545	Run 4.5mph	1	13	155.1	61.3	25.4822	1.639212
1570	Run 4.5mph	1	13	162.4	47.4	17.97241	1.453752
201001	Run 4.5mph	0	8	136.8	31.2	16.6718	1.084629
201019	Run 4.5mph	0	5	110	19.3	15.95041	0.768354
201029	Run 4.5mph	0	7	125.9667	26.7	16.82674	0.965382
201031	Run 4.5mph	0	5	109	17	14.30856	0.715075
201035	Run 4.5mph	0	8	135.5	34	18.51827	1.131645
201037	Run 4.5mph	0	7	112.5	16.7	13.19506	0.71719
201038	Run 4.5mph	0	7	128.8	35.1	21.15804	1.128281
201039	Run 4.5mph	0	6	125.2	28.1	17.92659	0.989885
201043	Run 4.5mph	0	7	125.65	26.6	16.84832	0.962475

201049	Run 4.5mph	0	7	121.6	20.6	13.93157	0.828029
201002	Run 4.5mph	1	6	121.8	20.3	13.68363	0.822058
201005	Run 4.5mph	1	7	119	22.3	15.74748	0.856733
201006	Run 4.5mph	1	8	134.5	28.6	15.80962	1.02811
201012	Run 4.5mph	1	7	122.7	22.8	15.14418	0.877598
201017	Run 4.5mph	1	7	129	25.5	15.3236	0.950726
201018	Run 4.5mph	1	7	130.15	27.7	16.35277	0.997497
201020	Run 4.5mph	1	7	122	21.2	14.24348	0.842009
201021	Run 4.5mph	1	7	130.2	24.1	14.21658	0.92568
201023	Run 4.5mph	1	6	118.5	21.2	15.0973	0.832349
201024	Run 4.5mph	1	8	133.45	30.9	17.35087	1.068455
201025	Run 4.5mph	1	6	123.85	28	18.25433	0.983753
201026	Run 4.5mph	1	7	118.05	26.2	18.8005	0.931343
201030	Run 4.5mph	1	6	122.7667	27.8	18.44522	0.97656
201041	Run 4.5mph	1	8	126.95	23.4	14.51946	0.902037
201044	Run 4.5mph	1	8	128.8	26.3	15.85346	0.966058
201046	Run 4.5mph	1	7	127.6	23.2	14.24907	0.899703
201051	Run 4.5mph	1	8	145	36	17.12247	1.198743
201052	Run 4.5mph	1	8	129.85	27.7	16.42842	0.996585
201054	Run 4.5mph	1	5	108.3	19.4	16.54035	0.76575
2204	Run 5mph	0	9	134.6	39.4	21.74734	1.221776
2233	Run 5mph	0	9	138.1	30	15.73019	1.06598
2213	Run 5mph	0	11	147.6	46.9	21.52782	1.391755
2216	Run 5mph	0	10	151.7	37.8	16.42558	1.252847
2229	Run 5mph	0	11	139.4	29.6	15.23232	1.06225
2203	Run 5mph	0	12	147.2	46	21.22962	1.375847
2243	Run 5mph	0	10	148.3	43.6	19.82459	1.340715
2518	Run 5mph	0	12	159.2	52.8	20.83281	1.528492
2207	Run 5mph	1	11	149.3	39.8	17.85515	1.279955
2217	Run 5mph	1	12	159.1	54.6	21.57011	1.55591
2271	Run 5mph	1	10	144.3	40.8	19.5942	1.279754
2511	Run 5mph	1	15	161.8	62.4	23.83568	1.682944
2219	Run 5mph	0	15	174.9	61	19.94115	1.714638
2227	Run 5mph	0	13	145	35.7	16.97979	1.19336
2512	Run 5mph	0	13	175.2	52.2	17.006	1.577903
2540	Run 5mph	0	15	169.4	92.8	32.3386	2.121626
2254	Run 5mph	0	13	154.7	41.8	17.46609	1.332793
2262	Run 5mph	0	15	175.1	61.7	20.12393	1.725974
2263	Run 5mph	0	13	144.3	34.3	16.47257	1.165721
2202	Run 5mph	0	13	148.9	53	23.90487	1.491528
2293	Run 5mph	0	14	178.2	89	28.02687	2.116518
2205	Run 5mph	1	15	156.9	52.2	21.20433	1.510388
2206	Run 5mph	1	15	162.5	51.7	19.5787	1.523624
2214	Run 5mph	1	14	163.2	59.8	22.4523	1.650488
2501	Run 5mph	0	17	188.2	76.6	21.62666	1.99516
2505	Run 5mph	0	16	178.2	70.6	22.23255	1.868647
2317	Run 5mph	0	18	168.4	86.6	30.53752	2.039386

2318	Run 5mph	0	17	173.4	112.8	37.51552	2.378325
2226	Run 5mph	0	17	177.2	65.6	20.89183	1.792265
2234	Run 5mph	1	17	162.2	46.8	17.7887	1.443121
2305	Run 5mph	1	17	166.5	90.9	32.78955	2.083841
2210	Run 5mph	1	17	158.2	50.4	20.13806	1.487007
2249	Run 5mph	1	17	160.2	74.4	28.98998	1.84264
2311	Run 5mph	1	16	166.7	80.9	29.11235	1.95817
3091	Run 5mph	0	9	140.45	36.875	18.69341	1.199075
3101	Run 5mph	0	8	137	36.075	19.22052	1.173389
3103	Run 5mph	0	6	129.5	27.225	16.23411	0.986302
3104	Run 5mph	0	8	132.167	29.467	16.86902	1.037534
4021	Run 5mph	0	8	132.667	26.583	15.10352	0.983095
4061	Run 5mph	0	8	135.933	31.55	17.07456	1.088409
4030	Run 5mph	1	8	131.733	31.583	18.1997	1.075557
4034	Run 5mph	1	8	131.233	31.333	18.19348	1.069356
4064	Run 5mph	1	9	133.233	39.933	22.49616	1.225668
4075	Run 5mph	1	6	130.567	28.617	16.78639	1.01641
4092	Run 5mph	1	9	153	61.65	26.33602	1.635377
3049	Run 5mph	0	7	122.35	23.2	15.49816	0.884842
3111	Run 5mph	0	9	136.5	34.875	18.71755	1.150565
4005	Run 5mph	0	9	134.233	31.9	17.70403	1.089438
4029	Run 5mph	0	7	128.3	27.05	16.43289	0.979267
3048	Run 5mph	1	9	134.367	32.483	17.99165	1.100536
3061	Run 5mph	1	9	138.167	34.2	17.91503	1.144026
3066	Run 5mph	1	8	132.05	28.975	16.61677	1.02782
4020	Run 5mph	1	9	137.2	30.8	16.36223	1.078376
4104	Run 5mph	1	9	133.133	32.633	18.41135	1.099238
3022	Run 5mph	0	10	144.8	32.875	15.67937	1.140983
4037	Run 5mph	0	11	138.033	33.6	17.63492	1.132752
3114	Run 5mph	1	10	140.55	27.5	13.92102	1.024362
3123	Run 5mph	1	10	145.45	33.125	15.65772	1.147677
4070	Run 5mph	1	10	138.5	38.483	20.06178	1.220137
4081	Run 5mph	1	10	142.733	35.6	17.47436	1.184141
4083	Run 5mph	1	11	164.633	61.1	22.54278	1.675485
4101	Run 5mph	1	11	137.633	28.2	14.88689	1.029708
4109	Run 5mph	1	12	157.067	51.433	20.84836	1.499044
3034	Run 5mph	0	11	142.8	42.525	20.85393	1.303165
3095	Run 5mph	0	12	160.9	78.967	30.50237	1.905923
3102	Run 5mph	0	11	149.6	38.05	17.00167	1.250368
3125	Run 5mph	0	10	153.5	41.817	17.74746	1.328976
3128	Run 5mph	0	11	145.067	35.917	17.06722	1.197475
4008	Run 5mph	0	12	135.9	27.967	15.14283	1.019987
4010	Run 5mph	0	11	144.567	48.3	23.11047	1.402357
3009	Run 5mph	1	11	164.5	58.367	21.56928	1.63423
3041	Run 5mph	1	10	142.45	36.583	18.02828	1.20067
3120	Run 5mph	1	11	157.667	43.3	17.41832	1.368573
4076	Run 5mph	1	12	156.433	49.467	20.21431	1.465598

4079	Run 5mph	1	12	152.133	59.8	25.83773	1.605179
3020	Run 5mph	0	13	147.5	33.7	15.4898	1.164793
4057	Run 5mph	0	13	158.267	47.45	18.94329	1.439788
3112	Run 5mph	1	14	160.3	69.25	26.94963	1.773347
4015	Run 5mph	1	14	154.667	45.9	19.18745	1.401459
4094	Run 5mph	1	13	167.167	79.983	28.62178	1.948361
4036	Run 5mph	1	14	157	72.7	29.4941	1.805378
1511	Run 5mph	0	12	163.4	72.1	27.00419	1.826043
1539	Run 5mph	0	12	172.6	69.7	23.39652	1.832457
1540	Run 5mph	0	11	163.5	63.6	23.79149	1.707332
1321	Run 5mph	1	12	57.3	50.9	155.0274	0.999506
1604	Run 5mph	1	12	154.5	47.9	20.06682	1.433362
1508	Run 5mph	1	11	166.6	42.6	15.34828	1.386592
1546	Run 5mph	1	12	145.4	32.7	15.46746	1.139579
1593	Run 5mph	1	12	158.2	61.8	24.69309	1.659355
1535	Run 5mph	0	11	148.9	51	23.0028	1.460989
1507	Run 5mph	0	12	162.3	52.3	19.85476	1.53236
1536	Run 5mph	0	12	153.7	49.5	20.95354	1.455917
1568	Run 5mph	1	12	165.6	52.5	19.14426	1.547809
1228	Run 5mph	0	13	148.8	49.1	22.17561	1.431079
1572	Run 5mph	0	13	157.8	63.7	25.58147	1.684908
1600	Run 5mph	0	13	187.5	80.2	22.81244	2.042034
100339	Run 5mph	0	9	133	28.5	16.11171	1.021623
100374	Run 5mph	0	9	133	32.4	18.31647	1.094577
100505	Run 5mph	0	9	143.3	26.9	13.09966	1.020087
100601	Run 5mph	0	8	142.2	33.6	16.61652	1.146186
100614	Run 5mph	0	9	139.4	36.8	18.93748	1.194205
100769	Run 5mph	0	8	127.8	29.1	17.81686	1.016929
100816	Run 5mph	0	9	129.3	26.1	15.61146	0.963579
100923	Run 5mph	0	9	132.4	28.3	16.14397	1.015939
100974	Run 5mph	0	8	120.2	23.2	16.05754	0.878646
100989	Run 5mph	0	8	130.4	29.3	17.23108	1.028863
101140	Run 5mph	0	9	137.1	34.6	18.40777	1.14767
101230	Run 5mph	0	9	131.4	27.1	15.69562	0.989561
101240	Run 5mph	0	8	132.2	30.7	17.5661	1.060766
101419	Run 5mph	0	8	133.4	29.8	16.74575	1.047673
111777	Run 5mph	0	8	125.1	25.7	16.42171	0.943181
112110	Run 5mph	0	9	133.2	38.7	21.81235	1.205049
112125	Run 5mph	0	8	125	23.6	15.104	0.900632
112198	Run 5mph	0	8	127.2	26.5	16.37841	0.965207
112335	Run 5mph	0	9	148.8	48.9	22.08528	1.427941
112772	Run 5mph	0	9	136.5	34.4	18.46261	1.14211
112895	Run 5mph	0	9	143.4	34.4	16.72862	1.164656
113182	Run 5mph	0	9	124.7	26.2	16.84878	0.951797
113347	Run 5mph	0	9	139.2	28.8	14.86326	1.046117
113527	Run 5mph	0	8	142.5	42.1	20.73253	1.295065
113546	Run 5mph	0	8	134.6	31	17.11085	1.07396

100234	Run 5mph	1	9	133.3	29.6	16.65833	1.043575
100576	Run 5mph	1	9	128.1	27.6	16.81941	0.989313
100589	Run 5mph	1	9	127.2	27.6	17.05827	0.986552
100841	Run 5mph	1	8	134.7	27.3	15.04622	1.003298
100912	Run 5mph	1	8	127.1	23.4	14.48521	0.90246
100953	Run 5mph	1	9	155.5	38.6	15.96344	1.279525
101104	Run 5mph	1	9	133.4	27.6	15.50949	1.00534
101300	Run 5mph	1	8	135.7	35.1	19.06108	1.151864
101373	Run 5mph	1	9	134.2	32	17.76826	1.091167
111581	Run 5mph	1	8	127.9	27.6	16.87206	0.9887
111665	Run 5mph	1	8	132	28.3	16.24197	1.014721
111703	Run 5mph	1	8	129.9	34.3	20.32712	1.11814
111797	Run 5mph	1	8	140.3	35.1	17.83166	1.167186
111901	Run 5mph	1	8	124.5	23.7	15.29008	0.90125
111932	Run 5mph	1	8	130	27.2	16.09467	0.987322
112484	Run 5mph	1	8	139	37.7	19.51245	1.208447
112564	Run 5mph	1	9	131.5	28.7	16.59703	1.020872
112780	Run 5mph	1	9	142.2	35.4	17.50669	1.17881
113034	Run 5mph	1	9	141.2	30.4	15.24769	1.083089
113147	Run 5mph	1	9	130	35	20.71006	1.1307
113199	Run 5mph	1	9	137.9	29.2	15.35516	1.049993
113212	Run 5mph	1	9	129.6	26.3	15.65834	0.968432
113274	Run 5mph	1	9	137	31.5	16.78299	1.090857
100042	Run 5mph	0	10	143	49	23.96205	1.407158
100131	Run 5mph	0	12	165.4	53.1	19.40991	1.556552
100160	Run 5mph	0	10	149.4	48.5	21.72904	1.423917
100342	Run 5mph	0	12	153	45	19.22338	1.380669
100356	Run 5mph	0	10	139.2	33.9	17.49529	1.141985
100438	Run 5mph	0	11	144.4	42.4	20.33441	1.306863
100518	Run 5mph	0	12	140.3	35.6	18.08567	1.176099
100529	Run 5mph	0	12	155.8	46.6	19.19778	1.417007
100594	Run 5mph	0	11	150.6	57.1	25.17597	1.559513
100787	Run 5mph	0	10	145	36.3	17.26516	1.204105
100791	Run 5mph	0	11	142.2	36.2	17.90232	1.193062
100804	Run 5mph	0	10	129.5	26.4	15.74216	0.970114
100861	Run 5mph	0	10	142.2	42.2	20.86956	1.295635
100890	Run 5mph	0	11	146.9	37.4	17.33118	1.229923
100990	Run 5mph	0	11	148.6	40.2	18.20491	1.284462
101082	Run 5mph	0	10	146.5	30.5	14.211	1.100968
101112	Run 5mph	0	11	141.4	32.2	16.10486	1.117747
101150	Run 5mph	0	10	127.1	33.1	20.48976	1.087495
101173	Run 5mph	0	12	172	53.1	17.94889	1.580883
101220	Run 5mph	0	11	142.8	30.9	15.15312	1.097525
101265	Run 5mph	0	10	133	28.7	16.22477	1.025472
101428	Run 5mph	0	10	137.2	29.4	15.61849	1.051731
101459	Run 5mph	0	10	140.9	35.5	17.8816	1.176309
101546	Run 5mph	0	11	146.4	41.6	19.40936	1.300616

111564	Run 5mph	0	10	140.6	31	15.68162	1.092688
111621	Run 5mph	0	10	137.9	32	16.82757	1.102994
111683	Run 5mph	0	10	142.6	35.2	17.31026	1.176532
111977	Run 5mph	0	10	145.3	48.7	23.06735	1.411417
112001	Run 5mph	0	11	158.8	44.4	17.60686	1.391105
112062	Run 5mph	0	12	168.4	86.5	30.50225	2.038119
112075	Run 5mph	0	12	152.7	47.1	20.1996	1.413855
112203	Run 5mph	0	11	149.9	39	17.35647	1.268068
112228	Run 5mph	0	11	138.5	34.1	17.77685	1.143316
112425	Run 5mph	0	12	153.8	34.3	14.50045	1.195559
112438	Run 5mph	0	11	154.8	48.1	20.07258	1.437683
112702	Run 5mph	0	12	149	47.8	21.53056	1.411327
112748	Run 5mph	0	12	170.3	46	15.86093	1.457689
112762	Run 5mph	0	12	164.5	54.5	20.14024	1.57508
112845	Run 5mph	0	12	165.2	60.8	22.27837	1.673335
113051	Run 5mph	0	12	161	42.6	16.43455	1.367926
100021	Run 5mph	1	11	144.9	32.5	15.47913	1.134276
100126	Run 5mph	1	12	153.8	39	16.48739	1.281045
100179	Run 5mph	1	12	169.1	61.3	21.43746	1.696339
100186	Run 5mph	1	10	142	32.6	16.16743	1.127083
100258	Run 5mph	1	10	151.6	59.3	25.80217	1.595726
100265	Run 5mph	1	11	146.9	34.4	15.94097	1.175842
100298	Run 5mph	1	12	166.4	59.3	21.41648	1.655748
100360	Run 5mph	1	10	137.8	41.9	22.06559	1.274692
100417	Run 5mph	1	11	155.3	49	20.3167	1.453945
100445	Run 5mph	1	10	146.2	42.4	19.83678	1.313297
100459	Run 5mph	1	12	162.5	54.4	20.60118	1.565913
100649	Run 5mph	1	11	149	41.5	18.69285	1.30803
100672	Run 5mph	1	12	162.2	53.9	20.48741	1.557015
100851	Run 5mph	1	10	149.1	43.2	19.43249	1.336934
100882	Run 5mph	1	12	157.4	45.6	18.40583	1.406256
100946	Run 5mph	1	10	140.5	37	18.74343	1.201429
101012	Run 5mph	1	11	147.7	58	26.58686	1.56061
101040	Run 5mph	1	11	146.9	35.4	16.40437	1.194103
101060	Run 5mph	1	11	164.8	38.7	14.2494	1.311152
101209	Run 5mph	1	12	156.7	54.2	22.073	1.54046
101256	Run 5mph	1	12	154.1	45.9	19.32891	1.39942
101331	Run 5mph	1	10	150.9	41.3	18.13726	1.311205
111570	Run 5mph	1	11	145.7	41.2	19.40788	1.29142
111636	Run 5mph	1	11	157	45	18.25632	1.394866
111785	Run 5mph	1	11	153.5	83.1	35.26828	1.922716
111911	Run 5mph	1	10	143	33.6	16.43112	1.148737
111948	Run 5mph	1	11	139	45	23.29072	1.329135
111950	Run 5mph	1	10	143.6	41.6	20.17365	1.290698
112085	Run 5mph	1	12	142.2	39.2	19.38594	1.245257
112399	Run 5mph	1	12	155.8	54.7	22.53473	1.544556
112411	Run 5mph	1	12	166	60.7	22.02787	1.675059

112576	Run 5mph	1	10	148.5	38.9	17.63992	1.261617
112656	Run 5mph	1	12	152	31.2	13.50416	1.130888
112852	Run 5mph	1	11	149.7	43.2	19.27703	1.339064
100071	Run 5mph	0	13	160	43.1	16.83594	1.373142
100084	Run 5mph	0	15	179.5	63.5	19.7081	1.770203
100208	Run 5mph	0	15	175.1	57.9	18.88453	1.667967
100302	Run 5mph	0	14	163.6	53.1	19.83937	1.549815
100404	Run 5mph	0	13	172.2	47.2	15.91753	1.484533
100567	Run 5mph	0	14	144.5	35.5	17.00171	1.188132
100756	Run 5mph	0	13	170.1	58.6	20.25298	1.659613
100874	Run 5mph	0	13	170.4	52.7	18.14978	1.568645
100901	Run 5mph	0	13	173.1	41.8	13.95025	1.393509
100932	Run 5mph	0	13	151.8	42.4	18.40019	1.333011
101124	Run 5mph	0	14	166	48.2	17.49165	1.479701
101193	Run 5mph	0	14	178.9	60.3	18.84068	1.719371
101313	Run 5mph	0	13	151.3	35.9	15.68255	1.217302
101329	Run 5mph	0	13	165.5	48.3	17.63401	1.47958
101366	Run 5mph	0	14	156.5	43	17.55657	1.359456
101444	Run 5mph	0	14	165.3	53.7	19.65299	1.565611
101461	Run 5mph	0	13	153.9	40.2	16.97262	1.30243
101474	Run 5mph	0	15	172.2	69.1	23.303	1.82228
101550	Run 5mph	0	13	170.9	52.1	17.83831	1.560826
111654	Run 5mph	0	13	171	68.5	23.42601	1.808733
111710	Run 5mph	0	13	155.9	42.7	17.56854	1.352286
111820	Run 5mph	0	13	167.5	59.1	21.06483	1.657065
111849	Run 5mph	0	13	154	35.8	15.09529	1.224029
111850	Run 5mph	0	15	157.5	50.3	20.27715	1.48281
111870	Run 5mph	0	14	173	68.6	22.92091	1.818515
111883	Run 5mph	0	14	180	61.7	19.04321	1.744961
111991	Run 5mph	0	14	158.8	46.9	18.59824	1.432696
112015	Run 5mph	0	14	171.9	57	19.28959	1.641924
112023	Run 5mph	0	14	163.4	50.5	18.91417	1.507799
112263	Run 5mph	0	13	165.5	51.3	18.72929	1.528315
112359	Run 5mph	0	14	173.7	83.7	27.74124	2.027116
112379	Run 5mph	0	15	184.7	70.8	20.75389	1.89826
112666	Run 5mph	0	15	167.4	54.8	19.55553	1.590718
112674	Run 5mph	0	15	177.4	87.8	27.89891	2.097379
112722	Run 5mph	0	15	179.5	64.1	19.89432	1.779179
112739	Run 5mph	0	15	174.1	57.2	18.87116	1.653334
112820	Run 5mph	0	15	163	55.3	20.81373	1.581718
112838	Run 5mph	0	15	179	67.3	21.00434	1.82439
112944	Run 5mph	0	15	170.8	62.5	21.42418	1.720928
112952	Run 5mph	0	15	173.4	55.4	18.42517	1.622553
100140	Run 5mph	1	15	166.1	80	28.99684	1.943644
100652	Run 5mph	1	13	167.4	70.1	25.01538	1.815951
100669	Run 5mph	1	13	164.2	69.4	25.74027	1.79241
100683	Run 5mph	1	14	152.5	46.2	19.86563	1.398534

100703	Run 5mph	1	13	173.4	63.1	20.98607	1.740184
100719	Run 5mph	1	15	172.6	71.7	24.06787	1.860551
100774	Run 5mph	1	15	154.7	53	22.146	1.514293
101219	Run 5mph	1	13	152.4	44.5	19.15976	1.370262
111679	Run 5mph	1	14	161	47.7	18.40207	1.453695
111819	Run 5mph	1	15	165.6	62.5	22.79079	1.699965
111985	Run 5mph	1	14	154.3	40.5	17.01074	1.308995
112048	Run 5mph	1	15	162.6	65	24.58512	1.723667
112052	Run 5mph	1	15	160.4	92.8	36.06943	2.076207
112102	Run 5mph	1	13	167.8	61.8	21.94849	1.698562
112162	Run 5mph	1	13	153.6	56.4	23.90544	1.561362
112173	Run 5mph	1	13	159.4	52	20.4657	1.516747
112189	Run 5mph	1	13	166.6	55.4	19.95996	1.597025
112212	Run 5mph	1	14	159.1	49.2	19.4368	1.471164
112237	Run 5mph	1	13	152.5	41.3	17.75867	1.316699
112304	Run 5mph	1	14	175.8	108.9	35.23629	2.346494
112401	Run 5mph	1	15	158.7	52.2	20.72606	1.517233
112457	Run 5mph	1	14	166.9	59.1	21.21655	1.654709
112517	Run 5mph	1	15	161.1	44.1	16.99212	1.393966
112534	Run 5mph	1	13	163.3	79.8	29.92475	1.927992
112630	Run 5mph	1	15	163.6	88.9	33.21507	2.044765
112643	Run 5mph	1	15	168.8	55.2	19.37288	1.602233
112680	Run 5mph	1	13	148.3	39.1	17.77847	1.264426
112714	Run 5mph	1	15	174.4	60.5	19.89127	1.705129
112867	Run 5mph	1	14	151.4	50.9	22.20578	1.469112
112871	Run 5mph	1	13	163	48.4	18.21672	1.472317
112884	Run 5mph	1	14	162.6	50.6	19.13857	1.50647
113040	Run 5mph	1	15	158.8	73.7	29.22581	1.826928
113163	Run 5mph	1	14	156.5	56.9	23.23184	1.580466
113267	Run 5mph	1	15	161.2	53.5	20.58845	1.546993
100016	Run 5mph	0	17	174.4	58.3	19.16795	1.671498
100221	Run 5mph	0	16	177	69.5	22.18392	1.847976
100482	Run 5mph	0	16	179.2	65.5	20.39695	1.79878
100724	Run 5mph	0	17	178.3	72.6	22.83673	1.897354
100961	Run 5mph	0	16	174.5	70.8	23.25104	1.855991
101059	Run 5mph	0	18	181.8	87	26.32276	2.107447
101133	Run 5mph	0	16	170	53.1	18.3737	1.57357
101507	Run 5mph	0	16	173.9	76.1	25.16434	1.926831
101534	Run 5mph	0	18	167.7	53.1	18.88115	1.565096
111642	Run 5mph	0	18	184	86.4	25.51985	2.109654
111721	Run 5mph	0	18	176.6	61.4	19.68734	1.727286
111802	Run 5mph	0	17	181.1	71.5	21.80063	1.893498
112251	Run 5mph	0	16	173.4	57.2	19.02383	1.650695
112299	Run 5mph	0	18	164.1	61	22.65232	1.671859
112346	Run 5mph	0	17	173.3	85.6	28.50209	2.049861
112463	Run 5mph	0	16	167.3	62.1	22.18706	1.700978
112476	Run 5mph	0	17	172.6	69.5	23.32938	1.829628

112618	Run 5mph	0	16	173.6	65.6	21.76729	1.777742
112621	Run 5mph	0	16	179.7	80.1	24.80484	2.006581
112691	Run 5mph	0	17	171.8	64.1	21.71759	1.748525
112753	Run 5mph	0	16	175.3	75.1	24.43859	1.919264
112790	Run 5mph	0	16	176.6	76.6	24.56107	1.945475
112964	Run 5mph	0	18	178.7	71.6	22.42146	1.884928
113017	Run 5mph	0	17	170.5	72.7	25.00838	1.865387
113023	Run 5mph	0	17	172.8	64.3	21.53394	1.755491
113081	Run 5mph	0	17	173	82.2	27.465	2.004287
113095	Run 5mph	0	17	183.4	94.3	28.0358	2.208433
113115	Run 5mph	0	16	177.3	90.1	28.66205	2.126275
113157	Run 5mph	0	18	170.4	62.5	21.52488	1.719329
113297	Run 5mph	0	18	169.5	54.9	19.10878	1.600166
113305	Run 5mph	0	18	183.5	72	21.38259	1.910553
113338	Run 5mph	0	18	182.5	67.8	20.35654	1.845781
113353	Run 5mph	0	18	163.5	57.7	21.58442	1.620239
113375	Run 5mph	0	18	168.7	88.4	31.06148	2.06353
113380	Run 5mph	0	18	179.8	69.4	21.46743	1.85807
100117	Run 5mph	1	18	171.8	70.1	23.75044	1.834724
100385	Run 5mph	1	17	164.7	59.9	22.08206	1.657974
100549	Run 5mph	1	18	164.9	52.6	19.34392	1.546795
100836	Run 5mph	1	16	174.3	64.3	21.1649	1.761515
101181	Run 5mph	1	16	161.8	50.9	19.44289	1.508316
111745	Run 5mph	1	18	164.6	62	22.884	1.688579
111860	Run 5mph	1	16	166	60.2	21.84642	1.667624
111928	Run 5mph	1	16	159	56	22.15102	1.576847
112318	Run 5mph	1	16	164.1	48.4	17.97332	1.476247
112442	Run 5mph	1	16	167	59	21.15529	1.653596
112498	Run 5mph	1	18	159.5	62.1	24.41014	1.669088
112503	Run 5mph	1	18	158.2	58.6	23.41449	1.612579
112972	Run 5mph	1	17	168.1	48.4	17.12813	1.490408
112987	Run 5mph	1	17	163.6	51.4	19.20421	1.52293
112993	Run 5mph	1	17	171.5	69.2	23.52761	1.820756
113000	Run 5mph	1	16	160.2	99.5	38.7702	2.154457
113067	Run 5mph	1	16	159.8	72	28.19544	1.808638
113079	Run 5mph	1	17	162.9	72.8	27.434	1.833328
113103	Run 5mph	1	17	155.7	49.1	20.2537	1.457025
113135	Run 5mph	1	16	165.6	61.1	22.28027	1.679379
113206	Run 5mph	1	16	170.9	67.7	23.17953	1.796925
113227	Run 5mph	1	17	170.9	62.3	21.33064	1.718363
113251	Run 5mph	1	18	163	46.8	17.61451	1.445938
113289	Run 5mph	1	16	172.7	63.7	21.35771	1.746261
113312	Run 5mph	1	18	158.6	55.8	22.18339	1.572245
113412	Run 5mph	1	18	161.5	55.4	21.2405	1.577464
113435	Run 5mph	1	18	168.2	65.2	23.046	1.749847
113455	Run 5mph	1	17	171.5	71.6	24.3436	1.854449
113463	Run 5mph	1	17	159.5	62.2	24.44945	1.670533

113477	Run 5mph	1	18	154.6	51.6	21.58891	1.492265
113494	Run 5mph	1	17	161	56.4	21.75842	1.590757
113517	Run 5mph	1	17	164.1	53.9	20.01574	1.56422
100601	Rope Skip	0	8	142.2	33.6	1.661652	1.146186
100974	Rope Skip	0	8	120.2	23.2	1.605754	0.878646
100989	Rope Skip	0	8	130.4	29.3	1.723108	1.028863
101240	Rope Skip	0	8	132.2	30.7	1.75661	1.060766
101419	Rope Skip	0	8	133.4	29.8	1.674575	1.047673
111695	Rope Skip	0	8	126	22.9	1.442429	0.888969
111777	Rope Skip	0	8	125.1	25.7	1.642171	0.943181
111963	Rope Skip	0	8	131.1	29.4	1.710574	1.03294
112090	Rope Skip	0	8	126.7	23.8	1.482599	0.909587
112125	Rope Skip	0	8	125	23.6	1.5104	0.900632
112198	Rope Skip	0	8	127.2	26.5	1.637841	0.965207
113527	Rope Skip	0	8	142.5	42.1	2.073253	1.295065
113546	Rope Skip	0	8	134.6	31	1.711085	1.07396
100424	Rope Skip	1	8	131.2	24.9	1.446544	0.944942
100912	Rope Skip	1	8	127.1	23.4	1.448521	0.90246
101300	Rope Skip	1	8	135.7	35.1	1.906108	1.151864
111581	Rope Skip	1	8	127.9	27.6	1.687206	0.9887
111665	Rope Skip	1	8	132	28.3	1.624197	1.014721
111703	Rope Skip	1	8	129.9	34.3	2.032712	1.11814
111734	Rope Skip	1	8	132.3	30.9	1.765382	1.064796
111797	Rope Skip	1	8	140.3	35.1	1.783166	1.167186
111901	Rope Skip	1	8	124.5	23.7	1.529008	0.90125
112484	Rope Skip	1	8	139	37.7	1.951245	1.208447
100193	Rope Skip	0	9	135.5	49.1	2.674256	1.378938
100339	Rope Skip	0	9	133	28.5	1.611171	1.021623
100374	Rope Skip	0	9	133	32.4	1.831647	1.094577
100505	Rope Skip	0	9	143.3	26.9	1.309966	1.020087
100614	Rope Skip	0	9	139.4	36.8	1.893748	1.194205
100816	Rope Skip	0	9	129.3	26.1	1.561146	0.963579
100923	Rope Skip	0	9	132.4	28.3	1.614397	1.015939
101140	Rope Skip	0	9	137.1	34.6	1.840777	1.14767
101230	Rope Skip	0	9	131.4	27.1	1.569562	0.989561
112110	Rope Skip	0	9	133.2	38.7	2.181235	1.205049
112335	Rope Skip	0	9	148.8	48.9	2.208528	1.427941
112772	Rope Skip	0	9	136.5	34.4	1.846261	1.14211
112895	Rope Skip	0	9	143.4	34.4	1.672862	1.164656
113182	Rope Skip	0	9	124.7	26.2	1.684878	0.951797
113347	Rope Skip	0	9	139.2	28.8	1.486326	1.046117
100234	Rope Skip	1	9	133.3	29.6	1.665833	1.043575
100576	Rope Skip	1	9	128.1	27.6	1.681941	0.989313
100589	Rope Skip	1	9	127.2	27.6	1.705827	0.986552
100953	Rope Skip	1	9	155.5	38.6	1.596344	1.279525
101071	Rope Skip	1	9	144.2	37	1.77939	1.213872
101104	Rope Skip	1	9	133.4	27.6	1.550949	1.00534

101373	Rope Skip	1	9	134.2	32	1.776826	1.091167
112564	Rope Skip	1	9	131.5	28.7	1.659703	1.020872
112780	Rope Skip	1	9	142.2	35.4	1.750669	1.17881
113034	Rope Skip	1	9	141.2	30.4	1.524769	1.083089
113147	Rope Skip	1	9	130	35	2.071006	1.1307
113199	Rope Skip	1	9	137.9	29.2	1.535516	1.049993
113212	Rope Skip	1	9	129.6	26.3	1.565834	0.968432
113274	Rope Skip	1	9	137	31.5	1.678299	1.090857
100042	Rope Skip	0	10	143	49	2.396205	1.407158
100160	Rope Skip	0	10	149.4	48.5	2.172904	1.423917
100356	Rope Skip	0	10	139.2	33.9	1.749529	1.141985
100471	Rope Skip	0	10	132.8	28.7	1.627368	1.024861
100787	Rope Skip	0	10	145	36.3	1.726516	1.204105
100804	Rope Skip	0	10	129.5	26.4	1.574216	0.970114
100861	Rope Skip	0	10	142.2	42.2	2.086956	1.295635
101082	Rope Skip	0	10	146.5	30.5	1.4211	1.100968
101150	Rope Skip	0	10	127.1	33.1	2.048976	1.087495
101265	Rope Skip	0	10	133	28.7	1.622477	1.025472
101428	Rope Skip	0	10	137.2	29.4	1.561849	1.051731
101459	Rope Skip	0	10	140.9	35.5	1.78816	1.176309
111564	Rope Skip	0	10	140.6	31	1.568162	1.092688
111621	Rope Skip	0	10	137.9	32	1.682757	1.102994
111683	Rope Skip	0	10	142.6	35.2	1.731026	1.176532
111977	Rope Skip	0	10	145.3	48.7	2.306735	1.411417
113530	Rope Skip	0	10	146.5	45.2	2.106023	1.360354
100186	Rope Skip	1	10	142	32.6	1.616743	1.127083
100258	Rope Skip	1	10	151.6	59.3	2.580217	1.595726
100360	Rope Skip	1	10	137.8	41.9	2.206559	1.274692
100397	Rope Skip	1	10	139.8	34	1.739661	1.145747
100445	Rope Skip	1	10	146.2	42.4	1.983678	1.313297
100851	Rope Skip	1	10	149.1	43.2	1.943249	1.336934
100946	Rope Skip	1	10	140.5	37	1.874343	1.201429
101331	Rope Skip	1	10	150.9	41.3	1.813726	1.311205
111839	Rope Skip	1	10	155.5	46	1.902379	1.406091
111911	Rope Skip	1	10	143	33.6	1.643112	1.148737
111950	Rope Skip	1	10	143.6	41.6	2.017365	1.290698
112576	Rope Skip	1	10	148.5	38.9	1.763992	1.261617
100438	Rope Skip	0	11	144.4	42.4	2.033441	1.306863
100594	Rope Skip	0	11	150.6	57.1	2.517597	1.559513
100890	Rope Skip	0	11	146.9	37.4	1.733118	1.229923
100990	Rope Skip	0	11	148.6	40.2	1.820491	1.284462
101112	Rope Skip	0	11	141.4	32.2	1.610486	1.117747
101220	Rope Skip	0	11	142.8	30.9	1.515312	1.097525
101396	Rope Skip	0	11	160.4	52.8	2.052226	1.533048
101546	Rope Skip	0	11	146.4	41.6	1.940936	1.300616
112001	Rope Skip	0	11	158.8	44.4	1.760686	1.391105
112203	Rope Skip	0	11	149.9	39	1.735647	1.268068

112228	Rope Skip	0	11	138.5	34.1	1.777685	1.143316
112438	Rope Skip	0	11	154.8	48.1	2.007258	1.437683
100021	Rope Skip	1	11	144.9	32.5	1.547913	1.134276
100265	Rope Skip	1	11	146.9	34.4	1.594097	1.175842
100417	Rope Skip	1	11	155.3	49	2.03167	1.453945
100649	Rope Skip	1	11	149	41.5	1.869285	1.30803
101012	Rope Skip	1	11	147.7	58	2.658686	1.56061
101040	Rope Skip	1	11	146.9	35.4	1.640437	1.194103
101060	Rope Skip	1	11	164.8	38.7	1.42494	1.311152
111270	Rope Skip	1	11	140.9	38.1	1.919124	1.221884
111570	Rope Skip	1	11	145.7	41.2	1.940788	1.29142
111636	Rope Skip	1	11	157	45	1.825632	1.394866
111785	Rope Skip	1	11	153.5	83.1	3.526828	1.922716
111948	Rope Skip	1	11	139	45	2.329072	1.329135
112852	Rope Skip	1	11	149.7	43.2	1.927703	1.339064
100342	Rope Skip	0	12	153	45	1.922338	1.380669
100518	Rope Skip	0	12	140.3	35.6	1.808567	1.176099
100529	Rope Skip	0	12	155.8	46.6	1.919778	1.417007
101173	Rope Skip	0	12	172	53.1	1.794889	1.580883
112062	Rope Skip	0	12	168.4	86.5	3.050225	2.038119
112075	Rope Skip	0	12	152.7	47.1	2.01996	1.413855
112425	Rope Skip	0	12	153.8	34.3	1.450045	1.195559
112702	Rope Skip	0	12	149	47.8	2.153056	1.411327
112748	Rope Skip	0	12	170.3	46	1.586093	1.457689
112762	Rope Skip	0	12	164.5	54.5	2.014024	1.57508
112845	Rope Skip	0	12	165.2	60.8	2.227837	1.673335
113051	Rope Skip	0	12	161	42.6	1.643455	1.367926
100126	Rope Skip	1	12	153.8	39	1.648739	1.281045
100298	Rope Skip	1	12	166.4	59.3	2.141648	1.655748
100459	Rope Skip	1	12	162.5	54.4	2.060118	1.565913
100672	Rope Skip	1	12	162.2	53.9	2.048741	1.557015
100882	Rope Skip	1	12	157.4	45.6	1.840583	1.406256
101209	Rope Skip	1	12	156.7	54.2	2.2073	1.54046
101256	Rope Skip	1	12	154.1	45.9	1.932891	1.39942
112085	Rope Skip	1	12	142.2	39.2	1.938594	1.245257
112399	Rope Skip	1	12	155.8	54.7	2.253473	1.544556
112411	Rope Skip	1	12	166	60.7	2.202787	1.675059
112594	Rope Skip	1	12	152.6	46.6	2.001137	1.405398
112656	Rope Skip	1	12	152	31.2	1.350416	1.130888
100071	Rope Skip	0	13	160	43.1	1.683594	1.373142
100404	Rope Skip	0	13	172.2	47.2	1.591753	1.484533
100756	Rope Skip	0	13	170.1	58.6	2.025298	1.659613
100874	Rope Skip	0	13	170.4	52.7	1.814978	1.568645
100901	Rope Skip	0	13	173.1	41.8	1.395025	1.393509
100932	Rope Skip	0	13	151.8	42.4	1.840019	1.333011
101313	Rope Skip	0	13	151.3	35.9	1.568255	1.217302
101329	Rope Skip	0	13	165.5	48.3	1.763401	1.47958

101461	Rope Skip	0	13	153.9	40.2	1.697262	1.30243
101550	Rope Skip	0	13	170.9	52.1	1.783831	1.560826
111654	Rope Skip	0	13	171	68.5	2.342601	1.808733
111710	Rope Skip	0	13	155.9	42.7	1.756854	1.352286
111820	Rope Skip	0	13	167.5	59.1	2.106483	1.657065
111849	Rope Skip	0	13	154	35.8	1.509529	1.224029
112263	Rope Skip	0	13	165.5	51.3	1.872929	1.528315
100652	Rope Skip	1	13	167.4	70.1	2.501538	1.815951
100669	Rope Skip	1	13	164.2	69.4	2.574027	1.79241
100703	Rope Skip	1	13	173.4	63.1	2.098607	1.740184
101219	Rope Skip	1	13	152.4	44.5	1.915976	1.370262
112102	Rope Skip	1	13	167.8	61.8	2.194849	1.698562
112162	Rope Skip	1	13	153.6	56.4	2.390544	1.561362
112173	Rope Skip	1	13	159.4	52	2.04657	1.516747
112189	Rope Skip	1	13	166.6	55.4	1.995996	1.597025
112237	Rope Skip	1	13	152.5	41.3	1.775867	1.316699
112534	Rope Skip	1	13	163.3	79.8	2.992475	1.927992
112680	Rope Skip	1	13	148.3	39.1	1.777847	1.264426
112871	Rope Skip	1	13	163	48.4	1.821672	1.472317
100302	Rope Skip	0	14	163.6	53.1	1.983937	1.549815
100567	Rope Skip	0	14	144.5	35.5	1.700171	1.188132
101124	Rope Skip	0	14	166	48.2	1.749165	1.479701
101163	Rope Skip	0	14	150.1	40.6	1.802041	1.296471
101193	Rope Skip	0	14	178.9	60.3	1.884068	1.719371
101366	Rope Skip	0	14	156.5	43	1.755657	1.359456
101444	Rope Skip	0	14	165.3	53.7	1.965299	1.565611
111883	Rope Skip	0	14	180	61.7	1.904321	1.744961
111991	Rope Skip	0	14	158.8	46.9	1.859824	1.432696
112015	Rope Skip	0	14	171.9	57	1.928959	1.641924
112023	Rope Skip	0	14	163.4	50.5	1.891417	1.507799
112359	Rope Skip	0	14	173.7	83.7	2.774124	2.027116
100683	Rope Skip	1	14	152.5	46.2	1.986563	1.398534
111679	Rope Skip	1	14	161	47.7	1.840207	1.453695
111985	Rope Skip	1	14	154.3	40.5	1.701074	1.308995
112212	Rope Skip	1	14	159.1	49.2	1.94368	1.471164
112304	Rope Skip	1	14	175.8	108.9	3.523629	2.346494
112457	Rope Skip	1	14	166.9	59.1	2.121655	1.654709
112867	Rope Skip	1	14	151.4	50.9	2.220578	1.469112
112884	Rope Skip	1	14	162.6	50.6	1.913857	1.50647
112911	Rope Skip	1	14	165.5	69.4	2.533748	1.798022
113163	Rope Skip	1	14	156.5	56.9	2.323184	1.580466
100084	Rope Skip	0	15	179.5	63.5	1.97081	1.770203
100208	Rope Skip	0	15	175.1	57.9	1.888453	1.667967
101474	Rope Skip	0	15	172.2	69.1	2.3303	1.82228
111850	Rope Skip	0	15	157.5	50.3	2.027715	1.48281
112379	Rope Skip	0	15	184.7	70.8	2.075389	1.89826
112558	Rope Skip	0	15	161	45	1.736044	1.408847

112666	Rope Skip	0	15	167.4	54.8	1.955553	1.590718
112674	Rope Skip	0	15	177.4	87.8	2.789891	2.097379
112722	Rope Skip	0	15	179.5	64.1	1.989432	1.779179
112739	Rope Skip	0	15	174.1	57.2	1.887116	1.653334
112820	Rope Skip	0	15	163	55.3	2.081373	1.581718
112838	Rope Skip	0	15	179	67.3	2.100434	1.82439
112944	Rope Skip	0	15	170.8	62.5	2.142418	1.720928
112952	Rope Skip	0	15	173.4	55.4	1.842517	1.622553
100140	Rope Skip	1	15	166.1	80	2.899684	1.943644
100719	Rope Skip	1	15	172.6	71.7	2.406787	1.860551
100774	Rope Skip	1	15	154.7	53	2.2146	1.514293
101401	Rope Skip	1	15	159	67.3	2.662078	1.740687
111819	Rope Skip	1	15	165.6	62.5	2.279079	1.699965
112048	Rope Skip	1	15	162.6	65	2.458512	1.723667
112052	Rope Skip	1	15	160.4	92.8	3.606943	2.076207
112401	Rope Skip	1	15	158.7	52.2	2.072606	1.517233
112517	Rope Skip	1	15	161.1	44.1	1.699212	1.393966
112630	Rope Skip	1	15	163.6	88.9	3.321507	2.044765
112643	Rope Skip	1	15	168.8	55.2	1.937288	1.602233
112714	Rope Skip	1	15	174.4	60.5	1.989127	1.705129
113040	Rope Skip	1	15	158.8	73.7	2.922581	1.826928
113267	Rope Skip	1	15	161.2	53.5	2.058845	1.546993
100221	Rope Skip	0	16	177	69.5	2.218392	1.847976
100482	Rope Skip	0	16	179.2	65.5	2.039695	1.79878
100961	Rope Skip	0	16	174.5	70.8	2.325104	1.855991
101133	Rope Skip	0	16	170	53.1	1.83737	1.57357
101507	Rope Skip	0	16	173.9	76.1	2.516434	1.926831
112251	Rope Skip	0	16	173.4	57.2	1.902383	1.650695
112463	Rope Skip	0	16	167.3	62.1	2.218706	1.700978
112618	Rope Skip	0	16	173.6	65.6	2.176729	1.777742
112621	Rope Skip	0	16	179.7	80.1	2.480484	2.006581
112753	Rope Skip	0	16	175.3	75.1	2.443859	1.919264
112790	Rope Skip	0	16	176.6	76.6	2.456107	1.945475
113115	Rope Skip	0	16	177.3	90.1	2.866205	2.126275
100154	Rope Skip	1	16	177	66	2.106674	1.797329
100836	Rope Skip	1	16	174.3	64.3	2.11649	1.761515
101181	Rope Skip	1	16	161.8	50.9	1.944289	1.508316
111860	Rope Skip	1	16	166	60.2	2.184642	1.667624
111928	Rope Skip	1	16	159	56	2.215102	1.576847
112318	Rope Skip	1	16	164.1	48.4	1.797332	1.476247
112442	Rope Skip	1	16	167	59	2.115529	1.653596
112582	Rope Skip	1	16	166.2	76.4	2.765867	1.896558
113000	Rope Skip	1	16	160.2	99.5	3.87702	2.154457
113067	Rope Skip	1	16	159.8	72	2.819544	1.808638
113135	Rope Skip	1	16	165.6	61.1	2.228027	1.679379
113206	Rope Skip	1	16	170.9	67.7	2.317953	1.796925
113289	Rope Skip	1	16	172.7	63.7	2.135771	1.746261

100016	Rope Skip	0	17	174.4	58.3	1.916795	1.671498
100724	Rope Skip	0	17	178.3	72.6	2.283673	1.897354
111802	Rope Skip	0	17	181.1	71.5	2.180063	1.893498
112346	Rope Skip	0	17	173.3	85.6	2.850209	2.049861
112476	Rope Skip	0	17	172.6	69.5	2.332938	1.829628
112691	Rope Skip	0	17	171.8	64.1	2.171759	1.748525
113017	Rope Skip	0	17	170.5	72.7	2.500838	1.865387
113023	Rope Skip	0	17	172.8	64.3	2.153394	1.755491
113081	Rope Skip	0	17	173	82.2	2.7465	2.004287
113095	Rope Skip	0	17	183.4	94.3	2.80358	2.208433
112972	Rope Skip	1	17	168.1	48.4	1.712813	1.490408
112987	Rope Skip	1	17	163.6	51.4	1.920421	1.52293
112993	Rope Skip	1	17	171.5	69.2	2.352761	1.820756
113079	Rope Skip	1	17	162.9	72.8	2.7434	1.833328
113103	Rope Skip	1	17	155.7	49.1	2.02537	1.457025
113227	Rope Skip	1	17	170.9	62.3	2.133064	1.718363
113455	Rope Skip	1	17	171.5	71.6	2.43436	1.854449
113463	Rope Skip	1	17	159.5	62.2	2.444945	1.670533
113494	Rope Skip	1	17	161	56.4	2.175842	1.590757
113517	Rope Skip	1	17	164.1	53.9	2.001574	1.56422
101059	Rope Skip	0	18	181.8	87	2.632276	2.107447
101534	Rope Skip	0	18	167.7	53.1	1.888115	1.565096
111642	Rope Skip	0	18	184	86.4	2.551985	2.109654
111721	Rope Skip	0	18	176.6	61.4	1.968734	1.727286
112299	Rope Skip	0	18	164.1	61	2.265232	1.671859
112964	Rope Skip	0	18	178.7	71.6	2.242146	1.884928
113157	Rope Skip	0	18	170.4	62.5	2.152488	1.719329
113297	Rope Skip	0	18	169.5	54.9	1.910878	1.600166
113305	Rope Skip	0	18	183.5	72	2.138259	1.910553
113338	Rope Skip	0	18	182.5	67.8	2.035654	1.845781
113353	Rope Skip	0	18	163.5	57.7	2.158442	1.620239
113375	Rope Skip	0	18	168.7	88.4	3.106148	2.06353
113380	Rope Skip	0	18	179.8	69.4	2.146743	1.85807
100117	Rope Skip	1	18	171.8	70.1	2.375044	1.834724
100549	Rope Skip	1	18	164.9	52.6	1.934392	1.546795
111745	Rope Skip	1	18	164.6	62	2.2884	1.688579
112498	Rope Skip	1	18	159.5	62.1	2.441014	1.669088
112503	Rope Skip	1	18	158.2	58.6	2.341449	1.612579
113251	Rope Skip	1	18	163	46.8	1.761451	1.445938
113312	Rope Skip	1	18	158.6	55.8	2.218339	1.572245
113412	Rope Skip	1	18	161.5	55.4	2.12405	1.577464
113426	Rope Skip	1	18	164.7	75.9	2.798044	1.883093
113435	Rope Skip	1	18	168.2	65.2	2.3046	1.749847
113477	Rope Skip	1	18	154.6	51.6	2.158891	1.492265
3079	Sweeping	0	5	114.5	21.033	1.604317	0.817612
4054	Sweeping	1	5	120.667	19.967	1.37131	0.811765
4090	Sweeping	1	5	110.567	15.5	1.267887	0.68428

3042	Sweeping	0	6	120.9	23.3	1.594055	0.88271
3078	Sweeping	0	6	114.35	19.5	1.491291	0.784595
4060	Sweeping	0	6	121.633	22.6	1.527586	0.870431
4066	Sweeping	0	6	119.533	21.4	1.497746	0.839446
3103	Sweeping	0	6	129.5	27.225	1.623411	0.986302
4077	Sweeping	1	6	110.867	18.7	1.521378	0.757768
4087	Sweeping	1	6	119.033	31.617	2.231444	1.03379
4073	Sweeping	1	6	121.4	23.7	1.608092	0.892286
3133	Sweeping	1	6	124.55	23.8	1.534227	0.903437
4075	Sweeping	1	6	130.567	28.617	1.678639	1.01641
4047	Sweeping	1	6	127.633	24.3	1.491696	0.922493
3122	Sweeping	1	6	131.4	24.6	1.424769	0.939369
3135	Sweeping	0	7	120.45	23.475	1.61805	0.88496
3108	Sweeping	0	7	123	27.1	1.791262	0.963983
3005	Sweeping	0	7	127	25.067	1.554157	0.936193
3049	Sweeping	0	7	122.35	23.2	1.549816	0.884842
3106	Sweeping	0	7	130.95	41.075	2.395337	1.235899
4029	Sweeping	0	7	128.3	27.05	1.643289	0.979267
4014	Sweeping	0	7	118.7	20.2	1.433672	0.811541
4063	Sweeping	0	7	141.033	55.683	2.799504	1.499066
3028	Sweeping	0	7	127.3	24.55	1.514937	0.926625
4053	Sweeping	0	7	128.567	28.567	1.728246	1.00926
3043	Sweeping	0	7	129	27.475	1.651043	0.989643
3064	Sweeping	0	7	129.2	32.1	1.923003	1.076671
4078	Sweeping	1	7	126.8	25.967	1.61504	0.953527
3080	Sweeping	1	7	116.45	21.35	1.574414	0.829752
4006	Sweeping	1	7	129.333	25.267	1.51055	0.947011
4071	Sweeping	1	7	122	21.617	1.452365	0.850876
3119	Sweeping	0	8	131.8	28.483	1.639664	1.017633
3003	Sweeping	0	8	130.5	32.275	1.895157	1.084118
3054	Sweeping	0	8	135.9	66.433	3.597037	1.624301
4032	Sweeping	0	8	129.767	26.8	1.591499	0.97879
4084	Sweeping	0	8	136.667	27.417	1.467887	1.011404
4021	Sweeping	0	8	132.667	26.583	1.510352	0.983095
3101	Sweeping	0	8	137	36.075	1.922052	1.173389
4097	Sweeping	0	8	138.033	33.75	1.771364	1.135469
3104	Sweeping	0	8	132.167	29.467	1.686902	1.037534
4061	Sweeping	0	8	135.933	31.55	1.707456	1.088409
3033	Sweeping	1	8	125.3	24.7	1.573239	0.923848
4088	Sweeping	1	8	128.033	25.117	1.53223	0.940211
3001	Sweeping	1	8	125.5	24.05	1.52696	0.911269
4030	Sweeping	1	8	131.733	31.583	1.81997	1.075557
4049	Sweeping	1	8	123.367	28.55	1.875893	0.992559
4002	Sweeping	1	8	138.267	33.2	1.736605	1.126235
3066	Sweeping	1	8	132.05	28.975	1.661677	1.02782
3008	Sweeping	1	8	134.6	31.8	1.755242	1.088777
4003	Sweeping	1	8	128.367	21.517	1.305795	0.866047

4035	Sweeping	1	8	157.033	63.383	2.570342	1.677151
3039	Sweeping	1	8	126.55	22.65	1.414308	0.885264
4033	Sweeping	1	8	132.467	32.033	1.825502	1.086161
4034	Sweeping	1	8	131.233	31.333	1.819348	1.069356
3111	Sweeping	0	9	136.5	34.875	1.871755	1.150565
4062	Sweeping	0	9	138.367	39.617	2.069267	1.238872
4005	Sweeping	0	9	134.233	31.9	1.770403	1.089438
4042	Sweeping	0	9	132.967	31.65	1.790136	1.08077
3068	Sweeping	0	9	141.1	38.525	1.935034	1.229887
3100	Sweeping	0	9	128.45	26.925	1.631877	0.977283
3127	Sweeping	0	9	147.5	35.7	1.640908	1.201474
3132	Sweeping	0	9	135.267	32.95	1.800826	1.111955
3091	Sweeping	0	9	140.45	36.875	1.869341	1.199075
3048	Sweeping	1	9	134.367	32.483	1.799165	1.100536
4027	Sweeping	1	9	139.867	41.717	2.132468	1.279223
3061	Sweeping	1	9	138.167	34.2	1.791503	1.144026
4064	Sweeping	1	9	133.233	39.933	2.249616	1.225668
4023	Sweeping	1	9	134.333	30.917	1.713294	1.071569
4104	Sweeping	1	9	133.133	32.633	1.841135	1.099238
4092	Sweeping	1	9	153	61.65	2.633602	1.635377
3056	Sweeping	1	9	134.15	30.375	1.687854	1.060851
4020	Sweeping	1	9	137.2	30.8	1.636223	1.078376
3006	Sweeping	1	9	134.75	29.375	1.617783	1.043768
3072	Sweeping	1	9	132.15	28.2	1.614785	1.013247
3118	Sweeping	1	9	146.95	42.425	1.964639	1.31638
4017	Sweeping	1	9	140.5	46.083	2.334469	1.351984
4102	Sweeping	1	9	136.8	33.217	1.774959	1.121792
3027	Sweeping	0	10	137.75	32.9	1.733855	1.119088
4013	Sweeping	0	10	149.8	45.617	2.03284	1.379214
3125	Sweeping	0	10	153.5	41.817	1.774746	1.328976
4069	Sweeping	0	10	133.95	26.45	1.474145	0.984194
3029	Sweeping	0	10	151.85	36.575	1.586188	1.231328
4028	Sweeping	0	10	148.667	50.267	2.274332	1.448759
4055	Sweeping	0	10	147.633	41.6	1.908651	1.304947
4041	Sweeping	0	10	135.467	38.3	2.087045	1.206378
3126	Sweeping	0	10	144	31.3	1.509452	1.108815
3022	Sweeping	0	10	144.8	32.875	1.567937	1.140983
4025	Sweeping	0	10	147.633	34.767	1.595146	1.184907
3070	Sweeping	0	10	140	35.7	1.821429	1.176875
4052	Sweeping	0	10	140.867	42.75	2.154357	1.299828
4040	Sweeping	1	10	150.667	59.333	2.613726	1.592302
3041	Sweeping	1	10	142.45	36.583	1.802828	1.20067
4100	Sweeping	1	10	138.3	34.45	1.801129	1.148954
4070	Sweeping	1	10	138.5	38.483	2.006178	1.220137
3123	Sweeping	1	10	145.45	33.125	1.565772	1.147677
4048	Sweeping	1	10	132.8	24.033	1.362736	0.931568
3114	Sweeping	1	10	140.55	27.5	1.392102	1.024362

4105	Sweeping	1	10	151.533	52.033	2.266024	1.487127
4068	Sweeping	1	10	150.867	56.45	2.480136	1.551029
4081	Sweeping	1	10	142.733	35.6	1.747436	1.184141
3086	Sweeping	0	11	144	38.4	1.851852	1.23768
3102	Sweeping	0	11	149.6	38.05	1.700167	1.250368
4050	Sweeping	0	11	142.533	52.433	2.580916	1.457457
4026	Sweeping	0	11	158.633	61.25	2.433991	1.653187
3128	Sweeping	0	11	145.067	35.917	1.706722	1.197475
3002	Sweeping	0	11	141.55	31.225	1.558413	1.099878
3034	Sweeping	0	11	142.8	42.525	2.085393	1.303165
3052	Sweeping	0	11	154.4	41.9	1.757598	1.333481
4019	Sweeping	0	11	144.633	34.1	1.63012	1.163123
3038	Sweeping	0	11	143.3	34.6	1.684937	1.167969
4037	Sweeping	0	11	138.033	33.6	1.763492	1.132752
4010	Sweeping	0	11	144.567	48.3	2.311047	1.402357
3017	Sweeping	1	11	133.9	29.533	1.6472	1.044161
4096	Sweeping	1	11	155.8	77.033	3.173525	1.856816
3060	Sweeping	1	11	157.75	63.875	2.566801	1.687183
3000	Sweeping	1	11	151	52.275	2.292663	1.488762
4089	Sweeping	1	11	157.5	54.6	2.201058	1.549689
4101	Sweeping	1	11	137.633	28.2	1.488689	1.029708
3120	Sweeping	1	11	157.667	43.3	1.741832	1.368573
4016	Sweeping	1	11	148.7	42.067	1.902478	1.316558
4083	Sweeping	1	11	164.633	61.1	2.254278	1.675485
3009	Sweeping	1	11	164.5	58.367	2.156928	1.63423
3113	Sweeping	0	12	157.05	62.475	2.532972	1.664258
3134	Sweeping	0	12	164.25	76.775	2.845831	1.892683
4008	Sweeping	0	12	135.9	27.967	1.514283	1.019987
4038	Sweeping	0	12	154.6	37.633	1.574526	1.259285
4039	Sweeping	0	12	155.967	40.733	1.674484	1.318644
4085	Sweeping	0	12	150.533	56.633	2.499229	1.552367
4046	Sweeping	0	12	153.633	36.45	1.544289	1.234763
4108	Sweeping	0	12	135.633	28.783	1.564607	1.035079
3095	Sweeping	0	12	160.9	78.967	3.050237	1.905923
4031	Sweeping	0	12	154.733	52.733	2.202503	1.510313
3058	Sweeping	0	12	175.4	65.95	2.143659	1.790141
3088	Sweeping	0	12	156.5	49.283	2.012188	1.462912
3004	Sweeping	0	12	159.95	48.775	1.906465	1.467412
4076	Sweeping	1	12	156.433	49.467	2.021431	1.465598
3124	Sweeping	1	12	167.65	50.217	1.786667	1.518628
3065	Sweeping	1	12	152.95	33.95	1.451245	1.186373
4109	Sweeping	1	12	157.067	51.433	2.084836	1.499044
3085	Sweeping	1	12	158	49.3	1.974844	1.468727
4098	Sweeping	1	12	158.133	46.733	1.868868	1.427567
4099	Sweeping	1	12	158.9	48.85	1.934714	1.464796
4103	Sweeping	1	12	151.467	40.75	1.776199	1.303722
4106	Sweeping	1	12	157.067	46.983	1.904456	1.427835

4107	Sweeping	1	12	152.767	50.117	2.147464	1.462115
4110	Sweeping	1	12	152.467	41.917	1.803178	1.327128
3094	Sweeping	1	12	168.7	51.575	1.812212	1.544396
4079	Sweeping	1	12	152.133	59.8	2.583773	1.605179
4057	Sweeping	0	13	158.267	47.45	1.894329	1.439788
3131	Sweeping	0	13	159.1	45.875	1.812323	1.416831
4056	Sweeping	0	13	159.533	51.317	2.016323	1.506499
4074	Sweeping	0	13	171.533	62.25	2.115651	1.72014
3087	Sweeping	0	13	167.5	48.275	1.72065	1.486229
3014	Sweeping	0	13	169.8	53.75	1.864245	1.583161
3020	Sweeping	0	13	147.5	33.7	1.54898	1.164793
3024	Sweeping	0	13	173.15	56.45	1.882864	1.638083
4091	Sweeping	0	13	168.5	66.033	2.32574	1.76308
3035	Sweeping	0	13	165.6	47.9	1.746686	1.473331
3115	Sweeping	0	13	152.25	38.525	1.661989	1.26753
3044	Sweeping	0	13	160.233	43.9	1.70986	1.387591
4086	Sweeping	0	13	167.767	83.95	2.982687	2.002591
3110	Sweeping	0	13	169.75	64.033	2.222206	1.739245
4012	Sweeping	0	13	156.467	62.25	2.542693	1.658585
3010	Sweeping	1	13	173.75	67.35	2.23094	1.803709
4022	Sweeping	1	13	158.967	49.317	1.951564	1.472557
3069	Sweeping	1	13	163.25	49.675	1.863938	1.493958
3015	Sweeping	1	13	164.5	45	1.662956	1.420909
4093	Sweeping	1	13	155.333	53.483	2.216605	1.524164
4067	Sweeping	1	13	159.167	59.85	2.362425	1.634933
4094	Sweeping	1	13	167.167	79.983	2.862178	1.948361
4043	Sweeping	1	13	162.8	54.133	2.042459	1.562917
4080	Sweeping	1	13	157.433	52.817	2.130994	1.522007
3021	Sweeping	0	14	159.75	48.225	1.88969	1.457767
4045	Sweeping	0	14	164.7	56.733	2.091455	1.61024
4007	Sweeping	0	14	163.933	51.367	1.911399	1.523632
3117	Sweeping	0	14	162.05	53.35	2.031591	1.547883
3055	Sweeping	0	14	184.6	71.3	2.092311	1.905049
3096	Sweeping	0	14	170.567	106.5	3.660662	2.290931
3081	Sweeping	0	14	179.8	68.05	2.104984	1.838543
3047	Sweeping	0	14	174.3	77.85	2.5625	1.952313
3097	Sweeping	0	14	182.3	97.35	2.929292	2.241213
3053	Sweeping	1	14	170.6	50.125	1.722251	1.527658
3098	Sweeping	1	14	159.75	44.825	1.756461	1.401561
3025	Sweeping	1	14	156.35	51.275	2.097538	1.493853
4015	Sweeping	1	14	154.667	45.9	1.918745	1.401459
3112	Sweeping	1	14	160.3	69.25	2.694963	1.773347
4051	Sweeping	1	14	158.967	58.9	2.33078	1.620117
4082	Sweeping	1	14	168.133	47.517	1.680905	1.475837
4095	Sweeping	1	14	167.4	86.733	3.095091	2.036257
4024	Sweeping	1	14	175.533	54.767	1.777466	1.62039
3011	Sweeping	1	14	170.25	60.675	2.09332	1.691554

4036	Sweeping	1	14	157	72.7	2.94941	1.805378
4065	Sweeping	0	15	170	77.817	2.69263	1.932636
3023	Sweeping	0	15	174.15	71.4	2.354243	1.86295
4009	Sweeping	0	15	190.4	117.15	3.231532	2.518894
3075	Sweeping	1	15	166.5	68.125	2.457412	1.784437
3093	Sweeping	1	15	166.2	53.925	1.952217	1.572516
3016	Sweeping	1	15	159.4	61.633	2.425698	1.661913
3026	Sweeping	0	16	180.4	71.4	2.193942	1.889171
3032	Sweeping	0	16	170.3	59.7	2.058472	1.677076
3084	Sweeping	1	16	165.65	54.125	1.972491	1.573581
4011	Sweeping	1	16	163.633	62.1	2.319262	1.6861
4058	Sweeping	1	16	165.733	68.533	2.495064	1.786903
1146	Sweeping	1	8	124.5	28.6	1.845132	0.997101
1306	Sweeping	1	8	135.9	26.5	1.434852	0.990855
1184	Sweeping	1	8	134.6	30.5	1.683487	1.064609
1104	Sweeping	0	9	131	25.5	1.485927	0.956542
1314	Sweeping	0	9	134.2	30.9	1.715748	1.070831
1315	Sweeping	0	9	134.3	27.1	1.50251	0.998161
1123	Sweeping	0	9	128	23.5	1.434326	0.907065
1140	Sweeping	0	9	124	28.1	1.827523	0.986114
1317	Sweeping	1	9	139.2	29.9	1.543095	1.067419
1304	Sweeping	1	9	136.5	31.9	1.712085	1.096694
1169	Sweeping	0	10	146.1	57.4	2.689128	1.54522
1102	Sweeping	0	10	143	41.8	2.04411	1.291886
1329	Sweeping	0	11	143.1	33.4	1.63105	1.145372
1190	Sweeping	0	11	156.8	62.4	2.538005	1.662133
1247	Sweeping	0	11	158.3	92.8	3.703277	2.065389
1113	Sweeping	0	11	144	34.5	1.663773	1.168407
1122	Sweeping	1	11	147	52.5	2.429543	1.476409
1197	Sweeping	1	11	150.7	54.3	2.390966	1.518307
1249	Sweeping	1	11	153.1	57.9	2.470178	1.581514
1225	Sweeping	1	11	163	62.9	2.367421	1.695141
1192	Sweeping	1	11	156.9	48.1	1.953886	1.445383
1204	Sweeping	1	11	150.1	55.1	2.445627	1.527879
1106	Sweeping	1	11	154	46.1	1.943835	1.402335
1220	Sweeping	0	12	145.5	38.6	1.823313	1.246252
1235	Sweeping	1	12	153.8	88.6	3.745597	1.99168
1299	Sweeping	1	12	139.3	33.4	1.721251	1.133218
1208	Sweeping	1	12	154.9	48.7	2.029673	1.447671
1118	Sweeping	1	12	151	59.4	2.605149	1.594664
1229	Sweeping	1	12	151.3	46.6	2.035673	1.40064
1108	Sweeping	1	12	162	63.7	2.427221	1.702544
1112	Sweeping	0	13	164.4	57.1	2.112674	1.614666
1282	Sweeping	0	13	137.8	29.7	1.564077	1.05932
1254	Sweeping	0	13	156.7	64	2.606405	1.684493
1326	Sweeping	0	13	154.4	67	2.810478	1.716418
1107	Sweeping	1	13	164	57.2	2.12671	1.614626

1187	Sweeping	1	13	143.4	77.4	3.76394	1.801363
1222	Sweeping	1	13	160.5	47.8	1.855572	1.45354
1268	Sweeping	0	14	169.4	57.5	2.003738	1.640102
100601	Sweeping	0	8	142.2	33.6	1.661652	1.146186
100769	Sweeping	0	8	127.8	29.1	1.781686	1.016929
100974	Sweeping	0	8	120.2	23.2	1.605754	0.878646
100989	Sweeping	0	8	130.4	29.3	1.723108	1.028863
101240	Sweeping	0	8	132.2	30.7	1.75661	1.060766
101419	Sweeping	0	8	133.4	29.8	1.674575	1.047673
111695	Sweeping	0	8	126	22.9	1.442429	0.888969
111777	Sweeping	0	8	125.1	25.7	1.642171	0.943181
111963	Sweeping	0	8	131.1	29.4	1.710574	1.03294
112090	Sweeping	0	8	126.7	23.8	1.482599	0.909587
112125	Sweeping	0	8	125	23.6	1.5104	0.900632
112198	Sweeping	0	8	127.2	26.5	1.637841	0.965207
113527	Sweeping	0	8	142.5	42.1	2.073253	1.295065
113546	Sweeping	0	8	134.6	31	1.711085	1.07396
100424	Sweeping	1	8	131.2	24.9	1.446544	0.944942
100841	Sweeping	1	8	134.7	27.3	1.504622	1.003298
100912	Sweeping	1	8	127.1	23.4	1.448521	0.90246
101300	Sweeping	1	8	135.7	35.1	1.906108	1.151864
111581	Sweeping	1	8	127.9	27.6	1.687206	0.9887
111665	Sweeping	1	8	132	28.3	1.624197	1.014721
111703	Sweeping	1	8	129.9	34.3	2.032712	1.11814
111734	Sweeping	1	8	132.3	30.9	1.765382	1.064796
111797	Sweeping	1	8	140.3	35.1	1.783166	1.167186
111901	Sweeping	1	8	124.5	23.7	1.529008	0.90125
111932	Sweeping	1	8	130	27.2	1.609467	0.987322
112484	Sweeping	1	8	139	37.7	1.951245	1.208447
100193	Sweeping	0	9	135.5	49.1	2.674256	1.378938
100339	Sweeping	0	9	133	28.5	1.611171	1.021623
100374	Sweeping	0	9	133	32.4	1.831647	1.094577
100505	Sweeping	0	9	143.3	26.9	1.309966	1.020087
100614	Sweeping	0	9	139.4	36.8	1.893748	1.194205
100816	Sweeping	0	9	129.3	26.1	1.561146	0.963579
100923	Sweeping	0	9	132.4	28.3	1.614397	1.015939
101140	Sweeping	0	9	137.1	34.6	1.840777	1.14767
101230	Sweeping	0	9	131.4	27.1	1.569562	0.989561
112110	Sweeping	0	9	133.2	38.7	2.181235	1.205049
112335	Sweeping	0	9	148.8	48.9	2.208528	1.427941
112772	Sweeping	0	9	136.5	34.4	1.846261	1.14211
112895	Sweeping	0	9	143.4	34.4	1.672862	1.164656
113182	Sweeping	0	9	124.7	26.2	1.684878	0.951797
113347	Sweeping	0	9	139.2	28.8	1.486326	1.046117
100234	Sweeping	1	9	133.3	29.6	1.665833	1.043575
100576	Sweeping	1	9	128.1	27.6	1.681941	0.989313
100589	Sweeping	1	9	127.2	27.6	1.705827	0.986552

100953	Sweeping	1	9	155.5	38.6	1.596344	1.279525
101071	Sweeping	1	9	144.2	37	1.77939	1.213872
101104	Sweeping	1	9	133.4	27.6	1.550949	1.00534
101373	Sweeping	1	9	134.2	32	1.776826	1.091167
112564	Sweeping	1	9	131.5	28.7	1.659703	1.020872
112780	Sweeping	1	9	142.2	35.4	1.750669	1.17881
113034	Sweeping	1	9	141.2	30.4	1.524769	1.083089
113147	Sweeping	1	9	130	35	2.071006	1.1307
113199	Sweeping	1	9	137.9	29.2	1.535516	1.049993
113212	Sweeping	1	9	129.6	26.3	1.565834	0.968432
113274	Sweeping	1	9	137	31.5	1.678299	1.090857
100042	Sweeping	0	10	143	49	2.396205	1.407158
100160	Sweeping	0	10	149.4	48.5	2.172904	1.423917
100356	Sweeping	0	10	139.2	33.9	1.749529	1.141985
100471	Sweeping	0	10	132.8	28.7	1.627368	1.024861
100787	Sweeping	0	10	145	36.3	1.726516	1.204105
100804	Sweeping	0	10	129.5	26.4	1.574216	0.970114
100861	Sweeping	0	10	142.2	42.2	2.086956	1.295635
101082	Sweeping	0	10	146.5	30.5	1.4211	1.100968
101150	Sweeping	0	10	127.1	33.1	2.048976	1.087495
101265	Sweeping	0	10	133	28.7	1.622477	1.025472
101428	Sweeping	0	10	137.2	29.4	1.561849	1.051731
101459	Sweeping	0	10	140.9	35.5	1.78816	1.176309
111564	Sweeping	0	10	140.6	31	1.568162	1.092688
111621	Sweeping	0	10	137.9	32	1.682757	1.102994
111683	Sweeping	0	10	142.6	35.2	1.731026	1.176532
111977	Sweeping	0	10	145.3	48.7	2.306735	1.411417
113530	Sweeping	0	10	146.5	45.2	2.106023	1.360354
100258	Sweeping	1	10	151.6	59.3	2.580217	1.595726
100360	Sweeping	1	10	137.8	41.9	2.206559	1.274692
100397	Sweeping	1	10	139.8	34	1.739661	1.145747
100445	Sweeping	1	10	146.2	42.4	1.983678	1.313297
100851	Sweeping	1	10	149.1	43.2	1.943249	1.336934
100946	Sweeping	1	10	140.5	37	1.874343	1.201429
101331	Sweeping	1	10	150.9	41.3	1.813726	1.311205
111839	Sweeping	1	10	155.5	46	1.902379	1.406091
111911	Sweeping	1	10	143	33.6	1.643112	1.148737
111950	Sweeping	1	10	143.6	41.6	2.017365	1.290698
112576	Sweeping	1	10	148.5	38.9	1.763992	1.261617
100438	Sweeping	0	11	144.4	42.4	2.033441	1.306863
100594	Sweeping	0	11	150.6	57.1	2.517597	1.559513
100791	Sweeping	0	11	142.2	36.2	1.790232	1.193062
100890	Sweeping	0	11	146.9	37.4	1.733118	1.229923
100990	Sweeping	0	11	148.6	40.2	1.820491	1.284462
101112	Sweeping	0	11	141.4	32.2	1.610486	1.117747
101220	Sweeping	0	11	142.8	30.9	1.515312	1.097525
101396	Sweeping	0	11	160.4	52.8	2.052226	1.533048

101546	Sweeping	0	11	146.4	41.6	1.940936	1.300616
112001	Sweeping	0	11	158.8	44.4	1.760686	1.391105
112203	Sweeping	0	11	149.9	39	1.735647	1.268068
112228	Sweeping	0	11	138.5	34.1	1.777685	1.143316
112438	Sweeping	0	11	154.8	48.1	2.007258	1.437683
100021	Sweeping	1	11	144.9	32.5	1.547913	1.134276
100265	Sweeping	1	11	146.9	34.4	1.594097	1.175842
100417	Sweeping	1	11	155.3	49	2.03167	1.453945
100649	Sweeping	1	11	149	41.5	1.869285	1.30803
101012	Sweeping	1	11	147.7	58	2.658686	1.56061
101040	Sweeping	1	11	146.9	35.4	1.640437	1.194103
101060	Sweeping	1	11	164.8	38.7	1.42494	1.311152
111270	Sweeping	1	11	140.9	38.1	1.919124	1.221884
111570	Sweeping	1	11	145.7	41.2	1.940788	1.29142
111636	Sweeping	1	11	157	45	1.825632	1.394866
111785	Sweeping	1	11	153.5	83.1	3.526828	1.922716
111948	Sweeping	1	11	139	45	2.329072	1.329135
112852	Sweeping	1	11	149.7	43.2	1.927703	1.339064
100131	Sweeping	0	12	165.4	53.1	1.940991	1.556552
100342	Sweeping	0	12	153	45	1.922338	1.380669
100518	Sweeping	0	12	140.3	35.6	1.808567	1.176099
100529	Sweeping	0	12	155.8	46.6	1.919778	1.417007
100692	Sweeping	0	12	161.1	47	1.810951	1.442538
101173	Sweeping	0	12	172	53.1	1.794889	1.580883
112062	Sweeping	0	12	168.4	86.5	3.050225	2.038119
112075	Sweeping	0	12	152.7	47.1	2.01996	1.413855
112425	Sweeping	0	12	153.8	34.3	1.450045	1.195559
112702	Sweeping	0	12	149	47.8	2.153056	1.411327
112748	Sweeping	0	12	170.3	46	1.586093	1.457689
112762	Sweeping	0	12	164.5	54.5	2.014024	1.57508
112845	Sweeping	0	12	165.2	60.8	2.227837	1.673335
113051	Sweeping	0	12	161	42.6	1.643455	1.367926
100126	Sweeping	1	12	153.8	39	1.648739	1.281045
100179	Sweeping	1	12	169.1	61.3	2.143746	1.696339
100298	Sweeping	1	12	166.4	59.3	2.141648	1.655748
100459	Sweeping	1	12	162.5	54.4	2.060118	1.565913
100672	Sweeping	1	12	162.2	53.9	2.048741	1.557015
100882	Sweeping	1	12	157.4	45.6	1.840583	1.406256
101209	Sweeping	1	12	156.7	54.2	2.2073	1.54046
101256	Sweeping	1	12	154.1	45.9	1.932891	1.39942
112085	Sweeping	1	12	142.2	39.2	1.938594	1.245257
112399	Sweeping	1	12	155.8	54.7	2.253473	1.544556
112411	Sweeping	1	12	166	60.7	2.202787	1.675059
112594	Sweeping	1	12	152.6	46.6	2.001137	1.405398
112656	Sweeping	1	12	152	31.2	1.350416	1.130888
100071	Sweeping	0	13	160	43.1	1.683594	1.373142
100404	Sweeping	0	13	172.2	47.2	1.591753	1.484533

100756	Sweeping	0	13	170.1	58.6	2.025298	1.659613
100874	Sweeping	0	13	170.4	52.7	1.814978	1.568645
100901	Sweeping	0	13	173.1	41.8	1.395025	1.393509
100932	Sweeping	0	13	151.8	42.4	1.840019	1.333011
101313	Sweeping	0	13	151.3	35.9	1.568255	1.217302
101329	Sweeping	0	13	165.5	48.3	1.763401	1.47958
101461	Sweeping	0	13	153.9	40.2	1.697262	1.30243
101550	Sweeping	0	13	170.9	52.1	1.783831	1.560826
111654	Sweeping	0	13	171	68.5	2.342601	1.808733
111710	Sweeping	0	13	155.9	42.7	1.756854	1.352286
111820	Sweeping	0	13	167.5	59.1	2.106483	1.657065
111849	Sweeping	0	13	154	35.8	1.509529	1.224029
112263	Sweeping	0	13	165.5	51.3	1.872929	1.528315
100652	Sweeping	1	13	167.4	70.1	2.501538	1.815951
100669	Sweeping	1	13	164.2	69.4	2.574027	1.79241
100703	Sweeping	1	13	173.4	63.1	2.098607	1.740184
101219	Sweeping	1	13	152.4	44.5	1.915976	1.370262
112102	Sweeping	1	13	167.8	61.8	2.194849	1.698562
112162	Sweeping	1	13	153.6	56.4	2.390544	1.561362
112173	Sweeping	1	13	159.4	52	2.04657	1.516747
112189	Sweeping	1	13	166.6	55.4	1.995996	1.597025
112237	Sweeping	1	13	152.5	41.3	1.775867	1.316699
112534	Sweeping	1	13	163.3	79.8	2.992475	1.927992
112680	Sweeping	1	13	148.3	39.1	1.777847	1.264426
112871	Sweeping	1	13	163	48.4	1.821672	1.472317
100102	Sweeping	0	14	179.4	96.7	3.004565	2.219003
100302	Sweeping	0	14	163.6	53.1	1.983937	1.549815
100567	Sweeping	0	14	144.5	35.5	1.700171	1.188132
101124	Sweeping	0	14	166	48.2	1.749165	1.479701
101163	Sweeping	0	14	150.1	40.6	1.802041	1.296471
101193	Sweeping	0	14	178.9	60.3	1.884068	1.719371
101366	Sweeping	0	14	156.5	43	1.755657	1.359456
101444	Sweeping	0	14	165.3	53.7	1.965299	1.565611
111870	Sweeping	0	14	173	68.6	2.292091	1.818515
111883	Sweeping	0	14	180	61.7	1.904321	1.744961
111991	Sweeping	0	14	158.8	46.9	1.859824	1.432696
112015	Sweeping	0	14	171.9	57	1.928959	1.641924
112023	Sweeping	0	14	163.4	50.5	1.891417	1.507799
100683	Sweeping	1	14	152.5	46.2	1.986563	1.398534
111679	Sweeping	1	14	161	47.7	1.840207	1.453695
111985	Sweeping	1	14	154.3	40.5	1.701074	1.308995
112212	Sweeping	1	14	159.1	49.2	1.94368	1.471164
112304	Sweeping	1	14	175.8	108.9	3.523629	2.346494
112457	Sweeping	1	14	166.9	59.1	2.121655	1.654709
112867	Sweeping	1	14	151.4	50.9	2.220578	1.469112
112884	Sweeping	1	14	162.6	50.6	1.913857	1.50647
112911	Sweeping	1	14	165.5	69.4	2.533748	1.798022

113163	Sweeping	1	14	156.5	56.9	2.323184	1.580466
100208	Sweeping	0	15	175.1	57.9	1.888453	1.667967
101474	Sweeping	0	15	172.2	69.1	2.3303	1.82228
111850	Sweeping	0	15	157.5	50.3	2.027715	1.48281
112379	Sweeping	0	15	184.7	70.8	2.075389	1.89826
112558	Sweeping	0	15	161	45	1.736044	1.408847
112666	Sweeping	0	15	167.4	54.8	1.955553	1.590718
112674	Sweeping	0	15	177.4	87.8	2.789891	2.097379
112722	Sweeping	0	15	179.5	64.1	1.989432	1.779179
112739	Sweeping	0	15	174.1	57.2	1.887116	1.653334
112820	Sweeping	0	15	163	55.3	2.081373	1.581718
112838	Sweeping	0	15	179	67.3	2.100434	1.82439
112944	Sweeping	0	15	170.8	62.5	2.142418	1.720928
112952	Sweeping	0	15	173.4	55.4	1.842517	1.622553
100140	Sweeping	1	15	166.1	80	2.899684	1.943644
100719	Sweeping	1	15	172.6	71.7	2.406787	1.860551
100774	Sweeping	1	15	154.7	53	2.2146	1.514293
101401	Sweeping	1	15	159	67.3	2.662078	1.740687
111819	Sweeping	1	15	165.6	62.5	2.279079	1.699965
112048	Sweeping	1	15	162.6	65	2.458512	1.723667
112052	Sweeping	1	15	160.4	92.8	3.606943	2.076207
112401	Sweeping	1	15	158.7	52.2	2.072606	1.517233
112517	Sweeping	1	15	161.1	44.1	1.699212	1.393966
112630	Sweeping	1	15	163.6	88.9	3.321507	2.044765
112643	Sweeping	1	15	168.8	55.2	1.937288	1.602233
112714	Sweeping	1	15	174.4	60.5	1.989127	1.705129
113040	Sweeping	1	15	158.8	73.7	2.922581	1.826928
100221	Sweeping	0	16	177	69.5	2.218392	1.847976
100482	Sweeping	0	16	179.2	65.5	2.039695	1.79878
100961	Sweeping	0	16	174.5	70.8	2.325104	1.855991
101133	Sweeping	0	16	170	53.1	1.83737	1.57357
101507	Sweeping	0	16	173.9	76.1	2.516434	1.926831
112251	Sweeping	0	16	173.4	57.2	1.902383	1.650695
112463	Sweeping	0	16	167.3	62.1	2.218706	1.700978
112618	Sweeping	0	16	173.6	65.6	2.176729	1.777742
112621	Sweeping	0	16	179.7	80.1	2.480484	2.006581
112753	Sweeping	0	16	175.3	75.1	2.443859	1.919264
112790	Sweeping	0	16	176.6	76.6	2.456107	1.945475
113115	Sweeping	0	16	177.3	90.1	2.866205	2.126275
100154	Sweeping	1	16	177	66	2.106674	1.797329
100836	Sweeping	1	16	174.3	64.3	2.11649	1.761515
101181	Sweeping	1	16	161.8	50.9	1.944289	1.508316
111860	Sweeping	1	16	166	60.2	2.184642	1.667624
111928	Sweeping	1	16	159	56	2.215102	1.576847
112318	Sweeping	1	16	164.1	48.4	1.797332	1.476247
112442	Sweeping	1	16	167	59	2.115529	1.653596
112582	Sweeping	1	16	166.2	76.4	2.765867	1.896558

113000	Sweeping	1	16	160.2	99.5	3.87702	2.154457
113067	Sweeping	1	16	159.8	72	2.819544	1.808638
113135	Sweeping	1	16	165.6	61.1	2.228027	1.679379
113206	Sweeping	1	16	170.9	67.7	2.317953	1.796925
113289	Sweeping	1	16	172.7	63.7	2.135771	1.746261
100016	Sweeping	0	17	174.4	58.3	1.916795	1.671498
100724	Sweeping	0	17	178.3	72.6	2.283673	1.897354
111802	Sweeping	0	17	181.1	71.5	2.180063	1.893498
112346	Sweeping	0	17	173.3	85.6	2.850209	2.049861
112476	Sweeping	0	17	172.6	69.5	2.332938	1.829628
112691	Sweeping	0	17	171.8	64.1	2.171759	1.748525
113017	Sweeping	0	17	170.5	72.7	2.500838	1.865387
113023	Sweeping	0	17	172.8	64.3	2.153394	1.755491
113081	Sweeping	0	17	173	82.2	2.7465	2.004287
113095	Sweeping	0	17	183.4	94.3	2.80358	2.208433
100385	Sweeping	1	17	164.7	59.9	2.208206	1.657974
112972	Sweeping	1	17	168.1	48.4	1.712813	1.490408
112987	Sweeping	1	17	163.6	51.4	1.920421	1.52293
112993	Sweeping	1	17	171.5	69.2	2.352761	1.820756
113079	Sweeping	1	17	162.9	72.8	2.7434	1.833328
113103	Sweeping	1	17	155.7	49.1	2.02537	1.457025
113227	Sweeping	1	17	170.9	62.3	2.133064	1.718363
113455	Sweeping	1	17	171.5	71.6	2.43436	1.854449
113463	Sweeping	1	17	159.5	62.2	2.444945	1.670533
113494	Sweeping	1	17	161	56.4	2.175842	1.590757
113517	Sweeping	1	17	164.1	53.9	2.001574	1.56422
101059	Sweeping	0	18	181.8	87	2.632276	2.107447
101534	Sweeping	0	18	167.7	53.1	1.888115	1.565096
111642	Sweeping	0	18	184	86.4	2.551985	2.109654
111721	Sweeping	0	18	176.6	61.4	1.968734	1.727286
112299	Sweeping	0	18	164.1	61	2.265232	1.671859
112964	Sweeping	0	18	178.7	71.6	2.242146	1.884928
113157	Sweeping	0	18	170.4	62.5	2.152488	1.719329
113297	Sweeping	0	18	169.5	54.9	1.910878	1.600166
113305	Sweeping	0	18	183.5	72	2.138259	1.910553
113338	Sweeping	0	18	182.5	67.8	2.035654	1.845781
113353	Sweeping	0	18	163.5	57.7	2.158442	1.620239
113375	Sweeping	0	18	168.7	88.4	3.106148	2.06353
113380	Sweeping	0	18	179.8	69.4	2.146743	1.85807
100117	Sweeping	1	18	171.8	70.1	2.375044	1.834724
100549	Sweeping	1	18	164.9	52.6	1.934392	1.546795
111745	Sweeping	1	18	164.6	62	2.2884	1.688579
112498	Sweeping	1	18	159.5	62.1	2.441014	1.669088
112503	Sweeping	1	18	158.2	58.6	2.341449	1.612579
113251	Sweeping	1	18	163	46.8	1.761451	1.445938
113312	Sweeping	1	18	158.6	55.8	2.218339	1.572245
113412	Sweeping	1	18	161.5	55.4	2.12405	1.577464

113426	Sweeping	1	18	164.7	75.9	2.798044	1.883093
113435	Sweeping	1	18	168.2	65.2	2.3046	1.749847
113477	Sweeping	1	18	154.6	51.6	2.158891	1.492265
1143	TV	0	8	132.6	27.2	1.54697	0.995102
1181	TV	1	8	146.2	61.9	2.895982	1.609669
1148	TV	1	8	129.9	40.5	2.400141	1.222656
1151	TV	0	9	128.3	24.4	1.482301	0.926445
1305	TV	0	9	139.1	31.2	1.612502	1.091821
1312	TV	0	9	143.6	44.3	2.1483	1.335095
1308	TV	1	9	135.8	35.1	1.903301	1.1522
1307	TV	1	9	133.4	29.5	1.657717	1.041987
1128	TV	1	9	158.7	63.7	2.529214	1.68871
1166	TV	0	10	121	24.8	1.693873	0.91313
1137	TV	0	10	145.7	35	1.648728	1.182975
1309	TV	0	10	137.8	33.4	1.758928	1.128365
1127	TV	1	10	132.8	43.3	2.455228	1.278554
1163	TV	1	10	146.3	36.6	1.709986	1.213733
1255	TV	1	10	154.5	54	2.26223	1.528808
1327	TV	0	11	146.8	46.3	2.148468	1.379177
1318	TV	0	11	147.5	33.8	1.553577	1.166651
1330	TV	0	11	156.5	47	1.918974	1.426067
1325	TV	0	11	145.5	39.6	1.870549	1.263513
1202	TV	0	11	141.1	27.9	1.401361	1.033948
1232	TV	0	11	157.8	54.5	2.188681	1.54933
1188	TV	1	11	149.7	55.1	2.458714	1.526264
1217	TV	1	11	139.6	31.1	1.595841	1.091489
1250	TV	1	11	147.9	42.8	1.956624	1.326008
1323	TV	0	12	144.7	41.1	1.96293	1.286217
1115	TV	0	12	161	68.9	2.658076	1.771578
1322	TV	0	12	155.4	52.4	2.169848	1.507745
1233	TV	1	12	154.8	42.1	1.756872	1.338272
1216	TV	1	12	143.9	32.2	1.555014	1.125539
1214	TV	1	12	155.5	46.8	1.935464	1.41919
1117	TV	1	12	158.7	51.2	2.0329	1.501531
1195	TV	1	12	150.9	41.3	1.813726	1.311205
1213	TV	0	13	151.2	40.3	1.762794	1.295053
1275	TV	0	13	148.1	38.8	1.768975	1.258526
1272	TV	0	13	163.6	76.8	2.869423	1.890041
1302	TV	0	14	171.2	53.4	1.821939	1.582752
1269	TV	0	15	172.3	76.3	2.570123	1.922496
201019	TV	0	5	110	19.3	1.595041	0.768354
201031	TV	0	5	109	17	1.430856	0.715075
201054	TV	1	5	108.3	19.4	1.654035	0.76575
201039	TV	0	6	125.2	28.1	1.792659	0.989885
201002	TV	1	6	121.8	20.3	1.368363	0.822058
201023	TV	1	6	118.5	21.2	1.50973	0.832349
201025	TV	1	6	123.85	28	1.825433	0.983753

201030	TV	1	6	122.7667	27.8	1.844522	0.97656
201029	TV	0	7	125.9667	26.7	1.682674	0.965382
201037	TV	0	7	112.5	16.7	1.319506	0.71719
201038	TV	0	7	128.8	35.1	2.115804	1.128281
201043	TV	0	7	125.65	26.6	1.684832	0.962475
201049	TV	0	7	121.6	20.6	1.393157	0.828029
201005	TV	1	7	119	22.3	1.574748	0.856733
201012	TV	1	7	122.7	22.8	1.514418	0.877598
201017	TV	1	7	129	25.5	1.53236	0.950726
201018	TV	1	7	130.15	27.7	1.635277	0.997497
201020	TV	1	7	122	21.2	1.424348	0.842009
201021	TV	1	7	130.2	24.1	1.421658	0.92568
201026	TV	1	7	118.05	26.2	1.88005	0.931343
201046	TV	1	7	127.6	23.2	1.424907	0.899703
100601	TV	0	8	142.2	33.6	1.661652	1.146186
100974	TV	0	8	120.2	23.2	1.605754	0.878646
100989	TV	0	8	130.4	29.3	1.723108	1.028863
101033	TV	0	8	128.3	28.1	1.707077	0.999529
101419	TV	0	8	133.4	29.8	1.674575	1.047673
111695	TV	0	8	126	22.9	1.442429	0.888969
111777	TV	0	8	125.1	25.7	1.642171	0.943181
112125	TV	0	8	125	23.6	1.5104	0.900632
112198	TV	0	8	127.2	26.5	1.637841	0.965207
113527	TV	0	8	142.5	42.1	2.073253	1.295065
113546	TV	0	8	134.6	31	1.711085	1.07396
201001	TV	0	8	136.8	31.2	1.66718	1.084629
201035	TV	0	8	135.5	34	1.851827	1.131645
100424	TV	1	8	131.2	24.9	1.446544	0.944942
100841	TV	1	8	134.7	27.3	1.504622	1.003298
100912	TV	1	8	127.1	23.4	1.448521	0.90246
101300	TV	1	8	135.7	35.1	1.906108	1.151864
111581	TV	1	8	127.9	27.6	1.687206	0.9887
111665	TV	1	8	132	28.3	1.624197	1.014721
111703	TV	1	8	129.9	34.3	2.032712	1.11814
111734	TV	1	8	132.3	30.9	1.765382	1.064796
111797	TV	1	8	140.3	35.1	1.783166	1.167186
111901	TV	1	8	124.5	23.7	1.529008	0.90125
111932	TV	1	8	130	27.2	1.609467	0.987322
112484	TV	1	8	139	37.7	1.951245	1.208447
201006	TV	1	8	134.5	28.6	1.580962	1.02811
201013	TV	1	8	134.6	39.4	2.174734	1.221776
201024	TV	1	8	133.45	30.9	1.735087	1.068455
201041	TV	1	8	126.95	23.4	1.451946	0.902037
201044	TV	1	8	128.8	26.3	1.585346	0.966058
201051	TV	1	8	145	36	1.712247	1.198743
201052	TV	1	8	129.85	27.7	1.642842	0.996585
100193	TV	0	9	135.5	49.1	2.674256	1.378938

100339	TV	0	9	133	28.5	1.611171	1.021623
100505	TV	0	9	143.3	26.9	1.309966	1.020087
100816	TV	0	9	129.3	26.1	1.561146	0.963579
100923	TV	0	9	132.4	28.3	1.614397	1.015939
101230	TV	0	9	131.4	27.1	1.569562	0.989561
112110	TV	0	9	133.2	38.7	2.181235	1.205049
112335	TV	0	9	148.8	48.9	2.208528	1.427941
112772	TV	0	9	136.5	34.4	1.846261	1.14211
112895	TV	0	9	143.4	34.4	1.672862	1.164656
113182	TV	0	9	124.7	26.2	1.684878	0.951797
113347	TV	0	9	139.2	28.8	1.486326	1.046117
100234	TV	1	9	133.3	29.6	1.665833	1.043575
100556	TV	1	9	134.6	27.9	1.539976	1.014798
100589	TV	1	9	127.2	27.6	1.705827	0.986552
100953	TV	1	9	155.5	38.6	1.596344	1.279525
101071	TV	1	9	144.2	37	1.77939	1.213872
101104	TV	1	9	133.4	27.6	1.550949	1.00534
101373	TV	1	9	134.2	32	1.776826	1.091167
112564	TV	1	9	131.5	28.7	1.659703	1.020872
112780	TV	1	9	142.2	35.4	1.750669	1.17881
113034	TV	1	9	141.2	30.4	1.524769	1.083089
113147	TV	1	9	130	35	2.071006	1.1307
113199	TV	1	9	137.9	29.2	1.535516	1.049993
113212	TV	1	9	129.6	26.3	1.565834	0.968432
113274	TV	1	9	137	31.5	1.678299	1.090857
100160	TV	0	10	149.4	48.5	2.172904	1.423917
100356	TV	0	10	139.2	33.9	1.749529	1.141985
100471	TV	0	10	132.8	28.7	1.627368	1.024861
100787	TV	0	10	145	36.3	1.726516	1.204105
100804	TV	0	10	129.5	26.4	1.574216	0.970114
100861	TV	0	10	142.2	42.2	2.086956	1.295635
101082	TV	0	10	146.5	30.5	1.4211	1.100968
101150	TV	0	10	127.1	33.1	2.048976	1.087495
101265	TV	0	10	133	28.7	1.622477	1.025472
101428	TV	0	10	137.2	29.4	1.561849	1.051731
101459	TV	0	10	140.9	35.5	1.78816	1.176309
111564	TV	0	10	140.6	31	1.568162	1.092688
111621	TV	0	10	137.9	32	1.682757	1.102994
111683	TV	0	10	142.6	35.2	1.731026	1.176532
111977	TV	0	10	145.3	48.7	2.306735	1.411417
113530	TV	0	10	146.5	45.2	2.106023	1.360354
100186	TV	1	10	142	32.6	1.616743	1.127083
100258	TV	1	10	151.6	59.3	2.580217	1.595726
100360	TV	1	10	137.8	41.9	2.206559	1.274692
100397	TV	1	10	139.8	34	1.739661	1.145747
100445	TV	1	10	146.2	42.4	1.983678	1.313297
100851	TV	1	10	149.1	43.2	1.943249	1.336934

100946	TV	1	10	140.5	37	1.874343	1.201429
101331	TV	1	10	150.9	41.3	1.813726	1.311205
111839	TV	1	10	155.5	46	1.902379	1.406091
111911	TV	1	10	143	33.6	1.643112	1.148737
111950	TV	1	10	143.6	41.6	2.017365	1.290698
112576	TV	1	10	148.5	38.9	1.763992	1.261617
100438	TV	0	11	144.4	42.4	2.033441	1.306863
100594	TV	0	11	150.6	57.1	2.517597	1.559513
100791	TV	0	11	142.2	36.2	1.790232	1.193062
100890	TV	0	11	146.9	37.4	1.733118	1.229923
100990	TV	0	11	148.6	40.2	1.820491	1.284462
101112	TV	0	11	141.4	32.2	1.610486	1.117747
101220	TV	0	11	142.8	30.9	1.515312	1.097525
101396	TV	0	11	160.4	52.8	2.052226	1.533048
101546	TV	0	11	146.4	41.6	1.940936	1.300616
112001	TV	0	11	158.8	44.4	1.760686	1.391105
112203	TV	0	11	149.9	39	1.735647	1.268068
112228	TV	0	11	138.5	34.1	1.777685	1.143316
112438	TV	0	11	154.8	48.1	2.007258	1.437683
112524	TV	0	11	151.9	43.3	1.876603	1.348506
100265	TV	1	11	146.9	34.4	1.594097	1.175842
100319	TV	1	11	147	39.3	1.818687	1.263482
100417	TV	1	11	155.3	49	2.03167	1.453945
100649	TV	1	11	149	41.5	1.869285	1.30803
101012	TV	1	11	147.7	58	2.658686	1.56061
101040	TV	1	11	146.9	35.4	1.640437	1.194103
101060	TV	1	11	164.8	38.7	1.42494	1.311152
111270	TV	1	11	140.9	38.1	1.919124	1.221884
111570	TV	1	11	145.7	41.2	1.940788	1.29142
111636	TV	1	11	157	45	1.825632	1.394866
111785	TV	1	11	153.5	83.1	3.526828	1.922716
111948	TV	1	11	139	45	2.329072	1.329135
112852	TV	1	11	149.7	43.2	1.927703	1.339064
100518	TV	0	12	140.3	35.6	1.808567	1.176099
100529	TV	0	12	155.8	46.6	1.919778	1.417007
100692	TV	0	12	161.1	47	1.810951	1.442538
101173	TV	0	12	172	53.1	1.794889	1.580883
111891	TV	0	12	161	47.1	1.81706	1.443832
112062	TV	0	12	168.4	86.5	3.050225	2.038119
112075	TV	0	12	152.7	47.1	2.01996	1.413855
112425	TV	0	12	153.8	34.3	1.450045	1.195559
112702	TV	0	12	149	47.8	2.153056	1.411327
112748	TV	0	12	170.3	46	1.586093	1.457689
112762	TV	0	12	164.5	54.5	2.014024	1.57508
112845	TV	0	12	165.2	60.8	2.227837	1.673335
113051	TV	0	12	161	42.6	1.643455	1.367926
100126	TV	1	12	153.8	39	1.648739	1.281045

100179	TV	1	12	169.1	61.3	2.143746	1.696339
100459	TV	1	12	162.5	54.4	2.060118	1.565913
100672	TV	1	12	162.2	53.9	2.048741	1.557015
100882	TV	1	12	157.4	45.6	1.840583	1.406256
101209	TV	1	12	156.7	54.2	2.2073	1.54046
101256	TV	1	12	154.1	45.9	1.932891	1.39942
112085	TV	1	12	142.2	39.2	1.938594	1.245257
112399	TV	1	12	155.8	54.7	2.253473	1.544556
112411	TV	1	12	166	60.7	2.202787	1.675059
112594	TV	1	12	152.6	46.6	2.001137	1.405398
112656	TV	1	12	152	31.2	1.350416	1.130888
100071	TV	0	13	160	43.1	1.683594	1.373142
100322	TV	0	13	166.7	55.3	1.990004	1.595854
100404	TV	0	13	172.2	47.2	1.591753	1.484533
100756	TV	0	13	170.1	58.6	2.025298	1.659613
100901	TV	0	13	173.1	41.8	1.395025	1.393509
100932	TV	0	13	151.8	42.4	1.840019	1.333011
101313	TV	0	13	151.3	35.9	1.568255	1.217302
101329	TV	0	13	165.5	48.3	1.763401	1.47958
101461	TV	0	13	153.9	40.2	1.697262	1.30243
101550	TV	0	13	170.9	52.1	1.783831	1.560826
111654	TV	0	13	171	68.5	2.342601	1.808733
111710	TV	0	13	155.9	42.7	1.756854	1.352286
111820	TV	0	13	167.5	59.1	2.106483	1.657065
111849	TV	0	13	154	35.8	1.509529	1.224029
112263	TV	0	13	165.5	51.3	1.872929	1.528315
100652	TV	1	13	167.4	70.1	2.501538	1.815951
100669	TV	1	13	164.2	69.4	2.574027	1.79241
100703	TV	1	13	173.4	63.1	2.098607	1.740184
101219	TV	1	13	152.4	44.5	1.915976	1.370262
112102	TV	1	13	167.8	61.8	2.194849	1.698562
112162	TV	1	13	153.6	56.4	2.390544	1.561362
112189	TV	1	13	166.6	55.4	1.995996	1.597025
112237	TV	1	13	152.5	41.3	1.775867	1.316699
112534	TV	1	13	163.3	79.8	2.992475	1.927992
112680	TV	1	13	148.3	39.1	1.777847	1.264426
112871	TV	1	13	163	48.4	1.821672	1.472317
100567	TV	0	14	144.5	35.5	1.700171	1.188132
101124	TV	0	14	166	48.2	1.749165	1.479701
101163	TV	0	14	150.1	40.6	1.802041	1.296471
101193	TV	0	14	178.9	60.3	1.884068	1.719371
101366	TV	0	14	156.5	43	1.755657	1.359456
101444	TV	0	14	165.3	53.7	1.965299	1.565611
111870	TV	0	14	173	68.6	2.292091	1.818515
111883	TV	0	14	180	61.7	1.904321	1.744961
111991	TV	0	14	158.8	46.9	1.859824	1.432696
112015	TV	0	14	171.9	57	1.928959	1.641924

112023	TV	0	14	163.4	50.5	1.891417	1.507799
112359	TV	0	14	173.7	83.7	2.774124	2.027116
100278	TV	1	14	168.6	53.4	1.878565	1.57318
100284	TV	1	14	168.1	54.5	1.928684	1.588654
100683	TV	1	14	152.5	46.2	1.986563	1.398534
111679	TV	1	14	161	47.7	1.840207	1.453695
111985	TV	1	14	154.3	40.5	1.701074	1.308995
112212	TV	1	14	159.1	49.2	1.94368	1.471164
112304	TV	1	14	175.8	108.9	3.523629	2.346494
112457	TV	1	14	166.9	59.1	2.121655	1.654709
112867	TV	1	14	151.4	50.9	2.220578	1.469112
112884	TV	1	14	162.6	50.6	1.913857	1.50647
112911	TV	1	14	165.5	69.4	2.533748	1.798022
113163	TV	1	14	156.5	56.9	2.323184	1.580466
100084	TV	0	15	179.5	63.5	1.97081	1.770203
100208	TV	0	15	175.1	57.9	1.888453	1.667967
101474	TV	0	15	172.2	69.1	2.3303	1.82228
111850	TV	0	15	157.5	50.3	2.027715	1.48281
112379	TV	0	15	184.7	70.8	2.075389	1.89826
112666	TV	0	15	167.4	54.8	1.955553	1.590718
112674	TV	0	15	177.4	87.8	2.789891	2.097379
112722	TV	0	15	179.5	64.1	1.989432	1.779179
112739	TV	0	15	174.1	57.2	1.887116	1.653334
112820	TV	0	15	163	55.3	2.081373	1.581718
112838	TV	0	15	179	67.3	2.100434	1.82439
112944	TV	0	15	170.8	62.5	2.142418	1.720928
112952	TV	0	15	173.4	55.4	1.842517	1.622553
100140	TV	1	15	166.1	80	2.899684	1.943644
100719	TV	1	15	172.6	71.7	2.406787	1.860551
101401	TV	1	15	159	67.3	2.662078	1.740687
111819	TV	1	15	165.6	62.5	2.279079	1.699965
112048	TV	1	15	162.6	65	2.458512	1.723667
112052	TV	1	15	160.4	92.8	3.606943	2.076207
112517	TV	1	15	161.1	44.1	1.699212	1.393966
112630	TV	1	15	163.6	88.9	3.321507	2.044765
112643	TV	1	15	168.8	55.2	1.937288	1.602233
112714	TV	1	15	174.4	60.5	1.989127	1.705129
113040	TV	1	15	158.8	73.7	2.922581	1.826928
100221	TV	0	16	177	69.5	2.218392	1.847976
100482	TV	0	16	179.2	65.5	2.039695	1.79878
100961	TV	0	16	174.5	70.8	2.325104	1.855991
101133	TV	0	16	170	53.1	1.83737	1.57357
101507	TV	0	16	173.9	76.1	2.516434	1.926831
112251	TV	0	16	173.4	57.2	1.902383	1.650695
112463	TV	0	16	167.3	62.1	2.218706	1.700978
112618	TV	0	16	173.6	65.6	2.176729	1.777742
112621	TV	0	16	179.7	80.1	2.480484	2.006581

112753	TV	0	16	175.3	75.1	2.443859	1.919264
112790	TV	0	16	176.6	76.6	2.456107	1.945475
113115	TV	0	16	177.3	90.1	2.866205	2.126275
100836	TV	1	16	174.3	64.3	2.11649	1.761515
101181	TV	1	16	161.8	50.9	1.944289	1.508316
111860	TV	1	16	166	60.2	2.184642	1.667624
111928	TV	1	16	159	56	2.215102	1.576847
112318	TV	1	16	164.1	48.4	1.797332	1.476247
112442	TV	1	16	167	59	2.115529	1.653596
112582	TV	1	16	166.2	76.4	2.765867	1.896558
113000	TV	1	16	160.2	99.5	3.87702	2.154457
113067	TV	1	16	159.8	72	2.819544	1.808638
113135	TV	1	16	165.6	61.1	2.228027	1.679379
113206	TV	1	16	170.9	67.7	2.317953	1.796925
113289	TV	1	16	172.7	63.7	2.135771	1.746261
100016	TV	0	17	174.4	58.3	1.916795	1.671498
100724	TV	0	17	178.3	72.6	2.283673	1.897354
111802	TV	0	17	181.1	71.5	2.180063	1.893498
112346	TV	0	17	173.3	85.6	2.850209	2.049861
112476	TV	0	17	172.6	69.5	2.332938	1.829628
112691	TV	0	17	171.8	64.1	2.171759	1.748525
113017	TV	0	17	170.5	72.7	2.500838	1.865387
113023	TV	0	17	172.8	64.3	2.153394	1.755491
113081	TV	0	17	173	82.2	2.7465	2.004287
113095	TV	0	17	183.4	94.3	2.80358	2.208433
113171	TV	0	17	169.2	61.6	2.151691	1.701197
100385	TV	1	17	164.7	59.9	2.208206	1.657974
112972	TV	1	17	168.1	48.4	1.712813	1.490408
112993	TV	1	17	171.5	69.2	2.352761	1.820756
113079	TV	1	17	162.9	72.8	2.7434	1.833328
113103	TV	1	17	155.7	49.1	2.02537	1.457025
113227	TV	1	17	170.9	62.3	2.133064	1.718363
113455	TV	1	17	171.5	71.6	2.43436	1.854449
113463	TV	1	17	159.5	62.2	2.444945	1.670533
113494	TV	1	17	161	56.4	2.175842	1.590757
113517	TV	1	17	164.1	53.9	2.001574	1.56422
101059	TV	0	18	181.8	87	2.632276	2.107447
101534	TV	0	18	167.7	53.1	1.888115	1.565096
111642	TV	0	18	184	86.4	2.551985	2.109654
111721	TV	0	18	176.6	61.4	1.968734	1.727286
112299	TV	0	18	164.1	61	2.265232	1.671859
112964	TV	0	18	178.7	71.6	2.242146	1.884928
113157	TV	0	18	170.4	62.5	2.152488	1.719329
113297	TV	0	18	169.5	54.9	1.910878	1.600166
113305	TV	0	18	183.5	72	2.138259	1.910553
113338	TV	0	18	182.5	67.8	2.035654	1.845781
113353	TV	0	18	163.5	57.7	2.158442	1.620239

113375	TV	0	18	168.7	88.4	3.106148	2.06353
113380	TV	0	18	179.8	69.4	2.146743	1.85807
100117	TV	1	18	171.8	70.1	2.375044	1.834724
100215	TV	1	18	165.6	71.4	2.60362	1.826143
100549	TV	1	18	164.9	52.6	1.934392	1.546795
111745	TV	1	18	164.6	62	2.2884	1.688579
112498	TV	1	18	159.5	62.1	2.441014	1.669088
112503	TV	1	18	158.2	58.6	2.341449	1.612579
113251	TV	1	18	163	46.8	1.761451	1.445938
113312	TV	1	18	158.6	55.8	2.218339	1.572245
113412	TV	1	18	161.5	55.4	2.12405	1.577464
113426	TV	1	18	164.7	75.9	2.798044	1.883093
113435	TV	1	18	168.2	65.2	2.3046	1.749847
113477	TV	1	18	154.6	51.6	2.158891	1.492265
113482	TV	1	18	161.3	66.5	2.555953	1.739402
113509	TV	1	18	159.2	63.8	2.517298	1.692244
2222	Walk 2.5mph	0	7	120.6	20.3	13.9573	0.818838
2224	Walk 2.5mph	0	7	127.8	25.1	15.36781	0.93919
2277	Walk 2.5mph	0	5	115.5	20	14.99222	0.798513
2278	Walk 2.5mph	0	5	113.5	17.3	13.42933	0.733503
2279	Walk 2.5mph	0	6	127.6	25.6	15.72312	0.948617
2312	Walk 2.5mph	0	8	141	52.9	26.60832	1.45816
2522	Walk 2.5mph	0	9	136.9	66.3	35.37583	1.627274
2285	Walk 2.5mph	0	9	135	33.3	18.2716	1.117417
2287	Walk 2.5mph	0	9	139.6	34.1	17.4978	1.146907
2543	Walk 2.5mph	0	9	141.9	49.9	24.78197	1.416657
2514	Walk 2.5mph	0	9	137.2	31.4	16.68097	1.089623
2558	Walk 2.5mph	0	7	122.9	20.7	13.70461	0.833696
2560	Walk 2.5mph	0	7	117.9	22	15.82687	0.84739
2204	Walk 2.5mph	0	9	134.6	39.4	21.74734	1.221776
2231	Walk 2.5mph	0	7	121.3	21.6	14.68021	0.848578
2233	Walk 2.5mph	0	9	138.1	30	15.73019	1.06598
2272	Walk 2.5mph	0	8	133.5	30.2	16.94511	1.055526
2306	Walk 2.5mph	0	8	130.3	35	20.6148	1.131733
2310	Walk 2.5mph	0	6	114.7	33.8	25.69152	1.055948
2319	Walk 2.5mph	0	5	121.5	28.1	19.03504	0.978184
2321	Walk 2.5mph	0	9	133.8	38.3	21.39373	1.200471
2322	Walk 2.5mph	0	7	135.9	53.3	28.85947	1.442854
2324	Walk 2.5mph	0	8	133.9	52.2	29.1145	1.418402
2546	Walk 2.5mph	0	8	137.2	37.5	19.92155	1.198785
2551	Walk 2.5mph	0	9	133.4	35.1	19.72402	1.144085
2553	Walk 2.5mph	0	9	149	78.4	35.31372	1.841597
2221	Walk 2.5mph	0	8	140.7	35.2	17.78093	1.170293
2225	Walk 2.5mph	1	9	139.5	30	15.41604	1.07025
2282	Walk 2.5mph	1	5	107.1	15.8	13.77457	0.682695
2255	Walk 2.5mph	1	9	132.3	32.3	18.45367	1.090475
2261	Walk 2.5mph	1	7	120.8	26.2	17.95426	0.939884

2320	Walk 2.5mph	1	9	132.3	43	24.56681	1.271879
2241	Walk 2.5mph	1	9	144	60.7	29.27276	1.583267
2242	Walk 2.5mph	1	8	148.9	57.7	26.02474	1.561263
2245	Walk 2.5mph	1	9	140	35.7	18.21429	1.176875
2275	Walk 2.5mph	1	7	118	33.8	24.27463	1.067888
2276	Walk 2.5mph	1	8	136	49	26.49221	1.379439
2527	Walk 2.5mph	1	7	119.9	29.4	20.45074	0.997014
2528	Walk 2.5mph	1	6	122	30.8	20.69336	1.029333
2554	Walk 2.5mph	1	7	131.6	41.4	23.90499	1.243587
2536	Walk 2.5mph	0	12	178.5	118.7	37.25412	2.472688
2213	Walk 2.5mph	0	11	147.6	46.9	21.52782	1.391755
2216	Walk 2.5mph	0	10	151.7	37.8	16.42558	1.252847
2229	Walk 2.5mph	0	11	139.4	29.6	15.23232	1.06225
2280	Walk 2.5mph	0	11	146.2	40.4	18.90108	1.279609
2536	Walk 2.5mph	0	12	178.5	118.7	37.25412	2.472688
2220	Walk 2.5mph	0	12	164.6	55.5	20.48488	1.59094
2256	Walk 2.5mph	0	12	163.4	86.9	32.54735	2.018916
2260	Walk 2.5mph	0	11	163.9	71.4	26.5791	1.818688
2284	Walk 2.5mph	0	10	151.1	50.7	22.20644	1.464853
2286	Walk 2.5mph	0	10	148.9	63.3	28.55054	1.641008
2294	Walk 2.5mph	0	10	139.2	54.2	27.97183	1.469818
2296	Walk 2.5mph	0	12	161.6	75	28.71961	1.857011
2532	Walk 2.5mph	0	10	144	61.6	29.70679	1.595849
2552	Walk 2.5mph	0	11	154.8	57.1	23.82836	1.57661
2203	Walk 2.5mph	0	12	147.2	46	21.22962	1.375847
2215	Walk 2.5mph	0	12	156.5	59.9	24.45672	1.624748
2240	Walk 2.5mph	0	11	150.9	78	34.25438	1.845785
2243	Walk 2.5mph	0	10	148.3	43.6	19.82459	1.340715
2307	Walk 2.5mph	0	10	137.3	42.9	22.75706	1.289105
2316	Walk 2.5mph	0	10	144.6	49	23.43471	1.413378
2532	Walk 2.5mph	0	10	144	61.6	29.70679	1.595849
2547	Walk 2.5mph	0	12	172.45	94.7	31.8437	2.160104
2552	Walk 2.5mph	0	11	154.8	57.1	23.82836	1.57661
2520	Walk 2.5mph	1	12	154.2	44.8	18.84123	1.381638
2207	Walk 2.5mph	1	11	149.3	39.8	17.85515	1.279955
2217	Walk 2.5mph	1	12	159.1	54.6	21.57011	1.55591
2223	Walk 2.5mph	1	10	135.8	31.3	16.97246	1.083342
2270	Walk 2.5mph	1	11	153.1	40.4	17.23578	1.303216
2281	Walk 2.5mph	1	11	145.6	38	17.92507	1.236133
2301	Walk 2.5mph	1	11	140.4	48	24.35045	1.381555
2531	Walk 2.5mph	1	12	159.4	86.3	33.9652	1.991743
2534	Walk 2.5mph	1	11	145.7	51.5	24.25985	1.456081
2289	Walk 2.5mph	1	10	147.3	73.1	33.69084	1.765512
2534	Walk 2.5mph	1	11	145.7	51.5	24.25985	1.456081
2526	Walk 2.5mph	1	10	149.7	61.1	27.2645	1.613508
2530	Walk 2.5mph	1	11	142.3	49.7	24.54407	1.415178
2544	Walk 2.5mph	1	12	154.6	49.4	20.66845	1.457704

2545	Walk 2.5mph	1	11	156.9	56.6	22.99167	1.577575
2211	Walk 2.5mph	1	12	155.9	43.7	17.97998	1.369227
2230	Walk 2.5mph	1	10	147.9	53.2	24.32066	1.490565
2244	Walk 2.5mph	1	11	148.8	66.9	30.21484	1.690107
2271	Walk 2.5mph	1	10	144.3	40.8	19.5942	1.279754
2526	Walk 2.5mph	1	10	149.7	61.1	27.2645	1.613508
2529	Walk 2.5mph	1	12	156.6	66.9	27.27989	1.724686
2530	Walk 2.5mph	1	11	142.3	49.7	24.54407	1.415178
2544	Walk 2.5mph	1	12	154.6	49.4	20.66845	1.457704
2545	Walk 2.5mph	1	11	156.9	56.6	22.99167	1.577575
2512	Walk 2.5mph	0	13	175.2	52.2	17.006	1.577903
2218	Walk 2.5mph	0	14	172.8	70.2	23.50984	1.84036
2219	Walk 2.5mph	0	15	174.9	61	19.94115	1.714638
2227	Walk 2.5mph	0	13	145	35.7	16.97979	1.19336
2300	Walk 2.5mph	0	15	182.3	103.5	31.14347	2.31628
2540	Walk 2.5mph	0	15	169.4	92.8	32.3386	2.121626
2535	Walk 2.5mph	0	14	185.7	103.6	30.04249	2.334521
2550	Walk 2.5mph	0	14	170.6	54.2	18.62264	1.593241
2228	Walk 2.5mph	0	14	184.6	69.3	20.3362	1.876121
2254	Walk 2.5mph	0	13	154.7	41.8	17.46609	1.332793
2257	Walk 2.5mph	0	15	170.8	61.2	20.97856	1.701584
2262	Walk 2.5mph	0	15	175.1	61.7	20.12393	1.725974
2263	Walk 2.5mph	0	13	144.3	34.3	16.47257	1.165721
2535	Walk 2.5mph	0	14	185.7	103.6	30.04249	2.334521
2550	Walk 2.5mph	0	14	170.6	54.2	18.62264	1.593241
2559	Walk 2.5mph	0	14	154.3	47.6	19.99287	1.427794
2201	Walk 2.5mph	0	15	162.6	64.3	24.32035	1.713659
2202	Walk 2.5mph	0	13	148.9	53	23.90487	1.491528
2212	Walk 2.5mph	0	13	172.4	72.3	24.32561	1.868049
2246	Walk 2.5mph	0	13	152	44.4	19.21745	1.36718
2274	Walk 2.5mph	0	13	140.6	35.6	18.00858	1.177095
2293	Walk 2.5mph	0	14	178.2	89	28.02687	2.116518
2308	Walk 2.5mph	0	13	159.3	84.3	33.21972	1.966295
2309	Walk 2.5mph	0	15	164.4	102	37.73954	2.205916
2315	Walk 2.5mph	0	13	157	101.7	41.25928	2.16258
2323	Walk 2.5mph	0	13	161.3	96.5	37.09015	2.125031
2548	Walk 2.5mph	0	13	153.9	60.7	25.62781	1.625551
2511	Walk 2.5mph	1	15	161.8	62.4	23.83568	1.682944
2205	Walk 2.5mph	1	15	156.9	52.2	21.20433	1.510388
2206	Walk 2.5mph	1	15	162.5	51.7	19.5787	1.523624
2214	Walk 2.5mph	1	14	163.2	59.8	22.4523	1.650488
2258	Walk 2.5mph	1	14	169.4	115.1	40.10962	2.382143
2259	Walk 2.5mph	1	14	162.7	96.2	36.34126	2.128756
2288	Walk 2.5mph	1	13	159.8	80	31.32827	1.914079
2291	Walk 2.5mph	1	13	157.8	78.1	31.3644	1.880086
2557	Walk 2.5mph	1	13	166	53.7	19.48759	1.568236
2555	Walk 2.5mph	1	13	156.4	72.9	29.80259	1.805305

2232	Walk 2.5mph	1	13	153.7	42.1	17.82109	1.334494
2252	Walk 2.5mph	1	14	171.9	72.3	24.46733	1.865899
2273	Walk 2.5mph	1	14	147.9	46	21.02914	1.378437
2292	Walk 2.5mph	1	15	170.2	91.3	31.51749	2.107045
2298	Walk 2.5mph	1	15	153.5	107.4	45.58139	2.207129
2299	Walk 2.5mph	1	13	161.7	94.6	36.18021	2.104492
2525	Walk 2.5mph	1	14	159.8	72.3	28.31293	1.812687
2555	Walk 2.5mph	1	13	156.4	72.9	29.80259	1.805305
2502	Walk 2.5mph	1	14	161.1	51.6	19.88193	1.516827
2537	Walk 2.5mph	0	18	178.5	105.6	33.14267	2.321968
2537	Walk 2.5mph	0	18	178.5	105.6	33.14267	2.321968
2542	Walk 2.5mph	0	16	174.5	105.2	34.54816	2.296509
2264	Walk 2.5mph	0	17	194.2	77.3	20.49656	2.030043
2290	Walk 2.5mph	0	16	171	122.5	41.89323	2.472518
2542	Walk 2.5mph	0	16	174.5	105.2	34.54816	2.296509
2556	Walk 2.5mph	0	17	172.7	74.2	24.87822	1.895598
2253	Walk 2.5mph	0	16	175.4	57.2	18.59246	1.658216
2317	Walk 2.5mph	0	18	168.4	86.6	30.53752	2.039386
2318	Walk 2.5mph	0	17	173.4	112.8	37.51552	2.378325
2556	Walk 2.5mph	0	17	172.7	74.2	24.87822	1.895598
2208	Walk 2.5mph	0	17	177	69.8	22.27968	1.852262
2226	Walk 2.5mph	0	17	177.2	65.6	20.89183	1.792265
2503	Walk 2.5mph	1	18	163.3	52	19.49984	1.531351
2209	Walk 2.5mph	1	17	164.9	52.5	19.30714	1.545213
2303	Walk 2.5mph	1	18	153.9	49.1	20.73024	1.450325
2313	Walk 2.5mph	1	17	163.2	81.9	30.74989	1.954639
2549	Walk 2.5mph	1	16	164.7	47.9	17.65828	1.470151
2234	Walk 2.5mph	1	17	162.2	46.8	17.7887	1.443121
2305	Walk 2.5mph	1	17	166.5	90.9	32.78955	2.083841
2210	Walk 2.5mph	1	17	158.2	50.4	20.13806	1.487007
2249	Walk 2.5mph	1	17	160.2	74.4	28.98998	1.84264
2251	Walk 2.5mph	1	16	154.7	130.8	54.65465	2.461534
2311	Walk 2.5mph	1	16	166.7	80.9	29.11235	1.95817
2314	Walk 2.5mph	1	18	161.5	86.6	33.20266	2.005843
2524	Walk 2.5mph	1	17	157.2	74.2	30.02609	1.826238
3003	Walk 2.5mph	0	8	130.5	32.275	18.95157	1.084118
3005	Walk 2.5mph	0	7	127	25.067	15.54157	0.936193
3028	Walk 2.5mph	0	7	127.3	24.55	15.14937	0.926625
3043	Walk 2.5mph	0	7	129	27.475	16.51043	0.989643
3068	Walk 2.5mph	0	9	141.1	38.525	19.35034	1.229887
3079	Walk 2.5mph	0	5	114.5	21.033	16.04317	0.817612
3079	Walk 2.5mph	0	5	114.5	21.033	16.04317	0.817612
3079	Walk 2.5mph	0	5	114.5	21.033	16.04317	0.817612
3091	Walk 2.5mph	0	9	140.45	36.875	18.69341	1.199075
3103	Walk 2.5mph	0	6	129.5	27.225	16.23411	0.986302
3106	Walk 2.5mph	0	7	130.95	41.075	23.95337	1.235899
3108	Walk 2.5mph	0	7	123	27.1	17.91262	0.963983

3119	Walk 2.5mph	0	8	131.8	28.483	16.39664	1.017633
3135	Walk 2.5mph	0	7	120.45	23.475	16.1805	0.88496
4005	Walk 2.5mph	0	9	134.233	31.9	17.70403	1.089438
4014	Walk 2.5mph	0	7	118.7	20.2	14.33672	0.811541
4014	Walk 2.5mph	0	7	118.7	20.2	14.33672	0.811541
4029	Walk 2.5mph	0	7	128.3	27.05	16.43289	0.979267
4032	Walk 2.5mph	0	8	129.767	26.8	15.91499	0.97879
4042	Walk 2.5mph	0	9	132.967	31.65	17.90136	1.08077
4053	Walk 2.5mph	0	7	128.567	28.567	17.28246	1.00926
4053	Walk 2.5mph	0	7	128.567	28.567	17.28246	1.00926
4060	Walk 2.5mph	0	6	121.633	22.6	15.27586	0.870431
4061	Walk 2.5mph	0	8	135.933	31.55	17.07456	1.088409
4062	Walk 2.5mph	0	9	138.367	39.617	20.69267	1.238872
4063	Walk 2.5mph	0	7	141.033	55.683	27.99504	1.499066
4066	Walk 2.5mph	0	6	119.533	21.4	14.97746	0.839446
4066	Walk 2.5mph	0	6	119.533	21.4	14.97746	0.839446
4097	Walk 2.5mph	0	8	138.033	33.75	17.71364	1.135469
3001	Walk 2.5mph	1	8	125.5	24.05	15.2696	0.911269
3033	Walk 2.5mph	1	8	125.3	24.7	15.73239	0.923848
3048	Walk 2.5mph	1	9	134.367	32.483	17.99165	1.100536
3056	Walk 2.5mph	1	9	134.15	30.375	16.87854	1.060851
3061	Walk 2.5mph	1	9	138.167	34.2	17.91503	1.144026
3066	Walk 2.5mph	1	8	132.05	28.975	16.61677	1.02782
3080	Walk 2.5mph	1	7	116.45	21.35	15.74414	0.829752
3122	Walk 2.5mph	1	6	131.4	24.6	14.24769	0.939369
3133	Walk 2.5mph	1	6	124.55	23.8	15.34227	0.903437
4002	Walk 2.5mph	1	8	138.267	33.2	17.36605	1.126235
4003	Walk 2.5mph	1	8	128.367	21.517	13.05795	0.866047
4006	Walk 2.5mph	1	7	129.333	25.267	15.1055	0.947011
4006	Walk 2.5mph	1	7	129.333	25.267	15.1055	0.947011
4006	Walk 2.5mph	1	7	129.333	25.267	15.1055	0.947011
4020	Walk 2.5mph	1	9	137.2	30.8	16.36223	1.078376
4023	Walk 2.5mph	1	9	134.333	30.917	17.13294	1.071569
4027	Walk 2.5mph	1	9	139.867	41.717	21.32468	1.279223
4030	Walk 2.5mph	1	8	131.733	31.583	18.1997	1.075557
4034	Walk 2.5mph	1	8	131.233	31.333	18.19348	1.069356
4035	Walk 2.5mph	1	8	157.033	63.383	25.70342	1.677151
4047	Walk 2.5mph	1	6	127.633	24.3	14.91696	0.922493
4049	Walk 2.5mph	1	8	123.367	28.55	18.75893	0.992559
4049	Walk 2.5mph	1	8	123.367	28.55	18.75893	0.992559
4049	Walk 2.5mph	1	8	123.367	28.55	18.75893	0.992559
4064	Walk 2.5mph	1	9	133.233	39.933	22.49616	1.225668
4071	Walk 2.5mph	1	7	122	21.617	14.52365	0.850876
4077	Walk 2.5mph	1	6	110.867	18.7	15.21378	0.757768
4078	Walk 2.5mph	1	7	126.8	25.967	16.1504	0.953527
4087	Walk 2.5mph	1	6	119.033	31.617	22.31444	1.03379
4090	Walk 2.5mph	1	5	110.567	15.5	12.67887	0.68428

4090	Walk 2.5mph	1	5	110.567	15.5	12.67887	0.68428
4092	Walk 2.5mph	1	9	153	61.65	26.33602	1.635377
4104	Walk 2.5mph	1	9	133.133	32.633	18.41135	1.099238
3003	Walk 2.5mph	0	8	130.5	32.275	18.95157	1.084118
3002	Walk 2.5mph	0	11	141.55	31.225	15.58413	1.099878
3004	Walk 2.5mph	0	12	159.95	48.775	19.06465	1.467412
3022	Walk 2.5mph	0	10	144.8	32.875	15.67937	1.140983
3027	Walk 2.5mph	0	10	137.75	32.9	17.33855	1.119088
3034	Walk 2.5mph	0	11	142.8	42.525	20.85393	1.303165
3038	Walk 2.5mph	0	11	143.3	34.6	16.84937	1.167969
3086	Walk 2.5mph	0	11	144	38.4	18.51852	1.23768
3113	Walk 2.5mph	0	12	157.05	62.475	25.32972	1.664258
3126	Walk 2.5mph	0	10	144	31.3	15.09452	1.108815
4008	Walk 2.5mph	0	12	135.9	27.967	15.14283	1.019987
4019	Walk 2.5mph	0	11	144.633	34.1	16.3012	1.163123
4026	Walk 2.5mph	0	11	158.633	61.25	24.33991	1.653187
4028	Walk 2.5mph	0	10	148.667	50.267	22.74332	1.448759
4037	Walk 2.5mph	0	11	138.033	33.6	17.63492	1.132752
4038	Walk 2.5mph	0	12	154.6	37.633	15.74526	1.259285
4041	Walk 2.5mph	0	10	135.467	38.3	20.87045	1.206378
4046	Walk 2.5mph	0	12	153.633	36.45	15.44289	1.234763
4050	Walk 2.5mph	0	11	142.533	52.433	25.80916	1.457457
4052	Walk 2.5mph	0	10	140.867	42.75	21.54357	1.299828
4055	Walk 2.5mph	0	10	147.633	41.6	19.08651	1.304947
4069	Walk 2.5mph	0	10	133.95	26.45	14.74145	0.984194
4085	Walk 2.5mph	0	12	150.533	56.633	24.99229	1.552367
4108	Walk 2.5mph	0	12	135.633	28.783	15.64607	1.035079
4108	Walk 2.5mph	0	12	135.633	28.783	15.64607	1.035079
4108	Walk 2.5mph	0	12	135.633	28.783	15.64607	1.035079
3009	Walk 2.5mph	1	11	164.5	58.367	21.56928	1.63423
3060	Walk 2.5mph	1	11	157.75	63.875	25.66801	1.687183
3123	Walk 2.5mph	1	10	145.45	33.125	15.65772	1.147677
4016	Walk 2.5mph	1	11	148.7	42.067	19.02478	1.316558
4048	Walk 2.5mph	1	10	132.8	24.033	13.62736	0.931568
4081	Walk 2.5mph	1	10	142.733	35.6	17.47436	1.184141
4096	Walk 2.5mph	1	11	155.8	77.033	31.73525	1.856816
4101	Walk 2.5mph	1	11	137.633	28.2	14.88689	1.029708
4103	Walk 2.5mph	1	12	151.467	40.75	17.76199	1.303722
4106	Walk 2.5mph	1	12	157.067	46.983	19.04456	1.427835
4107	Walk 2.5mph	1	12	152.767	50.117	21.47464	1.462115
4110	Walk 2.5mph	1	12	152.467	41.917	18.03178	1.327128
3014	Walk 2.5mph	0	13	169.8	53.75	18.64245	1.583161
3021	Walk 2.5mph	0	14	159.75	48.225	18.8969	1.457767
3023	Walk 2.5mph	0	15	174.15	71.4	23.54243	1.86295
3087	Walk 2.5mph	0	13	167.5	48.275	17.2065	1.486229
3097	Walk 2.5mph	0	14	182.3	97.35	29.29292	2.241213
3115	Walk 2.5mph	0	13	152.25	38.525	16.61989	1.26753

3117	Walk 2.5mph	0	14	162.05	53.35	20.31591	1.547883
4007	Walk 2.5mph	0	14	163.933	51.367	19.11399	1.523632
4012	Walk 2.5mph	0	13	156.467	62.25	25.42693	1.658585
4012	Walk 2.5mph	0	13	156.467	62.25	25.42693	1.658585
4056	Walk 2.5mph	0	13	159.533	51.317	20.16323	1.506499
4091	Walk 2.5mph	0	13	168.5	66.033	23.2574	1.76308
3011	Walk 2.5mph	1	14	170.25	60.675	20.9332	1.691554
3016	Walk 2.5mph	1	15	159.4	61.633	24.25698	1.661913
3025	Walk 2.5mph	1	14	156.35	51.275	20.97538	1.493853
3098	Walk 2.5mph	1	14	159.75	44.825	17.56461	1.401561
3112	Walk 2.5mph	1	14	160.3	69.25	26.94963	1.773347
4015	Walk 2.5mph	1	14	154.667	45.9	19.18745	1.401459
4024	Walk 2.5mph	1	14	175.533	54.767	17.77466	1.62039
4036	Walk 2.5mph	1	14	157	72.7	29.4941	1.805378
4043	Walk 2.5mph	1	13	162.8	54.133	20.42459	1.562917
4051	Walk 2.5mph	1	14	158.967	58.9	23.3078	1.620117
4080	Walk 2.5mph	1	13	157.433	52.817	21.30994	1.522007
4082	Walk 2.5mph	1	14	168.133	47.517	16.80905	1.475837
4093	Walk 2.5mph	1	13	155.333	53.483	22.16605	1.524164
4093	Walk 2.5mph	1	13	155.333	53.483	22.16605	1.524164
1104	Walk 2.5mph	0	9	131	25.5	14.85927	0.956542
1317	Walk 2.5mph	1	9	139.2	29.9	15.43095	1.067419
1146	Walk 2.5mph	1	8	124.5	28.6	18.45132	0.997101
1114	Walk 2.5mph	0	12	162	62.8	23.92928	1.689564
1220	Walk 2.5mph	0	12	145.5	38.6	18.23313	1.246252
1329	Walk 2.5mph	0	11	143.1	33.4	16.3105	1.145372
1515	Walk 2.5mph	0	12	152.1	43.6	18.84639	1.354229
1535	Walk 2.5mph	0	11	148.9	51	23.0028	1.460989
1578	Walk 2.5mph	0	12	164.7	88.6	32.66228	2.04648
1511	Walk 2.5mph	0	12	163.4	72.1	27.00419	1.826043
1537	Walk 2.5mph	0	11	146.4	82.1	38.30549	1.874712
1512	Walk 2.5mph	0	11	142.8	36.9	18.09547	1.207428
1541	Walk 2.5mph	0	11	148.7	43.5	19.67285	1.340491
1614	Walk 2.5mph	0	12	170.7	78.1	26.80304	1.93957
1564	Walk 2.5mph	0	12	160.9	80.2	30.97864	1.92187
1548	Walk 2.5mph	0	12	161.2	80.1	30.82495	1.922
1108	Walk 2.5mph	1	12	162	63.7	24.27221	1.702544
1299	Walk 2.5mph	1	12	139.3	33.4	17.21251	1.133218
1229	Walk 2.5mph	1	12	151.3	46.6	20.35673	1.40064
1235	Walk 2.5mph	1	12	153.8	88.6	37.45597	1.99168
1249	Walk 2.5mph	1	11	153.1	57.9	24.70178	1.581514
1235	Walk 2.5mph	1	12	153.8	88.6	37.45597	1.99168
1516	Walk 2.5mph	1	12	146.1	48.8	22.86227	1.416054
1604	Walk 2.5mph	1	12	154.5	47.9	20.06682	1.433362
1519	Walk 2.5mph	1	12	154.9	56.6	23.58922	1.569572
1558	Walk 2.5mph	1	12	153.9	60.3	25.45893	1.619781
1502	Walk 2.5mph	1	12	152.6	47.2	20.26903	1.415101

1524	Walk 2.5mph	1	12	154.8	82.7	34.51148	1.924155
1574	Walk 2.5mph	1	12	163.4	85.2	31.91064	1.997578
1591	Walk 2.5mph	1	12	153.7	63.6	26.92213	1.666008
1593	Walk 2.5mph	1	12	158.2	61.8	24.69309	1.659355
1577	Walk 2.5mph	1	11	143.9	35.9	17.33696	1.193343
1268	Walk 2.5mph	0	14	169.4	57.5	20.03738	1.640102
1326	Walk 2.5mph	0	13	154.4	67	28.10478	1.716418
1274	Walk 2.5mph	0	14	175.1	111.2	36.26874	2.369267
1187	Walk 2.5mph	1	13	143.4	77.4	37.6394	1.801363
1187	Walk 2.5mph	1	13	143.4	77.4	37.6394	1.801363
1590	Walk 2.5mph	1	13	160.4	85.3	33.15433	1.98421
1569	Walk 2.5mph	1	13	151.6	44.7	19.44953	1.370708
1571	Walk 2.5mph	1	14	149.3	39.7	17.81029	1.278225
100339	Walk 2.5mph	0	9	133	28.5	16.11171	1.021623
100374	Walk 2.5mph	0	9	133	32.4	18.31647	1.094577
100601	Walk 2.5mph	0	8	142.2	33.6	16.61652	1.146186
100614	Walk 2.5mph	0	9	139.4	36.8	18.93748	1.194205
100769	Walk 2.5mph	0	8	127.8	29.1	17.81686	1.016929
100816	Walk 2.5mph	0	9	129.3	26.1	15.61146	0.963579
100989	Walk 2.5mph	0	8	130.4	29.3	17.23108	1.028863
101033	Walk 2.5mph	0	8	128.3	28.1	17.07077	0.999529
101140	Walk 2.5mph	0	9	137.1	34.6	18.40777	1.14767
101230	Walk 2.5mph	0	9	131.4	27.1	15.69562	0.989561
101240	Walk 2.5mph	0	8	132.2	30.7	17.5661	1.060766
101419	Walk 2.5mph	0	8	133.4	29.8	16.74575	1.047673
101526	Walk 2.5mph	0	9	121.7	25.3	17.08203	0.925098
111695	Walk 2.5mph	0	8	126	22.9	14.42429	0.888969
111777	Walk 2.5mph	0	8	125.1	25.7	16.42171	0.943181
111963	Walk 2.5mph	0	8	131.1	29.4	17.10574	1.03294
112090	Walk 2.5mph	0	8	126.7	23.8	14.82599	0.909587
112110	Walk 2.5mph	0	9	133.2	38.7	21.81235	1.205049
112125	Walk 2.5mph	0	8	125	23.6	15.104	0.900632
112198	Walk 2.5mph	0	8	127.2	26.5	16.37841	0.965207
112335	Walk 2.5mph	0	9	148.8	48.9	22.08528	1.427941
112772	Walk 2.5mph	0	9	136.5	34.4	18.46261	1.14211
112895	Walk 2.5mph	0	9	143.4	34.4	16.72862	1.164656
113182	Walk 2.5mph	0	9	124.7	26.2	16.84878	0.951797
113347	Walk 2.5mph	0	9	139.2	28.8	14.86326	1.046117
113527	Walk 2.5mph	0	8	142.5	42.1	20.73253	1.295065
113546	Walk 2.5mph	0	8	134.6	31	17.11085	1.07396
201001	Walk 2.5mph	0	8	136.8	31.2	16.6718	1.084629
201019	Walk 2.5mph	0	5	110	19.3	15.95041	0.768354
201029	Walk 2.5mph	0	7	125.9667	26.7	16.82674	0.965382
201031	Walk 2.5mph	0	5	109	17	14.30856	0.715075
201035	Walk 2.5mph	0	8	135.5	34	18.51827	1.131645
201037	Walk 2.5mph	0	7	112.5	16.7	13.19506	0.71719
201038	Walk 2.5mph	0	7	128.8	35.1	21.15804	1.128281

201039	Walk 2.5mph	0	6	125.2	28.1	17.92659	0.989885
201043	Walk 2.5mph	0	7	125.65	26.6	16.84832	0.962475
201049	Walk 2.5mph	0	7	121.6	20.6	13.93157	0.828029
100234	Walk 2.5mph	1	9	133.3	29.6	16.65833	1.043575
100424	Walk 2.5mph	1	8	131.2	24.9	14.46544	0.944942
100556	Walk 2.5mph	1	9	134.6	27.9	15.39976	1.014798
100576	Walk 2.5mph	1	9	128.1	27.6	16.81941	0.989313
100589	Walk 2.5mph	1	9	127.2	27.6	17.05827	0.986552
100841	Walk 2.5mph	1	8	134.7	27.3	15.04622	1.003298
100912	Walk 2.5mph	1	8	127.1	23.4	14.48521	0.90246
100953	Walk 2.5mph	1	9	155.5	38.6	15.96344	1.279525
101071	Walk 2.5mph	1	9	144.2	37	17.7939	1.213872
101104	Walk 2.5mph	1	9	133.4	27.6	15.50949	1.00534
101300	Walk 2.5mph	1	8	135.7	35.1	19.06108	1.151864
101357	Walk 2.5mph	1	9	147.1	42.6	19.68721	1.319831
101373	Walk 2.5mph	1	9	134.2	32	17.76826	1.091167
101437	Walk 2.5mph	1	8	130.3	27.5	16.19735	0.994071
111665	Walk 2.5mph	1	8	132	28.3	16.24197	1.014721
111703	Walk 2.5mph	1	8	129.9	34.3	20.32712	1.11814
111734	Walk 2.5mph	1	8	132.3	30.9	17.65382	1.064796
111797	Walk 2.5mph	1	8	140.3	35.1	17.83166	1.167186
111901	Walk 2.5mph	1	8	124.5	23.7	15.29008	0.90125
111932	Walk 2.5mph	1	8	130	27.2	16.09467	0.987322
112139	Walk 2.5mph	1	8	140.1	34.1	17.37313	1.148534
112484	Walk 2.5mph	1	8	139	37.7	19.51245	1.208447
112564	Walk 2.5mph	1	9	131.5	28.7	16.59703	1.020872
112780	Walk 2.5mph	1	9	142.2	35.4	17.50669	1.17881
113147	Walk 2.5mph	1	9	130	35	20.71006	1.1307
113199	Walk 2.5mph	1	9	137.9	29.2	15.35516	1.049993
113212	Walk 2.5mph	1	9	129.6	26.3	15.65834	0.968432
113274	Walk 2.5mph	1	9	137	31.5	16.78299	1.090857
201002	Walk 2.5mph	1	6	121.8	20.3	13.68363	0.822058
201005	Walk 2.5mph	1	7	119	22.3	15.74748	0.856733
201006	Walk 2.5mph	1	8	134.5	28.6	15.80962	1.02811
201012	Walk 2.5mph	1	7	122.7	22.8	15.14418	0.877598
201013	Walk 2.5mph	1	8	134.6	39.4	21.74734	1.221776
201017	Walk 2.5mph	1	7	129	25.5	15.3236	0.950726
201018	Walk 2.5mph	1	7	130.15	27.7	16.35277	0.997497
201020	Walk 2.5mph	1	7	122	21.2	14.24348	0.842009
201021	Walk 2.5mph	1	7	130.2	24.1	14.21658	0.92568
201023	Walk 2.5mph	1	6	118.5	21.2	15.0973	0.832349
201024	Walk 2.5mph	1	8	133.45	30.9	17.35087	1.068455
201025	Walk 2.5mph	1	6	123.85	28	18.25433	0.983753
201026	Walk 2.5mph	1	7	118.05	26.2	18.8005	0.931343
201030	Walk 2.5mph	1	6	122.7667	27.8	18.44522	0.97656
201041	Walk 2.5mph	1	8	126.95	23.4	14.51946	0.902037
201044	Walk 2.5mph	1	8	128.8	26.3	15.85346	0.966058

201046	Walk 2.5mph	1	7	127.6	23.2	14.24907	0.899703
201051	Walk 2.5mph	1	8	145	36	17.12247	1.198743
201052	Walk 2.5mph	1	8	129.85	27.7	16.42842	0.996585
201054	Walk 2.5mph	1	5	108.3	19.4	16.54035	0.76575
100042	Walk 2.5mph	0	10	143	49	23.96205	1.407158
100131	Walk 2.5mph	0	12	165.4	53.1	19.40991	1.556552
100160	Walk 2.5mph	0	10	149.4	48.5	21.72904	1.423917
100342	Walk 2.5mph	0	12	153	45	19.22338	1.380669
100356	Walk 2.5mph	0	10	139.2	33.9	17.49529	1.141985
100471	Walk 2.5mph	0	10	132.8	28.7	16.27368	1.024861
100518	Walk 2.5mph	0	12	140.3	35.6	18.08567	1.176099
100529	Walk 2.5mph	0	12	155.8	46.6	19.19778	1.417007
100594	Walk 2.5mph	0	11	150.6	57.1	25.17597	1.559513
100692	Walk 2.5mph	0	12	161.1	47	18.10951	1.442538
100787	Walk 2.5mph	0	10	145	36.3	17.26516	1.204105
100791	Walk 2.5mph	0	11	142.2	36.2	17.90232	1.193062
100804	Walk 2.5mph	0	10	129.5	26.4	15.74216	0.970114
100861	Walk 2.5mph	0	10	142.2	42.2	20.86956	1.295635
100890	Walk 2.5mph	0	11	146.9	37.4	17.33118	1.229923
100990	Walk 2.5mph	0	11	148.6	40.2	18.20491	1.284462
101000	Walk 2.5mph	0	11	150.4	36.8	16.26867	1.230706
101082	Walk 2.5mph	0	10	146.5	30.5	14.211	1.100968
101112	Walk 2.5mph	0	11	141.4	32.2	16.10486	1.117747
101150	Walk 2.5mph	0	10	127.1	33.1	20.48976	1.087495
101173	Walk 2.5mph	0	12	172	53.1	17.94889	1.580883
101220	Walk 2.5mph	0	11	142.8	30.9	15.15312	1.097525
101265	Walk 2.5mph	0	10	133	28.7	16.22477	1.025472
101342	Walk 2.5mph	0	11	164.3	51.1	18.92979	1.520715
101396	Walk 2.5mph	0	11	160.4	52.8	20.52226	1.533048
101428	Walk 2.5mph	0	10	137.2	29.4	15.61849	1.051731
101459	Walk 2.5mph	0	10	140.9	35.5	17.8816	1.176309
101512	Walk 2.5mph	0	10	130.7	34.5	20.19612	1.124375
101546	Walk 2.5mph	0	11	146.4	41.6	19.40936	1.300616
111621	Walk 2.5mph	0	10	137.9	32	16.82757	1.102994
111683	Walk 2.5mph	0	10	142.6	35.2	17.31026	1.176532
111753	Walk 2.5mph	0	12	153.7	56.6	23.959	1.564741
111891	Walk 2.5mph	0	12	161	47.1	18.1706	1.443832
112001	Walk 2.5mph	0	11	158.8	44.4	17.60686	1.391105
112075	Walk 2.5mph	0	12	152.7	47.1	20.1996	1.413855
112203	Walk 2.5mph	0	11	149.9	39	17.35647	1.268068
112228	Walk 2.5mph	0	11	138.5	34.1	17.77685	1.143316
112425	Walk 2.5mph	0	12	153.8	34.3	14.50045	1.195559
112438	Walk 2.5mph	0	11	154.8	48.1	20.07258	1.437683
112524	Walk 2.5mph	0	11	151.9	43.3	18.76603	1.348506
112702	Walk 2.5mph	0	12	149	47.8	21.53056	1.411327
112748	Walk 2.5mph	0	12	170.3	46	15.86093	1.457689
112762	Walk 2.5mph	0	12	164.5	54.5	20.14024	1.57508

112845	Walk 2.5mph	0	12	165.2	60.8	22.27837	1.673335
113051	Walk 2.5mph	0	12	161	42.6	16.43455	1.367926
113530	Walk 2.5mph	0	10	146.5	45.2	21.06023	1.360354
100021	Walk 2.5mph	1	11	144.9	32.5	15.47913	1.134276
100126	Walk 2.5mph	1	12	153.8	39	16.48739	1.281045
100179	Walk 2.5mph	1	12	169.1	61.3	21.43746	1.696339
100186	Walk 2.5mph	1	10	142	32.6	16.16743	1.127083
100258	Walk 2.5mph	1	10	151.6	59.3	25.80217	1.595726
100319	Walk 2.5mph	1	11	147	39.3	18.18687	1.263482
100360	Walk 2.5mph	1	10	137.8	41.9	22.06559	1.274692
100397	Walk 2.5mph	1	10	139.8	34	17.39661	1.145747
100417	Walk 2.5mph	1	11	155.3	49	20.3167	1.453945
100445	Walk 2.5mph	1	10	146.2	42.4	19.83678	1.313297
100459	Walk 2.5mph	1	12	162.5	54.4	20.60118	1.565913
100649	Walk 2.5mph	1	11	149	41.5	18.69285	1.30803
100672	Walk 2.5mph	1	12	162.2	53.9	20.48741	1.557015
100851	Walk 2.5mph	1	10	149.1	43.2	19.43249	1.336934
100882	Walk 2.5mph	1	12	157.4	45.6	18.40583	1.406256
100946	Walk 2.5mph	1	10	140.5	37	18.74343	1.201429
101012	Walk 2.5mph	1	11	147.7	58	26.58686	1.56061
101040	Walk 2.5mph	1	11	146.9	35.4	16.40437	1.194103
101060	Walk 2.5mph	1	11	164.8	38.7	14.2494	1.311152
101209	Walk 2.5mph	1	12	156.7	54.2	22.073	1.54046
101256	Walk 2.5mph	1	12	154.1	45.9	19.32891	1.39942
101331	Walk 2.5mph	1	10	150.9	41.3	18.13726	1.311205
111270	Walk 2.5mph	1	11	140.9	38.1	19.19124	1.221884
111570	Walk 2.5mph	1	11	145.7	41.2	19.40788	1.29142
111636	Walk 2.5mph	1	11	157	45	18.25632	1.394866
111785	Walk 2.5mph	1	11	153.5	83.1	35.26828	1.922716
111839	Walk 2.5mph	1	10	155.5	46	19.02379	1.406091
111911	Walk 2.5mph	1	10	143	33.6	16.43112	1.148737
111948	Walk 2.5mph	1	11	139	45	23.29072	1.329135
111950	Walk 2.5mph	1	10	143.6	41.6	20.17365	1.290698
112085	Walk 2.5mph	1	12	142.2	39.2	19.38594	1.245257
112399	Walk 2.5mph	1	12	155.8	54.7	22.53473	1.544556
112411	Walk 2.5mph	1	12	166	60.7	22.02787	1.675059
112576	Walk 2.5mph	1	10	148.5	38.9	17.63992	1.261617
112594	Walk 2.5mph	1	12	152.6	46.6	20.01137	1.405398
112656	Walk 2.5mph	1	12	152	31.2	13.50416	1.130888
113247	Walk 2.5mph	1	12	138.2	33.5	17.53996	1.13148
100874	Walk 2.5mph		13	170.4	52.7	18.14978	1.568645
100036	Walk 2.5mph	0	14	155.6	51.2	21.1471	1.489835
100071	Walk 2.5mph	0	13	160	43.1	16.83594	1.373142
100084	Walk 2.5mph	0	15	179.5	63.5	19.7081	1.770203
100102	Walk 2.5mph	0	14	179.4	96.7	30.04565	2.219003
100208	Walk 2.5mph	0	15	175.1	57.9	18.88453	1.667967
100302	Walk 2.5mph	0	14	163.6	53.1	19.83937	1.549815

100322	Walk 2.5mph	0	13	166.7	55.3	19.90004	1.595854
100404	Walk 2.5mph	0	13	172.2	47.2	15.91753	1.484533
100567	Walk 2.5mph	0	14	144.5	35.5	17.00171	1.188132
100623	Walk 2.5mph	0	15	160.9	63	24.33484	1.687891
100630	Walk 2.5mph	0	13	159.5	45	17.68851	1.403629
100756	Walk 2.5mph	0	13	170.1	58.6	20.25298	1.659613
100901	Walk 2.5mph	0	13	173.1	41.8	13.95025	1.393509
100932	Walk 2.5mph	0	13	151.8	42.4	18.40019	1.333011
101028	Walk 2.5mph	0	14	166.8	62.8	22.57187	1.709234
101124	Walk 2.5mph	0	14	166	48.2	17.49165	1.479701
101163	Walk 2.5mph	0	14	150.1	40.6	18.02041	1.296471
101193	Walk 2.5mph	0	14	178.9	60.3	18.84068	1.719371
101313	Walk 2.5mph	0	13	151.3	35.9	15.68255	1.217302
101329	Walk 2.5mph	0	13	165.5	48.3	17.63401	1.47958
101366	Walk 2.5mph	0	14	156.5	43	17.55657	1.359456
101444	Walk 2.5mph	0	14	165.3	53.7	19.65299	1.565611
101461	Walk 2.5mph	0	13	153.9	40.2	16.97262	1.30243
101474	Walk 2.5mph	0	15	172.2	69.1	23.303	1.82228
101487	Walk 2.5mph	0	15	175.3	81.7	26.58632	2.008207
101494	Walk 2.5mph	0	14	162.3	58.3	22.13255	1.624528
101550	Walk 2.5mph	0	13	170.9	52.1	17.83831	1.560826
111654	Walk 2.5mph	0	13	171	68.5	23.42601	1.808733
111710	Walk 2.5mph	0	13	155.9	42.7	17.56854	1.352286
111820	Walk 2.5mph	0	13	167.5	59.1	21.06483	1.657065
111849	Walk 2.5mph	0	13	154	35.8	15.09529	1.224029
111850	Walk 2.5mph	0	15	157.5	50.3	20.27715	1.48281
111883	Walk 2.5mph	0	14	180	61.7	19.04321	1.744961
111991	Walk 2.5mph	0	14	158.8	46.9	18.59824	1.432696
112015	Walk 2.5mph	0	14	171.9	57	19.28959	1.641924
112023	Walk 2.5mph	0	14	163.4	50.5	18.91417	1.507799
112263	Walk 2.5mph	0	13	165.5	51.3	18.72929	1.528315
112327	Walk 2.5mph	0	15	165.8	72.6	26.40995	1.843467
112359	Walk 2.5mph	0	14	173.7	83.7	27.74124	2.027116
112379	Walk 2.5mph	0	15	184.7	70.8	20.75389	1.89826
112558	Walk 2.5mph	0	15	161	45	17.36044	1.408847
112666	Walk 2.5mph	0	15	167.4	54.8	19.55553	1.590718
112674	Walk 2.5mph	0	15	177.4	87.8	27.89891	2.097379
112722	Walk 2.5mph	0	15	179.5	64.1	19.89432	1.779179
112739	Walk 2.5mph	0	15	174.1	57.2	18.87116	1.653334
112820	Walk 2.5mph	0	15	163	55.3	20.81373	1.581718
112838	Walk 2.5mph	0	15	179	67.3	21.00434	1.82439
112944	Walk 2.5mph	0	15	170.8	62.5	21.42418	1.720928
112952	Walk 2.5mph	0	15	173.4	55.4	18.42517	1.622553
100140	Walk 2.5mph	1	15	166.1	80	28.99684	1.943644
100278	Walk 2.5mph	1	14	168.6	53.4	18.78565	1.57318
100284	Walk 2.5mph	1	14	168.1	54.5	19.28684	1.588654
100652	Walk 2.5mph	1	13	167.4	70.1	25.01538	1.815951

100669	Walk 2.5mph	1	13	164.2	69.4	25.74027	1.79241
100683	Walk 2.5mph	1	14	152.5	46.2	19.86563	1.398534
100703	Walk 2.5mph	1	13	173.4	63.1	20.98607	1.740184
100774	Walk 2.5mph	1	15	154.7	53	22.146	1.514293
101219	Walk 2.5mph	1	13	152.4	44.5	19.15976	1.370262
101401	Walk 2.5mph	1	15	159	67.3	26.62078	1.740687
111679	Walk 2.5mph	1	14	161	47.7	18.40207	1.453695
111819	Walk 2.5mph	1	15	165.6	62.5	22.79079	1.699965
111985	Walk 2.5mph	1	14	154.3	40.5	17.01074	1.308995
112048	Walk 2.5mph	1	15	162.6	65	24.58512	1.723667
112052	Walk 2.5mph	1	15	160.4	92.8	36.06943	2.076207
112102	Walk 2.5mph	1	13	167.8	61.8	21.94849	1.698562
112173	Walk 2.5mph	1	13	159.4	52	20.4657	1.516747
112189	Walk 2.5mph	1	13	166.6	55.4	19.95996	1.597025
112212	Walk 2.5mph	1	14	159.1	49.2	19.4368	1.471164
112237	Walk 2.5mph	1	13	152.5	41.3	17.75867	1.316699
112246	Walk 2.5mph	1	13	168.6	66.2	23.28857	1.765892
112304	Walk 2.5mph	1	14	175.8	108.9	35.23629	2.346494
112401	Walk 2.5mph	1	15	158.7	52.2	20.72606	1.517233
112457	Walk 2.5mph	1	14	166.9	59.1	21.21655	1.654709
112517	Walk 2.5mph	1	15	161.1	44.1	16.99212	1.393966
112534	Walk 2.5mph	1	13	163.3	79.8	29.92475	1.927992
112630	Walk 2.5mph	1	15	163.6	88.9	33.21507	2.044765
112643	Walk 2.5mph	1	15	168.8	55.2	19.37288	1.602233
112680	Walk 2.5mph	1	13	148.3	39.1	17.77847	1.264426
112714	Walk 2.5mph	1	15	174.4	60.5	19.89127	1.705129
112867	Walk 2.5mph	1	14	151.4	50.9	22.20578	1.469112
112871	Walk 2.5mph	1	13	163	48.4	18.21672	1.472317
112884	Walk 2.5mph	1	14	162.6	50.6	19.13857	1.50647
112911	Walk 2.5mph	1	14	165.5	69.4	25.33748	1.798022
113040	Walk 2.5mph	1	15	158.8	73.7	29.22581	1.826928
113163	Walk 2.5mph	1	14	156.5	56.9	23.23184	1.580466
113267	Walk 2.5mph	1	15	161.2	53.5	20.58845	1.546993
100016	Walk 2.5mph	0	17	174.4	58.3	19.16795	1.671498
100221	Walk 2.5mph	0	16	177	69.5	22.18392	1.847976
100724	Walk 2.5mph	0	17	178.3	72.6	22.83673	1.897354
100961	Walk 2.5mph	0	16	174.5	70.8	23.25104	1.855991
101059	Walk 2.5mph	0	18	181.8	87	26.32276	2.107447
101133	Walk 2.5mph	0	16	170	53.1	18.3737	1.57357
101507	Walk 2.5mph	0	16	173.9	76.1	25.16434	1.926831
101534	Walk 2.5mph	0	18	167.7	53.1	18.88115	1.565096
111642	Walk 2.5mph	0	18	184	86.4	25.51985	2.109654
111721	Walk 2.5mph	0	18	176.6	61.4	19.68734	1.727286
111802	Walk 2.5mph	0	17	181.1	71.5	21.80063	1.893498
112033	Walk 2.5mph	0	18	178.1	71.6	22.57279	1.882416
112251	Walk 2.5mph	0	16	173.4	57.2	19.02383	1.650695
112299	Walk 2.5mph	0	18	164.1	61	22.65232	1.671859

112346	Walk 2.5mph	0	17	173.3	85.6	28.50209	2.049861
112387	Walk 2.5mph	0	16	165.7	57.2	20.83296	1.62124
112463	Walk 2.5mph	0	16	167.3	62.1	22.18706	1.700978
112476	Walk 2.5mph	0	17	172.6	69.5	23.32938	1.829628
112618	Walk 2.5mph	0	16	173.6	65.6	21.76729	1.777742
112621	Walk 2.5mph	0	16	179.7	80.1	24.80484	2.006581
112691	Walk 2.5mph	0	17	171.8	64.1	21.71759	1.748525
112753	Walk 2.5mph	0	16	175.3	75.1	24.43859	1.919264
112964	Walk 2.5mph	0	18	178.7	71.6	22.42146	1.884928
113017	Walk 2.5mph	0	17	170.5	72.7	25.00838	1.865387
113023	Walk 2.5mph	0	17	172.8	64.3	21.53394	1.755491
113081	Walk 2.5mph	0	17	173	82.2	27.465	2.004287
113095	Walk 2.5mph	0	17	183.4	94.3	28.0358	2.208433
113157	Walk 2.5mph	0	18	170.4	62.5	21.52488	1.719329
113171	Walk 2.5mph	0	17	169.2	61.6	21.51691	1.701197
113297	Walk 2.5mph	0	18	169.5	54.9	19.10878	1.600166
113305	Walk 2.5mph	0	18	183.5	72	21.38259	1.910553
113338	Walk 2.5mph	0	18	182.5	67.8	20.35654	1.845781
113353	Walk 2.5mph	0	18	163.5	57.7	21.58442	1.620239
113375	Walk 2.5mph	0	18	168.7	88.4	31.06148	2.06353
113380	Walk 2.5mph	0	18	179.8	69.4	21.46743	1.85807
100117	Walk 2.5mph	1	18	171.8	70.1	23.75044	1.834724
100154	Walk 2.5mph	1	16	177	66	21.06674	1.797329
100215	Walk 2.5mph	1	18	165.6	71.4	26.0362	1.826143
100531	Walk 2.5mph	1	16	159.6	51.6	20.25741	1.511213
100549	Walk 2.5mph	1	18	164.9	52.6	19.34392	1.546795
100836	Walk 2.5mph	1	16	174.3	64.3	21.1649	1.761515
101181	Walk 2.5mph	1	16	161.8	50.9	19.44289	1.508316
111745	Walk 2.5mph	1	18	164.6	62	22.884	1.688579
111860	Walk 2.5mph	1	16	166	60.2	21.84642	1.667624
111928	Walk 2.5mph	1	16	159	56	22.15102	1.576847
112442	Walk 2.5mph	1	16	167	59	21.15529	1.653596
112498	Walk 2.5mph	1	18	159.5	62.1	24.41014	1.669088
112503	Walk 2.5mph	1	18	158.2	58.6	23.41449	1.612579
112582	Walk 2.5mph	1	16	166.2	76.4	27.65867	1.896558
112927	Walk 2.5mph	1	18	171.5	64.7	21.99764	1.756091
112933	Walk 2.5mph	1	17	162.5	63.7	24.12308	1.704625
112972	Walk 2.5mph	1	17	168.1	48.4	17.12813	1.490408
112987	Walk 2.5mph	1	17	163.6	51.4	19.20421	1.52293
112993	Walk 2.5mph	1	17	171.5	69.2	23.52761	1.820756
113000	Walk 2.5mph	1	16	160.2	99.5	38.7702	2.154457
113067	Walk 2.5mph	1	16	159.8	72	28.19544	1.808638
113079	Walk 2.5mph	1	17	162.9	72.8	27.434	1.833328
113103	Walk 2.5mph	1	17	155.7	49.1	20.2537	1.457025
113122	Walk 2.5mph	1	16	170.8	78.1	26.77166	1.94002
113135	Walk 2.5mph	1	16	165.6	61.1	22.28027	1.679379
113206	Walk 2.5mph	1	16	170.9	67.7	23.17953	1.796925

113227	Walk 2.5mph	1	17	170.9	62.3	21.33064	1.718363
113251	Walk 2.5mph	1	18	163	46.8	17.61451	1.445938
113289	Walk 2.5mph	1	16	172.7	63.7	21.35771	1.746261
113312	Walk 2.5mph	1	18	158.6	55.8	22.18339	1.572245
113397	Walk 2.5mph	1	18	168.2	50.9	17.99143	1.531689
113406	Walk 2.5mph	1	17	159.3	55.6	21.91004	1.571954
113412	Walk 2.5mph	1	18	161.5	55.4	21.2405	1.577464
113426	Walk 2.5mph	1	18	164.7	75.9	27.98044	1.883093
113435	Walk 2.5mph	1	18	168.2	65.2	23.046	1.749847
113446	Walk 2.5mph	1	18	165.4	57.9	21.16448	1.630708
113455	Walk 2.5mph	1	17	171.5	71.6	24.3436	1.854449
113463	Walk 2.5mph	1	17	159.5	62.2	24.44945	1.670533
113477	Walk 2.5mph	1	18	154.6	51.6	21.58891	1.492265
113482	Walk 2.5mph	1	18	161.3	66.5	25.55953	1.739402
113494	Walk 2.5mph	1	17	161	56.4	21.75842	1.590757
113509	Walk 2.5mph	1	18	159.2	63.8	25.17298	1.692244
2543	Walk 3mph	0	9	141.9	49.9	24.78197	1.416657
2554	Walk 3mph	1	7	131.6	41.4	23.90499	1.243587
2222	Walk 3mph	0	7	120.6	20.3	13.9573	0.818838
2279	Walk 3mph	0	6	127.6	25.6	15.72312	0.948617
2507	Walk 3mph	0	7	117.25	21.7	15.78462	0.839314
2508	Walk 3mph	0	7	122.8	23	15.25215	0.882015
2515	Walk 3mph	0	8	138.9	36.4	18.86674	1.185517
2285	Walk 3mph	0	9	135	33.3	18.2716	1.117417
2546	Walk 3mph	0	8	137.2	37.5	19.92155	1.198785
2553	Walk 3mph	0	9	149	78.4	35.31372	1.841597
2558	Walk 3mph	0	7	122.9	20.7	13.70461	0.833696
2231	Walk 3mph	0	7	121.3	21.6	14.68021	0.848578
2272	Walk 3mph	0	8	133.5	30.2	16.94511	1.055526
2506	Walk 3mph	1	9	135.6	45.6	24.79964	1.32556
2509	Walk 3mph	1	7	120.4	28.7	19.79835	0.985802
2242	Walk 3mph	1	8	148.9	57.7	26.02474	1.561263
2276	Walk 3mph	1	8	136	49	26.49221	1.379439
2276	Walk 3mph	1	8	136	49	26.49221	1.379439
2280	Walk 3mph	0	11	146.2	40.4	18.90108	1.279609
2294	Walk 3mph	0	10	139.2	54.2	27.97183	1.469818
2296	Walk 3mph	0	12	161.6	75	28.71961	1.857011
2518	Walk 3mph	0	12	159.2	52.8	20.83281	1.528492
2523	Walk 3mph	0	11	161.3	84.9	32.63164	1.983596
2547	Walk 3mph	0	12	172.45	94.7	31.8437	2.160104
2520	Walk 3mph	1	12	154.2	44.8	18.84123	1.381638
2281	Walk 3mph	1	11	145.6	38	17.92507	1.236133
2289	Walk 3mph	1	10	147.3	73.1	33.69084	1.765512
2504	Walk 3mph	1	14	161.1	47.1	18.14804	1.444188
2540	Walk 3mph	0	15	169.4	92.8	32.3386	2.121626
2516	Walk 3mph	0	15	175	72.6	23.70612	1.883355
2517	Walk 3mph	0	15	166.3	61	22.05694	1.680708

2548	Walk 3mph	0	13	153.9	60.7	25.62781	1.625551
2541	Walk 3mph	1	15	165.2	93.1	34.11376	2.104266
2291	Walk 3mph	1	13	157.8	78.1	31.3644	1.880086
2525	Walk 3mph	1	14	159.8	72.3	28.31293	1.812687
2299	Walk 3mph	1	13	161.7	94.6	36.18021	2.104492
2501	Walk 3mph	0	17	188.2	76.6	21.62666	1.99516
2524	Walk 3mph	1	17	157.2	74.2	30.02609	1.826238
2290	Walk 3mph	0	16	171	122.5	41.89323	2.472518
2549	Walk 3mph	1	16	164.7	47.9	17.65828	1.470151
2251	Walk 3mph	1	16	154.7	130.8	54.65465	2.461534
4090	Walk 3mph	1	5	110.567	15.5	12.67887	0.68428
3005	Walk 3mph	0	7	127	25.067	15.54157	0.936193
3005	Walk 3mph	0	7	127	25.067	15.54157	0.936193
3049	Walk 3mph	0	7	122.35	23.2	15.49816	0.884842
3049	Walk 3mph	0	7	122.35	23.2	15.49816	0.884842
3049	Walk 3mph	0	7	122.35	23.2	15.49816	0.884842
3054	Walk 3mph	0	8	135.9	66.433	35.97037	1.624301
3064	Walk 3mph	0	7	129.2	32.1	19.23003	1.076671
3078	Walk 3mph	0	6	114.35	19.5	14.91291	0.784595
3078	Walk 3mph	0	6	114.35	19.5	14.91291	0.784595
3101	Walk 3mph	0	8	137	36.075	19.22052	1.173389
3104	Walk 3mph	0	8	132.167	29.467	16.86902	1.037534
3106	Walk 3mph	0	7	130.95	41.075	23.95337	1.235899
3108	Walk 3mph	0	7	123	27.1	17.91262	0.963983
3111	Walk 3mph	0	9	136.5	34.875	18.71755	1.150565
3127	Walk 3mph	0	9	147.5	35.7	16.40908	1.201474
3132	Walk 3mph	0	9	135.267	32.95	18.00826	1.111955
3135	Walk 3mph	0	7	120.45	23.475	16.1805	0.88496
4032	Walk 3mph	0	8	129.767	26.8	15.91499	0.97879
4032	Walk 3mph	0	8	129.767	26.8	15.91499	0.97879
4042	Walk 3mph	0	9	132.967	31.65	17.90136	1.08077
4042	Walk 3mph	0	9	132.967	31.65	17.90136	1.08077
4053	Walk 3mph	0	7	128.567	28.567	17.28246	1.00926
4060	Walk 3mph	0	6	121.633	22.6	15.27586	0.870431
4060	Walk 3mph	0	6	121.633	22.6	15.27586	0.870431
3006	Walk 3mph	1	9	134.75	29.375	16.17783	1.043768
3006	Walk 3mph	1	9	134.75	29.375	16.17783	1.043768
3039	Walk 3mph	1	8	126.55	22.65	14.14308	0.885264
3048	Walk 3mph	1	9	134.367	32.483	17.99165	1.100536
3080	Walk 3mph	1	7	116.45	21.35	15.74414	0.829752
3080	Walk 3mph	1	7	116.45	21.35	15.74414	0.829752
3118	Walk 3mph	1	9	146.95	42.425	19.64639	1.31638
3133	Walk 3mph	1	6	124.55	23.8	15.34227	0.903437
3133	Walk 3mph	1	6	124.55	23.8	15.34227	0.903437
4003	Walk 3mph	1	8	128.367	21.517	13.05795	0.866047
4003	Walk 3mph	1	8	128.367	21.517	13.05795	0.866047
4017	Walk 3mph	1	9	140.5	46.083	23.34469	1.351984

4047	Walk 3mph	1	6	127.633	24.3	14.91696	0.922493
4047	Walk 3mph	1	6	127.633	24.3	14.91696	0.922493
4054	Walk 3mph	1	5	120.667	19.967	13.7131	0.811765
4054	Walk 3mph	1	5	120.667	19.967	13.7131	0.811765
4071	Walk 3mph	1	7	122	21.617	14.52365	0.850876
4071	Walk 3mph	1	7	122	21.617	14.52365	0.850876
4073	Walk 3mph	1	6	121.4	23.7	16.08092	0.892286
4077	Walk 3mph	1	6	110.867	18.7	15.21378	0.757768
4087	Walk 3mph	1	6	119.033	31.617	22.31444	1.03379
4087	Walk 3mph	1	6	119.033	31.617	22.31444	1.03379
4102	Walk 3mph	1	9	136.8	33.217	17.74959	1.121792
3003	Walk 3mph	0	8	130.5	32.275	18.95157	1.084118
3042	Walk 3mph	0	6	120.9	23.3	15.94055	0.88271
3042	Walk 3mph	0	6	120.9	23.3	15.94055	0.88271
3064	Walk 3mph	0	7	129.2	32.1	19.23003	1.076671
3078	Walk 3mph	0	6	114.35	19.5	14.91291	0.784595
3127	Walk 3mph	0	9	147.5	35.7	16.40908	1.201474
3127	Walk 3mph	0	9	147.5	35.7	16.40908	1.201474
3135	Walk 3mph	0	7	120.45	23.475	16.1805	0.88496
4005	Walk 3mph	0	9	134.233	31.9	17.70403	1.089438
4005	Walk 3mph	0	9	134.233	31.9	17.70403	1.089438
4021	Walk 3mph	0	8	132.667	26.583	15.10352	0.983095
4021	Walk 3mph	0	8	132.667	26.583	15.10352	0.983095
4063	Walk 3mph	0	7	141.033	55.683	27.99504	1.499066
4063	Walk 3mph	0	7	141.033	55.683	27.99504	1.499066
4084	Walk 3mph	0	8	136.667	27.417	14.67887	1.011404
4084	Walk 3mph	0	8	136.667	27.417	14.67887	1.011404
4097	Walk 3mph	0	8	138.033	33.75	17.71364	1.135469
4097	Walk 3mph	0	8	138.033	33.75	17.71364	1.135469
3006	Walk 3mph	1	9	134.75	29.375	16.17783	1.043768
3008	Walk 3mph	1	8	134.6	31.8	17.55242	1.088777
3033	Walk 3mph	1	8	125.3	24.7	15.73239	0.923848
3033	Walk 3mph	1	8	125.3	24.7	15.73239	0.923848
3048	Walk 3mph	1	9	134.367	32.483	17.99165	1.100536
3061	Walk 3mph	1	9	138.167	34.2	17.91503	1.144026
4023	Walk 3mph	1	9	134.333	30.917	17.13294	1.071569
4023	Walk 3mph	1	9	134.333	30.917	17.13294	1.071569
4030	Walk 3mph	1	8	131.733	31.583	18.1997	1.075557
4030	Walk 3mph	1	8	131.733	31.583	18.1997	1.075557
4033	Walk 3mph	1	8	132.467	32.033	18.25502	1.086161
4064	Walk 3mph	1	9	133.233	39.933	22.49616	1.225668
4064	Walk 3mph	1	9	133.233	39.933	22.49616	1.225668
4073	Walk 3mph	1	6	121.4	23.7	16.08092	0.892286
4075	Walk 3mph	1	6	130.567	28.617	16.78639	1.01641
4075	Walk 3mph	1	6	130.567	28.617	16.78639	1.01641
4077	Walk 3mph	1	6	110.867	18.7	15.21378	0.757768
4088	Walk 3mph	1	8	128.033	25.117	15.3223	0.940211

4088	Walk 3mph	1	8	128.033	25.117	15.3223	0.940211
4102	Walk 3mph	1	9	136.8	33.217	17.74959	1.121792
4102	Walk 3mph	1	9	136.8	33.217	17.74959	1.121792
3061	Walk 3mph	1	9	138.167	34.2	17.91503	1.144026
3027	Walk 3mph	0	10	137.75	32.9	17.33855	1.119088
3027	Walk 3mph	0	10	137.75	32.9	17.33855	1.119088
3070	Walk 3mph	0	10	140	35.7	18.21429	1.176875
3070	Walk 3mph	0	10	140	35.7	18.21429	1.176875
3070	Walk 3mph	0	10	140	35.7	18.21429	1.176875
3088	Walk 3mph	0	12	156.5	49.283	20.12188	1.462912
3095	Walk 3mph	0	12	160.9	78.967	30.50237	1.905923
3102	Walk 3mph	0	11	149.6	38.05	17.00167	1.250368
4013	Walk 3mph	0	10	149.8	45.617	20.3284	1.379214
4013	Walk 3mph	0	10	149.8	45.617	20.3284	1.379214
4031	Walk 3mph	0	12	154.733	52.733	22.02503	1.510313
4031	Walk 3mph	0	12	154.733	52.733	22.02503	1.510313
4039	Walk 3mph	0	12	155.967	40.733	16.74484	1.318644
4041	Walk 3mph	0	10	135.467	38.3	20.87045	1.206378
4041	Walk 3mph	0	10	135.467	38.3	20.87045	1.206378
4052	Walk 3mph	0	10	140.867	42.75	21.54357	1.299828
4052	Walk 3mph	0	10	140.867	42.75	21.54357	1.299828
3041	Walk 3mph	1	10	142.45	36.583	18.02828	1.20067
3114	Walk 3mph	1	10	140.55	27.5	13.92102	1.024362
3123	Walk 3mph	1	10	145.45	33.125	15.65772	1.147677
3123	Walk 3mph	1	10	145.45	33.125	15.65772	1.147677
4016	Walk 3mph	1	11	148.7	42.067	19.02478	1.316558
4044	Walk 3mph	1	11	144.333	35.9	17.23309	1.194765
4068	Walk 3mph	1	10	150.867	56.45	24.80136	1.551029
4068	Walk 3mph	1	10	150.867	56.45	24.80136	1.551029
4070	Walk 3mph	1	10	138.5	38.483	20.06178	1.220137
4076	Walk 3mph	1	12	156.433	49.467	20.21431	1.465598
4099	Walk 3mph	1	12	158.9	48.85	19.34714	1.464796
4100	Walk 3mph	1	10	138.3	34.45	18.01129	1.148954
4105	Walk 3mph	1	10	151.533	52.033	22.66024	1.487127
4105	Walk 3mph	1	10	151.533	52.033	22.66024	1.487127
4106	Walk 3mph	1	12	157.067	46.983	19.04456	1.427835
4106	Walk 3mph	1	12	157.067	46.983	19.04456	1.427835
3002	Walk 3mph	0	11	141.55	31.225	15.58413	1.099878
3002	Walk 3mph	0	11	141.55	31.225	15.58413	1.099878
3022	Walk 3mph	0	10	144.8	32.875	15.67937	1.140983
3022	Walk 3mph	0	10	144.8	32.875	15.67937	1.140983
3038	Walk 3mph	0	11	143.3	34.6	16.84937	1.167969
3038	Walk 3mph	0	11	143.3	34.6	16.84937	1.167969
3052	Walk 3mph	0	11	154.4	41.9	17.57598	1.333481
3095	Walk 3mph	0	12	160.9	78.967	30.50237	1.905923
3095	Walk 3mph	0	12	160.9	78.967	30.50237	1.905923
3125	Walk 3mph	0	10	153.5	41.817	17.74746	1.328976

3128	Walk 3mph	0	11	145.067	35.917	17.06722	1.197475
3134	Walk 3mph	0	12	164.25	76.775	28.45831	1.892683
3134	Walk 3mph	0	12	164.25	76.775	28.45831	1.892683
4008	Walk 3mph	0	12	135.9	27.967	15.14283	1.019987
4031	Walk 3mph	0	12	154.733	52.733	22.02503	1.510313
4037	Walk 3mph	0	11	138.033	33.6	17.63492	1.132752
4038	Walk 3mph	0	12	154.6	37.633	15.74526	1.259285
4038	Walk 3mph	0	12	154.6	37.633	15.74526	1.259285
4039	Walk 3mph	0	12	155.967	40.733	16.74484	1.318644
4069	Walk 3mph	0	10	133.95	26.45	14.74145	0.984194
4069	Walk 3mph	0	10	133.95	26.45	14.74145	0.984194
3017	Walk 3mph	1	11	133.9	29.533	16.472	1.044161
3041	Walk 3mph	1	10	142.45	36.583	18.02828	1.20067
3041	Walk 3mph	1	10	142.45	36.583	18.02828	1.20067
3065	Walk 3mph	1	12	152.95	33.95	14.51245	1.186373
3085	Walk 3mph	1	12	158	49.3	19.74844	1.468727
3094	Walk 3mph	1	12	168.7	51.575	18.12212	1.544396
3114	Walk 3mph	1	10	140.55	27.5	13.92102	1.024362
3120	Walk 3mph	1	11	157.667	43.3	17.41832	1.368573
4040	Walk 3mph	1	10	150.667	59.333	26.13726	1.592302
4040	Walk 3mph	1	10	150.667	59.333	26.13726	1.592302
4081	Walk 3mph	1	10	142.733	35.6	17.47436	1.184141
4083	Walk 3mph	1	11	164.633	61.1	22.54278	1.675485
4089	Walk 3mph	1	11	157.5	54.6	22.01058	1.549689
4089	Walk 3mph	1	11	157.5	54.6	22.01058	1.549689
4101	Walk 3mph	1	11	137.633	28.2	14.88689	1.029708
4101	Walk 3mph	1	11	137.633	28.2	14.88689	1.029708
4107	Walk 3mph	1	12	152.767	50.117	21.47464	1.462115
4107	Walk 3mph	1	12	152.767	50.117	21.47464	1.462115
4110	Walk 3mph	1	12	152.467	41.917	18.03178	1.327128
4110	Walk 3mph	1	12	152.467	41.917	18.03178	1.327128
3024	Walk 3mph	0	13	173.15	56.45	18.82864	1.638083
3020	Walk 3mph	0	13	147.5	33.7	15.4898	1.164793
3024	Walk 3mph	0	13	173.15	56.45	18.82864	1.638083
3047	Walk 3mph	0	14	174.3	77.85	25.625	1.952313
3096	Walk 3mph	0	14	170.567	106.5	36.60662	2.290931
3096	Walk 3mph	0	14	170.567	106.5	36.60662	2.290931
3110	Walk 3mph	0	13	169.75	64.033	22.22206	1.739245
3131	Walk 3mph	0	13	159.1	45.875	18.12323	1.416831
4045	Walk 3mph	0	14	164.7	56.733	20.91455	1.61024
4045	Walk 3mph	0	14	164.7	56.733	20.91455	1.61024
4057	Walk 3mph	0	13	158.267	47.45	18.94329	1.439788
3057	Walk 3mph	1	14	158.4	44.217	17.62296	1.386632
3093	Walk 3mph	1	15	166.2	53.925	19.52217	1.572516
4015	Walk 3mph	1	14	154.667	45.9	19.18745	1.401459
4015	Walk 3mph	1	14	154.667	45.9	19.18745	1.401459
4022	Walk 3mph	1	13	158.967	49.317	19.51564	1.472557

4051	Walk 3mph	1	14	158.967	58.9	23.3078	1.620117
4067	Walk 3mph	1	13	159.167	59.85	23.62425	1.634933
4095	Walk 3mph	1	14	167.4	86.733	30.95091	2.036257
4095	Walk 3mph	1	14	167.4	86.733	30.95091	2.036257
3035	Walk 3mph	0	13	165.6	47.9	17.46686	1.473331
3044	Walk 3mph	0	13	160.233	43.9	17.0986	1.387591
3055	Walk 3mph	0	14	184.6	71.3	20.92311	1.905049
3096	Walk 3mph	0	14	170.567	106.5	36.60662	2.290931
4057	Walk 3mph	0	13	158.267	47.45	18.94329	1.439788
3010	Walk 3mph	1	13	173.75	67.35	22.3094	1.803709
3011	Walk 3mph	1	14	170.25	60.675	20.9332	1.691554
3015	Walk 3mph	1	13	164.5	45	16.62956	1.420909
3069	Walk 3mph	1	13	163.25	49.675	18.63938	1.493958
4022	Walk 3mph	1	13	158.967	49.317	19.51564	1.472557
4022	Walk 3mph	1	13	158.967	49.317	19.51564	1.472557
4051	Walk 3mph	1	14	158.967	58.9	23.3078	1.620117
4094	Walk 3mph	1	13	167.167	79.983	28.62178	1.948361
3032	Walk 3mph	0	16	170.3	59.7	20.58472	1.677076
3084	Walk 3mph	1	16	165.65	54.125	19.72491	1.573581
4011	Walk 3mph	1	16	163.633	62.1	23.19262	1.6861
4058	Walk 3mph	1	16	165.733	68.533	24.95064	1.786903
4058	Walk 3mph	1	16	165.733	68.533	24.95064	1.786903
4011	Walk 3mph	1	16	163.633	62.1	23.19262	1.6861
4058	Walk 3mph	1	16	165.733	68.533	24.95064	1.786903
1314	Walk 3mph	0	9	134.2	30.9	17.15748	1.070831
1315	Walk 3mph	0	9	134.3	27.1	15.0251	0.998161
1140	Walk 3mph	0	9	124	28.1	18.27523	0.986114
1184	Walk 3mph	1	8	134.6	30.5	16.83487	1.064609
1317	Walk 3mph	1	9	139.2	29.9	15.43095	1.067419
1146	Walk 3mph	1	8	124.5	28.6	18.45132	0.997101
1310	Walk 3mph	1	9	139.9	36.1	18.44471	1.183613
1102	Walk 3mph	0	10	143	41.8	20.4411	1.291886
1169	Walk 3mph	0	10	146.1	57.4	26.89128	1.54522
1247	Walk 3mph	0	11	158.3	92.8	37.03277	2.065389
1113	Walk 3mph	0	11	144	34.5	16.63773	1.168407
1190	Walk 3mph	0	11	156.8	62.4	25.38005	1.662133
1547	Walk 3mph	0	12	156.1	55	22.57132	1.550288
1507	Walk 3mph	0	12	162.3	52.3	19.85476	1.53236
1536	Walk 3mph	0	12	153.7	49.5	20.95354	1.455917
1561	Walk 3mph	0	12	164.8	58.4	21.50297	1.635908
1598	Walk 3mph	0	12	161.7	59	22.56483	1.63259
1573	Walk 3mph	0	12	158.1	48.4	19.3634	1.454611
1122	Walk 3mph	1	11	147	52.5	24.29543	1.476409
1118	Walk 3mph	1	12	151	59.4	26.05149	1.594664
1106	Walk 3mph	1	11	154	46.1	19.43835	1.402335
1225	Walk 3mph	1	11	163	62.9	23.67421	1.695141
1204	Walk 3mph	1	11	150.1	55.1	24.45627	1.527879

1208	Walk 3mph	1	12	154.9	48.7	20.29673	1.447671
1286	Walk 3mph	1	10	163.6	99.4	37.13811	2.171293
1504	Walk 3mph	1	11	140.8	32.3	16.29286	1.117726
1508	Walk 3mph	1	11	166.6	42.6	15.34828	1.386592
1546	Walk 3mph	1	12	145.4	32.7	15.46746	1.139579
1247	Walk 3mph	0	11	158.3	92.8	37.03277	2.065389
1503	Walk 3mph	0	11	139.2	31.5	16.25669	1.097767
1540	Walk 3mph	0	11	163.5	63.6	23.79149	1.707332
1197	Walk 3mph	1	11	150.7	54.3	23.90966	1.518307
1299	Walk 3mph	1	12	139.3	33.4	17.21251	1.133218
1229	Walk 3mph	1	12	151.3	46.6	20.35673	1.40064
1225	Walk 3mph	1	11	163	62.9	23.67421	1.695141
1186	Walk 3mph	1	12	158.6	61.6	24.48919	1.658124
1568	Walk 3mph	1	12	165.6	52.5	19.14426	1.547809
1550	Walk 3mph	1	12	159.2	41.4	16.33481	1.341076
1254	Walk 3mph	0	13	156.7	64	26.06405	1.684493
1282	Walk 3mph	0	13	137.8	29.7	15.64077	1.05932
1566	Walk 3mph	0	13	169.8	62.1	21.53854	1.711009
1595	Walk 3mph	0	13	180.4	89.9	27.624	2.138378
1572	Walk 3mph	0	13	157.8	63.7	25.58147	1.684908
1600	Walk 3mph	0	13	187.5	80.2	22.81244	2.042034
1545	Walk 3mph	1	13	155.1	61.3	25.4822	1.639212
1570	Walk 3mph	1	13	162.4	47.4	17.97241	1.453752
2562	wii	0	5	104.7	15.5	1.413964	0.669649
2277	wii	0	5	115.5	20	1.499222	0.798513
2278	wii	0	5	113.5	17.3	1.342933	0.733503
2561	wii	0	5	121.7	27.9	1.883749	0.975069
2563	wii	0	5	120.2	21	1.453484	0.832806
2564	wii	0	5	113.7	20.6	1.593479	0.806272
2510	wii	1	5	105.2	18.7	1.689702	0.742171
2279	wii	0	6	127.6	25.6	1.572312	0.948617
2519	wii	1	6	125.8	48.5	3.064647	1.330104
2528	wii	1	6	122	30.8	2.069336	1.029333
2231	wii	0	7	121.3	21.6	1.468021	0.848578
2558	wii	0	7	122.9	20.7	1.370461	0.833696
2222	wii	0	7	120.6	20.3	1.39573	0.818838
2224	wii	0	7	127.8	25.1	1.536781	0.93919
2560	wii	0	7	117.9	22	1.582687	0.84739
2507	wii	0	7	117.25	21.7	1.578462	0.839314
2508	wii	0	7	122.8	23	1.525215	0.882015
2275	wii	1	7	118	33.8	2.427463	1.067888
2527	wii	1	7	119.9	29.4	2.045074	0.997014
2554	wii	1	7	131.6	41.4	2.390499	1.243587
2509	wii	1	7	120.4	28.7	1.979835	0.985802
2261	wii	1	7	120.8	26.2	1.795426	0.939884
2306	wii	0	8	130.3	35	2.06148	1.131733
2515	wii	0	8	138.9	36.4	1.886674	1.185517

2546	wii	0	8	137.2	37.5	1.992155	1.198785
2312	wii	0	8	141	52.9	2.660832	1.45816
2221	wii	0	8	140.7	35.2	1.778093	1.170293
2272	wii	0	8	133.5	30.2	1.694511	1.055526
2242	wii	1	8	148.9	57.7	2.602474	1.561263
2276	wii	1	8	136	49	2.649221	1.379439
2513	wii	1	8	142.1	28.9	1.431231	1.05667
2204	wii	0	9	134.6	39.4	2.174734	1.221776
2514	wii	0	9	137.2	31.4	1.668097	1.089623
2287	wii	0	9	139.6	34.1	1.74978	1.146907
2233	wii	0	9	138.1	30	1.573019	1.06598
2285	wii	0	9	135	33.3	1.82716	1.117417
2522	wii	0	9	136.9	66.3	3.537583	1.627274
2551	wii	0	9	133.4	35.1	1.972402	1.144085
2553	wii	0	9	149	78.4	3.531372	1.841597
2543	wii	0	9	141.9	49.9	2.478197	1.416657
2241	wii	1	9	144	60.7	2.927276	1.583267
2320	wii	1	9	132.3	43	2.456681	1.271879
2225	wii	1	9	139.5	30	1.541604	1.07025
2255	wii	1	9	132.3	32.3	1.845367	1.090475
2506	wii	1	9	135.6	45.6	2.479964	1.32556
2245	wii	1	9	140	35.7	1.821429	1.176875
2532	wii	0	10	144	61.6	2.970679	1.595849
2307	wii	0	10	137.3	42.9	2.275706	1.289105
2286	wii	0	10	148.9	63.3	2.855054	1.641008
2247	wii	0	10	140.1	46.69	2.378743	1.359994
2243	wii	0	10	148.3	43.6	1.982459	1.340715
2284	wii	0	10	151.1	50.7	2.220644	1.464853
2316	wii	0	10	144.6	49	2.343471	1.413378
2216	wii	0	10	151.7	37.8	1.642558	1.252847
2223	wii	1	10	135.8	31.3	1.697246	1.083342
2289	wii	1	10	147.3	73.1	3.369084	1.765512
2526	wii	1	10	149.7	61.1	2.72645	1.613508
2230	wii	1	10	147.9	53.2	2.432066	1.490565
2271	wii	1	10	144.3	40.8	1.95942	1.279754
2240	wii	0	11	150.9	78	3.425438	1.845785
2523	wii	0	11	161.3	84.9	3.263164	1.983596
2552	wii	0	11	154.8	57.1	2.382836	1.57661
2213	wii	0	11	147.6	46.9	2.152782	1.391755
2260	wii	0	11	163.9	71.4	2.65791	1.818688
2280	wii	0	11	146.2	40.4	1.890108	1.279609
2229	wii	0	11	139.4	29.6	1.523232	1.06225
2545	wii	1	11	156.9	56.6	2.299167	1.577575
2244	wii	1	11	148.8	66.9	3.021484	1.690107
2301	wii	1	11	140.4	48	2.435045	1.381555
2530	wii	1	11	142.3	49.7	2.454407	1.415178
2534	wii	1	11	145.7	51.5	2.425985	1.456081

2207	wii	1	11	149.3	39.8	1.785515	1.279955
2281	wii	1	11	145.6	38	1.792507	1.236133
2296	wii	0	12	161.6	75	2.871961	1.857011
2203	wii	0	12	147.2	46	2.122962	1.375847
2518	wii	0	12	159.2	52.8	2.083281	1.528492
2547	wii	0	12	172.45	94.7	3.18437	2.160104
2220	wii	0	12	164.6	55.5	2.048488	1.59094
2536	wii	0	12	178.5	118.7	3.725412	2.472688
2215	wii	0	12	156.5	59.9	2.445672	1.624748
2256	wii	0	12	163.4	86.9	3.254735	2.018916
2520	wii	1	12	154.2	44.8	1.884123	1.381638
2531	wii	1	12	159.4	86.3	3.39652	1.991743
2211	wii	1	12	155.9	43.7	1.797998	1.369227
2217	wii	1	12	159.1	54.6	2.157011	1.55591
2529	wii	1	12	156.6	66.9	2.727989	1.724686
2544	wii	1	12	154.6	49.4	2.066845	1.457704
2227	wii	0	13	145	35.7	1.697979	1.19336
2308	wii	0	13	159.3	84.3	3.321972	1.966295
2274	wii	0	13	140.6	35.6	1.800858	1.177095
2263	wii	0	13	144.3	34.3	1.647257	1.165721
2246	wii	0	13	152	44.4	1.921745	1.36718
2254	wii	0	13	154.7	41.8	1.746609	1.332793
2323	wii	0	13	161.3	96.5	3.709015	2.125031
2202	wii	0	13	148.9	53	2.390487	1.491528
2512	wii	0	13	175.2	52.2	1.7006	1.577903
2548	wii	0	13	153.9	60.7	2.562781	1.625551
2212	wii	0	13	172.4	72.3	2.432561	1.868049
2315	wii	0	13	157	101.7	4.125928	2.16258
2555	wii	1	13	156.4	72.9	2.980259	1.805305
2232	wii	1	13	153.7	42.1	1.782109	1.334494
2557	wii	1	13	166	53.7	1.948759	1.568236
2218	wii	0	14	172.8	70.2	2.350984	1.84036
2550	wii	0	14	170.6	54.2	1.862264	1.593241
2293	wii	0	14	178.2	89	2.802687	2.116518
2559	wii	0	14	154.3	47.6	1.999287	1.427794
2228	wii	0	14	184.6	69.3	2.03362	1.876121
2535	wii	0	14	185.7	103.6	3.004249	2.334521
2273	wii	1	14	147.9	46	2.102914	1.378437
2259	wii	1	14	162.7	96.2	3.634126	2.128756
2214	wii	1	14	163.2	59.8	2.24523	1.650488
2502	wii	1	14	161.1	51.6	1.988193	1.516827
2252	wii	1	14	171.9	72.3	2.446733	1.865899
2258	wii	1	14	169.4	115.1	4.010962	2.382143
2504	wii	1	14	161.1	47.1	1.814804	1.444188
2525	wii	1	14	159.8	72.3	2.831293	1.812687
2262	wii	0	15	175.1	61.7	2.012393	1.725974
2300	wii	0	15	182.3	103.5	3.114347	2.31628

2517	wii	0	15	166.3	61	2.205694	1.680708
2516	wii	0	15	175	72.6	2.370612	1.883355
2201	wii	0	15	162.6	64.3	2.432035	1.713659
2257	wii	0	15	170.8	61.2	2.097856	1.701584
2540	wii	0	15	169.4	92.8	3.23386	2.121626
2219	wii	0	15	174.9	61	1.994115	1.714638
2206	wii	1	15	162.5	51.7	1.95787	1.523624
2511	wii	1	15	161.8	62.4	2.383568	1.682944
2205	wii	1	15	156.9	52.2	2.120433	1.510388
2292	wii	1	15	170.2	91.3	3.151749	2.107045
2541	wii	1	15	165.2	93.1	3.411376	2.104266
2542	wii	0	16	174.5	105.2	3.454816	2.296509
2505	wii	0	16	178.2	70.6	2.223255	1.868647
2253	wii	0	16	175.4	57.2	1.859246	1.658216
2290	wii	0	16	171	122.5	4.189323	2.472518
2251	wii	1	16	154.7	130.8	5.465465	2.461534
2549	wii	1	16	164.7	47.9	1.765828	1.470151
2264	wii	0	17	194.2	77.3	2.049656	2.030043
2208	wii	0	17	177	69.8	2.227968	1.852262
2501	wii	0	17	188.2	76.6	2.162666	1.99516
2556	wii	0	17	172.7	74.2	2.487822	1.895598
2226	wii	0	17	177.2	65.6	2.089183	1.792265
2318	wii	0	17	173.4	112.8	3.751552	2.378325
2209	wii	1	17	164.9	52.5	1.930714	1.545213
2234	wii	1	17	162.2	46.8	1.77887	1.443121
2524	wii	1	17	157.2	74.2	3.002609	1.826238
2249	wii	1	17	160.2	74.4	2.898998	1.84264
2537	wii	0	18	178.5	105.6	3.314267	2.321968
2317	wii	0	18	168.4	86.6	3.053752	2.039386
2503	wii	1	18	163.3	52	1.949984	1.531351
2303	wii	1	18	153.9	49.1	2.073024	1.450325
1146	wii	1	8	124.5	28.6	1.845132	0.997101
1306	wii	1	8	135.9	26.5	1.434852	0.990855
1184	wii	1	8	134.6	30.5	1.683487	1.064609
1104	wii	0	9	131	25.5	1.485927	0.956542
1314	wii	0	9	134.2	30.9	1.715748	1.070831
1315	wii	0	9	134.3	27.1	1.50251	0.998161
1123	wii	0	9	128	23.5	1.434326	0.907065
1140	wii	0	9	124	28.1	1.827523	0.986114
1317	wii	1	9	139.2	29.9	1.543095	1.067419
1304	wii	1	9	136.5	31.9	1.712085	1.096694
1169	wii	0	10	146.1	57.4	2.689128	1.54522
1102	wii	0	10	143	41.8	2.04411	1.291886
1329	wii	0	11	143.1	33.4	1.63105	1.145372
1190	wii	0	11	156.8	62.4	2.538005	1.662133
1247	wii	0	11	158.3	92.8	3.703277	2.065389
1113	wii	0	11	144	34.5	1.663773	1.168407

1122	wii	1	11	147	52.5	2.429543	1.476409
1197	wii	1	11	150.7	54.3	2.390966	1.518307
1249	wii	1	11	153.1	57.9	2.470178	1.581514
1225	wii	1	11	163	62.9	2.367421	1.695141
1192	wii	1	11	156.9	48.1	1.953886	1.445383
1204	wii	1	11	150.1	55.1	2.445627	1.527879
1106	wii	1	11	154	46.1	1.943835	1.402335
1220	wii	0	12	145.5	38.6	1.823313	1.246252
1235	wii	1	12	153.8	88.6	3.745597	1.99168
1299	wii	1	12	139.3	33.4	1.721251	1.133218
1208	wii	1	12	154.9	48.7	2.029673	1.447671
1118	wii	1	12	151	59.4	2.605149	1.594664
1229	wii	1	12	151.3	46.6	2.035673	1.40064
1108	wii	1	12	162	63.7	2.427221	1.702544
1112	wii	0	13	164.4	57.1	2.112674	1.614666
1282	wii	0	13	137.8	29.7	1.564077	1.05932
1254	wii	0	13	156.7	64	2.606405	1.684493
1326	wii	0	13	154.4	67	2.810478	1.716418
1107	wii	1	13	164	57.2	2.12671	1.614626
1187	wii	1	13	143.4	77.4	3.76394	1.801363
1222	wii	1	13	160.5	47.8	1.855572	1.45354
1268	wii	0	14	169.4	57.5	2.003738	1.640102

0=male
1=female

REEmlmin	REEml_kgl	VO2ml	ml_kg	Allo75	chMET	adMETs	VO2net
128.558156	6.427908	283.333	14.16665	29.95882	2.203929	4.047614	7.738742
120.926099	6.989948	253.357	14.64491	29.86746	2.095139	4.184261	7.654965
151.454327	5.389834	376.75	13.40747	30.86907	2.487549	3.830707	8.01764
112.790795	7.138658	408.5	25.85443	51.54651	3.621749	7.38698	18.71577
116.49212	6.694949	337.25	19.38218	39.58585	2.895046	5.537767	12.68723
145.527747	5.684678	457.1	17.85547	40.16347	3.140982	5.101563	12.17079
133.742531	6.191784	436.75	20.21991	43.59058	3.265603	5.777116	14.02812
130.10554	6.40914	385.467	18.98852	40.30558	2.962725	5.425292	12.57938
144.196183	5.744868	618.545	24.64323	55.15895	4.289607	7.040922	18.89836
222.0751	4.166512	569.8	10.69043	28.88531	2.565799	3.054409	6.523919
157.636548	4.663803	299.563	8.862811	21.36981	1.90034	2.532232	4.199007
140.364054	5.357407	327.786	12.51092	28.30508	2.335256	3.574547	7.153509
171.487386	4.89964	593	16.94286	41.2101	3.45798	4.840816	12.04322
218.795812	4.191491	759.154	14.54318	39.09102	3.469692	4.155194	10.35169
221.718148	4.191269	907.4	17.15312	46.26015	4.092583	4.900891	12.96185
173.522638	4.92962	574.25	16.31392	39.7369	3.309366	4.66112	11.3843
158.887899	5.261189	545.75	18.07119	42.36318	3.434812	5.163198	12.81
220.870941	3.827919	684.455	11.86231	32.69362	3.098891	3.38923	8.034386
197.481586	4.030236	675.538	13.78649	36.47562	3.420764	3.938997	9.756253
184.074034	4.67193	771.385	19.5783	49.05113	4.190624	5.5938	14.90637
170.372392	4.996258	351.706	10.31396	24.92378	2.064337	2.946845	5.317701
159.003353	5.300112	541.923	18.0641	42.27627	3.408249	5.161171	12.76399
167.536458	5.031125	400.214	12.01844	28.87082	2.388817	3.43384	6.987314
180.966802	4.724982	554.923	14.48885	36.04406	3.066435	4.139672	9.763869
226.836815	3.737015	944	15.55189	43.40907	4.161582	4.443398	11.81488
182.51803	4.244605	599.125	13.93314	35.67928	3.282552	3.980897	9.688534
153.514327	5.117144	520.923	17.3641	40.63802	3.393318	4.961171	12.24696
157.314865	4.870429	452.1	13.9969	33.36819	2.873854	3.999115	9.126475
167.052601	4.679345	624.8	17.5014	42.77991	3.740139	5.0004	12.82206
194.389483	4.531223	752.5	17.54079	44.89144	3.871094	5.011655	13.00957
242.610713	3.832713	907.333	14.33385	40.43095	3.739872	4.095387	10.50114
203.471042	4.357915	728.182	15.5961	40.76825	3.578799	4.456029	11.23819
198.063103	4.542732	556.636	12.76688	32.80624	2.810397	3.64768	8.224149
214.610367	4.232946	700.571	13.81797	36.87196	3.264386	3.947991	9.585022
220.238457	4.06344	518.353	9.563708	25.94933	2.353599	2.732488	5.500268
209.538199	4.27629	482.067	9.838102	26.02917	2.300616	2.810886	5.561812
185.628677	4.910812	832.154	22.01466	54.58645	4.482896	6.289902	17.10384
151.749277	4.84822	585.818	18.71623	44.26948	3.860434	5.347494	13.86801
207.703998	2.841368	883.733	12.08937	35.34948	4.254771	3.454106	9.248003
184.986116	3.477183	1010.6	18.99624	51.30333	5.463113	5.427497	15.51906
168.265436	4.124153	692.727	16.9786	42.91084	4.11687	4.851029	12.85445
276.14743	3.540352	1111.786	14.25367	42.35944	4.02606	4.072476	10.71331
205.372949	4.378954	586.071	12.49618	32.70172	2.853691	3.570338	8.117229
263.736767	3.693792	1210.727	16.95696	49.29156	4.590664	4.844846	13.26317
190.445592	4.714	512.316	12.68109	31.97068	2.690091	3.623168	7.967089
164.791244	5.567272	696.462	23.52912	54.88184	4.226329	6.722606	17.96185

201.473544	3.011563	1169.364	17.47928	49.98965	5.804057	4.994081	14.46772
173.480707	4.294077	779.182	19.28668	48.62425	4.491462	5.510481	14.99261
174.107944	3.627249	807.235	16.8174	44.26588	4.636405	4.80497	13.19015
170.331474	4.279685	1079.444	27.12171	68.12207	6.337314	7.74906	22.84202
165.853508	4.364566	447.438	11.77468	29.23447	2.69779	3.364195	7.410118
271.41857	3.618914	1037	13.82667	40.68954	3.820667	3.950476	10.20775
203.266826	4.418844	719.375	15.63859	40.72743	3.539067	4.468168	11.21974
228.007621	4.108245	1270.3	22.88829	62.47217	5.571305	6.539511	18.78004
236.389308	3.946399	1420.7	23.71786	65.98303	6.010001	6.776532	19.77146
298.6018	3.436154	1525.333	17.55274	53.59198	5.108251	5.015068	14.11658
179.119387	4.098842	702.727	16.08071	41.34524	3.923232	4.594488	11.98187
193.840307	3.550189	822.2	15.05861	40.93389	4.241636	4.302459	11.50842
179.616222	5.031267	652.231	18.26978	44.6581	3.631248	5.219936	13.23851
291.956722	3.463306	1273.462	15.10631	45.77363	4.361818	4.316089	11.643
178.552764	5.015527	639.867	17.97379	43.90381	3.58363	5.135369	12.95827
176.325218	5.140677	671.267	19.57047	47.36142	3.806982	5.591562	14.42979
200.572107	4.51739	878.455	19.78502	51.07193	4.379747	5.652864	15.26763
195.221978	4.670382	444.727	10.6394	27.05274	2.278058	3.039829	5.96902
319.85469	3.314556	1520.846	15.76006	49.39577	4.754803	4.502875	12.44551
219.379063	4.139228	874.182	16.494	44.50358	3.984801	4.712571	12.35477
267.385403	3.698277	1447.909	20.0264	58.3966	5.415064	5.72183	16.32813
330.764425	3.252354	1173.071	11.53462	36.62972	3.546545	3.295606	8.282267
220.286218	2.820566	1098.438	14.06451	41.81068	4.986413	4.018431	11.24394
175.841764	4.176764	884	20.99762	53.48607	5.027247	5.999321	16.82086
223.783076	2.797288	1162.25	14.52813	43.44923	5.193646	4.150893	11.73084
241.960879	2.557726	913.875	9.660412	30.12787	3.776954	2.760118	7.102686
262.725026	3.742522	1248.333	17.78252	51.47278	4.751481	5.08072	14.04
306.156768	3.439964	1618.462	18.18497	55.85474	5.286383	5.195705	14.745
262.942193	3.79426	888	12.81385	36.97116	3.377168	3.661101	9.019593
176.62647	3.839706	820.364	17.834	46.44492	4.644627	5.095429	13.99429
244.463998	2.541206	1062.615	11.04589	34.59351	4.346714	3.15597	8.504688
202.524052	3.38669	867.5	14.50669	40.34072	4.283442	4.144768	11.12
222.652481	3.079564	1228.286	16.98874	49.53883	5.516606	4.853926	13.90918
270.732384	2.352149	1211.5	10.52563	34.476	4.474899	3.007323	8.173481
243.991273	3.954478	992.8	16.09076	45.09703	4.068998	4.597361	12.13628
339.642295	3.281568	1154.467	11.15427	35.57757	3.399067	3.186934	7.872702
332.850284	3.263238	943.909	9.25401	29.40898	2.835837	2.644003	5.990772
247.475153	3.848758	1100.067	17.10835	48.44633	4.445161	4.8881	13.25959
242.044659	3.954978	1091.429	17.83381	49.88063	4.509205	5.095373	13.87883
242.374337	3.97335	1312.571	21.51756	60.13474	5.41547	6.147874	17.54421
192.667655	3.726647	983.5	19.02321	51.01013	5.104645	5.435203	15.29656
251.530029	2.341993	933.063	8.687737	27.96776	3.709549	2.482211	6.345745
189.633834	3.632832	1196.7	22.92529	61.62152	6.310583	6.550082	19.29246
243.615445	2.668296	767.733	8.408905	25.99304	3.151413	2.402544	5.740608
233.898653	4.089137	946.462	16.54654	45.50469	4.046462	4.727582	12.4574
380.344896	3.104856	840.882	6.864343	22.83668	2.210841	1.961241	3.759487
279.473384	2.136647	1756.167	13.42635	45.40566	6.283843	3.836101	11.28971

229.28143	2.834134	965.364	11.93281	35.78736	4.210389	3.409373	9.098672
282.814355	3.658659	1583	20.47865	60.72203	5.597311	5.851044	16.82
262.622647	3.762502	1357.8	19.45272	56.22691	5.170156	5.557921	15.69022
253.187634	3.859568	1340.25	20.43064	58.14441	5.293505	5.837326	16.57107
358.923586	3.181947	1070.417	9.489512	30.92582	2.982298	2.711289	6.307566
195.145513	3.717057	516.625	9.840476	26.48841	2.647383	2.811565	6.123419
188.382969	3.737757	941.3	18.67659	49.76284	4.996736	5.336168	14.93883
186.784826	3.991129	684.167	14.61895	38.23646	3.662862	4.176844	10.62782
240.762966	2.648657	1106.235	12.1698	37.57721	4.594706	3.477086	9.521144
217.539297	2.923915	1158.364	15.56941	45.72623	5.324849	4.448402	12.64549
228.183522	2.786124	991.591	12.10734	36.42249	4.345585	3.459239	9.321215
232.543299	2.685257	1194.059	13.78821	42.06175	5.134781	3.939489	11.10295
184.098282	3.749456	602	12.26069	32.45528	3.269992	3.503055	8.511237
131.225032	6.239007	491.42	23.36424	50.03535	2.37401	6.675496	17.12523
125.652752	6.293021	394.92	19.77863	41.80946	1.900939	5.651038	13.48561
112.862778	7.28147	247.42	15.96258	31.67277	1.775784	4.560737	8.681111
138.309994	5.936051	342.42	14.69614	32.28808	2.160379	4.198896	8.760086
127.033029	6.514514	441.75	22.65385	47.60478	2.086975	6.472527	16.13933
136.510756	6.040299	352.67	15.60487	34.02414	2.393742	4.458534	9.564568
132.945081	6.212387	388.25	18.14252	39.02125	2.920379	5.183578	11.93014
150.22095	5.517758	442.5	16.25344	37.12679	2.198758	4.643841	10.73569
120.467547	6.442115	276	14.75936	30.69219	1.539062	4.21696	8.317243
152.726621	4.830522	390.67	12.35633	29.30013	1.836287	3.530379	7.525805
134.610211	5.679756	351.83	14.84515	32.75455	2.447513	4.241471	9.165392
135.553177	5.695512	695.25	29.21218	64.52207	3.554811	8.346339	23.51667
148.2506	5.180508	494.42	17.27714	39.96023	3.386438	4.936327	12.09663
137.42327	5.655279	427.42	17.5893	39.05262	2.691053	5.025514	11.93402
138.975887	5.649426	378.75	15.39634	34.28872	2.244444	4.398955	9.746915
138.721557	5.909332	506.25	21.5655	47.46908	2.074795	6.16157	15.65616
148.948917	5.49627	399.17	14.72952	33.60709	2.17733	4.208434	9.23325
143.991703	5.744273	422.67	16.86161	37.72894	2.382313	4.817603	11.11734
138.245848	5.958873	569.67	24.55474	53.8899	2.922584	7.01564	18.59587
188.107499	4.57961	864.17	21.03883	53.26182	4.049152	6.011095	16.45922
149.572809	5.529494	415.92	15.37597	35.06585	2.666154	4.393134	9.846477
129.561069	6.413914	504.17	24.95891	52.91313	2.664042	7.131117	18.545
229.293976	4.117845	622.83	11.18528	30.55467	2.520763	3.195795	7.067436
142.628229	5.809704	516.33	21.03177	46.8154	2.940711	6.009078	15.22207
153.73806	5.381666	586	20.51318	47.4241	3.630281	5.860908	15.13151
150.82938	5.489695	468.25	17.04277	39.01885	2.710564	4.869362	11.55307
163.440276	5.091597	590.83	18.40592	43.81106	2.781424	5.258834	13.31432
141.162708	5.436235	452.92	17.44214	39.3736	2.119717	4.983468	12.0059
127.963273	5.993596	463.42	21.70585	46.65803	2.319071	6.201673	15.71226
140.08276	5.544099	212.67	8.416907	18.87083	1.075449	2.404831	2.872808
129.838585	6.006318	428.67	19.83023	42.7589	2.252246	5.665779	13.82391
153.973089	5.405789	642.33	22.55135	52.09774	3.613671	6.443242	17.14556
164.102754	5.084516	530.83	16.4471	39.20178	3.049345	4.69917	11.36258
257.803164	3.880649	562.92	8.4735	24.19128	2.902847	2.421	4.59285

149.103028	5.563546	509.58	19.01418	43.26247	2.75702	5.432623	13.45063
151.770889	5.535649	687.25	25.06656	57.35877	4.142056	7.161876	19.53092
148.928487	5.602396	513.5	19.31686	43.8619	3.143942	5.519102	13.71446
175.372551	4.861332	730.58	20.2517	49.63214	3.355132	5.786199	15.39037
169.195547	5.013201	572.42	16.96059	40.87987	3.275651	4.845884	11.94739
156.702661	5.317903	563.25	19.1146	44.53477	2.90965	5.461315	13.7967
162.909392	5.163531	538.33	17.06276	40.43887	3.137487	4.875074	11.89923
137.841503	5.580628	652.33	26.41012	58.87686	4.109165	7.545749	20.82949
139.437492	5.551519	576	22.93268	51.33891	3.393225	6.552193	17.38116
136.355385	5.669663	497.67	20.69314	45.82528	2.551107	5.912326	15.02348
155.498682	4.923493	417.42	13.2166	31.33165	1.491691	3.776173	8.293111
146.475821	5.130502	304.92	10.68021	24.68774	2.169014	3.051489	5.549709
160.774817	4.842615	528.67	15.9238	38.22356	3.090192	4.549656	11.08118
149.426896	5.157097	238.83	8.242623	19.12367	1.371246	2.355035	3.085526
156.653677	4.926216	566.58	17.81698	42.30979	2.959879	5.090566	12.89076
131.032932	6.08974	494.58	22.98555	49.50514	3.75165	6.567299	16.89581
236.08338	3.724711	772.33	12.18513	34.38138	3.359125	3.481465	8.460417
133.293582	5.884926	490.33	21.64812	47.22667	2.48583	6.185178	15.7632
156.723468	4.892563	663.33	20.70771	49.2642	3.967997	5.916488	15.81514
154.797533	4.940399	407.58	13.00801	30.77594	2.548649	3.716574	8.067611
172.036285	4.93294	690.33	19.79441	48.10288	2.727715	5.655545	14.86147
185.204492	4.674874	513.5	12.96161	32.51841	2.517157	3.703316	8.286733
163.617813	5.129085	658.92	20.6558	49.08962	3.555388	5.901657	15.52671
162.756174	5.142375	640.08	20.2237	47.96826	2.313514	5.778199	15.08132
182.625586	4.740444	636.08	16.51084	41.13436	2.07476	4.717382	11.77039
149.254256	5.543334	431.5	16.026	36.50598	2.430986	4.578857	10.48266
175.857865	4.925991	805.83	22.57227	55.17499	3.695112	6.44922	17.64628
166.622573	5.056831	760.08	23.06768	55.26724	3.05253	6.590765	18.01085
178.043597	4.828301	749.67	20.33003	50.09808	2.415719	5.808581	15.50173
158.210113	4.870551	629.08	19.36644	46.23425	2.884629	5.533268	14.49589
181.195391	4.343442	918.5	22.0174	55.9557	2.630524	6.290687	17.67396
163.107795	4.769234	813.58	23.78889	57.52819	3.613181	6.796825	19.01966
175.50344	4.394948	536.58	13.43701	33.77812	2.158581	3.839145	9.042059
154.513865	4.997699	374.58	12.11566	28.5691	2.291009	3.461618	7.117965
158.286299	4.850498	662.58	20.30399	48.52836	4.132602	5.801139	15.45349
231.095685	3.748511	874.58	14.18621	39.75115	2.79794	4.053204	10.4377
153.196121	5.043494	1077.25	35.46502	83.25864	4.683696	10.13286	30.42153
154.882145	5.028641	472.58	15.34351	36.14617	2.87143	4.383859	10.31487
150.975432	5.139589	528.25	17.98298	41.86548	2.537589	5.137994	12.84339
147.623891	5.23489	534.42	18.95106	43.67127	2.793185	5.41459	13.71617
184.453728	4.34776	832.17	19.61509	50.06056	4.00409	5.60431	15.26733
191.621382	4.158179	768.75	16.68186	43.46399	3.037577	4.766245	12.52368
160.485403	4.831424	475	14.29991	34.32996	2.332204	4.085688	9.468483
171.920178	5.225537	530.08	16.11185	38.58731	2.572205	4.603387	10.88632
202.898097	4.447861	813.67	17.83699	46.35571	3.076257	5.096283	13.38913
156.648556	5.922441	492.58	18.62306	42.23354	2.303606	5.320875	12.70062
182.894255	5.000526	1096	29.96582	73.69233	3.245772	8.561664	24.9653

213.170411	4.240763	696.92	13.86436	36.91652	2.585974	3.961247	9.623602
193.425084	4.649641	677.08	16.27596	41.33519	3.268234	4.650275	11.62632
183.665118	4.795434	720.67	18.81645	46.80986	2.760342	5.376128	14.02102
169.501588	5.415386	662.42	21.16358	50.05819	2.33798	6.046737	15.74819
173.206348	5.268634	910.83	27.70586	66.34192	4.002241	7.915959	22.43722
178.013252	5.120179	518.25	14.90638	36.19628	2.912826	4.258965	9.786198
178.664054	5.004595	663.67	18.5902	45.44133	3.190721	5.311485	13.5856
194.730435	4.555098	907.58	21.22994	54.28537	3.183598	6.065698	16.67484
225.114198	3.794081	1065	17.94954	49.81698	3.826393	5.12844	14.15546
162.17521	4.433076	637.83	17.43515	42.87908	2.962105	4.981471	13.00207
157.020172	4.557915	800.58	23.2389	56.30057	3.913286	6.639685	18.68098
161.831819	4.205281	678.17	17.62259	43.89215	4.308029	5.035025	13.41731
160.096518	4.833102	701.42	21.17494	50.79971	3.869903	6.049984	16.34184
141.375579	5.882561	400.42	16.66126	36.89009	2.384588	4.760359	10.7787
150.402986	5.469199	649	23.6	54.04371	3.82327	6.742857	18.1308
185.975968	3.574193	552.58	10.6198	28.5224	2.827653	3.034228	7.045606
190.674529	3.37776	684.92	12.13322	33.25768	2.354324	3.466633	8.755456
161.21654	4.528554	709.42	19.92753	48.67612	3.155362	5.693579	15.39897
185.515638	4.831136	1048.92	27.31563	67.99766	4.216764	7.804464	22.48449
185.792642	4.882855	569.83	14.97582	37.19455	2.526962	4.278806	10.09297
216.887704	4.136473	802.5	15.30525	41.18523	3.739864	4.372928	11.16877
239.840428	3.915762	811.33	13.2462	37.0568	3.042449	3.78463	9.330442
180.118425	5.014852	801.08	22.30364	54.60103	3.191554	6.37247	17.28879
168.865864	5.408034	616.92	19.75725	46.70378	2.060383	5.644927	14.34921
194.591056	4.575921	682.83	16.05714	41.0043	2.86398	4.587755	11.48122
195.390398	4.663255	748.42	17.86205	45.44487	3.482319	5.103444	13.1988
175.937533	5.159458	637.08	18.6827	45.14693	2.866502	5.337914	13.52324
176.811435	5.110157	839.67	24.26792	58.85747	2.760529	6.933691	19.15776
173.552921	5.165266	464.92	13.8369	33.31377	1.696293	3.953401	8.671639
207.953064	4.305446	860.42	17.81408	46.96239	3.080299	5.089737	13.50863
148.471382	5.027304	556.75	18.85179	43.94703	2.932887	5.386227	13.82449
217.756525	2.826795	1334	17.31725	51.30364	4.98226	4.947787	14.49046
203.73784	3.189634	1140.17	17.85002	50.46281	4.739255	5.100006	14.66039
185.912936	3.556441	854.08	16.33821	43.9317	3.957189	4.66806	12.78177
192.807635	3.531275	921.75	16.88187	45.89006	3.440393	4.823391	13.35059
149.333039	5.295498	503.25	17.84574	41.12415	3.210526	5.098784	12.55025
179.79542	4.152319	959.08	22.14965	56.81839	3.67112	6.328472	17.99733
172.576349	4.102416	839.83	19.9641	50.84348	3.470372	5.70403	15.86169
204.958319	3.354473	928.67	15.19918	42.49428	4.310974	4.342623	11.84471
201.699296	3.455708	1043.08	17.87106	49.39603	3.684753	5.106016	14.41535
162.597531	4.529179	738.83	20.58022	50.376	3.41261	5.880064	16.05104
242.301959	3.878383	841	13.46138	37.84568	2.32964	3.84611	9.583002
275.926731	3.593966	1137.25	14.81276	43.84712	3.550245	4.232218	11.2188
160.441495	5.736815	561.25	20.06829	46.15003	2.980141	5.733799	14.33148
185.804266	4.93727	604.5	16.06303	39.78503	2.236818	4.589437	11.12576
193.056639	4.739563	614.92	15.09636	38.13808	3.129364	4.313245	10.3568
227.884273	4.023878	954.58	16.85554	46.23918	3.645801	4.81587	12.83167

182.95186	5.019255	758.17	20.80027	51.10854	3.233134	5.942936	15.78102
162.231137	5.636353	664.92	23.10114	53.50783	3.680301	6.600325	17.46478
280.232835	3.548733	1264.42	16.01201	47.73172	4.144552	4.574859	12.46327
219.887644	4.16983	623.92	11.83168	31.88358	2.116777	3.38048	7.66185
253.634278	3.845857	1095.17	16.60607	47.3228	3.535658	4.74459	12.76021
212.442665	4.310668	908.42	18.43273	48.83867	2.76679	5.266493	14.12206
211.953867	4.345543	1007.92	20.66468	54.61075	3.596503	5.904196	16.31914
186.159241	3.763302	625.75	12.64985	33.54781	2.815523	3.614242	8.886546
194.269713	3.868605	949	18.89798	50.30697	3.498618	5.399424	15.02938
165.895044	4.886452	626.58	18.45596	44.5499	3.046531	5.273133	13.56951
188.851085	3.671788	735.42	14.2986	38.29165	3.756935	4.085315	10.62681
186.976717	3.792631	1004.42	20.37363	53.98588	3.889332	5.821037	16.581
184.082111	3.939018	715.92	15.31937	40.05408	2.674637	4.376962	11.38035
187.035117	3.828764	947.08	19.38751	51.25524	3.079134	5.539289	15.55875
172.833108	4.241303	566.58	13.9038	35.12898	2.624027	3.972515	9.6625
183.684359	3.909592	712.42	15.16336	39.69909	2.625659	4.332388	11.25376
184.547821	3.68234	664.42	13.25738	35.27393	3.982617	3.787822	9.575038
174.833487	4.170945	806.58	19.24231	48.96151	3.432255	5.497803	15.07137
196.524127	3.810453	600.83	11.64964	31.21923	2.610375	3.328468	7.839183
195.381188	3.267244	572.92	9.580602	26.64208	2.475672	2.737315	6.313358
208.64483	4.397151	975.33	20.5549	53.94788	3.650597	5.872829	16.15775
205.251048	4.474137	789.08	17.20065	44.76504	3.256221	4.914473	12.72652
217.607937	4.240465	820.5	15.98885	42.79398	3.024885	4.568244	11.74839
244.552522	3.928555	953.17	15.31197	43.00964	3.701631	4.374848	11.38341
212.263891	4.396973	1064.75	22.05593	58.13744	3.817403	6.301694	17.65896
225.05075	4.186991	1175.08	21.86195	59.1948	4.526851	6.246272	17.67496
175.581306	5.210128	657.42	19.50801	47.00245	2.930593	5.573718	14.29788
231.778553	4.105909	977.25	17.31178	47.45235	2.988532	4.946223	13.20587
252.507493	3.823959	891.67	13.5034	38.49314	3.342718	3.858114	9.679441
211.056254	4.406185	1096.67	22.89499	60.23159	3.404962	6.541426	18.4888
187.368653	4.86356	625.33	16.2318	40.43917	2.739671	4.637656	11.36824
201.012197	4.578866	746.58	17.00638	43.77518	3.216908	4.858965	12.42751
292.779699	3.487549	948.08	11.29339	34.18452	2.579248	3.226683	7.80584
248.234535	3.876666	1148.58	17.93731	50.74092	3.41078	5.124947	14.06065
241.683449	3.882465	876	14.07229	39.52752	1.899187	4.020654	10.18982
218.099251	3.238296	949.17	14.0931	40.37298	3.717425	4.026599	10.8548
187.620576	3.804379	609.92	12.36734	32.77369	2.729926	3.533525	8.562958
190.80057	3.840978	830.25	16.71364	44.37164	3.044889	4.775325	12.87266
186.17938	4.13732	1160.33	25.78511	66.78396	4.021941	7.367175	21.64779
190.112104	3.554627	611.75	11.43821	30.9323	3.256934	3.268061	7.883587
199.979126	3.341339	1138.58	19.02389	52.91337	4.09679	5.435398	15.68255
228.518147	2.857084	1301.08	16.26696	48.64696	4.942374	4.647702	13.40987
195.686145	3.614914	978.5	18.07585	49.03027	4.374749	5.164529	14.46094
190.694219	3.61047	890.42	16.85859	45.44798	4.095391	4.816739	13.24812
210.675255	4.36859	946	19.61638	51.69362	3.633849	5.60468	15.24779
230.807695	4.068315	888.25	15.65667	42.96931	3.114809	4.473335	11.58836
218.55862	4.254845	1076.75	20.9619	56.11794	3.527437	5.989115	16.70706

222.67269	4.173809	825.58	15.47479	41.82234	3.018574	4.421368	11.30098
267.453193	3.751097	1543.92	21.65386	62.92274	4.843671	6.186816	17.90276
344.174438	3.231685	1295.42	12.16357	39.07495	3.849688	3.475305	8.931883
259.208736	3.809092	1037.33	15.24364	43.78202	2.738825	4.355327	11.43455
280.265251	3.600067	935.5	12.0167	35.69438	2.806528	3.433343	8.416631
325.77097	3.346389	1543.67	15.85691	49.80839	4.385426	4.530545	12.51052
196.066885	3.911559	765.92	15.2802	40.65768	3.600094	4.365771	11.36864
181.333206	4.100984	771.3	17.44352	44.98124	4.129678	4.983862	13.34253
182.910449	4.080545	739.83	16.50485	42.70628	2.911799	4.715672	12.42431
188.204871	3.6705	848.17	16.54159	44.26431	3.813371	4.726168	12.87109
180.877921	3.940695	571.17	12.44379	32.38962	3.217859	3.555369	8.503095
211.62437	3.055948	841.75	12.15523	35.06455	3.276312	3.472924	9.099287
198.747033	3.374313	816.67	13.86537	38.4114	4.382923	3.961533	10.49105
191.446584	4.029012	787.92	16.58185	43.53568	3.426931	4.737673	12.55284
236.505698	2.726825	875.58	10.09512	30.80758	2.574629	2.88432	7.368295
204.640391	3.736564	1065	19.44602	52.90058	3.741174	5.556005	15.70945
208.090192	3.429587	1161	19.13473	53.40413	3.902521	5.467067	15.70515
213.500147	2.936728	1074.25	14.77648	43.14739	3.501124	4.221851	11.83975
279.371956	3.590115	1240.58	15.94227	47.34988	4.033357	4.554936	12.35216
265.688711	3.72113	1232.17	17.25728	50.16456	3.18456	4.930652	13.53615
371.972382	3.17518	1933.33	16.50303	54.29368	5.241792	4.715152	13.32785
214.319779	3.145978	1012.92	14.86855	42.71646	4.731502	4.248157	11.72257
197.639072	3.665073	887	16.44877	44.57394	3.34616	4.699649	12.7837
202.199682	3.280705	692.33	11.23311	31.47409	2.880747	3.209459	7.952401
266.878921	3.7378	999.58	13.99972	40.69527	3.928858	3.99992	10.26192
238.566192	3.996084	895.25	14.99581	41.68344	2.473613	4.284518	10.99973
197.516304	3.649262	1168	21.57968	58.53214	4.853118	6.165622	17.93041
205.473961	3.308759	1019.33	16.41433	46.07826	3.497804	4.689809	13.10557
214.298455	3.126938	1217.5	17.76516	51.11451	5.790725	5.075761	14.63823
150.597417	5.53667	651.3093	23.94519	54.68404	5.933638	6.841484	18.40853
163.525554	5.062711	231.4069	7.1643	17.07947	0.996824	2.046943	2.10159
163.525554	5.062711	217.2887	6.727204	16.03745	0.936008	1.922058	1.664494
146.84804	5.134547	716.869	25.06535	57.96492	2.571911	7.161528	19.9308
230.15741	3.718213	1186.632	19.17015	53.77099	5.286679	5.477184	15.45193
144.461819	5.451389	598.0628	22.56841	51.205	5.904408	6.448116	17.11702
139.086067	5.475829	952.1956	37.48802	84.15906	5.723888	10.71086	32.01219
139.086067	5.475829	450.3617	17.73078	39.80487	2.707238	5.065936	12.25495
176.090452	4.347912	722.8977	17.84933	45.0283	4.752129	5.099807	13.50141
153.591611	5.035791	864.3243	28.3385	66.59657	4.920901	8.096715	23.30271
145.743192	5.715419	935.829	36.69918	82.46912	4.286048	10.48548	30.98376
142.363537	5.834571	747.4779	30.63434	68.08571	6.693434	8.752669	24.79977
182.514935	4.752993	682.0417	17.7615	44.21428	2.350503	5.074715	13.00851
182.514935	4.752993	515.4346	13.42278	33.41374	1.776329	3.835079	8.669783
160.892602	5.20688	871.811	28.21395	66.52019	4.44856	8.061128	23.00707
162.41131	5.205491	521.2308	16.70612	39.48336	3.426625	4.773176	11.50062
150.569116	5.556056	695.7469	25.67332	58.57662	3.946307	7.335234	20.11726
198.694809	4.48521	890.2938	20.09693	51.84783	3.170639	5.741979	15.61172

139.872091	5.952004	905.5812	38.53537	84.84505	6.474352	11.01011	32.58337
151.812778	5.40259	740.1008	26.33811	60.64027	4.875089	7.525173	20.93552
147.197983	5.275913	383.2101	13.73513	31.56704	1.956453	3.924323	8.459216
147.197983	5.275913	329.8935	11.82414	27.17507	1.684249	3.378326	6.548226
167.972318	4.652973	683.432	18.93163	46.40501	2.943999	5.409038	14.27866
167.972318	4.652973	279.8553	7.752224	19.00217	1.205524	2.214921	3.099252
164.69611	4.692197	915.7785	26.09056	63.50532	3.977863	7.454444	21.39836
150.966681	5.117515	795.0188	26.94979	62.80741	9.200555	7.69994	21.83228
153.21141	5.124127	489.2901	16.36422	38.266	2.756607	4.675491	11.24009
157.31592	4.931534	531.6557	16.66632	39.60841	2.477137	4.761807	11.73479
237.204425	3.723774	1355.569	21.28052	60.11973	3.880573	6.080148	17.55674
150.460866	6.06697	926.4243	37.35582	83.36259	7.483107	10.67309	31.28885
178.170676	5.090591	1002.745	28.64986	69.68503	5.198398	8.185673	23.55927
228.770049	3.985541	1790.313	31.19012	85.85094	4.464632	8.911463	27.20458
173.05745	5.181361	543.1189	16.26104	39.09175	2.201872	4.646013	11.07968
192.993905	4.617079	1338.751	32.02754	81.43622	9.228959	9.150725	27.41046
163.745761	3.781657	890.7474	20.57153	52.77019	4.515399	5.877581	16.78988
164.679815	4.499449	952.4714	26.02381	64.00901	4.672988	7.435374	21.52436
248.760275	2.502618	1066.652	10.73091	33.88309	3.61288	3.065973	8.228287
248.760275	2.502618	500.0806	5.030992	15.88548	1.693834	1.437426	2.528373
175.038182	3.740132	1365.152	29.16991	76.2951	6.011333	8.334261	25.42978
175.038182	3.740132	527.9247	11.28044	29.50445	2.324672	3.222984	7.54031
190.174738	3.521754	1244.596	23.04807	62.47886	4.367508	6.585164	19.52632
201.252949	4.452499	1011.127	22.37007	58.00319	3.933133	6.391448	17.91757
203.867302	4.403181	1391.843	30.0614	78.41597	5.684347	8.588973	25.65822
174.066748	5.211579	854.3019	25.5779	61.48958	3.208331	7.307972	20.36632
175.806856	5.201386	918.0651	27.16169	65.49165	5.893953	7.760483	21.9603
242.085188	3.87957	668.5554	10.71403	30.11265	2.487065	3.061151	6.834459
310.938039	3.350625	1416.511	15.26413	47.37606	3.393125	4.361179	11.9135
181.903868	4.929644	963.3672	26.10751	64.34608	3.598241	7.459289	21.17787
207.293358	4.410497	1140.149	24.25849	63.51674	4.820586	6.930997	19.84799
169.038607	5.366305	803.7286	25.51519	60.44724	3.920794	7.290055	20.14889
247.22287	3.797586	498.1522	7.652108	21.73583	1.707154	2.186316	3.854521
247.22287	3.797586	495.5076	7.611484	21.62044	1.698091	2.17471	3.813898
188.507889	4.7603	763.0279	19.26838	48.33581	4.343841	5.505252	14.50808
176.719188	5.122295	1162.696	33.70133	81.67739	5.323256	9.628952	28.57904
161.280631	5.780668	589.8806	21.14267	48.59157	4.638228	6.040764	15.36201
197.913726	4.549741	1168.876	26.87071	69.00833	7.075109	7.677346	22.32097
284.538029	3.465749	1415.164	17.23708	51.88591	3.143267	4.924879	13.77133
224.457172	4.11848	949.5743	17.42338	47.34036	5.135613	4.978109	13.3049
200.556034	4.527224	1282.59	28.95237	74.69389	5.488754	8.272106	24.42515
200.556034	4.527224	485.5731	10.96102	28.2782	2.077976	3.131719	6.433794
246.067694	3.868989	1288.042	20.25223	57.19225	4.431021	5.786352	16.38324
214.868063	4.213099	862.6221	16.91416	45.20047	3.906955	4.832617	12.70106
183.592495	3.497	1274.941	24.28459	65.36881	6.646739	6.938454	20.78759
197.683749	3.333621	576.7235	9.725523	26.98837	3.115454	2.778721	6.391901
197.683749	3.333621	610.2791	10.29138	28.55864	3.296721	2.940396	6.957763

181.98068	3.639614	792.8636	15.85727	42.16683	2.521269	4.530649	12.21766
181.98068	3.639614	538.9285	10.77857	28.66181	1.713767	3.079591	7.138956
162.318064	4.521395	1133.638	31.57766	77.29538	5.066396	9.022189	27.05627
158.822594	4.963206	809.8961	25.30925	60.19589	3.948065	7.231215	20.34605
158.822594	4.963206	541.7818	16.93068	40.26817	2.641067	4.837338	11.96748
188.070461	3.463544	1185.41	21.83076	59.26096	4.963752	6.237359	18.36721
188.35389	3.418401	770.2632	13.97937	38.08688	3.199407	3.994105	10.56097
171.947106	4.672476	783.6751	21.29552	52.45057	5.649062	6.084434	16.62304
171.947106	4.672476	284.4651	7.73003	19.03896	2.050545	2.20858	3.057554
153.96966	4.950793	676.0014	21.73638	51.33071	4.417247	6.210394	16.78559
193.799292	3.347138	781.3868	13.49545	37.22692	4.0834	3.855844	10.14832
242.480685	2.706258	1659.877	18.52541	56.99607	8.179063	5.292975	15.81916
242.480685	2.706258	567.527	6.334007	19.48747	2.796496	1.809716	3.627749
205.99426	3.274948	1142.889	18.16994	51.17008	5.30809	5.19141	14.89499
184.87348	3.843523	1138.824	23.67617	62.35163	5.558354	6.764621	19.83265
188.612058	3.423086	999.955	18.148	49.44436	7.524399	5.185144	14.72492
185.957716	3.858044	642.4039	13.32788	35.11744	3.060347	3.807966	9.469838
185.957716	3.858044	696.1986	14.44395	38.05817	3.31662	4.126844	10.58591
184.748213	4.336813	988.6829	23.20852	59.29251	6.783007	6.631005	18.87171
210.984744	3.005481	1115.311	15.88762	45.98785	3.710235	4.53932	12.88214
210.984744	3.005481	793.3799	11.30171	32.7136	2.639287	3.229059	8.296227
152.086352	5.208437	1291.93	44.24418	102.8494	7.560337	12.64119	39.03574
152.086352	5.208437	563.7402	19.30617	44.87887	3.298991	5.516049	14.09773
180.679638	3.919298	1094.552	23.74299	61.86723	4.379934	6.783712	19.8237
172.911072	4.039978	1102.381	25.75657	65.87929	4.733124	7.359019	21.71659
158.912051	4.772134	770.1682	23.12817	55.55874	3.366745	6.60805	18.35604
179.107992	5.045296	853.3129	24.03698	58.67283	4.565539	6.867709	18.99169
179.107992	5.045296	345.5777	9.734583	23.76153	1.848968	2.781309	4.689288
280.843451	3.559486	1449.787	18.37499	54.76416	3.862905	5.249998	14.81551
234.586657	4.016895	1654.925	28.33776	78.33731	4.492594	8.096502	24.32086
282.845446	3.531154	2718.811	33.94271	101.5441	5.363351	9.697917	30.41155
227.622562	4.138592	1073.245	19.51355	53.14065	3.649541	5.575299	15.37495
227.622562	4.138592	624.6226	11.35677	30.92756	2.124013	3.244793	7.218183
198.747442	4.622034	909.3078	21.14669	54.15139	4.9327	6.041912	16.52466
198.747442	4.622034	403.6821	9.387956	24.0402	2.189845	2.682273	4.765922
229.46924	4.07583	1114.261	19.79149	54.21327	4.869141	5.654712	15.71566
229.46924	4.07583	560.3142	9.952295	27.26154	2.448483	2.843513	5.876465
212.398894	4.290887	1164.595	23.52717	62.40523	4.097814	6.722049	19.23628
186.252389	4.825191	988.5692	25.6106	63.83614	6.00962	7.317315	20.78541
191.738792	4.665177	605.3754	14.72933	37.29438	2.668726	4.20838	10.06415
235.349613	3.988976	1001.569	16.97575	47.04808	5.829936	4.850213	12.98677
243.977643	3.884994	1197.519	19.06877	53.68002	3.972423	5.448221	15.18378
243.977643	3.884994	830.2077	13.21987	37.21491	2.753974	3.777105	9.334874
225.261185	4.095658	728.8201	13.25127	36.0868	2.210075	3.786078	9.155617
265.2204	3.678508	1066.468	14.79151	43.1019	5.220141	4.226146	11.113
225.877735	4.077215	1356.745	24.48998	66.81376	5.754537	6.997138	20.41277
302.683714	3.416295	1526.717	17.23157	52.86683	3.850208	4.923305	13.81527

257.54576	3.737965	715.0174	10.37761	29.89869	2.961319	2.965032	6.639646
283.013866	3.528851	1175.872	14.66175	43.87623	5.243618	4.18907	11.13289
219.263581	4.184419	977.852	18.6613	50.2082	3.345972	5.331799	14.47688
280.143566	3.586985	1894.002	24.25099	72.09284	4.924819	6.928853	20.664
229.895689	2.594759	1111.885	12.54949	38.50211	5.298341	3.585569	9.954733
191.248078	3.735314	1244.495	24.30654	65.01906	6.062563	6.944727	20.57123
156.446477	4.684026	437.6544	13.10343	31.50079	2.965524	3.743836	8.419399
176.551726	4.193628	516.0513	12.25775	31.22348	2.03797	3.502214	8.064123
177.532487	4.177235	597.5064	14.05897	35.8964	3.060938	4.01685	9.881739
177.532487	4.177235	425.3267	10.00769	25.55236	2.178886	2.859339	5.830452
178.578831	4.313498	646.4204	15.61402	39.60635	4.931164	4.461148	11.30052
159.570795	4.879841	766.6175	23.44396	56.06191	4.429198	6.698274	18.56412
184.279277	3.783969	760.7153	15.62044	41.26439	3.375914	4.462982	11.83647
123.840622	2.433018	1152.714	22.64664	60.48995	6.893089	6.470469	20.21362
123.840622	2.433018	597.5239	11.73917	31.35573	3.57312	3.354049	9.306155
230.065095	2.665876	1562.209	18.10207	55.17356	3.796633	5.172021	15.4362
223.690843	2.704847	1408.339	17.02949	51.35445	4.841125	4.865569	14.32465
194.185503	3.269116	1770.473	29.80594	82.74647	7.57678	8.515984	26.53683
158.022134	4.90752	689.7449	21.42065	51.0266	4.981133	6.120185	16.51313
181.053218	3.835873	1142.048	24.19593	63.42024	8.052731	6.913123	20.36006
201.645031	3.273458	986.6358	16.01681	44.87158	4.428117	4.576233	12.74336
201.645031	3.273458	455.7183	7.398024	20.72578	2.045308	2.113721	4.124566
232.14411	2.724696	1408.571	16.53252	50.22837	3.842842	4.723578	13.80783
182.460512	3.898729	733.1526	15.66565	40.97415	3.013662	4.475901	11.76692
183.09226	3.822385	1016.261	21.2163	55.81535	5.450468	6.061801	17.39392
189.634528	3.703799	779.8481	15.23141	40.74343	3.809141	4.351831	11.52761
173.105739	4.191422	378.1142	9.155308	23.20921	3.195721	2.615802	4.963885
179.517535	3.852308	570.6779	12.24631	31.99644	3.114145	3.498945	8.393999
206.277689	3.238268	1488.19	23.36248	66.0015	6.934415	6.674994	20.12421
184.446948	3.771921	1521.259	31.10959	82.26622	4.476839	8.888455	27.33767
215.548211	4.319603	1065.097	21.34463	56.73013	5.742983	6.098466	17.02503
215.548211	4.319603	277.0178	5.551459	14.75476	1.493675	1.586131	1.231855
231.578334	4.055663	1242.913	21.7673	59.83615	5.291793	6.219229	17.71164
245.207772	3.849416	1535.409	24.10375	68.09567	7.817717	6.886786	20.25434
210.56357	4.288464	1229.4	25.0387	66.27994	6.35166	7.153913	20.75023
210.56357	4.288464	590.246	12.0213	31.82159	3.049489	3.434658	7.73284
216.358164	4.309924	1162.47	23.15677	61.63877	2.966151	6.616221	18.84685
254.816452	3.866714	612.214	9.290046	26.4691	2.405045	2.654299	5.423332
254.816452	3.866714	358.4568	5.439405	15.4979	1.408175	1.554116	1.572691
164.7121	5.545862	848.5915	28.5721	66.70085	3.333237	8.163458	23.02624
200.216224	4.529779	1105.975	25.02206	64.51766	6.231424	7.14916	20.49228
200.216224	4.529779	452.9438	10.2476	26.42273	2.552033	2.927885	5.717818
245.674945	3.838671	934.7785	14.60591	41.31176	3.133335	4.173118	10.76724
252.003448	3.761245	1013.715	15.13007	43.28723	2.661228	4.322878	11.36883
191.17221	4.743727	1384.488	34.35454	86.55864	8.205881	9.815583	29.61081
331.561803	3.299122	2042.791	20.32628	64.35753	5.979658	5.807508	17.02716
331.561803	3.299122	1270.211	12.63892	40.01763	3.718162	3.611119	9.339793

236.129169	4.043308	1330.417	22.78111	62.97645	5.719825	6.508889	18.73781
236.129169	4.043308	413.2984	7.077027	19.56384	1.776882	2.022008	3.03372
243.884175	3.927281	1300.279	20.93847	58.77841	7.488577	5.98242	17.01119
187.198616	4.824707	1048.238	27.01644	67.42735	4.766779	7.718984	22.19174
205.38658	4.484423	1310.765	28.61932	74.45189	5.138415	8.176949	24.1349
284.376077	3.532622	1669.749	20.74222	62.13042	4.417543	5.92635	17.2096
284.376077	3.532622	681.1805	8.46187	25.34634	1.802153	2.417677	4.929247
293.473029	3.497891	2614.429	31.16125	94.30949	7.964444	8.903215	27.66336
293.473029	3.497891	851.5493	10.14957	30.71767	2.59411	2.899878	6.651684
211.546662	4.343874	1274.729	26.17513	69.14665	6.27293	7.47861	21.83126
211.546662	4.343874	764.1873	15.69173	41.45273	3.760559	4.483352	11.34786
275.859337	3.591918	2131.434	27.75305	82.15821	6.634717	7.929442	24.16113
210.282638	4.353678	1855.104	38.40795	101.253	4.43043	10.9737	34.05427
230.044733	4.050083	1360.364	23.95007	65.74972	5.960533	6.842877	19.89999
230.044733	4.050083	522.694	9.202359	25.26308	2.290221	2.629245	5.152276
200.021626	3.496882	1739.796	30.41601	83.64718	9.465604	8.69029	26.91913
210.179427	2.715496	657.8804	8.499747	25.21107	3.146288	2.428499	5.78425
186.848674	3.908968	1162.09	24.31151	63.92472	3.8601	6.946145	20.40254
210.238278	3.137885	1034.403	15.43885	44.17064	3.324413	4.4111	12.30097
162.041019	3.641371	1134.816	25.50148	65.86511	3.304441	7.286138	21.86011
177.505143	3.971032	1133.346	25.3545	65.55893	7.168788	7.244142	21.38346
199.037743	3.246945	694.3634	11.3273	31.69505	3.754885	3.236371	8.080353
231.490807	4.119053	1519.724	27.04135	74.03933	6.941122	7.726101	22.9223
355.638523	3.198188	1382.179	12.42967	40.36321	3.409202	3.551334	9.231479
355.638523	3.198188	970.1079	8.723992	28.32967	2.392811	2.492569	5.525804
233.432702	4.059699	1498.922	26.06821	71.78408	5.437226	7.44806	22.00851
224.527932	4.204643	1402.306	26.26041	70.98831	5.381166	7.502975	22.05577
276.388359	3.62239	2152.64	28.21284	83.38309	7.618573	8.060813	24.59045
125.865228	6.521514	318.562	16.5058	34.59601	2.46789	4.715944	9.984288
119.464744	7.027338	310.3173	18.25396	37.06545	3.844384	5.215417	11.22662
121.539859	6.264941	498.4549	25.69355	53.92307	5.967966	7.341015	19.42861
151.984834	5.408713	862.518	30.69459	70.67054	5.08323	8.769883	25.28588
126.69156	6.240964	595.4687	29.33343	62.26399	4.161237	8.380981	23.09247
128.070344	6.041054	573.3329	27.04401	58.03033	4.008723	7.726859	21.00295
145.288803	5.188886	751.0239	26.82228	61.70001	5.035958	7.66351	21.6334
144.574422	5.200519	833.5172	29.98263	68.84636	7.452482	8.566467	24.78211
148.286087	5.553786	852.2006	31.91762	72.5535	6.001596	9.119321	26.36384
119.150431	7.134756	295.7353	17.7087	35.79858	4.670178	5.059629	10.57394
171.544364	4.887304	753.9939	21.48131	52.28625	4.36541	6.137517	16.594
147.968635	5.562731	753.3244	28.32047	64.31626	5.301897	8.091562	22.75773
131.065065	6.362382	429.1852	20.83423	44.38583	3.75814	5.952638	14.47185
130.773612	5.864288	855.921	38.38211	83.40749	5.785226	10.96632	32.51782
132.78227	5.823784	657.195	28.82434	62.98587	4.533431	8.235526	23.00056
140.556792	5.512031	820.1509	32.16278	72.27509	5.889521	9.189365	26.65075
145.997016	5.27065	757.5467	27.34826	62.74073	5.35715	7.813787	22.0776
128.856368	6.078131	527.7669	24.89466	53.41832	4.265992	7.112761	18.81653
137.528675	5.706584	820.9825	34.06566	75.47813	5.102597	9.733047	28.35908

139.746463	5.333834	545.9476	20.83769	47.14384	5.6295	5.953627	15.50386
134.824879	5.811417	607.6555	26.19205	57.48327	5.627838	7.483442	20.38063
162.081535	5.194921	1149.524	36.84372	87.07673	6.633217	10.52678	31.6488
169.512484	4.985661	1280.557	37.66343	90.94726	7.187873	10.76098	32.67777
149.093824	5.213071	954.6734	33.38019	77.19342	5.27436	9.537196	28.16712
154.27552	4.992735	746.5594	24.1605	56.96335	4.33934	6.902999	19.16776
135.14999	5.775641	526.1061	22.48317	49.44945	5.867285	6.423762	16.70753
142.396225	5.414305	823.7085	31.31972	70.92621	8.283106	8.94849	25.90541
168.882124	4.69117	920.8678	25.57966	62.65712	4.351201	7.308475	20.88849
145.929642	5.268218	660.8591	23.85773	54.73297	5.875782	6.816494	18.58951
131.225032	6.239007	860.5	40.9119	87.61429	4.157005	11.68911	34.67289
125.652752	6.293021	778.5	38.98933	82.41837	3.747292	11.13981	32.69631
112.862778	7.28147	465.5	30.03226	59.58966	3.340989	8.580645	22.75079
138.309994	5.936051	882.25	37.86481	83.19069	5.566246	10.81852	31.92876
127.033029	6.514514	781.83	40.09385	84.25319	3.693627	11.45538	33.57933
136.510756	6.040299	763.33	33.77566	73.64292	5.18109	9.65019	27.73536
132.945081	6.212387	697.42	32.58972	70.09452	5.245926	9.311348	26.37733
150.22095	5.517758	1078.17	39.6022	90.46099	5.357366	11.31492	34.08445
120.467547	6.442115	694.67	37.14813	77.2498	3.873697	10.61375	30.70601
152.726621	4.830522	691.83	21.88158	51.88704	3.251845	6.251881	17.05106
134.610211	5.679756	739.75	31.21308	68.86899	5.146087	8.918023	25.53332
135.553177	5.695512	799.5	33.59244	74.1969	4.087841	9.597839	27.89693
148.2506	5.180508	812	28.37474	65.62782	5.561644	8.107069	23.19423
137.42327	5.655279	816.5	33.60082	74.60217	5.140716	9.600235	27.94554
138.975887	5.649426	881.75	35.8435	79.82596	5.225185	10.241	30.19407
138.721557	5.909332	666.5	28.39191	62.4951	2.731557	8.111973	22.48257
148.948917	5.49627	1156.83	42.68745	97.39633	6.310097	12.19642	37.19118
143.991703	5.744273	1185.58	47.29645	105.8289	6.682336	13.51327	41.55217
138.245848	5.958873	1376	59.31034	130.1675	7.059306	16.94581	53.35147
188.107499	4.57961	860.83	20.95752	53.05596	4.033502	5.987862	16.37791
149.572809	5.529494	1055.58	39.02329	88.99502	6.766538	11.14951	33.4938
129.561069	6.413914	910.58	45.07822	95.56626	4.811519	12.87949	38.6643
229.293976	4.117845	1519	27.27942	74.51879	6.147806	7.79412	23.16158
142.628229	5.809704	1087.33	44.29043	98.5877	6.19279	12.65441	38.48072
153.73806	5.381666	959	33.5702	77.61044	5.941023	9.591487	28.18854
150.82938	5.489695	1115.25	40.59145	92.93279	6.455861	11.59756	35.10175
163.440276	5.091597	1417.25	44.15109	105.0915	6.671924	12.6146	39.05949
141.162708	5.436235	839.17	32.31679	72.9514	3.927411	9.233368	26.88055
127.963273	5.993596	787.58	36.88899	79.29509	3.94125	10.53971	30.8954
140.08276	5.544099	929.75	36.79701	82.49943	4.701643	10.51343	31.25291
129.838585	6.006318	676.58	31.29852	67.48738	3.554773	8.942433	25.2922
153.973089	5.405789	1351.75	47.45813	109.637	7.604782	13.55947	42.05234
164.102754	5.084516	861.33	26.68722	63.60919	4.947898	7.62492	21.6027
257.803164	3.880649	896.25	13.49104	38.51601	4.621751	3.854582	9.610387
149.103028	5.563546	831	31.00746	70.55047	4.496023	8.859275	25.44392
151.770889	5.535649	835.5	30.47379	69.7319	5.035559	8.706798	24.93814
148.928487	5.602396	942.5	35.45499	80.50602	5.770526	10.13	29.85259

175.372551	4.861332	1109.5	30.75537	75.37417	5.095293	8.787249	25.89404
169.195547	5.013201	1063.67	31.51615	75.96291	6.08681	9.004614	26.50295
156.702661	5.317903	1370.58	46.51237	108.3683	7.080174	13.28925	41.19447
162.909392	5.163531	1111.92	35.24311	83.52645	6.480476	10.06946	30.07958
137.841503	5.580628	686.5	27.79352	61.96092	4.324409	7.941006	22.21289
139.437492	5.551519	957.08	38.10487	85.30459	5.638174	10.88711	32.55335
136.355385	5.669663	788.08	32.7684	72.56614	4.039779	9.3624	27.09874
155.498682	4.923493	1193.42	37.78678	89.57839	4.264804	10.79622	32.86329
146.475821	5.130502	1049.83	36.77163	84.99911	7.467847	10.50618	31.64113
160.774817	4.842615	818.67	24.65873	59.19095	4.785305	7.045353	19.81612
149.426896	5.157097	1025.83	35.40397	82.14059	5.88982	10.11542	30.24687
156.653677	4.926216	1231.75	38.73428	91.98187	6.434803	11.06694	33.80806
131.032932	6.08974	806	37.45875	80.67682	6.113935	10.7025	31.36901
236.08338	3.724711	1250.08	19.72264	55.64911	5.437022	5.63504	15.99793
133.293582	5.884926	818.25	36.12583	78.81064	4.148289	10.32167	30.2409
156.723468	4.892563	930.42	29.04567	69.10044	5.565712	8.298763	24.15311
154.797533	4.940399	759.67	24.24505	57.3619	4.750313	6.927156	19.30465
172.036285	4.93294	1245.17	35.7038	86.76468	4.920065	10.20109	30.77086
185.204492	4.674874	1211.83	30.58864	76.74154	5.940343	8.73961	25.91376
163.617813	5.129085	1207.67	37.85799	89.97156	6.516322	10.81657	32.72891
162.756174	5.142375	1167.5	36.88784	87.49366	4.219829	10.53938	31.74546
182.625586	4.740444	1424.5	36.97599	92.12033	4.646422	10.56457	32.23555
149.254256	5.543334	852.75	31.67131	72.14479	4.804225	9.048945	26.12798
175.857865	4.925991	1423.17	39.86471	97.44412	6.525908	11.38992	34.93872
166.622573	5.056831	1457.33	44.22853	105.966	5.852731	12.63672	39.1717
178.043597	4.828301	1941.67	52.65546	129.7557	6.256791	15.04442	47.82716
158.210113	4.870551	1333.92	41.06517	98.03647	6.116654	11.73291	36.19462
181.195391	4.343442	1348.5	32.32495	82.15162	3.862016	9.2357	27.98151
163.107795	4.769234	1205.5	35.24854	85.24082	5.353733	10.07101	30.4793
175.50344	4.394948	968.17	24.24486	60.94705	3.894802	6.927103	19.84991
154.513865	4.997699	1074.25	34.74626	81.93272	6.570336	9.927502	29.74856
158.286299	4.850498	949.58	29.09877	69.54867	5.92266	8.313933	24.24827
231.095685	3.748511	1075.42	17.44396	48.87967	3.440463	4.983988	13.69545
153.196121	5.043494	1435.42	47.25663	110.9409	6.240957	13.50189	42.21313
154.882145	5.028641	923.83	29.99448	70.66088	5.613258	8.569852	24.96584
150.975432	5.139589	1005.67	34.23557	79.70253	4.831004	9.781593	29.09599
147.623891	5.23489	1063.08	37.69787	86.87186	5.556264	10.77082	32.46298
184.453728	4.34776	1323.92	31.20613	79.64259	6.370206	8.916037	26.85837
191.621382	4.158179	1764.42	38.28787	99.7577	6.971788	10.93939	34.12969
160.485403	4.831424	851.42	25.63206	61.53519	4.18039	7.323445	20.80063
171.920178	5.225537	855.25	25.99544	62.25814	4.150087	7.427269	20.7699
202.898097	4.447861	1422.67	31.18728	81.05114	5.378715	8.91065	26.73942
195.031801	4.663936	1667.92	39.88617	101.4286	5.465544	11.39605	35.22223
156.648556	5.922441	1038.83	39.27524	89.06872	4.858205	11.2215	33.3528
182.894255	5.000526	1668.75	45.62543	112.2026	4.941955	13.03584	40.6249
213.170411	4.240763	1841.33	36.63099	97.537	6.832393	10.466	32.39023
193.425084	4.649641	1492.83	35.88534	91.13608	7.205821	10.25295	31.2357

183.665118	4.795434	1418.58	37.03864	92.14139	5.433507	10.58247	32.24321
169.501588	5.415386	1117.25	35.69489	84.42909	3.943282	10.19854	30.2795
173.206348	5.268634	1106.83	33.66783	80.61792	4.863477	9.619381	28.3992
178.013252	5.120179	1390	39.98044	97.08215	7.8125	11.42298	34.86026
178.664054	5.004595	1142.33	31.99804	78.21507	5.491971	9.142297	26.99344
194.730435	4.555098	1506.5	35.23977	90.10877	5.284482	10.0685	30.68467
225.114198	3.794081	1207.08	20.34416	56.46298	4.336866	5.812617	16.55008
162.17521	4.433076	1306.67	35.71796	87.84285	6.068221	10.20513	31.28488
157.020172	4.557915	986.25	28.62845	69.35777	4.820852	8.179556	24.07053
161.831819	4.205281	1151.17	29.91373	74.5054	7.31273	8.546779	25.70845
160.096518	4.833102	1221.92	36.88815	88.49645	6.741628	10.53947	32.05505
141.375579	5.882561	1070	44.52212	98.5775	6.372082	12.7206	38.63955
150.402986	5.469199	987.33	35.90291	82.21722	5.816377	10.25797	30.43371
185.975968	3.574193	1201.17	23.08477	62.00052	6.146607	6.595649	19.51058
190.674529	3.37776	1655.58	29.32826	80.39003	5.690843	8.379501	25.9505
161.21654	4.528554	1012.08	28.42921	69.44282	4.501534	8.122632	23.90066
185.515638	4.831136	1321	34.40104	85.63561	5.310553	9.828869	29.56991
185.792642	4.882855	1372	36.05782	89.55464	6.084257	10.30223	31.17496
216.887704	4.136473	1569.25	29.92867	80.53573	7.313123	8.551049	25.7922
239.840428	3.915762	1799.42	29.37829	82.18697	6.747741	8.393796	25.46252
180.118425	5.014852	1512	42.09706	103.0568	6.023904	12.02773	37.08221
168.865864	5.408034	1194.5	38.2546	90.42933	3.989379	10.92989	32.84657
194.591056	4.575921	1897.33	44.61681	113.9356	7.957931	12.74766	40.04089
195.390398	4.663255	1569.67	37.46229	95.31206	7.303508	10.70351	32.79904
175.937533	5.159458	1215	35.6305	86.10146	5.466817	10.18014	30.47104
176.811435	5.110157	1198.08	34.62659	83.98056	3.93885	9.893311	29.51643
173.552921	5.165266	940.08	27.97857	67.36128	3.429947	7.993878	22.81331
207.953064	4.305446	1294.92	26.80994	70.67773	4.635807	7.659982	22.50449
148.471382	5.027304	958.67	32.46098	75.67257	5.05015	9.274565	27.43367
217.756525	2.826795	1152.92	14.96657	44.33958	4.305957	4.276164	12.13978
203.73784	3.189634	1521.42	23.81871	67.33656	6.323967	6.805345	20.62907
185.912936	3.556441	1275.08	24.39177	65.58687	5.907798	6.969078	20.83533
192.807635	3.531275	993.42	18.19451	49.45821	3.707898	5.19843	14.66323
149.333039	5.295498	758.83	26.90887	62.00942	4.841021	7.688247	21.61337
179.79542	4.152319	1560.33	36.03533	92.438	5.972555	10.29581	31.88302
172.576349	4.102416	1381.92	32.85045	83.66171	5.710413	9.385844	28.74804
204.958319	3.354473	1090.5	17.84779	49.89934	5.062204	5.099369	14.49332
201.699296	3.455708	1843	31.57606	87.277	6.510527	9.021732	28.12035
242.301959	3.878383	1714.83	27.44826	77.16873	4.750222	7.84236	23.56988
275.926731	3.593966	1906.08	24.82683	73.48966	5.950364	7.09338	21.23287
160.441495	5.736815	1306.08	46.70075	107.3953	6.935061	13.34307	40.96394
185.804266	4.93727	1015	26.97101	66.80199	3.755782	7.706003	22.03374
193.056639	4.739563	1244.08	30.54231	77.15934	6.331196	8.726375	25.80275
227.884273	4.023878	2100	37.08085	101.7225	8.020471	10.59453	33.05698
182.95186	5.019255	1152.92	31.63018	77.71879	4.916503	9.037194	26.61092
162.231137	5.636353	1105	38.39072	88.92221	6.116123	10.96878	32.75436
280.232835	3.548733	2433.25	30.8135	91.85494	7.975777	8.803858	27.26477

219.887644	4.16983	1619.83	30.71758	82.7766	5.495606	8.776451	26.54775
253.634278	3.845857	1940.17	29.4188	83.83564	6.263664	8.405372	25.57294
212.442665	4.310668	1533	31.10606	82.41747	4.669083	8.887446	26.79539
211.953867	4.345543	1940.42	39.78309	105.1351	6.923889	11.3666	35.43754
186.159241	3.763302	1082.33	21.87984	58.02605	4.869876	6.251383	18.11654
194.269713	3.868605	1618.42	32.22853	85.79326	5.966525	9.208151	28.35992
165.895044	4.886452	1236.92	36.43358	87.94513	6.0141	10.40959	31.54713
188.851085	3.671788	1213.5	23.5938	63.18419	6.199234	6.741086	19.92201
186.976717	3.792631	1866.08	37.85152	100.2986	7.225866	10.81472	34.05889
184.082111	3.939018	1065.75	22.80508	59.62626	3.981582	6.515738	18.86607
187.035117	3.828764	1755.83	35.9433	95.02416	5.708531	10.26951	32.11453
172.833108	4.241303	764	18.74847	47.36937	3.538348	5.356705	14.50716
183.684359	3.909592	1167.58	24.85112	65.06255	4.303173	7.100319	20.94152
184.547821	3.68234	862.5	17.20973	45.78996	5.169933	4.917065	13.52739
174.833487	4.170945	1233.5	29.4272	74.87667	5.248936	8.407772	25.25626
196.524127	3.810453	1508	29.23897	78.35594	6.551679	8.353992	25.42852
195.381188	3.267244	1259.5	21.06187	58.5696	5.442486	6.017678	17.79463
208.64483	4.397151	1503.25	31.68072	83.14843	5.626567	9.051633	27.28357
205.251048	4.474137	1686.17	36.75575	95.65756	6.958156	10.50164	32.28161
217.607937	4.240465	1390.17	27.08985	72.50567	5.125051	7.739958	22.84939
244.552522	3.928555	2835.92	45.55695	127.9645	11.01328	13.01627	41.62839
212.263891	4.396973	1552.17	32.15267	84.75154	5.564929	9.186476	27.75569
225.05075	4.186991	1513	28.14884	76.21757	5.828646	8.042525	23.96185
175.581306	5.210128	1414.92	41.98576	101.1601	6.307315	11.99593	36.77563
231.778553	4.105909	748.58	13.26094	36.34882	2.289235	3.78884	9.15503
252.507493	3.823959	1994.92	30.21096	86.12014	7.478613	8.631701	26.387
211.056254	4.406185	2393.25	49.96347	131.4427	7.430607	14.27528	45.55728
187.368653	4.86356	1253.92	32.54822	81.08917	5.493625	9.29949	27.68466
201.012197	4.578866	2176.75	49.58428	127.6322	9.379309	14.16694	45.00542
292.779699	3.487549	2318.25	27.61465	83.58816	6.30679	7.8899	24.1271
248.234535	3.876666	2272.67	35.49217	100.3999	6.748834	10.14062	31.6155
241.683449	3.882465	2097.58	33.69606	94.64856	4.547599	9.627447	29.8136
218.099251	3.238296	2018.5	29.9703	85.85697	7.905456	8.562944	26.73201
187.620576	3.804379	1412.83	28.64793	75.91759	6.323651	8.185123	24.84355
190.80057	3.840978	1899.5	38.23855	101.5163	6.966296	10.9253	34.39757
186.17938	4.13732	1167	25.93333	67.16785	4.045061	7.409524	21.79601
190.112104	3.554627	953.92	17.83595	48.23365	5.078635	5.095985	14.28132
199.979126	3.341339	1585.42	26.48989	73.67942	5.704591	7.56854	23.14855
228.518147	2.857084	1337.42	16.7213	50.0057	5.080418	4.777515	13.86422
195.686145	3.614914	1772.92	32.75119	88.83673	7.926499	9.357482	29.13627
190.694219	3.61047	1076	20.37223	54.92018	4.948947	5.820637	16.76176
210.675255	4.36859	1881.42	39.01337	102.8091	7.227058	11.14668	34.64478
230.807695	4.068315	1699.5	29.95611	82.21373	5.959603	8.558889	25.8878
218.55862	4.254845	2067.42	40.24802	107.7496	6.772875	11.49943	35.99317
222.67269	4.173809	1909.33	35.78875	96.72309	6.981097	10.22536	31.61494
267.453193	3.751097	2005.75	28.13114	81.74471	6.292549	8.037467	24.38004
344.174438	3.231685	2712.33	25.46789	81.81451	8.060416	7.276539	22.2362

259.208736	3.809092	2179.33	32.02542	91.9818	5.754007	9.150121	28.21633
280.265251	3.600067	2201.42	28.27771	83.99607	6.604326	8.079347	24.67765
325.77097	3.346389	2742.75	28.17411	88.49816	7.791903	8.049747	24.82773
196.066885	3.911559	1632.5	32.56858	86.65875	7.673325	9.305308	28.65702
182.910449	4.080545	1451.75	32.38706	83.80147	5.713752	9.253446	28.30652
188.204871	3.6705	1720.17	33.54793	89.77225	7.733882	9.585122	29.87743
180.877921	3.940695	1272.75	27.72876	72.17447	7.170423	7.922502	23.78806
211.62437	3.055948	1367.33	19.74484	56.9585	5.322007	5.641382	16.68889
198.747033	3.374313	1225.42	20.80509	57.63662	6.576611	5.944312	17.43078
191.446584	4.029012	913.17	19.21775	50.45624	3.971686	5.490787	15.18874
236.505698	2.726825	1112.92	12.83156	39.15847	3.272524	3.666161	10.10474
204.640391	3.736564	2324.83	42.44947	115.4787	8.166754	12.12842	38.7129
208.090192	3.429587	1542.67	25.42513	70.96034	5.185445	7.264324	21.99555
213.500147	2.936728	1207.5	16.60935	48.4994	3.935404	4.74553	13.67263
279.371956	3.590115	2164.75	27.81847	82.62318	7.038006	7.948135	24.22836
265.688711	3.72113	1664.42	23.3112	67.76248	4.301716	6.660344	19.59007
371.972382	3.17518	3995.17	34.10303	112.1963	10.83201	9.743723	30.92785
214.319779	3.145978	2200.08	32.29475	92.7809	10.27691	9.227072	29.14877
197.639072	3.665073	1530.67	28.38516	76.91994	5.77437	8.110047	24.72009
202.199682	3.280705	1402.08	22.74885	63.74011	5.833978	6.499672	19.46815
266.878921	3.7378	3844.17	53.83992	156.5053	15.10954	15.38283	50.10212
238.566192	3.996084	2235.92	37.4526	104.1059	6.17794	10.70074	33.45651
197.516304	3.649262	1711.75	31.62587	85.78116	7.112436	9.035962	27.9766
205.473961	3.308759	1391.92	22.41417	62.92099	4.776337	6.404049	19.10541
214.298455	3.126938	1090.17	15.90723	45.76879	5.185113	4.544922	12.78029
125.865228	6.521514	402.4977	20.8548	43.71147	3.118138	5.958515	14.33329
119.464744	7.027338	378.3273	22.25455	45.18882	4.68693	6.358442	15.22721
132.309476	6.182686	485.5059	22.68719	48.796	8.354802	6.482055	16.50451
121.539859	6.264941	344.5529	17.76046	37.27388	4.125308	5.074417	11.49552
151.984834	5.408713	714.182	25.41573	58.51661	4.209015	7.261637	20.00702
156.609275	5.237768	585.4781	19.58121	45.78859	6.011333	5.594631	14.34344
126.69156	6.240964	629.4039	31.00512	65.81235	4.398382	8.858605	24.76415
128.070344	6.041054	523.5897	24.69763	52.99553	3.66092	7.056465	18.65657
145.288803	5.188886	745.7107	26.63253	61.26351	5.000331	7.609293	21.44364
144.574422	5.200519	703.7178	25.31359	58.12526	6.291944	7.232454	20.11307
148.286087	5.553786	690.1055	25.84665	58.75327	4.860046	7.384757	20.29286
119.150431	7.134756	424.6896	25.43051	51.40842	6.706591	7.265861	18.29576
171.544364	4.887304	605.932	17.26302	42.01879	3.508174	4.932291	12.37572
147.968635	5.562731	700.1088	26.31988	59.7729	4.927366	7.519966	20.75715
131.065065	6.362382	505.257	24.52704	52.25308	4.424259	7.007725	18.16466
130.773612	5.864288	586.1253	26.28365	57.11653	3.961659	7.509613	20.41936
132.78227	5.823784	530.0082	23.24597	50.79623	3.656077	6.641707	17.42219
140.556792	5.512031	591.1566	23.18261	52.09516	4.245108	6.623603	17.67058
145.997016	5.27065	612.8451	22.12437	50.75641	4.333862	6.321249	16.85372
128.856368	6.078131	535.9162	25.27907	54.24316	4.331864	7.22259	19.20094
137.528675	5.706584	602.2486	24.98957	55.36853	3.743115	7.139877	19.28298
139.746463	5.333834	587.3126	22.41651	50.71581	6.056033	6.404718	17.08268

134.824879	5.811417	602.4316	25.96688	56.98909	5.579456	7.419108	20.15546
162.081535	5.194921	880.9764	28.23642	66.73418	5.083588	8.067549	23.0415
169.512484	4.985661	896.6945	26.37337	63.68473	5.033222	7.535248	21.38771
177.458854	4.796185	699.5557	18.90691	46.6306	6.918423	5.401975	14.11073
162.782974	5.167713	462.5046	14.68269	34.78429	2.841235	4.195053	9.514973
163.455738	5.140118	910.6383	28.63642	68.00261	5.571161	8.181835	23.49631
182.128557	4.767763	1023.324	26.78858	66.59866	5.618688	7.653879	22.02081
182.128557	4.767763	1046.695	27.40039	68.11969	5.747012	7.828684	22.63263
140.531266	5.954715	980.6232	41.55183	91.5837	6.977972	11.87195	35.59712
161.523475	5.227297	710.3594	22.98898	54.20125	4.397871	6.56828	17.76168
149.093824	5.213071	522.3339	18.26342	42.23512	2.88578	5.218121	13.05035
174.554991	4.43033	862.3596	21.8873	54.83606	4.251109	6.253514	17.45697
154.27552	4.992735	933.3454	30.20535	71.21534	5.425024	8.630101	25.21262
135.14999	5.775641	508.7153	21.73997	47.81486	5.673337	6.21142	15.96433
142.396225	5.414305	615.0819	23.38714	52.96221	6.185184	6.682041	17.97284
168.882124	4.69117	532.0039	14.77789	36.19828	2.513777	4.222253	10.08672
145.929642	5.268218	469.614	16.95357	38.89387	4.175397	4.843878	11.68536
134.735575	5.858068	602.2519	26.18486	57.34325	4.46988	7.48139	20.3268
161.953242	5.224298	957.2539	30.87916	72.8628	5.91068	8.822616	25.65486
165.117077	5.111984	822.7344	25.47165	60.72364	4.982734	7.277615	20.35967
152.459114	5.484141	602.9013	21.6871	49.79809	3.954512	6.196314	16.20296
161.294067	5.219873	920.8764	29.80182	70.26394	5.709301	8.514807	24.58195
163.928893	5.154997	1114.188	35.03734	83.2028	6.796773	10.01067	29.88235
193.098005	4.619569	1421.353	34.00365	86.46088	7.360784	9.71533	29.38408
241.307853	4.055594	1605.592	26.98474	74.94582	6.653707	7.709924	22.92914
171.776021	4.979015	1424.625	41.29348	100.0775	8.293505	11.79814	36.31447
205.282438	4.42419	1184.431	25.52653	66.62257	5.769764	7.293296	21.10234
164.530341	5.141573	1129.045	35.28267	83.9168	6.862232	10.08076	30.1411
189.505306	4.737633	1261.657	31.54143	79.32251	6.657636	9.011838	26.8038
175.604541	5.134636	1172.657	34.2882	82.91847	6.677826	9.79663	29.15357
185.146652	4.87228	1351.868	35.57548	88.32764	7.301607	10.16442	30.7032
158.085982	4.761626	1201.926	36.20258	86.90085	7.602987	10.34359	31.44095
224.780909	4.12442	1744.267	32.00491	86.95923	7.759856	9.144259	27.88049
239.68678	4.00814	2336.778	39.07655	108.6655	9.749298	11.16473	35.06841
231.033238	4.017969	1896.279	32.97876	90.81368	8.207817	9.422502	28.96079
166.725977	5.466425	753.3668	24.70055	58.04724	4.518593	7.0573	19.23413
209.405422	4.390051	1443.825	30.26887	79.54736	6.894879	8.648248	25.87882
211.033713	4.280603	1384.521	28.08359	74.41567	6.560663	8.023883	23.80299
184.638422	4.884614	1573.527	41.6277	103.218	8.522208	11.89363	36.74309
216.220142	4.239611	1785.622	35.01219	93.56466	8.258351	10.00348	30.77258
214.257454	4.25114	1987.053	39.42565	105.0477	9.274137	11.26447	35.17451
206.946386	4.45046	1307.192	28.11166	73.40907	6.316574	8.031902	23.6612
293.963964	3.524748	2026.964	24.30412	73.44656	6.89528	6.944035	20.77937
210.677441	3.163325	2116.125	31.77365	90.7686	10.04438	9.078185	28.61032
199.611195	3.738037	1865.601	34.93635	94.44149	9.346174	9.981814	31.19831
247.140062	3.898108	2297.595	36.23966	102.2602	9.296731	10.35419	32.34156
269.163597	3.702388	2528.517	34.78015	101.5582	9.393977	9.937185	31.07776

229.283526	4.131235	1468.502	26.4595	72.21957	6.404744	7.559858	22.32827
261.289612	3.743404	1680.586	24.07716	69.59356	6.431888	6.879188	20.33375
230.449362	4.115167	1916.408	34.22157	93.61533	8.315962	9.777593	30.10641
283.427407	3.583153	2657.039	33.59088	100.1764	9.37467	9.597394	30.00773
284.221815	3.643869	2421.535	31.04532	92.26136	8.519878	8.870092	27.40145
261.383607	3.77722	1085.248	15.68278	45.2324	4.151938	4.480794	11.90556
264.134234	3.762596	1996.096	28.43441	82.30544	7.557126	8.124118	24.67182
232.832225	4.070493	2012.452	35.18273	96.75615	8.643359	10.05221	31.11224
265.423242	3.770217	2496.407	35.46033	102.7155	9.405383	10.13152	31.69011
234.626493	4.066317	1630.227	28.2535	77.8693	6.94818	8.072429	24.18719
226.65904	4.189631	2222.952	41.08968	111.4377	9.80747	11.73991	36.90005
298.889199	3.467392	1969.569	22.84883	69.62107	6.589628	6.528236	19.38143
125.865228	6.521514	470.2569	24.36564	51.07015	3.643066	6.961612	17.84413
119.464744	7.027338	328.6661	19.3333	39.2571	4.0717	5.523801	12.30596
132.309476	6.182686	448.619	20.9635	45.08866	7.720035	5.989572	14.78082
121.539859	6.264941	349.9548	18.03891	37.85826	4.189985	5.153974	11.77397
151.984834	5.408713	565.0749	20.10943	46.2995	3.330256	5.74555	14.70071
156.609275	5.237768	466.5458	15.60354	36.48723	4.790208	4.458153	10.36577
126.69156	6.240964	493.5815	24.31436	51.61036	3.449232	6.94696	18.0734
128.070344	6.041054	430.8	20.32075	43.60375	3.012138	5.80593	14.2797
145.288803	5.188886	641.77	22.92036	52.7243	4.303361	6.548673	17.73147
144.574422	5.200519	563.9741	20.28684	46.5828	5.042496	5.79624	15.08632
148.286087	5.553786	383.8616	14.37684	32.68069	2.703333	4.107668	8.823052
119.150431	7.134756	328.2725	19.65703	39.73719	5.183997	5.616296	12.52228
171.544364	4.887304	626.9554	17.86198	43.47667	3.629893	5.103422	12.97467
147.968635	5.562731	532.407	20.0153	45.45509	3.747081	5.718658	14.45257
131.065065	6.362382	426.8006	20.71848	44.13922	3.73726	5.919565	14.3561
130.773612	5.864288	400.2945	17.95043	39.00776	2.705617	5.128694	12.08614
132.78227	5.823784	600.1402	26.32194	57.51771	4.139858	7.520554	20.49816
140.556792	5.512031	503.1849	19.73274	44.34273	3.613382	5.637926	14.22071
145.997016	5.27065	584.0466	21.08471	48.37129	4.130207	6.024204	15.81406
128.856368	6.078131	456.3725	21.527	46.19208	3.688904	6.150572	15.44887
137.528675	5.706584	483.8516	20.07683	44.48355	3.00725	5.736237	14.37025
139.746463	5.333834	572.4243	21.84826	49.43017	5.902513	6.242359	16.51442
134.824879	5.811417	564.3823	24.32682	53.38969	5.227061	6.950521	18.51541
169.384941	5.041218	1407.149	41.87945	100.8291	7.010696	11.96556	36.83823
155.994988	5.324061	1054.223	35.98032	83.71089	5.993258	10.28009	30.65626
152.429313	5.424531	1012.599	36.03556	82.96748	6.64219	10.29588	30.61103
157.785369	5.294811	1133.819	38.04761	88.89583	7.004022	10.87074	32.7528
145.441344	5.659196	511.7159	19.91112	44.83107	2.75062	5.688893	14.25193
140.501841	5.903439	646.5535	27.16611	60.00283	4.601744	7.761746	21.26267
139.713998	5.920085	579.6377	24.56092	54.13431	2.712479	7.017405	18.64083
147.918827	5.581843	665.5053	25.11341	56.9793	3.33175	7.175259	19.53157
161.222002	5.20071	838.7783	27.05736	63.84486	3.404271	7.730676	21.85665
162.081535	5.194921	630.8306	20.21893	47.78557	3.640146	5.776837	15.02401
169.512484	4.985661	691.789	20.34674	49.132	3.88307	5.813353	15.36107
177.458854	4.796185	566.3588	15.307	37.75204	5.601141	4.373427	10.51081

146.076673	5.350794	607.2293	22.24283	50.84295	4.709548	6.355095	16.89204
135.183677	5.77708	1040.887	44.48235	97.83441	4.893967	12.70924	38.70527
164.673652	4.691557	242.4324	6.906906	16.81165	1.268978	1.973402	2.215349
161.486749	4.708068	954.3254	27.8229	67.33268	4.375884	7.949399	23.11483
154.017255	4.984377	584.6234	18.91985	44.60744	3.379584	5.405671	13.93547
135.306404	5.709131	667.2706	28.15488	62.12133	3.533	8.044251	22.44575
144.785611	5.323	652.8473	24.00174	54.81317	3.119794	6.85764	18.67874
163.306361	4.789043	1584.717	46.47263	112.3016	9.70395	13.2779	41.68359
171.538895	4.550103	830.6076	22.03203	54.59337	2.968834	6.294866	17.48193
149.093824	5.213071	455.1533	15.91445	36.80299	2.514622	4.546986	10.70138
174.554991	4.43033	601.8964	15.27656	38.27362	2.967123	4.364731	10.84623
154.27552	4.992735	777.4213	25.15927	59.31815	4.518723	7.188362	20.16653
135.14999	5.775641	409.1342	17.48437	38.45509	4.562781	4.995533	11.70873
142.396225	5.414305	528.6176	20.09953	45.51713	5.31571	5.742723	14.68522
168.882124	4.69117	602.4307	16.73419	40.99022	2.846551	4.781196	12.04302
145.929642	5.268218	396.6629	14.31996	32.852	3.52678	4.091418	9.051743
154.191393	5.410224	1246.456	43.73529	101.0516	6.832469	12.4958	38.32506
151.315443	5.625109	855.1544	31.79013	72.39863	5.651468	9.082893	26.16502
147.131734	5.637231	959.8239	36.77486	83.12108	6.115926	10.5071	31.13763
153.561269	5.426193	1080.478	38.17942	88.05945	5.221372	10.90841	32.75323
150.153313	5.540713	1097.017	40.48032	92.36049	6.717579	11.56581	34.93961
211.954596	4.33445	1563.809	31.97973	84.5672	4.193182	9.137065	27.64528
170.744057	4.96349	1108.965	32.23735	78.07266	4.310696	9.210672	27.27386
171.733381	4.992249	950.2538	27.62366	66.89918	4.204032	7.892474	22.63141
146.744232	5.600925	903.7051	34.49256	78.03703	4.847862	9.855017	28.89163
155.896496	5.413073	866.6507	30.09204	69.71075	4.215281	8.597725	24.67897
151.179766	5.107425	1360.239	45.95401	107.188	8.997491	13.12972	40.84658
147.467477	5.285573	1093.988	39.21102	90.11752	6.240681	11.20315	33.92545
145.098966	5.257209	968.2299	35.08079	80.40752	5.819382	10.02308	29.82358
177.364331	4.594931	1314.332	34.05005	84.87202	5.056088	9.728585	29.45512
171.057897	4.623186	907.7576	24.53399	60.50881	4.832085	7.009711	19.9108
146.491352	5.307658	1137.567	41.2162	94.47029	9.310896	11.77606	35.90855
157.034933	4.907342	1021.095	31.90921	75.89333	6.286588	9.116918	27.00187
148.655633	5.179639	597.9878	20.83581	48.22596	3.962391	5.953089	15.65617
166.840043	4.712996	741.7406	20.95312	51.10926	4.260422	5.986607	16.24013
163.158012	4.661657	1117.884	31.93956	77.68656	4.389029	9.125588	27.2779
151.270653	5.180502	732.578	25.08829	58.31989	4.020752	7.168082	19.90779
142.575887	5.421136	555.9089	21.13722	47.86707	2.995607	6.039206	15.71609
156.486035	4.967811	728.1039	23.11441	54.75962	4.007846	6.604117	18.1466
169.770944	5.007992	1262.855	37.25235	89.88838	5.571025	10.64353	32.24436
147.976554	5.605172	942.1338	35.68689	80.89276	6.447679	10.19625	30.08171
192.781069	4.568272	1372.09	32.51398	82.87019	6.652546	9.28971	27.94571
161.567989	5.297311	1043.49	34.21278	80.40134	4.57912	9.775079	28.91546
165.859657	5.010866	1015.081	30.66709	73.55795	5.083878	8.762026	25.65623
154.735489	5.39148	954.1887	33.24699	76.95252	4.784458	9.499141	27.85551
157.242023	5.348368	1042.95	35.47448	82.60435	7.514708	10.13557	30.12612
174.367458	4.911759	1006.559	28.35377	69.20985	4.504448	8.101078	23.44201

162.082284	5.228461	1113.277	35.91218	84.73877	4.450383	10.26062	30.68371
164.415637	5.137989	1009.087	31.53395	75.00081	4.381301	9.009701	26.39597
173.795061	4.93736	956.0717	27.16113	66.15817	3.815885	7.760323	22.22377
201.559045	4.459271	971.8131	21.50029	55.74795	3.539703	6.14294	17.04102
160.199906	4.914108	1190.994	36.53355	87.29638	7.306357	10.43816	31.61944
225.246	3.798415	1880.99	31.7199	88.02286	6.97865	9.062829	27.92149
184.226408	4.344962	969.8907	22.87478	58.37117	5.107972	6.535652	18.52982
186.762035	4.323195	1104.757	25.57309	65.56228	7.242442	7.306596	21.24989
170.226957	4.600729	969.9278	26.21426	64.65292	5.045428	7.48979	21.61354
182.690947	4.42351	931.3039	22.54973	57.16482	5.647217	6.44278	18.12622
194.794557	4.234664	746.1033	16.21964	42.24065	2.719128	4.634182	11.98497
176.498913	4.537247	988.3628	25.40778	63.4533	4.468797	7.259367	20.87054
194.613812	4.589948	1639.572	38.66915	98.67477	6.662493	11.04833	34.07921
228.95035	4.009638	1480.573	25.92947	71.27753	6.368313	7.408419	21.91983
180.210758	4.978198	1100.53	30.40137	74.57105	6.030839	8.686105	25.42317
183.812396	4.91477	1173.917	31.38814	77.62177	6.492297	8.968041	26.47337
190.451533	4.7376	1467.048	36.49373	91.89137	7.724217	10.42678	31.75613
171.036411	5.31169	1003.274	31.15759	74.22118	4.08601	8.902169	25.8459
168.370868	5.448895	1338.463	43.31595	102.1263	7.122414	12.37598	37.86705
221.117949	4.18784	1035.626	19.61413	52.87223	6.098638	5.604038	15.42629
193.190279	4.643997	1244.872	29.9248	75.99844	4.750769	8.549944	25.28081
201.867056	4.546555	1183.641	26.65859	68.81496	5.917645	7.616739	22.11203
187.992497	4.82032	761.1303	19.51616	48.77088	3.29703	5.576046	14.69584
174.769604	5.125208	839.9407	24.63169	59.52273	3.027044	7.037626	19.50648
198.072014	4.574411	854.0095	19.72308	50.59374	4.311611	5.635167	15.14867
162.512731	4.724207	1184.613	34.43643	83.3984	5.153033	9.83898	29.71222
168.266477	4.28159	1079.066	27.45715	68.74697	4.7207	7.844899	23.17556
184.885764	3.773179	1177.106	24.02258	63.55776	5.918033	6.863593	20.2494
172.111653	4.147269	1135.215	27.35457	69.42917	4.945611	7.815592	23.2073
190.43013	3.283278	916.0936	15.79472	43.58819	3.323812	4.512776	12.51144
163.673793	4.623553	1105.008	31.21491	76.14001	5.348041	8.918547	26.59136
179.058315	4.62683	893.1203	23.07804	57.56079	5.213458	6.593727	18.45121
162.93614	4.276539	712.8428	18.70978	46.48363	3.916566	5.345653	14.43324
181.33873	4.02975	1259.304	27.98454	72.48052	4.410712	7.995583	23.95479
223.316222	2.687319	1385.536	16.67312	50.34045	3.841467	4.763748	13.9858
169.72117	3.771582	1123.856	24.97458	64.68466	3.805585	7.135594	21.203
174.537252	4.040214	1259.563	29.15656	74.74932	5.552238	8.330446	25.11635
178.495634	5.013922	1077.197	30.25835	73.91077	4.883185	8.645242	25.24443
206.257855	4.426134	1371.969	29.44139	76.92274	4.111137	8.411825	25.01525
208.169353	4.429135	1103.565	23.4801	61.47867	8.092569	6.708601	19.05097
224.003629	4.218524	1196.397	22.53101	60.82111	5.638882	6.437432	18.31249
208.37586	4.424116	1093.032	23.20663	60.79491	4.055062	6.630464	18.78251
206.795261	4.390558	1145.253	24.31535	63.69945	4.552904	6.947242	19.92479
178.134338	5.193421	1049.787	30.60603	74.06799	3.855635	8.744581	25.41261
207.669506	4.34455	896.6382	18.75812	49.32264	2.811032	5.359463	14.41357
207.665842	4.514475	1006.424	21.87879	56.97873	3.20281	6.251083	17.36432
225.733077	4.141891	1137.737	20.8759	56.72107	2.809249	5.964544	16.73401

240.076031	3.948619	1570.03	25.82287	72.10749	4.788249	7.377963	21.87425
198.22611	4.653195	990.1698	23.24342	59.38168	4.306365	6.640978	18.59023
172.307014	4.418129	1176.927	30.17762	75.41386	6.893938	8.622178	25.75949
208.073623	3.394349	1802.142	29.39873	82.26096	5.570579	8.399639	26.00439
195.802523	3.599311	1284.232	23.6072	64.11272	4.263272	6.744914	20.00789
195.028366	3.618337	1206.515	22.38432	60.65145	9.023871	6.395521	18.76599
182.293535	3.997665	992.6034	21.76762	56.56556	4.885951	6.219319	17.76995
191.826874	3.539241	1060.665	19.56946	53.09806	4.507491	5.591274	16.03022
180.511968	3.932723	1151.143	25.07937	65.27845	10.25814	7.165534	21.14665
165.052354	4.210519	884.9744	22.57588	56.48929	4.057533	6.45025	18.36536
191.826527	3.506883	1480.137	27.05918	73.58877	5.691077	7.731195	23.5523
205.376183	3.383463	1218.509	20.07428	56.03213	4.598296	5.735508	16.69082
180.356581	3.870313	597.9878	12.83236	33.52763	3.395265	3.666388	8.962043
162.088974	5.195159	928.3104	29.75354	70.31974	3.907137	8.501011	24.55838
155.852621	4.652317	844.6075	25.21216	60.65566	5.41927	7.203475	20.55985
199.163426	4.620961	1496.818	34.72896	88.98383	6.349908	9.92256	30.108
227.956431	4.122178	1219.038	22.04408	60.11367	3.979066	6.298308	17.9219
210.734266	4.464709	1460.991	30.95319	81.13176	5.055849	8.843769	26.48848
236.047055	4.028107	1178.311	20.1077	55.63355	6.138174	5.745057	16.07959
198.725956	4.754209	1256.158	30.05162	76.41208	4.50129	8.586178	25.29741
196.02302	4.623184	1295.248	30.54831	77.95224	7.804559	8.728087	25.92512
181.267054	5.049222	981.3966	27.33695	66.91503	5.26343	7.810558	22.28773
191.460831	4.762707	1134.649	28.22511	71.07095	4.556609	8.064317	23.4624
221.538652	4.252181	1260.072	24.18564	64.97809	5.896599	6.910182	19.93346
258.547896	3.774422	1337.076	19.51935	56.15495	4.913924	5.576958	15.74493
197.480448	4.624835	656.2826	15.36962	39.28891	3.323279	4.391319	10.74478
236.679678	4.004732	829.7266	14.03937	38.92641	3.799574	4.011248	10.03463
218.705911	4.263273	1383.06	26.96024	72.15278	6.112338	7.702925	22.69697
217.193754	3.098342	1888.127	26.93476	77.93681	7.609784	7.695646	23.83642
214.315667	3.088122	1406.915	20.27255	58.51246	6.707882	5.792158	17.18443
212.93884	3.374625	1130.153	17.91051	50.47953	6.247274	5.117288	14.53588
177.789267	3.995264	962.715	21.63405	55.87631	5.845639	6.181156	17.63878
207.815108	3.362704	1075.718	17.40645	48.8042	3.311728	4.97327	14.04374
192.380409	3.411	1073.31	19.03033	52.1514	3.301831	5.437236	15.61933
191.015172	3.673369	1241.617	23.87726	64.11878	6.500098	6.822074	20.20389
199.609807	3.603065	1046.196	18.8844	51.52057	6.463109	5.395544	15.28134
174.138411	4.216426	1236.6	29.94189	75.90435	6.577043	8.554825	25.72546
225.809834	2.829697	1328.91	16.653	49.77294	4.139609	4.758001	13.82331
168.87331	4.31901	1022.181	26.14274	65.37254	3.777788	7.469355	21.82373
189.158861	3.908241	1209.481	24.98927	65.91206	5.338751	7.139791	21.08103
323.752638	3.348011	2288.127	23.66212	74.2011	7.042573	6.760607	20.31411
179.069905	5.044223	1178.525	33.19789	81.03404	8.432934	9.48511	28.15366
211.809078	4.394379	1008.563	20.92454	55.13377	6.564196	5.97844	16.53016
241.557221	4.005924	1191.625	19.76161	55.06829	5.20315	5.646173	15.75568
198.271358	4.610962	1200.585	27.92059	71.49764	6.149742	7.977311	23.30963
224.081024	4.172831	1204.19	22.42439	60.70357	7.312774	6.406969	18.25156
259.154313	3.77776	1301.383	18.9706	54.59617	4.122157	5.420172	15.19284

244.924398	3.969601	1622.189	26.29155	73.68642	5.662801	7.511871	22.32195
207.505806	4.424431	1265.994	26.99347	70.6402	3.742355	7.71242	22.56904
232.781036	4.083878	1189.003	20.8597	57.31611	3.966452	5.959914	16.77582
216.5016	4.28716	1130.245	22.38108	59.66285	3.973244	6.394595	18.09392
293.345666	3.504727	1554.845	18.5764	56.18793	5.764941	5.307544	15.07168
198.578523	3.718699	1611.714	30.18192	81.5891	9.813189	8.623405	26.46322
199.532981	3.661156	1238.443	22.72373	61.74171	6.191632	6.492493	19.06257
179.827614	3.892373	771.5762	16.70078	43.54089	5.159886	4.771652	12.80841
187.055277	3.921494	1045.374	21.91559	57.59474	4.571817	6.261598	17.9941
174.371317	4.305465	759.3628	18.7497	47.29966	3.398709	5.357056	14.44423
187.570572	3.81241	1261.917	25.64871	67.92925	3.940594	7.328203	21.8363
267.664488	2.457892	1555.367	14.28252	46.13834	3.871808	4.080721	11.82463
204.099362	3.453458	1304.177	22.06729	61.18513	5.541835	6.30494	18.61383
184.574644	3.626221	1315.767	25.85005	69.04636	5.03977	7.385727	22.22382
191.455029	3.783696	1276.289	25.2231	67.27226	4.272518	7.206599	21.4394
215.154713	3.100212	1615.387	23.27648	67.18264	6.034748	6.650422	20.17626
194.832658	3.424124	1293.783	22.73784	62.44925	4.954136	6.496525	19.31371
248.889081	3.919513	1984.363	31.24981	88.21467	8.281879	8.928517	27.3303
235.420373	4.065982	1663.185	28.72513	79.23766	6.027359	8.20718	24.65915
260.129716	3.76454	1950.234	28.22336	81.37259	7.1396	8.063816	24.45882
214.926942	4.272901	1839.458	36.56974	97.38989	6.772962	10.4485	32.29684
266.805191	3.675003	1329.073	18.30679	53.43753	4.981436	5.230512	14.63179
266.344486	3.761928	1866.131	26.35779	76.45701	4.893369	7.530797	22.59586
203.63931	4.525318	1389.761	30.88357	79.98906	6.824618	8.823876	26.35825
226.961985	4.141642	1362.624	24.8654	67.65356	4.328013	7.104401	20.72376
303.297821	3.454417	1515.197	17.25737	52.82607	4.882383	4.930678	13.80295
250.242381	3.903937	1367.414	21.3325	60.36099	4.159601	6.095001	17.42857
233.65109	4.084809	1224.121	21.40072	58.85418	6.715497	6.11449	17.31591
227.251827	4.109436	1641.48	29.68318	80.9453	7.223175	8.480907	25.57374
257.364764	3.824142	1617.899	24.04011	68.85574	3.870146	6.868602	20.21597
244.976809	3.919629	1384.134	22.14615	62.26847	6.06593	6.327471	18.22652
227.849222	2.848115	2056.182	25.70228	76.86775	6.681982	7.343509	22.85417
222.407637	3.10192	1363.182	19.0123	55.32412	6.129206	5.432087	15.91038
208.521253	3.098384	1001.634	14.88311	42.62826	6.371824	4.252318	11.78473
208.174322	3.202682	1574.781	24.22739	68.79154	8.132872	6.922112	21.02471
239.031922	2.575775	1621.028	17.46797	54.21625	6.499158	4.990849	14.8922
190.79559	3.655088	1151.018	22.05015	59.26922	6.032728	6.300043	18.39506
182.939996	4.148299	1413.219	32.04577	82.58104	5.94657	9.155935	27.89747
236.569124	2.66107	1622.265	18.2482	56.03321	4.661938	5.213771	15.58713
200.797518	3.637636	946.4183	17.14526	46.73356	3.694297	4.898646	13.50762
210.565499	3.480421	1205.719	19.92924	55.5814	4.564625	5.694068	16.44882
215.822965	2.928398	1981.806	26.89018	78.78808	5.95143	7.682908	23.96178
193.918521	3.624645	1237.059	23.12261	62.5353	6.379274	6.606459	19.49796
261.945997	3.769007	1919.439	27.61783	79.74176	7.784703	7.890809	23.84883
253.342951	3.867831	1331.721	20.33162	57.84054	5.256595	5.809035	16.46379
264.402063	3.734492	1771.287	25.01817	72.57113	6.303663	7.148049	21.28368
223.622762	4.211351	1227.58	23.11827	62.40638	5.49923	6.60522	18.90692

276.241953	3.629986	1270.473	16.69478	49.30908	3.921573	4.769938	13.0648
233.517786	4.082479	1199.126	20.96374	57.65246	4.636532	5.989641	16.88126
232.051448	4.056843	1359.368	23.76517	65.35668	5.858046	6.79005	19.70833
243.408091	3.919615	1372.535	22.102	62.04468	4.991566	6.314859	18.18239
252.502073	3.849117	1345.097	20.50452	58.35467	3.999398	5.858435	16.65541
286.368468	3.575137	1574.552	19.65733	58.8075	3.75112	5.616381	16.0822
274.25306	3.651838	1155.521	15.38643	45.29476	3.02309	4.396124	11.7346
277.883874	3.627727	1541.937	20.12972	59.55181	4.653462	5.751349	16.50199
191.287012	3.758095	1409.406	27.68971	73.96015	5.408921	7.911345	23.93161
195.401252	3.489308	1151.324	20.55937	56.24148	5.351624	5.874104	17.07006
189.868823	3.92291	1121.838	23.17847	61.13588	5.571352	6.622421	19.25556
204.047798	3.458437	1318.762	22.3519	61.94803	4.85466	6.386258	18.89346
223.733941	2.928455	1734.269	22.69985	67.11142	6.274878	6.485673	19.7714
246.681953	2.479216	1577.289	15.85215	50.06613	6.097126	4.529186	13.37294
214.49458	2.979091	1468.931	20.40183	59.42951	5.235927	5.829093	17.42273
228.676678	2.927998	1416.577	18.13799	53.92025	6.194672	5.182282	15.20999
205.58244	3.364688	1446.542	23.67499	66.19117	8.108382	6.764282	20.3103
216.666175	3.200387	1340.044	19.79386	56.77768	8.021225	5.655388	16.59347
213.183683	3.346683	1356.06	21.28823	60.14153	5.836333	6.082352	17.94155
269.185611	3.707791	1967.857	27.10547	79.12087	6.279625	7.744419	23.39768
267.237775	3.737591	1439.17	20.12825	58.53054	4.424211	5.750928	16.39066
297.554943	3.476109	1288.855	15.05671	45.79822	4.492347	4.301918	11.5806
261.108089	3.756951	1455.091	20.93656	60.45074	5.800517	5.981876	17.17961
248.776042	3.881062	1322.203	20.6272	58.3653	5.577663	5.893485	16.74614
267.925779	3.685361	1450.588	19.95307	58.26306	5.458616	5.700877	16.26771
249.417576	3.878967	1565.509	24.34695	68.94413	5.084935	6.956271	20.46798
289.829113	3.525902	1758.722	21.39564	64.42335	4.549027	6.113041	17.86974
319.101172	3.383894	1592.796	16.89073	52.63521	5.346779	4.825923	13.50684
242.642165	3.938996	1583.467	25.70563	72.01508	6.961313	7.344466	21.76663
203.608288	3.399137	1418.749	23.6853	65.89243	6.968033	6.767227	20.28616
206.600399	3.243334	1627.474	25.54904	72.17876	7.877399	7.299725	22.3057
192.450503	3.97625	1257.726	25.98606	68.54122	5.48694	7.42459	22.00981
193.029299	3.755434	1485.004	28.89113	77.35802	7.693154	8.254609	25.1357
218.79502	3.161778	1806.193	26.10106	75.28086	8.267053	7.457444	22.93928
217.424232	2.986597	1694.014	23.26942	67.97017	5.927264	6.648405	20.28282
185.260038	3.773117	997.7241	20.32025	53.78973	4.179695	5.805785	16.54713
210.396441	3.37715	1273.875	20.44742	57.44611	4.829308	5.842121	17.07027
214.239882	3.19761	1674.15	24.98731	71.48883	7.81437	7.139232	21.7897
195.130453	3.509541	1333.272	23.97971	65.4806	6.832721	6.851346	20.47017
221.581569	3.094715	1618.592	22.60603	65.75861	5.713672	6.458867	19.51132
202.922546	3.26242	1348.829	21.68536	60.89956	6.856108	6.195816	18.42294
197.156517	3.495683	1285.19	22.78705	62.44647	4.923981	6.510586	19.29137
302.331328	3.475073	1746.671	20.07668	61.3157	5.062979	5.736194	16.60161
223.184765	4.203103	1371.856	25.83533	69.74092	6.36083	7.381523	21.63223
301.396982	3.488391	1595.224	18.46324	56.29066	5.843206	5.275212	14.97485
243.600274	3.967431	641.5719	10.44905	29.24954	1.992413	2.985444	6.481623
266.892024	3.727542	1380.584	19.2819	56.08906	5.172819	5.509115	15.55436

240.317654	3.939634	1351.962	22.16331	61.93943	5.074383	6.332376	18.22368
267.006284	3.729138	1569.572	21.9214	63.76708	5.439439	6.263257	18.19226
244.900635	3.91841	1455.584	23.28935	65.48281	5.707495	6.654099	19.37094
227.587445	4.145491	1373.536	25.01887	68.10215	4.979357	7.148249	20.87338
268.822566	3.733647	1608.915	22.34604	65.09291	5.754277	6.384583	18.61239
259.159032	3.822405	1508.964	22.2561	63.86406	6.789473	6.358886	18.4337
232.760244	4.033973	1525.618	26.44052	72.87254	5.45914	7.554433	22.40654
302.994348	3.427538	1897.052	21.45986	65.80215	6.442813	6.13139	18.03233
262.253661	3.778871	1661.357	23.93886	69.09449	5.931465	6.839675	20.15999
220.033602	3.138853	1888.528	26.94048	77.95336	4.852161	7.69728	23.80163
217.541379	3.046798	1777.364	24.89305	72.3607	8.71179	7.112301	21.84626
195.261619	3.712198	1110.061	21.10383	56.83392	7.188621	6.029664	17.39163
205.981976	3.32229	1334.947	21.5314	60.41853	4.47403	6.151828	18.20911
202.80644	3.265804	1480.139	23.83476	66.90887	6.165444	6.809932	20.56896
197.903677	3.377196	1010.208	17.23904	47.6966	4.339384	4.925439	13.86184
213.570241	3.300931	1382.31	21.36491	60.59365	6.472389	6.10426	18.06398
187.301162	4.002162	1205.439	25.75724	67.36911	5.935701	7.359212	21.75508
194.910872	3.493026	1216.032	21.79268	59.56199	4.824847	6.22648	18.29966
195.4177	3.839248	1200.97	23.59471	63.02226	6.145659	6.741344	19.75546
196.318165	3.543649	1472.495	26.57934	72.51395	4.704747	7.594096	23.03569
212.020886	3.251854	1244.159	19.0822	54.22384	4.999106	5.452056	15.83034
201.737958	3.484248	939.2929	16.22268	44.74991	4.656005	4.63505	12.73843
187.452731	3.632805	1005.565	19.48769	52.23033	6.699762	5.567911	15.85488
209.076869	3.144013	1550.555	23.31662	66.58418	5.772336	6.661892	20.17261
204.58662	3.206687	1456.602	22.83075	64.52461	5.469233	6.523072	19.62406
114.767488	7.404354	149.774	9.662839	19.17289	1.305021	2.760811	2.258485
128.558156	6.427908	172.143	8.60715	18.20191	1.339028	2.459186	2.179242
120.926099	6.989948	169.75	9.812139	20.0113	1.40375	2.803468	2.822191
120.926099	6.989948	162.524	9.394451	19.15945	1.343994	2.684129	2.404503
151.454327	5.389834	193.923	6.901174	15.88911	1.280406	1.971764	1.511341
151.454327	5.389834	181.9	6.47331	14.904	1.201022	1.849517	1.083476
150.938907	5.409997	234.739	8.413584	19.33669	1.555192	2.403881	3.003588
131.952524	6.283454	210.154	10.00733	21.42265	1.592649	2.859238	3.72388
119.194861	6.374057	189.635	10.14091	21.08809	1.590966	2.897403	3.766852
112.790795	7.138658	148.059	9.370823	18.6828	1.312687	2.677378	2.232165
116.49212	6.694949	146.5	8.41954	17.19593	1.257596	2.405583	1.724591
165.986076	4.910831	227.545	6.732101	16.23229	1.370868	1.923457	1.82127
145.527747	5.684678	210.636	8.227969	18.50771	1.447394	2.350848	2.543291
194.013169	4.000272	217.446	4.483423	11.83164	1.12078	1.280978	0.483151
151.468554	4.91781	201.454	6.540714	15.40859	1.330005	1.868776	1.622904
133.742531	6.191784	147.15	6.8125	14.68656	1.100248	1.946429	0.620716
131.523507	6.353793	168.953	8.161981	17.40957	1.284584	2.331994	1.808188
130.10554	6.40914	168.5	8.300493	17.61886	1.295102	2.371569	1.891353
130.10554	6.40914	189.429	9.331478	19.80726	1.455964	2.666137	2.922338
144.196183	5.744868	204.45	8.145418	18.2319	1.41786	2.327262	2.40055
144.196183	5.744868	205.3	8.179283	18.30769	1.423755	2.336938	2.434415
222.0751	4.166512	271.125	5.086773	13.74435	1.220871	1.453364	0.920261

134.343229	6.10651	178.931	8.133227	17.61443	1.331894	2.323779	2.026717
133.433888	6.149027	179.63	8.27788	17.86628	1.34621	2.365109	2.128853
137.766273	5.989838	228.193	9.921435	21.72733	1.656378	2.834696	3.931597
157.636548	4.663803	208.083	6.156302	14.84394	1.320017	1.758943	1.492499
147.699329	5.023787	228.43	7.769728	18.09225	1.546588	2.219922	2.745941
178.592128	4.31382	242.019	5.84587	14.82857	1.355149	1.670248	1.53205
146.162813	5.092781	255.583	8.905331	20.61202	1.748619	2.54438	3.81255
140.364054	5.357407	228.579	8.724389	19.73833	1.628472	2.492683	3.366983
140.364054	5.357407	163.188	6.22855	14.09166	1.162605	1.779586	0.871143
171.487386	4.89964	225.333	6.438086	15.65935	1.313992	1.839453	1.538446
171.487386	4.89964	298.952	8.541486	20.77545	1.743289	2.440424	3.641846
218.795812	4.191491	246.833	4.728602	12.71014	1.128143	1.351029	0.537111
218.795812	4.191491	251.8	4.823755	12.96591	1.150845	1.378216	0.632264
176.52913	4.849701	222.519	6.113159	15.01554	1.260523	1.746617	1.263458
179.277911	4.780744	259.243	6.913147	17.10738	1.44604	1.975185	2.132402
221.718148	4.191269	288.687	5.457221	14.71755	1.302045	1.559206	1.265952
221.718148	4.191269	363.846	6.877996	18.54923	1.641029	1.965142	2.686727
173.522638	4.92962	226.588	6.437159	15.67942	1.305812	1.839188	1.507539
173.522638	4.92962	223.3	6.34375	15.45189	1.286864	1.8125	1.41413
158.887899	5.261189	220.389	7.297649	17.10743	1.387072	2.085043	2.03646
220.870941	3.827919	281.4	4.87695	13.44133	1.274047	1.393414	1.04903
220.870941	3.827919	289.923	5.024662	13.84844	1.312635	1.435618	1.196743
197.481586	4.030236	190.077	3.879122	10.26319	0.962505	1.108321	-0.15111
151.507251	5.242465	213.065	7.372491	17.0938	1.406302	2.106426	2.130026
184.074034	4.67193	250.105	6.347843	15.90377	1.35872	1.813669	1.675913
184.074034	4.67193	218	5.532995	13.86227	1.184306	1.580856	0.861065
162.682983	5.180987	315.867	10.05946	23.81261	1.941611	2.874131	4.878472
170.372392	4.996258	198.333	5.816217	14.05495	1.164115	1.661776	0.819959
170.372392	4.996258	193.5	5.674487	13.71245	1.135747	1.621282	0.678229
159.003353	5.300112	195.85	6.528333	15.27857	1.231735	1.865238	1.228222
159.003353	5.300112	196.45	6.548333	15.32537	1.235509	1.870952	1.248222
167.536458	5.031125	178.545	5.361712	12.87996	1.065708	1.531918	0.330587
167.536458	5.031125	176.158	5.29003	12.70777	1.051461	1.511437	0.258905
257.584721	3.885139	284.254	4.287391	12.23408	1.103536	1.224969	0.402252
172.203913	4.906094	260.344	7.417208	18.05374	1.511836	2.119202	2.511114
292.237432	3.727518	326.601	4.165829	12.39597	1.117588	1.190237	0.438311
180.966802	4.724982	209.882	5.479948	13.63252	1.159782	1.565699	0.754966
213.685751	4.28228	282.203	5.655371	15.03094	1.320645	1.61582	1.373091
226.836815	3.737015	301.053	4.959687	13.84368	1.327179	1.417053	1.222672
226.836815	3.737015	260.333	4.288847	11.9712	1.147666	1.225385	0.551832
182.51803	4.244605	228.5	5.313953	13.6077	1.251931	1.518272	1.069348
153.514327	5.117144	197.6	6.586667	15.41509	1.287176	1.881905	1.469522
153.514327	5.117144	176.211	5.8737	13.7465	1.147847	1.6782	0.756556
157.314865	4.870429	217.227	6.725294	16.03289	1.380842	1.921513	1.854865
189.383273	4.153142	232.134	5.090658	13.22864	1.225737	1.454474	0.937516
167.052601	4.679345	183	5.12605	12.52997	1.095463	1.464586	0.446706
245.816466	3.990527	305.2	4.954545	13.8803	1.241577	1.415584	0.964018

194.389483	4.531223	243.6	5.678322	14.5323	1.253154	1.622378	1.147098
194.389483	4.531223	223.6	5.212121	13.33917	1.150268	1.489177	0.680898
242.610713	3.832713	247.235	3.905766	11.01684	1.019061	1.115933	0.073054
242.610713	3.832713	249.167	3.936288	11.10293	1.027024	1.124654	0.103575
203.471042	4.357915	198.19	4.244806	11.09593	0.974045	1.212802	-0.11311
203.471042	4.357915	213.65	4.575926	11.96148	1.050027	1.307408	0.218012
198.063103	4.542732	205.333	4.709472	12.10163	1.036705	1.345564	0.166741
198.063103	4.542732	238.538	5.471055	14.05862	1.204354	1.563159	0.928323
214.610367	4.232946	254.455	5.018836	13.3923	1.18566	1.433953	0.78589
220.238457	4.06344	274.474	5.064096	13.74048	1.246258	1.446885	1.000656
209.538199	4.27629	243.048	4.960163	13.12336	1.159922	1.41719	0.683873
185.628677	4.910812	251.333	6.649021	16.48658	1.353956	1.89972	1.73821
185.628677	4.910812	259.05	6.853175	16.99279	1.395528	1.95805	1.942363
151.749277	4.84822	183.35	5.857827	13.85551	1.208243	1.673665	1.009608
151.749277	4.84822	176.25	5.63099	13.31898	1.161455	1.608854	0.782771
207.703998	2.841368	276.571	3.783461	11.06289	1.331563	1.080989	0.942093
195.320262	3.196731	311.018	5.090311	14.23163	1.592349	1.454375	1.89358
184.986116	3.477183	204.905	3.851598	10.40205	1.107678	1.100456	0.374415
168.265436	4.124153	211.273	5.17826	13.08726	1.255594	1.479503	1.054107
168.265436	4.124153	226.278	5.546029	14.01675	1.344768	1.58458	1.421877
276.14743	3.540352	369.667	4.739321	14.08444	1.338658	1.354092	1.198969
276.14743	3.540352	376.524	4.827231	14.3457	1.363489	1.379209	1.286879
293.69089	3.459257	419.863	4.945383	15.01159	1.429609	1.412967	1.486126
229.750171	4.023646	347.175	6.080123	16.71365	1.511098	1.737178	2.056477
205.372949	4.378954	280.533	5.981514	15.65324	1.365969	1.709004	1.60256
205.372949	4.378954	277.208	5.910618	15.46771	1.349779	1.688748	1.531664
263.736767	3.693792	275.2	3.854342	11.20404	1.043465	1.10124	0.160549
263.736767	3.693792	285.476	3.998263	11.6224	1.082428	1.142361	0.304471
190.445592	4.714	252.286	6.244703	15.74371	1.324714	1.784201	1.530703
190.445592	4.714	233.25	5.773515	14.55578	1.224759	1.649576	1.059515
164.791244	5.567272	207.632	7.014595	16.36159	1.25997	2.00417	1.447323
164.791244	5.567272	197.182	6.661554	15.53812	1.196556	1.903301	1.094282
194.742507	3.44068	292.127	5.161254	14.15661	1.500068	1.474644	1.720574
201.473544	3.011563	255.895	3.825037	10.93937	1.270117	1.092868	0.813475
173.480707	4.294077	194.222	4.807475	12.12027	1.11956	1.373564	0.513398
174.107944	3.627249	213.611	4.450229	11.71366	1.226888	1.271494	0.82298
174.107944	3.627249	204.526	4.260958	11.21547	1.174708	1.217417	0.63371
177.308047	3.567566	190.572	3.834447	10.18103	1.074807	1.095556	0.26688
181.592387	3.526066	248.064	4.816777	12.90352	1.366048	1.376222	1.290711
170.331474	4.279685	251.7	6.324121	15.8844	1.477707	1.806892	2.044435
170.331474	4.279685	255.941	6.430678	16.15205	1.502605	1.837337	2.150993
165.853508	4.364566	246.846	6.495947	16.12829	1.488338	1.855985	2.131381
165.853508	4.364566	248.619	6.542605	16.24414	1.499028	1.869316	2.178039
271.41857	3.618914	255	3.4	10.00563	0.939508	0.971429	-0.21891
271.41857	3.618914	281.227	3.749693	11.03471	1.036138	1.071341	0.130779
203.266826	4.418844	287.4	6.247826	16.27116	1.413905	1.785093	1.828982
203.266826	4.418844	248.65	5.405435	14.07732	1.223269	1.54441	0.986591

220.889429	4.183512	353.897	6.702595	18.06764	1.602145	1.915027	2.519083
317.918124	3.357108	395.316	4.174403	13.02213	1.243452	1.192687	0.817295
228.007621	4.108245	298.111	5.371369	14.66082	1.307461	1.534677	1.263124
228.007621	4.108245	292.158	5.264108	14.36806	1.281352	1.504031	1.155863
373.202248	3.14408	470.278	3.961904	13.07726	1.260116	1.131973	0.817824
236.389308	3.946399	293.053	4.892371	13.61056	1.239705	1.39782	0.945971
236.389308	3.946399	309	5.158598	14.3512	1.307166	1.473885	1.212199
298.6018	3.436154	326.6	3.758343	11.47496	1.093764	1.073812	0.322189
298.6018	3.436154	302.857	3.485121	10.64076	1.01425	0.995749	0.048967
179.299342	4.002217	239.738	5.351295	13.84453	1.337082	1.528941	1.349077
230.839599	2.674851	313.434	3.631912	11.06975	1.3578	1.037689	0.957061
179.119387	4.098842	178.9	4.093822	10.52566	0.998775	1.169663	-0.00502
179.119387	4.098842	202.588	4.635881	11.91935	1.131022	1.324537	0.537039
193.840307	3.550189	265.588	4.864249	13.22251	1.370138	1.389785	1.31406
193.840307	3.550189	260.458	4.770293	12.96711	1.343673	1.362941	1.220104
206.50782	3.086813	271.617	4.060045	11.61147	1.315287	1.160013	0.973231
184.898395	3.742882	260.695	5.277227	13.99064	1.409937	1.507779	1.534344
179.616222	5.031267	229	6.414566	15.67958	1.274941	1.832733	1.383299
179.616222	5.031267	223.3	6.254902	15.2893	1.243206	1.787115	1.223635
291.956722	3.463306	401.071	4.757663	14.41619	1.373734	1.359332	1.294357
291.956722	3.463306	396.625	4.704923	14.25638	1.358506	1.344264	1.241617
178.552764	5.015527	219.067	6.153567	15.03105	1.226903	1.758162	1.13804
176.325218	5.140677	189.316	5.519417	13.35724	1.073675	1.576976	0.37874
200.572107	4.51739	231.8	5.220721	13.47647	1.155694	1.491634	0.703331
200.572107	4.51739	234.92	5.290991	13.65786	1.17125	1.511712	0.773601
195.221978	4.670382	239	5.717703	14.53837	1.224247	1.63363	1.047321
195.221978	4.670382	235.385	5.63122	14.31847	1.20573	1.60892	0.960838
319.85469	3.314556	433.176	4.48887	14.06918	1.35429	1.282534	1.174314
319.85469	3.314556	371.565	3.850415	12.06811	1.161668	1.100118	0.535858
219.379063	4.139228	372.533	7.028925	18.96522	1.698125	2.008264	2.889697
219.379063	4.139228	321.316	6.062566	16.35782	1.464661	1.732162	1.923338
222.583067	4.264043	335.815	6.433238	17.29208	1.508718	1.838068	2.169194
237.698581	3.915957	334.273	5.506969	15.37127	1.406289	1.57342	1.591012
267.385403	3.698277	385.944	5.338091	15.56577	1.4434	1.525169	1.639815
267.385403	3.698277	353	4.882434	14.23708	1.320192	1.394981	1.184158
330.764425	3.252354	415.5	4.085546	12.97419	1.256181	1.167299	0.833191
213.345108	2.926545	304.506	4.177037	12.20535	1.427293	1.193439	1.250492
220.286218	2.820566	298.25	3.818822	11.35252	1.35392	1.091092	0.998256
220.286218	2.820566	265.714	3.402228	10.11407	1.206222	0.972065	0.581662
175.841764	4.176764	241.429	5.734656	14.60757	1.37299	1.638473	1.557892
175.841764	4.176764	201.9	4.795724	12.21588	1.148191	1.370207	0.61896
197.248749	3.673161	254.694	4.742905	12.8392	1.291233	1.355116	1.069744
223.783076	2.797288	261.629	3.270363	9.780665	1.169119	0.934389	0.473074
241.960879	2.557726	307.826	3.253975	10.14815	1.272214	0.929707	0.696249
241.960879	2.557726	368.72	3.897674	12.15565	1.523883	1.113621	1.339948
262.725026	3.742522	316.944	4.514872	13.06862	1.206372	1.289963	0.77235
262.725026	3.742522	315.368	4.492422	13.00364	1.200373	1.283549	0.7499

226.218072	4.173765	296.318	5.467122	14.83401	1.309878	1.562035	1.293357
306.156768	3.439964	339.7	3.816854	11.72339	1.109562	1.09053	0.37689
208.227704	4.374532	357.233	7.504895	19.71271	1.715588	2.144256	3.130363
262.942193	3.79426	323	4.660895	13.44784	1.228407	1.331684	0.866635
262.942193	3.79426	329.8	4.759019	13.73095	1.254268	1.35972	0.964759
340.51532	3.286827	409.574	3.953417	12.61283	1.202806	1.129548	0.66659
176.62647	3.839706	201.55	4.381522	11.41076	1.141109	1.251863	0.541816
244.463998	2.541206	306.3	3.183992	9.971619	1.252945	0.909712	0.642786
202.524052	3.38669	283.588	4.742274	13.18748	1.400268	1.354935	1.355584
202.524052	3.38669	271	4.531773	12.60211	1.338113	1.294792	1.145083
191.647961	3.714108	246.051	4.76843	12.78021	1.28387	1.362409	1.054322
222.652481	3.079564	278.091	3.846349	11.21588	1.248991	1.098957	0.766785
222.652481	3.079564	261.81	3.621162	10.55924	1.175868	1.034618	0.541598
270.732384	2.352149	320.364	2.783354	9.116691	1.183324	0.795244	0.431204
186.423182	3.958029	256.896	5.454268	14.28867	1.378026	1.558362	1.496238
214.842899	2.971548	270.922	3.747192	10.92674	1.261024	1.070626	0.775645
243.991273	3.954478	240.053	3.890648	10.90419	0.983859	1.111614	-0.06383
339.642295	3.281568	413.727	3.997362	12.74995	1.218126	1.142104	0.715794
339.642295	3.281568	411.632	3.977121	12.68539	1.211957	1.13632	0.695553
240.736608	3.946502	327.468	5.368328	15.00277	1.360275	1.533808	1.421826
332.850284	3.263238	348.882	3.420412	10.86997	1.048165	0.977261	0.157174
268.55718	3.699135	444.118	6.117328	17.85648	1.653719	1.747808	2.418193
247.475153	3.848758	326.786	5.082208	14.39147	1.32048	1.45206	1.23345
247.475153	3.848758	342	5.318818	15.06149	1.381957	1.519662	1.47006
242.044659	3.954978	327.957	5.358775	14.98833	1.354944	1.531078	1.403796
242.044659	3.954978	275.524	4.502026	12.59203	1.138319	1.286293	0.547048
313.051852	3.373404	359.212	3.870819	12.01406	1.147452	1.105948	0.497415
242.374337	3.97335	308.263	5.053492	14.1229	1.271847	1.443855	1.080142
242.374337	3.97335	303.667	4.978148	13.91234	1.252884	1.422328	1.004798
192.667655	3.726647	226.238	4.375977	11.73404	1.17424	1.250279	0.64933
192.667655	3.726647	251.571	4.865977	13.04796	1.305725	1.390279	1.13933
251.530029	2.341993	313.25	2.916667	9.389401	1.245378	0.833333	0.574674
251.530029	2.341993	368.588	3.431918	11.04811	1.465384	0.980548	1.089925
204.639225	3.279475	281.281	4.507708	12.66928	1.374521	1.287917	1.228234
189.633834	3.632832	230.625	4.418103	11.87554	1.21616	1.262315	0.785271
189.633834	3.632832	203.65	3.901341	10.48652	1.073912	1.114669	0.268509
243.615445	2.668296	307.143	3.364107	10.3989	1.26077	0.961174	0.695811
242.478256	2.604493	310.79	3.338238	10.36943	1.281723	0.953782	0.733746
341.991263	3.250868	391.245	3.719059	11.91069	1.14402	1.062588	0.468191
264.655568	3.748662	312.722	4.42949	12.8397	1.181619	1.265569	0.680828
233.898653	4.089137	244.417	4.273024	11.75126	1.04497	1.220864	0.183887
233.898653	4.089137	245.864	4.298322	11.82083	1.051156	1.228092	0.209184
380.344896	3.104856	475.267	3.879731	12.90731	1.249568	1.108494	0.774874
380.344896	3.104856	434.684	3.548441	11.80515	1.142868	1.01384	0.443585
279.473384	2.136647	346	2.64526	8.945823	1.238043	0.755789	0.508613
279.473384	2.136647	365.611	2.795191	9.452865	1.308214	0.798626	0.658544
229.28143	2.834134	303.333	3.749481	11.24497	1.322972	1.07128	0.915347

229.28143	2.834134	289.381	3.577021	10.72775	1.262121	1.022006	0.742887
189.675544	3.959823	204.951	4.278727	11.25637	1.080535	1.222493	0.318903
282.814355	3.658659	310.7	4.019405	11.91809	1.098601	1.148401	0.360746
282.814355	3.658659	356.333	4.609741	13.66852	1.259954	1.317069	0.951082
262.622647	3.762502	324.778	4.65298	13.44916	1.236672	1.329423	0.890478
262.622647	3.762502	348.429	4.991819	14.42855	1.326729	1.426234	1.229317
280.092904	3.656565	411.568	5.37295	15.89535	1.469398	1.535129	1.716385
271.727983	3.662102	327.933	4.419582	12.97126	1.206843	1.262738	0.75748
253.187634	3.859568	285.579	4.353338	12.38935	1.127934	1.243811	0.493771
253.187634	3.859568	335.4	5.112805	14.55074	1.324709	1.460801	1.253237
358.923586	3.181947	324.5	2.876773	9.37525	0.904092	0.821935	-0.30517
358.923586	3.181947	314.684	2.789752	9.091653	0.876744	0.797072	-0.39219
195.145513	3.717057	248.176	4.727162	12.72449	1.271748	1.350618	1.010105
195.145513	3.717057	248.8	4.739048	12.75648	1.274946	1.354014	1.02199
188.382969	3.737757	207.733	4.121687	10.98203	1.102716	1.177625	0.383929
186.784826	3.991129	235.15	5.024573	13.14197	1.258935	1.435592	1.033444
186.784826	3.991129	232.65	4.971154	13.00225	1.245551	1.42033	0.980025
240.762966	2.648657	303.947	3.343751	10.32464	1.262433	0.955358	0.695094
240.762966	2.648657	313.273	3.446348	10.64143	1.301168	0.984671	0.79769
215.370824	2.902572	258.225	3.480121	10.21399	1.198979	0.99432	0.57755
217.539297	2.923915	267.476	3.595108	10.55857	1.229553	1.027174	0.671192
217.539297	2.923915	268.3	3.606183	10.5911	1.23334	1.030338	0.682268
228.183522	2.786124	218.118	2.663223	8.011772	0.955888	0.760921	-0.1229
343.655198	3.254311	356.206	3.373163	10.81318	1.036521	0.963761	0.118852
298.877318	3.451239	341.538	3.943857	12.03097	1.142736	1.126816	0.492618
298.877318	3.451239	359.7	4.15358	12.67074	1.203504	1.186737	0.70234
193.53231	3.721775	228.867	4.401288	11.819	1.182578	1.257511	0.679513
232.543299	2.685257	286.722	3.310878	10.10003	1.232983	0.945965	0.62562
184.098282	3.749456	219.75	4.47556	11.84726	1.193656	1.278731	0.726104
131.225032	6.239007	306.08	14.55237	31.16442	1.478647	4.15782	8.313363
125.652752	6.293021	172.92	8.660289	18.30672	0.832347	2.474368	2.367268
112.862778	7.28147	128.17	8.269032	16.40732	0.919902	2.362581	0.987563
138.309994	5.936051	206.58	8.866094	19.47921	1.303344	2.53317	2.930043
127.033029	6.514514	215.08	11.02974	23.1779	1.01611	3.151355	4.515229
136.510756	6.040299	201.58	8.919469	19.4476	1.368221	2.54842	2.87917
132.945081	6.212387	215.92	10.08972	21.70114	1.624129	2.882777	3.877333
150.22095	5.517758	230.67	8.472727	19.35375	1.146186	2.420779	2.95497
120.467547	6.442115	172.42	9.220321	19.17372	0.961468	2.634377	2.778206
152.726621	4.830522	230.83	7.300819	17.31218	1.084982	2.085948	2.470297
134.610211	5.679756	189.42	7.992405	17.63456	1.317704	2.283544	2.312649
135.553177	5.695512	177.42	7.454622	16.46531	0.907148	2.129892	1.75911
148.2506	5.180508	201.08	7.026593	16.25177	1.37726	2.007598	1.846084
137.42327	5.655279	202.83	8.346914	18.53222	1.277026	2.384832	2.691635
138.975887	5.649426	197.92	8.045528	17.91795	1.172859	2.298722	2.396102
138.721557	5.909332	248.08	10.56784	23.26149	1.016721	3.019382	4.658507
148.948917	5.49627	219.75	8.108856	18.50129	1.198658	2.316816	2.612586
143.991703	5.744273	244.17	9.740695	21.79543	1.376226	2.783056	3.996421

138.245848	5.958873	222.58	9.593966	21.05572	1.141904	2.741133	3.635093
188.107499	4.57961	235.5	5.733414	14.51469	1.103458	1.638118	1.153804
149.572809	5.529494	242.33	8.958595	20.43063	1.553397	2.559599	3.429101
129.561069	6.413914	251.42	12.44653	26.38677	1.328507	3.556153	6.03262
229.293976	4.117845	356	6.393334	17.46458	1.440829	1.826667	2.275488
142.628229	5.809704	245.83	10.01344	22.28929	1.400103	2.860983	4.203738
153.73806	5.381666	212.25	7.429902	17.17708	1.314893	2.122829	2.048235
150.82938	5.489695	285.42	10.38835	23.78379	1.652214	2.968101	4.898658
163.440276	5.091597	258.17	8.042679	19.14375	1.215375	2.297908	2.951082
141.162708	5.436235	234.25	9.021065	20.36401	1.096317	2.577447	3.58483
127.963273	5.993596	237.08	11.10445	23.86968	1.186408	3.1727	5.110854
140.08276	5.544099	576.5	22.81632	51.15452	2.915297	6.518949	17.27222
129.838585	6.006318	211.42	9.780266	21.08869	1.110808	2.794362	3.773947
153.973089	5.405789	227.83	7.998806	18.47871	1.281744	2.285373	2.593017
164.102754	5.084516	260.58	8.073741	19.24382	1.496898	2.306783	2.989225
257.803164	3.880649	205.83	3.09831	8.845467	1.061417	0.885231	-0.78234
149.103028	5.563546	197.17	7.35709	16.73939	1.066764	2.102026	1.793544
151.770889	5.535649	197.5	7.20356	16.4836	1.190333	2.05816	1.667911
148.928487	5.602396	230.25	8.661551	19.66739	1.409723	2.474729	3.059155
175.372551	4.861332	258.75	7.172557	17.57825	1.188289	2.049302	2.311225
169.195547	5.013201	214.92	6.368	15.3487	1.229871	1.819429	1.354799
156.702661	5.317903	240.75	8.170156	19.0355	1.243672	2.33433	2.852253
162.909392	5.163531	288.25	9.136292	21.65309	1.679974	2.610369	3.972761
137.841503	5.580628	213.25	8.633603	19.24715	1.343307	2.466744	3.052976
139.437492	5.551519	167.33	6.662022	14.91413	0.985744	1.903435	1.110503
136.355385	5.669663	184.33	7.664449	16.97304	0.944894	2.189843	1.994786
155.498682	4.923493	287.42	9.100465	21.57381	1.027124	2.600133	4.176972
146.475821	5.130502	186.42	6.529597	15.09343	1.326078	1.865599	1.399096
160.774817	4.842615	189.33	5.702711	13.68882	1.106675	1.629346	0.860096
149.426896	5.157097	217.67	7.512338	17.42934	1.249756	2.146382	2.355241
156.653677	4.926216	256.42	8.063522	19.14836	1.339567	2.303863	3.137306
131.032932	6.08974	184.5	8.574615	18.46758	1.39953	2.44989	2.484876
236.08338	3.724711	436.58	6.887967	19.43499	1.898834	1.967991	3.163255
133.293582	5.884926	280.58	12.38764	27.02437	1.422459	3.539325	6.502712
156.723468	4.892563	259.58	8.103518	19.27849	1.552791	2.315291	3.210955
154.797533	4.940399	227.08	7.247311	17.14658	1.41996	2.07066	2.306912
172.036285	4.93294	250.33	7.177921	17.44324	0.989134	2.050835	2.244981
185.204492	4.674874	273.08	6.893	17.29333	1.338627	1.969429	2.218126
163.617813	5.129085	247.67	7.76395	18.45145	1.336373	2.218271	2.634865
162.756174	5.142375	224.5	7.093207	16.82426	0.811436	2.026631	1.950832
182.625586	4.740444	294.17	7.635821	19.02354	0.959521	2.181663	2.895377
149.254256	5.543334	191.08	7.09675	16.16585	1.076507	2.027643	1.553417
175.857865	4.925991	285.75	8.004202	19.56524	1.310299	2.286915	3.078211
166.622573	5.056831	281.42	8.540819	20.46272	1.130201	2.440234	3.483989
178.043597	4.828301	357.83	9.703864	23.91265	1.153063	2.772533	4.875563
158.210113	4.870551	270.83	8.337592	19.90466	1.241884	2.382169	3.467041
181.195391	4.343442	282.33	6.767745	17.19975	0.808575	1.933641	2.424302

163.107795	4.769234	233.92	6.839766	16.54047	1.03886	1.954219	2.070532
175.50344	4.394948	248	6.210402	15.61179	0.997667	1.774401	1.815455
154.513865	4.997699	194	6.274865	14.79632	1.186544	1.792819	1.277166
158.286299	4.850498	175.25	5.370331	12.83557	1.093058	1.53438	0.519833
231.095685	3.748511	324	5.255474	14.72635	1.036535	1.501564	1.506964
153.196121	5.043494	361.75	11.90947	27.95898	1.572826	3.402704	6.865971
154.882145	5.028641	199.92	6.490909	15.29126	1.214728	1.854545	1.462268
150.975432	5.139589	187.92	6.397277	14.89325	0.902724	1.827793	1.257687
147.623891	5.23489	224.67	7.967021	18.35939	1.174254	2.276292	2.732132
184.453728	4.34776	220.92	5.207307	13.28981	1.062984	1.487802	0.859547
191.621382	4.158179	255.83	5.551505	14.46425	1.010866	1.586144	1.393325
160.485403	4.831424	223.33	6.723365	16.14086	1.096529	1.920961	1.891941
171.920178	5.225537	263.67	8.014286	19.19393	1.279455	2.289796	2.788748
202.898097	4.447861	317.75	6.965605	18.10258	1.201323	1.990173	2.517743
195.031801	4.663936	284.58	6.805366	17.30572	0.932529	1.94439	2.14143
156.648556	5.922441	253.75	9.593573	21.75639	1.18669	2.741021	3.671132
182.894255	5.000526	459.58	12.56541	30.90102	1.361033	3.590118	7.564887
213.170411	4.240763	337	6.7042	17.85121	1.250464	1.915486	2.463437
193.425084	4.649641	312	7.5	19.04735	1.50601	2.142857	2.850359
183.665118	4.795434	220.75	5.763708	14.33843	0.845526	1.646774	0.968274
169.501588	5.415386	306.42	9.789776	23.15575	1.081495	2.797079	4.37439
173.206348	5.268634	235	7.148289	17.11664	1.032604	2.042368	1.879655
178.013252	5.120179	250.5	7.205108	17.49574	1.407936	2.058602	2.08493
178.664054	5.004595	290.58	8.139496	19.89594	1.397019	2.32557	3.1349
194.730435	4.555098	256	5.988304	15.31221	0.897994	1.710944	1.433206
225.114198	3.794081	250.08	4.214855	11.69787	0.898502	1.204244	0.420774
162.17521	4.433076	237.75	6.49892	15.9831	1.104119	1.856834	2.065845
157.020172	4.557915	210	6.095791	14.76819	1.026493	1.741655	1.537876
161.831819	4.205281	271.33	7.050646	17.56087	1.723606	2.01447	2.845365
160.096518	4.833102	233.33	7.043925	16.89871	1.287338	2.01255	2.210822
141.375579	5.882561	170.17	7.080681	15.67751	1.013399	2.023052	1.19812
150.402986	5.469199	187.92	6.833455	15.64853	1.10704	1.952416	1.364255
185.975968	3.574193	275.83	5.301059	14.23745	1.411473	1.514588	1.726866
190.674529	3.37776	304.25	5.389725	14.77347	1.04582	1.539922	2.011966
161.21654	4.528554	238.92	6.711236	16.39325	1.06267	1.917496	2.182681
185.515638	4.831136	265.92	6.925	17.23862	1.069025	1.978571	2.093864
185.792642	4.882855	268.08	7.045466	17.4984	1.188825	2.01299	2.162611
216.887704	4.136473	356.83	6.805447	18.31293	1.662923	1.944413	2.668974
239.840428	3.915762	335	5.469388	15.30084	1.256234	1.562682	1.553626
180.118425	5.014852	297.92	8.294679	20.30601	1.186932	2.369908	3.279828
168.865864	5.408034	224.33	7.184307	16.98285	0.749215	2.052659	1.776273
194.591056	4.575921	296.92	6.982246	17.8302	1.245365	1.994927	2.406324
195.390398	4.663255	266.25	6.354415	16.16699	1.238833	1.815547	1.69116
175.937533	5.159458	212.25	6.22434	15.04118	0.955006	1.778383	1.064882
176.811435	5.110157	302.92	8.754913	21.23347	0.99589	2.501404	3.644756
173.552921	5.165266	222.75	6.629464	15.96112	0.812719	1.894133	1.464199
207.953064	4.305446	206.75	4.280538	11.28457	0.740164	1.223011	-0.02491

148.471382	5.027304	203.83	6.901771	16.08931	1.07375	1.971935	1.874466
217.756525	2.826795	299.58	3.888983	11.5214	1.11888	1.111138	1.062187
203.73784	3.189634	290.58	4.549198	12.86079	1.207831	1.299771	1.359564
185.912936	3.556441	317.5	6.073649	16.33139	1.471065	1.735328	2.517208
192.807635	3.531275	269.83	4.941941	13.4337	1.007129	1.411983	1.410666
149.333039	5.295498	183.08	6.492199	14.96077	1.167974	1.854914	1.196701
179.79542	4.152319	298.92	6.903464	17.7088	1.144191	1.972418	2.751145
172.576349	4.102416	245.92	5.845912	14.88805	1.016198	1.670261	1.743496
204.958319	3.354473	292.67	4.790016	13.39206	1.358602	1.368576	1.435543
201.699296	3.455708	307.08	5.261192	14.54206	1.084782	1.503198	1.805484
242.301959	3.878383	384.92	6.161184	17.32171	1.06626	1.760338	2.282802
275.926731	3.593966	382.92	4.987561	14.76363	1.195392	1.425017	1.393595
160.441495	5.736815	273.08	9.764365	22.45461	1.450008	2.789819	4.027551
185.804266	4.93727	233.92	6.215821	15.39539	0.865569	1.775949	1.278552
193.056639	4.739563	209.42	5.141286	12.98848	1.065751	1.468939	0.401722
227.884273	4.023878	357.42	6.311161	17.31317	1.365084	1.803189	2.287284
182.95186	5.019255	255.58	7.011797	17.22875	1.089893	2.003371	1.992542
162.231137	5.636353	247.92	8.613418	19.95076	1.372226	2.460976	2.977065
280.232835	3.548733	394.17	4.991579	14.87988	1.292022	1.426165	1.442845
219.887644	4.16983	385.75	7.315154	19.71261	1.308736	2.090044	3.145324
253.634278	3.845857	393.33	5.964064	16.99597	1.269831	1.704018	2.118207
212.442665	4.310668	329.17	6.679179	17.69691	1.002558	1.908337	2.368511
211.953867	4.345543	356.08	7.300461	19.29299	1.27058	2.085846	2.954918
186.159241	3.763302	275.08	5.560879	14.74763	1.237705	1.588823	1.797577
194.269713	3.868605	290.92	5.793257	15.42182	1.072516	1.655216	1.924653
165.895044	4.886452	203.08	5.981738	14.43901	0.987407	1.709068	1.095286
188.851085	3.671788	306.25	5.954348	15.94574	1.564496	1.701242	2.28256
186.976717	3.792631	357.17	7.244828	19.19728	1.38304	2.069951	3.452196
184.082111	3.939018	250.33	5.3566	14.00539	0.935219	1.530457	1.417583
187.035117	3.828764	323.67	6.625793	17.51677	1.052312	1.893084	2.797029
172.833108	4.241303	218.25	5.355828	13.53189	1.010791	1.530237	1.114525
183.684359	3.909592	247.33	5.264245	13.78229	0.911547	1.50407	1.354653
184.547821	3.68234	197.25	3.93579	10.47197	1.182341	1.124512	0.25345
174.833487	4.170945	275.58	6.574421	16.72842	1.172681	1.878406	2.403476
196.524127	3.810453	318.83	6.181871	16.56646	1.385194	1.766249	2.371418
195.381188	3.267244	295.75	4.945652	13.75305	1.277979	1.413043	1.678408
208.64483	4.397151	332	6.996839	18.36373	1.242654	1.999097	2.599687
205.251048	4.474137	238.92	5.208065	13.55409	0.985928	1.488019	0.733928
217.607937	4.240465	262.83	5.121695	13.70815	0.968959	1.463341	0.88123
244.552522	3.928555	351	5.638554	15.83808	1.363107	1.611015	1.71
212.263891	4.396973	334.25	6.923874	18.25071	1.198372	1.97825	2.5269
225.05075	4.186991	392.42	7.300837	19.76821	1.51175	2.085953	3.113847
175.581306	5.210128	276.83	8.21454	19.79205	1.23403	2.347011	3.004412
231.778553	4.105909	326.33	5.780868	15.84561	0.997951	1.651677	1.674959
252.507493	3.823959	368.33	5.577969	15.9007	1.380806	1.593705	1.754009
211.056254	4.406185	400.33	8.35762	21.98703	1.242952	2.387891	3.951435
187.368653	4.86356	281.33	7.302531	18.1932	1.232552	2.086437	2.438971

201.012197	4.578866	286.33	6.522323	16.78875	1.233756	1.863521	1.943458
292.779699	3.487549	325.33	3.875283	11.73029	0.885059	1.107224	0.387734
248.234535	3.876666	402.75	6.289726	17.79232	1.195991	1.797064	2.41306
241.683449	3.882465	363	5.831325	16.37956	0.786992	1.666093	1.94886
218.099251	3.238296	360.58	5.353823	15.33728	1.412212	1.529664	2.115527
187.620576	3.804379	261.42	5.300809	14.04725	1.170083	1.514517	1.49643
190.80057	3.840978	290.67	5.851434	15.53448	1.066014	1.671838	2.010457
186.17938	4.13732	294	6.533333	16.92146	1.019064	1.866667	2.396014
190.112104	3.554627	233.25	4.361199	11.79396	1.241814	1.246057	0.806572
199.979126	3.341339	281	4.695071	13.05895	1.011082	1.341449	1.353732
228.518147	2.857084	266.67	3.334083	9.970706	1.012991	0.952595	0.477
195.686145	3.614914	291.42	5.383408	14.60235	1.302902	1.538116	1.768493
190.694219	3.61047	236.67	4.480944	12.07989	1.088538	1.28027	0.870473
210.675255	4.36859	310.17	6.431726	16.94906	1.191449	1.837636	2.063136
230.807695	4.068315	274.58	4.839864	13.28288	0.962864	1.382818	0.771549
218.55862	4.254845	358	6.969455	18.65821	1.172809	1.991273	2.71461
222.67269	4.173809	391.67	7.341518	19.84127	1.432066	2.097577	3.16771
267.453193	3.751097	466.75	6.546283	19.02248	1.464314	1.870367	2.795187
344.174438	3.231685	430.83	4.045352	12.99552	1.280327	1.155815	0.813667
259.208736	3.809092	407.58	5.98942	17.20251	1.076119	1.711263	2.180327
280.265251	3.600067	372.33	4.782659	14.2064	1.117001	1.366474	1.182592
325.77097	3.346389	459.42	4.71926	14.82374	1.30517	1.34836	1.372871
196.066885	3.911559	286.92	5.72409	15.23071	1.348625	1.635454	1.812531
182.910449	4.080545	250.33	5.584607	14.45016	0.985241	1.595602	1.504061
188.204871	3.6705	304.58	5.940127	15.89542	1.369391	1.697179	2.269627
180.877921	3.940695	246.5	5.37037	13.9784	1.388732	1.534392	1.429675
211.62437	3.055948	292.92	4.229892	12.20209	1.140121	1.20854	1.173944
198.747033	3.374313	186.67	3.16927	8.779869	1.001825	0.905506	-0.20504
191.446584	4.029012	256.42	5.396384	14.16821	1.115257	1.541824	1.367372
236.505698	2.726825	337.08	3.88641	11.86027	0.991179	1.110403	1.159585
204.640391	3.736564	392.67	7.169829	19.50467	1.379387	2.048522	3.433265
208.090192	3.429587	362.08	5.967532	16.6551	1.217076	1.705009	2.537945
213.500147	2.936728	261.67	3.599312	10.51001	0.852818	1.028375	0.662584
279.371956	3.590115	422.58	5.430433	16.12884	1.373886	1.551552	1.840318
265.688711	3.72113	427.17	5.982773	17.3911	1.104027	1.709364	2.261643
371.972382	3.17518	454.58	3.880324	12.76596	1.232492	1.108664	0.705144
214.319779	3.145978	353.83	5.193835	14.92158	1.652793	1.483953	2.047856
197.639072	3.665073	291.33	5.402503	14.64005	1.099027	1.543572	1.73743
202.199682	3.280705	245.83	3.98861	11.1757	1.022885	1.139603	0.707905
266.878921	3.7378	465	6.512605	18.93125	1.827687	1.860744	2.774805
238.566192	3.996084	451.83	7.568342	21.03751	1.248425	2.162383	3.572258
197.516304	3.649262	339.33	6.269376	17.00489	1.409939	1.79125	2.620114
205.473961	3.308759	302.42	4.869887	13.67073	1.037746	1.391396	1.561128
214.298455	3.126938	284.92	4.157413	11.96184	1.355149	1.187832	1.030475
201.252949	4.452499	236.0154	5.22158	13.539	0.918065	1.49188	0.769081
181.903868	4.929644	318.5085	8.631667	21.2741	1.189651	2.46619	3.702023
169.038607	5.366305	325.8531	10.34454	24.50693	1.589595	2.955584	4.978238

197.913726	4.549741	279.0835	6.415713	16.47659	1.689269	1.833061	1.865972
284.538029	3.465749	281.3552	3.426982	10.31567	0.624927	0.979138	-0.03877
246.067694	3.868989	302.9338	4.76311	13.45101	1.042129	1.360889	0.894121
214.868063	4.213099	334.9025	6.566716	17.54853	1.516828	1.876204	2.353616
162.318064	4.521395	257.1072	7.16176	17.53046	1.14905	2.046217	2.640366
184.748213	4.336813	127.7401	2.998594	7.660728	0.87638	0.856741	-1.33822
158.912051	4.772134	233.8336	7.02203	16.86839	1.02219	2.006294	2.249896
280.843451	3.559486	627.9785	7.95917	23.72122	1.673226	2.274049	4.399684
234.586657	4.016895	374.7432	6.416836	17.7388	1.017308	1.833382	2.399941
282.845446	3.531154	522.3761	6.521549	19.51007	1.030482	1.8633	2.990395
212.398894	4.290887	431.4989	8.717149	23.12202	1.518298	2.490614	4.426263
235.349613	3.988976	206.0145	3.491771	9.677402	1.19917	0.997649	-0.49721
225.261185	4.095658	339.0747	6.164995	16.78894	1.02821	1.761427	2.069337
265.2204	3.678508	310.9196	4.312338	12.56599	1.521887	1.232097	0.633831
225.877735	4.077215	395.3314	7.135946	19.46834	1.67677	2.038842	3.05873
302.683714	3.416295	372.0988	4.199761	12.88496	0.938391	1.199932	0.783466
283.013866	3.528851	330.6468	4.122778	12.33768	1.474468	1.177937	0.593927
280.143566	3.586985	344.6215	4.412567	13.11759	0.896091	1.260733	0.825582
191.248078	3.735314	297.2897	5.806439	15.532	1.448248	1.658983	2.071125
178.578831	4.313498	245.3187	5.925572	15.03074	1.871393	1.693021	1.612074
159.570795	4.879841	162.6295	4.973379	11.89292	0.939606	1.420965	0.093538
230.065095	2.665876	455.9345	5.283134	16.10254	1.108057	1.509467	2.617258
223.690843	2.704847	542.1219	6.555283	19.76823	1.863529	1.872938	3.850436
181.053218	3.835873	228.1918	4.834572	12.67195	1.609011	1.381306	0.998699
232.14411	2.724696	334.1501	3.92195	11.91549	0.911623	1.120557	1.197253
183.09226	3.822385	406.8242	8.493198	22.3437	2.181902	2.426628	4.670813
184.446948	3.771921	188.261	3.849918	10.18073	0.554024	1.099977	0.077997
245.207772	3.849416	369.5833	5.801936	16.39109	1.881777	1.657696	1.95252
216.358164	4.309924	357.3538	7.118602	18.94832	0.911822	2.033886	2.808678
243.884175	3.927281	336.2582	5.414786	15.20037	1.936581	1.547082	1.487504
205.38658	4.484423	319.3673	6.973085	18.14017	1.251973	1.99231	2.488662
210.282638	4.353678	348.631	7.218033	19.02855	0.832614	2.062295	2.864355
210.238278	3.137885	295.8451	4.415599	12.63305	0.950801	1.2616	1.277714
162.041019	3.641371	309.6389	6.958178	17.97155	0.901629	1.988051	3.316806
177.505143	3.971032	272.6227	6.098942	15.76999	1.724429	1.742555	2.12791
199.037743	3.246945	303.5194	4.951377	13.85451	1.641331	1.414679	1.704432
231.490807	4.119053	306.95	5.461744	14.95428	1.40195	1.560498	1.34269
114.767488	7.404354	170.955	11.02935	21.88432	1.489577	3.151244	3.625001
150.938907	5.409997	402.15	14.41398	33.12721	2.664323	4.11828	9.003982
131.952524	6.283454	301.95	14.37857	30.78014	2.288323	4.108163	8.095118
129.932359	6.307396	291.571	14.15393	30.15393	2.244021	4.043981	7.846536
119.194861	6.374057	322.027	17.2207	35.81056	2.701685	4.920199	10.84664
194.013169	4.000272	633.699	13.06596	34.48075	3.266268	3.733131	9.065687
151.468554	4.91781	301.286	9.782013	23.04443	1.989099	2.794861	4.864203
134.343229	6.10651	301.92	13.72364	29.72178	2.247378	3.921039	7.617126
133.433888	6.149027	419.202	19.31806	41.69448	3.141646	5.519447	13.16904
137.766273	5.989838	492.596	21.41722	46.90239	3.575592	6.119205	15.42738

147.699329	5.023787	392.108	13.33701	31.05598	2.654772	3.810573	8.31322
178.592128	4.31382	482.5	11.65459	29.5629	2.701687	3.329883	7.34077
146.162813	5.092781	538.119	18.74979	43.39772	3.681641	5.357083	13.65701
179.277911	4.780744	667.153	17.79075	44.02527	3.721334	5.08307	13.01
151.507251	5.242465	720.447	24.92896	57.80011	4.755198	7.122561	19.6865
162.682983	5.180987	573.253	18.25646	43.21645	3.523743	5.216133	13.07548
257.584721	3.885139	943.651	14.23305	40.61405	3.663459	4.066585	10.34791
172.203913	4.906094	406.811	11.59006	28.2106	2.36238	3.311445	6.683963
292.237432	3.727518	649.565	8.285268	24.6539	2.22273	2.367219	4.55775
213.685751	4.28228	709.298	14.21439	37.77925	3.319351	4.061254	9.932109
189.383273	4.153142	685.564	15.0343	39.06829	3.619982	4.295514	10.88116
112.790795	7.138658	254.692	16.11975	32.13827	2.258092	4.605642	8.981089
116.49212	6.694949	395.9	22.75287	46.4701	3.398513	6.500821	16.05792
145.527747	5.684678	554.75	21.66992	48.74357	3.811988	6.191406	15.98524
133.742531	6.191784	317.375	14.69329	31.67615	2.37303	4.198082	8.501503
130.10554	6.40914	386.077	19.01857	40.36936	2.967414	5.433878	12.60943
144.196183	5.744868	773.462	30.81522	68.97373	5.363956	8.804348	25.07035
157.636548	4.663803	367.909	10.88488	26.24538	2.333907	3.109966	6.221078
140.364054	5.357407	417.417	15.93195	36.04493	2.973817	4.551985	10.57454
171.487386	4.89964	591.667	16.90477	41.11746	3.450207	4.829935	12.00513
218.795812	4.191491	504.182	9.658659	25.96178	2.304349	2.759617	5.467168
221.718148	4.191269	453.917	8.580662	23.14114	2.04727	2.451618	4.389392
173.522638	4.92962	422.75	12.00994	29.25342	2.436282	3.431412	7.080323
158.887899	5.261189	327.538	10.84563	25.42474	2.061441	3.098751	5.58444
220.870941	3.827919	594.75	10.30763	28.40878	2.692749	2.945036	6.479706
197.481586	4.030236	558.923	11.40659	30.17901	2.830254	3.259026	7.376355
184.074034	4.67193	741.333	18.81556	47.14017	4.027363	5.375874	14.14363
170.372392	4.996258	678.1	19.88563	48.05382	3.980105	5.681609	14.88937
159.003353	5.300112	541.667	18.05557	42.2563	3.406639	5.158733	12.75545
167.536458	5.031125	445.786	13.38697	32.15831	2.66083	3.824848	8.355842
226.836815	3.737015	1005.421	16.56377	46.23346	4.432354	4.732506	12.82676
153.514327	5.117144	445.2	14.84	34.73075	2.900055	4.24	9.722856
157.314865	4.870429	555.727	17.20517	41.0166	3.532578	4.915763	12.33474
167.052601	4.679345	732.8	20.52661	50.17464	4.386642	5.864746	15.84727
245.816466	3.990527	646.249	10.49106	29.391	2.62899	2.997444	6.500528
195.320262	3.196731	642.602	10.51722	29.40432	3.289991	3.004919	7.320487
293.69089	3.459257	906.327	10.67523	32.40441	3.085989	3.050066	7.215973
229.750171	4.023646	942.224	16.5013	45.36042	4.101081	4.714656	12.47765
194.742507	3.44068	513.948	9.080353	24.90616	2.639116	2.594387	5.639673
177.308047	3.567566	538.399	10.83298	28.76318	3.036518	3.095137	7.265412
181.592387	3.526066	851.51	16.53417	44.29292	4.689128	4.72405	13.00811
220.889429	4.183512	677.243	12.82657	34.57555	3.065982	3.664735	8.64306
317.918124	3.357108	1218.096	12.86268	40.12537	3.831477	3.675052	9.505574
373.202248	3.14408	897.807	7.563665	24.96577	2.405685	2.161047	4.419585
179.299342	4.002217	405.164	9.043839	23.39766	2.259707	2.583954	5.041622
230.839599	2.674851	764.959	8.863951	27.01656	3.313812	2.532558	6.189101
206.50782	3.086813	638.756	9.547922	27.30646	3.093132	2.727978	6.461109

184.898395	3.742882	650.395	13.16589	34.90455	3.517581	3.761683	9.423008
194.389483	4.531223	620.846	14.47193	37.03744	3.193825	4.134838	9.940711
242.610713	3.832713	447.875	7.075434	19.9574	1.846064	2.021553	3.242722
203.471042	4.357915	661.357	14.16485	37.02696	3.250374	4.047101	9.806938
198.063103	4.542732	654.273	15.00626	38.56063	3.303356	4.287503	10.46353
214.610367	4.232946	471.833	9.306371	24.83318	2.198556	2.658963	5.073425
209.538199	4.27629	960.5	19.60204	51.86213	4.58389	5.600583	15.32575
185.628677	4.910812	593	15.68783	38.89877	3.19455	4.482237	10.77702
151.749277	4.84822	498.056	15.91233	37.63743	3.282098	4.546381	11.06411
207.703998	2.841368	650.75	8.902189	26.03012	3.133064	2.543483	6.060821
184.986116	3.477183	759.333	14.27318	38.54771	4.104811	4.07805	10.79599
168.265436	4.124153	799.818	19.60338	49.54457	4.753311	5.600966	15.47923
276.14743	3.540352	787	10.08974	29.98498	2.849927	2.882784	6.549392
205.372949	4.378954	818.333	17.44846	45.66153	3.984619	4.985276	13.06951
263.736767	3.693792	881.143	12.34094	35.87342	3.340994	3.525982	8.647146
190.445592	4.714	695.071	17.20473	43.37537	3.649709	4.915636	12.49073
164.791244	5.567272	1325.167	44.76916	104.4244	8.041489	12.79119	39.20188
201.473544	3.011563	1313	19.62631	56.13001	6.516985	5.607517	16.61475
173.480707	4.294077	704.067	17.4274	43.93675	4.058474	4.979257	13.13332
174.107944	3.627249	546.188	11.37892	29.95099	3.137065	3.251119	7.751668
170.331474	4.279685	750.6	18.8593	47.36922	4.406702	5.38837	14.57961
165.853508	4.364566	706.286	18.58647	46.14694	4.258493	5.310421	14.22191
271.41857	3.618914	630.4	8.405333	24.73548	2.322612	2.401524	4.786419
203.266826	4.418844	1092.286	23.74535	61.83979	5.373656	6.784385	19.3265
228.007621	4.108245	1622.357	29.23166	79.786	7.115363	8.351902	25.12341
236.389308	3.946399	871.909	14.55608	40.49497	3.688445	4.158879	10.60968
298.6018	3.436154	1321.222	15.20394	46.42062	4.424695	4.343982	11.76778
179.119387	4.098842	568.889	13.01805	33.47083	3.176033	3.719444	8.919213
193.840307	3.550189	858.462	15.72275	42.73922	4.428707	4.492214	12.17256
222.583067	4.264043	994.057	19.04324	51.18685	4.466005	5.440925	14.77919
237.698581	3.915957	915.589	15.08384	42.10261	3.851891	4.309668	11.16788
213.345108	2.926545	782.045	10.72764	31.34629	3.665634	3.06504	7.801096
197.248749	3.673161	1064.14	19.81639	53.64362	5.394914	5.661825	16.14323
226.218072	4.173765	584.304	10.78052	29.25091	2.582924	3.080148	6.606751
208.227704	4.374532	462.265	9.71145	25.50855	2.219998	2.7747	5.336918
340.51532	3.286827	1404.456	13.55653	43.25022	4.124502	3.873293	10.2697
191.647961	3.714108	716.033	13.87661	37.19168	3.736189	3.964745	10.1625
186.423182	3.958029	705.563	14.98011	39.24372	3.784739	4.28003	11.02208
214.842899	2.971548	546.996	7.565643	22.06127	2.546028	2.161612	4.594095
240.736608	3.946502	754.988	12.37685	34.58937	3.136158	3.536244	8.430351
268.55718	3.699135	1100.934	15.16438	44.26483	4.09944	4.33268	11.46525
313.051852	3.373404	1042.475	11.23357	34.8662	3.330039	3.209591	7.860163
204.639225	3.279475	618.815	9.916907	27.87227	3.023932	2.833402	6.637432
242.478256	2.604493	633.389	6.803319	21.13286	2.612148	1.943805	4.198826
179.616222	5.031267	736.667	20.63493	50.43942	4.101339	5.895694	15.60366
291.956722	3.463306	1201.889	14.25728	43.20099	4.116668	4.07351	10.79398
178.552764	5.015527	606.571	17.03851	41.61924	3.397153	4.868146	12.02298

176.325218	5.140677	701.824	20.46134	49.51738	3.980281	5.846097	15.32066
200.572107	4.51739	1199	27.0045	69.70789	5.9779	7.715573	22.48711
195.221978	4.670382	416.824	9.971866	25.3554	2.135128	2.849105	5.301484
319.85469	3.314556	938.692	9.727378	30.48791	2.934745	2.779251	6.412822
219.379063	4.139228	1632.6	30.80377	83.11375	7.441913	8.801078	26.66455
267.385403	3.698277	1530.714	21.1717	61.73626	5.724748	6.049057	17.47342
330.764425	3.252354	1048.538	10.31011	32.74111	3.170045	2.945745	7.057754
220.286218	2.820566	799.846	10.24131	30.44515	3.63094	2.926087	7.42074
175.841764	4.176764	845.867	20.09185	51.17885	4.810387	5.740529	15.91509
223.783076	2.797288	1075.714	13.44643	40.21419	4.80695	3.841836	10.64914
241.960879	2.557726	1273.5	13.46195	41.98369	5.263248	3.84627	10.90422
262.725026	3.742522	1818.455	25.90392	74.98074	6.921514	7.401119	22.1614
306.156768	3.439964	2308.182	25.93463	79.65766	7.539216	7.409894	22.49467
262.942193	3.79426	1186.455	17.12056	49.39709	4.512228	4.891589	13.3263
176.62647	3.839706	585.267	12.7232	33.1349	3.313586	3.635199	8.88349
244.463998	2.541206	1241.412	12.90449	40.41426	5.078097	3.686997	10.36328
202.524052	3.38669	928.857	15.53273	43.19395	4.586403	4.437922	12.14604
222.652481	3.079564	735.733	10.17611	29.67335	3.304401	2.907461	7.096549
270.732384	2.352149	922.833	8.017663	26.26132	3.408654	2.290761	5.665514
243.991273	3.954478	1310.059	21.23272	59.50822	5.369286	6.066492	17.27825
339.642295	3.281568	1257.667	12.15137	38.75791	3.702916	3.471821	8.869804
332.850284	3.263238	782.733	7.673853	24.38729	2.351607	2.192529	4.410615
247.475153	3.848758	940.733	14.63037	41.42935	3.801323	4.180107	10.78162
242.044659	3.954978	1185.864	19.37686	54.19651	4.89936	5.536246	15.42188
242.374337	3.97335	1264.167	20.72405	57.91714	5.215763	5.921157	16.7507
192.667655	3.726647	1282.917	24.81464	66.53967	6.658705	7.089898	21.088
251.530029	2.341993	847.65	7.892458	25.40758	3.369975	2.254988	5.550465
189.633834	3.632832	1246.6	23.88123	64.19102	6.573721	6.823207	20.24839
243.615445	2.668296	1540.455	16.87245	52.155	6.323306	4.820701	14.20416
341.991263	3.250868	1411.013	13.41267	42.95554	4.125874	3.832192	10.1618
264.655568	3.748662	814.528	11.53722	33.44279	3.077691	3.29635	7.788561
189.675544	3.959823	491.309	10.25697	26.9838	2.59026	2.930564	6.297149
280.092904	3.656565	1123.322	14.66478	43.38431	4.010534	4.189937	11.00821
271.727983	3.662102	736.211	9.921981	29.12054	2.709368	2.834852	6.259879
215.370824	2.902572	680.104	9.165822	26.90125	3.157828	2.618806	6.26325
343.655198	3.254311	798.706	7.563504	24.24594	2.324149	2.161001	4.309193
193.53231	3.721775	565.884	10.88238	29.22301	2.923977	3.109253	7.160609
233.898653	4.089137	883.611	15.44774	42.48289	3.777752	4.413641	11.35861
380.344896	3.104856	688.176	5.617763	18.68949	1.809347	1.605075	2.512907
279.473384	2.136647	1445.067	11.04791	37.36218	5.170678	3.156547	8.911266
229.28143	2.834134	904.778	11.18391	33.54136	3.946146	3.195402	8.349772
282.814355	3.658659	923.3	11.94437	35.41671	3.264686	3.412678	8.285713
262.622647	3.762502	1225.538	17.55785	50.7499	4.666536	5.016529	13.79535
253.187634	3.859568	1202.462	18.33021	52.16671	4.749292	5.237204	14.47065
358.923586	3.181947	960	8.510638	27.73572	2.674664	2.431611	5.328692
195.145513	3.717057	744	14.17143	38.14639	3.81254	4.04898	10.45437
188.382969	3.737757	713.077	14.14835	37.69758	3.785252	4.042387	10.4106

186.784826	3.991129	748.667	15.99716	41.84122	4.008179	4.570617	12.00603
240.762966	2.648657	610.187	6.712728	20.72717	2.534389	1.917922	4.064071
217.539297	2.923915	769.6	10.34409	30.37983	3.537752	2.955453	7.420171
228.183522	2.786124	710.857	8.679573	26.11075	3.115286	2.479878	5.893449
298.877318	3.451239	1216.923	14.05223	42.86716	4.071647	4.014922	10.60099
232.543299	2.685257	1068.944	12.34346	37.65447	4.596753	3.526704	9.658207
184.098282	3.749456	578.3	11.778	31.17756	3.141257	3.365144	8.028548
150.597417	5.53667	475.3293	17.47534	39.90873	4.330403	4.992955	11.93867
230.15741	3.718213	939.8718	15.18371	42.58931	4.187313	4.338204	11.4655
176.090452	4.347912	546.2994	13.48887	34.02824	3.591221	3.853964	9.140962
142.363537	5.834571	380.8582	15.60894	34.69133	3.410468	4.459698	9.774371
162.41131	5.205491	614.6426	19.70008	46.55933	4.040724	5.628595	14.49459
198.694809	4.48521	855.5104	19.31175	49.82215	3.046763	5.517642	14.82654
164.69611	4.692197	592.0318	16.867	41.05487	2.571606	4.819144	12.17481
150.966681	5.117515	399.3178	13.5362	31.54657	4.621206	3.867485	8.418682
237.204425	3.723774	931.3084	14.62023	41.3037	2.666046	4.177207	10.89645
150.460866	6.06697	817.7819	32.97508	73.5866	6.605558	9.42145	26.90811
178.170676	5.090591	639.0337	18.25811	44.40918	3.312858	5.216602	13.16751
173.05745	5.181361	328.7324	9.842287	23.66098	1.332723	2.812082	4.660927
163.745761	3.781657	551.6817	12.74092	32.68306	2.796599	3.640262	8.95926
164.679815	4.499449	744.1392	20.33167	50.00845	3.650875	5.809049	15.83222
190.174738	3.521754	590.6734	10.9384	29.65187	2.072778	3.125256	7.416642
203.867302	4.403181	987.131	21.32032	55.61463	4.031486	6.091521	16.91714
175.806856	5.201386	395.6458	11.7055	28.22403	2.540035	3.344428	6.504111
207.293358	4.410497	630.0355	13.40501	35.09875	2.66381	3.830003	8.994514
188.507889	4.7603	331.0925	8.360922	20.97384	1.884876	2.388835	3.600621
161.280631	5.780668	339.101	12.15416	27.93354	2.66635	3.472616	6.37349
224.457172	4.11848	649.5282	11.91795	32.38177	3.512864	3.405128	7.799468
188.35389	3.418401	548.6947	9.958162	27.13108	2.279088	2.845189	6.539761
153.96966	4.950793	559.6008	17.99359	42.49208	3.656642	5.141027	13.0428
172.911072	4.039978	698.9827	16.33137	41.77184	3.001115	4.666106	12.29139
191.738792	4.665177	546.1856	13.28919	33.64797	2.407795	3.796911	8.62401
257.54576	3.737965	724.1072	10.50954	30.27879	2.998966	3.002725	6.771574
219.263581	4.184419	759.9529	14.50292	39.02009	2.600374	4.143691	10.3185
176.551726	4.193628	733.9666	17.43389	44.40836	2.898553	4.98111	13.24026
158.022134	4.90752	624.3197	19.38881	46.18651	4.508651	5.53966	14.48129
182.460512	3.898729	668.9189	14.29314	37.38428	2.749626	4.083754	10.39441
189.634528	3.703799	1032.723	20.17037	53.95496	5.0443	5.762963	16.46657
173.105739	4.191422	557.6275	13.50188	34.22801	4.71292	3.857679	9.310454
191.17221	4.743727	867.5429	21.52712	54.23906	5.14194	6.150605	16.78339
187.198616	4.824707	692.6496	17.85179	44.55432	3.149769	5.100513	13.02709
275.859337	3.591918	1443.173	18.79132	55.62852	4.492302	5.368947	15.1994
224.527932	4.204643	947.1001	17.73596	47.94463	3.634373	5.067416	13.53131
276.388359	3.62239	1303.019	17.07758	50.47279	4.611614	4.879307	13.45519
201.252949	4.452499	592.8628	13.11643	34.00951	2.306148	3.747552	8.663935
181.903868	4.929644	641.2655	17.37847	42.83198	2.39517	4.965277	12.44882
169.038607	5.366305	535.936	17.01384	40.30696	2.614433	4.861098	11.64754

197.913726	4.549741	526.6294	12.10642	31.09125	3.187644	3.458978	7.556682	
284.538029	3.465749	699.4744	8.519786	25.64569	1.553625	2.434224	5.054036	
246.067694	3.868989	882.5058	13.87588	39.18544	3.035927	3.964536	10.00689	
214.868063	4.213099	968.1138	18.98262	50.72812	4.384744	5.423607	14.76952	
162.318064	4.521395	576.2815	16.05241	39.29288	2.575487	4.586403	11.53101	
184.748213	4.336813	573.1002	13.45306	34.36951	3.931839	3.84373	9.116244	
158.912051	4.772134	423.2405	12.70992	30.53191	1.850171	3.631407	7.937791	
280.843451	3.559486	700.0326	8.872403	26.44299	1.865212	2.534972	5.312917	
234.586657	4.016895	810.4015	13.87674	38.36106	2.199982	3.964782	9.859843	
282.845446	3.531154	1399.985	17.47797	52.28763	2.761726	4.993704	13.94681	
212.398894	4.290887	884.2769	17.86418	47.38429	3.11147	5.104051	13.57329	
235.349613	3.988976	944.8191	16.01388	44.38228	5.499606	4.575395	12.02491	
225.261185	4.095658	921.9342	16.76244	45.64865	2.795674	4.789269	12.66678	
265.2204	3.678508	825.7419	11.45273	33.37282	4.041836	3.272209	7.774223	
225.877735	4.077215	678.3093	12.24385	33.40377	2.877001	3.498243	8.166635	
302.683714	3.416295	810.8312	9.151594	28.07729	2.044825	2.614741	5.735299	
283.013866	3.528851	827.9819	10.32396	30.89513	3.692257	2.949704	6.795113	
280.143566	3.586985	859.8625	11.00976	32.7296	2.23583	3.145647	7.422778	
191.248078	3.735314	674.0036	13.16413	35.21354	3.283411	3.761181	9.428819	
178.578831	4.313498	592.7336	14.31724	36.31694	4.521619	4.090639	10.00374	
159.570795	4.879841	434.4108	13.28473	31.76799	2.509845	3.795638	8.404893	
230.065095	2.665876	727.6983	8.432194	25.7006	1.768524	2.409198	5.766318	
223.690843	2.704847	983.943	11.89774	35.87904	3.382276	3.399354	9.192892	
181.053218	3.835873	939.9743	19.91471	52.19868	6.627883	5.689917	16.07884	
232.14411	2.724696	610.0788	7.160549	21.75486	1.664408	2.045871	4.435853	
183.09226	3.822385	849.1917	17.72843	46.63952	4.554432	5.065265	13.90604	
184.446948	3.771921	598.4212	12.23765	32.36125	1.761065	3.496472	8.465731	
245.207772	3.849416	797.1471	12.51408	35.35362	4.058769	3.575452	8.664668	
216.358164	4.309924	746.7406	14.87531	39.59515	1.905378	4.250089	10.56539	
243.884175	3.927281	907.076	14.6067	41.00388	5.224039	4.173343	10.67942	
205.38658	4.484423	612.109	13.36483	34.768	2.399568	3.818522	8.880402	
210.282638	4.353678	778.3886	16.11571	42.48505	1.858977	4.604487	11.76203	
210.238278	3.137885	598.0327	8.925861	25.53694	1.921985	2.550246	5.787976	
162.041019	3.641371	548.7513	12.33149	31.84972	1.597894	3.523283	8.690119	
177.505143	3.971032	945.3408	21.14856	54.68368	5.979593	6.042447	17.17753	
199.037743	3.246945	771.8098	12.5907	35.23019	4.173689	3.597342	9.343753	
231.490807	4.119053	1167.185	20.76842	56.86401	5.330951	5.933833	16.64936	
134.625146	6.631781	686.273	33.80655	71.75875	5.097658	9.659015	27.17477	4.3
148.985616	5.935682	830.556	33.08988	74.0651	5.57474	9.454252	27.1542	4.3
175.604041	5.149679	1120.667	32.86413	79.41651	6.381784	9.389753	27.71446	4.3
138.288369	6.402239	674.625	31.23264	67.33209	4.878393	8.923611	24.8304	4.3
163.890945	5.426852	889.286	29.44656	69.02974	5.426084	8.413302	24.0197	4.3
176.370509	5.039157	951	27.17143	66.08905	5.392058	7.763265	22.13227	4.3
178.795511	5.079418	1028.5	29.21875	71.17005	5.752382	8.348214	24.13933	4.3
153.514327	5.117144	1101.167	36.70557	85.90377	7.173057	10.4873	31.58842	4.3
167.052601	4.679345	1245.286	34.88196	85.26444	7.454454	9.966275	30.20262	4.3
190.445592	4.714	1302.25	32.23391	81.2659	6.837911	9.209689	27.51991	4.3

298.6018	3.436154	2457.286	28.27717	86.33579	8.229307	8.079191	24.84101	4.3
214.610367	4.232946	1574.667	31.05852	82.87676	7.33733	8.873863	26.82557	4.3
236.389308	3.946399	2123.286	35.44718	98.61396	8.982158	10.12777	31.50078	4.3
203.471042	4.357915	1360.143	29.13136	76.14943	6.684701	8.323245	24.77344	4.3
194.389483	4.531223	1357.667	31.64725	80.99352	6.984262	9.042071	27.11603	4.3
209.538199	4.27629	1379.5	28.15306	74.486	6.583525	8.043732	23.87677	4.3
151.749277	4.84822	872.222	27.86652	65.91265	5.747784	7.961862	23.0183	4.3
173.480707	4.294077	1087.375	26.91522	67.85679	6.267988	7.690064	22.62115	4.3
179.119387	4.098842	1330.5	30.44622	78.28054	7.428007	8.698921	26.34738	4.3
184.986116	3.477183	1446.917	27.19769	73.45306	7.82176	7.770768	23.72051	4.3
201.473544	3.011563	1760	26.30792	75.23901	8.735638	7.516549	23.29636	4.3
339.642295	3.281568	2786.429	26.92202	85.87025	8.204011	7.692006	23.64045	4.3
178.552764	5.015527	1158.857	32.55216	79.51377	6.490278	9.300618	27.53664	4.3
291.956722	3.463306	2248.8	26.67616	80.83141	7.702511	7.621759	23.21285	4.3
332.850284	3.263238	2819.111	27.63834	87.83388	8.469607	7.896669	24.37511	4.3
319.85469	3.314556	3101.8	32.14301	100.7438	9.697529	9.183716	28.82845	4.3
223.783076	2.797288	1949.75	24.37188	72.88891	8.712679	6.963393	21.57459	4.3
222.652481	3.079564	1924.556	26.61903	77.62057	8.643766	7.605438	23.53947	4.3
176.62647	3.839706	1368.167	29.74276	77.4588	7.746104	8.497932	25.90306	4.3
243.615445	2.668296	2158.333	23.64001	73.07442	8.85959	6.754289	20.97171	4.3
271.727983	3.662102	1979.545	26.6785	78.30012	7.285024	7.62243	23.0164	4.5
184.098282	3.749456	1280.25	26.07434	69.02138	6.954166	7.449811	22.32488	4.3
228.183522	2.786124	2125.714	25.95499	78.08038	9.315809	7.415713	23.16887	4.3
232.543299	2.685257	2255.625	26.04648	79.45633	9.699806	7.441851	23.36122	4.3
142.840843	6.130508	1110	47.63948	104.6661	7.770887	13.61128	41.50898	4.6
131.318409	6.734277	944.92	48.45744	101.8284	7.19564	13.84498	41.72316	4.7
135.516034	6.44302	1075.33	51.12585	109.4878	7.935076	14.60739	44.68283	4.4
154.068049	5.722119	1208.42	44.88097	102.2354	7.843417	12.82313	39.15885	4.7
153.558465	5.666364	1186.83	43.79446	99.9221	7.728848	12.5127	38.1281	4.3
171.691839	5.210678	1446.5	43.89985	105.1785	8.424978	12.54281	38.68917	4.3
143.235541	6.101621	1135	48.34931	106.4245	7.924011	13.81409	42.24769	4.3
167.739245	5.299818	1219.92	38.54408	91.42207	7.272717	11.01259	33.24426	4.3
190.389934	4.805764	1356	34.22773	85.87139	7.122225	9.779352	29.42197	4.3
156.892622	5.722458	1174.17	42.82635	97.99773	7.483908	12.2361	37.10389	4.7
174.368471	5.166473	1375.33	40.75052	98.22037	7.887492	11.64301	35.58405	4.5
136.355385	5.669663	913.83	37.99709	84.14515	6.701826	10.85631	32.32743	4.5
137.841503	5.580628	1130.33	45.76235	102.0193	8.200215	13.07496	40.18172	4.5
131.032932	6.08974	830.67	38.60529	83.14617	6.339399	11.03008	32.51555	4.5
191.621382	4.158179	1766.42	38.33127	99.87077	9.218282	10.95179	34.17309	4.6
181.195391	4.343442	1561.67	37.43486	95.1381	8.618707	10.69567	33.09142	4.5
137.42327	5.655279	1050.5	43.23045	95.98234	7.644266	12.35156	37.57517	4.5
141.162708	5.436235	1072	41.28317	93.19196	7.594074	11.79519	35.84693	4.3
176.811435	5.110157	1548.42	44.75202	108.538	8.757465	12.78629	39.64187	4.7
242.301959	3.878383	2095.75	33.54542	94.31044	8.649332	9.584405	29.66704	4.7
219.887644	4.16983	1921.17	36.43203	98.17568	8.737053	10.40915	32.2622	4.6
193.056639	4.739563	1635.83	40.15982	101.4561	8.473316	11.47423	35.42026	4.6
183.665118	4.795434	1614.08	42.14308	104.8397	8.788168	12.04088	37.34765	4.6

194.730435	4.555098	1480.08	34.62175	88.5285	7.600661	9.89193	30.06666	4.7
156.648556	5.922441	1182.75	44.71645	101.4083	7.550341	12.77613	38.79401	4.7
162.231137	5.636353	1235.17	42.91318	99.39732	7.613643	12.26091	37.27683	4.7
185.912936	3.556441	1527.25	29.21569	78.55785	8.214867	8.347339	25.65925	4.3
203.73784	3.189634	1904.42	29.81479	84.28777	9.347404	8.518513	26.62516	4.6
165.895044	4.886452	1239.08	36.4972	88.0987	7.46906	10.42777	31.61075	4.7
225.114198	3.794081	1689.5	28.47488	79.02891	7.50508	8.13568	24.6808	4.5
141.375579	5.882561	1006.92	41.89739	92.76603	7.122305	11.97068	36.01483	4.5
183.684359	3.909592	1501.42	31.95667	83.66554	8.173913	9.130476	28.04707	4.5
174.833487	4.170945	1525.25	36.38738	92.58665	8.724015	10.3964	32.21644	4.5
267.453193	3.751097	2025	28.40112	82.52925	7.571418	8.114606	24.65003	4.7
344.174438	3.231685	2749.33	25.81531	82.93057	7.988188	7.375801	22.58362	4.4
241.683449	3.882465	2222.92	35.70956	100.3042	9.197651	10.20273	31.82709	4.5
202.199682	3.280705	1903.17	30.87907	86.52022	9.412329	8.822593	27.59837	4.4
187.620576	3.804379	1557.67	31.58485	83.70048	8.302234	9.024243	27.78047	4.4
198.747033	3.374313	1545.92	26.24652	72.71107	7.77833	7.499006	22.87221	4.6
205.473961	3.308759	1544.83	24.87649	69.83321	7.518374	7.107568	21.56773	4.6
214.298455	3.126938	2094.33	30.55944	87.92661	9.772959	8.731268	27.4325	4.3
187.86276	4.892259	867.7626	22.59798	56.25388	4.61913	6.456567	17.70572	4.7
225.261185	4.095658	1253.245	22.78627	62.05317	5.56352	6.510364	18.69061	4.4
197.913726	4.549741	1605.44	36.90667	94.78228	8.111817	10.54476	32.35693	4.5
225.877735	4.077215	2383.937	43.03135	117.3985	10.5541	12.29467	38.95414	4.3
210.755752	4.354458	2029.386	41.92946	110.5938	9.629089	11.97985	37.57501	4.7
152.086352	5.208437	1404.52	48.1	111.8126	9.235017	13.74286	42.89156	4.7
158.822594	4.963206	1164.332	36.38538	86.53949	7.331022	10.39582	31.42217	4.5
181.98068	3.639614	1759.47	35.1894	93.57382	9.668444	10.05411	31.54979	4.3
185.957716	3.858044	1367.959	28.38089	74.7804	7.356291	8.108826	24.52285	4.3
197.102177	3.268693	1556.571	25.81378	71.93346	7.89728	7.375366	22.54509	4.6
200.804597	3.157305	1468.965	23.09693	65.22568	7.315395	6.599124	19.93963	4.3
158.912051	4.772134	1110.776	33.35664	80.12966	6.989879	9.530468	28.5845	4.5
284.376077	3.532622	2373.823	29.48848	88.32862	8.347478	8.425281	25.95586	4.5
293.473029	3.497891	2860.17	34.09023	103.174	9.745938	9.740065	30.59234	4.5
200.216224	4.529779	1738.166	39.32502	101.3969	8.681444	11.23572	34.79524	4.7
243.884175	3.927281	2382.346	38.36306	107.6927	9.76835	10.96087	34.43578	4.5
231.490807	4.119053	2663.125	47.38657	129.7446	11.50424	13.53902	43.26751	4.4
205.38658	4.484423	1925.481	42.04107	109.368	9.374911	12.01173	37.55665	4.6
199.037743	3.246945	1960	31.9739	89.46656	9.847379	9.1354	28.72695	4.5
187.610547	3.958028	1897.048	40.02211	105.0134	10.11163	11.43489	36.06408	4.6
167.208252	5.359239	871.4625	27.93149	66.0135	5.211839	7.980425	22.57225	4.5
129.987588	6.735108	620.9552	32.17384	67.43607	4.777034	9.192527	25.43874	4.5
153.006814	5.730592	870.0978	32.58793	74.07721	5.68666	9.310837	26.85734	4.5
123.549628	7.267625	479.6608	28.21534	57.29247	3.882333	8.061526	20.94771	4.5
174.590482	5.135014	1136.435	33.42454	80.71146	6.509143	9.54987	28.28953	4.5
123.366481	7.387214	433.6757	25.9686	52.49618	3.515344	7.419601	18.58139	4.5
176.371272	5.024823	914.2138	26.04598	63.39682	5.183462	7.441708	21.02116	4.5
156.676829	5.575688	848.463	30.19441	69.51894	5.41537	8.626975	24.61872	4.5
152.677495	5.739755	707.178	26.58564	60.37644	4.631842	7.595897	20.84588	4.5

135.622146	6.583599	502.2902	24.38302	51.94626	3.7036	6.966576	17.79942	4.5
126.69156	6.240964	711.693	35.05877	74.41674	5.617525	10.01679	28.8178	4.5
130.773612	5.864288	985.6311	44.1987	96.04743	7.536926	12.6282	38.33441	4.5
149.093824	5.213071	896.1659	31.33447	72.4626	6.010751	8.952706	26.1214	4.5
132.78227	5.823784	623.5636	27.34928	59.76262	4.696136	7.81408	21.5255	4.5
140.556792	5.512031	746.845	29.28804	65.81507	5.313475	8.368011	23.77601	4.5
145.997016	5.27065	683.3352	24.66914	56.59447	4.680474	7.048326	19.39849	4.5
128.856368	6.078131	596.0632	28.11619	60.33098	4.625795	8.033196	22.03806	4.5
137.528675	5.706584	733.9741	30.45536	67.47889	5.336881	8.701531	24.74877	4.5
128.070344	6.041054	656.9107	30.98636	66.48971	5.129296	8.853245	24.9453	4.5
154.27552	4.992735	636.3462	20.59373	48.55395	4.124739	5.883923	15.60099	4.5
145.288803	5.188886	829.4473	29.62312	68.14285	5.708956	8.463748	24.43423	4.5
139.746463	5.333834	620.3912	23.67905	53.57222	4.439405	6.765443	18.34522	4.5
144.574422	5.200519	887.832	31.9364	73.33262	6.141003	9.124686	26.73588	4.5
135.14999	5.775641	597.3297	25.52691	56.14386	4.419754	7.293403	19.75127	4.5
142.396225	5.414305	616.1877	23.42919	53.05743	4.327276	6.694054	18.01488	4.5
134.824879	5.811417	654.0972	28.19384	61.87658	4.851458	8.055384	22.38243	4.5
168.882124	4.69117	847.9723	23.55479	57.69721	5.02109	6.729939	18.86362	4.5
145.929642	5.268218	728.3079	26.29271	60.31915	4.990816	7.512202	21.02449	4.5
121.539859	6.264941	493.8722	25.45733	53.4273	4.063458	7.273522	19.19239	4.5
189.118303	4.799957	1323	33.57868	84.12744	6.995621	9.593909	28.77872	5
164.178789	5.472626	1025.5	34.18333	80.00087	6.246239	9.766667	28.71071	5
205.372949	4.378954	1165.667	24.85431	65.04215	5.675855	7.101231	20.47535	5
185.628677	4.910812	1050.7	27.7963	68.92232	5.660225	7.941799	22.88548	5
164.791244	5.567272	1178.083	39.8001	92.83401	7.148942	11.37146	34.23283	5
203.266826	4.418844	1551.111	33.7198	87.81617	7.630911	9.63423	29.30096	5
198.063103	4.542732	1385	31.76606	81.6272	6.992721	9.076016	27.22332	5
220.889429	4.183512	1830.143	34.6618	93.435	8.285335	9.903371	30.47829	5
170.331474	4.279685	1385.636	34.81497	87.44538	8.134938	9.947136	30.53529	5
193.840307	3.550189	1908.5	34.95421	95.01621	9.845733	9.986918	31.40402	5
168.265436	4.124153	1118.667	27.41831	69.29561	6.648228	7.833803	23.29416	5
204.639225	3.279475	1423.443	22.81159	64.11382	6.955866	6.517596	19.53211	4.9
242.374337	3.97335	1927.5	31.59836	88.30738	7.952575	9.028103	27.62501	5
179.616222	5.031267	1185.778	33.21507	81.18994	6.601731	9.49002	28.1838	5
222.583067	4.264043	1704.041	32.64446	87.74597	7.655753	9.32699	28.38042	5
313.051852	3.373404	2730.384	29.42224	91.31934	8.721827	8.406355	26.04884	5
195.221978	4.670382	1228.857	29.39849	74.75137	6.294665	8.399569	24.72811	5
243.991273	3.954478	2530.833	41.01836	114.9608	10.37264	11.71953	37.06389	5
176.325218	5.140677	1113.444	32.46192	78.55933	6.314718	9.274835	27.32125	5
219.379063	4.139228	1932.111	36.45492	98.36151	8.80718	10.41569	32.3157	5
306.156768	3.439964	2955	33.20225	101.98	9.651918	9.486356	29.76228	5
189.633834	3.632832	1859.818	35.6287	95.76737	9.807417	10.17963	31.99587	5
192.667655	3.726647	1698	32.84333	88.06833	8.813104	9.383808	29.11668	5
202.524052	3.38669	2082	34.81605	96.81772	10.28026	9.947444	31.42936	5
280.092904	3.656565	2460.122	32.11648	95.01345	8.783236	9.176136	28.45991	4.9
264.655568	3.748662	1511.923	21.41534	62.07635	5.712795	6.118669	17.66668	5
298.877318	3.451239	2541.444	29.34693	89.52455	8.503302	8.384837	25.89569	5

358.923586	3.181947	2948.125	26.13586	85.17538	8.213796	7.467389	22.95391	5
253.187634	3.859568	2242.5	34.18445	97.28695	8.857068	9.766986	30.32488	5
186.784826	3.991129	1352.625	28.90224	75.59499	7.241621	8.257784	24.91111	5
240.762966	2.648657	2222.667	24.45178	75.50079	9.231764	6.986223	21.80312	5
188.382969	3.737757	1587.636	31.50071	83.93209	8.427705	9.000204	27.76296	5
217.539297	2.923915	2308.333	31.02598	91.12106	10.61111	8.864566	28.10207	5
229.28143	2.834134	2205.222	27.25862	81.7506	9.61797	7.788176	24.42448	5
183.307101	4.97104	1700.92	46.12664	113.6671	9.279073	13.17904	41.1556	4.9
180.506763	5.003652	1083.17	30.0255	73.58544	6.000717	8.578715	25.02185	4.9
155.074092	5.696018	1288.25	47.31864	108.0872	8.307319	13.51961	41.62262	4.9
161.655752	5.485993	1275	43.26874	100.8111	7.88713	12.3625	37.78275	4.8
153.900316	5.789426	1142.42	42.97559	97.58269	7.423117	12.27874	37.18616	4.8
168.003617	5.324996	1086.42	34.43487	81.61091	6.466646	9.838533	29.10987	4.8
155.498682	4.923493	1255.42	39.74987	94.23213	8.073509	11.3571	34.82637	4.9
154.797533	4.940399	1131.92	36.12549	85.47011	7.312261	10.32157	31.18509	4.8
175.50344	4.394948	1570.17	39.32011	98.84341	8.946662	11.23432	34.92516	4.8
148.2506	5.180508	1037.75	36.26341	83.87348	6.999972	10.36097	31.0829	4.8
231.095685	3.748511	1605.75	26.04623	72.98408	6.948421	7.44178	22.29772	4.8
142.831037	6.15651	1228.17	52.93836	116.183	8.598761	15.12525	46.78185	5
177.151759	5.07962	1517.42	43.51025	105.7353	8.56565	12.4315	38.43063	5.1
168.648329	5.286781	1497.08	46.93041	111.5326	8.876933	13.40869	41.64363	5
154.38098	5.707245	1190	43.99261	100.3279	7.708203	12.56932	38.28536	5.1
158.210113	4.870551	1462.67	45.02878	107.499	9.245111	12.86537	40.15823	5
163.107795	4.769234	1443.5	42.2076	102.0698	8.849976	12.05931	37.43837	5
149.426896	5.157097	1128.5	38.94737	90.36161	7.552188	11.12782	33.79027	5.1
154.882145	5.028641	1134.25	36.8263	86.75525	7.32331	10.5218	31.79766	5
158.286299	4.850498	1308.58	40.0999	95.84237	8.267172	11.45711	35.2494	5.2
173.206348	5.268634	1297.42	39.46525	94.49988	7.490603	11.27578	34.19661	4.9
173.552921	5.165266	1181.83	35.17351	84.68384	6.809623	10.04957	30.00825	4.8
150.402986	5.469199	1137.92	41.37891	94.7572	7.565807	11.82255	35.90971	4.8
160.096518	4.833102	1390.17	41.9674	100.6818	8.683324	11.99068	37.13429	4.8
161.831819	4.205281	1446.33	37.58361	93.60859	8.937241	10.73817	33.37833	4.9
161.21654	4.528554	1302.5	36.58708	89.36969	8.079196	10.45345	32.05852	4.8
204.958319	3.354473	1974.67	32.31866	90.35738	9.634495	9.233902	28.96418	4.9
149.333039	5.295498	1042.83	36.97979	85.21709	6.98325	10.56565	31.68429	4.9
188.851085	3.671788	1673.33	32.53417	87.12649	8.860579	9.295477	28.86238	4.9
194.591056	4.575921	1859.42	43.72534	111.6591	9.555527	12.49295	39.14942	5
280.232835	3.548733	2583.58	32.71721	97.52988	9.219405	9.347775	29.16848	5.1
185.792642	4.882855	1494.5	39.27727	97.5506	8.043914	11.22208	34.39441	5.1
195.031801	4.663936	1552	37.11409	94.37937	7.957677	10.60403	32.45016	5.1
180.118425	5.014852	1636.67	45.56812	111.5542	9.086633	13.01946	40.55326	5.2
160.441495	5.736815	1248.08	44.62688	102.6261	7.779035	12.75054	38.89007	5.1
207.953064	4.305446	1434.5	29.69979	78.29611	6.898191	8.485655	25.39435	5
201.699296	3.455708	2029.5	34.77136	96.10888	10.06201	9.934674	31.31565	5
162.17521	4.433076	1120.25	30.62215	75.31049	6.907653	8.749185	26.18907	5
179.79542	4.152319	1574.17	36.35497	93.25792	8.75534	10.38713	32.20265	5.1
186.159241	3.763302	1602.83	32.40201	85.93118	8.609994	9.257716	28.6387	5.1

195.381188	3.267244	2129.67	35.61321	99.03448	10.90008	10.1752	32.34597	5.1
175.581306	5.210128	1517.75	45.03709	108.512	8.644143	12.86774	39.82696	4.9
208.64483	4.397151	2063.17	43.48093	114.119	9.888431	12.42312	39.08378	4.9
211.62437	3.055948	2042.83	29.49935	85.09762	9.653094	8.428386	26.4434	4.8
180.877921	3.940695	1629.92	35.51024	92.42869	9.011161	10.14578	31.56954	4.9
228.518147	2.857084	2349.75	29.37812	87.8564	10.28255	8.393748	26.52103	4.8
213.500147	2.936728	2464.58	33.90069	98.99018	11.54369	9.685911	30.96396	5
265.2204	3.678508	2867.291	39.76825	115.8832	10.81097	11.36236	36.08974	4.9
261.559189	3.752643	1890.875	27.12877	78.38595	7.229243	7.751076	23.37612	4.8
246.067694	3.868989	1744.941	27.43618	77.4797	7.091305	7.838908	23.56719	4.9
123.840622	2.433018	1478.239	29.04202	77.57224	11.93662	8.297721	26.60901	4.8
183.09226	3.822385	1788.883	37.3462	98.24949	9.770391	10.67034	33.52382	4.8
184.748213	4.336813	1312.869	30.81852	78.73434	7.106261	8.805292	26.48171	4.9
159.570795	4.879841	1496.917	45.77728	109.4679	9.380896	13.07922	40.89744	4.9
201.619076	3.262445	2381.451	38.53481	108.0439	11.81164	11.00994	35.27236	4.8
214.868063	4.213099	1648.491	32.32335	86.37915	7.672108	9.235244	28.11025	5.2
220.352023	4.213232	1558.769	29.80438	80.15036	7.073994	8.515537	25.59115	5
212.398894	4.290887	1508.993	30.48471	80.85992	7.104524	8.709916	26.19382	5
195.597307	3.725663	1300.009	24.76208	66.6541	6.646354	7.074879	21.03641	5.1
210.56357	4.288464	1834.721	37.36703	98.91426	8.713383	10.67629	33.07856	4.8
245.207772	3.849416	1628.806	25.56995	72.23785	6.642555	7.305701	21.72054	4.9
288.0794	3.592012	2729.226	34.03025	101.8377	9.473867	9.722928	30.43824	5.2
159.175701	5.585112	1418.365	49.76721	114.9885	8.91069	14.2192	44.18209	5
169.785573	5.240295	1276.516	39.39864	93.99775	7.5184	11.25675	34.15834	5
156.685754	5.824749	1485.013	55.20495	125.7234	9.477653	15.77284	49.3802	5
174.714028	5.199822	1158.552	34.48072	83.01588	6.631135	9.851635	29.2809	5
182.913166	4.970466	1345.547	36.56377	90.05606	7.356205	10.44679	31.5933	5
159.867536	5.49373	1294.599	44.48793	103.3274	8.097946	12.71084	38.9942	5
151.977381	5.822888	1171.345	44.87913	101.4389	7.707366	12.82261	39.05624	5
158.523091	5.601523	1278.102	45.1626	104.1659	8.062558	12.9036	39.56108	5
142.442195	6.13975	815.3822	35.14578	77.13389	5.724302	10.04165	29.00603	5
160.881859	5.490848	1403.452	47.89939	111.4415	8.723495	13.68554	42.40854	5
176.51214	5.101507	1487.424	42.98914	104.2624	8.426753	12.28261	37.88763	5
155.077659	5.722423	1013.306	37.39137	85.31269	6.534184	10.68325	31.66894	5
165.016072	5.375116	944.5416	30.76683	72.42147	5.723937	8.790522	25.39171	5
162.784668	5.462573	1300.601	43.64432	101.9722	7.989701	12.46981	38.18175	5
150.129592	5.841618	897.3708	34.91715	78.61802	5.977308	9.976329	29.07553	5
186.960768	4.831028	1347.347	34.81517	86.83528	7.206576	9.947191	29.98414	5
144.398498	6.11858	1114.616	47.22951	104.0978	7.71903	13.49414	41.11093	5
152.685774	5.761727	1128.311	42.57778	96.60387	7.38976	12.16508	36.81605	5
217.531024	4.448487	1765.376	36.10176	95.4675	8.115513	10.31479	31.65327	5
175.859531	5.112196	1396.712	40.60209	98.33045	7.942202	11.6006	35.4899	5
177.10744	5.148472	1362.677	39.61271	95.93436	7.694071	11.31792	34.46424	5
151.417489	5.779293	1137.797	43.42737	98.25141	7.514304	12.40782	37.64807	5
161.113155	5.594207	1406.597	48.84016	113.1424	8.73049	13.95433	43.24596	5
197.892365	4.700531	1273.821	30.25704	77.07206	6.43694	8.644868	25.55651	5
166.266271	5.363428	1235.03	39.83968	94.00615	7.428025	11.38277	34.47625	5

151.179766	5.107425	1089.125	36.79475	85.824	7.204169	10.51278	31.68732	5
145.301087	5.264532	990.8101	35.89892	82.28272	6.819014	10.25683	30.63439	5
145.098966	5.257209	1009.866	36.58934	83.8652	6.959841	10.4541	31.33213	5
146.076673	5.350794	1117.985	40.95183	93.60822	7.653412	11.70052	35.60104	5
135.183677	5.77708	1153.493	49.29456	108.4184	8.532782	14.08416	43.51748	5
177.364331	4.594931	1571.563	40.71407	101.4825	8.860648	11.63259	36.11913	5
146.491352	5.307658	1222.858	44.30645	101.5533	8.347646	12.65898	38.99879	5
164.673652	4.691557	1293.22	36.84387	89.67925	7.853228	10.52682	32.15231	5
157.034933	4.907342	1067.37	33.35531	79.33274	6.797021	9.530088	28.44796	5
145.256171	5.262905	1097.233	39.75482	91.1207	7.553779	11.35852	34.49191	5
147.825748	5.223525	1213.029	42.86321	98.86244	8.205803	12.24663	37.63969	5
161.486749	4.708068	1264.067	36.85327	89.1866	7.827684	10.52951	32.14521	5
165.706713	4.720989	1010.751	28.79633	70.09128	6.09964	8.227524	24.07535	5
135.306404	5.709131	960.5431	40.52924	89.42431	7.099021	11.57978	34.82011	5
144.785611	5.323	1152.885	42.38546	96.79639	7.962701	12.11013	37.06246	5
171.538895	4.550103	1227.775	32.56697	80.69797	7.157412	9.304847	28.01686	5
148.655633	5.179639	1172.25	40.84495	94.53853	7.885676	11.66999	35.66531	5
166.840043	4.712996	1281.352	36.19638	88.29089	7.680121	10.34182	31.48338	5
154.838284	5.093365	1066.93	35.09639	82.41018	6.89061	10.02754	30.00303	5
163.158012	4.661657	1302.892	37.22549	90.54354	7.985463	10.63586	32.56384	5
151.270653	5.180502	941.9122	32.25727	74.98481	6.226668	9.216362	27.07676	5
142.575887	5.421136	1175.397	44.6919	101.2087	8.244009	12.76911	39.27076	5
156.486035	4.967811	1128.651	35.8302	84.8842	7.212473	10.2372	30.86239	5
216.754105	4.423553	1830.652	37.36024	98.8459	8.445753	10.67435	32.93668	5
222.746767	4.194854	2449.302	46.12622	124.5149	10.9959	13.17892	41.93136	5
216.551346	4.464976	1935.303	39.90315	105.3035	8.936923	11.4009	35.43817	5
202.115841	4.491463	1745.018	38.77818	100.4363	8.633751	11.07948	34.28671	5
174.987603	5.161876	1431.535	42.22818	101.8949	8.180782	12.0652	37.06631	5
194.613812	4.589948	2150.379	50.71648	129.4168	11.04947	14.49042	46.12653	5
178.495634	5.013922	1424.812	40.0228	97.76197	7.982333	11.43508	35.00888	5
206.257855	4.426134	1822.136	39.10163	102.1625	8.834263	11.1719	34.6755	5
228.95035	4.009638	1773.382	31.05747	85.3739	7.745704	8.873563	27.04783	5
182.565722	5.029359	1327.873	36.58053	89.78972	7.273398	10.45158	31.55117	5
180.210758	4.978198	1445.624	39.93436	97.95438	8.021851	11.40982	34.95616	5
152.829696	5.789004	1236.972	46.85501	106.208	8.093796	13.38715	41.06601	5
198.110156	4.694553	1377.502	32.64223	83.19705	6.953213	9.326352	27.94768	5
183.812396	4.91477	1606.858	42.96412	106.2487	8.741837	12.27546	38.04935	5
190.451533	4.7376	1408.65	35.04104	88.23351	7.39637	10.01173	30.30344	5
167.058223	5.477319	1337.229	43.84358	103.0341	8.00457	12.52674	38.36627	5
171.036411	5.31169	1275.601	39.61494	94.36761	7.458067	11.31855	34.30325	5
170.622856	5.154769	1282.521	38.74686	92.93805	7.516702	11.07053	33.5921	5
224.003629	4.218524	1882.685	35.45546	95.70988	8.404708	10.13013	31.23694	5
168.370868	5.448895	1287.469	41.66566	98.23537	7.646625	11.90447	36.21676	5
159.719797	5.56515	1105.496	38.51904	89.15504	6.921474	11.00544	32.95389	5
162.38373	5.523256	927.7417	31.55584	73.47957	5.713268	9.015955	26.03258	5
179.647827	5.060502	1378.417	38.82863	94.77836	7.672882	11.0939	33.76813	5
193.190279	4.643997	1519.167	36.51843	92.74391	7.863577	10.43384	31.87443	5

167.35141	5.398433	1369.23	44.16871	104.221	8.181765	12.61963	38.77027	5
169.583578	5.299487	1471.072	45.97099	109.3381	8.674612	13.13457	40.67151	5
179.139139	5.08918	1326.437	37.68287	91.78667	7.404507	10.76653	32.59369	5
216.353931	4.442586	1295.562	26.60293	70.27675	5.988163	7.600836	22.16034	5
201.867056	4.546555	1533.868	34.54658	89.17659	7.598409	9.870453	30.00003	5
298.651768	3.452622	2624.127	30.33672	92.51725	8.786577	8.667635	26.8841	5
206.795261	4.390558	1435.759	30.4832	79.85752	6.9429	8.709486	26.09264	5
187.992497	4.82032	1333.965	34.20422	85.47633	7.09584	9.772635	29.3839	5
174.769604	5.125208	1358.933	39.8514	96.30131	7.775567	11.38611	34.72619	5
178.134338	5.193421	1344.434	39.19632	94.85687	7.547302	11.19895	34.0029	5
209.450671	4.354484	1789.452	37.20274	97.97408	8.543547	10.62935	32.84825	5
207.669506	4.34455	1556.54	32.5636	85.62278	7.495274	9.303884	28.21905	5
207.665842	4.514475	1841.044	40.0227	104.2307	8.865416	11.43506	35.50822	5
225.733077	4.141891	1863.426	34.1913	92.8998	8.254998	9.768944	30.04941	5
240.076031	3.948619	2177.954	35.82161	100.0279	9.071933	10.23475	31.87299	5
198.22611	4.653195	1765.78	41.45023	105.8959	8.907907	11.84292	36.79704	5
159.015873	4.892796	1317.095	40.52601	96.76197	8.282792	11.57886	35.63321	5
172.307014	4.418129	1952.877	50.07377	125.1343	11.33371	14.30679	45.65564	5
208.073623	3.394349	2153.477	35.13013	98.29804	10.34959	10.03718	31.73578	5
160.199906	4.914108	1697.263	52.06329	124.4045	10.59466	14.87523	47.14918	5
225.246	3.798415	2306.873	38.90174	107.9525	10.24157	11.11478	35.10333	5
162.512731	4.724207	1529.757	44.46969	107.697	9.413154	12.70563	39.74548	5
204.008865	3.440284	2159.244	36.41221	101.0441	10.58407	10.40349	32.97193	5
181.162232	4.323681	1612.848	38.49278	97.93385	8.902781	10.99794	34.1691	5
184.885764	3.773179	1708.824	34.87395	92.2678	9.242591	9.963986	31.10077	5
184.226408	4.344962	1622.45	38.26532	97.64429	8.806825	10.93295	33.92036	5
195.802523	3.599311	2391.88	43.96837	119.4098	12.21578	12.56239	40.36906	5
172.111653	4.147269	1623.058	39.10982	99.2654	9.43026	11.17424	34.96255	5
195.028366	3.618337	1889.469	35.05509	94.98352	9.688176	10.01574	31.43675	5
186.762035	4.323195	1630.704	37.74779	96.77483	8.731456	10.78508	33.42459	5
182.293535	3.997665	1473.741	32.31888	83.98418	8.084438	9.233965	28.32121	5
170.226957	4.600729	1605.746	43.39854	107.0349	9.432971	12.39958	38.79781	5
190.43013	3.283278	1543.723	26.61591	73.45109	8.106504	7.604544	23.33263	5
163.673793	4.623553	1271.727	35.92448	87.62768	7.769885	10.26414	31.30093	5
179.058315	4.62683	1267.623	32.75511	81.69714	7.079386	9.358604	28.12828	5
191.826874	3.539241	1765.335	32.57075	88.37463	9.20275	9.30593	29.03151	5
180.511968	3.932723	1561.983	34.03013	88.57613	8.65307	9.722893	30.0974	5
182.690947	4.42351	1303.164	31.5536	79.99013	7.13316	9.015314	27.13009	5
169.633448	4.117317	1514.286	36.75453	93.11832	8.926815	10.50129	32.63721	5
181.33873	4.02975	1740.186	38.6708	100.1582	9.59633	11.0488	34.64106	5
223.316222	2.687319	2209.774	26.59175	80.28736	9.895271	7.597643	23.90443	5
162.779921	4.844641	1373.611	40.88129	98.42591	8.438457	11.68037	36.03665	5
169.72117	3.771582	1653.529	36.74508	95.17049	9.742618	10.49859	32.9735	5
181.758156	4.369186	1294.949	31.12859	79.05562	7.124573	8.893882	26.7594	5
165.052354	4.210519	1340.423	34.19446	85.56128	8.121198	9.769846	29.98394	5
191.826527	3.506883	1945.347	35.56393	96.71783	10.14118	10.16112	32.05704	5
205.376183	3.383463	1996.092	32.88455	91.78868	9.719201	9.395586	29.50109	5

176.498913	4.537247	1490.739	38.32235	95.70608	8.446168	10.94924	33.7851	5
162.088974	5.195159	1455.931	46.66445	110.2871	8.982295	13.3327	41.46929	5
174.537252	4.040214	1490.964	34.51305	88.48187	8.542381	9.860871	30.47284	5
199.163426	4.620961	1975.887	45.84424	117.4638	9.920932	13.09835	41.22328	5
248.889081	3.919513	2781.329	43.80045	123.6437	11.17497	12.51441	39.88094	5
235.420373	4.065982	2236.759	38.63142	106.564	9.501129	11.03755	34.56544	5
222.403987	4.188399	1858.01	34.99077	94.45547	8.354211	9.997363	30.80237	5
210.734266	4.464709	2060.521	43.6551	114.4249	9.777814	12.47288	39.19039	5
179.069905	5.044223	1397.863	39.37643	96.11551	7.806244	11.25041	34.33221	5
236.047055	4.028107	2244.901	38.30888	105.9922	9.510394	10.9454	34.28078	5
222.796735	4.227642	2226.802	42.2543	113.8475	9.994768	12.07266	38.02666	5
198.725956	4.754209	1852.721	44.32347	112.701	9.322996	12.66385	39.56926	5
196.02302	4.623184	1935.968	45.65963	116.5129	9.87623	13.04561	41.03645	5
211.809078	4.394379	1410.631	29.2662	77.11308	6.659917	8.361771	24.87182	5
241.557221	4.005924	2253.518	37.37177	104.1413	9.329127	10.67765	33.36585	5
181.267054	5.049222	1289.815	35.92798	87.94405	7.115549	10.26514	30.87876	5
211.939411	4.38798	1770.817	36.66288	96.65257	8.3553	10.47511	32.2749	5
198.271358	4.610962	1535.415	35.70733	91.43752	7.744008	10.20209	31.09637	5
224.081024	4.172831	2163.498	40.28861	109.0626	9.654981	11.51103	36.11577	5
191.460831	4.762707	1554.204	38.6618	97.35058	8.117609	11.04623	33.89909	5
260.129716	3.76454	2322.789	33.61489	96.91727	8.929348	9.604254	29.85035	5
221.538652	4.252181	1796.187	34.47576	92.62394	8.107782	9.850217	30.22358	5
258.547896	3.774422	2361.2	34.47007	99.16645	9.132543	9.848591	30.69565	5
197.480448	4.624835	1414.241	33.1204	84.66474	7.161424	9.462972	28.49557	5
236.679678	4.004732	2022.816	34.22701	94.8999	8.54664	9.779144	30.22227	5
181.555674	5.071388	1309.67	36.58296	89.48486	7.213599	10.45227	31.51157	5
214.926942	4.272901	1760.689	35.00375	93.21946	8.192034	10.00107	30.73085	5
259.154313	3.77776	2105.727	30.69573	88.34032	8.125379	8.770208	26.91797	5
244.924398	3.969601	2289.28	37.10341	103.9885	9.346886	10.60097	33.13381	5
207.505806	4.424431	1941.715	41.40118	108.3443	9.357403	11.82891	36.97675	5
232.781036	4.083878	2267.14	39.77439	109.2879	9.739369	11.36411	35.69052	5
216.5016	4.28716	1877.572	37.17963	99.11239	8.672322	10.62275	32.89247	5
218.705911	4.263273	1786.163	34.81799	93.18221	8.166962	9.947997	30.55471	5
293.345666	3.504727	2310.551	27.60515	83.49711	7.876547	7.887185	24.10042	5
266.344486	3.761928	2504.679	35.37682	102.6188	9.403907	10.10766	31.61489	5
226.961985	4.141642	1991.986	36.35012	98.90106	8.77674	10.38575	32.20848	5
303.297821	3.454417	3094.702	35.24718	107.8942	10.20351	10.07062	31.79276	5
250.242381	3.903937	2298.735	35.86171	101.4718	9.186036	10.2462	31.95777	5
233.65109	4.084809	1938.604	33.89168	93.20563	8.297005	9.683338	29.80687	5
227.251827	4.109436	1889.879	34.17503	93.19447	8.316232	9.764294	30.06559	5
257.364764	3.824142	2400.385	35.66694	102.1574	9.326782	10.19055	31.8428	5
244.976809	3.919629	2209.123	35.34597	99.3825	9.017683	10.09885	31.42634	5
229.457886	4.141839	2101.894	37.94033	103.5091	9.160261	10.84009	33.79849	5
227.849222	2.848115	2221.855	27.77318	83.0612	9.751426	7.935196	24.92507	5
217.193754	3.098342	2358.143	33.63969	97.3378	10.85732	9.611341	30.54135	5
214.315667	3.088122	2821.116	40.65009	117.3279	13.16337	11.61431	37.56197	5
179.827614	3.892373	1541.443	33.36458	86.98535	8.571784	9.532736	29.4722	5

212.93884	3.374625	1958.752	31.04202	87.48979	9.198658	8.869149	27.6674	5
222.407637	3.10192	2458.632	34.29055	99.78246	11.05462	9.797299	31.18863	5
189.14276	3.568731	1666.991	31.45265	84.86454	8.813399	8.986472	27.88392	5
177.789267	3.995264	1498.515	33.67449	86.97433	8.428602	9.621284	29.67923	5
187.055277	3.921494	1929.587	40.45256	106.3104	10.3156	11.55787	36.53106	5
207.207927	3.315327	1923.263	30.77221	86.52242	9.281802	8.792059	27.45688	5
174.371317	4.305465	1702.935	42.04778	106.0735	9.766142	12.01365	37.74231	5
208.174322	3.202682	2050.689	31.54905	89.58075	9.850823	9.014015	28.34637	5
239.031922	2.575775	2241.308	24.15202	74.96189	9.376605	6.900579	21.57625	5
207.815108	3.362704	1791.099	28.98218	81.26024	8.618712	8.280622	25.61947	5
192.380409	3.411	1783.803	31.62771	86.67375	9.27227	9.036489	28.21671	5
191.015172	3.673369	1487.143	28.5989	76.79804	7.785469	8.171114	24.92553	5
199.609807	3.603065	1872.342	33.79679	92.20467	9.380011	9.656226	30.19373	5
187.570572	3.81241	1704.13	34.63679	91.73371	9.085275	9.896227	30.82438	5
174.138411	4.216426	1227.574	29.72334	75.35033	7.049416	8.492384	25.50692	5
267.664488	2.457892	2428.288	22.29833	72.03263	9.072133	6.370951	19.84043	5
190.79559	3.655088	2073.046	39.71353	106.7471	10.86527	11.34672	36.05844	5
204.099362	3.453458	1776.135	30.05304	83.3269	8.702303	8.586582	26.59958	5
182.939996	4.148299	1002.787	22.73894	58.5976	5.481509	6.496839	18.59064	5
225.809834	2.829697	2500.841	31.33886	93.66643	11.07499	8.953961	28.50917	5
236.569124	2.66107	2466.553	27.74525	85.19499	10.42635	7.927214	25.08418	5
200.797518	3.637636	1777.303	32.19752	87.76213	8.85122	9.199291	28.55988	5
168.87331	4.31901	1559.422	39.88292	99.73123	9.234273	11.39512	35.56391	5
210.565499	3.480421	2181.371	36.05572	100.5572	10.35958	10.30163	32.5753	5
184.574644	3.626221	1892.885	37.18831	99.33124	10.25539	10.62523	33.56209	5
189.158861	3.908241	1675.827	34.62452	91.32614	8.859362	9.89272	30.71628	5
191.455029	3.783696	1808.955	35.7501	95.34873	9.44846	10.21432	31.96641	5
215.822965	2.928398	1881.644	25.53113	74.80607	8.718461	7.294608	22.60273	5
194.832658	3.424124	2213.213	38.89653	106.8289	11.35956	11.11329	35.47241	5
193.918521	3.624645	1689.544	31.58026	85.40911	8.712649	9.022932	27.95562	5
236.18927	4.051274	2444.536	41.93029	115.8631	10.3499	11.98008	37.87901	5
261.945997	3.769007	2517.581	36.22419	104.5911	9.611069	10.34977	32.45518	5
253.342951	3.867831	3256.201	49.71299	141.4263	12.85294	14.20371	45.84516	5
269.185611	3.707791	2401.329	33.07616	96.54933	8.920719	9.450332	29.36837	5
264.402063	3.734492	2435.311	34.39705	99.77679	9.210636	9.827729	30.66256	5
302.331328	3.475073	3342.01	38.4139	117.319	11.05413	10.9754	34.93883	5
223.622762	4.211351	1727.041	32.52432	87.79743	7.723012	9.292662	28.31297	5
276.241953	3.629986	2341.098	30.76344	90.86175	8.47481	8.789555	27.13346	5
223.184765	4.203103	1517.888	28.58547	77.16475	6.80104	8.167277	24.38237	5
301.396982	3.488391	2303.239	26.65786	81.2744	7.641879	7.616532	23.16947	5
243.600274	3.967431	1821.779	29.67066	83.05568	7.478558	8.477332	25.70323	5
267.237775	3.737591	3067.833	42.90675	124.7677	11.47979	12.25907	39.16916	5
233.517786	4.082479	2049.159	35.82446	98.52098	8.775175	10.23556	31.74198	5
240.317654	3.939634	2104.9	34.50655	96.43485	8.758822	9.859015	30.56692	5
297.554943	3.476109	2338.463	27.31849	83.09504	7.858927	7.805283	23.84238	5
243.408091	3.919615	1962.652	31.6047	88.72062	8.063216	9.029915	27.68509	5
261.108089	3.756951	2435.156	35.03822	101.1668	9.326238	10.01092	31.28127	5

252.502073	3.849117	2191.522	33.40734	95.07534	8.679223	9.544955	29.55823	5
286.368468	3.575137	2319.786	28.96113	86.64103	8.100705	8.274608	25.38599	5
248.776042	3.881062	2646.964	41.2943	116.8435	10.63995	11.79837	37.41324	5
274.25306	3.651838	2092.947	27.8688	82.04049	7.631444	7.962514	24.21696	5
277.883874	3.627727	2734.318	35.69605	105.6033	9.839785	10.19887	32.06833	5
267.006284	3.729138	2407.027	33.61769	97.79038	9.014869	9.605054	29.88855	5
267.925779	3.685361	2473.985	34.03006	99.36795	9.233846	9.722874	30.3447	5
249.417576	3.878967	2512.83	39.07978	110.6636	10.07479	11.16565	35.20081	5
289.829113	3.525902	2277.417	27.7058	83.42355	7.857792	7.915943	24.1799	5
319.101172	3.383894	2230.883	23.6573	73.72132	6.991147	6.759228	20.27341	5
308.466427	3.423601	2464.023	27.34765	84.25608	7.987979	7.813615	23.92405	5
244.900635	3.91841	2047.736	32.76378	92.12213	8.361497	9.361079	28.84537	5
227.587445	4.145491	1817.646	33.10831	90.12186	7.986585	9.459518	28.96282	5
268.822566	3.733647	2221.413	30.85296	89.87315	8.263492	8.815131	27.11931	5
259.159032	3.822405	1932.037	28.49612	81.76985	7.455025	8.14175	24.67372	5
232.760244	4.033973	1990.01	34.48891	95.05466	8.549613	9.853974	30.45494	5
302.994348	3.427538	1990.01	22.51143	69.02654	6.567813	6.431836	19.08389	5
262.253661	3.778871	1867.009	26.90214	77.64737	7.119095	7.686327	23.12327	5
220.033602	3.138853	2538.155	36.20764	104.7683	11.53531	10.34504	33.06879	5
203.608288	3.399137	2312.21	38.60116	107.3883	11.35617	11.0289	35.20203	5
195.261619	3.712198	1669.762	31.74453	85.49001	8.551411	9.069866	28.03233	5
214.912993	3.342348	2046.561	31.82833	90.12941	9.522744	9.093807	28.48598	5
191.287012	3.758095	1640.322	32.22637	86.07772	8.575187	9.207533	28.46827	5
205.981976	3.32229	2105.387	33.95785	95.28798	10.22122	9.702244	30.63556	5
204.795652	3.401921	1891.004	31.41202	87.49737	9.233612	8.974863	28.0101	5
195.401252	3.489308	1734.5	30.97321	84.72922	8.876605	8.849489	27.4839	5
189.868823	3.92291	2347.65	48.50516	127.9379	12.36459	13.85862	44.58225	5
204.047798	3.458437	1972.345	33.42958	92.64968	9.666094	9.551308	29.97114	5
202.80644	3.265804	1551.955	24.99123	70.1553	7.652396	7.140351	21.72542	5
197.903677	3.377196	1762.148	30.07078	83.19919	8.904067	8.591651	26.69358	5
192.450503	3.97625	1792.845	37.04226	97.7032	9.315877	10.5835	33.06601	5
193.029299	3.755434	1889.434	36.75942	98.4259	9.788328	10.50269	33.00398	5
218.79502	3.161778	1842.716	26.62884	76.80311	8.422111	7.608241	23.46706	5
246.681953	2.479216	2365.894	23.77783	75.09793	9.590867	6.793665	21.29861	5
214.49458	2.979091	2197.454	30.52019	88.90381	10.2448	8.720055	27.5411	5
217.424232	2.986597	2163.871	29.7235	86.82259	9.952297	8.492428	26.7369	5
185.260038	3.773117	2472.069	50.34763	133.2752	13.34378	14.38504	46.57451	5
205.58244	3.364688	1930.913	31.6025	88.35513	9.392401	9.029286	28.23781	5
216.666175	3.200387	2059.565	30.42194	87.26379	9.505707	8.691983	27.22155	5
210.396441	3.37715	1686.679	27.07349	76.06175	8.016669	7.735284	23.69634	5
187.301162	4.002162	1790.532	38.25923	100.0686	9.559641	10.93121	34.25707	5
213.183683	3.346683	1901.666	29.85346	84.33922	8.920315	8.52956	26.50678	5
194.910872	3.493026	1863.12	33.38925	91.2568	9.558832	9.539786	29.89622	5
196.318165	3.543649	1545.184	27.89141	76.09355	7.870815	7.968973	24.34776	5
212.020886	3.251854	2151.286	32.99519	93.75889	10.14658	9.427196	29.74333	5
221.581569	3.094715	2080.151	29.05239	84.5104	9.387745	8.300684	25.95768	5
202.922546	3.26242	1837.454	29.54106	82.96093	9.054951	8.440302	26.27864	5

187.452731	3.632805	1246.002	24.14733	64.71897	6.647022	6.899238	20.51453	5
197.156517	3.495683	1823.628	32.33384	88.60883	9.249648	9.238239	28.83815	5
196.254664	3.641088	1874.185	34.77152	94.21518	9.54976	9.93472	31.13043	5
169.384941	5.041218	950.0466	28.2752	68.07544	4.733319	8.078627	23.23398	
137.93758	5.945585	928.2667	40.01149	87.81259	7.866882	11.43186	34.06591	
155.994988	5.324061	1601.327	54.65281	127.1538	9.103542	15.61509	49.32874	
160.061745	5.213738	931.2528	30.33397	71.40256	4.976875	8.666848	25.12023	
157.785369	5.294811	1244.948	41.77676	97.60877	7.690506	11.93622	36.48195	
137.953042	6.02415	955.1723	41.71058	91.24416	5.292271	11.91731	35.68643	
145.441344	5.659196	769.0532	29.92425	67.3762	4.133881	8.549785	24.26505	
156.367402	5.318619	1345.009	45.74862	106.5283	8.601597	13.07103	40.43	
140.501841	5.903439	756.5905	31.78952	70.21472	5.384915	9.082719	25.88608	
139.713998	5.920085	951.4901	40.31738	88.86286	4.452603	11.51925	34.39729	
147.918827	5.581843	752.3977	28.39237	64.41886	3.766764	8.112105	22.81052	
192.552035	4.573683	1045.815	24.8412	63.27661	4.026119	7.097487	20.26752	
161.222002	5.20071	1020.724	32.92659	77.69394	4.142718	9.407596	27.72588	
139.637602	5.607936	903.6077	36.28946	81.06444	4.747802	10.36842	30.68153	
135.183677	5.77708	1181.577	50.49476	111.0581	5.555454	14.42707	44.71768	
164.673652	4.691557	1458.646	41.55686	101.1508	7.635073	11.87339	36.8653	
145.256171	5.262905	1136.857	41.19046	94.41128	6.352371	11.7687	35.92755	
147.825748	5.223525	1121.238	39.61971	91.38141	5.960237	11.31992	34.39618	
161.486749	4.708068	1135.782	33.11318	80.13541	5.207922	9.46091	28.40512	
154.017255	4.984377	1244.676	40.28076	94.97019	7.195205	11.50879	35.29638	
165.706713	4.720989	985.77	28.08461	68.35893	4.374278	8.024176	23.36363	
135.306404	5.709131	937.5848	39.56054	87.28695	4.964233	11.30301	33.85141	
171.538895	4.550103	1159.831	30.76474	76.23221	4.145573	8.789924	26.21463	
210.591732	4.289037	1755.868	35.76106	94.66312	8.337783	10.21745	31.47203	
154.191393	5.410224	1222.616	42.8988	99.11888	6.70179	12.2568	37.48858	
164.801265	5.086459	1015.692	31.34852	74.79167	5.797365	8.956719	26.26206	
151.315443	5.625109	1573.326	58.48796	133.2001	10.39766	16.71085	52.86285	
177.689012	4.828506	1344.742	36.5419	90.0022	9.171375	10.44054	31.7134	
147.131734	5.637231	1055.454	40.43886	91.4027	6.725275	11.55396	34.80163	
153.561269	5.426193	1231.649	43.52118	100.38	5.951904	12.43462	38.09499	
171.374181	4.953011	1462.134	42.25821	102.4897	10.60559	12.07377	37.3052	
150.153313	5.540713	1246.972	46.01372	104.9856	7.63583	13.14678	40.47301	
181.968965	4.70204	1171.365	30.26783	75.49341	7.182311	8.647952	25.56579	
211.954596	4.33445	1429.634	29.23586	77.31133	3.833407	8.353104	24.90141	
170.744057	4.96349	1042.744	30.31232	73.4106	4.053286	8.660663	25.34883	
171.733381	4.992249	1270.677	36.9383	89.45744	5.621622	10.5538	31.94605	
146.744232	5.600925	844.6705	32.23933	72.93926	4.531175	9.211237	26.63841	
155.896496	5.413073	864.5166	30.01794	69.53908	4.204901	8.576553	24.60486	
151.179766	5.107425	1094.847	36.98809	86.27497	7.242023	10.56802	31.88066	
145.301087	5.264532	677.6907	24.55401	56.27944	5.278485	7.015432	19.28948	
145.098966	5.257209	1035.594	37.52151	86.00182	6.224262	10.72043	32.26431	
177.364331	4.594931	1749.242	45.31715	112.956	6.729138	12.94776	40.72222	
171.057897	4.623186	1575.095	42.57014	104.9918	8.38439	12.1629	37.94695	
146.491352	5.307658	1345.528	48.75102	111.7406	11.01304	13.92886	43.44336	

157.034933	4.907342	1018.92	31.84124	75.73165	6.273196	9.097496	26.93389
148.655633	5.179639	954.1257	33.2448	76.94744	6.322234	9.498513	28.06516
166.840043	4.712996	1217.989	34.40647	83.92493	6.995907	9.830421	29.69348
154.838284	5.093365	1050.53	34.55689	81.14338	3.878694	9.873398	29.46353
163.158012	4.661657	1000.936	28.59817	69.55932	3.929867	8.170907	23.93652
151.270653	5.180502	1073.404	36.76042	85.45279	5.891376	10.50298	31.57992
142.575887	5.421136	1254.692	47.70694	108.0365	6.761117	13.63055	42.2858
156.486035	4.967811	1082.46	34.3638	81.41019	5.958396	9.818228	29.39599
211.395037	4.314184	1514.324	30.90458	81.76582	5.334251	8.829879	26.59039
210.952432	4.349535	1922.816	39.64569	104.624	9.654135	11.32734	35.29616
169.770944	5.007992	1233.632	36.39032	87.80836	5.442111	10.39724	31.38233
154.706813	5.390481	1210.153	42.16563	97.59533	12.77838	12.04732	36.77514
177.131702	4.879661	1049.622	28.9152	70.97459	5.775524	8.261486	24.03554
147.976554	5.605172	1258.237	47.66051	108.0338	8.610996	13.61729	42.05533
192.781069	4.568272	1340.046	31.75465	80.93482	6.497181	9.072756	27.18637
161.567989	5.297311	1269.497	41.62287	97.81534	5.570904	11.89225	36.32556
165.859657	5.010866	1158.717	35.00656	83.96659	5.80326	10.00188	29.9957
154.735489	5.39148	984.9734	34.31963	79.43521	4.938818	9.805609	28.92815
157.242023	5.348368	1256.628	42.74244	99.52821	9.05431	12.21213	37.39408
174.367458	4.911759	1402.701	39.5127	96.44813	6.277221	11.28934	34.60094
162.082284	5.228461	1206.585	38.92209	91.841	4.823384	11.1206	33.69363
164.415637	5.137989	1356.913	42.40352	100.8531	5.891509	12.11529	37.26553
173.795061	4.93736	1121.745	31.86777	77.62245	4.477124	9.105077	26.93041
210.908668	4.330773	981.4898	20.15379	53.24012	3.181001	5.758227	15.82302
201.559045	4.459271	1028.392	22.75204	58.99361	3.745785	6.500583	18.29277
160.199906	4.914108	1437.083	44.0823	105.334	8.816035	12.59494	39.1682
225.246	3.798415	1973.93	33.28718	92.37208	7.323466	9.510624	29.48877
181.162232	4.323681	1344.806	32.09561	81.65806	8.758896	9.170173	27.77192
163.003444	4.794219	1327.457	39.04286	94.27822	5.142155	11.1551	34.24864
184.226408	4.344962	1418.726	33.46051	85.38351	7.47178	9.560145	29.11554
186.762035	4.323195	1669.419	38.64396	99.07237	10.94419	11.04113	34.32076
170.226957	4.600729	1769.487	47.82397	117.9495	9.204622	13.66399	43.22324
182.690947	4.42351	1314.111	31.81868	80.66213	7.968476	9.091051	27.39517
194.794557	4.234664	1172.128	25.48104	66.36002	4.271748	7.280296	21.24637
162.779921	4.844641	1151.446	34.26921	82.50665	5.824014	9.791204	29.42457
181.758156	4.369186	1328.356	31.93165	81.0951	6.356186	9.123327	27.56246
176.498913	4.537247	1399.555	35.97829	89.85202	6.327968	10.27951	31.44104
194.613812	4.589948	1907.584	44.99018	114.8046	7.751573	12.85434	40.40024
228.95035	4.009638	1702.916	29.8234	81.98157	7.32467	8.520973	25.81377
183.812396	4.91477	1509.702	40.36636	99.82457	8.349343	11.53324	35.45159
190.451533	4.7376	1612.313	40.10729	100.9903	8.489059	11.45923	35.36969
171.036411	5.31169	1437.033	44.62836	106.3102	5.852567	12.75096	39.31667
168.370868	5.448895	1419.116	45.92609	108.2802	7.551599	13.12174	40.4772
221.117949	4.18784	2212.365	41.90084	112.9487	13.02826	11.97167	37.713
193.190279	4.643997	1289.669	31.00166	78.73327	4.921728	8.857617	26.35766
201.867056	4.546555	1477.893	33.28587	85.92226	7.388763	9.510249	28.73932
187.992497	4.82032	1183.906	30.35657	75.86106	5.128392	8.673307	25.53625

174.769604	5.125208	1166.648	34.21255	82.67496	4.204457	9.775013	29.08734
209.450671	4.354484	1762.97	36.65218	96.52418	7.893882	10.47205	32.2977
159.015873	4.892796	1052.885	32.39646	77.35145	6.279572	9.256133	27.50367
162.512731	4.724207	1572.577	45.71445	110.7116	6.840665	13.06127	40.99024
184.885764	3.773179	1475.969	30.12183	79.69486	7.4206	8.606236	26.34865
172.111653	4.147269	1542.872	37.17764	94.36129	6.721588	10.62218	33.03037
190.43013	3.283278	688.7999	11.87586	32.77344	2.499134	3.393103	8.592582
163.673793	4.623553	971.5976	27.44626	66.94744	4.702359	7.841789	22.82271
179.058315	4.62683	1068.174	27.60139	68.84282	6.235306	7.886111	22.97456
162.93614	4.276539	922.0241	24.20011	60.12409	5.065869	6.914316	19.92357
169.633448	4.117317	1078.588	26.17933	66.32585	4.609923	7.479809	22.06201
181.33873	4.02975	1377.858	30.61906	79.30398	4.825945	8.748303	26.58931
223.316222	2.687319	1647.024	19.81978	59.84103	4.566454	5.662794	17.13246
169.72117	3.771582	1492.922	33.17603	85.92659	5.055309	9.478867	29.40445
174.537252	4.040214	1200.731	27.7947	71.25787	5.292901	7.941341	23.75448
202.115841	4.491463	1650.983	36.68851	95.02399	5.779372	10.48243	32.19705
178.495634	5.013922	1433.386	40.26364	98.35027	6.49787	11.5039	35.24972
206.257855	4.426134	1548.108	33.2212	86.79841	4.638943	9.491771	28.79506
224.003629	4.218524	1795.459	33.81279	91.2756	8.462395	9.660798	29.59427
298.651768	3.452622	1987.293	22.97449	70.06481	7.61411	6.56414	19.52187
206.795261	4.390558	1722.276	36.56637	95.79373	6.846835	10.44753	32.17581
178.134338	5.193421	1309.136	38.16724	92.36646	4.808168	10.90493	32.97382
207.669506	4.34455	1376.725	28.80179	75.73147	4.316143	8.229082	24.45724
207.665842	4.514475	1608.461	34.96654	91.06304	5.11871	9.99044	30.45207
225.733077	4.141891	1811.566	33.23974	90.31433	4.473037	9.497067	29.09784
240.076031	3.948619	1860.496	30.60026	85.44781	5.674104	8.74293	26.65164
198.22611	4.653195	1651.762	38.77375	99.05814	7.183706	11.07821	34.12055
172.307014	4.418129	1466.455	37.60141	93.96589	8.589867	10.74326	33.18328
204.008865	3.440284	2641.161	44.53897	123.5958	10.23161	12.72542	41.09868
195.802523	3.599311	1846.339	33.94006	92.17482	6.129304	9.697159	30.34075
195.028366	3.618337	2086.15	38.70409	104.8707	15.60292	11.05831	35.08575
182.293535	3.997665	1573.028	34.49623	89.64227	7.74301	9.856065	30.49856
191.826874	3.539241	1633.948	30.14665	81.79727	6.943765	8.613327	26.6074
180.511968	3.932723	1127.717	24.56901	63.95003	10.04939	7.019716	20.63628
165.052354	4.210519	1358.587	34.65784	86.72074	6.229008	9.90224	30.44732
191.826527	3.506883	1676.099	30.64167	83.33151	6.444544	8.754763	27.13479
205.376183	3.383463	1955.472	32.21535	89.92076	7.379378	9.204385	28.83188
180.356581	3.870313	1541.238	33.07378	86.41325	8.750868	9.449652	29.20347
162.088974	5.195159	1178.263	37.76485	89.25374	4.959157	10.78996	32.56969
199.163426	4.620961	2005.048	46.52084	119.1974	8.505957	13.29167	41.89988
210.734266	4.464709	1677.222	35.53437	93.13954	5.804131	10.15268	31.06966
236.047055	4.028107	1505.607	25.69296	71.08673	7.843157	7.340845	21.66485
222.796735	4.227642	2126.827	40.35724	108.7362	11.25736	11.53064	36.1296
198.725956	4.754209	1913.507	45.77768	116.3986	6.856821	13.07934	41.02347
196.02302	4.623184	2021.917	47.68672	121.6855	12.18312	13.62478	43.06354
181.267054	5.049222	1256.003	34.98616	85.63867	6.736202	9.996046	29.93694
211.939411	4.38798	1894.524	39.2241	103.4046	9.105863	11.20688	34.83612

191.460831	4.762707	1732.05	43.08582	108.4903	6.955694	12.31023	38.32311
221.538652	4.252181	2192.557	42.08362	113.0635	10.26023	12.02389	37.83144
258.547896	3.774422	1128.724	16.47773	47.40454	4.148206	4.707922	12.7033
197.480448	4.624835	1391.162	32.57991	83.2831	7.044557	9.308546	27.95508
236.679678	4.004732	2098.935	35.51498	98.47102	9.611672	10.14714	31.51025
181.555674	5.071388	1567.308	43.77955	107.0883	5.470204	12.50844	38.70817
218.705911	4.263273	1881.512	36.67665	98.15649	8.315211	10.47904	32.41338
217.193754	3.098342	2011.512	28.69489	83.02981	8.107067	8.19854	25.59655
214.315667	3.088122	2467.845	35.55972	102.6356	11.76618	10.15992	32.4716
212.93884	3.374625	1960.521	31.07007	87.56882	10.83739	8.877162	27.69544
177.789267	3.995264	1955.178	43.9366	113.4792	11.87191	12.55331	39.94133
207.815108	3.362704	1523.241	24.64791	69.10784	4.689481	7.042261	21.28521
192.380409	3.411	1587.452	28.14631	77.13319	4.883487	8.041803	24.73531
191.015172	3.673369	1593.489	30.64402	82.28989	8.342211	8.755433	26.97065
199.609807	3.603065	2035.214	36.73671	100.2254	12.57299	10.4962	33.13364
174.138411	4.216426	1276.515	30.90835	78.35438	6.789335	8.830956	26.69192
225.809834	2.829697	2447.486	30.67024	91.66804	7.624019	8.762927	27.84055
168.87331	4.31901	1419.061	36.29311	90.75454	5.244579	10.36946	31.9741
189.158861	3.908241	1514.439	31.29007	82.53115	6.684866	8.94002	27.38183
222.403987	4.188399	1852.469	34.88642	94.17378	6.569614	9.967548	30.69802
179.069905	5.044223	1106.532	31.16992	76.0839	7.917789	8.905691	26.1257
211.809078	4.394379	1746.887	36.24247	95.49475	11.36955	10.35499	31.84809
191.639383	4.720182	1696.682	41.79019	105.4887	10.16884	11.94005	37.07
241.557221	4.005924	1919.763	31.83686	88.71756	8.382516	9.096247	27.83094
198.271358	4.610962	1557.245	36.215	92.73755	7.976656	10.34714	31.60404
224.081024	4.172831	1691.307	31.49547	85.25927	10.27092	8.998705	27.32264
244.924398	3.969601	2052.715	33.26929	93.2427	7.1657	9.505512	29.29969
207.505806	4.424431	1794.89	38.27058	100.1517	5.305806	10.93445	33.84615
232.781036	4.083878	2476.511	43.44756	119.3807	8.261514	12.41359	39.36368
216.5016	4.28716	2467.517	48.86172	130.2542	8.674269	13.96049	44.57456
293.345666	3.504727	2196.743	26.24543	79.38438	8.144922	7.498694	22.7407
179.827614	3.892373	1646.758	35.64412	92.92839	11.01263	10.18403	31.75175
187.055277	3.921494	1726.24	36.18952	95.10699	7.549505	10.33986	32.26803
174.371317	4.305465	1586.404	39.17048	98.81494	7.100331	11.19156	34.86501
187.570572	3.81241	1706.683	34.68868	91.87113	5.329468	9.911052	30.87627
267.664488	2.457892	1557.417	14.30135	46.19916	3.876912	4.086101	11.84346
204.099362	3.453458	1636.064	27.68297	76.75552	6.95212	7.909421	24.22952
184.574644	3.626221	1349.45	26.51178	70.81387	5.168782	7.574794	22.88556
191.455029	3.783696	2062.141	40.75377	108.694	6.903244	11.64393	36.97007
215.154713	3.100212	1444.48	20.81383	60.07474	5.396274	5.946809	17.71362
194.832658	3.424124	1728.651	30.38051	83.43979	6.619328	8.680147	26.95639
248.889081	3.919513	2810.492	44.25972	124.9402	11.72979	12.64563	40.34021
235.420373	4.065982	3167.421	54.70502	150.9026	11.47869	15.63001	50.63904
260.129716	3.76454	2521.437	36.48968	105.2058	9.230713	10.42562	32.72514
214.926942	4.272901	1704.309	33.88289	90.23446	6.275339	9.680825	29.60999
266.344486	3.761928	3169.965	44.77352	129.8762	8.31228	12.79243	41.01159
203.63931	4.525318	2081.756	46.26125	119.8176	10.22276	13.2175	41.73593

226.961985	4.141642	2198.992	40.12759	109.1788	6.984513	11.46503	35.98595
303.297821	3.454417	2453.227	27.94108	85.52968	7.904974	7.983167	24.48667
250.242381	3.903937	2456.615	38.32473	108.441	7.472897	10.94992	34.4208
233.65109	4.084809	1509.275	26.38592	72.56401	8.279843	7.538835	22.30111
227.251827	4.109436	1886.112	34.1069	93.00869	8.299655	9.744829	29.99747
257.364764	3.824142	2318.877	34.45583	98.68847	5.546942	9.844522	30.63168
244.976809	3.919629	2718.845	43.50152	122.3135	11.91526	12.42901	39.58189
229.457886	4.141839	2433.142	43.91953	119.8216	9.968722	12.54844	39.77769
227.849222	2.848115	1694.936	21.1867	63.363	5.508038	6.053342	18.33858
222.407637	3.10192	2014.222	28.09236	81.74628	9.056443	8.026388	24.99044
189.14276	3.568731	1409.868	26.60129	71.77475	7.732657	7.600369	23.03256
208.521253	3.098384	1501.478	22.31022	63.90099	9.551549	6.374348	19.21184
207.207927	3.315327	1070.941	17.13505	48.17875	4.241584	4.89573	13.81973
208.174322	3.202682	1500.217	23.08026	65.53436	7.747792	6.59436	19.87758
239.031922	2.575775	1960.37	21.12467	65.56575	7.859676	6.035621	18.5489
190.79559	3.655088	2199.936	42.14437	113.281	11.53033	12.04125	38.48928
182.939996	4.148299	890.1211	20.18415	52.01399	3.74547	5.7669	16.03585
236.569124	2.66107	1905.049	21.42912	65.80059	5.47458	6.122606	18.76805
200.797518	3.637636	1316.072	23.84188	64.98682	5.13722	6.811966	20.20424
210.565499	3.480421	1656.201	27.37523	76.34782	6.270067	7.821494	23.89481
215.822965	2.928398	1228.572	16.6699	48.84273	3.689443	4.762829	13.7415
193.918521	3.624645	1284.687	24.01285	64.94296	6.624882	6.860813	20.3882
261.945997	3.769007	2741.267	39.4427	113.884	11.1178	11.26934	35.67369
253.342951	3.867831	3370.486	51.45779	146.39	13.30404	14.70223	47.58996
264.402063	3.734492	2552.91	36.05805	104.5949	9.085309	10.3023	32.32356
223.622762	4.211351	1773.887	33.40654	90.17893	7.946538	9.544725	29.19518
276.241953	3.629986	3482.573	45.76312	135.1642	10.74967	13.07518	42.13313
233.517786	4.082479	2393.45	41.84352	115.074	9.254495	11.95529	37.76105
243.408091	3.919615	2297.725	37.0004	103.8674	8.356253	10.57154	33.08079
252.502073	3.849117	2051.621	31.27471	89.00598	6.100117	8.93563	27.42559
286.368468	3.575137	2229.151	27.8296	83.25591	5.310596	7.951313	24.25446
274.25306	3.651838	1837.033	24.46116	72.00903	4.80607	6.988902	20.80932
277.883874	3.627727	2476.289	32.32753	95.63784	7.473275	9.236438	28.69981
308.466427	3.423601	2586.91	28.71154	88.45812	8.680959	8.203298	25.28794
218.629432	3.312567	615.4823	9.32549	26.58016	2.432187	2.664426	6.012923
214.912993	3.342348	2585.373	40.20798	113.8584	12.4267	11.48799	36.86563
191.287012	3.758095	1812.069	35.60057	95.09034	6.954233	10.17159	31.84248
204.795652	3.401921	1118.977	18.58765	51.77542	3.309545	5.310757	15.18573
195.401252	3.489308	2073.235	37.02206	101.2762	9.636881	10.57773	33.53275
189.868823	3.92291	2383.728	49.25057	129.904	11.83824	14.07159	45.32767
204.047798	3.458437	2088.926	35.40552	98.12597	7.689804	10.11586	31.94708
223.733941	2.928455	2112.827	27.6548	81.76057	7.644565	7.901372	24.72635
246.681953	2.479216	1635.355	16.43573	51.90924	6.321583	4.695922	13.95651
214.49458	2.979091	1396.604	19.39728	56.50331	4.97812	5.54208	16.41819
205.58244	3.364688	1195.653	19.56879	54.71094	6.70206	5.591082	16.2041
216.666175	3.200387	1574.006	23.24972	66.69065	9.421672	6.642778	20.04934
213.183683	3.346683	1602.833	25.16222	71.08595	6.898415	7.189205	21.81553

236.18927	4.051274	2856.148	48.99054	135.3722	16.77344	13.9973	44.93926
269.185611	3.707791	2798.96	38.55317	112.5367	8.931756	11.01519	34.84537
267.237775	3.737591	3323.186	46.47813	135.1528	10.21594	13.27947	42.74054
297.554943	3.476109	1930.384	22.55122	68.59436	6.728419	6.443205	19.07511
261.108089	3.756951	2627.16	37.80086	109.1435	10.4728	10.80025	34.04391
248.776042	3.881062	2235.059	34.86832	98.66099	9.428508	9.962376	30.98725
267.925779	3.685361	2507.899	34.49655	100.7301	9.437316	9.856156	30.81119
249.417576	3.878967	1943.719	30.22892	85.60031	6.313402	8.636833	26.34995
289.829113	3.525902	2251.537	27.39096	82.47554	5.823719	7.825988	23.86506
319.101172	3.383894	2138.03	22.67265	70.65292	7.177051	6.477899	19.28875
192.450503	3.97625	1527.978	31.56979	83.26894	6.665941	9.019941	27.59354
193.029299	3.755434	1640.79	31.92198	85.47333	8.500211	9.120566	28.16655
218.79502	3.161778	1682.336	24.31121	70.11858	7.700151	6.94606	21.14943
217.424232	2.986597	1373.511	18.86691	55.11041	4.805842	5.390546	15.88031
185.260038	3.773117	2012.588	40.98957	108.5035	8.43119	11.7113	37.21645
210.396441	3.37715	1748.147	28.06015	78.83371	6.627294	8.017185	24.683
221.581569	3.094715	1636.946	22.86237	66.50427	5.778461	6.532106	19.76766
202.922546	3.26242	1692.868	27.21652	76.43287	8.604857	7.776149	23.9541
197.156517	3.495683	1459.738	25.88188	70.92766	5.592734	7.394823	22.3862
196.254664	3.641088	1215.943	22.55924	61.12539	7.072557	6.445497	18.91815
302.331328	3.475073	2493.677	28.66295	87.53883	7.228284	8.189415	25.18788
223.184765	4.203103	1910.89	35.98663	97.14373	8.860146	10.28189	31.78352
301.396982	3.488391	1887.629	21.84756	66.60877	6.914269	6.24216	18.35917
243.600274	3.967431	1830.072	29.80574	83.4338	5.683322	8.515926	25.83831
240.317654	3.939634	2466.65	40.43688	113.0082	9.258193	11.5534	36.49725
267.006284	3.729138	2861.597	39.96644	116.2582	9.917022	11.41898	36.2373
244.900635	3.91841	544.6609	8.714574	24.50283	2.135671	2.489878	4.796164
227.587445	4.145491	1954.024	35.59243	96.88369	7.083749	10.16926	31.44694
268.822566	3.733647	2456.08	34.11223	99.36723	8.784161	9.74635	30.37858
259.159032	3.822405	1832.59	27.02935	77.56093	8.245606	7.722671	23.20694
232.760244	4.033973	2348.853	40.70802	112.1951	8.404935	11.63086	36.67405
302.994348	3.427538	2365.804	26.76249	82.06152	8.034799	7.646425	23.33495
262.253661	3.778871	2096.928	30.2151	87.20953	7.486563	8.632886	26.43623
220.033602	3.138853	3003.594	42.84728	123.9803	7.717083	12.24208	39.70843
195.261619	3.712198	2040.475	38.79231	104.4701	13.21387	11.08352	35.08011
205.981976	3.32229	2324.644	37.49426	105.2114	7.790969	10.71265	34.17197
202.80644	3.265804	1538.661	24.77715	69.55436	6.409217	7.079187	21.51135
197.903677	3.377196	1790.021	30.54644	84.51523	7.689103	8.727554	27.16924
187.301162	4.002162	1198.513	25.60924	66.98202	5.901595	7.316927	21.60708
194.910872	3.493026	1392.967	24.96357	68.2284	5.526873	7.132447	21.47054
196.318165	3.543649	2007.785	36.24161	98.87464	6.415043	10.35475	32.69796
222.18528	2.927342	1394.82	18.37707	54.24214	5.724702	5.250593	15.44973
212.020886	3.251854	1634.203	25.06447	71.22303	6.566327	7.161277	21.81262
187.452731	3.632805	1070.706	20.75012	55.61385	7.133779	5.928605	17.11731
131.225032	6.239007	599.5	28.50283	61.03982	2.896135	8.143665	22.26382
125.652752	6.293021	462.83	23.17975	48.99896	2.227822	6.622785	16.88673
112.862778	7.28147	298.58	19.26323	38.22187	2.14297	5.503779	11.98176

138.309994	5.936051	372.17	15.97296	35.09332	2.348076	4.563703	10.03691
127.033029	6.514514	545.33	27.96564	58.76699	2.576322	7.990183	21.45113
136.510756	6.040299	431.83	19.10752	41.66117	2.931039	5.459292	13.06722
132.945081	6.212387	495.25	23.14252	49.77533	3.725222	6.61215	16.93014
150.22095	5.517758	639.08	23.47401	53.62031	3.175553	6.706861	17.95626
120.467547	6.442115	477.5	25.53476	53.09971	2.662689	7.295646	19.09264
152.726621	4.830522	494.33	15.63494	37.0746	2.323525	4.467127	10.80442
134.610211	5.679756	422	17.80591	39.28721	2.935652	5.087402	12.12615
135.553177	5.695512	426.33	17.91303	39.56518	2.179824	5.118007	12.21751
148.2506	5.180508	534.08	18.66303	43.16565	3.658082	5.332295	13.48252
137.42327	5.655279	688.92	28.35062	62.94541	4.337468	8.100176	22.69534
138.975887	5.649426	580	23.57724	52.50814	3.437037	6.736353	17.92781
138.721557	5.909332	704.42	30.00724	66.05071	2.886967	8.573498	24.09791
148.948917	5.49627	436.5	16.10701	36.74999	2.380952	4.602003	10.61074
143.991703	5.744273	453.42	18.08832	40.47379	2.555631	5.168092	12.34405
138.245848	5.958873	574.17	24.74871	54.31559	2.94567	7.071059	18.78983
188.107499	4.57961	642.92	15.65234	39.62541	3.012464	4.472098	11.07273
149.572809	5.529494	483.5	17.87431	40.76346	3.099359	5.106945	12.34481
129.561069	6.413914	650.75	32.21535	68.29685	3.438573	9.204385	25.80143
229.293976	4.117845	884.5	15.88456	43.39162	3.579812	4.538446	11.76672
142.628229	5.809704	520.67	21.20855	47.20891	2.965429	6.059587	15.39885
153.73806	5.381666	547.75	19.17422	44.32859	3.393322	5.478349	13.79256
150.82938	5.489695	549.5	20	45.78934	3.180897	5.714286	14.5103
163.440276	5.091597	645.17	20.09875	47.84046	3.037238	5.742501	15.00716
141.162708	5.436235	557.25	21.45993	48.4433	2.607994	6.131409	16.0237
127.963273	5.993596	408.17	19.11803	41.09535	2.042586	5.462295	13.12444
140.08276	5.544099	584.08	23.11632	51.82712	2.953628	6.604662	17.57222
129.838585	6.006318	501.75	23.2109	50.04847	2.636211	6.631685	17.20458
153.973089	5.405789	597.58	20.98023	48.46818	3.361913	5.994353	15.57444
164.102754	5.084516	645.42	19.99752	47.66425	3.707606	5.713578	14.91301
257.803164	3.880649	491.42	7.397227	21.11859	2.534138	2.113494	3.516578
149.103028	5.563546	352	13.13433	29.8842	1.904453	3.752665	7.570783
151.770889	5.535649	500.92	18.27042	41.80743	3.019045	5.220119	12.73477
148.928487	5.602396	465.67	17.51759	39.77638	2.851099	5.005025	11.91519
175.372551	4.861332	563.17	15.61109	38.2591	2.586315	4.460311	10.74976
169.195547	5.013201	523.33	15.50607	37.37406	2.994735	4.430307	10.49287
156.702661	5.317903	603.42	20.47782	47.71092	3.117161	5.850806	15.15992
162.909392	5.163531	650.5	20.61807	48.86499	3.791234	5.890876	15.45454
137.841503	5.580628	509.42	20.62429	45.97834	3.208945	5.892655	15.04366
139.437492	5.551519	363.75	14.48222	32.42106	2.142857	4.137778	8.930705
136.355385	5.669663	429.42	17.8553	39.54085	2.201251	5.101515	12.18564
155.498682	4.923493	828.5	26.23247	62.18741	2.960726	7.494991	21.30897
146.475821	5.130502	574.92	20.1373	46.54819	4.089629	5.753515	15.0068
160.774817	4.842615	391.25	11.78464	28.28791	2.286942	3.36704	6.942024
149.426896	5.157097	420.58	14.51527	33.67682	2.414767	4.147221	9.358174
156.653677	4.926216	567	17.83019	42.34116	2.962073	5.09434	12.90397
131.032932	6.08974	363.33	16.88572	36.36763	2.756049	4.824491	10.79598

236.08338	3.724711	622.58	9.822508	27.71504	2.707811	2.806431	6.097796
133.293582	5.884926	537.08	23.71214	51.72945	2.722839	6.774898	17.82721
156.723468	4.892563	653.17	20.39053	48.50964	3.90722	5.825867	15.49797
154.797533	4.940399	457.92	14.61462	34.57707	2.863432	4.175607	9.674224
172.036285	4.93294	477.17	13.68229	33.24968	1.885451	3.909227	8.749354
185.204492	4.674874	737	18.60312	46.67199	3.612745	5.315179	13.92825
163.617813	5.129085	491.58	15.41003	36.62277	2.652458	4.402866	10.28095
162.756174	5.142375	505.25	15.96367	37.86396	1.826183	4.561047	10.82129
182.625586	4.740444	521.42	13.53459	33.71947	1.700763	3.867025	8.794144
149.254256	5.543334	422.25	15.68245	35.72341	2.378873	4.4807	10.13912
175.857865	4.925991	519.42	14.54958	35.56457	2.381787	4.157023	9.623589
166.622573	5.056831	557.17	16.90956	40.51316	2.237631	4.831303	11.85273
178.043597	4.828301	700.67	19.00122	46.82357	2.257822	5.42892	14.17292
158.210113	4.870551	750.17	23.09423	55.13376	3.439884	6.598353	18.22368
181.195391	4.343442	820.58	19.67016	49.99034	2.350087	5.620045	15.32672
163.107795	4.769234	501.83	14.67339	35.48437	2.228672	4.192398	9.904158
175.50344	4.394948	792.58	19.84774	49.89352	3.18843	5.670784	15.4528
154.513865	4.997699	461.25	14.91898	35.1794	2.821101	4.262565	9.921277
158.286299	4.850498	675.83	20.71002	49.49881	4.215244	5.917148	15.85952
231.095685	3.748511	580.67	9.418816	26.39244	1.857668	2.69109	5.670305
153.196121	5.043494	534.83	17.60757	41.33601	2.325348	5.030735	12.56408
154.882145	5.028641	577.33	18.74448	44.15817	3.507899	5.355566	13.71584
150.975432	5.139589	395.08	13.44953	31.31134	1.897872	3.842723	8.309943
147.623891	5.23489	454.17	16.10532	37.11347	2.373752	4.60152	10.87043
184.453728	4.34776	650.5	15.33294	39.1319	3.129962	4.38084	10.98518
191.621382	4.158179	884.42	19.19189	50.0038	3.494626	5.483398	15.03371
160.485403	4.831424	534	16.07611	38.5941	2.621888	4.593173	11.24468
171.920178	5.225537	534.08	16.23343	38.87849	2.591615	4.638124	11.0079
202.898097	4.447861	755.75	16.56729	43.05594	2.857278	4.733511	12.11943
195.031801	4.663936	660.58	15.79692	40.17083	2.16463	4.513407	11.13299
156.648556	5.922441	475.83	17.98979	40.79741	2.225272	5.139941	12.06735
182.894255	5.000526	920.92	25.17895	61.92039	2.727278	7.193985	20.17842
213.170411	4.240763	703	13.98532	37.23858	2.608534	3.995805	9.744556
193.425084	4.649641	623.67	14.99207	38.07456	3.010426	4.283448	10.34243
183.665118	4.795434	697.5	18.21149	45.3049	2.671595	5.203282	13.41605
169.501588	5.415386	455.08	14.5393	34.38979	1.606184	4.154085	9.123911
173.206348	5.268634	653.08	19.86555	47.56824	2.869672	5.675872	14.59692
178.013252	5.120179	546.33	15.71404	38.15748	3.07065	4.489725	10.59386
178.664054	5.004595	539.08	15.10028	36.91068	2.591731	4.314366	10.09568
194.730435	4.555098	945.92	22.12678	56.57862	3.318086	6.321938	17.57169
225.114198	3.794081	822.25	13.85822	38.46198	2.954227	3.959493	10.06414
162.17521	4.433076	507.33	13.86792	34.10602	2.356058	3.962262	9.434841
157.020172	4.557915	573.17	16.63774	40.30803	2.801691	4.753639	12.07982
161.831819	4.205281	607.42	15.78411	39.31311	3.858595	4.509746	11.57883
160.096518	4.833102	630	19.01887	45.62718	3.475862	5.433962	14.18577
141.375579	5.882561	441.17	18.35684	40.64433	2.627263	5.244812	12.47428
150.402986	5.469199	440.33	16.012	36.66729	2.593991	4.574857	10.5428

185.975968	3.574193	602.42	11.57765	31.09498	3.082694	3.307901	8.00346
190.674529	3.37776	809.67	14.34314	39.31516	2.783136	4.098039	10.96538
161.21654	4.528554	595.33	16.72275	40.84795	2.647912	4.777929	12.1942
185.515638	4.831136	513.08	13.36146	33.2611	2.062633	3.81756	8.530322
185.792642	4.882855	497	13.06176	32.44071	2.203991	3.731932	8.178906
216.887704	4.136473	883.92	16.85809	45.3638	4.119303	4.816596	12.72161
239.840428	3.915762	673.25	10.99184	30.75012	2.524656	3.140525	7.076075
180.118425	5.014852	714.33	19.88835	48.68821	2.845936	5.682387	14.8735
168.865864	5.408034	504	16.14091	38.1552	1.683254	4.611689	10.73288
194.591056	4.575921	507.17	11.9264	30.45582	2.127212	3.407542	7.350475
195.390398	4.663255	493.25	11.77208	29.95067	2.29504	3.36345	7.108821
175.937533	5.159458	726.75	21.31232	51.50143	3.269966	6.089233	16.15286
176.811435	5.110157	539.5	15.59249	37.81677	1.773679	4.454996	10.48233
173.552921	5.165266	533	15.8631	38.19203	1.944688	4.532313	10.69783
207.953064	4.305446	720.33	14.91366	39.31617	2.578778	4.261047	10.60822
148.471382	5.027304	480.17	16.25876	37.90219	2.529474	4.64536	11.23146
217.756525	2.826795	786.5	10.20991	30.24761	2.937442	2.917117	7.383115
203.73784	3.189634	564.25	8.833659	24.97315	2.345374	2.523903	5.644026
185.912936	3.556441	543.33	10.39369	27.94751	2.517398	2.969625	6.837247
192.807635	3.531275	633.83	11.60861	31.55574	2.365744	3.316745	8.077333
149.333039	5.295498	470.58	16.68723	38.45445	3.002105	4.767781	11.39174
179.79542	4.152319	724.58	16.73395	42.926	2.773512	4.781128	12.58163
172.576349	4.102416	518.33	12.32153	31.3798	2.14186	3.520438	8.219118
204.958319	3.354473	584.75	9.570376	26.75712	2.714465	2.734393	6.215903
201.699296	3.455708	773	13.24379	36.60614	2.730677	3.783939	9.788077
242.301959	3.878383	778.75	12.46499	35.04438	2.157202	3.561425	8.586603
275.926731	3.593966	579	7.541517	22.32357	1.807511	2.154719	3.947552
160.441495	5.736815	485.92	17.37476	39.95585	2.580152	4.964218	11.63795
185.804266	4.93727	551	14.64141	36.26394	2.038853	4.183259	9.704136
193.056639	4.739563	574	14.09177	35.60017	2.92112	4.02622	9.352205
227.884273	4.023878	835.5	14.75288	40.47103	3.191002	4.215109	10.729
182.95186	5.019255	740.42	20.31331	49.912	3.157441	5.803802	15.29405
162.231137	5.636353	542.25	18.83925	43.63626	3.001328	5.382642	13.20289
280.232835	3.548733	824.25	10.4379	31.11535	2.70175	2.982258	6.889171
219.887644	4.16983	702.67	13.32505	35.90786	2.383953	3.807158	9.155223
253.634278	3.845857	838.5	12.71418	36.23197	2.707022	3.632622	8.86832
212.442665	4.310668	782.75	15.88276	42.08237	2.384034	4.537931	11.57209
211.953867	4.345543	679	13.92107	36.78932	2.422837	3.977447	9.575523
186.159241	3.763302	661	13.36244	35.43764	2.974128	3.817841	9.599142
194.269713	3.868605	669.33	13.32875	35.48152	2.467576	3.808215	9.460149
165.895044	4.886452	528.92	15.57938	37.60626	2.571693	4.451252	10.69293
188.851085	3.671788	650.67	12.65083	33.87891	3.323985	3.614522	8.979039
186.976717	3.792631	692	14.03651	37.19383	2.679574	4.010432	10.24388
184.082111	3.939018	599.58	12.82991	33.54512	2.239997	3.665687	8.890888
187.035117	3.828764	798.42	16.34432	43.20987	2.595812	4.669806	12.51556
172.833108	4.241303	438.25	10.7546	27.17229	2.029687	3.072743	6.513298
183.684359	3.909592	651.75	13.87204	36.3183	2.402057	3.96344	9.962447

184.547821	3.68234	497	9.916795	26.38564	2.979081	2.83337	6.234455
174.833487	4.170945	818.58	19.52859	49.68994	3.483319	5.579598	15.35765
196.524127	3.810453	671.42	13.01832	34.8871	2.917061	3.719521	9.20787
195.381188	3.267244	894.25	14.95401	41.58465	3.864186	4.272575	11.68677
208.64483	4.397151	884.5	18.64067	48.92386	3.310626	5.325907	14.24352
205.251048	4.474137	679.67	14.81569	38.55814	2.804729	4.233056	10.34156
217.607937	4.240465	629.75	12.27176	32.84522	2.321659	3.506218	8.031297
244.552522	3.928555	1022.25	16.42169	46.12672	3.969903	4.69191	12.49313
212.263891	4.396973	656.25	13.59399	35.83254	2.352825	3.883998	9.197019
225.05075	4.186991	750.25	13.95814	37.79394	2.890246	3.98804	9.771149
175.581306	5.210128	736.08	21.84214	52.62627	3.281237	6.24061	16.63201
231.778553	4.105909	508	8.999114	24.66697	1.553517	2.571176	4.893205
252.507493	3.823959	673.17	10.19445	29.06056	2.523599	2.912699	6.370489
211.056254	4.406185	722	15.07307	39.65387	2.241679	4.306591	10.66688
187.368653	4.86356	524.25	13.60805	33.90248	2.296824	3.888013	8.744487
201.012197	4.578866	737	16.78815	43.21347	3.175629	4.796616	12.20929
292.779699	3.487549	925.58	11.02537	33.37324	2.518037	3.150106	7.537824
248.234535	3.876666	1003.08	15.66505	44.31316	2.978708	4.475728	11.78838
241.683449	3.882465	650.75	10.45382	29.36362	1.41084	2.986804	6.57135
218.099251	3.238296	723.41	10.74105	30.77027	2.833235	3.068873	7.502758
187.620576	3.804379	697.5	14.1432	37.47975	3.121923	4.040913	10.33882
190.80057	3.840978	641.17	12.9073	34.2665	2.35145	3.687799	9.06632
186.17938	4.13732	664.58	14.76844	38.25057	2.30357	4.219556	10.63112
190.112104	3.554627	459.33	8.588336	23.22539	2.445456	2.45381	5.03371
199.979126	3.341339	704.08	11.76408	32.7208	2.533391	3.361165	8.422738
228.518147	2.857084	629.33	7.868297	23.53045	2.390617	2.248085	5.011213
195.686145	3.614914	794.83	14.68291	39.82701	3.553583	4.195117	11.068
190.694219	3.61047	603.08	11.41829	30.78184	2.773802	3.26237	7.807823
210.675255	4.36859	644.58	13.3661	35.2227	2.476011	3.818885	8.997506
230.807695	4.068315	629.08	11.08843	30.4319	2.205982	3.168123	7.020117
218.55862	4.254845	817.67	15.9182	42.61524	2.67869	4.548056	11.66335
222.67269	4.173809	657.33	12.32109	33.29911	2.4034	3.520311	8.147279
267.453193	3.751097	844.75	11.84783	34.42794	2.650196	3.385093	8.096729
344.174438	3.231685	992.67	9.320845	29.94282	2.949985	2.663099	6.08916
259.208736	3.809092	719	10.56576	30.34644	1.89835	3.018789	6.756668
280.265251	3.600067	791.58	10.16802	30.20305	2.374764	2.905147	6.567948
325.77097	3.346389	1009.25	10.36723	32.56468	2.867188	2.962066	7.020843
196.066885	3.911559	638.17	12.73157	33.87627	2.999624	3.637592	8.820012
182.910449	4.080545	653.83	14.58628	37.74197	2.573323	4.167509	10.50573
188.204871	3.6705	676	13.18381	35.2791	3.039295	3.766804	9.513313
180.877921	3.940695	603.25	13.1427	34.2088	3.398592	3.755058	9.202006
211.62437	3.055948	520.5	7.516245	21.68233	2.025922	2.147499	4.460298
198.747033	3.374313	515.42	8.750764	24.24235	2.766168	2.500218	5.376451
191.446584	4.029012	621.92	13.08837	34.36353	2.704941	3.739534	9.059356
236.505698	2.726825	759.25	8.753877	26.71447	2.232563	2.501108	6.027052
204.640391	3.736564	775.42	14.15853	38.51659	2.723926	4.045293	10.42196
208.090192	3.429587	671.42	11.06584	30.88424	2.256874	3.161669	7.636256

213.500147	2.936728	972.58	13.37799	39.0638	3.169768	3.822283	10.44126
279.371956	3.590115	866.17	11.13086	33.05958	2.81608	3.180245	7.540744
265.688711	3.72113	738.83	10.34776	30.07952	1.909516	2.956503	6.626629
371.972382	3.17518	1259.75	10.75331	35.37754	3.41553	3.072374	7.578127
214.319779	3.145978	617.5	9.06422	26.04097	2.884436	2.589777	5.918242
197.639072	3.665073	572.75	10.62123	28.7821	2.160668	3.034638	6.95616
202.199682	3.280705	702	11.39	31.9137	2.920984	3.254286	8.109297
266.878921	3.7378	887.58	12.43109	36.13548	3.488641	3.551741	8.693292
238.566192	3.996084	717.92	12.02546	33.42684	1.983643	3.435846	8.029377
197.516304	3.649262	622	11.49192	31.17037	2.584452	3.283405	7.842655
205.473961	3.308759	575.75	9.271337	26.02647	1.975671	2.648953	5.962577
214.298455	3.126938	615.5	8.981075	25.84064	2.927467	2.566021	5.854137
146.84804	5.134547	452.7487	15.83037	36.60856	1.624327	4.522964	10.69583
144.461819	5.451389	451.1037	17.02278	38.62264	4.453546	4.863652	11.57139
153.591611	5.035791	519.5375	17.03402	40.03059	2.957909	4.866862	11.99823
145.743192	5.715419	637.2882	24.99169	56.16047	2.918747	7.140484	19.27627
160.892602	5.20688	534.2111	17.28839	40.76093	2.725901	4.939539	12.0815
150.569116	5.556056	240.9517	8.891207	20.28631	1.366689	2.540345	3.335151
139.872091	5.952004	265.221	11.286	24.84889	1.912971	3.224571	5.333996
151.812778	5.40259	427.5275	15.2145	35.02953	3.811391	4.347001	9.811912
153.21141	5.124127	392.5576	13.12902	30.70082	2.211627	3.751148	8.004889
157.31592	4.931534	284.8734	8.930201	21.2231	1.327307	2.551486	3.998667
228.770049	3.985541	2661.203	46.36242	127.6127	6.636432	13.2464	42.37688
192.993905	4.617079	890.1403	21.29522	54.14723	6.136368	6.084349	16.67814
174.066748	5.211579	525.0198	15.71916	37.78904	1.971712	4.491187	10.50758
242.085188	3.87957	468.7838	7.512561	21.11466	1.743903	2.146446	3.632991
310.938039	3.350625	1128.963	12.16555	37.75885	2.70433	3.475871	8.814924
176.719188	5.122295	574.5392	16.65331	40.36039	2.630455	4.758089	11.53101
183.592495	3.497	574.908	10.95063	29.4767	2.997208	3.128751	7.453629
188.070461	3.463544	593.0469	10.92167	29.64757	2.483308	3.120478	7.45813
193.799292	3.347138	550.4185	9.506364	26.2231	2.876397	2.716104	6.159226
205.99426	3.274948	709.1364	11.27403	31.74986	3.293548	3.221151	7.99908
184.87348	3.843523	523.1335	10.87596	28.64203	2.553302	3.107416	7.032433
188.612058	3.423086	665.9435	12.08609	32.92863	5.01105	3.453168	8.663003
180.679638	3.919298	613.7812	13.31413	34.69268	2.456093	3.804036	9.394828
186.252389	4.825191	571.9315	14.81688	36.93206	3.476834	4.233394	9.991687
229.895689	2.594759	834.2711	9.416152	28.88896	3.975459	2.690329	6.821393
156.446477	4.684026	279.3663	8.36426	20.10778	1.892972	2.389789	3.680234
184.279277	3.783969	500.9811	10.28709	27.17532	2.223262	2.939167	6.503118
194.185503	3.269116	508.3223	8.557614	23.75742	2.175377	2.445033	5.288498
179.517535	3.852308	564.5899	12.11566	31.6551	3.080923	3.461618	8.263355
206.277689	3.238268	687.6847	10.79568	30.49894	3.204356	3.084479	7.55741
231.578334	4.055663	569.1401	9.967427	27.39947	2.423155	2.847836	5.911765
164.7121	5.545862	305.6171	10.29014	24.02206	1.200453	2.940039	4.744276
245.674945	3.838671	807.1633	12.61193	35.67192	2.705575	3.603408	8.773256
252.003448	3.761245	983.4539	14.67842	41.99503	2.581786	4.193833	10.91717
200.021626	3.496882	541.5748	9.468091	26.03823	2.946514	2.705169	5.971209

210.179427	2.715496	735.6718	9.504804	28.19216	3.518323	2.715658	6.789307
186.848674	3.908968	613.5908	12.83663	33.75265	2.038157	3.667608	8.92766
233.432702	4.059699	892.6577	15.52448	42.7498	3.238048	4.435566	11.46478
169.384941	5.041218	543.3488	16.17109	38.93357	2.707071	4.620313	11.12988
155.078103	5.329144	535.5164	18.40263	42.74182	2.572539	5.257893	13.07348
137.93758	5.945585	539.1273	23.23825	51.00061	4.569001	6.639499	17.29266
155.994988	5.324061	806.8946	27.53907	64.07168	4.587194	7.868305	22.215
160.061745	5.213738	468.8805	15.27298	35.95079	2.505829	4.363709	10.05924
157.785369	5.294811	738.1494	24.77011	57.87381	4.559825	7.077175	19.4753
137.953042	6.02415	472.2293	20.62137	45.11036	2.616455	5.891819	14.59722
145.441344	5.659196	431.1105	16.77473	37.76929	2.317343	4.79278	11.11553
156.367402	5.318619	721.2283	24.53157	57.12316	4.612395	7.009021	19.21296
140.501841	5.903439	468.1951	19.67206	43.45044	3.332306	5.620589	13.76862
139.713998	5.920085	664.1893	28.14362	62.03087	3.108148	8.041033	22.22353
147.918827	5.581843	541.6566	20.43987	46.37561	2.711721	5.839963	14.85803
192.552035	4.573683	340.6837	8.092249	20.61293	1.311545	2.312071	3.518567
161.222002	5.20071	499.2996	16.10644	38.00493	2.02646	4.601839	10.90573
139.637602	5.607936	451.8046	18.14476	40.53229	2.373905	5.184218	12.53683
146.076673	5.350794	396.9257	14.5394	33.23435	3.078476	4.154115	9.188608
135.183677	5.77708	720.3442	30.78394	67.70616	3.386862	8.795411	25.00686
164.673652	4.691557	693.2772	19.75149	48.0758	3.62886	5.643282	15.05993
145.256171	5.262905	494.2357	17.90709	41.04425	2.761622	5.116311	12.64418
147.825748	5.223525	658.4656	23.26734	53.66526	3.500249	6.64781	18.04381
161.486749	4.708068	612.3141	17.85172	43.20198	2.807654	5.100492	13.14365
154.017255	4.984377	550.7299	17.82297	42.02133	3.183653	5.092278	12.8386
165.706713	4.720989	615.6619	17.54022	42.69351	2.731952	5.011493	12.81923
135.306404	5.709131	488.4657	20.61037	45.47502	2.586281	5.888677	14.90124
144.785611	5.323	478.3932	17.58799	40.16598	2.286121	5.025139	12.26499
171.538895	4.550103	512.4843	13.59375	33.68407	1.831768	3.883928	9.043645
210.591732	4.289037	832.5337	16.95588	44.88391	3.953307	4.844537	12.66684
154.191393	5.410224	488.9905	17.15756	39.64302	2.68041	4.90216	11.74734
164.801265	5.086459	542.9058	16.75635	39.97751	3.098797	4.787529	11.66989
151.315443	5.625109	643.1036	23.9072	54.44609	4.250085	6.830627	18.28209
177.689012	4.828506	565.9929	15.38024	37.88132	3.86017	4.394355	10.55174
147.131734	5.637231	542.2705	20.77665	46.96081	3.455307	5.936185	15.13942
153.561269	5.426193	541.4867	19.1338	44.13142	2.616716	5.466801	13.70761
171.374181	4.953011	703.517	20.33286	49.31369	5.102962	5.809389	15.37985
150.153313	5.540713	441.5534	16.29348	37.17545	2.703852	4.655281	10.75277
181.968965	4.70204	675.4833	17.45435	43.53429	4.141776	4.986957	12.75231
211.954596	4.33445	650.0494	13.29344	35.15319	1.743037	3.798127	8.958994
170.744057	4.96349	581.725	16.91061	40.95424	2.261243	4.831603	11.94712
171.733381	4.992249	496.115	14.42195	34.92718	2.194869	4.120556	9.429698
146.744232	5.600925	434.8939	16.599	37.5541	2.332958	4.742573	10.99808
155.896496	5.413073	744.6758	25.8568	59.89945	3.62201	7.387656	20.44372
151.179766	5.107425	518.5524	17.51866	40.8624	3.430038	5.005332	12.41124
145.301087	5.264532	585.4368	21.21148	48.61813	4.559926	6.060423	15.94695
145.098966	5.257209	356.4938	12.91644	29.60535	2.142646	3.690412	7.659234

177.364331	4.594931	757.718	19.63	48.92909	2.914856	5.608571	15.03507
171.057897	4.623186	843.7559	22.80421	56.24262	4.491397	6.515489	18.18103
146.491352	5.307658	720.837	26.11728	59.86256	5.899993	7.462081	20.80963
157.034933	4.907342	657.3464	20.54208	48.85756	4.047094	5.869164	15.63473
148.655633	5.179639	569.0856	19.82877	45.89509	3.770879	5.665362	14.64913
166.840043	4.712996	488.727	13.80585	33.67549	2.807158	3.944528	9.092852
154.838284	5.093365	611.7172	20.12228	47.24931	2.258541	5.749222	15.02891
163.158012	4.661657	719.8698	20.56771	50.02682	2.826347	5.876488	15.90605
151.270653	5.180502	346.8884	11.87974	27.61548	1.903896	3.394211	6.699238
142.575887	5.421136	367.903	13.98871	31.67864	1.982506	3.996774	8.567572
156.486035	4.967811	564.2437	17.9125	42.43594	3.105878	5.117857	12.94469
211.395037	4.314184	794.5075	16.21444	42.89937	2.798675	4.632697	11.90025
210.952432	4.349535	1129.504	23.28873	61.45841	5.671047	6.653924	18.9392
169.770944	5.007992	489.2883	14.43328	34.82692	2.158473	4.123795	9.425291
154.706813	5.390481	549.4526	19.14469	44.31174	5.801837	5.469911	13.75421
177.131702	4.879661	525.4092	14.47408	35.52775	2.891054	4.135452	9.594421
147.976554	5.605172	514.1425	19.47509	44.1449	3.518636	5.564313	13.86992
192.781069	4.568272	481.4872	11.40965	29.0804	2.334479	3.2599	6.841377
161.567989	5.297311	510.3353	16.7323	39.32156	2.239492	4.780658	11.43499
165.859657	5.010866	674.8458	20.38809	48.90278	3.379863	5.825169	15.37722
154.735489	5.39148	477.4843	16.63708	38.50771	2.394184	4.753453	11.2456
157.242023	5.348368	480.3174	16.33733	38.04239	3.460804	4.667808	10.98896
174.367458	4.911759	534.1608	15.04678	36.72829	2.390421	4.299081	10.13502
162.082284	5.228461	538.6964	17.3773	41.00368	2.153466	4.964944	12.14884
164.415637	5.137989	674.6691	21.08341	50.14508	2.929311	6.023831	15.94542
173.795061	4.93736	616.9398	17.5267	42.69095	2.462337	5.007629	12.58934
210.908668	4.330773	741.9315	15.23473	40.24548	2.404594	4.352781	10.90396
201.559045	4.459271	524.3841	11.60142	30.08124	1.910001	3.314691	7.142147
225.246	3.798415	961.2727	16.21033	44.98374	3.566412	4.631524	12.41192
181.162232	4.323681	579.1961	13.8233	35.16941	3.77238	3.949513	9.499615
163.003444	4.794219	753.8249	22.17132	53.5379	2.920082	6.334663	17.3771
184.226408	4.344962	600.4461	14.16146	36.1368	3.162276	4.046133	9.816502
186.762035	4.323195	726.0345	16.80636	43.08682	4.759654	4.801816	12.48316
170.226957	4.600729	570.5839	15.42119	38.03367	2.968097	4.406053	10.82046
182.690947	4.42351	573.0335	13.8749	35.17365	3.474746	3.964258	9.451393
194.794557	4.234664	650.2901	14.13674	36.81618	2.369943	4.039069	9.902077
162.779921	4.844641	503.5224	14.98579	36.07982	2.546817	4.281653	10.14115
181.758156	4.369186	740.9244	17.81068	45.23284	3.545324	5.088766	13.4415
176.498913	4.537247	649.1505	16.68767	41.67572	2.935077	4.767906	12.15043
194.613812	4.589948	850.1698	20.05118	51.16598	3.454713	5.728907	15.46123
228.95035	4.009638	934.7819	16.37096	45.00214	4.020731	4.677417	12.36132
180.210758	4.978198	465.6193	12.86241	31.55001	2.551567	3.674974	7.884213
183.812396	4.91477	714.3779	19.10101	47.23613	3.950837	5.457432	14.18624
190.451533	4.7376	557.6725	13.87245	34.93089	2.936225	3.963557	9.134849
171.036411	5.31169	548.5626	17.03611	40.58208	2.234117	4.867459	11.72442
168.370868	5.448895	729.2458	23.60019	55.6423	3.880564	6.742911	18.15129
221.117949	4.18784	782.7327	14.82448	39.96116	4.609389	4.235567	10.63664

193.190279	4.643997	701.8675	16.87181	42.84845	2.678517	4.820518	12.22782
201.867056	4.546555	783.4794	17.64593	45.5502	3.917026	5.041695	13.09938
187.992497	4.82032	793.8219	20.35441	50.86565	3.438642	5.815545	15.53409
174.769604	5.125208	636.282	18.6593	45.09038	2.293083	5.331228	13.53409
209.450671	4.354484	844.3004	17.55302	46.2262	3.780443	5.015149	13.19854
159.015873	4.892796	609.1571	18.7433	44.75245	3.633109	5.355227	13.8505
162.512731	4.724207	653.9718	19.01081	46.04052	2.844758	5.43166	14.2866
184.885764	3.773179	867.5143	17.70437	46.84137	4.361525	5.058393	13.93119
172.111653	4.147269	771.4343	18.58878	47.18054	3.360786	5.31108	14.44151
190.43013	3.283278	689.1028	11.88108	32.78785	2.500233	3.394595	8.597804
163.673793	4.623553	536.6826	15.16052	36.97984	2.597448	4.331578	10.53697
179.058315	4.62683	629.132	16.25664	40.54698	3.672465	4.644754	11.62981
162.93614	4.276539	414.9116	10.89007	27.05589	2.279645	3.111448	6.613529
169.633448	4.117317	486.6293	11.81139	29.92439	2.07987	3.374683	7.694073
181.33873	4.02975	631.8922	14.04205	36.36919	2.213202	4.012014	10.0123
223.316222	2.687319	800.4792	9.632722	29.08367	2.219368	2.752206	6.945402
169.72117	3.771582	618.9995	13.75555	35.62714	2.096047	3.930156	9.983964
174.537252	4.040214	507.5444	11.74871	30.12043	2.237289	3.356775	7.708499
222.746767	4.194854	770.8527	14.517	39.18777	2.89356	4.147714	10.32215
202.115841	4.491463	646.6347	14.36966	37.2177	2.263586	4.105617	9.878196
178.495634	5.013922	568.7331	15.97565	39.02303	2.578199	4.564471	10.96173
206.257855	4.426134	692.3795	14.85793	38.81993	2.074732	4.245123	10.4318
208.169353	4.429135	470.0615	10.00131	26.18673	3.447016	2.857517	5.572173
224.003629	4.218524	789.0815	14.86029	40.11446	3.719115	4.245798	10.64177
298.651768	3.452622	1025.468	11.85512	36.1543	3.928974	3.387177	8.402496
206.795261	4.390558	676.5306	14.36371	37.62892	2.689519	4.103916	9.973149
178.134338	5.193421	612.5234	17.85782	43.21675	2.249663	5.102235	12.6644
207.669506	4.34455	820.8533	17.17266	45.15383	2.57344	4.906475	12.82811
207.665842	4.514475	990.7231	21.53746	56.08981	3.152843	6.15356	17.02298
225.733077	4.141891	868.4497	15.93486	43.29595	2.144337	4.552816	11.79296
240.076031	3.948619	899.2972	14.79107	41.30243	2.74266	4.226021	10.84245
198.22611	4.653195	744.813	17.48387	44.66734	3.23928	4.995392	12.83068
172.307014	4.418129	570.6792	14.6328	36.56736	3.342795	4.1808	10.21467
208.073623	3.394349	652.3318	10.64163	29.77647	2.016414	3.040465	7.247278
204.008865	3.440284	806.0289	13.59239	37.71895	3.122481	3.883541	10.15211
195.802523	3.599311	782.3712	14.38182	39.05833	2.597243	4.109093	10.78251
195.028366	3.618337	911.9967	16.92016	45.84602	6.821084	4.834332	13.30182
182.293535	3.997665	628.9496	13.79275	35.842	3.095916	3.940787	9.795089
191.826874	3.539241	654.3128	12.07219	32.75563	2.780623	3.449198	8.532951
180.511968	3.932723	599.9762	13.07138	34.02315	5.346549	3.734679	9.138655
165.052354	4.210519	657.3383	16.76883	41.95893	3.01384	4.791095	12.55831
191.826527	3.506883	862.7333	15.77209	42.89296	3.31718	4.506311	12.26521
205.376183	3.383463	759.203	12.50746	34.91133	2.86501	3.573561	9.124
180.356581	3.870313	504.581	10.82792	28.29055	2.864918	3.093691	6.957605
162.088974	5.195159	625.9255	20.06171	47.41401	2.634439	5.731918	14.86655
199.163426	4.620961	978.9585	22.71365	58.19777	4.153007	6.489615	18.09269
210.734266	4.464709	727.8366	15.42027	40.41824	2.518724	4.40579	10.95556

236.047055	4.028107	660.0498	11.26365	31.16402	3.438396	3.218185	7.235542
222.796735	4.227642	679.0858	12.88588	34.71896	3.594422	3.68168	8.658237
198.725956	4.754209	813.7622	19.46799	49.50115	2.916018	5.562284	14.71378
196.02302	4.623184	620.977	14.64568	37.37241	3.741717	4.184481	10.0225
181.267054	5.049222	980.6875	27.3172	66.86669	5.259627	7.804915	22.26798
211.939411	4.38798	709.8599	14.69689	38.7447	3.411879	4.199112	10.30891
191.460831	4.762707	755.6636	18.7976	47.33245	3.03465	5.370744	14.0349
221.538652	4.252181	714.4982	13.71398	36.84452	3.343548	3.918279	9.461796
258.547896	3.774422	830.1312	12.1187	34.86413	3.050838	3.462487	8.344282
197.480448	4.624835	534.8465	12.52568	32.01904	2.708352	3.578766	7.900845
236.679678	4.004732	500.854	8.474687	23.49744	2.293565	2.421339	4.469954
181.555674	5.071388	629.2185	17.57594	42.99216	2.196093	5.021696	12.50455
218.705911	4.263273	889.4799	17.33879	46.40322	3.930994	4.95394	13.07552
217.193754	3.098342	699.0638	9.972379	28.85548	2.817462	2.849251	6.874038
214.315667	3.088122	705.4807	10.16543	29.34037	3.363587	2.904408	7.077306
212.93884	3.374625	653.8671	10.36239	29.20569	3.614454	2.960684	6.98777
177.789267	3.995264	656.7991	14.75953	38.12084	3.988107	4.217008	10.76426
207.815108	3.362704	774.9638	12.53987	35.15929	2.385819	3.582819	9.177163
192.380409	3.411	847.9505	15.03458	41.20133	2.608555	4.295595	11.62358
191.015172	3.673369	602.0699	11.57827	31.0917	3.151948	3.308076	7.904899
199.609807	3.603065	776.9603	14.02455	38.2619	4.799845	4.007015	10.42149
174.138411	4.216426	513.7656	12.43984	31.5357	2.732539	3.554241	8.223418
225.809834	2.829697	682.0602	8.54712	25.54586	2.124646	2.442034	5.717423
168.87331	4.31901	548.6856	14.03288	35.09061	2.027838	4.009394	9.713869
189.158861	3.908241	675.1557	13.9495	36.7934	2.980195	3.985571	10.04126
323.752638	3.348011	1138.949	11.77817	36.9347	3.505546	3.365193	8.430163
222.403987	4.188399	653.9692	12.3158	33.24577	2.319243	3.518801	8.127405
179.069905	5.044223	637.3813	17.9544	43.82562	4.560781	5.129829	12.91018
211.809078	4.394379	517.144	10.72913	28.27002	3.365813	3.065465	6.334749
191.639383	4.720182	754.5141	18.58409	46.9108	4.522082	5.30974	13.86391
241.557221	4.005924	867.0484	14.37891	40.06871	3.785909	4.10826	10.37299
198.271358	4.610962	685.8804	15.95071	40.84577	3.513276	4.557345	11.33975
224.081024	4.172831	690.6208	12.86072	34.8144	4.193985	3.674492	8.687892
259.154313	3.77776	883.6363	12.88099	37.07067	2.798935	3.680284	9.103235
244.924398	3.969601	782.6462	12.6847	35.55098	2.732093	3.624201	8.715102
207.505806	4.424431	740.9399	15.79829	41.34313	2.190264	4.513798	11.37386
232.781036	4.083878	770.5145	13.5178	37.14281	2.570397	3.862228	9.433921
216.5016	4.28716	652.4207	12.91922	34.43969	2.293509	3.691206	8.632062
179.827614	3.892373	499.8941	10.82022	28.20958	3.343023	3.091491	6.927847
187.055277	3.921494	630.1531	13.21076	34.71821	2.755899	3.774502	9.289262
174.371317	4.305465	702.6928	17.35044	43.76977	3.145069	4.957269	13.04498
187.570572	3.81241	650.7196	13.22601	35.02838	2.032006	3.77886	9.413598
267.664488	2.457892	829.6609	7.618557	24.61102	2.065293	2.176731	5.160665
204.099362	3.453458	681.8209	11.53673	31.98745	2.897259	3.296209	8.083274
184.574644	3.626221	813.5245	15.9828	42.69061	3.116034	4.566514	12.35658
191.455029	3.783696	749.7094	14.81639	39.51665	2.509735	4.233255	11.0327
215.154713	3.100212	583.0054	8.400653	24.24672	2.177986	2.400187	5.300441

194.832658	3.424124	953.9625	16.7656	46.04655	3.652901	4.790171	13.34147
235.420373	4.065982	956.6887	16.52312	45.57868	3.467026	4.720892	12.45714
260.129716	3.76454	630.7678	9.128333	26.31849	2.309174	2.608095	5.363793
214.926942	4.272901	688.8438	13.69471	36.47075	2.536352	3.912773	9.421806
266.344486	3.761928	1042.269	14.72132	42.70266	2.733038	4.206091	10.95939
203.63931	4.525318	730.197	16.2266	42.02722	3.585737	4.636172	11.70128
226.961985	4.141642	681.7017	12.43981	33.84613	2.165244	3.554232	8.29817
303.297821	3.454417	962.9601	10.96765	33.57279	3.102923	3.133616	7.513237
250.242381	3.903937	703.9529	10.9821	31.07421	2.141388	3.137744	7.078167
233.65109	4.084809	720.0271	12.58789	34.61799	3.950051	3.596539	8.503078
227.251827	4.109436	714.6864	12.9238	35.2429	3.144909	3.692515	8.814368
257.364764	3.824142	752.9508	11.18798	32.04463	1.801119	3.196565	7.363834
244.976809	3.919629	1003.59	16.05745	45.14883	4.398207	4.587842	12.13782
229.457886	4.141839	765.3354	13.81472	37.68942	3.135623	3.947063	9.67288
227.849222	2.848115	918.813	11.48516	34.34865	2.985869	3.281475	8.637047
222.407637	3.10192	595.1399	8.300417	24.15348	2.675897	2.371548	5.198497
189.14276	3.568731	601.9231	11.35704	30.6432	3.301347	3.244869	7.788309
208.521253	3.098384	555.0688	8.247679	23.62303	3.531032	2.35648	5.149295
207.207927	3.315327	606.5121	9.704194	27.28535	2.40216	2.772627	6.388867
208.174322	3.202682	706.3672	10.86719	30.85642	3.647996	3.104911	7.664506
239.031922	2.575775	658.7927	7.099059	22.03372	2.641286	2.028303	4.523284
190.79559	3.655088	802.0059	15.3641	41.29759	4.203482	4.389742	11.70901
182.939996	4.148299	599.2584	13.58863	35.01751	2.521572	3.882465	9.440327
236.569124	2.66107	706.2622	7.944457	24.39437	2.029601	2.269845	5.283387
200.797518	3.637636	578.6796	10.48333	28.57484	2.258847	2.995236	6.84569
210.565499	3.480421	728.4503	12.0405	33.58021	2.757776	3.440143	8.56008
215.822965	2.928398	710.1624	9.635854	28.233	2.132642	2.753101	6.707455
261.945997	3.769007	1050.645	15.1172	43.64833	4.261121	4.3192	11.34819
253.342951	3.867831	627.1618	9.57499	27.23947	2.475545	2.735711	5.707159
264.402063	3.734492	853.5555	12.05587	34.9709	3.037637	3.444534	8.321376
223.622762	4.211351	747.6301	14.07966	38.0072	3.349183	4.022761	9.868312
276.241953	3.629986	664.9212	8.737466	25.80665	2.052414	2.496419	5.10748
233.517786	4.082479	793.5424	13.87312	38.15251	3.068305	3.963748	9.790639
243.408091	3.919615	807.0514	12.996	36.48233	2.935045	3.713142	9.076382
252.502073	3.849117	751.5079	11.45591	32.60286	2.23447	3.273118	7.606795
286.368468	3.575137	627.6437	7.835752	23.44168	1.495261	2.238786	4.260615
274.25306	3.651838	723.7941	9.637738	28.37168	1.8936	2.753639	5.985899
277.883874	3.627727	781.9999	10.20888	30.20196	2.360024	2.916822	6.581149
308.466427	3.423601	818.2004	9.081026	27.97796	2.745656	2.594579	5.657425
218.629432	3.312567	862.4358	13.06721	37.24507	3.408067	3.733488	9.754642
214.912993	3.342348	802.6754	12.48329	35.34938	3.858092	3.566654	9.14094
191.287012	3.758095	613.4588	12.05224	32.19193	2.354289	3.443496	8.29414
204.795652	3.401921	880.9129	14.6331	40.76013	2.605435	4.180887	11.23118
195.401252	3.489308	648.688	11.58371	31.688	3.015253	3.309633	8.094407
189.868823	3.92291	1265.979	26.1566	68.99102	6.287196	7.473314	22.23369
204.047798	3.458437	664.8318	11.26833	31.23005	2.447395	3.219524	7.809897
223.733941	2.928455	876.7988	11.47642	33.92969	3.172406	3.278978	8.547969

246.681953	2.479216	662.7872	6.661178	21.03811	2.562052	1.903194	4.181962
214.49458	2.979091	899.232	12.48933	36.38081	3.205264	3.568381	9.510242
205.58244	3.364688	866.5007	14.18168	39.64952	4.857045	4.051909	10.81699
216.666175	3.200387	605.2244	8.939799	25.64336	3.622747	2.554228	5.739413
213.183683	3.346683	640.9127	10.06142	28.4246	2.758417	2.874693	6.714742
236.18927	4.051274	737.5964	12.65174	34.95967	4.331718	3.614783	8.600465
269.185611	3.707791	1571.601	21.64739	63.18875	5.015133	6.18497	17.9396
267.237775	3.737591	946.6766	13.24023	38.50101	2.910217	3.782924	9.502641
297.554943	3.476109	737.1727	8.611831	26.19473	2.56944	2.460523	5.135722
261.108089	3.756951	706.3496	10.1633	29.3448	2.815764	2.903801	6.406353
248.776042	3.881062	783.8993	12.22932	34.60324	3.306848	3.494091	8.348257
267.925779	3.685361	798.7488	10.98692	32.08185	3.005721	3.139119	7.301555
249.417576	3.878967	899.565	13.99012	39.61634	2.92188	3.997178	10.11116
289.829113	3.525902	1041.218	12.66688	38.14062	2.693165	3.61911	9.140983
319.101172	3.383894	876.7832	9.297807	28.974	2.943231	2.656516	5.913914
203.608288	3.399137	611.2563	10.20461	28.38921	3.002119	2.915604	6.805477
192.450503	3.97625	832.0388	17.19088	45.34293	3.629844	4.911681	13.21463
193.029299	3.755434	749.7856	14.58727	39.05843	3.88431	4.167791	10.83184
218.79502	3.161778	559.668	8.087689	23.32657	2.561634	2.310768	4.925911
217.424232	2.986597	604.0118	8.296865	24.23522	2.113405	2.370533	5.310269
185.260038	3.773117	543.3964	11.06714	29.29582	2.276412	3.162039	7.294019
210.396441	3.37715	453.0581	7.272201	20.43092	1.717561	2.077772	3.895051
221.581569	3.094715	629.4932	8.791805	25.57445	2.222127	2.511944	5.69709
202.922546	3.26242	642.4602	10.32894	29.00704	3.26563	2.951126	7.066522
197.156517	3.495683	557.9282	9.892343	27.10934	2.137605	2.826384	6.396661
196.254664	3.641088	469.8502	8.717072	23.61934	2.732893	2.490592	5.075983
302.331328	3.475073	681.148	7.829287	23.91124	1.974406	2.236939	4.354215
223.184765	4.203103	528.3639	9.950356	26.86039	2.449844	2.842959	5.747253
301.396982	3.488391	640.252	7.410324	22.59257	2.345203	2.117235	3.921933
243.600274	3.967431	778.5867	12.68056	35.4961	2.417915	3.623019	8.713134
240.317654	3.939634	691.6624	11.33873	31.68815	2.596049	3.239637	7.399095
267.006284	3.729138	1237.988	17.29033	50.29578	4.290314	4.940094	13.56119
244.900635	3.91841	829.2854	13.26857	37.30732	3.251713	3.791019	9.350156
227.587445	4.145491	652.2677	11.88101	32.34049	2.364608	3.394575	7.735523
268.822566	3.733647	893.6678	12.41205	36.1557	3.196199	3.546301	8.678406
259.159032	3.822405	551.5895	8.135539	23.34499	2.481837	2.32444	4.313134
232.760244	4.033973	545.402	9.452374	26.05163	1.95162	2.700678	5.418401
302.994348	3.427538	545.402	6.169706	18.9181	1.852307	1.762773	2.742168
262.253661	3.778871	582.4099	8.392074	24.22195	2.079351	2.397735	4.613202
220.033602	3.138853	1118.125	15.95042	46.15319	2.872778	4.557264	12.81157
195.261619	3.712198	356.1584	6.771072	18.23492	2.306438	1.934592	3.058874
205.981976	3.32229	711.1813	11.47067	32.18745	2.383501	3.277333	8.148377
202.80644	3.265804	567.6087	9.140237	25.65845	2.364346	2.611496	5.874433
197.903677	3.377196	613.4402	10.46826	28.96337	2.635055	2.990932	7.091067
187.301162	4.002162	440.1527	9.404973	24.59909	2.167356	2.687135	5.402811
194.910872	3.493026	854.1694	15.3077	41.83775	3.389087	4.373627	11.81467
196.318165	3.543649	578.6714	10.44533	28.49704	1.848904	2.984381	6.901683

222.18528	2.927342	430.8269	5.676243	16.75411	1.768225	1.621784	2.748901
212.020886	3.251854	822.5269	12.61544	35.84796	3.304962	3.604413	9.36359
187.452731	3.632805	292.0701	5.660273	15.1705	1.945971	1.617221	2.027469
150.597417	5.53667	129.9693	4.778283	10.91224	1.184062	1.365224	-0.75839
230.15741	3.718213	282.9728	4.571451	12.82262	1.260699	1.306129	0.853237
176.090452	4.347912	186.6221	4.607953	11.62443	1.226802	1.316558	0.260041
142.363537	5.834571	157.5402	6.456566	14.3499	1.410724	1.844733	0.621994
162.41131	5.205491	181.9134	5.830558	13.77999	1.195917	1.665874	0.625067
198.694809	4.48521	336.2669	7.590675	19.58309	1.197561	2.168764	3.105465
164.69611	4.692197	232.3238	6.618912	16.11066	1.009144	1.891118	1.926715
150.966681	5.117515	91.61415	3.105564	7.237624	1.060228	0.887304	-2.01195
237.204425	3.723774	212.8467	3.341392	9.43979	0.609314	0.954684	-0.38238
150.460866	6.06697	97.54823	3.933396	8.777698	0.787937	1.123828	-2.13357
178.170676	5.090591	223.0723	6.373494	15.50225	1.156444	1.820998	1.282904
173.05745	5.181361	146.8357	4.396278	10.56871	0.59529	1.25608	-0.78508
163.745761	3.781657	196.4489	4.536926	11.63814	0.995844	1.296265	0.755269
164.679815	4.499449	243.4632	6.652	16.36148	1.194472	1.900571	2.152551
190.174738	3.521754	312.8012	5.792615	15.70266	1.097675	1.655033	2.27086
203.867302	4.403181	318.5438	6.879996	17.94665	1.300947	1.965713	2.476814
175.806856	5.201386	149.2743	4.416399	10.64872	0.958337	1.261828	-0.78499
207.293358	4.410497	154.272	3.282383	8.594363	0.652267	0.937824	-1.12811
188.507889	4.7603	152.8568	3.86002	9.683077	0.870198	1.102863	-0.90028
161.280631	5.780668	157.8515	5.65776	13.00306	1.241186	1.616503	-0.12291
224.457172	4.11848	270.8823	4.970317	13.50465	1.465021	1.420091	0.851837
188.35389	3.418401	239.6931	4.350147	11.85201	0.995602	1.242899	0.931746
153.96966	4.950793	144.4375	4.644293	10.96755	0.943809	1.326941	-0.3065
172.911072	4.039978	232.2651	5.426755	13.88037	0.997241	1.550501	1.386776
191.738792	4.665177	235.9719	5.741409	14.53714	1.040254	1.640403	1.076231
257.54576	3.737965	128.4993	1.865012	5.373241	0.532193	0.53286	-1.87295
219.263581	4.184419	270.54	5.162977	13.89098	0.925722	1.475136	0.978558
176.551726	4.193628	289.6581	6.88024	17.52565	1.143907	1.965783	2.686612
158.022134	4.90752	193.9553	6.023457	14.34861	1.400688	1.720988	1.115937
182.460512	3.898729	167.4778	3.578585	9.359936	0.688426	1.022453	-0.32014
189.634528	3.703799	159.849	3.122051	8.351365	0.780777	0.892015	-0.58175
173.105739	4.191422	250.1349	6.056535	15.35365	2.114074	1.730439	1.865113
191.17221	4.743727	168.0029	4.168806	10.5036	0.995756	1.191088	-0.57492
187.198616	4.824707	248.5257	6.405302	15.98628	1.130151	1.830086	1.580595
275.859337	3.591918	259.2714	3.37593	9.99387	0.807059	0.964551	-0.21599
224.527932	4.204643	193.0692	3.615528	9.773656	0.740878	1.033008	-0.58911
276.388359	3.62239	208.1398	2.727913	8.062352	0.736644	0.779404	-0.89448
125.865228	6.521514	118.5121	6.140523	12.87048	0.91811	1.754435	-0.38099
119.464744	7.027338	93.33248	5.490146	11.14798	1.156255	1.568613	-1.53719
121.539859	6.264941	73.1265	3.769407	7.910856	0.875539	1.076974	-2.49553
151.984834	5.408713	141.3457	5.030094	11.58118	0.833017	1.43717	-0.37862
126.69156	6.240964	175.7554	8.6579	18.37751	1.228209	2.473686	2.416937
128.070344	6.041054	134.948	6.365473	13.65887	0.943552	1.818707	0.324419
145.288803	5.188886	164.7048	5.882313	13.53124	1.104421	1.680661	0.693427

144.574422	5.200519	126.1429	4.537513	10.41907	1.127844	1.296432	-0.66301
148.286087	5.553786	107.3248	4.019654	9.137272	0.755831	1.148473	-1.53413
119.150431	7.134756	98.91087	5.922807	11.9731	1.561976	1.69223	-1.21195
171.544364	4.887304	140.4177	4.000505	9.737369	0.812979	1.143002	-0.8868
147.968635	5.562731	89.9271	3.380718	7.677669	0.632907	0.965919	-2.18201
131.065065	6.362382	79.80263	3.873914	8.253094	0.698788	1.106833	-2.48847
130.773612	5.864288	176.7686	7.926844	17.22569	1.19479	2.264812	2.062556
132.78227	5.823784	180.4832	7.915928	17.29759	1.245	2.261694	2.092145
140.556792	5.512031	133.9418	5.252619	11.8035	0.961839	1.500748	-0.25941
145.997016	5.27065	121.0091	4.368558	10.02208	0.855741	1.248159	-0.90209
128.856368	6.078131	132.2814	6.239687	13.38896	1.069243	1.782768	0.161556
137.528675	5.706584	130.0667	5.396958	11.95786	0.808395	1.541988	-0.30963
139.746463	5.333834	97.34635	3.71551	8.406084	1.00378	1.061574	-1.61832
134.824879	5.811417	115.8682	4.994318	10.96095	1.07312	1.426948	-0.8171
169.384941	5.041218	211.4783	6.293998	15.15345	1.053627	1.798285	1.25278
137.93758	5.945585	121.4439	5.23465	11.4884	1.029214	1.495614	-0.71093
155.994988	5.324061	170.814	5.829829	13.56353	0.971077	1.665665	0.505768
152.429313	5.424531	152.483	5.426441	12.49372	1.000219	1.550412	0.001911
157.785369	5.294811	175.5638	5.891401	13.76489	1.084523	1.683257	0.59659
137.953042	6.02415	215.1513	9.395254	20.55263	1.192077	2.684358	3.371103
145.441344	5.659196	199.9816	7.781386	17.52025	1.074958	2.223253	2.122189
139.713998	5.920085	204.1276	8.649473	19.06416	0.955238	2.471278	2.729388
147.918827	5.581843	178.3271	6.729325	15.26803	0.892767	1.922664	1.147482
192.552035	4.573683	207.897	4.938172	12.57873	0.80035	1.410906	0.364489
161.222002	5.20071	145.6016	4.696827	11.08269	0.59094	1.341951	-0.50388
162.081535	5.194921	174.5605	5.594887	13.223	1.007284	1.598539	0.399966
169.512484	4.985661	131.6139	3.870997	9.347437	0.73876	1.105999	-1.11466
139.637602	5.607936	237.6252	9.543179	21.31783	1.248548	2.726623	3.935243
146.076673	5.350794	154.4686	5.658192	12.93357	1.198028	1.616626	0.307398
135.183677	5.77708	147.9592	6.323044	13.90689	0.695664	1.806584	0.545964
164.673652	4.691557	168.4646	4.79956	11.6823	0.881804	1.371303	0.108003
145.256171	5.262905	163.3591	5.918807	13.5663	0.912795	1.691088	0.655903
147.825748	5.223525	165.0033	5.830507	13.44785	0.877119	1.665859	0.606982
161.486749	4.708068	240.1961	7.002803	16.9471	1.101376	2.000801	2.294734
154.017255	4.984377	155.6002	5.035604	11.87247	0.899492	1.438744	0.051227
165.706713	4.720989	238.0322	6.781544	16.50651	1.05625	1.937584	2.060555
135.306404	5.709131	195.9928	8.269738	18.24647	1.037724	2.362782	2.560607
144.785611	5.323	188.5812	6.933134	15.83331	0.901183	1.980895	1.610134
171.538895	4.550103	279.3695	7.410333	18.36213	0.998548	2.117238	2.860229
149.093824	5.213071	122.7102	4.290567	9.92216	0.677947	1.225876	-0.9225
174.554991	4.43033	191.9566	4.871994	12.20621	0.946274	1.391998	0.441664
154.27552	4.992735	156.8103	5.074766	11.9648	0.911452	1.449933	0.08203
135.14999	5.775641	66.19514	2.828852	6.221774	0.738227	0.808243	-2.94679
142.396225	5.414305	71.20278	2.70733	6.130983	0.716006	0.773523	-2.70698
168.882124	4.69117	162.4078	4.511327	11.05045	0.767395	1.288951	-0.17984
145.929642	5.268218	125.6033	4.534416	10.40259	1.116755	1.295548	-0.7338
210.591732	4.289037	191.3983	3.898132	10.31875	0.908859	1.113752	-0.39091

154.191393	5.410224	168.0376	5.896056	13.623	0.921101	1.684587	0.485832
151.315443	5.625109	153.3254	5.699827	12.98075	1.013283	1.628522	0.074718
147.131734	5.637231	150.3337	5.759913	13.01895	0.957915	1.645689	0.122682
153.561269	5.426193	182.3394	6.443089	14.86075	0.881149	1.840883	1.016896
150.153313	5.540713	159.0675	5.869648	13.39228	0.974049	1.677042	0.328935
181.968965	4.70204	150.3079	3.883925	9.687208	0.921624	1.109693	-0.81812
211.954596	4.33445	315.1057	6.44388	17.0402	0.844922	1.841108	2.10943
170.744057	4.96349	213.6197	6.209874	15.03912	0.830368	1.77425	1.246384
171.733381	4.992249	257.5919	7.488137	18.13483	1.139616	2.139468	2.495887
146.744232	5.600925	195.6382	7.467107	16.89381	1.049487	2.133459	1.866182
155.896496	5.413073	141.8483	4.925289	11.40985	0.689933	1.407226	-0.48778
151.179766	5.107425	229.9738	7.769384	18.12214	1.521194	2.219824	2.661959
147.467477	5.285573	229.9922	8.243449	18.94567	1.311997	2.355271	2.957876
145.098966	5.257209	150.3555	5.447665	12.48641	0.903687	1.556476	0.190456
177.364331	4.594931	187.9605	4.869444	12.13741	0.723063	1.39127	0.274513
171.057897	4.623186	178.4568	4.823157	11.89548	0.949944	1.378045	0.199971
146.491352	5.307658	115.7503	4.193851	9.612587	0.947407	1.198243	-1.11381
157.034933	4.907342	187.9128	5.872276	13.9667	1.156925	1.677793	0.964934
148.655633	5.179639	151.5857	5.281733	12.22494	1.004438	1.509066	0.102094
166.840043	4.712996	170.9062	4.827858	11.7762	0.981654	1.379388	0.114863
154.838284	5.093365	245.2399	8.067102	18.94244	0.905458	2.304886	2.973737
163.158012	4.661657	227.9358	6.51245	15.84023	0.894919	1.8607	1.850793
151.270653	5.180502	199.3396	6.8267	15.86925	1.094075	1.950486	1.646198
142.575887	5.421136	198.7598	7.557407	17.1144	1.07105	2.159259	2.13627
156.486035	4.967811	197.5744	6.272204	14.85928	1.087548	1.792058	1.304394
210.952432	4.349535	333.4586	6.875435	18.14411	1.674239	1.96441	2.525901
169.770944	5.007992	278.2345	8.207507	19.80438	1.227419	2.345002	3.199514
154.706813	5.390481	282.0907	9.828945	22.74979	2.978682	2.80827	4.438464
177.131702	4.879661	161.1296	4.438832	10.89545	0.886612	1.268238	-0.44083
147.976554	5.605172	161.9093	6.132929	13.90173	1.108058	1.752265	0.527756
192.781069	4.568272	204.9915	4.857618	12.38088	0.993896	1.387891	0.289346
161.567989	5.297311	161.2165	5.285787	12.4218	0.707462	1.510225	-0.01152
165.859657	5.010866	178.4078	5.389963	12.92834	0.893528	1.539989	0.379097
154.735489	5.39148	164.6025	5.735278	13.27471	0.825344	1.638651	0.343798
157.242023	5.348368	148.9498	5.06632	11.79721	1.07322	1.44752	-0.28205
174.367458	4.911759	212.844	5.995605	14.63491	0.952497	1.71303	1.083845
162.082284	5.228461	263.9117	8.513281	20.08803	1.055	2.432366	3.28482
164.415637	5.137989	257.1595	8.036234	19.11349	1.116547	2.296067	2.898245
173.795061	4.93736	278.9459	7.924599	19.30247	1.113332	2.264171	2.987239
210.908668	4.330773	277.178	5.69154	15.0353	0.898332	1.626154	1.360767
201.559045	4.459271	188.4801	4.169914	10.81214	0.686514	1.191404	-0.28936
160.199906	4.914108	166.9513	5.121205	12.23705	1.024192	1.463202	0.207098
225.246	3.798415	390.7767	6.589826	18.2868	1.449818	1.882808	2.791411
181.162232	4.323681	278.5435	6.647817	16.91346	1.81419	1.899376	2.324136
163.003444	4.794219	252.2728	7.419788	17.91683	0.977226	2.11994	2.62557
184.226408	4.344962	268.3948	6.330067	16.15287	1.413513	1.80859	1.985104
186.762035	4.323195	231.3626	5.355616	13.73031	1.516741	1.530176	1.032421

170.226957	4.600729	127.286	3.440162	8.484559	0.662124	0.982903	-1.16057
182.690947	4.42351	202.1624	4.894974	12.40903	1.225867	1.398564	0.471464
194.794557	4.234664	248.5871	5.404067	14.07376	0.90596	1.544019	1.169402
162.779921	4.844641	179.4194	5.339863	12.85627	0.907504	1.525675	0.495222
181.758156	4.369186	229.4307	5.515162	14.00656	1.097826	1.575761	1.145976
176.498913	4.537247	193.1793	4.96605	12.40219	0.873444	1.418871	0.428802
194.613812	4.589948	475.164	11.2067	28.59691	1.930856	3.201914	6.616751
228.95035	4.009638	270.5412	4.738025	13.02436	1.163666	1.353721	0.728387
180.210758	4.978198	145.9725	4.032391	9.890989	0.799921	1.152112	-0.94581
183.812396	4.91477	214.6027	5.73804	14.18997	1.186851	1.63944	0.82327
190.451533	4.7376	187.5312	4.664956	11.74638	0.987379	1.332844	-0.07264
171.036411	5.31169	243.7345	7.569393	18.03122	0.992651	2.162684	2.257703
168.370868	5.448895	219.2875	7.096684	16.73189	1.166903	2.027624	1.647788
221.117949	4.18784	169.6568	3.213198	8.661556	0.999082	0.918056	-0.97464
193.190279	4.643997	262.5508	6.311318	16.02852	1.001965	1.803234	1.667321
201.867056	4.546555	203.4908	4.583126	11.83062	1.017357	1.309464	0.03657
187.992497	4.82032	234.2123	6.005442	15.0076	1.01455	1.715841	1.185122
174.769604	5.125208	242.7615	7.119105	17.20339	0.874883	2.03403	1.993897
209.450671	4.354484	260.8718	5.42353	14.28296	1.168081	1.54958	1.069047
198.072014	4.574411	253.4193	5.852639	15.01322	1.27943	1.672183	1.278228
162.512731	4.724207	200.5758	5.830691	14.12081	0.872499	1.665912	1.106484
168.266477	4.28159	222.2948	5.656357	14.16234	0.972496	1.616102	1.374767
184.885764	3.773179	247.1057	5.042974	13.34245	1.242351	1.44085	1.269795
172.111653	4.147269	243.1089	5.858046	14.86842	1.059114	1.673727	1.710777
190.43013	3.283278	291.6689	5.028774	13.87775	1.058246	1.436793	1.745496
163.673793	4.623553	226.5144	6.398712	15.60786	1.096289	1.828203	1.775158
179.058315	4.62683	143.0699	3.696896	9.220722	0.835149	1.056256	-0.92993
162.93614	4.276539	222.821	5.848321	14.52989	1.224244	1.670949	1.571782
169.633448	4.117317	212.4888	5.157494	13.06661	0.908184	1.47357	1.040178
181.33873	4.02975	292.7368	6.505262	16.84876	1.02531	1.858646	2.475513
223.316222	2.687319	382.9617	4.608444	13.91408	1.06178	1.316698	1.921124
169.72117	3.771582	337.8833	7.508518	19.44721	1.144136	2.145291	3.736936
174.537252	4.040214	247.3166	5.72492	14.6771	1.090188	1.635692	1.684706
178.495634	5.013922	205.735	5.779074	14.1163	0.932645	1.651164	0.765152
206.257855	4.426134	324.8217	6.970422	18.21191	0.973336	1.991549	2.544288
208.169353	4.429135	201.3851	4.284789	11.21899	1.47678	1.224225	-0.14435
224.003629	4.218524	243.4988	4.585664	12.37872	1.147663	1.31019	0.36714
208.37586	4.424116	240.4708	5.105536	13.37509	0.892128	1.458725	0.68142
298.651768	3.452622	251.4511	2.906949	8.865259	0.963409	0.830557	-0.54567
206.795261	4.390558	232.5581	4.93754	12.93498	0.924525	1.410726	0.546982
178.134338	5.193421	321.6024	9.376162	22.69074	1.181174	2.678904	4.182742
207.669506	4.34455	337.095	7.052196	18.54306	1.056819	2.014913	2.707645
207.665842	4.514475	328.0562	7.131657	18.57291	1.043995	2.037616	2.617182
225.733077	4.141891	355.4948	6.522841	17.72295	0.877772	1.863669	2.38095
240.076031	3.948619	356.6059	5.865228	16.378	1.08757	1.67578	1.91661
198.22611	4.653195	271.7295	6.378626	16.29594	1.181784	1.822465	1.725431
172.307014	4.418129	167.3732	4.291622	10.72476	0.980401	1.226178	-0.12651

208.073623	3.394349	361.7267	5.900926	16.51145	1.118129	1.685979	2.506576
195.802523	3.599311	243.6631	4.479101	12.1644	0.80889	1.279743	0.87979
195.028366	3.618337	166.5908	3.090739	8.374512	1.24598	0.883068	-0.5276
182.293535	3.997665	192.7441	4.226844	10.98392	0.948756	1.20767	0.229178
191.826874	3.539241	240.1109	4.43009	12.02022	1.020396	1.26574	0.890849
180.511968	3.932723	177.1322	3.85909	10.04472	1.578473	1.102597	-0.07363
165.052354	4.210519	163.4587	4.169865	10.43383	0.749444	1.19139	-0.04065
191.826527	3.506883	251.0131	4.588904	12.47975	0.965137	1.311116	1.082021
205.376183	3.383463	322.1555	5.30734	14.81406	1.215721	1.516383	1.923877
180.356581	3.870313	174.8883	3.752968	9.805536	0.992984	1.072277	-0.11734
162.088974	5.195159	276.7528	8.870282	20.96409	1.164816	2.534366	3.675122
199.163426	4.620961	191.7327	4.448555	11.39825	0.813382	1.271016	-0.17241
227.956431	4.122178	311.556	5.633925	15.36358	1.016951	1.609693	1.511747
210.734266	4.464709	369.1061	7.820045	20.49721	1.277315	2.234298	3.355336
236.047055	4.028107	208.2219	3.553275	9.831125	1.08469	1.015221	-0.47483
198.725956	4.754209	278.8147	6.670209	16.9603	0.999099	1.905774	1.916
196.02302	4.623184	200.2727	4.723414	12.05306	1.20675	1.349547	0.100229
181.267054	5.049222	144.3461	4.020783	9.84202	0.774158	1.148795	-1.02844
211.939411	4.38798	179.1215	3.70852	9.776589	0.860932	1.059577	-0.67946
191.460831	4.762707	225.4912	5.609234	14.12408	0.905544	1.602638	0.846527
221.538652	4.252181	269.125	5.165547	13.87796	1.259391	1.475871	0.913366
258.547896	3.774422	335.6514	4.900021	14.0968	1.233562	1.400006	1.125599
197.480448	4.624835	132.4658	3.102244	7.930177	0.670779	0.886355	-1.52259
236.679678	4.004732	235.9424	3.992258	11.06918	1.080453	1.140645	-0.01247
181.555674	5.071388	235.6959	6.583685	16.10422	0.822624	1.881053	1.512298
218.705911	4.263273	237.5463	4.630532	12.39254	1.049819	1.323009	0.367259
217.193754	3.098342	213.5291	3.046064	8.813907	0.860594	0.870304	-0.05228
214.315667	3.088122	215.4838	3.104954	8.961797	1.027383	0.88713	0.016832
212.93884	3.374625	215.2993	3.412033	9.616578	1.190134	0.974867	0.037408
177.789267	3.995264	220.7658	4.96103	12.81333	1.340498	1.417437	0.965766
207.815108	3.362704	304.6375	4.92941	13.82108	0.937863	1.408403	1.566706
192.380409	3.411	306.3266	5.431322	14.8842	0.942354	1.551806	2.020322
199.609807	3.603065	203.1479	3.66693	10.00415	1.254992	1.047694	0.063865
174.138411	4.216426	208.6989	5.053242	12.81025	1.109996	1.443783	0.836815
225.809834	2.829697	518.4459	6.496816	19.41786	1.614981	1.856233	3.667119
168.87331	4.31901	229.9316	5.880604	14.70504	0.849784	1.680173	1.561594
189.158861	3.908241	273.0777	5.6421	14.88169	1.205388	1.612029	1.73386
179.069905	5.044223	135.8846	3.827735	9.343272	0.972322	1.093639	-1.21649
211.809078	4.394379	190.7989	3.958483	10.43015	1.241808	1.130995	-0.4359
191.639383	4.720182	191.76	4.723153	11.9224	1.149289	1.349472	0.002971
241.557221	4.005924	181.1938	3.004872	8.373467	0.79117	0.858535	-1.00105
198.271358	4.610962	225.6124	5.2468	13.43574	1.155652	1.499086	0.635838
224.081024	4.172831	235.6777	4.388783	11.88058	1.431217	1.253938	0.215952
259.154313	3.77776	307.4561	4.481867	12.89852	0.973873	1.280533	0.704107
244.924398	3.969601	325.3883	5.273716	14.78046	1.135878	1.506776	1.304115
207.505806	4.424431	322.8736	6.884298	18.01577	0.954434	1.966942	2.459867
232.781036	4.083878	266.553	4.676368	12.84924	0.889207	1.336105	0.59249

216.5016	4.28716	290.7459	5.757345	15.34776	1.022083	1.644956	1.470185
293.345666	3.504727	263.2353	3.144985	9.512616	0.976005	0.898567	-0.35974
198.578523	3.718699	221.7612	4.152831	11.22612	1.350229	1.186523	0.434132
199.532981	3.661156	236.6963	4.343052	11.80033	1.18337	1.240872	0.681897
179.827614	3.892373	184.3976	3.99129	10.40576	1.233152	1.140369	0.098917
187.055277	3.921494	224.8594	4.714035	12.3886	0.983396	1.346867	0.79254
174.371317	4.305465	233.9054	5.775441	14.56964	1.046899	1.650126	1.469976
187.570572	3.81241	344.1575	6.995071	18.52607	1.074702	1.998592	3.182661
267.664488	2.457892	360.5615	3.310941	10.69568	0.897554	0.945983	0.853049
204.099362	3.453458	254.0001	4.297803	11.91635	1.079322	1.227944	0.844345
184.574644	3.626221	318.9987	6.267166	16.73981	1.221857	1.790619	2.640945
191.455029	3.783696	310.2546	6.131514	16.3533	1.038612	1.751861	2.347818
215.154713	3.100212	260.1789	3.748975	10.82063	0.971974	1.071136	0.648763
194.832658	3.424124	287.8202	5.058351	13.89271	1.102117	1.445243	1.634227
248.889081	3.919513	311.8805	4.911504	13.86462	1.301655	1.403287	0.991991
235.420373	4.065982	263.1977	4.545729	12.5393	0.953825	1.29878	0.479747
260.129716	3.76454	266.7407	3.860212	11.12963	0.976509	1.102918	0.095672
214.926942	4.272901	268.815	5.344235	14.23238	0.989788	1.526924	1.071334
266.344486	3.761928	358.0543	5.057264	14.66979	0.93889	1.444933	1.295336
226.961985	4.141642	283.5467	5.17421	14.07794	0.900611	1.478346	1.032568
303.297821	3.454417	240.9781	2.744625	8.401496	0.776498	0.784179	-0.70979
250.242381	3.903937	329.2518	5.136533	14.53398	1.001567	1.467581	1.232596
233.65109	4.084809	319.1019	5.578705	15.34202	1.750585	1.593916	1.493896
227.251827	4.109436	367.2712	6.641433	18.11103	1.616142	1.897552	2.531997
257.364764	3.824142	344.045	5.112111	14.64212	0.822984	1.460603	1.287968
244.976809	3.919629	247.5768	3.961228	11.13781	1.084998	1.131779	0.041599
229.457886	4.141839	279.8001	5.050542	13.77893	1.146357	1.443012	0.908703
227.849222	2.848115	278.7435	3.484294	10.42047	0.905834	0.995513	0.636179
222.407637	3.10192	186.2671	2.597867	7.559564	0.837503	0.742248	-0.50405
208.521253	3.098384	147.3085	2.188833	6.269262	0.937093	0.625381	-0.90955
207.207927	3.315327	245.0057	3.920091	11.02215	0.970373	1.120026	0.604765
208.174322	3.202682	234.262	3.60403	10.23332	1.209834	1.029723	0.401348
239.031922	2.575775	243.6722	2.625778	8.149762	0.976951	0.750222	0.050003
182.939996	4.148299	251.7212	5.707963	14.70926	1.059198	1.630847	1.559664
236.569124	2.66107	370.9892	4.173107	12.81401	1.06612	1.192316	1.512037
200.797518	3.637636	247.3506	4.480989	12.21402	0.965521	1.280283	0.843353
210.565499	3.480421	312.9043	5.171973	14.42431	1.184597	1.477706	1.691551
215.822965	2.928398	299.5048	4.063838	11.90702	0.899423	1.161096	1.135439
261.945997	3.769007	281.2193	4.04632	11.68306	1.140546	1.156092	0.277313
253.342951	3.867831	300.1956	4.583138	13.03837	1.184938	1.309468	0.715307
264.402063	3.734492	317.0316	4.477847	12.98906	1.128253	1.279385	0.743355
223.622762	4.211351	239.0219	4.501354	12.15113	1.070754	1.286101	0.290002
276.241953	3.629986	352.4892	4.631921	13.68067	1.08803	1.323406	1.001935
233.517786	4.082479	258.1157	4.512511	12.40987	0.998028	1.289289	0.430033
243.408091	3.919615	280.39	4.515137	12.67488	1.019709	1.290039	0.595522
252.502073	3.849117	375.8458	5.729356	16.30541	1.117508	1.636959	1.880239
286.368468	3.575137	399.2469	4.984355	14.91136	0.951142	1.424102	1.409219

274.25306	3.651838	349.5573	4.654558	13.70214	0.914517	1.329874	1.00272
277.883874	3.627727	357.2499	4.663836	13.7975	1.078156	1.332525	1.036109
308.466427	3.423601	359.4246	3.989174	12.29035	1.20613	1.139764	0.565573
214.912993	3.342348	164.4297	2.557227	7.241391	0.790338	0.730636	-0.78512
191.287012	3.758095	242.959	4.77326	12.74954	0.932411	1.363789	1.015166
204.795652	3.401921	276.091	4.58623	12.77482	0.816582	1.310351	1.184308
195.401252	3.489308	195.2461	3.486538	9.53765	0.907549	0.996154	-0.00277
189.868823	3.92291	228.6647	4.724478	12.46135	1.135611	1.349851	0.801569
204.047798	3.458437	278.2163	4.71553	13.06904	1.024177	1.347294	1.257093
223.733941	2.928455	281.5039	3.684606	10.89342	1.018529	1.052745	0.756151
246.681953	2.479216	229.6671	2.308212	7.290066	0.887795	0.659489	-0.171
214.49458	2.979091	277.329	3.851792	11.22008	0.988524	1.100512	0.872701
205.58244	3.364688	176.4399	2.887723	8.073573	0.989008	0.825064	-0.47697
216.666175	3.200387	222.3235	3.283951	9.419847	1.330782	0.938272	0.083564
213.183683	3.346683	244.6781	3.8411	10.85152	1.053067	1.097457	0.494417
236.18927	4.051274	302.8609	5.19487	14.35462	1.778626	1.484248	1.143596
269.185611	3.707791	328.97	4.531267	13.22677	1.049776	1.294648	0.823477
267.237775	3.737591	287.6246	4.022722	11.69759	0.884199	1.149349	0.285131
297.554943	3.476109	322.6494	3.769269	11.46504	1.124605	1.076934	0.29316
261.108089	3.756951	279.9866	4.028584	11.63184	1.116127	1.151024	0.271633
248.776042	3.881062	347.8673	5.426947	15.35572	1.467465	1.550556	1.545885
267.925779	3.685361	297.0262	4.085642	11.9301	1.117721	1.167326	0.400281
249.417576	3.878967	343.2795	5.338717	15.11784	1.115007	1.525348	1.45975
289.829113	3.525902	427.5201	5.200974	15.66039	1.105803	1.485993	1.675073
319.101172	3.383894	305.3507	3.238078	10.09056	1.025017	0.925165	-0.14582
242.642165	3.938996	223.3304	3.625493	10.15693	0.981816	1.035855	-0.3135
203.608288	3.399137	210.4781	3.513825	9.775453	1.033741	1.00395	0.114689
192.450503	3.97625	226.603	4.681881	12.349	0.988576	1.33768	0.705631
218.79502	3.161778	216.3037	3.125776	9.015388	0.990035	0.893079	-0.036
217.424232	2.986597	260.6555	3.580432	10.45847	0.91202	1.022981	0.593836
185.260038	3.773117	242.9973	4.949029	13.10057	1.017971	1.414008	1.175912
210.396441	3.37715	273.944	4.397175	12.35366	1.038532	1.256336	1.020025
221.581569	3.094715	268.7319	3.753239	10.91778	0.948631	1.072354	0.658525
202.922546	3.26242	223.0833	3.586548	10.0722	1.133934	1.024728	0.324127
197.156517	3.495683	250.21	4.436347	12.15753	0.958636	1.267528	0.940665
196.254664	3.641088	159.5128	2.95942	8.018698	0.927809	0.845549	-0.68167
302.331328	3.475073	339.2487	3.899411	11.9091	0.983362	1.114117	0.424338
223.184765	4.203103	322.518	6.073786	16.39582	1.495406	1.735367	1.870683
301.396982	3.488391	303.6052	3.513949	10.71332	1.112087	1.003985	0.025558
243.600274	3.967431	318.7604	5.191537	14.53242	0.989916	1.483296	1.224106
240.317654	3.939634	199.3025	3.267254	9.130938	0.748051	0.933501	-0.67238
267.006284	3.729138	286.1949	3.997135	11.62725	0.991824	1.142039	0.267997
244.900635	3.91841	243.1277	3.890043	10.93766	0.953329	1.111441	-0.02837
227.587445	4.145491	269.4168	4.907409	13.35812	0.976692	1.402117	0.761918
268.822566	3.733647	272.8668	3.789817	11.03955	0.975907	1.082805	0.05617
259.159032	3.822405	244.8897	3.611942	10.3645	1.101864	1.031984	-0.21046
232.760244	4.033973	291.5597	5.053027	13.92662	1.043292	1.443722	1.019054

302.994348	3.427538	279.4375	3.161057	9.692715	0.949032	0.903159	-0.26648	
262.253661	3.778871	416.3269	5.998947	17.3147	1.486392	1.713985	2.220076	
220.033602	3.138853	366.5545	5.229023	15.13039	0.941782	1.494006	2.09017	
217.541379	3.046798	237.9039	3.331987	9.68563	1.166091	0.951996	0.285189	
195.261619	3.712198	151.8625	2.88712	7.775194	0.983443	0.824891	-0.82508	
205.981976	3.32229	299.1755	4.825412	13.54043	1.002677	1.378689	1.503122	
202.80644	3.265804	253.0148	4.074312	11.4374	1.05392	1.164089	0.808508	
197.903677	3.377196	234.6441	4.004166	11.07864	1.007923	1.144047	0.62697	
187.301162	4.002162	172.8036	3.692384	9.657579	0.850902	1.054967	-0.30978	
194.910872	3.493026	264.1063	4.733087	12.93609	1.047894	1.352311	1.240061	
196.318165	3.543649	308.509	5.568755	15.19272	0.985712	1.591073	2.025106	
222.18528	2.927342	195.3757	2.57412	7.597825	0.801873	0.735463	-0.35322	
212.020886	3.251854	479.5687	7.355348	20.90091	1.926936	2.101528	4.103494	
187.452731	3.632805	176.2055	3.414835	9.15234	1.174002	0.975667	-0.21797	
209.076869	3.144013	226.8818	3.411756	9.742792	0.844625	0.974788	0.267743	
204.58662	3.206687	338.3748	5.303681	14.98934	1.270526	1.515337	2.096994	
134.625146	6.631781	385.917	19.01069	40.35263	2.866604	5.431626	12.37891	2.5
148.985616	5.935682	465.5	18.54582	41.51111	3.124463	5.298805	12.61013	2.5
132.886634	6.644332	581.944	29.0972	61.53309	4.379252	8.313486	22.45287	2.5
125.179625	7.235816	306.1	17.69364	36.08517	2.445286	5.055326	10.45783	2.5
150.309685	5.871472	435.875	17.02637	38.29852	2.899846	4.864676	11.1549	2.5
227.002264	4.291158	882	16.67297	44.96523	3.885424	4.763705	12.38181	2.5
262.715185	3.962522	793.154	11.96311	34.13677	3.019064	3.418031	8.000585	2.6
172.595718	5.183055	460.5	13.82883	33.21976	2.668085	3.951094	8.645774	2.5
175.604041	5.149679	544.444	15.9661	38.58224	3.100407	4.561743	10.81642	2.5
219.003595	4.38885	692.984	13.88745	36.91032	3.164259	3.967844	9.498605	2.5
167.82469	5.344735	525.749	16.7436	39.63522	3.132727	4.783885	11.39886	2.5
136.129308	6.576295	321.242	15.51894	33.10202	2.35983	4.433982	8.942642	2.5
138.76165	6.307348	393.5	17.88636	38.73716	2.835798	5.11039	11.57902	2.5
189.118303	4.799957	540.778	13.72533	34.3872	2.859469	3.921523	8.925373	2.5
138.288369	6.402239	349.214	16.16731	34.8539	2.525259	4.619233	9.765075	2.5
164.178789	5.472626	465.154	15.50513	36.2874	2.833216	4.430038	10.03251	2.5
163.890945	5.426852	470.222	15.57026	36.50041	2.869115	4.448647	10.14341	2.5
176.370509	5.039157	592.8	16.93714	41.1962	3.361106	4.839184	11.89799	2.5
170.284573	5.038005	732.6	21.67456	52.2612	4.30221	6.19273	16.63655	2.5
156.007661	5.551874	513.273	18.26594	42.0551	3.29005	5.218841	12.71407	2.5
185.98109	4.855903	638.083	16.66013	41.44557	3.430903	4.760037	11.80423	2.5
227.168089	4.262065	830.071	15.57356	42.07943	3.653995	4.44959	11.3115	2.5
223.813848	4.287622	791.167	15.15646	40.73946	3.534933	4.330416	10.86883	2.5
184.419618	4.917856	572.045	15.25453	37.74911	3.101866	4.358438	10.33668	2.5
177.203212	5.048525	549.896	15.66655	38.13293	3.103194	4.476158	10.61803	2.5
297.821356	3.798742	1123.076	14.32495	42.62576	3.770972	4.092843	10.52621	2.5
178.795511	5.079418	560.615	15.92656	38.79339	3.135509	4.550446	10.84714	2.5
153.514327	5.117144	532.615	17.75383	41.55014	3.469481	5.072524	12.63669	2.5
112.790795	7.138658	430.833	27.26791	54.36459	3.819753	7.790832	20.12925	2.5
157.314865	4.870429	489.25	15.14706	36.11012	3.110005	4.327731	10.27663	2.5
140.364054	5.357407	429.778	16.40374	37.11233	3.061881	4.686783	11.04633	2.5

182.51803	4.244605	629.727	14.64481	37.5017	3.450218	4.184233	10.40021	2.5
226.836815	3.737015	865.636	14.26089	39.80556	3.816118	4.07454	10.52387	2.5
220.870941	3.827919	827.333	14.33853	39.51832	3.745776	4.096722	10.51061	2.5
167.052601	4.679345	541.9	15.17927	37.10377	3.243888	4.336935	10.49993	2.5
157.636548	4.663803	453.143	13.4066	32.32569	2.874606	3.830456	8.742794	2.5
197.481586	4.030236	719.556	14.68482	38.85237	3.643661	4.195662	10.65458	2.5
147.699329	5.023787	432.277	14.7033	34.23747	2.926736	4.200943	9.679513	2.5
151.468554	4.91781	476.972	15.4861	36.4821	3.148984	4.424601	10.56829	2.5
178.592128	4.31382	624.291	15.07949	38.25047	3.495624	4.308427	10.76567	2.5
373.202248	3.14408	1503.42	12.66571	41.80635	4.028432	3.618775	9.521632	2.5
205.372949	4.378954	649.364	13.84571	36.23336	3.161877	3.955918	9.46676	2.5
185.628677	4.910812	540.929	14.31029	35.48309	2.914038	4.088655	9.399479	2.5
164.791244	5.567272	609.583	20.59402	48.03569	3.699123	5.884006	15.02675	2.5
190.445592	4.714	626	15.49505	39.06504	3.287028	4.427157	10.78105	2.5
373.202248	3.14408	1456.961	12.27431	40.51444	3.903945	3.506947	9.130234	2.5
228.007621	4.108245	729.231	13.1393	35.8629	3.198275	3.754085	9.031052	2.5
298.6018	3.436154	980.545	11.2836	34.45107	3.283788	3.223886	7.847448	2.5
263.736767	3.693792	1014.182	14.20423	41.28975	3.845433	4.058351	10.51044	2.5
214.610367	4.232946	727.125	14.34172	38.26953	3.388117	4.097633	10.10877	2.5
242.610713	3.832713	787.222	12.43637	35.07878	3.244795	3.553248	8.603654	2.5
220.238457	4.06344	846.727	15.62227	42.3881	3.844592	4.463506	11.55883	2.5
271.41857	3.618914	874.25	11.65667	34.3036	3.22104	3.330476	8.037752	2.5
251.21301	4.078133	803.595	13.04537	36.547	3.198859	3.72725	8.96724	2.5
229.750171	4.023646	733.517	12.84618	35.31288	3.192672	3.670338	8.822536	2.3
203.266826	4.418844	642.25	13.96196	36.36099	3.15964	3.98913	9.543112	2.5
236.389308	3.946399	852.154	14.22628	39.57746	3.604875	4.064651	10.27988	2.5
276.14743	3.540352	1145	14.67949	43.62491	4.146336	4.194139	11.13914	2.5
198.063103	4.542732	627.25	14.38647	36.96799	3.16692	4.110419	9.843736	2.5
194.389483	4.531223	635.889	14.82259	37.93485	3.271211	4.235025	10.29136	2.5
209.538199	4.27629	665	13.57143	35.90662	3.173646	3.877551	9.295139	2.5
251.21301	4.078133	711.961	11.55781	32.37954	2.834093	3.302231	7.479675	2.5
317.918124	3.357108	1137.469	12.01129	37.46943	3.577868	3.431797	8.65418	2.5
229.750171	4.023646	816.524	14.29989	39.30899	3.553965	4.085684	10.27625	2.5
179.299342	4.002217	637.637	14.23297	36.82265	3.556271	4.066563	10.23075	2.5
170.331474	4.279685	615.429	15.46304	38.83879	3.613126	4.418011	11.18335	2.5
193.840307	3.550189	711.714	13.03505	35.43325	3.671651	3.724301	9.484866	2.5
151.749277	4.84822	451.143	14.41351	34.09227	2.97295	4.118147	9.565295	2.5
173.480707	4.294077	504	12.47525	31.45173	2.905222	3.564356	8.181171	2.5
165.853508	4.364566	550.889	14.49708	35.99369	3.32154	4.142023	10.13251	2.5
174.107944	3.627249	632.8	13.18333	34.70048	3.634527	3.766667	9.556085	2.5
230.839599	2.674851	1014.817	11.75918	35.84096	4.3962	3.359765	9.084327	2.5
181.592387	3.526066	655.728	12.73258	34.10895	3.610988	3.637881	9.206517	2.7
207.703998	2.841368	939.692	12.85488	37.58785	4.524188	3.672824	10.01352	2.5
181.592387	3.526066	678.843	13.18142	35.31132	3.738279	3.766119	9.655352	2.5
195.320262	3.196731	893.406	14.62203	40.88067	4.574057	4.177723	11.4253	2.6
177.308047	3.567566	549.312	11.05256	29.34619	3.098066	3.157873	7.484989	2.3
184.898395	3.742882	601.584	12.17781	32.28502	3.253592	3.479375	8.434931	2.5

194.742507	3.44068	689.541	12.1827	33.41549	3.540783	3.480772	8.742023	2.3
179.119387	4.098842	611.833	14.00076	35.99746	3.415783	4.000216	9.901913	2.5
184.986116	3.477183	659.538	12.39733	33.48159	3.565338	3.542095	8.920148	2.5
201.473544	3.011563	894.2	13.36622	38.22655	4.4383	3.818919	10.35466	2.5
168.265436	4.124153	564.4	13.83333	34.96165	3.354224	3.952381	9.709181	2.5
195.320262	3.196731	835.773	13.67877	38.24348	4.278988	3.908221	10.48204	2.5
206.50782	3.086813	903.35	13.50299	38.6177	4.374411	3.857997	10.41618	2.5
177.308047	3.567566	609.098	12.25549	32.54017	3.435253	3.501569	8.687927	2.5
184.898395	3.742882	592.691	11.99779	31.80776	3.205496	3.427941	8.254911	2.5
194.742507	3.44068	751.397	13.27557	36.41306	3.858413	3.793019	9.834885	2.5
222.583067	4.264043	724.587	13.88098	37.31107	3.255355	3.965993	9.616934	2.5
262.725026	3.742522	824.357	11.74298	33.99089	3.137718	3.355136	8.000455	2.5
242.374337	3.97335	834.714	13.68384	38.24198	3.443904	3.909667	9.710486	2.5
179.616222	5.031267	580.727	16.26686	39.76224	3.233155	4.647675	11.2356	2.5
339.642295	3.281568	1225.9	11.84444	37.77894	3.609386	3.384127	8.562876	2.5
313.051852	3.373404	1084.448	11.68586	36.27002	3.464116	3.338818	8.312458	2.5
340.51532	3.286827	1371.063	13.2342	42.22188	4.026436	3.7812	9.947371	2.7
226.218072	4.173765	839.061	15.48083	42.00433	3.70908	4.423094	11.30707	2.7
262.942193	3.79426	914.769	13.20013	38.08566	3.478974	3.771466	9.40587	2.5
195.221978	4.670382	599.8	14.34928	36.48583	3.0724	4.099795	9.6789	2.5
242.044659	3.954978	765.25	12.50408	34.97355	3.161607	3.572596	8.549107	2.5
243.991273	3.954478	821.3	13.31118	37.3068	3.366104	3.803195	9.356705	2.5
176.325218	5.140677	625	18.22157	44.09704	3.544587	5.206164	13.0809	2.5
340.51532	3.286827	1296.425	12.51375	39.92341	3.807244	3.575359	9.226927	2.5
226.218072	4.173765	787.27	14.52528	39.41162	3.480138	4.150079	10.35151	2.5
208.227704	4.374532	632.228	13.2821	34.88739	3.036234	3.794886	8.907569	2.5
247.475153	3.848758	914.462	14.2218	40.27239	3.695167	4.063373	10.37305	2.5
219.379063	4.139228	783.571	14.78436	39.89068	3.571767	4.224102	10.64513	2.5
267.385403	3.698277	854.615	11.8204	34.46806	3.196192	3.377257	8.122124	2.5
200.572107	4.51739	658.833	14.83858	38.30347	3.284769	4.239595	10.32119	2.5
178.552764	5.015527	545.091	15.31154	37.40085	3.052829	4.374727	10.29602	2.5
306.156768	3.439964	1274.25	14.31742	43.97564	4.162083	4.09069	10.87745	2.5
291.956722	3.463306	1034	12.26572	37.16635	3.541621	3.504491	8.802411	2.5
332.850284	3.263238	1290.636	12.65329	40.21181	3.877527	3.615227	9.390056	2.5
330.764425	3.252354	1206.8	11.86627	37.68292	3.648518	3.390364	8.613919	2.5
319.85469	3.314556	1228.5	12.73057	39.90062	3.840807	3.637306	9.416014	2.5
237.698581	3.915957	867.49	14.29143	39.89082	3.649538	4.083267	10.37548	2.5
204.639225	3.279475	886.743	14.21063	39.94012	4.333202	4.060179	10.93115	2.5
189.633834	3.632832	673.3	12.89847	34.67015	3.550527	3.685276	9.265635	2.5
192.667655	3.726647	646.5	12.50484	33.53132	3.355519	3.57281	8.778188	2.5
202.524052	3.38669	800.846	13.39207	37.24115	3.954325	3.826307	10.00538	2.5
270.732384	2.352149	1216	10.56473	34.60406	4.49152	3.018493	8.212577	2.5
244.463998	2.541206	946.308	9.836881	30.80713	3.87095	2.810538	7.295676	2.5
223.783076	2.797288	920.364	11.50455	34.40663	4.112751	3.287014	8.707262	2.5
220.286218	2.820566	1110.5	14.21895	42.26981	5.041169	4.062557	11.39838	2.5
197.248749	3.673161	720.66	13.42011	36.32869	3.653559	3.834318	9.746951	2.5
213.345108	2.926545	836.958	11.48091	33.54733	3.923024	3.280259	8.554361	2.5

175.841764	4.176764	556.091	13.20881	33.64607	3.162451	3.773946	9.032048	2.5
222.652481	3.079564	909.091	12.57387	36.66516	4.083004	3.592535	9.494309	2.5
176.62647	3.839706	593.818	12.90909	33.61901	3.361999	3.688311	9.069381	2.5
243.615445	2.668296	1024.727	11.22373	34.69406	4.20633	3.206781	8.555439	2.5
251.530029	2.341993	1322.667	12.31534	39.64581	5.258485	3.518667	9.973342	2.5
241.960879	2.557726	1047	11.06765	34.51663	4.327146	3.162187	8.509927	2.5
214.842899	2.971548	776.121	10.73473	31.30226	3.612505	3.067066	7.763183	2.5
213.345108	2.926545	711.135	9.754938	28.50404	3.333261	2.787125	6.828394	2.5
191.647961	3.714108	604.655	11.71812	31.40656	3.15503	3.348034	8.004012	2.5
343.655198	3.254311	1165.991	11.04158	35.39544	3.39291	3.154738	7.787271	2.6
343.655198	3.254311	1151.967	10.90878	34.96972	3.352101	3.116794	7.654468	2.5
341.991263	3.250868	1287.515	12.23874	39.19589	3.76476	3.496782	8.987868	2.7
282.814355	3.658659	940.3	12.16429	36.06881	3.324796	3.475513	8.505636	2.5
380.344896	3.104856	1553.25	12.67959	42.18318	4.083793	3.622741	9.574736	2.5
341.991263	3.250868	1222.082	11.61675	37.20391	3.57343	3.319071	8.365882	2.5
271.727983	3.662102	815.882	10.99571	32.27189	3.002569	3.141633	7.333612	2.5
233.898653	4.089137	746.7	13.0542	35.90039	3.192408	3.72977	8.965059	2.5
298.877318	3.451239	1152.667	13.31024	40.60368	3.856656	3.802926	9.859003	2.5
358.923586	3.181947	1227.182	10.87927	35.45497	3.419062	3.108364	7.697326	2.5
271.727983	3.662102	925.811	12.47724	36.62009	3.407124	3.564925	8.815135	2.5
262.622647	3.762502	899.4	12.88539	37.24443	3.424686	3.681539	9.122885	2.5
253.187634	3.859568	870.357	13.26764	37.75892	3.437597	3.790753	9.40807	2.5
193.53231	3.721775	550.423	10.58506	28.42458	2.844088	3.024302	6.863283	2.5
195.145513	3.717057	593.636	11.30735	30.43692	3.042017	3.230672	7.590295	2.5
184.098282	3.749456	535.4	10.90428	28.86471	2.908229	3.115508	7.154821	2.5
228.183522	2.786124	878	10.72039	32.25014	3.84778	3.062969	7.934267	2.5
189.675544	3.959823	525.555	10.97192	28.86467	2.770811	3.134834	7.012097	2.5
186.784826	3.991129	565.417	12.08156	31.59981	3.027104	3.451874	8.090431	2.5
240.762966	2.648657	928.333	10.21268	31.53413	3.855796	2.91791	7.564027	2.5
188.382969	3.737757	643.2	12.7619	34.00346	3.414321	3.646259	9.024147	2.5
217.539297	2.923915	830	11.15591	32.76411	3.815403	3.187404	8.231999	2.5
279.473384	2.136647	1517.9	11.60474	39.24527	5.431286	3.31564	9.468093	2.5
229.28143	2.834134	884.615	10.93467	32.79389	3.858206	3.124192	8.100539	2.5
232.543299	2.685257	794.875	9.178695	28.00015	3.418181	2.622484	6.493438	2.5
215.370824	2.902572	799.996	10.78162	31.64353	3.714505	3.080462	7.879045	2.5
168.993372	5.236046	595.08	18.4378	43.94664	3.521322	5.267943	13.20175	2.5
148.751155	5.934143	621.25	24.78358	55.45486	4.176438	7.081023	18.84944	2.6
147.398924	6.004029	430.75	17.54582	39.0559	2.922342	5.013093	11.5418	2.5
155.663784	5.665652	541.75	19.71793	45.14354	3.480257	5.633693	14.05227	2.6
187.91345	4.877701	643.17	16.69487	41.59286	3.422693	4.769964	11.81717	2.5
135.516034	6.44302	696.58	33.11843	70.9243	5.140204	9.462409	26.67541	2.6
135.516034	6.44302	555.5	26.41088	56.55984	4.099146	7.545965	19.96786	2.7
135.516034	6.44302	523.42	24.88566	53.29352	3.862421	7.110187	18.44264	2.7
183.307101	4.97104	721.75	19.57288	48.23228	3.937382	5.592252	14.60184	2.6
155.074092	5.696018	555.25	20.39486	46.58678	3.580547	5.827102	14.69884	2.7
193.014982	4.699087	663.58	16.15533	40.89876	3.437971	4.615807	11.45624	2.3
153.558465	5.666364	517.42	19.09299	43.56285	3.369531	5.45514	13.42662	2.4

158.912425	5.579203	542.42	19.04364	43.9943	3.413327	5.44104	13.46444	2.7
143.235541	6.101621	476.75	20.30884	44.70298	3.328434	5.802525	14.20722	2.6
168.648329	5.286781	704.25	22.0768	52.46671	4.175849	6.307658	16.79002	2.7
134.009471	6.634132	685.33	33.92723	71.92605	5.114042	9.693494	27.2931	2.6
134.009471	6.634132	661.33	32.73911	69.40723	4.93495	9.354031	26.10498	2.7
154.38098	5.707245	488.25	18.04991	41.16393	3.162631	5.157116	12.34266	2.5
153.966176	5.745007	394.5	14.72015	33.49237	2.562251	4.205757	8.975143	2.4
167.739245	5.299818	653.92	20.66098	49.00544	3.898432	5.903137	15.36116	2.3
158.556237	5.550329	635.83	22.2575	51.45677	4.010123	6.359286	16.70717	2.5
158.556237	5.550329	663.25	23.21735	53.67583	4.183058	6.633528	17.66702	2.7
141.069075	6.241994	537	23.76106	51.80754	3.806646	6.788875	17.51907	2.5
168.003617	5.324996	621.42	19.69635	46.68052	3.698849	5.62753	14.37136	2.7
190.389934	4.805764	677.67	17.10554	42.9148	3.559379	4.887296	12.29977	2.7
234.579329	4.212764	919.75	16.51761	45.12091	3.920848	4.719317	12.30484	2.7
137.4247	6.421715	523.42	24.45888	52.60657	3.808777	6.988251	18.03716	2.7
137.4247	6.421715	557.92	26.07103	56.07401	4.059823	7.448865	19.64931	2.7
174.368471	5.166473	534.33	15.832	38.15964	3.064373	4.523429	10.66553	2.3
136.355385	5.669663	527.33	21.9264	48.55637	3.867321	6.264687	16.25674	2.3
137.841503	5.580628	505	20.44534	45.57941	3.663628	5.841527	14.86472	2.5
158.210113	4.870551	594.75	18.30958	43.71116	3.759241	5.231308	13.43903	2.5
153.196121	5.043494	636.75	20.96296	49.21322	4.156437	5.989418	15.91947	2.3
163.107795	4.769234	622	18.18713	43.98158	3.813429	5.196324	13.4179	2.7
149.426896	5.157097	576	19.87921	46.12166	3.854728	5.679773	14.72211	2.5
127.963273	5.993596	438.92	20.55831	44.19132	3.430047	5.873804	14.56472	2.3
138.975887	5.649426	610.92	24.83415	55.30737	4.39587	7.09547	19.18472	2.7
135.553177	5.695512	465.17	19.54496	43.1697	3.431642	5.584274	13.84945	2.5
160.774817	4.842615	473.75	14.26958	34.25277	2.946668	4.077022	9.426963	2.3
131.032932	6.08974	382.08	17.75712	38.24441	2.915908	5.073464	11.66738	2.5
140.08276	5.544099	577.42	22.85273	51.23616	4.121992	6.529352	17.30863	2.3
140.08276	5.544099	542.58	21.47386	48.1447	3.873282	6.135388	15.92976	2.4
140.08276	5.544099	561.5	22.22266	49.82353	4.008345	6.349332	16.67856	2.5
154.882145	5.028641	546.92	17.75714	41.83221	3.531201	5.073469	12.7285	2.4
154.513865	4.997699	583.17	18.86244	44.4782	3.774224	5.389268	13.86474	2.7
181.195391	4.343442	652.42	15.63919	39.74591	3.600643	4.468339	11.29575	2.4
155.498682	4.923493	641.08	20.29826	48.11962	4.122736	5.799503	15.37477	2.6
154.797533	4.940399	478.83	15.28197	36.15596	3.093266	4.366277	10.34157	2.6
236.08338	3.724711	823.75	12.99639	36.67042	3.489233	3.713253	9.271676	2.7
137.42327	5.655279	545	22.42798	49.79569	3.965849	6.407995	16.7727	2.6
146.475821	5.130502	653.92	22.90438	52.9444	4.464355	6.544108	17.77388	2.7
146.475821	5.130502	558.83	19.57373	45.24547	3.815169	5.592494	14.44323	2.4
146.475821	5.130502	616.42	21.59089	49.90823	4.20834	6.168827	16.46039	2.7
175.50344	4.394948	693.75	17.37285	43.6721	3.952914	4.963671	12.9779	2.6
129.838585	6.006318	480.5	22.22788	47.92883	3.700749	6.350822	16.22156	2.7
120.467547	6.442115	433.75	23.19519	48.23456	3.600555	6.627196	16.75307	2.5
141.162708	5.436235	508.33	19.576	44.19055	3.601022	5.593143	14.13977	2.3
152.726621	4.830522	467.83	14.79679	35.08711	3.063186	4.227653	9.966264	2.3
112.862778	7.28147	406.08	26.19871	51.98318	3.597998	7.485346	18.91724	2.5

112.862778	7.28147	448.92	28.96258	57.46722	3.977574	8.275023	21.68111	2.6
231.095685	3.748511	878.33	14.24704	39.9216	3.80072	4.070583	10.49853	2.7
158.286299	4.850498	547.08	16.76462	40.06896	3.456269	4.789893	11.91413	2.3
168.993372	5.236046	691.83	21.43548	51.09162	4.093829	6.124422	16.19943	2.5
168.865864	5.408034	573.67	18.37214	43.42955	3.397193	5.249182	12.9641	2.5
211.953867	4.345543	986.33	20.22204	53.44096	4.653513	5.777726	15.8765	2.7
173.206348	5.268634	680.17	20.68958	49.54139	3.926935	5.911309	15.42095	2.7
171.920178	5.225537	609.83	18.53587	44.39273	3.547169	5.295962	13.31033	2.7
194.591056	4.575921	723.42	17.01164	43.44175	3.717643	4.860469	12.43572	2.6
176.811435	5.110157	623.83	18.02977	43.72796	3.528222	5.151363	12.91961	2.4
185.515638	4.831136	858	22.34375	55.62101	4.624947	6.383929	17.51261	2.7
242.301959	3.878383	1037.58	16.60792	46.69194	4.282178	4.745121	12.72954	2.5
169.501588	5.415386	606.83	19.38754	45.85733	3.580084	5.539297	13.97215	2.7
160.441495	5.736815	586.08	20.95613	48.19173	3.65292	5.987465	15.21931	2.4
175.937533	5.159458	510.67	14.97566	36.18883	2.902564	4.27876	9.816201	2.3
239.840428	3.915762	808.67	13.20278	36.93531	3.3717	3.772222	9.287013	2.4
213.170411	4.240763	843.83	16.78696	44.69848	3.958476	4.796274	12.5462	2.6
173.552921	5.165266	611.75	18.20685	43.83485	3.524861	5.201956	13.04158	2.3
185.804266	4.93727	566.42	15.05115	37.2788	3.048477	4.300329	10.11388	2.4
183.665118	4.795434	732.75	19.13185	47.5945	3.989598	5.466244	14.33642	2.3
182.95186	5.019255	543.33	14.90617	36.62609	2.969798	4.258907	9.886917	2.4
216.887704	4.136473	727.75	13.87962	37.34897	3.355423	3.965605	9.743145	2.5
194.730435	4.555098	725.25	16.96491	43.37961	3.724379	4.847118	12.40981	2.7
193.425084	4.649641	769.5	18.4976	46.97736	3.978284	5.285027	13.84795	2.7
156.648556	5.922441	495.17	18.72098	42.45561	3.161025	5.348852	12.79854	2.4
227.884273	4.023878	866.5	15.30027	41.97265	3.802369	4.371505	11.27639	2.6
162.231137	5.636353	548.92	19.07098	44.17301	3.383567	5.448851	13.43463	2.6
162.231137	5.636353	521.08	18.10374	41.93266	3.21196	5.172498	12.46739	2.7
162.231137	5.636353	681.92	23.69176	54.87587	4.203385	6.769075	18.05541	2.7
201.699296	3.455708	790.17	13.53796	37.41924	3.917564	3.867988	10.08225	2.4
203.73784	3.189634	730.25	11.43249	32.32015	3.584263	3.266424	8.242852	2.5
160.096518	4.833102	684.33	20.65902	49.56198	4.274484	5.902577	15.82592	2.7
172.576349	4.102416	776.67	18.46269	47.01976	4.500443	5.275054	14.36027	2.6
141.375579	5.882561	443.17	18.44006	40.82859	3.1347	5.268589	12.5575	2.3
161.21654	4.528554	585.17	16.43736	40.15083	3.629714	4.696388	11.90881	2.3
217.756525	2.826795	1011.75	13.13398	38.91039	4.646244	3.752566	10.30719	2.6
149.333039	5.295498	508.67	18.03794	41.56706	3.406279	5.153698	12.74245	2.7
172.833108	4.241303	556.92	13.66675	34.53004	3.222299	3.904785	9.425445	2.5
183.684359	3.909592	681	14.4946	37.94823	3.707447	4.141316	10.58501	2.5
184.547821	3.68234	538.67	10.74825	28.59789	2.918864	3.070928	7.065909	2.4
174.833487	4.170945	722.58	17.23835	43.86249	4.132961	4.925243	13.06741	2.7
225.05075	4.186991	833.17	15.50084	41.97104	3.702143	4.428811	11.31385	2.3
210.675255	4.36859	768.58	15.93738	41.99861	3.648174	4.553536	11.56879	2.7
265.688711	3.72113	1027	14.38375	41.8116	3.865426	4.109644	10.66262	2.4
212.263891	4.396973	865.17	17.9217	47.23998	4.075917	5.120485	13.52473	2.7
325.77097	3.346389	1283.33	13.18264	41.4082	3.939363	3.766469	9.836251	2.7
187.368653	4.86356	637.33	16.54328	41.2152	3.401476	4.726652	11.67972	2.7

222.67269	4.173809	780.5	14.6298	39.53867	3.505145	4.179944	10.45599	2.5
218.55862	4.254845	826.25	16.08523	43.06241	3.78045	4.59578	11.83038	2.3
241.683449	3.882465	911.58	14.64386	41.13299	3.771793	4.183959	10.76139	2.7
241.683449	3.882465	864.33	13.88482	39.00094	3.57629	3.967091	10.00235	2.7
217.607937	4.240465	691.83	13.4815	36.08307	3.17925	3.851856	9.241032	2.5
252.507493	3.823959	883	13.3721	38.11886	3.496926	3.820601	9.548143	2.3
208.090192	3.429587	900.42	14.84005	41.41787	4.327066	4.240014	11.41046	2.7
202.199682	3.280705	892.75	14.48494	40.5854	4.41519	4.138553	11.20423	2.4
188.204871	3.6705	795.08	15.50619	41.49364	4.224545	4.430341	11.83569	2.7
182.910449	4.080545	741.92	16.55148	42.82692	4.056193	4.728994	12.47093	2.6
211.62437	3.055948	927	13.38628	38.61579	4.380403	3.824652	10.33033	2.5
180.877921	3.940695	620.42	13.51678	35.18247	3.430048	3.861936	9.57608	2.5
204.640391	3.736564	775.92	14.16766	38.54142	3.791627	4.047902	10.43109	2.3
213.500147	2.936728	970.33	13.34704	38.97343	4.544868	3.813441	10.41031	2.7
195.686145	3.614914	724.33	13.38056	36.29442	3.701488	3.823018	9.765649	2.6
198.747033	3.374313	673.75	11.43888	31.68928	3.389988	3.268251	8.064567	2.6
190.694219	3.61047	767.58	14.53282	39.1781	4.025188	4.152235	10.92235	2.7
191.446584	4.029012	655.17	13.78812	36.20072	3.422208	3.939462	9.759106	2.6
190.112104	3.554627	652	12.19079	32.96748	3.429555	3.483083	8.636163	2.6
190.112104	3.554627	714.5	13.35939	36.12771	3.758309	3.816967	9.804758	2.7
150.652548	5.907943	321.9483	12.62542	28.37142	2.137025	3.607264	6.71748	2.7
153.21141	5.124127	470.4085	15.73273	36.78932	3.070323	4.495065	10.6086	2.7
146.84804	5.134547	703.0188	24.58108	56.84502	4.78739	7.023165	19.44653	2.7
243.977643	3.884994	1354.207	21.56381	60.70372	5.550537	6.161087	17.67881	2.7
186.252389	4.825191	642.5928	16.64748	41.49496	3.450118	4.756423	11.82229	2.7
174.066748	5.211579	635.9538	19.04053	45.77367	3.653505	5.440152	13.82895	2.5
198.786751	4.559329	1362.398	31.24766	80.29512	6.853565	8.927903	26.68833	2.7
214.868063	4.213099	728.7639	14.28949	38.18644	3.391681	4.082711	10.07639	2.7
302.683714	3.416295	1827.404	20.62533	63.27895	6.037338	5.892951	17.20903	2.7
265.2204	3.678508	1333.553	18.49588	53.89629	5.028094	5.284537	14.81737	2.7
284.538029	3.465749	1198.472	14.59771	43.94106	4.211992	4.170774	11.13196	2.6
181.903868	4.929644	571.5293	15.4886	38.17409	3.14193	4.425314	10.55895	2.7
197.913726	4.549741	846.3358	19.456	49.96614	4.276287	5.558856	14.90625	2.7
280.143566	3.586985	1811.011	23.18836	68.93389	6.464582	6.625246	19.60138	2.3
283.013866	3.528851	912.5122	11.37796	34.04928	3.224267	3.250845	7.849106	2.7
282.845446	3.531154	1598.987	19.96238	59.7201	5.653218	5.703538	16.43123	2.3
206.277689	3.238268	917.5117	14.40364	40.69181	4.447944	4.115325	11.16537	2.5
156.446477	4.684026	469.1906	14.04762	33.77065	2.999049	4.013607	9.363597	2.7
179.517535	3.852308	609.9519	13.0891	34.19843	3.397729	3.739742	9.236789	2.7
229.895689	2.594759	987.031	11.1403	34.1787	4.293386	3.182944	8.545545	2.3
193.799292	3.347138	589.441	10.18033	28.08222	3.041502	2.908665	6.83319	2.7
229.895689	2.594759	1130.672	12.76153	39.15267	4.918196	3.646153	10.16678	2.7
178.715688	3.662207	1009.862	20.69389	54.69494	5.650662	5.912541	17.03169	2.7
183.09226	3.822385	887.7376	18.53314	48.75655	4.848581	5.295184	14.71076	2.5
193.451667	3.417874	1318.353	23.29246	63.88801	6.814896	6.654987	19.87458	2.7
197.102177	3.268693	922.7008	15.30184	42.64056	4.681332	4.371954	12.03314	2.5
181.053218	3.835873	1115.068	23.62432	61.92198	6.158786	6.749806	19.78845	2.6

223.690843	2.704847	1524.632	18.4357	55.59502	6.8158	5.267342	15.73085	2.3
232.14411	2.724696	1315.172	15.43629	46.89784	5.665326	4.410369	12.71159	2.3
200.804597	3.157305	1061.115	16.6842	47.11613	5.284316	4.766914	13.52689	2.3
201.619076	3.262445	1341.224	21.70265	60.84991	6.652267	6.200758	18.44021	2.7
162.318064	4.521395	883.9446	24.62241	60.27042	5.445756	7.034975	20.10102	2.4
233.432702	4.059699	941.833	16.3797	45.10483	4.034709	4.679916	12.32001	2.7
252.003448	3.761245	1061.041	15.83643	45.30812	4.210423	4.524695	12.07519	2.6
355.638523	3.198188	1384.464	12.45022	40.42994	3.892897	3.557205	9.252028	2.3
210.179427	2.715496	691.3965	8.932771	26.49546	3.289554	2.55222	6.217275	2.3
210.179427	2.715496	738.462	9.540853	28.29909	3.513484	2.725958	6.825356	2.7
230.323957	2.700164	1025.993	12.02805	36.55381	4.454565	3.436587	9.32789	2.3
177.505143	3.971032	868.9344	19.43925	50.26392	4.895263	5.554071	15.46822	2.3
170.215367	4.287541	991.0935	24.96457	62.66452	5.822585	7.132735	20.67703	2.5
159.175701	5.585112	591.8908	20.7681	47.98527	3.718475	5.933743	15.18299	2.5
169.785573	5.240295	694.6685	21.44039	51.15273	4.091446	6.125825	16.20009	2.5
174.714028	5.199822	608.2398	18.10238	43.58333	3.481345	5.172107	12.90255	2.5
182.913166	4.970466	763.6147	20.7504	51.10795	4.174739	5.928686	15.77993	2.5
159.867536	5.49373	511.1439	17.56508	40.79655	3.197296	5.018595	12.07135	2.5
151.977381	5.822888	548.2412	21.00541	47.47788	3.607387	6.001546	15.18252	2.5
160.881859	5.490848	529.2981	18.06478	42.02905	3.28998	5.161366	12.57393	2.5
157.237484	5.59564	520.4415	18.52105	42.64245	3.309907	5.291728	12.92541	2.5
176.51214	5.101507	672.904	19.44809	47.16785	3.812225	5.556598	14.34659	2.5
155.077659	5.722423	387.343	14.2931	32.61134	2.497735	4.083742	8.570675	2.5
165.016072	5.375116	583.3053	19.00017	44.72415	3.534839	5.428621	13.62506	2.5
162.784668	5.462573	558.1959	18.73141	43.76475	3.429045	5.35183	13.26883	2.5
148.426488	5.86666	467.3321	18.47162	41.42717	3.148576	5.277607	12.60496	2.5
142.675018	6.23035	516.0524	22.53504	49.29663	3.616978	6.438583	16.30469	2.5
150.129592	5.841618	537.0686	20.89761	47.0522	3.577367	5.970746	15.05599	2.5
161.280506	5.485731	703.1036	23.91509	55.68764	4.359508	6.832882	18.42936	2.5
145.25005	6.102943	399.2895	16.77687	37.05571	2.74898	4.793391	10.67393	2.5
186.960768	4.831028	594.0728	15.35072	38.28745	3.177526	4.385919	10.51969	2.5
144.398498	6.11858	619.7696	26.26142	57.88236	4.292078	7.503264	20.14284	2.5
152.685774	5.761727	541.6994	20.44149	46.37928	3.547805	5.840425	14.67976	2.5
217.531024	4.448487	1086.427	22.21732	58.75148	4.994354	6.347805	17.76883	2.5
175.859531	5.112196	788.7437	22.9286	55.52864	4.485078	6.551027	17.8164	2.5
177.10744	5.148472	560.5126	16.29397	39.46086	3.164817	4.65542	11.1455	2.5
151.417489	5.779293	533.3459	20.35671	46.05565	3.522353	5.816203	14.57742	2.5
161.113155	5.594207	590.1988	20.49301	47.47379	3.663256	5.855147	14.89881	2.5
197.892365	4.700531	764.4364	18.15763	46.25192	3.86289	5.187896	13.4571	2.5
166.266271	5.363428	531.0938	17.13206	40.42499	3.194237	4.894874	11.76863	2.5
167.208252	5.359239	343.6515	11.01447	26.03168	2.05523	3.146992	5.655232	2.5
129.987588	6.735108	252.7836	13.09759	27.45244	1.944675	3.74217	6.362487	2.5
153.006814	5.730592	336.6702	12.60937	28.66297	2.200361	3.602677	6.878778	2.5
123.549628	7.267625	179.0825	10.53427	21.39028	1.449478	3.00979	3.266641	2.5
174.590482	5.135014	527.4735	15.51393	37.46204	3.021204	4.43255	10.37891	2.5
123.366481	7.387214	194.2326	11.6307	23.51175	1.574436	3.323056	4.243483	2.5
176.371272	5.024823	328.5182	9.359493	22.78133	1.862652	2.674141	4.334671	2.5

156.676829	5.575688	359.7179	12.80135	29.47354	2.295923	3.657528	7.225661	2.5
152.677495	5.739755	274.0753	10.30358	23.39961	1.795126	2.943881	4.563828	2.5
135.622146	6.583599	240.4372	11.67171	24.86574	1.772846	3.334774	5.088111	2.5
151.179766	5.107425	631.1665	21.32319	49.73649	4.17494	6.092341	16.21577	2.5
139.637602	5.607936	773.6576	31.07059	69.40637	5.540468	8.877311	25.46265	2.5
147.467477	5.285573	574.121	20.57781	47.29337	3.893204	5.879375	15.29224	2.5
145.301087	5.264532	466.9625	16.91893	38.77932	3.213758	4.833981	11.6544	2.5
145.098966	5.257209	416.4528	15.08887	34.5847	2.87013	4.311106	9.831662	2.5
146.076673	5.350794	481.633	17.64224	40.32685	3.297125	5.040639	12.29144	2.5
135.183677	5.77708	499.4529	21.34414	46.94428	3.694625	6.098327	15.56706	2.5
177.364331	4.594931	838.7149	21.72836	54.1594	4.728768	6.208104	17.13343	2.5
171.057897	4.623186	564.5836	15.25902	37.6337	3.300541	4.359719	10.63583	2.5
146.491352	5.307658	398.3807	14.43408	33.08388	2.719483	4.124023	9.126425	2.5
164.673652	4.691557	640.1139	18.23686	44.38916	3.887166	5.210532	13.54531	2.5
184.899616	4.340367	581.5395	13.65116	34.87563	3.145163	3.900332	9.310796	2.5
157.034933	4.907342	627.074	19.59606	46.60755	3.993213	5.598875	14.68872	2.5
145.559616	5.293077	508.09	18.476	42.30981	3.490597	5.278857	13.18292	2.5
147.825748	5.223525	468.8327	16.56653	38.21009	3.171523	4.733293	11.343	2.5
161.486749	4.708068	733.1767	21.37541	51.72947	4.540166	6.107261	16.66734	2.5
154.017255	4.984377	521.141	16.8654	39.76366	3.383653	4.818687	11.88103	2.5
165.706713	4.720989	751.3994	21.40739	52.10633	4.534514	6.116397	16.6864	2.5
135.306404	5.709131	465.358	19.63536	43.32374	3.43929	5.610103	13.92623	2.5
144.785611	5.323	505.8977	18.59918	42.47526	3.494116	5.314052	13.27618	2.5
163.306361	4.789043	1189.491	34.88243	84.29374	7.2838	9.966409	30.09339	2.5
171.538895	4.550103	789.5895	20.94402	51.89737	4.602977	5.984006	16.39392	2.5
148.655633	5.179639	668.4867	23.29222	53.91149	4.496881	6.65492	18.11258	2.5
166.840043	4.712996	496.3548	14.02132	34.20108	2.975034	4.006092	9.308327	2.5
163.158012	4.661657	694.0122	19.82892	48.22987	4.25362	5.665406	15.16726	2.5
151.270653	5.180502	419.184	14.35562	33.37087	2.771086	4.101605	9.175115	2.5
142.575887	5.421136	356.0267	13.53714	30.65602	2.497103	3.867753	8.116	2.5
156.486035	4.967811	665.2058	21.11765	50.02915	4.250896	6.033613	16.14984	2.5
126.69156	6.240964	370.492	18.25084	38.73975	2.924362	5.214524	12.00987	2.5
130.773612	5.864288	426.4719	19.1243	41.55868	3.261146	5.464086	13.26001	2.5
149.093824	5.213071	318.5855	11.13935	25.76033	2.136812	3.182672	5.926283	2.5
132.78227	5.823784	315.3699	13.83201	30.22519	2.37509	3.952003	8.008228	2.5
174.554991	4.43033	474.988	12.05553	30.20372	2.721137	3.444438	7.625203	2.5
140.556792	5.512031	319.9141	12.54565	28.19215	2.276049	3.584472	7.03362	2.5
145.997016	5.27065	284.8015	10.28164	23.58753	1.950735	2.937612	5.010993	2.5
128.856368	6.078131	277.9798	13.11226	28.13594	2.157284	3.746359	7.034124	2.5
137.528675	5.706584	330.5411	13.7154	30.38874	2.403434	3.918685	8.008814	2.5
128.070344	6.041054	360.0114	16.98167	36.43883	2.811044	4.851906	10.94062	2.5
154.27552	4.992735	582.082	18.83761	44.41354	3.773003	5.382173	13.84487	2.5
145.288803	5.188886	451.8109	16.1361	37.11831	3.109743	4.610315	10.94722	2.5
139.746463	5.333834	285.7775	10.90754	24.67755	2.044972	3.11644	5.573705	2.5
144.574422	5.200519	343.2137	12.34582	28.34856	2.373959	3.527377	7.145299	2.5
135.14999	5.775641	249.3492	10.65595	23.43668	1.844981	3.044557	4.880308	2.5
142.396225	5.414305	335.289	12.74863	28.87038	2.35462	3.642466	7.334324	2.5

134.824879	5.811417	253.5647	10.92951	23.98683	1.880697	3.122718	5.118095	2.5
168.882124	4.69117	477.5935	13.26648	32.49612	2.827969	3.790424	8.575315	2.5
145.929642	5.268218	283.9331	10.25029	23.5156	1.945685	2.928655	4.982073	2.5
121.539859	6.264941	253.5665	13.07044	27.43093	2.086282	3.734411	6.805496	2.5
216.754105	4.423553	1051.8	21.46531	56.79187	4.852504	6.132945	17.04176	2.5
222.746767	4.194854	950.4956	17.9001	48.32025	4.267158	5.114316	13.70525	2.5
216.551346	4.464976	846.0042	17.44338	46.03267	3.906714	4.983824	12.97841	2.5
202.115841	4.491463	1162.371	25.83047	66.90142	5.751014	7.380133	21.339	2.5
174.987603	5.161876	783.5334	23.11308	55.77091	4.477651	6.603737	17.9512	2.5
159.683626	5.563889	526.7162	18.35248	42.47812	3.298499	5.243566	12.78859	2.5
178.495634	5.013922	839.5327	23.58238	57.60366	4.703379	6.737822	18.56846	2.5
206.257855	4.426134	996.0476	21.37441	55.84582	4.829138	6.106975	16.94828	2.5
228.95035	4.009638	918.6394	16.08825	44.22501	4.012396	4.596644	12.07862	2.5
208.169353	4.429135	755.612	16.07685	42.09451	3.629795	4.593386	11.64772	2.5
182.565722	5.029359	827.0665	22.7842	55.92558	4.53024	6.509772	17.75484	2.5
180.210758	4.978198	603.8815	16.68181	40.91856	3.350974	4.766232	11.70361	2.5
152.829696	5.789004	418.2276	15.84195	35.90953	2.736559	4.526272	10.05295	2.5
198.110156	4.694553	854.7057	20.25369	51.6217	4.314295	5.786768	15.55914	2.5
183.812396	4.91477	783.6211	20.95244	51.81463	4.263157	5.98641	16.03767	2.5
190.451533	4.7376	567.4892	14.11665	35.54578	2.979704	4.033328	9.379047	2.5
183.125613	4.976239	508.2325	13.81067	34.01548	2.775322	3.945905	8.834427	2.5
167.058223	5.477319	719.7048	23.59688	55.45357	4.308108	6.741965	18.11956	2.5
171.036411	5.31169	609.9375	18.94216	45.12253	3.566127	5.412046	13.63047	2.5
170.622856	5.154769	764.1608	23.08643	55.375	4.478655	6.596123	17.93166	2.5
224.003629	4.218524	782.7858	14.74173	39.79441	3.494523	4.211922	10.5232	2.5
168.370868	5.448895	521.7041	16.88363	39.80663	3.098542	4.823894	11.43473	2.5
159.719797	5.56515	540.6184	18.83688	43.59929	3.384793	5.381965	13.27173	2.5
218.02629	4.266659	654.7369	12.81286	34.25714	3.003018	3.660816	8.546196	2.5
221.117949	4.18784	682.6825	12.92959	34.85325	3.087413	3.694169	8.741753	2.5
162.38373	5.523256	433.689	14.75133	34.3493	2.670767	4.214665	9.228072	2.5
179.647827	5.060502	575.4688	16.21039	39.56858	3.203316	4.631539	11.14989	2.5
175.082611	5.074858	658.3791	19.08345	46.25	3.760391	5.452415	14.00859	2.5
193.190279	4.643997	620.094	14.90611	37.85624	3.209758	4.258887	10.26211	2.5
169.583578	5.299487	642.3717	20.07411	47.74456	3.787936	5.735461	14.77463	2.5
179.139139	5.08918	672.8209	19.11423	46.5578	3.755857	5.461209	14.02505	2.5
228.412944	4.035564	941.3645	16.63188	45.61897	4.121327	4.751966	12.59632	2.5
208.37586	4.424116	818.9232	17.3869	45.54886	3.930029	4.967687	12.96279	2.5
201.867056	4.546555	650.9812	14.66174	37.84698	3.224802	4.189068	10.11518	2.5
206.795261	4.390558	630.2657	13.38144	35.05565	3.047777	3.823268	8.990881	2.5
187.992497	4.82032	710.4202	18.2159	45.52153	3.778982	5.204544	13.39558	2.5
174.769604	5.125208	646.3526	18.95462	45.80403	3.698312	5.415606	13.82941	2.5
178.134338	5.193421	761.3014	22.19538	53.71382	4.273749	6.341536	17.00196	2.5
209.450671	4.354484	807.7394	16.79292	44.22445	3.856466	4.797977	12.43844	2.5
198.072014	4.574411	703.5311	16.24783	41.67901	3.551896	4.642238	11.67342	2.5
207.669506	4.34455	398.2373	8.331323	21.9064	1.917649	2.380378	3.986773	2.5
207.665842	4.514475	721.9621	15.69483	40.87389	3.476557	4.484237	11.18035	2.5
225.733077	4.141891	739.5286	13.56933	36.86868	3.27612	3.876952	9.427441	2.5

240.076031	3.948619	1068.062	17.56681	49.05335	4.448848	5.019087	13.61819	2.5
198.22611	4.653195	708.4517	16.63032	42.4867	3.573957	4.75152	11.97713	2.5
207.049279	4.580736	704.418	15.58447	40.40886	3.402176	4.452706	11.00373	2.5
159.015873	4.892796	639.7608	19.68495	47.00079	4.023251	5.624271	14.79215	2.5
172.307014	4.418129	670.5353	17.19321	42.96583	3.891515	4.912347	12.77508	2.5
208.073623	3.394349	1009.978	16.47599	46.10166	4.853946	4.707425	13.08164	2.5
160.199906	4.914108	585.2447	17.95229	42.89674	3.653215	5.129226	13.03818	2.5
225.246	3.798415	1002.635	16.90783	46.91931	4.451287	4.83081	13.10942	2.5
168.266477	4.28159	584.4711	14.87204	37.23648	3.473485	4.249154	10.59045	2.5
181.162232	4.323681	818.2042	19.52755	49.68224	4.516417	5.579299	15.20387	2.5
163.003444	4.794219	950.7864	27.96431	67.52643	5.832922	7.989802	23.17009	2.5
184.885764	3.773179	852.2371	17.39259	46.01648	4.609533	4.969313	13.61942	2.5
184.226408	4.344962	702.8932	16.57767	42.30239	3.815377	4.736477	12.23271	2.5
195.802523	3.599311	1246.863	22.92027	62.24716	6.367961	6.54865	19.32096	2.5
172.111653	4.147269	821.3392	19.79131	50.2327	4.77213	5.654659	15.64404	2.5
195.028366	3.618337	713.8387	13.24376	35.88463	3.660179	3.783932	9.625424	2.5
186.762035	4.323195	592.9746	13.72626	35.19032	3.175027	3.92179	9.403068	2.5
182.293535	3.997665	751.7931	16.48669	42.84249	4.12408	4.710483	12.48903	2.5
170.226957	4.600729	715.1736	19.32901	47.67165	4.201294	5.522576	14.72829	2.5
190.43013	3.283278	973.39	16.78259	46.31438	5.111533	4.795025	13.49931	2.5
163.673793	4.623553	463.1765	13.08408	31.91495	2.829876	3.738309	8.46053	2.5
179.058315	4.62683	439.8014	11.36438	28.3448	2.456191	3.246965	6.737549	2.5
191.826874	3.539241	705.8485	13.02304	35.33557	3.679612	3.720867	9.483795	2.5
180.511968	3.932723	702.2179	15.29887	39.82102	3.890146	4.371104	11.36614	2.5
182.690947	4.42351	720.1748	17.43765	44.20541	3.942039	4.982185	13.01414	2.5
162.93614	4.276539	581.2918	15.257	37.90534	3.567605	4.359143	10.98046	2.5
169.633448	4.117317	732.5216	17.77965	45.0451	4.318262	5.0799	13.66233	2.5
181.33873	4.02975	763.3618	16.96359	43.93605	4.20959	4.846741	12.93385	2.5
223.316222	2.687319	1048.856	12.62161	38.10789	4.696728	3.606173	9.934288	2.5
194.794557	4.234664	868.376	18.87774	49.16312	4.457907	5.39364	14.64308	2.5
162.779921	4.844641	597.2098	17.7741	42.79297	3.668817	5.078315	12.92946	2.5
169.72117	3.771582	826.0308	18.35624	47.54303	4.866988	5.24464	14.58466	2.5
181.758156	4.369186	654.0476	15.7223	39.92908	3.59845	4.492085	11.35311	2.5
165.052354	4.210519	589.8773	15.04789	37.65279	3.57388	4.299397	10.83737	2.5
191.826527	3.506883	785.2186	14.355	39.03912	4.093378	4.101429	10.84812	2.5
205.376183	3.383463	878.4577	14.47212	40.39516	4.27731	4.134891	11.08866	2.5
176.498913	4.537247	676.797	17.39838	43.45065	3.834568	4.970966	12.86113	2.5
180.356581	3.870313	668.4867	14.34521	37.48032	3.706472	4.098631	10.4749	2.5
162.088974	5.195159	569.337	18.24798	43.12742	3.512497	5.213709	13.05282	2.5
155.852621	4.652317	628.2192	18.75281	45.11569	4.030854	5.357946	14.10049	2.5
		872.4876	16.55574	44.60682		4.730212	16.55574	2.5
216.595068	4.230372	767.83	14.99668	40.11554	3.545002	4.284766	10.76631	2.5
199.163426	4.620961	709.9654	16.47251	42.20649	3.564738	4.706433	11.85155	2.5
248.889081	3.919513	889.0772	14.00122	39.52384	3.572182	4.000347	10.0817	2.5
323.752638	3.348011	1413.635	14.61877	45.84241	4.366405	4.176791	11.27076	2.5
235.420373	4.065982	903.0533	15.59677	43.02337	3.835918	4.456221	11.53079	2.5
222.403987	4.188399	941.5762	17.73213	47.86682	4.23363	5.066324	13.54373	2.5

227.956431	4.122178	933.8723	16.88738	46.05148	4.096714	4.824966	12.7652	2.5
210.734266	4.464709	744.4529	15.77231	41.34097	3.532662	4.506373	11.3076	2.5
179.069905	5.044223	484.9644	13.66097	33.34561	2.708241	3.903134	8.616748	2.5
244.219266	3.876496	873.8897	13.87127	39.0797	3.5783	3.963219	9.994769	2.5
203.353659	4.51897	688.2557	15.29457	39.61325	3.384526	4.369878	10.7756	2.5
236.047055	4.028107	797.1792	13.60374	37.63854	3.377205	3.886783	9.575634	2.5
198.725956	4.754209	793.6862	18.98771	48.27993	3.993873	5.425059	14.2335	2.5
196.02302	4.623184	573.1458	13.51759	34.49378	2.92387	3.862169	8.894406	2.5
244.891724	3.89955	891.8719	14.20178	39.97907	3.641903	4.057652	10.30223	2.5
211.809078	4.394379	580.0439	12.03411	31.70849	2.738522	3.438316	7.639727	2.5
191.639383	4.720182	616.8574	15.19353	38.3522	3.218845	4.341009	10.47335	2.5
241.557221	4.005924	952.8101	15.80116	44.032	3.944449	4.514618	11.79524	2.5
181.267054	5.049222	502.7927	14.00537	34.28216	2.773768	4.001534	8.956147	2.5
211.939411	4.38798	612.2013	12.67497	33.41442	2.888567	3.621421	8.286995	2.5
198.271358	4.610962	596.232	13.86586	35.507	3.007151	3.961674	9.254899	2.5
224.081024	4.172831	649.4205	12.09349	32.73748	2.89815	3.455283	7.920661	2.5
191.460831	4.762707	772.4921	19.21622	48.38653	4.034726	5.490349	14.45351	2.5
260.129716	3.76454	901.9951	13.05347	37.63532	3.467482	3.729564	9.288935	2.5
289.13936	3.539037	749.0295	9.168047	27.56337	2.590548	2.619442	5.62901	2.5
233.885023	4.01175	795.5015	13.64497	37.70419	3.40125	3.898562	9.633216	2.5
221.538652	4.252181	632.9322	12.14841	32.6384	2.856983	3.470974	7.896229	2.5
258.547896	3.774422	808.5487	11.80363	33.9577	3.127269	3.372466	8.029209	2.5
197.480448	4.624835	545.7116	12.78013	32.66948	2.76337	3.651466	8.155296	2.5
236.679678	4.004732	871.4196	14.74483	40.88243	3.681852	4.21281	10.7401	2.5
181.555674	5.071388	566.8223	15.83303	38.72886	3.12203	4.523722	10.76164	2.5
214.926942	4.272901	907.0112	18.03203	48.0216	4.220091	5.152009	13.75913	2.5
244.924398	3.969601	989.2871	16.03383	44.93745	4.039153	4.581093	12.06422	2.5
207.505806	4.424431	942.5392	20.09679	52.59201	4.542231	5.741939	15.67235	2.5
232.781036	4.083878	811.0979	14.22979	39.09914	3.484381	4.065654	10.14591	2.5
216.5016	4.28716	728.7854	14.43139	38.47079	3.366189	4.123255	10.14423	2.5
218.705911	4.263273	879.2151	17.1387	45.86772	4.020079	4.89677	12.87542	2.5
266.805191	3.675003	950.9427	13.09838	38.23419	3.564184	3.742396	9.423382	2.5
293.345666	3.504727	932.4449	11.14032	33.69606	3.178656	3.182949	7.635594	2.5
266.344486	3.761928	1312.322	18.53563	53.76697	4.927161	5.295893	14.7737	2.5
203.63931	4.525318	801.3135	17.80697	46.1204	3.934965	5.087705	13.28165	2.5
226.961985	4.141642	820.2918	14.96883	40.72705	3.614226	4.276808	10.82719	2.5
303.297821	3.454417	1184.663	13.49275	41.30228	3.905941	3.855071	10.03833	2.5
250.242381	3.903937	875.0361	13.65111	38.62624	3.496754	3.900317	9.747172	2.5
233.65109	4.084809	683.0588	11.94159	32.8406	2.923414	3.411882	7.856777	2.5
227.251827	4.109436	879.6783	15.90738	43.37905	3.870941	4.544967	11.79795	2.5
257.364764	3.824142	1108.513	16.47122	47.17691	4.307167	4.706062	12.64708	2.5
244.976809	3.919629	959.1556	15.34649	43.14983	3.915292	4.384711	11.42686	2.5
229.457886	4.141839	884.6137	15.96776	43.56336	3.855233	4.562216	11.82592	2.5
227.849222	2.848115	1128.195	14.10244	42.17614	4.9515	4.029269	11.25433	2.5
198.578523	3.718699	686.8649	12.86264	34.77085	3.458908	3.675039	9.143939	2.5
199.532981	3.661156	943.1282	17.3051	47.019	4.726678	4.944315	13.64395	2.5
217.193754	3.098342	974.7375	13.90496	40.23455	4.487871	3.972845	10.80662	2.5

214.315667	3.088122	971.6794	14.00114	40.41135	4.53387	4.000327	10.91302	2.5
179.827614	3.892373	719.7939	15.57996	40.61876	4.002689	4.451416	11.68758	2.5
212.93884	3.374625	934.355	14.80753	41.73399	4.387903	4.230722	11.4329	2.5
189.14276	3.568731	635.1793	11.98452	32.33623	3.3582	3.424147	8.415784	2.5
177.789267	3.995264	641.6038	14.41806	37.23891	3.608788	4.119447	10.4228	2.5
208.521253	3.098384	846.09	12.57192	36.00852	4.057572	3.591976	9.473533	2.5
187.055277	3.921494	774.2982	16.23267	42.65986	4.139408	4.637905	12.31117	2.5
207.207927	3.315327	970.9697	15.53552	43.68131	4.685968	4.438719	12.22019	2.5
174.371317	4.305465	658.7934	16.2665	41.03533	3.778106	4.647572	11.96104	2.5
208.174322	3.202682	955.6296	14.70199	41.74501	4.590526	4.20057	11.49931	2.5
239.031922	2.575775	1225.509	13.20591	40.98788	5.126967	3.773118	10.63014	2.5
207.815108	3.362704	792.8713	12.82963	35.97173	3.815272	3.665609	9.466928	2.5
191.015172	3.673369	827.8422	15.92004	42.75088	4.333908	4.548584	12.24667	2.5
199.609807	3.603065	771.0769	13.91836	37.97217	3.862921	3.976673	10.31529	2.5
187.570572	3.81241	835.6148	16.98404	44.98133	4.454936	4.852583	13.17163	2.5
174.138411	4.216426	757.4189	18.33944	46.4915	4.349522	5.23984	14.12301	2.5
213.440116	3.224171	879.2544	13.28179	37.88533	4.119443	3.794797	10.05762	2.5
267.664488	2.457892	1231.605	11.3095	36.53428	4.601302	3.231287	8.851612	2.5
190.79559	3.655088	750.4053	14.37558	38.64052	3.933033	4.107309	10.72049	2.5
204.099362	3.453458	912.2086	15.435	42.79603	4.469434	4.41	11.98154	2.5
182.939996	4.148299	773.9273	17.54937	45.22424	4.230498	5.014107	13.40107	2.5
225.809834	2.829697	962.7972	12.06513	36.06057	4.263752	3.447179	9.23543	2.5
236.569124	2.66107	1237.499	13.92012	42.74335	5.231025	3.977178	11.25905	2.5
200.797518	3.637636	781.725	14.16169	38.6011	3.893101	4.046196	10.52405	2.5
168.87331	4.31901	670.7446	17.15459	42.89677	3.971881	4.901312	12.83558	2.5
210.565499	3.480421	660.3316	10.91457	30.44006	3.135992	3.118449	7.434151	2.5
184.574644	3.626221	820.6814	16.12341	43.06617	4.446339	4.606688	12.49719	2.5
189.158861	3.908241	865.3223	17.87856	47.15675	4.57458	5.10816	13.97032	2.5
191.455029	3.783696	703.3838	13.90087	37.07486	3.673885	3.971676	10.11717	2.5
215.154713	3.100212	871.6403	12.55966	36.25081	4.051226	3.588474	9.459446	2.5
215.822965	2.928398	1086.877	14.74731	43.20954	5.035964	4.213517	11.81891	2.5
194.832658	3.424124	963.5302	16.93375	46.50837	4.945425	4.838213	13.50962	2.5
193.918521	3.624645	747.1592	13.96559	37.77007	3.852954	3.990169	10.34095	2.5
236.18927	4.051274	850.708	14.5919	40.32079	3.601806	4.169115	10.54063	2.5
261.945997	3.769007	873.2435	12.56466	36.27829	3.333678	3.589902	8.795648	2.5
269.185611	3.707791	1099.368	15.1428	44.20185	4.084051	4.326516	11.43501	2.5
264.402063	3.734492	1018.573	14.38662	41.7318	3.852363	4.110463	10.65213	2.5
302.331328	3.475073	1073.77	12.34219	37.69398	3.551635	3.52634	8.867116	2.5
223.622762	4.211351	594.6637	11.19894	30.23086	2.659227	3.199697	6.987588	2.5
276.241953	3.629986	908.1428	11.93354	35.24647	3.28749	3.409584	8.303559	2.5
223.184765	4.203103	677.9809	12.768	34.46645	3.037756	3.648001	8.564899	2.5
301.396982	3.488391	988.5086	11.44107	34.8815	3.279756	3.268878	7.95268	2.5
243.600274	3.967431	717.2015	11.68081	32.69753	2.944173	3.337373	7.713374	2.5
267.237775	3.737591	942.3917	13.1803	38.32675	3.526417	3.765801	9.442713	2.5
266.892024	3.727542	840.1891	11.73448	34.1344	3.148049	3.35271	8.006942	2.5
233.517786	4.082479	756.0037	13.21685	36.34769	3.237456	3.776242	9.134368	2.5
240.317654	3.939634	993.7371	16.29077	45.52753	4.135098	4.654506	12.35114	2.5

297.554943	3.476109	963.42	11.25491	34.23421	3.237789	3.215688	7.778798	2.5
232.051448	4.056843	789.8334	13.80828	37.97419	3.4037	3.945222	9.751433	2.5
243.408091	3.919615	962.4031	15.49763	43.50491	3.953867	4.427896	11.57802	2.5
261.108089	3.756951	934.5769	13.44715	38.82634	3.579272	3.842043	9.690198	2.5
252.502073	3.849117	847.8005	12.92379	36.78034	3.357598	3.692511	9.074671	2.5
286.368468	3.575137	912.554	11.39268	34.08271	3.186643	3.255052	7.817547	2.5
248.776042	3.881062	794.7297	12.39828	35.08132	3.194559	3.542366	8.517218	2.5
274.25306	3.651838	602.488	8.022476	23.61666	2.196832	2.292136	4.370638	2.5
267.006284	3.729138	1009.968	14.10569	41.032	3.782561	4.030198	10.37656	2.5
267.925779	3.685361	822.1235	11.30844	33.0207	3.068475	3.230982	7.623077	2.5
249.417576	3.878967	995.0353	15.47489	43.8208	3.989435	4.421397	11.59592	2.5
289.829113	3.525902	872.5685	10.61519	31.96286	3.010631	3.032911	7.089287	2.5
319.101172	3.383894	1071.445	11.36209	35.40675	3.357696	3.246311	7.978194	2.5
244.900635	3.91841	1050.934	16.81495	47.2787	4.291268	4.804271	12.89654	2.5
242.642165	3.938996	910.2002	14.77598	41.39533	3.751204	4.221708	10.83698	2.5
227.587445	4.145491	649.4029	11.82883	32.19845	2.853422	3.379667	7.683342	2.5
268.822566	3.733647	979.632	13.606	39.63361	3.644158	3.887429	9.872354	2.5
259.159032	3.822405	749.4966	11.05452	31.72104	2.892033	3.158435	7.232117	2.5
232.760244	4.033973	732.9367	12.70254	35.0094	3.148891	3.629298	8.668569	2.5
302.994348	3.427538	930.6199	10.52737	32.27997	3.07141	3.007821	7.099836	2.5
262.253661	3.778871	762.9592	10.99365	31.73085	2.909242	3.141043	7.214778	2.5
220.033602	3.138853	1008.879	14.392	41.64383	4.585114	4.112	11.25315	2.5
218.629432	3.312567	889.6015	13.47881	38.41825	4.068993	3.851089	10.16624	2.5
217.541379	3.046798	931.5302	13.04664	37.9248	4.282083	3.727612	9.999843	2.5
190.679831	3.695346	801.3026	15.52912	41.62069	4.202346	4.436891	11.83377	2.5
195.261619	3.712198	533.954	10.15122	27.33786	2.734557	2.900347	6.439018	2.5
214.912993	3.342348	859.0262	13.35966	37.83103	3.997088	3.817046	10.01731	2.5
191.287012	3.758095	746.1813	14.65975	39.15669	3.900847	4.1885	10.90166	2.5
205.981976	3.32229	760.7439	12.27006	34.43061	3.693255	3.505732	8.947774	2.5
204.795652	3.401921	768.7229	12.76948	35.56906	3.753609	3.648424	9.367562	2.5
195.401252	3.489308	786.0608	14.0368	38.39857	4.022803	4.010514	10.54749	2.5
204.047798	3.458437	717.2604	12.15696	33.69286	3.515159	3.473416	8.698518	2.5
202.80644	3.265804	872.6918	14.05301	39.44956	4.303077	4.015145	10.7872	2.5
197.903677	3.377196	675.7073	11.53084	31.90329	3.414324	3.294526	8.153646	2.5
223.733941	2.928455	1016.741	13.30812	39.34506	4.544419	3.802321	10.37967	2.5
213.570241	3.300931	728.1926	11.25491	31.92038	3.409616	3.215688	7.953978	2.5
206.600399	3.243334	812.935	12.76193	36.05382	3.934818	3.646266	9.518597	2.5
192.450503	3.97625	598.5924	12.36761	32.62099	3.110371	3.533603	8.391362	2.5
193.029299	3.755434	829.688	16.14179	43.22077	4.298249	4.61194	12.38636	2.5
218.79502	3.161778	674.316	9.744451	28.10502	3.081953	2.784129	6.582673	2.5
246.681953	2.479216	1059.559	10.64883	33.63239	4.295242	3.042523	8.169616	2.5
214.49458	2.979091	805.85	11.19236	32.6028	3.756971	3.197817	8.21327	2.5
217.424232	2.986597	740.4385	10.17086	29.70917	3.405501	2.90596	7.184262	2.5
185.260038	3.773117	695.4344	14.16363	37.49256	3.753828	4.046752	10.39052	2.5
228.676678	2.927998	832.7595	10.66273	31.69796	3.641646	3.046495	7.734735	2.5
205.58244	3.364688	993.5177	16.26052	45.4616	4.832697	4.645862	12.89583	2.5
216.666175	3.200387	748.7596	11.05996	31.72494	3.455821	3.15999	7.859577	2.5

210.396441	3.37715	640.8684	10.28681	28.90033	3.046004	2.939089	6.909662	2.5
187.301162	4.002162	559.8561	11.96274	31.28902	2.989069	3.417925	7.960575	2.5
213.183683	3.346683	599.1194	9.405328	26.57106	2.810344	2.687237	6.058646	2.5
194.910872	3.493026	812.9764	14.56947	39.8201	4.171016	4.162706	11.07644	2.5
195.4177	3.839248	652.2643	12.81462	34.2283	3.337795	3.661321	8.975375	2.5
195.130453	3.509541	598.6261	10.76666	29.40015	3.067825	3.076188	7.257116	2.5
196.318165	3.543649	685.1776	12.36783	33.742	3.490139	3.533665	8.824178	2.5
222.18528	2.927342	1125.719	14.8316	43.77725	5.066576	4.237601	11.90426	2.5
212.020886	3.251854	744.1108	11.41274	32.43037	3.509611	3.260783	8.160888	2.5
201.737958	3.484248	483.6357	8.352948	23.04143	2.397346	2.386557	4.8687	2.5
221.581569	3.094715	868.1616	12.12516	35.27084	3.918023	3.464332	9.030447	2.5
202.922546	3.26242	712.9472	11.46217	32.18953	3.513396	3.274907	8.199754	2.5
187.452731	3.632805	614.3826	11.90664	31.91183	3.277534	3.401897	8.273835	2.5
209.076869	3.144013	929.8597	13.98285	39.93017	4.447454	3.995101	10.83884	2.5
197.156517	3.495683	697.9687	12.37533	33.91381	3.540176	3.535809	8.879649	2.5
204.58662	3.206687	814.7416	12.77024	36.09146	3.98238	3.648641	9.563558	2.5
219.003595	4.38885	711.183	14.25216	37.87965	3.247358	4.072047	9.863315	2.9
178.592128	4.31382	656.537	15.85838	40.22619	3.676181	4.530966	11.54456	2.8
134.625146	6.631781	456	22.46305	47.68072	3.387183	6.418015	15.83127	3.1
150.309685	5.871472	477.714	18.6607	41.97474	3.178198	5.331629	12.78923	3.1
137.827949	6.351518	563.162	25.95217	56.01296	4.085978	7.414905	19.60065	3
142.368326	6.189927	444.502	19.32617	42.32314	3.122197	5.521764	13.13625	3
181.734546	4.992707	550.075	15.11195	37.11897	3.026805	4.3177	10.11924	3
172.595718	5.183055	594.5	17.85285	42.88631	3.444466	5.100815	12.6698	3.1
184.419618	4.917856	682.559	18.20157	45.0419	3.701119	5.20045	13.28372	3.2
297.821356	3.798742	1422.797	18.14792	54.00151	4.77735	5.18512	14.34918	3.2
136.129308	6.576295	359.775	17.38043	37.07262	2.642892	4.965839	10.80414	3
138.288369	6.402239	434.714	20.12565	43.38737	3.143533	5.750185	13.72341	3.1
163.890945	5.426852	543.857	18.00851	42.21624	3.318408	5.145289	12.58166	3.1
189.383273	4.153142	759.419	16.65393	43.27707	4.009958	4.758264	12.50078	3
146.162813	5.092781	585.809	20.41146	47.24378	4.007921	5.831847	15.31868	3
220.870941	3.827919	995.444	17.25206	47.5483	4.506903	4.929161	13.42414	3.1
197.481586	4.030236	872.875	17.81378	47.13082	4.420032	5.08965	13.78354	3.1
197.481586	4.030236	840.625	17.15561	45.38948	4.256726	4.901603	13.12538	3.1
190.445592	4.714	761.75	18.8552	47.53642	3.99983	5.387199	14.1412	3.1
220.238457	4.06344	988.75	18.24262	49.49793	4.489452	5.212177	14.17918	3.1
271.41857	3.618914	1055.545	14.07393	41.41721	3.888993	4.021124	10.45502	3.1
220.889429	4.183512	759.908	14.3922	38.79588	3.440219	4.112056	10.20869	3
293.69089	3.459257	1240.493	14.61122	44.35203	4.223805	4.174636	11.15197	3
317.918124	3.357108	1379.676	14.56891	45.44798	4.339721	4.162546	11.2118	3.2
179.299342	4.002217	795.94	17.76652	45.96442	4.439169	5.076148	13.7643	3.2
165.853508	4.364566	652	17.15789	42.60003	3.93118	4.902256	12.79333	3.1
207.703998	2.841368	1209.375	16.54412	48.37522	5.822589	4.726891	13.70275	3.1
186.423182	3.958029	762.592	16.19091	42.41569	4.09065	4.625975	12.23288	2.8
313.051852	3.373404	1264.808	13.6294	42.30227	4.040251	3.894113	10.25599	3
268.55718	3.699135	1035.057	14.25698	41.61614	3.85414	4.073424	10.55785	3
240.736608	3.946502	855.606	14.02633	39.19913	3.554117	4.007522	10.07983	3

237.698581	3.915957	1151.039	18.96275	52.92959	4.842431	5.417929	15.04679	3.2
242.478256	2.604493	1124.388	12.07721	37.51491	4.637067	3.450631	9.472715	3.2
220.286218	2.820566	1320.455	16.90723	50.26149	5.99427	4.830638	14.08667	3.1
214.842899	2.971548	938.375	12.97891	37.84624	4.367726	3.708259	10.00736	3
241.960879	2.557726	1291.571	13.65297	42.57944	5.337933	3.900849	11.09524	3.1
280.092904	3.656565	1076.222	14.0499	41.56524	3.842375	4.014256	10.39333	2.9
215.370824	2.902572	867.344	11.68927	34.30745	4.027212	3.339792	8.7867	2.8
380.344896	3.104856	2419.8	19.75347	65.71695	6.36212	5.643848	16.64861	3.1
189.675544	3.959823	693.897	14.48637	38.11039	3.658337	4.138962	10.52654	3.2
279.473384	2.136647	2075.235	15.86571	53.65516	7.425519	4.53306	13.72906	3.1
112.862778	7.28147	527.67	34.04323	67.54818	4.675324	9.726636	26.76176	3.1
148.751155	5.934143	576.17	22.9852	51.43087	3.873382	6.5672	17.05106	2.8
148.751155	5.934143	530.5	21.16328	47.35421	3.566359	6.046652	15.22914	2.8
142.831037	6.15651	648	27.93103	61.29979	9.956906	7.980296	21.77452	3
142.831037	6.15651	636.5	27.43534	60.21191	27.43534	7.83867	21.27883	3
142.831037	6.15651	687.67	29.64095	65.05251	29.64095	8.468842	23.48444	3
262.896153	3.957313	627.42	9.444403	26.96314	2.38657	2.698401	5.48709	2.9
168.282175	5.242435	680.58	21.20187	50.46618	4.044279	6.057677	15.95943	2.8
131.318409	6.734277	532.75	27.32051	57.41132	4.056933	7.805861	20.58624	2.9
131.318409	6.734277	574	29.4359	61.85658	4.371055	8.410256	22.70162	3
180.506763	5.003652	630.92	17.48912	42.86171	3.495271	4.996891	12.48547	2.8
161.655752	5.485993	630.58	21.39953	49.85839	3.900758	6.114152	15.91354	3
193.014982	4.699087	707.75	17.23068	43.62111	3.666814	4.92305	12.53159	2.9
153.558465	5.666364	674.42	24.88635	56.78106	4.391943	7.110385	19.21998	3
177.151759	5.07962	780	22.36559	54.35118	4.403005	6.390169	17.28597	3
181.385575	5.080828	732.75	20.52521	50.17122	4.039737	5.864346	15.44438	3
171.691839	5.210678	688.08	20.88255	50.03194	4.007645	5.966443	15.67187	2.8
143.235541	6.101621	732.25	31.19276	68.66022	5.112209	8.912217	25.09114	3
153.966176	5.745007	523.33	19.52724	44.42982	3.398993	5.579211	13.78223	2.8
153.966176	5.745007	627.17	23.40187	53.24566	4.073427	6.686247	17.65686	3
167.739245	5.299818	672.92	21.2613	50.42932	4.011703	6.074656	15.96148	2.8
167.739245	5.299818	746.75	23.594	55.96222	4.45185	6.741142	18.29418	2.8
158.556237	5.550329	697.92	24.43099	56.48162	4.401719	6.980282	18.88066	2.9
141.069075	6.241994	573.67	25.38363	55.34531	4.066589	7.252465	19.14163	2.9
141.069075	6.241994	617.67	27.33053	59.59025	4.378493	7.808723	21.08854	3
150.975432	5.139589	572.75	19.49787	45.39225	3.793664	5.570821	14.35828	2.8
150.975432	5.139589	625	21.2766	49.53323	4.139746	6.079027	16.13701	3
133.293582	5.884926	553.08	24.41854	53.2705	4.149337	6.976727	18.53362	2.8
158.210113	4.870551	742.17	22.84795	54.5458	4.69104	6.527986	17.9774	3
127.963273	5.993596	528.83	24.76956	53.24363	4.13267	7.077016	18.77596	2.9
127.963273	5.993596	592.17	27.7363	59.62083	4.627656	7.924657	21.7427	2.9
184.453728	4.34776	688.75	16.23453	41.43289	3.733999	4.638438	11.88677	2.9
135.553177	5.695512	629.58	26.45294	58.42762	4.644524	7.557983	20.75743	2.8
135.553177	5.695512	656.58	27.58739	60.93333	4.843708	7.882113	21.89188	2.8
131.032932	6.08974	510.42	23.72171	51.09065	3.895357	6.777631	17.63197	3
131.032932	6.08974	511.08	23.75238	51.15671	3.900394	6.786395	17.66264	3
191.621382	4.158179	771.25	16.73611	43.60533	4.024864	4.781745	12.57793	2.8

137.42327	5.655279	609.75	25.09259	55.71179	4.437021	7.169312	19.43731	2.8
137.42327	5.655279	634.58	26.1144	57.98046	4.617704	7.461258	20.45912	2.9
125.652752	6.293021	582.67	29.18165	61.6862	4.637145	8.337614	22.88863	2.9
125.652752	6.293021	606.58	30.37913	64.21751	4.827431	8.67975	24.0861	3
129.838585	6.006318	514	23.77758	51.27038	3.958762	6.793595	17.77126	2.8
129.838585	6.006318	499	23.08368	49.77416	3.843234	6.595338	17.07737	2.8
134.610211	5.679756	555.25	23.42827	51.69247	4.124873	6.693791	17.74851	3
120.467547	6.442115	604.5	32.3262	67.22257	5.017949	9.236058	25.88409	3
152.726621	4.830522	691.75	21.87905	51.88104	4.529335	6.251158	17.04853	2.8
152.726621	4.830522	815.17	25.78265	61.1375	5.337445	7.366471	20.95213	2.9
160.485403	4.831424	783.92	23.59996	56.65672	4.884681	6.742847	18.76854	2.9
168.993372	5.236046	744.83	23.07761	55.00567	4.407451	6.593604	17.84157	3.1
142.840843	6.130508	616.33	26.45193	58.11609	4.314802	7.557695	20.32142	3.1
142.840843	6.130508	696.33	29.88541	65.65959	4.874866	8.538688	23.7549	3.1
168.282175	5.242435	1045.25	32.56231	77.50708	6.211294	9.303516	27.31987	3.1
131.318409	6.734277	584	29.94872	62.93423	4.447206	8.556777	23.21444	3.2
181.385575	5.080828	751.92	21.06218	51.48379	4.145423	6.017767	15.98136	3.1
181.385575	5.080828	804.5	22.53501	55.08393	4.435303	6.438575	17.45419	3.2
143.235541	6.101621	665.25	28.33866	62.37789	4.644448	8.096759	22.23704	3
168.648329	5.286781	697.5	21.8652	51.96384	4.135825	6.247201	16.57842	3.1
168.648329	5.286781	871.17	27.3094	64.90227	5.165601	7.802687	22.02262	3.1
153.900316	5.789426	666.83	25.08483	56.95897	4.33287	7.167094	19.2954	3.1
153.900316	5.789426	672.92	25.31392	57.47916	4.372441	7.232549	19.5245	3.1
234.579329	4.212764	1221	21.9277	59.89957	5.205062	6.265057	17.71493	3.1
234.579329	4.212764	1433.17	25.73802	70.30816	6.109532	7.353719	21.52525	3.2
156.892622	5.722458	612.42	22.33724	51.11336	3.903434	6.382067	16.61478	3.1
156.892622	5.722458	736.5	26.8629	61.46923	4.694293	7.675113	21.14044	3.2
174.368471	5.166473	719.17	21.30874	51.36014	4.124427	6.088212	16.14227	3.1
174.368471	5.166473	801.08	23.7357	57.20982	4.594179	6.78163	18.56923	3.1
150.975432	5.139589	571.5	19.45532	45.29318	3.785384	5.558663	14.31573	3
156.653677	4.926216	748.5	23.53774	55.89481	4.778056	6.725067	18.61152	3
137.841503	5.580628	622.42	25.19919	56.1773	4.515476	7.199769	19.61856	3.2
137.841503	5.580628	690.33	27.94858	62.3066	5.008143	7.985309	22.36796	3.2
158.210113	4.870551	752.5	23.16596	55.305	4.756333	6.618847	18.29541	3
163.107795	4.769234	695.08	20.32398	49.14906	4.261476	5.80685	15.55474	3.1
154.513865	4.997699	741.75	23.99166	56.57304	4.80054	6.854759	18.99396	3
154.513865	4.997699	742.42	24.01333	56.62414	4.804876	6.86095	19.01563	3.1
155.498682	4.923493	793.08	25.11098	59.52878	5.100236	7.174565	20.18748	3.2
155.498682	4.923493	783.25	24.79973	58.79094	5.03702	7.085638	19.87624	3.2
156.723468	4.892563	748.5	23.36653	55.58961	4.775928	6.676151	18.47397	3.2
175.50344	4.394948	949.58	23.77933	59.77679	5.410606	6.794094	19.38438	3.1
175.50344	4.394948	986.25	24.69762	62.0852	5.619548	7.056462	20.30267	3.2
134.610211	5.679756	559.08	23.58987	52.04904	4.153325	6.739964	17.91012	3
148.2506	5.180508	567.75	19.83961	45.88694	3.829664	5.668459	14.6591	3.2
148.2506	5.180508	692.42	24.19611	55.96307	4.670605	6.913173	19.0156	3.2
120.467547	6.442115	627.75	33.56952	69.80805	5.210947	9.591291	27.1274	3.1
139.437492	5.551519	590.58	23.51316	52.63843	4.235446	6.718045	17.96164	3

139.437492	5.551519	630.17	25.08938	56.16709	4.519373	7.168395	19.53786	3
160.485403	4.831424	715.67	21.54529	51.72405	4.459409	6.155798	16.71387	3.1
160.485403	4.831424	774.92	23.32902	56.00626	4.828601	6.665434	18.49759	3.1
163.107795	4.769234	751.92	21.98596	53.16821	4.609957	6.281704	17.21673	3.1
171.920178	5.225537	594.67	18.07508	43.28915	3.458989	5.164307	12.84954	3
171.920178	5.225537	725.75	22.05927	52.83116	4.221436	6.302649	16.83373	3
178.664054	5.004595	626.75	17.55602	42.91343	3.50798	5.016006	12.55143	2.9
178.664054	5.004595	735.5	20.60224	50.35951	4.116665	5.886355	15.59765	2.9
178.664054	5.004595	740.58	20.74454	50.70734	4.145098	5.927011	15.73994	2.9
212.442665	4.310668	1063.83	21.58615	57.19386	5.00761	6.16747	17.27548	2.9
280.232835	3.548733	1159.83	14.68753	43.78346	4.138808	4.196436	11.13879	2.9
185.792642	4.882855	635.25	16.69514	41.46471	3.419134	4.770039	11.81228	3
202.898097	4.447861	794.08	17.40755	45.23965	3.913689	4.973584	12.95968	2.8
202.898097	4.447861	826	18.10728	47.05817	4.071009	5.17351	13.65942	2.8
219.887644	4.16983	651.75	12.35943	33.30575	2.964014	3.531267	8.189603	2.8
219.887644	4.16983	1020.08	19.34424	52.12816	4.639097	5.526927	15.17441	3
193.056639	4.739563	769.33	18.88714	47.71477	3.984996	5.396327	14.14758	3
183.665118	4.795434	917.5	23.95561	59.59461	4.995505	6.844461	19.16018	2.9
183.665118	4.795434	913.67	23.85561	59.34584	4.974652	6.81589	19.06018	2.9
194.730435	4.555098	876.42	20.50105	52.42159	4.500683	5.857444	15.94595	3
194.730435	4.555098	762.33	17.83228	45.59749	3.914796	5.094937	13.27718	2.8
162.17521	4.433076	665.75	18.19834	44.75604	4.105128	5.199527	13.76527	2.8
150.402986	5.469199	588.83	21.412	49.03322	3.915015	6.117714	15.9428	2.9
160.096518	4.833102	700.42	21.14475	50.72729	4.374986	6.041358	16.31165	2.9
160.096518	4.833102	721.42	21.77872	52.24819	4.506157	6.222491	16.94561	3
172.576349	4.102416	850.92	20.22773	51.51487	4.930687	5.779352	16.12532	2.9
162.597531	4.529179	639.08	17.80167	43.5747	3.930441	5.086192	13.27249	2.9
190.674529	3.37776	923.33	16.3566	44.83415	4.84244	4.673314	12.97884	3
190.674529	3.37776	942.08	16.68875	45.7446	4.940775	4.768215	13.31099	3
161.831819	4.205281	702.92	18.26573	45.49401	4.343522	5.218779	14.06045	3
186.159241	3.763302	777.67	15.72099	41.69257	4.177445	4.49171	11.95768	2.8
187.035117	3.828764	805.42	16.48762	43.58871	4.30625	4.710747	12.65885	3
157.020172	4.557915	645.5	18.7373	45.39461	4.110937	5.353514	14.17939	2.9
185.975968	3.574193	815.5	15.67275	42.09348	4.384975	4.477927	12.09855	2.8
185.975968	3.574193	1073	20.62153	55.3848	5.769563	5.891865	17.04734	2.8
183.684359	3.909592	747.08	15.90107	41.63049	4.067194	4.543163	11.99148	2.8
183.684359	3.909592	908.5	19.33678	50.62551	4.945985	5.524795	15.42719	2.9
168.865864	5.408034	617	19.75981	46.70983	3.653788	5.645659	14.35177	3.1
168.865864	5.408034	688.5	22.04964	52.12272	4.077201	6.299897	16.64161	3.1
173.206348	5.268634	646	19.65019	47.05255	3.729655	5.61434	14.38156	3.2
173.206348	5.268634	761.92	23.17627	55.49579	4.398915	6.621793	17.90764	3.2
176.811435	5.110157	829	23.95954	58.10954	4.688611	6.845582	18.84938	3.2
176.811435	5.110157	917.67	26.52225	64.32495	5.190105	7.577787	21.4121	3.2
195.390398	4.663255	810.83	19.35155	49.23447	4.149795	5.529015	14.6883	3.2
280.232835	3.548733	1176.08	14.89331	44.3969	4.196796	4.255231	11.34458	3
280.232835	3.548733	1434.25	18.16265	54.14279	5.118065	5.189329	14.61392	3.2
195.031801	4.663936	949	22.69412	57.71006	4.865873	6.484034	18.03018	3.2

180.118425	5.014852	810.08	22.55422	55.21447	4.497485	6.444063	17.53937	3.1
275.926731	3.593966	1197.25	15.59427	46.16044	4.339014	4.455505	12.0003	3.2
275.926731	3.593966	1148.83	14.96359	44.29359	4.163533	4.275313	11.36963	3.2
160.441495	5.736815	745.33	26.65034	61.28641	4.645494	7.614382	20.91352	3.1
219.887644	4.16983	1016.08	19.26839	51.92375	4.620905	5.505254	15.09856	3.1
173.552921	5.165266	654.5	19.47917	46.89809	3.771184	5.565476	14.3139	3.2
185.804266	4.93727	777.75	20.6667	51.18744	4.185857	5.904772	15.72943	3.2
185.804266	4.93727	964.75	25.63575	63.4948	5.192292	7.324499	20.69848	3.2
193.056639	4.739563	799.92	19.63813	49.612	4.143447	5.610895	14.89857	3.1
156.648556	5.922441	732.08	27.67788	62.76814	4.673391	7.907967	21.75544	3.1
156.648556	5.922441	757.75	28.64839	64.96908	4.837261	8.185255	22.72595	3.1
148.471382	5.027304	636.42	21.54945	50.23578	4.286483	6.156987	16.52215	3.2
162.17521	4.433076	690.08	18.86341	46.39166	4.255151	5.389545	14.43033	3.2
162.17521	4.433076	733.25	20.04346	49.29383	4.521345	5.726704	15.61039	3.2
165.895044	4.886452	622.08	18.32342	44.22995	3.749841	5.235262	13.43696	3.1
186.976717	3.792631	866.58	17.57769	46.57721	4.634695	5.022196	13.78506	3.1
196.524127	3.810453	893.25	17.31944	46.41342	4.545243	4.948411	13.50898	3.1
150.402986	5.469199	645	23.45455	53.71062	4.288479	6.701299	17.98535	3.2
179.79542	4.152319	912.25	21.06813	54.04406	5.073822	6.019466	16.91581	3.2
225.114198	3.794081	1039.25	17.51555	48.61248	4.616546	5.004442	13.72147	3.2
225.114198	3.794081	1172.17	19.75579	54.83002	5.207002	5.64451	15.9617	3.2
161.21654	4.528554	719.5	20.21067	49.36775	4.462942	5.774478	15.68212	3.2
204.958319	3.354473	1036.58	16.9653	47.43205	5.057516	4.847229	13.61083	3.2
192.807635	3.531275	926.58	16.97033	46.13053	4.805723	4.848666	13.43905	3.1
192.807635	3.531275	935.92	17.14139	46.59553	4.854165	4.897541	13.61012	3.2
149.333039	5.295498	562.08	19.93191	45.93157	3.763936	5.694833	14.63642	3.2
149.333039	5.295498	622	22.05674	50.82806	4.165187	6.301925	16.76124	3.2
184.547821	3.68234	712.92	14.22511	37.84879	3.863064	4.064318	10.54277	3.1
184.547821	3.68234	845.58	16.87212	44.89168	4.581902	4.820605	13.18978	3.2
174.833487	4.170945	866.67	20.67586	52.60913	4.957117	5.907388	16.50491	3.2
174.833487	4.170945	949.33	22.64785	57.62681	5.429909	6.470815	18.47691	3.2
231.778553	4.105909	781.5	13.84411	37.94731	3.371753	3.95546	9.738201	2.8
175.581306	5.210128	612.67	18.18012	43.80303	3.489381	5.19432	12.96999	2.9
231.778553	4.105909	943	16.70505	45.78927	4.068539	4.772871	12.59914	2.8
280.265251	3.600067	1229.42	15.79216	46.90901	4.38663	4.512047	12.1921	2.9
344.174438	3.231685	1488.92	13.98047	44.91167	4.326062	3.99442	10.74878	3
344.174438	3.231685	1508.33	14.16272	45.49715	4.382458	4.046492	10.93104	3
248.234535	3.876666	1086.25	16.96391	47.98736	4.375902	4.846831	13.08724	2.9
205.251048	4.474137	886.33	19.32054	50.2821	4.318273	5.520156	14.84641	3
230.807695	4.068315	846.33	14.91777	40.94142	3.666819	4.262221	10.84946	2.9
230.807695	4.068315	966.5	17.03594	46.75468	4.187469	4.867412	12.96763	3
208.64483	4.397151	902.17	19.01307	49.90123	4.323951	5.432305	14.61592	2.8
181.333206	4.100984	619.9	14.01949	36.15178	3.418569	4.00557	9.918511	2.8
197.639072	3.665073	911.83	16.90923	45.82171	4.613612	4.831207	13.24415	2.9
180.877921	3.940695	812.33	17.69782	46.0652	4.49104	5.05652	13.75713	2.9
180.877921	3.940695	786.92	17.14423	44.62426	4.350559	4.89835	13.20353	2.8
187.620576	3.804379	712.83	14.45404	38.3035	3.799317	4.129726	10.64966	2.9

198.747033	3.374313	726.08	12.32733	34.15057	3.653287	3.522096	8.953022	3
199.979126	3.341339	1034.08	17.27786	48.05693	5.17094	4.936532	13.93652	2.9
236.505698	2.726825	1111.75	12.81807	39.1173	4.700732	3.662307	10.09125	3
236.505698	2.726825	1384.92	15.96762	48.72888	5.855757	4.562179	13.2408	3
211.056254	4.406185	1040.08	21.71357	57.12354	4.927975	6.203877	17.30739	3.1
201.012197	4.578866	808	18.40547	47.3765	4.019657	5.258705	13.8266	3.2
267.453193	3.751097	1060.25	14.87027	43.21068	3.964245	4.248648	11.11917	3
344.174438	3.231685	1388.25	13.03521	41.87506	4.033565	3.724346	9.803526	3
208.64483	4.397151	1136.17	23.94457	62.84434	5.445474	6.841307	19.54742	3.2
218.099251	3.238296	1013.92	15.05449	43.12712	4.648893	4.301283	11.8162	3
208.090192	3.429587	1112.42	18.33407	51.16953	5.345855	5.238307	14.90449	3.1
186.17938	4.13732	943.58	20.96844	54.30869	5.068123	5.990984	16.83112	3.2
190.80057	3.840978	924	18.60091	49.38199	4.842753	5.314545	14.75993	3.1
187.620576	3.804379	810.67	16.43794	43.56088	4.320795	4.696555	12.63356	3
187.620576	3.804379	932.5	18.90829	50.10734	4.970137	5.402368	15.10391	3.1
198.747033	3.374313	734.92	12.47742	34.56636	3.697766	3.564977	9.103106	3.1
228.518147	2.857084	1120.25	14.0061	41.88579	4.902236	4.001743	11.14902	3.2
238.566192	3.996084	988.67	16.56064	46.03314	4.144217	4.73161	12.56455	2.8
197.516304	3.649262	752.83	13.9091	37.72667	3.811483	3.974028	10.25984	2.9
205.473961	3.308759	872	14.04187	39.41829	4.243847	4.011962	10.73311	3
214.298455	3.126938	1011.17	14.7545	42.45212	4.718513	4.215571	11.62756	2.8
214.298455	3.126938	1061	15.48159	44.54414	4.951039	4.423312	12.35465	2.9
205.473961	3.308759	855.25	13.77214	38.66112	4.162328	3.934898	10.46338	3
214.298455	3.126938	1025.83	14.96841	43.06759	4.786922	4.276688	11.84147	3
165.921881	5.36964	549.3308	17.7777	41.91458	3.31078	5.079342	12.40806	2.9
155.602143	5.741776	388.9645	14.35293	32.74787	2.499737	4.100838	8.611157	2.8
156.459802	5.567964	600.5464	21.37176	49.20586	3.838343	6.106217	15.80379	3
153.591611	5.035791	495.1343	16.23391	38.15032	3.223707	4.63826	11.19812	3.1
153.21141	5.124127	580.1577	19.40327	45.3725	3.786648	5.543791	14.27914	3.2
146.84804	5.134547	730.8712	25.55494	59.09712	4.977058	7.301411	20.42039	3
167.972318	4.652973	354.3383	9.815465	24.05956	2.109504	2.804419	5.162493	3
192.993905	4.617079	650.8031	15.56945	39.58835	3.372143	4.448415	10.95237	2.8
228.770049	3.985541	927.9593	16.16654	44.49846	4.056297	4.619011	12.181	2.9
310.938039	3.350625	887.6912	9.565638	29.68937	2.854881	2.733039	6.215013	2.9
176.719188	5.122295	716.5428	20.76936	50.3359	4.054697	5.934102	15.64706	2.9
242.085188	3.87957	954.7894	15.30111	43.00502	3.944022	4.371746	11.42154	2.9
225.261185	4.095658	856.1449	15.56627	42.39116	3.800677	4.447506	11.47061	2.9
220.352023	4.213232	795.897	15.21792	40.92424	3.611934	4.347976	11.00468	2.9
212.398894	4.290887	723.4508	14.61517	38.76637	3.406095	4.175762	10.32428	2.9
234.586657	4.016895	1120.619	19.18868	53.04548	4.776994	5.48248	15.17179	2.9
235.349613	3.988976	1151.246	19.51264	54.07906	4.891642	5.575041	15.52367	2.8
210.755752	4.354458	1130.146	23.35012	61.58863	5.362349	6.671464	18.99567	2.8
183.592495	3.497	822.7431	15.6713	42.18371	4.481355	4.477513	12.1743	2.9
194.185503	3.269116	870.6725	14.65779	40.69256	4.483715	4.187939	11.38867	2.8
180.679638	3.919298	665.0738	14.42676	37.59189	3.680956	4.121932	10.50747	2.9
205.99426	3.274948	944.8575	15.02158	42.3037	4.586815	4.291881	11.74663	2.8
188.612058	3.423086	994.0572	18.04097	49.15273	5.27038	5.154562	14.61788	2.9

184.279277	3.783969	811.7684	16.66876	44.03372	4.405099	4.762502	12.88479	2.9
248.760275	2.502618	1082.297	10.8883	34.38006	4.350763	3.110943	8.385681	2.9
156.137439	4.833976	579.4978	17.94111	42.77105	3.71146	5.126031	13.10713	2.9
184.748213	4.336813	568.949	13.35561	34.12056	3.079591	3.815889	9.018798	2.9
159.570795	4.879841	967.6155	29.59069	70.76068	6.063863	8.454482	24.71085	2.9
310.938039	3.350625	831.0326	8.955093	27.79439	2.672663	2.558598	5.604467	3.2
169.038607	5.366305	599.1514	19.02068	45.06129	3.544465	5.43448	13.65437	3.2
246.067694	3.868989	972.5764	15.29208	43.1848	3.952475	4.369166	11.42309	3
188.070461	3.463544	974.1608	17.94035	48.7002	5.179765	5.125813	14.4768	3.2
156.446477	4.684026	670.2002	20.06587	48.2386	4.283894	5.733107	15.38185	3
179.517535	3.852308	925.3762	19.85786	51.88345	5.154796	5.673674	16.00555	3
205.99426	3.274948	1045.479	16.62129	46.80878	5.075282	4.748939	13.34634	3.2
201.645031	3.273458	993.2437	16.12409	45.1721	4.925704	4.606882	12.85063	3
195.597307	3.725663	730.0944	13.90656	37.43342	3.73264	3.973303	10.1809	3.1
178.578831	4.313498	1039.286	25.10353	63.67733	5.81976	7.172436	20.79003	3.2
245.674945	3.838671	1029.044	16.07881	45.47775	4.18864	4.593946	12.24014	2.8
164.7121	5.545862	498.4501	16.78283	39.17909	3.02619	4.795095	11.23697	2.9
243.884175	3.927281	929.7756	14.97223	42.03001	3.812365	4.277781	11.04495	2.9
308.605671	3.432766	2810.222	31.25942	96.25446	9.10619	8.931263	27.82666	2.8
245.207772	3.849416	881.5393	13.83892	39.09643	3.595071	3.953978	9.989506	2.9
288.0794	3.592012	2062.246	25.71379	76.95019	7.158603	7.346797	22.12178	2.8
199.037743	3.246945	1097.041	17.89626	50.07575	5.511723	5.113218	14.64932	2.8
187.610547	3.958028	1179.44	24.8827	65.28931	6.28664	7.109343	20.92467	2.9
114.767488	7.404354	207.5	13.3871	26.56252	1.808003	3.824885	5.982743	
128.558156	6.427908	218.133	10.90665	23.06476	1.696765	3.116186	4.478742	
120.926099	6.989948	233.833	13.51636	27.56584	1.933685	3.861817	6.52641	
150.938907	5.409997	361.191	12.94591	29.75321	2.392962	3.698833	7.535917	
131.952524	6.283454	313.42	14.92476	31.94937	2.375248	4.264218	8.641308	
129.932359	6.307396	368.699	17.89801	38.13042	2.837623	5.113717	11.59061	
119.194861	6.374057	172.757	9.238342	19.2112	1.449366	2.639526	2.864286	
145.527747	5.684678	325.923	12.73137	28.6375	2.239594	3.637533	7.04669	
194.013169	4.000272	408.859	8.430082	22.24679	2.107378	2.408595	4.429811	
151.468554	4.91781	348.309	11.30873	26.64107	2.299547	3.231067	6.390924	
133.742531	6.191784	365.583	16.92514	36.48763	2.733483	4.835754	10.73336	
131.523507	6.353793	298.741	14.43193	30.78343	2.271389	4.123409	8.07814	
130.10554	6.40914	370.571	18.25473	38.74801	2.848234	5.215637	11.84559	
144.196183	5.744868	567.75	22.61952	50.62929	3.937344	6.462721	16.87465	
134.343229	6.10651	436.833	19.85605	43.00297	3.251619	5.673156	13.74954	
133.433888	6.149027	331.261	15.26548	32.94773	2.482585	4.361567	9.116457	
137.766273	5.989838	327.578	14.24252	31.19025	2.377781	4.069292	8.252684	
157.636548	4.663803	262.176	7.756686	18.70275	1.663168	2.216196	3.092883	
147.699329	5.023787	389.903	13.26201	30.88134	2.639843	3.789145	8.23822	
178.592128	4.31382	490.994	11.85976	30.08333	2.749248	3.388502	7.545939	
146.162813	5.092781	348.086	12.12843	28.07211	2.381495	3.465266	7.035651	
140.364054	5.357407	471.7	18.00382	40.73239	3.360547	5.143948	12.64641	
171.487386	4.89964	498.429	14.24083	34.63796	2.906505	4.068808	9.341189	
176.52913	4.849701	448.244	12.3144	30.24743	2.539207	3.518399	7.464694	

179.277911	4.780744	629.691	16.79176	41.55316	3.512374	4.797646	12.01102
221.718148	4.191269	627.273	11.85771	31.979	2.829146	3.387918	7.666443
173.522638	4.92962	470.5	13.36648	32.55762	2.711462	3.818994	8.436857
158.887899	5.261189	246.667	8.167781	19.14723	1.552459	2.333652	2.906593
220.870941	3.827919	465.917	8.074818	22.25495	2.109454	2.307091	4.246899
197.481586	4.030236	376.583	7.685367	20.33357	1.906927	2.195819	3.655131
151.507251	5.242465	326.489	11.2972	26.1936	2.15494	3.227771	6.054732
184.074034	4.67193	1162.818	29.51315	73.94173	6.317121	8.432328	24.84122
162.682983	5.180987	465.764	14.83325	35.11306	2.863016	4.238071	9.652262
170.372392	4.996258	775.2	22.73314	54.93485	4.550033	6.495182	17.73688
159.003353	5.300112	442.917	14.7639	34.55265	2.785583	4.218257	9.463788
167.536458	5.031125	317.2	9.525526	22.88232	1.893319	2.721579	4.494401
257.584721	3.885139	672.842	10.14845	28.95863	2.612119	2.899556	6.263307
172.203913	4.906094	451.219	12.85524	31.29011	2.62026	3.672926	7.949148
292.237432	3.727518	669.88	8.544388	25.42494	2.292246	2.441254	4.816869
213.685751	4.28228	721.444	14.4578	38.42618	3.376191	4.130799	10.17552
226.836815	3.737015	781.1	12.8682	35.91825	3.443445	3.67663	9.131189
182.51803	4.244605	350.5	8.151163	20.87309	1.920358	2.328904	3.906557
153.514327	5.117144	357.429	11.9143	27.8836	2.32831	3.404086	6.797156
157.314865	4.870429	309.3	9.575851	22.82853	1.966121	2.735958	4.705422
189.383273	4.153142	434.113	9.520022	24.73883	2.292246	2.720006	5.36688
167.052601	4.679345	454.1	12.71989	31.09212	2.718305	3.634254	8.040543
245.816466	3.990527	449.194	7.29211	20.42906	1.827355	2.08346	3.301583
194.389483	4.531223	524.417	12.22417	31.28483	2.697764	3.492621	7.692949
242.610713	3.832713	430.067	6.794107	19.16388	1.772663	1.941174	2.961395
203.471042	4.357915	530.364	11.35926	29.69314	2.606582	3.245504	7.001348
198.063103	4.542732	530.417	12.16553	31.26098	2.67802	3.475865	7.622796
214.610367	4.232946	420.267	8.28929	22.1192	1.958279	2.368369	4.056344
209.538199	4.27629	569	11.61224	30.72311	2.715495	3.317784	7.335955
185.628677	4.910812	564	14.92063	36.99647	3.038324	4.263039	10.00982
151.749277	4.84822	570.7	18.23323	43.12704	3.760809	5.209493	13.38501
207.703998	2.841368	984.5	13.46785	39.38018	4.739918	3.847958	10.62648
195.320262	3.196731	550.746	9.013846	25.20115	2.819707	2.575385	5.817115
184.986116	3.477183	856.333	16.09648	43.47193	4.629174	4.598996	12.6193
168.265436	4.124153	341.615	8.372917	21.16127	2.030215	2.392262	4.248764
276.14743	3.540352	951.154	12.19428	36.23931	3.44437	3.484081	8.65393
293.69089	3.459257	705.918	8.3147	25.23908	2.403609	2.375628	4.855443
229.750171	4.023646	570.55	9.992119	27.46734	2.48335	2.854891	5.968473
205.372949	4.378954	724.25	15.44243	40.41186	3.526511	4.412123	11.06348
263.736767	3.693792	723.182	10.1286	29.44245	2.74206	2.893886	6.434807
190.445592	4.714	464.571	11.49928	28.99119	2.43939	3.285509	6.785282
164.791244	5.567272	544.917	18.40936	42.93995	3.306711	5.259817	12.84209
194.742507	3.44068	425.852	7.523887	20.63699	2.186744	2.149682	4.083207
201.473544	3.011563	702.667	10.50324	30.03862	3.487639	3.000927	7.491681
174.107944	3.627249	511	10.64583	28.02141	2.934961	3.041667	7.018585
177.308047	3.567566	258.185	5.194869	13.79316	1.456138	1.484248	1.627303
181.592387	3.526066	656.845	12.75427	34.16705	3.61714	3.644078	9.228206

170.331474	4.279685	439.923	11.05334	27.76287	2.582746	3.158098	6.773656
165.853508	4.364566	403.692	10.62347	26.37621	2.434028	3.035278	6.258908
271.41857	3.618914	672.214	8.962853	26.37616	2.476669	2.560815	5.343939
203.266826	4.418844	1015	22.06522	57.46424	4.993437	6.304348	17.64637
220.889429	4.183512	518.609	9.82214	26.47675	2.347822	2.806326	5.638628
317.918124	3.357108	1079.49	11.39905	35.55954	3.395497	3.256871	8.041942
228.007621	4.108245	1313	23.65766	64.57211	5.758579	6.759331	19.54941
373.202248	3.14408	1094.554	9.221179	30.43681	2.932871	2.634623	6.0771
236.389308	3.946399	719.375	12.0096	33.41067	3.043179	3.431314	8.0632
298.6018	3.436154	709.333	8.162635	24.92214	2.375515	2.332181	4.726481
179.299342	4.002217	340.935	7.610156	19.68852	1.901485	2.17433	3.607939
230.839599	2.674851	670.432	7.768621	23.67809	2.90432	2.219606	5.093771
179.119387	4.098842	350.071	8.010778	20.59658	1.9544	2.288794	3.911936
193.840307	3.550189	527.25	9.656593	26.24956	2.720023	2.759027	6.106405
206.50782	3.086813	534.306	7.986637	22.84128	2.58734	2.281896	4.899823
184.898395	3.742882	444.334	8.994615	23.84593	2.403125	2.56989	5.251733
179.616222	5.031267	317.25	8.886555	21.72203	1.766266	2.539016	3.855288
291.956722	3.463306	1117	13.2503	40.14972	3.82591	3.785799	9.78699
178.552764	5.015527	415.333	11.66666	28.49764	2.326108	3.333331	6.65113
176.325218	5.140677	387.75	11.30466	27.3578	2.199062	3.229904	6.163988
200.572107	4.51739	733.667	16.52403	42.6542	3.657872	4.721152	12.00664
195.221978	4.670382	424.364	10.15225	25.81406	2.173751	2.900643	5.481867
319.85469	3.314556	676.417	7.009503	21.96944	2.114763	2.002715	3.694946
219.379063	4.139228	652.9	12.31887	33.23837	2.976127	3.519677	8.17964
222.583067	4.264043	686.223	13.14603	35.33559	3.082997	3.75601	8.881991
237.698581	3.915957	861.506	14.19285	39.61565	3.624363	4.0551	10.27689
267.385403	3.698277	529.273	7.320512	21.34647	1.979439	2.091575	3.622235
330.764425	3.252354	771.529	7.586323	24.09137	2.332563	2.167521	4.333968
213.345108	2.926545	678.396	9.305844	27.19178	3.179806	2.658812	6.379299
175.841764	4.176764	689.7	16.38242	41.73003	3.922276	4.680692	12.20566
197.248749	3.673161	725.805	13.51592	36.58805	3.679643	3.861692	9.842761
262.725026	3.742522	892	12.70655	36.78003	3.395185	3.630444	8.964031
226.218072	4.173765	735.137	13.56341	36.80178	3.249683	3.875261	9.389648
306.156768	3.439964	829.067	9.31536	28.61193	2.707982	2.661531	5.875396
208.227704	4.374532	630.211	13.23973	34.77609	3.026547	3.782779	8.865195
262.942193	3.79426	637.077	9.19303	26.52418	2.422879	2.62658	5.398771
340.51532	3.286827	1186.477	11.45248	36.53756	3.484357	3.272137	8.165653
176.62647	3.839706	335	7.282609	18.96603	1.896658	2.080745	3.442903
244.463998	2.541206	644.769	6.70238	20.9905	2.63748	1.914966	4.161175
202.524052	3.38669	567.182	9.484649	26.37525	2.800566	2.7099	6.097959
191.647961	3.714108	483.7	9.374031	25.124	2.523898	2.678295	5.659923
222.652481	3.079564	612.846	8.476432	24.71711	2.752478	2.421838	5.396867
270.732384	2.352149	550.857	4.785899	15.6759	2.034692	1.3674	2.43375
186.423182	3.958029	377.437	8.013524	20.99321	2.024625	2.289578	4.055495
214.842899	2.971548	543.321	7.514813	21.91305	2.528922	2.14709	4.543266
243.991273	3.954478	477.357	7.736742	21.6835	1.956451	2.210498	3.782265
339.642295	3.281568	694.231	6.707546	21.39433	2.044006	1.916442	3.425978

240.736608	3.946502	585.64	9.600656	26.83078	2.4327	2.743044	5.654154
268.55718	3.699135	802.194	11.0495	32.25351	2.987051	3.157001	7.350369
247.475153	3.848758	714.923	11.11855	31.48481	2.888868	3.17673	7.269795
242.044659	3.954978	638.077	10.42609	29.16148	2.636195	2.978884	6.471117
313.051852	3.373404	720.413	7.763071	24.09465	2.301258	2.21802	4.389668
242.374337	3.97335	1255.091	20.57526	57.50132	5.178316	5.878646	16.60191
192.667655	3.726647	665.462	12.87161	34.5148	3.453937	3.677602	9.144958
204.639225	3.279475	393.986	6.313878	17.74567	1.925271	1.803965	3.034403
189.633834	3.632832	564.8	10.81992	29.08317	2.978371	3.091407	7.187091
243.615445	2.668296	1467.077	16.06875	49.67065	6.022102	4.591072	13.40046
242.478256	2.604493	475.962	5.112374	15.88035	1.962906	1.460678	2.507881
341.991263	3.250868	776.817	7.384192	23.64868	2.271453	2.109769	4.133324
264.655568	3.748662	653.529	9.256785	26.83251	2.469357	2.644796	5.508122
233.898653	4.089137	606.538	10.60381	29.16158	2.593166	3.02966	6.514674
380.344896	3.104856	696.056	5.68209	18.9035	1.830065	1.623454	2.577234
279.473384	2.136647	741.267	5.667179	19.16544	2.652371	1.619194	3.530532
189.675544	3.959823	502.266	10.48572	27.58558	2.648027	2.99592	6.525897
282.814355	3.658659	785.8	10.16559	30.14237	2.778501	2.904454	6.506929
262.622647	3.762502	707.846	10.14106	29.31212	2.695297	2.897446	6.378558
280.092904	3.656565	689.018	8.995013	26.61086	2.459962	2.570004	5.338448
271.727983	3.662102	753.672	10.1573	29.8112	2.773627	2.902087	6.495202
253.187634	3.859568	561.182	8.554604	24.3459	2.216467	2.444172	4.695036
358.923586	3.181947	756.2	6.703901	21.84766	2.106855	1.9154	3.521954
195.145513	3.717057	376.714	7.175505	19.31489	1.930426	2.050144	3.458447
186.784826	3.991129	617.111	13.18613	34.48886	3.30386	3.767466	9.195004
215.370824	2.902572	462.543	6.233733	18.29571	2.147659	1.781067	3.331161
217.539297	2.923915	621.615	8.35504	24.53815	2.857484	2.387154	5.431125
343.655198	3.254311	590.682	5.59358	17.93105	1.718822	1.598166	2.339269
298.877318	3.451239	796.583	9.198418	28.06032	2.665251	2.628119	5.747179
193.53231	3.721775	472.466	9.085885	24.39878	2.441277	2.595967	5.364109
184.098282	3.749456	311.429	6.342749	16.78989	1.691645	1.812214	2.593294
146.84804	5.134547	670.0263	23.42749	54.1773	2.403853	6.693569	18.29295
144.461819	5.451389	282.4327	10.65784	24.18135	2.788332	3.045096	5.206448
153.591611	5.035791	432.3341	14.17489	33.31153	2.46143	4.049968	9.139098
145.743192	5.715419	924.1029	36.23933	81.43577	4.232343	10.35409	30.52391
160.892602	5.20688	440.5377	14.25688	33.61354	2.247917	4.073395	9.050003
150.569116	5.556056	305.0868	11.25782	25.686	1.730466	3.216519	5.70176
139.872091	5.952004	428.8774	18.2501	40.18207	3.093383	5.214315	12.2981
151.812778	5.40259	501.1049	17.83291	41.05811	4.46733	5.095118	12.43032
153.21141	5.124127	242.4666	8.109251	18.96263	1.366031	2.316929	2.985123
157.31592	4.931534	305.8236	9.586947	22.7839	1.42492	2.739128	4.655413
228.770049	3.985541	605.1789	10.54319	29.02016	1.509178	3.012339	6.557645
192.993905	4.617079	871.9852	20.86089	53.04286	4.518201	5.960254	16.24381
174.066748	5.211579	392.5361	11.75258	28.25334	1.474169	3.357879	6.540999
242.085188	3.87957	322.3462	5.165804	14.51891	1.199147	1.475944	1.286234
310.938039	3.350625	917.6648	9.888629	30.69185	2.198184	2.825323	6.538004
176.719188	5.122295	580.9629	16.8395	40.81164	2.659865	4.811287	11.71721

183.592495	3.497	611.232	11.64251	31.33911	3.186579	3.326433	8.145514
188.070461	3.463544	427.4128	7.871322	21.3672	1.789736	2.248949	4.407778
193.799292	3.347138	598.4921	10.33665	28.51343	3.127622	2.953329	6.989513
205.99426	3.274948	818.2643	13.00897	36.63579	3.800387	3.716849	9.734023
184.87348	3.843523	438.0895	9.10789	23.98579	2.13822	2.602254	5.264366
188.612058	3.423086	421.1166	7.64277	20.82278	3.168792	2.183648	4.219683
180.679638	3.919298	513.8049	11.14544	29.04173	2.05603	3.184412	7.226145
186.252389	4.825191	273.6664	7.089803	17.67181	1.663648	2.025658	2.264612
229.895689	2.594759	659.8667	7.447705	22.84972	3.144389	2.127916	4.852946
156.446477	4.684026	262.8922	7.871024	18.92204	1.781344	2.248864	3.186998
184.279277	3.783969	516.9365	10.61471	28.0408	2.294069	3.032775	6.830744
194.185503	3.269116	794.4434	13.37447	37.12984	3.399839	3.821277	10.10535
179.517535	3.852308	214.1619	4.595749	12.0075	1.168665	1.313071	0.743441
206.277689	3.238268	197.2048	3.095837	8.746069	0.918901	0.884525	-0.14243
231.578334	4.055663	455.1232	7.970634	21.91048	1.93772	2.277324	3.914971
164.7121	5.545862	405.1771	13.64233	31.84766	1.591521	3.897808	8.096465
245.674945	3.838671	1020.278	15.94184	45.09034	3.419925	4.554813	12.10317
252.003448	3.761245	593.1829	8.853476	25.32984	1.557237	2.529565	5.092231
200.021626	3.496882	1053.482	18.41752	50.65008	5.731616	5.262148	14.92064
210.179427	2.715496	500.2642	6.463362	19.17095	2.392495	1.846675	3.747865
186.848674	3.908968	330.5093	6.914421	18.18079	1.097849	1.975549	3.005452
233.432702	4.059699	824.1821	14.3336	39.47047	2.989658	4.095315	10.2739
